# rDock: A Fast, Versatile and Open Source Program for Docking Ligands to Proteins and Nucleic Acids

**DOI:** 10.1371/journal.pcbi.1003571

**Published:** 2014-04-10

**Authors:** Sergio Ruiz-Carmona, Daniel Alvarez-Garcia, Nicolas Foloppe, A. Beatriz Garmendia-Doval, Szilveszter Juhos, Peter Schmidtke, Xavier Barril, Roderick E. Hubbard, S. David Morley

**Affiliations:** 1 Departament de Fisicoquímica, Facultat de Farmàcia, Universitat de Barcelona, Barcelona, Spain; 2 Institut de Biomedicina de la Universitat de Barcelona (IBUB), Barcelona, Spain; 3 Vernalis (R&D) Ltd, Granta Park, Cambridge, United Kingdom; 4 Amper Programas, Madrid, Spain; 5 Omixon Biocomputing, Budapest, Hungary; 6 Discngine, Paris, France; 7 Catalan Institution for Research and Advanced Studies (ICREA), Barcelona, Spain; 8 YSBL, University of York, Heslington, York, United Kingdom; 9 Enspiral Discovery Limited, Cambridge, United Kingdom; 10 Ariana Pharma, Paris, France; UCSD, United States of America

## Abstract

Identification of chemical compounds with specific biological activities is an important step in both chemical biology and drug discovery. When the structure of the intended target is available, one approach is to use molecular docking programs to assess the chemical complementarity of small molecules with the target; such calculations provide a qualitative measure of affinity that can be used in virtual screening (VS) to rank order a list of compounds according to their potential to be active. rDock is a molecular docking program developed at Vernalis for high-throughput VS (HTVS) applications. Evolved from RiboDock, the program can be used against proteins and nucleic acids, is designed to be computationally very efficient and allows the user to incorporate additional constraints and information as a bias to guide docking. This article provides an overview of the program structure and features and compares rDock to two reference programs, AutoDock Vina (open source) and Schrödinger's Glide (commercial). In terms of computational speed for VS, rDock is faster than Vina and comparable to Glide. For binding mode prediction, rDock and Vina are superior to Glide. The VS performance of rDock is significantly better than Vina, but inferior to Glide for most systems unless pharmacophore constraints are used; in that case rDock and Glide are of equal performance. The program is released under the Lesser General Public License and is freely available for download, together with the manuals, example files and the complete test sets, at http://rdock.sourceforge.net/

This is a *PLOS Computational Biology* Software Article.

## Introduction

The discovery of small molecules with biological activities is important to probe biological mechanism in chemical biology and to provide drug candidates as potential therapeutic agents. The first step in this process is to identify compounds that bind to a specific target (hits); experimentally this is usually achieved with high-throughput (HTS) or fragment screening (FS). The resulting hits are then optimised to higher affinity compounds, usually guided by a model of how the compounds bind to the target, increasingly with crystal structures of the target used to guide the optimisation.

Computational methods are often used as a central part of this process. Molecular docking can play an important role in the optimisation, where a proposed position and conformation (so-called pose) of the compound can be generated and provide useful models for how the compounds are binding, in advance of any experimental structure determination. However, if the structure of the target is known and a druggable cavity has been identified [1], molecular docking can also be used to screen virtual chemical collections to identify those molecules that offer good shape and chemical complementarity [2]. Such virtual screening (VS) offers opportunities for small research groups without access to HTS or FS to identify new hit compounds, as setting up a low-throughput assay to test a few tens of compounds is relatively fast and inexpensive. Such VS has been successful, but it requires a docking program that is computationally efficient and can be finely tuned to achieve optimal performance [3]–[5]. rDock is a molecular docking platform which has been optimised for such tasks.

rDock has its origins in the program RiboDock [6], designed initially for VS of RNA targets. Developed at the company now known as Vernalis (http://www.vernalis.com), the software, scoring functions, and search protocols have been refined continuously over a number of years to meet the demands of in-house discovery projects on heat-shock proteins [7]–[9], kinases [10]–[13] and other targets. The major components of the platform now include fast intermolecular scoring functions (vdW, polar, desolvation) validated against protein and RNA targets, a Genetic Algorithm (GA)-based stochastic search engine, a wide variety of external restraint terms (tethered template, pharmacophoric restraints), and novel Genetic Programming-based post-docking filtering [14]. In this paper we describe the platform, benchmark it against two other state of the art docking programs for both binding mode prediction and VS and discuss its use in high-throughput VS (HTVS).

### Design and implementation

The rDock platform is a collection of command-line programs and scripts (Table 1 and Figure S1). The main tasks are carried out by the programs *rbcavity* (cavity generation) and *rbdock*(docking). rDock is written in ANSI C++ and compiles under the Linux operating system using the GNU g++ compiler. Apart from the C++ Standard Template Library (STL) there are minimal external dependencies (*e.g.* OpenBabel bindings for running *sdtether* and *sdrmsd* [15]). The core functionality is compiled into a single shared library, which is linked with each of the (light-weight) command-line applications. Scoring functions and docking protocols are assembled at run-time from well-defined C++ object class hierarchies, allowing for customisation at source code level by extending the base classes. Ancillary scripts are provided for file management and output processing and are described in the manuals.

**Table 1:** List of main programs and utilities included in the rDock package.

Name	Language	Use	Description
rbdock	C++	Docking	The main rDock docking engine
rbcavity	C++	Cavity definition	Cavity mapping and preparation of docking site (.as file).
rbcalcgrid	C++	Preparation	Calculation of vdW grid files (usually called by make_grid.csh wrapper script)
sdtether	python	Preparation	Prepares a ligand SD file for tethered scaffold docking, annotating the atom indices of the tethered substructure. Requires OpenBabel python bindings [15]
sdrmsd	python	Analysis	Calculation of ligand Root Mean Squared Displacement (RMSD) between reference and docked poses, taking into account ligand topological symmetry. Requires OpenBabel python bindings [15]
sdfilter	perl	Analysis	Utility for filtering SD files by arbitrary data field expressions. Useful for simple post-docking filtering by score components.
sdsort	perl	Analysis	Utility for sorting SD files by arbitrary data field. Useful for simple post-docking filtering by score components.
sdreport	perl	Analysis	Utility for reporting SD file data field values in tab-delimited or CSV format.

### Preparation

The receptor is provided in Tripos MOL2 format with standard atom typing. Amino acid ionisation states in the vicinity of the cavity must be defined, as the rDock scoring functions depend on formal charge assignments. Metal ions, cofactors and structural water molecules can be included as part of the receptor. The user should also resolve other structural issues such as alternate locations or missing atoms. The docking volume is defined by the *rbcavity* program which provides two mapping algorithms; the accessible volume within a specific distance of a reference ligand, and a two probe sphere method [6]. In the examples presented in this paper, the reference ligand method is used with a distance of 6 Å.

Ligands to be docked are read in the MDL SDFile format (SDF) and should have the correct topology and bond orders. The program can protonate and deprotonate certain ionisable groups, but pre-processing the ligands with a dedicated program is preferable. Since the program only samples exocyclic dihedral angles, a correct input geometry is required for bonds, angles and rings. In the case of flexible rings, a variety of low-energy conformers should be pregenerated by a suitable program. We have used LigPrep [16] for all ligand preparation steps. The execution of the programs is controlled by a series of parameter (.prm) files; this allows user controlled tuning of the docking protocol and scoring functions (described in more detail in the Manual). The following sections describe the main characteristics of the program and the available options.

### Scoring

The rDock master scoring function (S^total^) is a weighted sum of intermolecular (S^inter^), ligand intramolecular (S^intra^), site intramolecular (S^site^), and external restraint terms (S^restraint^). S^inter^ is the main term of interest as it represents the protein-ligand (or RNA-ligand) interaction score. S^intra^ reports the change in energy of the ligand relative to the input ligand conformation. Similarly, S^site^ represents the relative energy of the flexible regions of the active site. In the current implementation, the only flexible bonds in the active site are terminal OH and NH_3_^+^ bonds. S^restraint^ is a collection of non-physical restraint functions that can be used to bias the docking calculation in several useful ways (*vide infra*). S^inter^, S^intra^, and S^site^ are built from a common set of constituent potentials, which are described in the Manual. Briefly, they mainly consist of a van der Waals potential (vdW), an empirical term for attractive and repulsive polar interactions, and an optional desolvation potential that combines a weighted solvent accessible surface area approach [17] with a rapid probabilistic approximation to the calculation of solvent accessible surface areas [18] for computational efficiency. The vdW term can be calculated during docking, or precalculated and stored on grid files by the ancillary program *rbcalcgrid*; this increases computational performance. Two distinct scoring functions have been optimized using a binding affinity validation set (described in the Manual). The default scoring function (SF3) uses the repulsive polar term but not the desolvation term, while the solvation scoring function (SF5) does the opposite. The default SF3 is slightly faster and works better for proteins while the solvation term is generally better for nucleic acids. More importantly, the weighting terms of the scoring function can be re-optimized with larger or more focused validation sets to improve its performance.

### Sampling

rDock uses a combination of stochastic and deterministic search techniques to generate low energy ligand poses. The standard docking protocol to generate a single ligand pose uses 3 stages of Genetic Algorithm search (GA1, GA2, GA3), followed by low temperature Monte Carlo (MC) and Simplex minimization (MIN) stages. The GA stages are interdependent and are designed to be used sequentially. Several scoring function parameters are varied between the stages to promote efficient sampling of the starting poses, whilst minimising the likelihood that the poses become trapped early in the search. The variations are in the functional form of the S^inter^ vdW potential (switched from 4–8 potential in GA1/GA2 to 6–12 potential in GA3/MC/MIN), the tolerances on the polar distance and angular functions (relaxed in GA1 and progressively tightened in GA2/GA3/MC), and the weight of the ligand dihedral potential (reduced in GA1 and progressively increased in GA2/GA3/MC). All scoring function parameters are at their final reported values for the final MC/MIN stages. The GA chromosome consists of the ligand centre of mass (COM), the ligand orientation, as represented by the Euler angles (heading, attitude, bank) required to rotate the ligand principal axes from the Cartesian reference axes, the ligand rotatable dihedral angles, and the receptor rotatable dihedral angles. The initial population is generated such that the ligand COM lies on a randomly selected grid point within the defined docking volume, and the ligand orientation and all dihedral angles are randomised. Mutations are applied to a randomly selected degree of freedom and the magnitude of the mutation is selected from rectangular distributions of defined width. A generation is considered to have passed when the number of new individuals created is equal to the population size. Instead of having a fixed number of generations, the GA is allowed to continue until the population converges (scoring improvement <0.1 units over the last three generations). This allows early termination of poorly performing runs for which the initial population is not able to generate a good solution. Once the GA converges, a low temperature Monte Carlo simulation is used to refine the pose, followed by Simplex routine to generate a minimised solution. A more detailed description of the sampling protocol can be found in the Manual. In a typical docking calculation, the whole process is repeated 10 to 100 times and the overall lowest scoring pose is taken as the correct solution (see below for discussion on convergence), but it is also possible to access the minimisation stage directly or simply score a pre-docked pose.

### Biased docking

The main limitation in molecular docking is the quality of the scoring functions. It is therefore usual to introduce empirical bias, which can improve the quality of the results and also reduce the search space, thus improving performance. rDock implements several pseudo-energy scoring functions that are added to the total scoring function under optimisation, and a restricted search protocol.

#### Pharmacophoric restraints

This feature ensures that pharmacophores (derived from known ligands or hot-spot mapping methods) are satisfied by all generated poses. rDock recognizes nine feature types: neutral hydrogen bond acceptor, neutral hydrogen bond donor, hydrophobic, hydrophobic aliphatic, hydrophobic aromatic, negatively charged, positively charged, and any heavy atom. Each pharmacophore restraint is defined by a combination of feature type and position, specified as a tolerance sphere with coordinate (x,y,z), and radius (r). Restraints are classified as either mandatory or optional, where the user can specify how many optional restraints (N_opt_) should be met. Ligands that have insufficient quantities of the defined restraint feature types are removed prior to docking. The penalty score for a single pharmacophore restraint is proportional to the square of the distance from the nearest ligand feature of the required type to the surface of the tolerance sphere, and is zero when the nearest ligand feature is within the tolerance sphere. The total pharmacophore restraint score, S^ph4^, is the sum of all the mandatory restraints plus the N_opt_ lowest scoring optional restraints.

#### Tethered template

Tethered template docking can be used to enforce partial binding modes obtained from crystal structures of related molecules or constituent fragments. The template is defined by a reference bound ligand structure and a SMARTS query string defining the substructure to be tethered. The *sdtether* utility prealigns molecules with matching substructures with the reference substructure coordinates prior to docking. Non-matching molecules are rejected. Molecules that have more than one substructure match with the query are replicated within the library of compounds to be docked, and each replicate prealigned and docked individually, thus ensuring that all possible substructure alignments are examined. In this mode, the centre of mass and principal axes of the tethered substructure, rather than the whole molecule, define the ligand position and orientation. Dihedral angle mutations operate exclusively on the free (untethered) end of each ligand rotatable bond, ensuring the tethered substructure coordinates remain unchanged. Some movement of the tethered region is allowed up to user-defined maximum deviations from the reference coordinates for ligand translation (typically 0.1 Å) and ligand rotation (typically 1°). For greater sampling efficiency, tethering in rDock is enforced absolutely during pose generation by restricting the randomisation and mutation functions for the tethered degrees of freedom, rather than through the use of an external penalty function.

#### Other

1) To ensure that all poses are contained wholly within the defined docking volume, a cavity penalty function (S^cavity^) is calculated over all non-hydrogen ligand atoms. If the atom is within the docking volume this term is zero, else, it is proportional to the square of the distance to the nearest docking volume grid point.2) When experimental NMR distance limits (NOE or STD) are known for a specific ligand, restraints can be used to ensure that a minimum distance is fulfilled between an atom (or group of atoms) of the ligand and an atom (or group of atoms) of the receptor.

## Results

### Benchmarking

The performance of rDock was compared with that of Glide (version 57111 [19]) and AutoDock Vina [20] for database enrichment and binding mode prediction for various test sets. As detailed in Supporting Information Text S1, all receptors, docking cavities and ligands were prepared in the same manner and running parameters modified to ensure exhaustive sampling by all programs.

#### Protein-ligand binding mode predictions

The CCDC-Astex Diverse Set of 85 diverse protein-ligand complexes was selected for comparing binding mode prediction [21]. The results, represented by percentage of correct predictions (ligand RMSD below 2 Å) can be seen in Table 2. rDock calculations converge after 20–50 GA runs (Figure S2; convergence also discussed below). The predicted binding mode is correct in approximately 80% of cases for rDock and Vina, while Glide's performance is close to 70%. Failures for rDock and Vina are due to scoring errors, as a correct pose is nearly always generated (99% and 97% of times, respectively). However, Glide fails to sample the correct binding mode in 16% of cases. Figure S3 shows the docking outcome for each system and program. Although no obvious trend can be identified, it would seem that rDock and Vina have a higher coincidence in the type of systems for which they succeed or fail.

**Table 2:** Percentage of top-ranked poses with an RMSD below 2 Å.

	% Correct (top 1)	% Correct (all)
**rDock**	76±3^1^	99±0.2^1^
**Glide**	67.6	83.8
**Vina**	81.2±2^1^	97±0.5^1^

1 Average and standard deviation taking 100 random sets of 100 docking poses out of a pool of 1000 solutions.

#### RNA-ligand binding mode predictions

We selected 56 RNA-ligand complexes from the original RiboDock [6] and DOCK6 [22] sets to assess the performance of rDock with RNA as the receptor. RNA structures are more challenging than proteins (less closed cavities, less hydrophobic, featureless) and the ligands themselves are larger and more flexible (7.7±4.3 rotatable bonds vs. 5.1±3.1 for the Astex set). For this reason the success cut-off criterion is an RMSD below 2.5 Å, relative to the crystal structure. The scoring function SF5, which includes a solvation term, is better for RNA than SF3, as independently assessed [23]. After 50 GA runs, the top-ranked docking solution is correct in 54±3% of the systems (Figure S4), and at least one correct pose is generated in 98% of cases, confirming that as with proteins, errors are attributable to scoring rather than sampling problems. However, both SF3 and SF5 have been primarily optimized for proteins suggesting that development of an RNA-specific scoring function could result in improvements. Vina and Glide can work with but have not been optimised for ligand docking to RNA. On the same set of complexes, we obtain success rates of 29±2 for Vina and 17.8 for Glide.

#### Virtual screening (DUD)

VS enrichment was assessed using the DUD benchmark set [24] which consists of 39 protein-ligand complexes with crystal structure, with an average of about 100 known active ligands per complex and 36 decoys per active ligand. The decoys are physically similar but topologically dissimilar to the ligands in order to avoid bias. The DUD-E benchmark set [25] was published recently, adding more protein-ligand complexes. For our test set, 20 of the original DUD sets were substituted with DUD-E data with more ligands and decoys per system. Figures S5 and S6 show the ROC curves for all systems and the most relevant parameters are summarized in Table S1. The results are summarised in Table 3. Using most metrics, Glide outperforms the other programs in ∼70% of the systems, while rDock is better in ∼20% of systems and Vina in the remaining 10%. On average, rDock AUC is 11% lower than Glide and 5% better than Vina. In terms of logAUC, on average, Glide outperforms rDock by 30%, while rDock outperforms Vina by 8%.

**Table 3:** Average values of different VS performance metrics over the 39 DUD/DUD-E systems.

Program	AUC^1^	logAUC^2^	EFmax^3^	EF 1%^4^	EF 20%^4^
rDock	0.69	0.26	98.7	11.4	2.5
	(18%)	(18%)	(33%)	(19%)	(18%)
Glide	0.78	0.37	334.6	22.6	3.2
	(69%)	(72%)	(41%)	(69%)	(72%)
Vina	0.66	0.24	124.3	8.9	2.2
	(13%)	(10%)	(26%)	(11%)	(10%)

The values in parentheses indicate the percentage of systems for which the program provides the optimal performance on a given metric.

1 Area Under the ROC Curve.

2 Area Under the semilogarithmic ROC Curve.

3 Maximal Enrichment Factor.

4 Enrichment Factor when the top x% of the virtual collection is selected.

### Sampling exhaustiveness and computing performance

**Figure 1:**
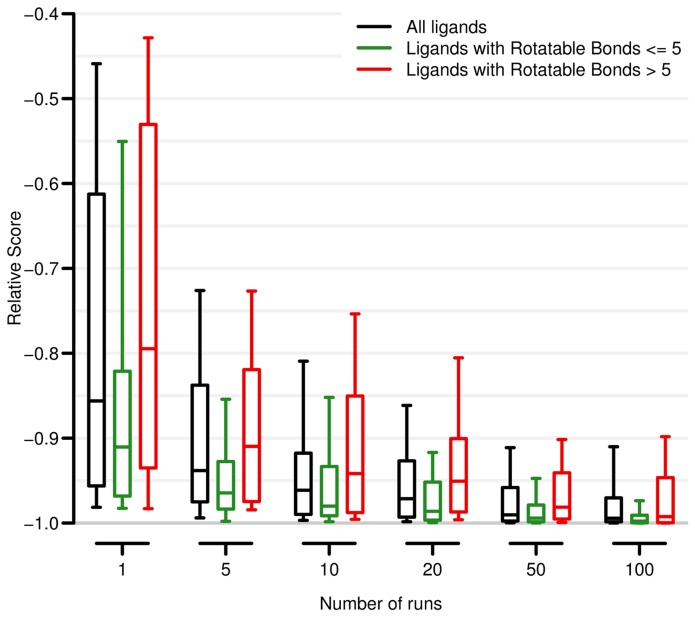
Relative score vs. the number of docking runs for all the protein-ligand complexes in the CCDC-Astex set. The boxplot indicates the median value (out of 1000 possible solutions) and the first and last quartile, while the whiskers span the 10% to 90% range. The whole set (black) has been sub-divided into ligands with 5 or fewer rotatable bonds (green) and the rest (red).

A distinctive feature of rDock is that the GA converges very quickly. This behaviour was designed for VS, where it is important to discard poor ligands early on. Multiple docking runs (which includes GA optimisation followed by MC and Simplex minimisation) are necessary to reach the global minimum score (S_min_), but few docking runs are necessary to reach a similar score (Figure 1). For instance, after 5 runs, approximately 80% of ligands reach a score of 0.8*S_min_, and the median value is 0.94*S_min_. Convergence depends on the dimensionality of the problem and fewer docking runs are necessary when the ligands contain fewer rotatable bonds (Figure 1) or when the cavity has a smaller size (Figure S7). System-specific multi-step HTVS protocols (see section below and Manual) achieve optimal performance with an average of 8–10 runs per ligand. Table 4 shows the average computing times per ligand on 4 DUD systems [24]. Precalculating the van der Waals potentials on a grid saves 20% to 40% of docking computing time, depending on the system. For exhaustive docking, rDock is approximately 5-fold faster than Vina, but still 8-fold slower than Glide SP. HTVS protocols achieve a further reduction of 5 to 8-fold in computing time, bringing the performance of rDock to be very similar to Glide SP with no negative impact on the results (Table S3). Using a relatively modest 100-core computing facility, a VS campaign of 1 million compounds can be completed in less than 1 day and the 21 million commercially accessible compounds compiled in ZINC database [26] could be screened in 10 to 20 days for most systems.

**Table 4:** Average computing times (in seconds per ligand) on 4 DUD systems.

	Vina^1^	Glide SP^1^	rDock			
			Grid-based SF		Indexed SF	
			VS^2^	Full^1^^,^ 3	VS^2^	Full^1^^,^ 3
ADA	86.4	4.2	4.2	27.0	5.4	33.0
COMT	77.4	3.0	3.0	22.5	5.0	31.8
PARP	54.0	1.5	3.9	16.5	5.7	29.1
Trypsin	372.0	6.0	14.1	53.1	20.1	82.5
**Average**	**147.5**	**3.7**	**6.3**	**29.8**	**9.1**	**44.1**

1 Default program parameters were used.

2 On HTVS mode, the average number of docking runs needed for these 4 systems is 10.

3 50 docking runs are used for default docking.

All figures were obtained on Intel Xeon X5660 CPUs at 2.80 GHz.

### Considerations for real VS applications

#### Design of multi-step HTVS protocols

Different docking protocols are required for different applications. For detailed docking, where the user is interested primarily in high accuracy, a suggested rDock protocol is to allow receptor flexibility, bypass the pre-calculation of van der Waals potentials and perform exhaustive sampling (50–100 GA runs). For HTVS applications, where computing performance is important, the recommended rDock protocol is to limit the search space (i.e. rigid receptor), apply the grid-based scoring function and to use a multi-step protocol to stop sampling of poor scorers as soon as possible. An example is for the DUD system COMT, where the computational time can be reduced by 7.5-fold without affecting performance by: 1) 5 GA runs for all ligands; 2) ligands achieving a score of −20 or lower run 10 further GAs; 3) for those ligands achieving a score of −25 or lower, continue until 50 GAs. The optimal protocol is specific for each particular system and parameter-set, but can be identified with a purpose-built script (see Manual).

#### Guided docking

Usually, VS applications exploit existing information to optimize the cavity definition (e.g. choice of protein conformation, displaceable water molecules) and to bias the docking protocol with empirical restraints (e.g. pharmacophoric points, shape similarity). This is an essential step common to all successful docking-based VS undertakings [3], [27]. For this reason, we have compared the outcome of VS on Hsp90, a DUD system for which we have developed and used optimal docking protocols [7], [8], [28]. The cavity includes 2 interstitial water molecules and two pharmacophoric points. As shown in Table 5 and Figures S8 and S9, all VS performance metrics improve significantly, particularly those related to early enrichment (logAUC, EF1%). As scoring functions are supplemented with empirical information, performance increases and the difference between programs reduce (Table S2).

**Table 5:** VS performance metrics for Hsp90 using an unbiased protocol with default parameters (rDock, Glide & Vina) or an optimized cavity definition and empirical pharmacophoric restraints (rDock-guided & Glide-guided).

Program	AUC	logAUC	EFmax	EF 1%	EF 20%
rDock	0.63	0.20	3.9	0.0	1.5
	*(0.8)*	*(0.7)*	*(0.5)*	*(1.0)*	*(0.7)*
Glide	0.77	0.28	7.4	0.0	2.1
	*(1.0)*	*(1.0)*	*(1.0)*	*(1.0)*	*(1.0)*
Vina	0.55	0.16	1.4	0.0	0.75
	*(0.7)*	*(0.6)*	*(0.2)*	*(1.0)*	*(0.4)*
rDock-guided	0.92	0.46	36.9	12.3	4.3
	*(1.2)*	*(1.6)*	*(5.0)*	*(–)*	*(2.0)*
Glide-guided	0.90	0.46	17.4	6.9	4.6
	*(1.2)*	*(1.6)*	*(2.3)*	*(–)*	*(2.2)*

Note that Vina does not support pharmacophoric restraints. The numbers in parentheses indicate performance relative to the best non-guided result (Glide).

### Availability and future directions

The program is released under the Lesser General Public License and the source code, scripts, manuals, and test sets are available at http://rdock.sourceforge.net/. The current version has prototype code to sample fully the degrees of freedom and occupancy of interstitial water molecules, as previously described for GOLD [29], or to dock simultaneously to an ensemble of receptor coordinates to simulate receptor flexibility in an efficient way. These features require further validation. Future developments will aim at improving the scoring functions for both protein-ligand and RNA-ligand interactions.

## Supporting Information

Figure S1 Workflow summary of an rDock docking job. Shapes in gray background are not covered with any rDock program and must be carried out with independent software.
(TIF)

Figure S2 Binding mode prediction in the protein-ligand set (CCDC-Astex): Percentage of top-ranked poses with RMSD below 2.0 Å as a function of the number of docking runs. The boxplot indicates the median value (out of 100 possible solutions) and the first and last quartile, while the whiskers span the 10% to 90% range. The whole set (black) has been sub-divided into ligands with 5 or fewer rotatable bonds (green) and the rest (red).
(TIF)

Figure S3 Matrix representation of the docking outcome for each system in the CCDC-Astex set for the three programs evaluated. A black area indicates that the best-scoring pose for a particular system-program combination has an RMSD below 2.0 Å.
(TIF)

Figure S4 Binding mode prediction in the RNA-ligand set: Percentage of top-ranked poses with RMSD below 2.5 Å as a function of the number of GA runs. The boxplot indicates the median value (out of 100 possible solutions) and the first and last quartile, while the whiskers span the 10% to 90% range.
(TIF)

Figure S5 Receiver Operating Characteristic (ROC) Curves of all DUD systems. In the Y-axis, the true positive rate is the fraction of true positives out of the total actual positives and, in the X-axis, the false positive rate is the fraction of false positives out of the total actual negatives. In gray, ROC curve in case of random results.
(TIF)

Figure S6 Semilogarithmic Receiver Operating Characteristic (ROC) Curves of all DUD systems. In the Y-axis, the true positive rate is the fraction of true positives out of the total actual positives and, in the X-axis in logarithmic scale, the false positive rate is the fraction of false positives out of the total actual negatives. In gray, semilogarithmic ROC curve in case of random results.
(TIF)

Figure S7 Relative score vs. the number of docking runs for all the protein-ligand complexes in the CCDC-Astex set. The boxplot indicates the median value (out of 100 possible solutions) and the first and last quartile, while the whiskers span the 10% to 90% range. The whole set (black) has been sub-divided into systems with relatively small cavities (green) and the rest (red).
(TIF)

Figure S8 ROC curve of HSP90 without pharmacophoric restraints in normal (A) or semilogarithmic scale (B).
(TIF)

Figure S9 ROC curve of HSP90 with pharmacophoric restraints in normal (A) or semilogarithmic scale (B). It should be noted that using these settings, Glide only produces an output for 13 actives (out of 24) and 451 decoys (out of 864).
(TIF)

Software S1 Compressed file with the source code of the rDock software for ligand docking to Proteins and Nucleic Acids.
(GZ) Click here for additional data file.

Table S1 Summary of statistics for all DUD systems and averages for each and all programs.
(DOCX)

Table S2 Spearman's rank correlation coefficient (ρ) between programs on the Hsp90 DUD set.
(DOCX)

Table S3 AUC for the 4 DUD systems used for calculating the time performance.
(DOCX)

Text S1 Supporting Methods: Test set preparation, execution and analysis.
(DOCX)

Text S2 Full Acknowledgements.
(DOCX)
