# Supplementary figures and images for: rDock: A Fast, Versatile and Open Source Program for Docking Ligands to Proteins and Nucleic Acids
Source: PLoS Comput Biol. 2014 Apr 10;10(4):e1003571. doi: 10.1371/journal.pcbi.1003571 (PMC3983074; doi:10.1371/journal.pcbi.1003571)

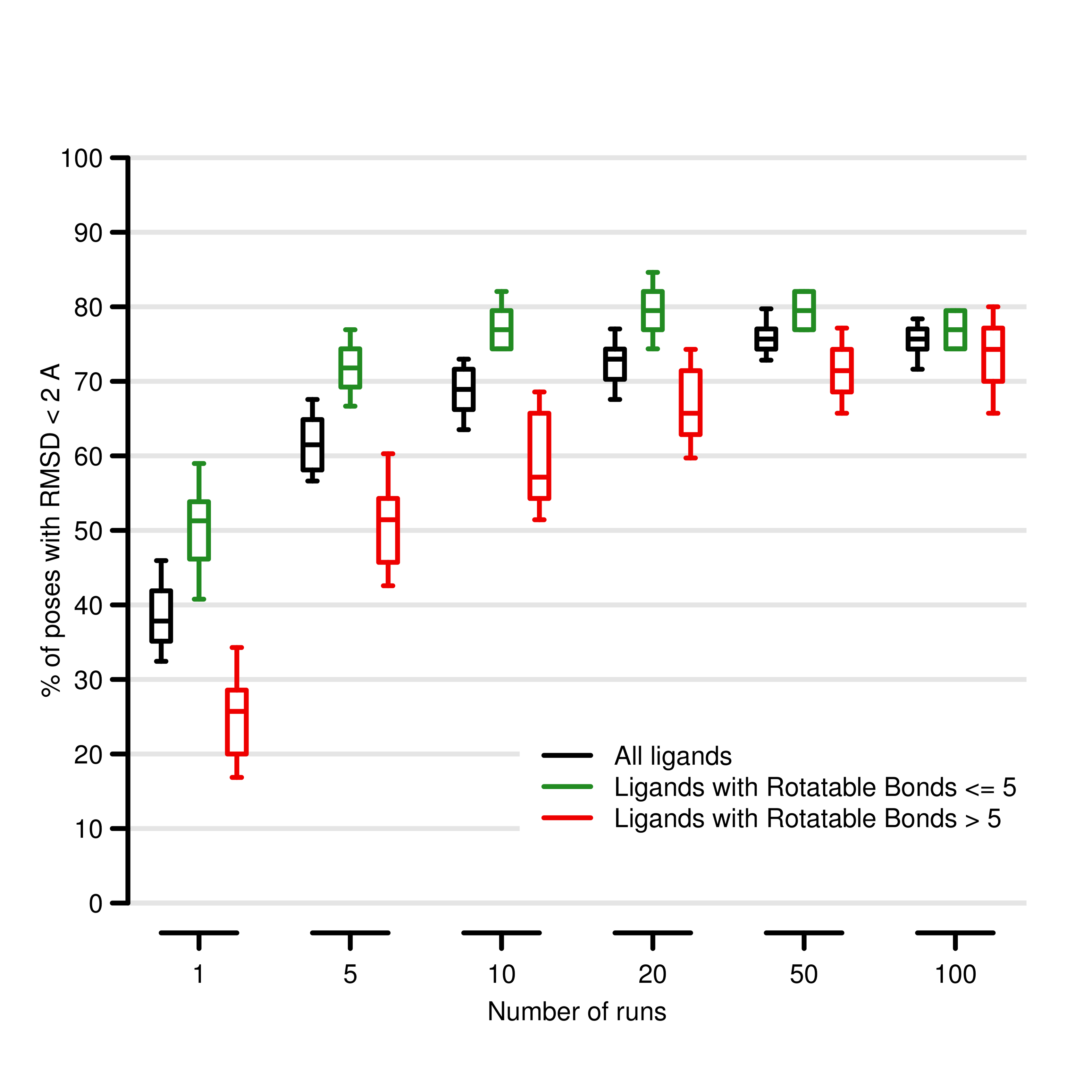

Supplement: Figure S2 — Binding mode prediction in the protein-ligand set (CCDC-Astex): Percentage of top-ranked poses with RMSD below 2.0 Å as a function of the number of docking runs. The boxplot indicates the median value (out of 100 possible solutions) and the first and last quartile, while the whiskers span the 10% to 90% range. The whole set (black) has been sub-divided into ligands with 5 or fewer rotatable bonds (green) and the rest (red). (TIF) [file pcbi.1003571.s002.tif]

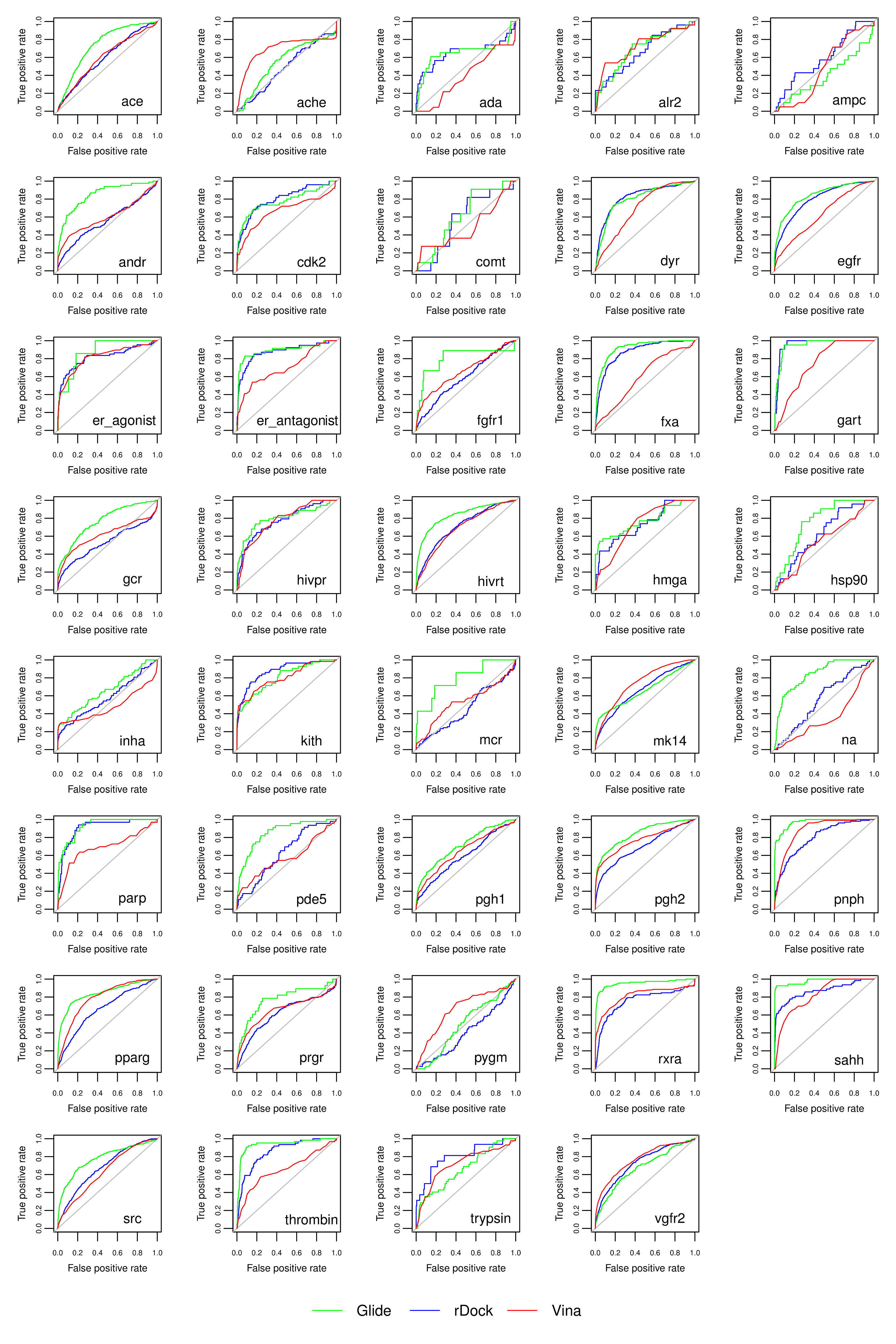

Supplement: Figure S5 — Receiver Operating Characteristic (ROC) Curves of all DUD systems. In the Y-axis, the true positive rate is the fraction of true positives out of the total actual positives and, in the X-axis, the false positive rate is the fraction of false positives out of the total actual negatives. In gray, ROC curve in case of random results. (TIF) [file pcbi.1003571.s005.tif]

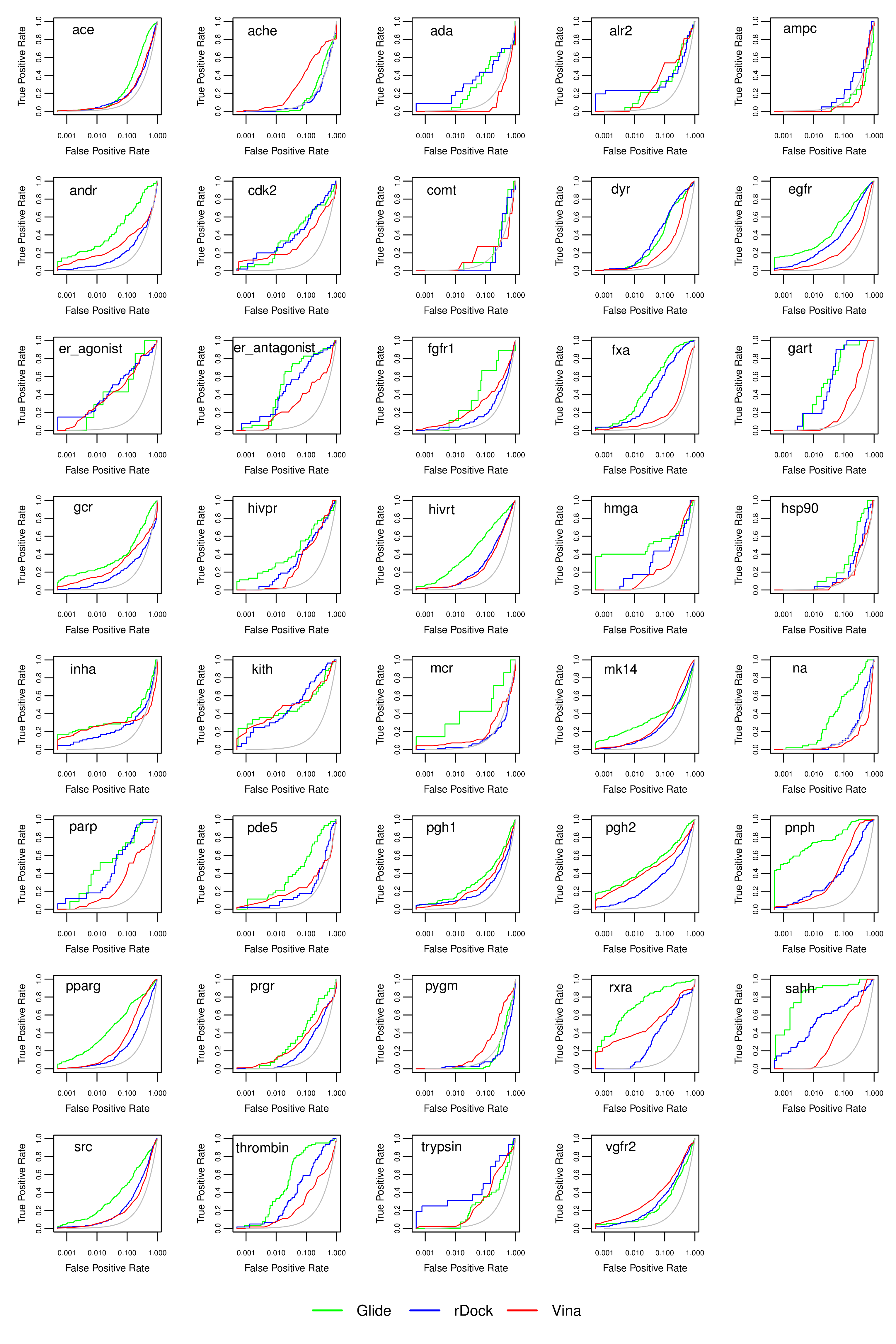

Supplement: Figure S6 — Semilogarithmic Receiver Operating Characteristic (ROC) Curves of all DUD systems. In the Y-axis, the true positive rate is the fraction of true positives out of the total actual positives and, in the X-axis in logarithmic scale, the false positive rate is the fraction of false positives out of the total actual negatives. In gray, semilogarithmic ROC curve in case of random results. (TIF) [file pcbi.1003571.s006.tif]

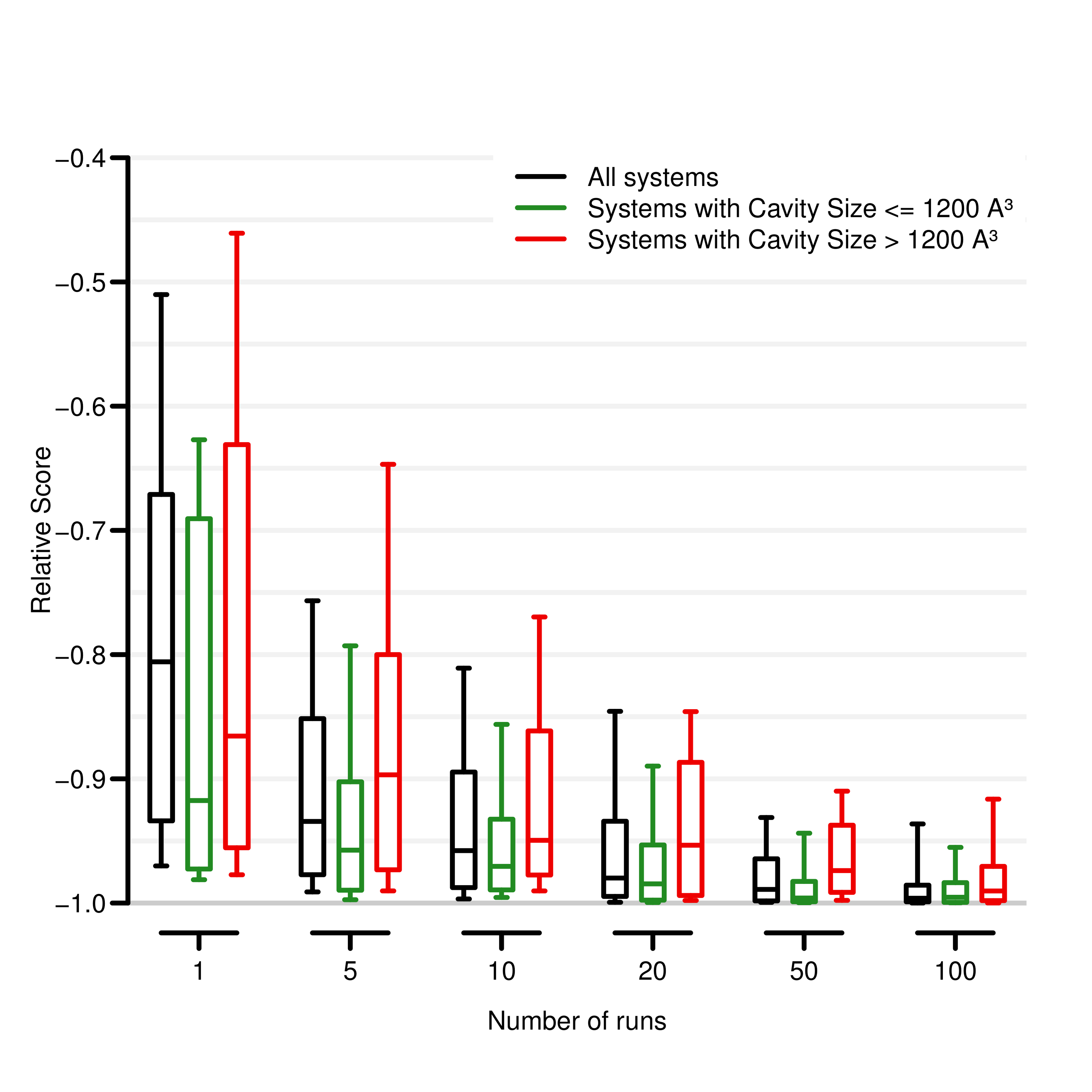

Supplement: Figure S7 — Relative score vs. the number of docking runs for all the protein-ligand complexes in the CCDC-Astex set. The boxplot indicates the median value (out of 100 possible solutions) and the first and last quartile, while the whiskers span the 10% to 90% range. The whole set (black) has been sub-divided into systems with relatively small cavities (green) and the rest (red). (TIF) [file pcbi.1003571.s007.tif]

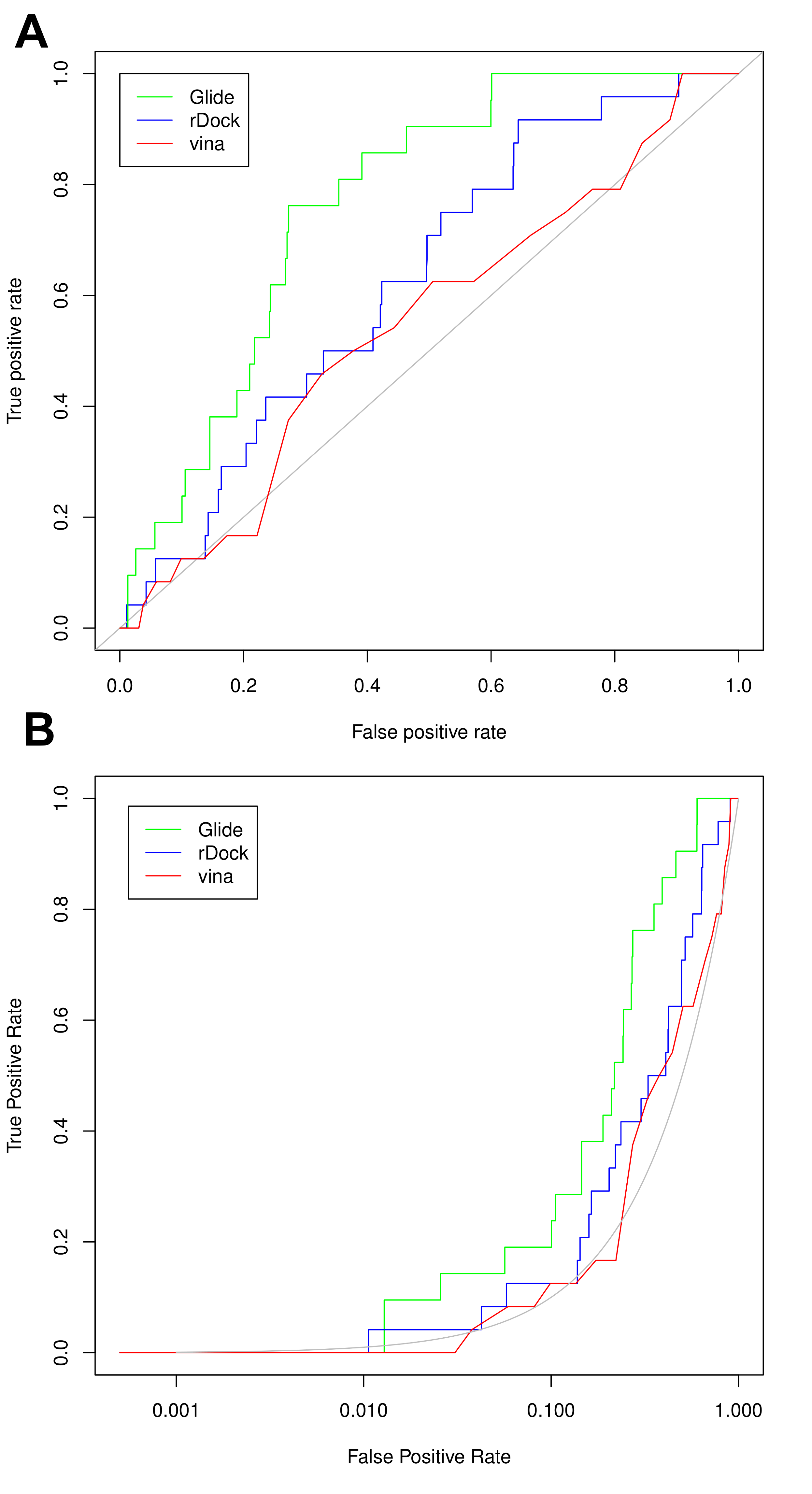

Supplement: Figure S8 — ROC curve of HSP90 without pharmacophoric restraints in normal (A) or semilogarithmic scale (B). (TIF) [file pcbi.1003571.s008.tif]

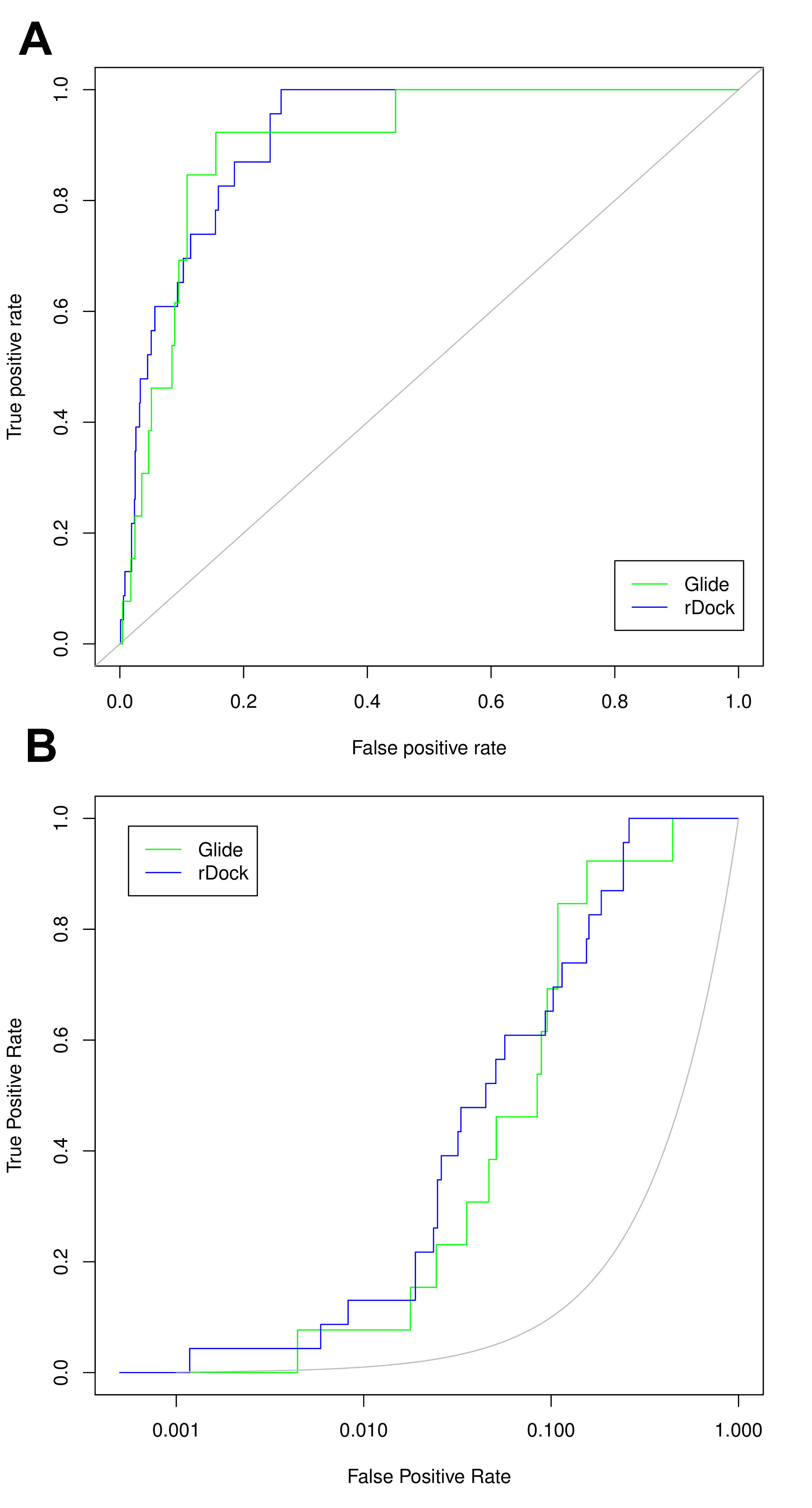

Supplement: Figure S9 — ROC curve of HSP90 with pharmacophoric restraints in normal (A) or semilogarithmic scale (B). It should be noted that using these settings, Glide only produces an output for 13 actives (out of 24) and 451 decoys (out of 864). (TIF) [file pcbi.1003571.s009.tif]
