# Supplementary material for: rDock: A Fast, Versatile and Open Source Program for Docking Ligands to Proteins and Nucleic Acids
Source: PLoS Comput Biol. 2014 Apr 10;10(4):e1003571. doi: 10.1371/journal.pcbi.1003571 (PMC3983074; doi:10.1371/journal.pcbi.1003571)
Supplement: Table S1 — Summary of statistics for all DUD systems and averages for each and all programs. (DOCX) [file pcbi.1003571.s011.docx]

**Table S1. Summary of statistics for all DUD systems and averages for each and all programs**.

| **DUD system** | **Program** | **AUC** | **logAUC** | **EFmax** | **EF1** | **EF20** |
| --- | --- | --- | --- | --- | --- | --- |
| ace | rDock | 0.6 | 0.14 | 11.95 | 4.24 | 1.47 |
|  | Glide | 0.74 | 0.19 | 60.09 | 3.22 | 2.42 |
|  | Vina | 0.6 | 0.15 | 8.59 | 3.08 | 1.62 |
| ache | rDock | 0.49 | 0.12 | 1.84 | 0.97 | 0.88 |
|  | Glide | 0.55 | 0.14 | 1.48 | 0 | 1.33 |
|  | Vina | 0.69 | 0.28 | 11.63 | 5.23 | 2.78 |
| ada | rDock | 0.66 | 0.35 | 70.35 | 19.54 | 2.82 |
|  | Glide | 0.65 | 0.3 | 8.3 | 4.32 | 3.02 |
|  | Vina | 0.39 | 0.09 | 1 | 0 | 0.19 |
| alr2 | rDock | 0.67 | 0.35 | 207.92 | 20.79 | 2.11 |
|  | Glide | 0.69 | 0.29 | 13.45 | 7.77 | 2.29 |
|  | Vina | 0.72 | 0.31 | 6.71 | 3.15 | 2.44 |
| ampc | rDock | 0.59 | 0.2 | 2.66 | 0 | 1.9 |
|  | Glide | 0.4 | 0.12 | 1.22 | 0 | 0.95 |
|  | Vina | 0.48 | 0.12 | 1.22 | 0 | 0.39 |
| andr | rDock | 0.56 | 0.16 | 53.28 | 5.55 | 1.71 |
|  | Glide | 0.85 | 0.4 | 797.68 | 27.18 | 3.69 |
|  | Vina | 0.6 | 0.23 | 586.11 | 12.63 | 2.08 |
| cdk2 | rDock | 0.8 | 0.4 | 87.4 | 21.36 | 3.5 |
|  | Glide | 0.78 | 0.39 | 69.29 | 21.65 | 3.44 |
|  | Vina | 0.67 | 0.32 | 174.8 | 17.48 | 2.38 |
| comt | rDock | 0.57 | 0.16 | 1.78 | 0 | 0.45 |
|  | Glide | 0.62 | 0.21 | 4.76 | 0 | 1.34 |
|  | Vina | 0.49 | 0.24 | 5.44 | 0 | 1.24 |
| dyr | rDock | 0.83 | 0.27 | 14.83 | 7.33 | 3.66 |
|  | Glide | 0.8 | 0.25 | 14.55 | 5.82 | 3.69 |
|  | Vina | 0.7 | 0.17 | 18.57 | 3.12 | 1.92 |
| egfr | rDock | 0.8 | 0.25 | 322.88 | 15.45 | 3.1 |
|  | Glide | 0.84 | 0.35 | 2514.17 | 24.99 | 3.67 |
|  | Vina | 0.63 | 0.16 | 129.17 | 6.51 | 1.74 |
| er agonist | rDock | 0.82 | 0.44 | 326.57 | 28.2 | 3.73 |
|  | Glide | 0.88 | 0.44 | 36.04 | 28.03 | 4.28 |
|  | Vina | 0.82 | 0.4 | 45.72 | 24.19 | 3.51 |
| er antagonist | rDock | 0.88 | 0.49 | 107.15 | 22.96 | 4.22 |
|  | Glide | 0.89 | 0.52 | 36.23 | 33.44 | 4.28 |
|  | Vina | 0.7 | 0.3 | 17.86 | 16.67 | 2.43 |

| **DUD system** | **Program** | **AUC** | **logAUC** | **EFmax** | **EF1** | **EF20** |
| --- | --- | --- | --- | --- | --- | --- |
| fgfr1 | rDock | 0.59 | 0.16 | 12.41 | 3.57 | 1.55 |
|  | Glide | 0.81 | 0.35 | 18.09 | 11.06 | 3.33 |
|  | Vina | 0.66 | 0.24 | 28.21 | 11.76 | 2.16 |
| fxa | rDock | 0.88 | 0.35 | 89.57 | 12.9 | 3.88 |
|  | Glide | 0.92 | 0.42 | 53.09 | 24.99 | 4.49 |
|  | Vina | 0.6 | 0.17 | 35.83 | 3.98 | 1.28 |
| gart | rDock | 0.96 | 0.56 | 44.13 | 18.91 | 4.96 |
|  | Glide | 0.95 | 0.57 | 42.03 | 27.02 | 4.74 |
|  | Vina | 0.74 | 0.26 | 2.6 | 0 | 1.99 |
| gcr | rDock | 0.53 | 0.16 | 57.91 | 7.33 | 1.74 |
|  | Glide | 0.77 | 0.31 | 327.16 | 22.93 | 2.92 |
|  | Vina | 0.62 | 0.24 | 144.77 | 13.36 | 2.32 |
| hivpr | rDock | 0.77 | 0.31 | 14.4 | 11.12 | 3.2 |
|  | Glide | 0.8 | 0.43 | 186.91 | 29.32 | 3.67 |
|  | Vina | 0.77 | 0.28 | 5.7 | 1.76 | 2.69 |
| hivrt | rDock | 0.71 | 0.2 | 111.18 | 5.03 | 2.48 |
|  | Glide | 0.84 | 0.33 | 312 | 25.84 | 3.74 |
|  | Vina | 0.69 | 0.19 | 37.29 | 5.91 | 2.23 |
| hmga | rDock | 0.73 | 0.34 | 29.87 | 15.93 | 2.81 |
|  | Glide | 0.77 | 0.53 | 492 | 37.85 | 2.99 |
|  | Vina | 0.73 | 0.28 | 6.09 | 2.72 | 2 |
| hsp90 | rDock | 0.63 | 0.2 | 3.93 | 0 | 1.45 |
|  | Glide | 0.77 | 0.28 | 7.4 | 0 | 2.13 |
|  | Vina | 0.55 | 0.16 | 1.41 | 0 | 0.75 |
| inha | rDock | 0.58 | 0.23 | 144.82 | 12.85 | 1.86 |
|  | Glide | 0.66 | 0.34 | 438.17 | 26.68 | 2.21 |
|  | Vina | 0.48 | 0.28 | 325.84 | 19.91 | 1.62 |
| kith | rDock | 0.88 | 0.46 | 140 | 31.03 | 3.94 |
|  | Glide | 0.8 | 0.48 | 437.86 | 36.87 | 3.09 |
|  | Vina | 0.8 | 0.47 | 350 | 32.5 | 3.25 |
| mcr | rDock | 0.46 | 0.11 | 4.55 | 2.1 | 0.9 |
|  | Glide | 0.8 | 0.45 | 126.29 | 28.06 | 3.57 |
|  | Vina | 0.54 | 0.17 | 218.64 | 6.49 | 1.63 |
| mk14 | rDock | 0.66 | 0.17 | 16.04 | 7.09 | 2.09 |
|  | Glide | 0.66 | 0.26 | 807.95 | 24.16 | 2.34 |
|  | Vina | 0.74 | 0.2 | 124.11 | 8.84 | 2.54 |

| **DUD system** | **Program** | **AUC** | **logAUC** | **EFmax** | **EF1** | **EF20** |
| --- | --- | --- | --- | --- | --- | --- |
| na | rDock | 0.57 | 0.15 | 1.63 | 0 | 1.22 |
|  | Glide | 0.84 | 0.36 | 17.07 | 6.03 | 3.36 |
|  | Vina | 0.37 | 0.09 | 1 | 0 | 0.64 |
| parp | rDock | 0.91 | 0.49 | 134.42 | 16.8 | 4.54 |
|  | Glide | 0.93 | 0.58 | 68.43 | 42.77 | 4.33 |
|  | Vina | 0.69 | 0.29 | 11.2 | 7.91 | 2.88 |
| pde5 | rDock | 0.63 | 0.2 | 38.98 | 4.33 | 1.41 |
|  | Glide | 0.85 | 0.4 | 100.97 | 20.19 | 3.74 |
|  | Vina | 0.57 | 0.24 | 38.98 | 12.99 | 1.69 |
| pgh1 | rDock | 0.6 | 0.19 | 275.23 | 9.17 | 1.69 |
|  | Glide | 0.71 | 0.26 | 160.74 | 13.23 | 2.53 |
|  | Vina | 0.65 | 0.2 | 41.48 | 7.67 | 1.97 |
| pgh2 | rDock | 0.7 | 0.23 | 52.98 | 13.53 | 2.52 |
|  | Glide | 0.84 | 0.42 | 1955.11 | 35.47 | 3.6 |
|  | Vina | 0.77 | 0.34 | 637.02 | 31.1 | 2.92 |
| pnph | rDock | 0.8 | 0.3 | 134.95 | 20.24 | 3.05 |
|  | Glide | 0.97 | 0.72 | 1720.44 | 73.02 | 4.88 |
|  | Vina | 0.88 | 0.35 | 50.61 | 12.19 | 3.68 |
| pparg | rDock | 0.67 | 0.17 | 9.51 | 4.57 | 2.11 |
|  | Glide | 0.84 | 0.34 | 202.49 | 28.86 | 3.84 |
|  | Vina | 0.79 | 0.23 | 8.24 | 5.67 | 3.08 |
| prgr | rDock | 0.62 | 0.16 | 5.92 | 1.7 | 2.2 |
|  | Glide | 0.77 | 0.28 | 12.83 | 10.57 | 3.21 |
|  | Vina | 0.66 | 0.24 | 35.5 | 12.01 | 2.33 |
| pygm | rDock | 0.41 | 0.09 | 5.68 | 2.56 | 0.58 |
|  | Glide | 0.5 | 0.1 | 1.18 | 0 | 0.53 |
|  | Vina | 0.67 | 0.2 | 2.95 | 2.38 | 2 |
| rxra | rDock | 0.76 | 0.26 | 8.51 | 6.81 | 3.32 |
|  | Glide | 0.95 | 0.65 | 654.83 | 67.62 | 4.7 |
|  | Vina | 0.81 | 0.45 | 1111.72 | 29.6 | 3.41 |
| sahh | rDock | 0.87 | 0.51 | 328.38 | 48.48 | 3.97 |
|  | Glide | 0.98 | 0.83 | 698.49 | 86.39 | 4.71 |
|  | Vina | 0.82 | 0.29 | 7.63 | 2.67 | 3.26 |
| src | rDock | 0.68 | 0.17 | 262.56 | 4.77 | 2.16 |
|  | Glide | 0.78 | 0.27 | 133.95 | 18.12 | 3.28 |
|  | Vina | 0.64 | 0.15 | 32.84 | 5.16 | 1.63 |

| **DUD system** | **Program** | **AUC** | **logAUC** | **EFmax** | **EF1** | **EF20** |
| --- | --- | --- | --- | --- | --- | --- |
| thrombin | rDock | 0.86 | 0.36 | 36.89 | 8.02 | 3.76 |
|  | Glide | 0.94 | 0.5 | 40.36 | 32.25 | 4.75 |
|  | Vina | 0.66 | 0.28 | 11.9 | 7.44 | 2.42 |
| trypsin | rDock | 0.8 | 0.45 | 320.75 | 30.84 | 3.43 |
|  | Glide | 0.66 | 0.24 | 7.42 | 0 | 2.02 |
|  | Vina | 0.72 | 0.27 | 35.35 | 2.21 | 2.69 |
| vgfr2 | rDock | 0.73 | 0.22 | 365.4 | 8.73 | 2.55 |
|  | Glide | 0.68 | 0.2 | 170.25 | 7.83 | 2.3 |
|  | Vina | 0.77 | 0.27 | 426.3 | 17.46 | 2.84 |
| **Summary** | **Program** | **AUC** | **logAUC** | **EFmax** | **EF1** | **EF20** |
| Averages | rDock | 0.7 | 0.27 | 98.95 | 11.66 | 2.54 |
|  | Glide | 0.78 | 0.37 | 326.94 | 22.91 | 3.22 |
|  | Vina | 0.66 | 0.25 | 121.54 | 9.12 | 2.17 |
|  | All | 0.71 | 0.3 | 182.48 | 14.56 | 2.64 |

**AUC**: Area Under the ROC Curve. **LogAUC**: Area Under the semilog ROC Curve. **EFmax**: Maximal Enrichment Factor. **EFX**: Enrichment Factor when the top x% of the virtual collection is selected.
