# Supplementary material for: rDock: A Fast, Versatile and Open Source Program for Docking Ligands to Proteins and Nucleic Acids
Source: PLoS Comput Biol. 2014 Apr 10;10(4):e1003571. doi: 10.1371/journal.pcbi.1003571 (PMC3983074; doi:10.1371/journal.pcbi.1003571)
Supplement: Table S2 — Spearman's rank correlation coefficient (ρ) between programs on the Hsp90 DUD set. (DOCX) [file pcbi.1003571.s012.docx]

**Table S2.** **Spearman's rank correlation coefficient (ρ)* between programs on the Hsp90 DUD set.**

| **Program** | **rDock** | **rDock-guided** | **Glide** | **Glide-guided** | **Vina** |
| --- | --- | --- | --- | --- | --- |
| rDock | 1 | 0,37 | 0,46 | 0,22 | 0,33 |
| rDock-guided |  | 1 | 0,37 | 0,52 | 0,13 |
| Glide |  |  | 1 | 0,41 | 0,31 |
| Glide-guided |  |  |  | 1 | 0,13 |
| Vina |  |  |  |  | 1 |

* The introduction of empirical information produces results very different to the default parameters restraints (ρ = 0.37 for rDock; ρ = 0.41 for Glide) while the output of different programs becomes more similar (ρ = 0.52, comparing rDock-guided with Glide-guided). ρ is calculated from the rank of 464 molecules for which Glide-guided produces an output.
