# Supplementary material for: rDock: A Fast, Versatile and Open Source Program for Docking Ligands to Proteins and Nucleic Acids
Source: PLoS Comput Biol. 2014 Apr 10;10(4):e1003571. doi: 10.1371/journal.pcbi.1003571 (PMC3983074; doi:10.1371/journal.pcbi.1003571)
Supplement: Table S3 — AUC for the 4 DUD systems used for calculating the time performance. (DOCX) [file pcbi.1003571.s013.docx]

**Table S3.** **AUC for the 4 DUD systems used for calculating the time performance.**

|  | **Vina^1^** | **Glide SP^1^** | **rDock** | | | |
| --- | --- | --- | --- | --- | --- | --- |
|  |  |  | **Grid-based SF** | | **Indexed SF** | |
|  |  |  | **VS^2^** | **Full^1,3^** | **VS^2^** | **Full^1,3^** |
| ADA | 0.39 | 0.67 | 0.68 | 0.64 | 0.57 | 0.62 |
| COMT | 0.51 | 0.69 | 0.67 | 0.62 | 0.65 | 0.64 |
| PARP | 0.68 | 0.9 | 0.88 | 0.86 | 0.87 | 0.86 |
| Trypsin | 0.74 | 0.49 | 0.63 | 0.62 | 0.66 | 0.76 |
| **Average** | **0.58** | **0.69** | **0.72** | **0.69** | **0.69** | **0.72** |

^1^ Default program parameters were used. ^2^ On HTVS mode, the average number of docking runs needed for these 4 systems is 10. ^3^ 50 docking runs are used for default docking.
