# Supplementary material for: rDock: A Fast, Versatile and Open Source Program for Docking Ligands to Proteins and Nucleic Acids
Source: PLoS Comput Biol. 2014 Apr 10;10(4):e1003571. doi: 10.1371/journal.pcbi.1003571 (PMC3983074; doi:10.1371/journal.pcbi.1003571)
Supplement: Text S1 — Supporting Methods: Test set preparation, execution and analysis. (DOCX) [file pcbi.1003571.s014.docx]

**Text S1. Supporting Methods: Test set preparation, execution and analysis.**

**DUD and ASTEX sets:**

- Protein Preparation

The receptor structure files in DUD [1] and Astex [2] sets were processed using the Preparation Wizard tool from Maestro [3], and were then used as input for the three programs. To define the cavity, rDock was run using the crystallographic ligand provided as reference with the “reference ligand method” and the following parameter values (if not in the list, the default value was considered): radius=6.0, small_sphere=1.0 and max_cavities=1. The coordinates obtained for the center and the size of the binding site were applied for Glide[4] and Vina[5] to ensure the least dissimilar cavities between each program.

- Ligand Preparation

The structure of the ligands in DUD set was converted to smiles format and processed with the LigPrep software [6] applying the following filters: maximum atoms=100, maximum stereoisomers=8, maximum tautomers=6 and ionizing at pH=7 with a tolerance of +- 1.

The results insdf format, compatible for running rDock, were converted to mae and pdbqt formats for running Glide and Vina, respectively.

For the Astex set of complexes, the ligands had already been manually prepared, thus LigPrep processing was not needed. Hence, the process was the same as for DUD set after the ligands had been processed with LigPrep.

- Docking

The Molecular Docking process was defined to be the most similar as possible. The exhaustiveness of all programs was set higher than default to remove uncertainty due to low sampling.

For the DUD set, rDock was run with a receptor flexibility=3, scoring function “dock.prm” and 100 docking runs. Glide was run with “expanded sampling” option turned on and with the following options increased with respect to the default values to avoid filtering of intermediate poses and bad scored ligands which facilitated analysis of the results: postdock_npose=5000, poses_per_lig=5000 and nreport=(5000*number of ligands). Vina had all parameters as default but the following ones, for the same reason as Glide: exhaustiveness=16, num_modes=100 and energy_range=30.

For the Astex set, all the parameters were the same as in DUD but the number of runs in rDock and Vina were adjusted to give the same number of poses. A pool of 1000 docking poses was obtained from rDock and Vina (the 50 jobs from Vina each generated 20 docking poses) which allowed us to generate statistical parameters. For Glide, as there is no control for defining an exact number of output docking poses, no repeated sampling was generated for this purpose.

- Results analysis

For the DUD set, ROC curves were generated using ROCR package for R [7] and several statistical values, such as AUC and Enrichment Factors, were calculated.

For the Astex set, the RMSD of each predicted binding mode with respect to the crystallized ligand was calculated using Open Babel toolkit [8]. Random sets of 100 ligands were selected from all the resulting binding modes (if more than 100 ligands were available) and the percentage of the top-scored binding mode with an RMSD below 2Å was calculated.

**RNA:**

The structure of the RNA-ligand complex was downloaded from the PDB and prepared using MOE [9]. The cavity was defined using the crystallographic ligand in the PDB as reference with the “reference ligand method” from rDock and the following parameters different from default: radius=4.0, small_sphere=1.0 and max_cavities=1.

Like in the Astex and DUD sets, the coordinates obtained for the center and the size of the binding site were applied for Glide and Vina to ensure the least dissimilar cavities between each program. In Glide, the cavity was defined a little bigger than the rest to ensure that the program did not fail in any system due to a small cavity size.

The docking jobs for rDock were run with receptor flexibility=3, scoring function “dock_solv.prm” and 1000 docking runs, for statistical purposes in analysis of results. Glide and Vina were set to run with the same parameters than in DUD and ASTEX sets.

The RMSD of each predicted binding mode with respect to the crystallized ligand was calculated and random sets of 100 ligands were selected for calculating the percentage of top-scored binding modes with an RMSD below 2.5 Å.

**Pharmacophoric restraints:**

Based on the knowledge available on the DUD systems and on their pharmacophoric properties, we selected HSP90. Three structural waters were added near to ASP78, the volume around residues TRP147 and GLY93 was excluded and hydrogen-bonds between ASP78 and the ligand and between one of the structural waters added and the ligand were added as pharmacophoric restraints.

rDock and Glide were run with the same parameters as in the same DUD system without any pharmacophoric restraint (Vina cannot use pharmacophoric restraints).

The results were processed the same way as in DUD set.

**References**

1. Huang N, Shoichet BK, Irwin JJ. (2006) Benchmarking sets for molecular docking. J Med Chem 49: 6789-6801.

2. Hartshorn MJ, Verdonk ML, Chessari G, Brewerton SC, Mooij WT, et al. (2007) Diverse, high-quality test set for the validation of protein-ligand docking performance. J Med Chem 50: 726-741.

3. Schrödinger L. (2011) Suite 2011: Maestro, version 9.4.

4. Friesner RA, Banks JL, Murphy RB, Halgren TA, Klicic JJ, et al. (2004) Glide: A new approach for rapid, accurate docking and scoring. 1. method and assessment of docking accuracy. J Med Chem 47: 1739-1749.

5. Trott O, Olson AJ. (2010) AutoDockvina: Improving the speed and accuracy of docking with a new scoring function, efficient optimization, and multithreading. J ComputChem 31: 455-461.

6. Schrödinger L. (2011) Suite 2011: LigPrep, version 2.5.

7. Tobias Sing, Oliver Sander, Niko Beerenwinkel, Thomas Lengauer. (2005). ROCR: visualizing classifier performance in R. Bioinformatics*.* 21(20):3940-3941.

8. O'Boyle NM, Banck M, James CA, Morley C, Vandermeersch T, et al. (2011) Open babel: An open chemical toolbox. J Cheminform 3: 33-2946-3-33.

9. Chemical Computing Group Inc., 1010 Sherbooke St. West, Suite #910, Montreal, QC, Canada, H3A 2R7,(2013) *Molecular Operating Environment (MOE)*, 2013.08;
