# Supplementary material for: rDock: A Fast, Versatile and Open Source Program for Docking Ligands to Proteins and Nucleic Acids
Source: PLoS Comput Biol. 2014 Apr 10;10(4):e1003571. doi: 10.1371/journal.pcbi.1003571 (PMC3983074; doi:10.1371/journal.pcbi.1003571)
Supplement: Text S2 — Full Acknowledgements. (DOCX) [file pcbi.1003571.s015.docx]

**Text S2: Full Acknowledgements**

The development of the initial RiboDock program at RiboTargets was directed by Mohammad Afshar and he (with managerial support from Rod Hubbard, David Knowles and Simon Sturge) oversaw the further development into the program rDock. Many expert users at RiboTargets (subsequently Vernalis) provided ideas and testing of many aspects of the program. In particular:

- I-Jen Chen performed the initial validation experiments

- Ben Davis and Fareed Aboul-ela helped with development of NMR restrained docking protocols

- Michael Brunsteiner helped with improvements to the Simplex minimization algorithm

- Alba Macias tested the docking protocols with explicit water molecules

- Christine Richardson provided user feedback on feature developments

Maintenance and distribution of rDock was transferred to the University of York (Rod Hubbard) in 2006. There, a number of students helped maintain and validate the software and generate an initial website with user interfaces. Most recently, Sanjana Sood and Paul Bond made substantial contributions to this work.
