# Supplementary material for: rDock: A Fast, Versatile and Open Source Program for Docking Ligands to Proteins and Nucleic Acids
Source: PLoS Comput Biol. 2014 Apr 10;10(4):e1003571. doi: 10.1371/journal.pcbi.1003571 (PMC3983074; doi:10.1371/journal.pcbi.1003571)
Supplement: Software S1 — Compressed file with the source code of the rDock software for ligand docking to Proteins and Nucleic Acids. (GZ) [file pcbi.1003571.s010.gz › rDock_2013.1_src/docs/rDock_reference_guide.html]

rDock Reference Guide

# rDock Reference Guide

### Dr. David Morley

Enspiral Discovery Ltd.

`<d.morley@enspiral-discovery.com>`

rDock/2006.1/901

Copyright © 2006 Vernalis

| **Revision History** | |
| --- | --- |
| Revision Added Acknowledgements section | 27/09/2006 |
| Revision Completed concise reference guide (at least some content in all sections) | 26/06/2006 |
| Revision Concise reference guide in progress | 20/06/2006 |
| Revision Initial draft describing rDock/enspiral\_dev/449 receptor .prm file | 26/01/2006 |

---

**Table of Contents**

Preface

Acknowledgements

1. Introduction

2. Configuration

3. Molecular files and atom typing

4. Cavity mapping

5. Common use cases
:   Standard docking

    Tethered scaffold docking

    Docking with pharmacophore restraints

    Docking with explicit waters

6. Scoring function reference

7. Docking protocol reference

8. System definition file reference
:   Receptor definition

    Ligand definition

    Solvent definition

    Cavity mapping

    Cavity restraint

    Pharmacophore restraints

    NMR restraints

    Example system definition files

9. rDock file formats
:   .prm file format

    Water PDB file format

    Pharmacophore restraints file format

10. rDock programs
:   Executable (binary) programs

    Scripts

    Command-line reference

**List of Tables**

6.1. Scoring function terms and C++ implementation classes

6.2. Scoring function data files

7.1. Chromsome elements

7.2. Search algorithm components and C++ implementation classes

7.3. Docking protocol data files

8.1. Receptor definition parameters

8.2. Ligand definition parameters

8.3. Solvent definition parameters

8.4. Two sphere site mapping parameters

8.5. Reference ligand site mapping parameters

8.6. Pharmacophore restraint parameters

9.1. Conversion of temperature factor values to solvent flexibility modes

9.2. Pharmacophore restraint types

10.1. Core rDock C++ executables

10.2. Auxiliary rDock C++ executables

10.3. Core scripts

10.4. Auxiliary scripts

**List of Examples**

5.1. Example ligand definition for tethered scaffold

7.1. Standard rDock docking protocol (`dock.prm`)

8.1. Full system definition file with all sections and common parameters enumerated explicitly

9.1. Example .prm file

9.2. Valid rDock PDB file for explicit, flexible waters

## Preface

It is intended to develop this document into a full reference guide for the
rDock platform, describing the software tools, parameter
files, scoring functions, and search engines. The reader is encouraged to
cross-reference the descriptions with the corresponding source code files to discover
the finer implementation details.

## Acknowledgements

**Third-party source code.** Two third-party C++ libraries are included within the rDock source code, to
provide support for specific numerical calculations. The source code for each
library can be distributed freely without licensing restrictions and we are grateful
to the respective authors for their contributions.

- Nelder-Meads Simplex search, from Prof. Virginia Torczon's group,
  College of William and Mary, Department of Computer Science, VA. (http://www.cs.wm.edu/~va/software/)
- Template Numerical Toolkit, from Roldan Pozo, Mathematical and
  Computational Sciences Division, National Institute of Standards and
  Technology (http://math.nist.gov/tnt/)

## Chapter 1. Introduction

The rDock platform is a suite of command-line tools for high-throughput docking and
virtual screening. The programs and methods were developed and validated over a number
of years at RiboTargets (more recently, Vernalis) for proprietary use. The original
program (RiboDock) was designed for high-throughput virtual screening of large ligand
libraries against RNA targets, in particular the bacterial ribosome. Since 2002 the
programs have been substantially rewritten and validated for docking of drug-like
ligands to protein and RNA targets. A variety of experimental restraints can be
incorporated into the docking calculation, in support of an integrated Structure-Based
Drug Design process.

## Chapter 2. Configuration

Before launching rDock, make sure the following environment variable are defined.
Precise details are likely to be site-specific.

**RBT\_ROOT environment variable.** `$RBT_ROOT` environment variable should be defined to point to
the rDock installation directory.

**RBT\_HOME environment variable.** `$RBT_HOME` environment variable is optional, but can defined to
to point to a user project directory containing rDock input files and customised
data files.

**PATH environment variable.** `$RBT_ROOT/bin` should be added to the `$PATH`
environment variable.

**LD\_LIBRARY\_PATH.** `$RBT_ROOT/lib` should be added to the
`$LD_LIBRARY_PATH` environment variable.

**Input file locations.** The search path for the majority of input files for rDock is:

- Current working directory
- `$RBT_HOME`, if defined
- The appropriate subdirectory of `$RBT_ROOT/data/`.
  For example, the default location for scoring function files is
  `$RBT_ROOT/data/sf/`.

The exception is that input ligand SD files are always specified as
an absolute path. If you wish to customise a scoring function or docking protocol,
it is sufficient to copy the relevant file to the current working directory or to
`$RBT_HOME`, and to modify the copied file.

**Launching rDock.** For small scale experimentation, the rDock executables can be launched directly
from the command line. However, serious virtual screening campaigns will likely need
access to a compute farm. In common with other docking tools, rDock uses the
embarrassingly parallel approach to distributed computing. Large ligand libraries
are split into smaller chunks, each of which is docked independently on a single
machine. Docking jobs are controlled by a distributed resource manager (DRM) such as
Condor. The RBT\_TEST suite provides some examples of the generation of Condor
command files for rDock.

## Chapter 3. Molecular files and atom typing

Macromolecular targets (protein or RNA) are input from Tripos MOL2 files
(`RbtMOL2FileSource`) or from pairs of Charmm PSF
(`RbtPsfFileSource`) and CRD
(`RbtCrdFileSource`) files. Ligands are input from MDL SD files
(`RbtMdlFileSource`). Explicit structural waters are input
optionally from PDB files (`RbtPdbFileSource`). Ligand docking
poses are output to MDL SD files.

The rDock scoring functions have been defined and validated for implicit non-polar
hydrogen (extended carbon) models only. If you provide all-atom models, be aware that
the non-polar hydrogens will be removed automatically. Polar hydrogens must be defined
explicitly in the molecular files, and are not added by rDock. Positive ionisable and
negative ionisable groups can be automatically protonated and deprotonated respectively
to create common charged groups such as guanidinium and carboxylic acid groups.

### Tip

MOL2 is now the preferred file format for rDock as it eliminates many of the
atom typing issues inherent in preparing and loading PSF files. The use of PSF/CRD
files is strongly discouraged. The recommendation is to prepare an all-atom MOL2
file with correct Tripos atom types assigned, and allow rDock to remove non-polar
hydrogens on-the-fly.

**Atomic properties.** rDock requires the following properties to be defined per atom. Depending on the
file format, these properties may be loaded directly from the molecular input file,
or derived internally once the model is loaded.

- Cartesian (x,y,z) coordinates
- Element (atomic number)
- Formal hybridisation state (sp, sp2, sp3, aromatic, trigonal
  planar)
- Formal charge
- Distributed formal charge (known informally as
  *group* charge)
- Tripos force field type (rDock uses a modified version of the Sybyl
  5.2 types, extended to include carbon types with implicit non-polar
  hydrogens)
- Atom name
- Substructure (residue) name
- Atomic radius (assigned per element from
  `$RBT_ROOT/data/RbtElements.dat`)

### Note

The rDock scoring functions do not use partial charges and therefore
partial charges do not have to be defined. The atomic radii are simplified
radii defined per element, and are used for cavity mapping and in the polar
scoring function term, but are *not* used in the vdW
scoring function term. The latter has its own indepedent parameterisation
based on the Tripos force field types.

**Difference between formal charge and distributed formal charge.** The formal charge on an atom is always an integer. For example, a charged
carboxylic acid group ( `COO-`) can be defined formally as a formal
double bond to a neutral oxygen sp2, and a formal single bond to a formally charged
oxygen sp3. In reality of course, both oxygens are equivalent. rDock distributes the
integer formal charge across all equivalent atoms in the charged group that are
topologically equivalent. In negatively charged acid groups, the formal charge is
distributed equally between the acid oxygens. In positively charged amines, the
formal charge is distributed equally between the hydrogens. In charged guanidinium,
amidinium, and imidazole groups, the central carbon also receives an equal portion
of the formal charge (in addition to the hydrogens). The distributed formal charge
is also known as the group charge. The polar scoring functions in rDock use the
distributed formal charge to scale the polar interaction strength of the polar
interactions.

**Parsing a MOL2 file.** `MOLECULE`, `ATOM`, `BOND` and
`SUBSTRUCTURE` records are parsed. The atom name, substructure
name, Cartesian coordinates and Tripos atom type are read directly for each atom.
The element type (atomic number) and formal hybridisation state are derived from the
Tripos type using an internal lookup table. Formal charges are *not*  read from the MOL2 file and do not have to be assigned correctly in the
file. Distributed formal charges are assigned directly by rDock based on standard
substructure and atom names as described below.

**Parsing an SD file.** Cartesian coordinates, element and formal charge are read directly for each atom.
Formal bond orders are read for each bond. Atom names are derived from element name
and atom ID ( e.g. `C1`, `N2`,
`C3` etc). The substructure name is `MOL`. Formal
hybridisation states are derived internally for each atom based on connectivity
patterns and formal bond orders. The Tripos types are asssigned using internal rules
based on atomic number, formal hybridisation state and formal charges. The integer
formal charges are distributed automatically across all topologically equivalent
atoms in the charged group.

**Assigning distributed formal charges to the receptor.** rDock provides a file format independent method for assigning distributed formal
charges directly to the receptor atoms, which is used by the MOL2 and PSF/CRD file
readers. The method uses a lookup table based on standard substructure and atom
names, and does not require the integer formal charges to be assigned to operate
correctly. The lookup table file is
`$RBT_ROOT/data/sf/RbtIonicAtoms.prm`. Each section name
represents a substructure name that contains formally charged atoms. The entries
within the section represent the atom names and distributed formal charges for that
substructure name. The file provided with rDock contains entries for all standard
amino acids and nucleic acids, common metals, and specific entries required for
processing the GOLD CCDC/Astex validation sets.

### Important

You may have to extend `RbtIonicAtoms.prm` if you
are working with non-standard receptor substructure names and/or atom names,
in order for the distributed formal charges to be assigned
correctly.

## Chapter 4. Cavity mapping

Virtual screening is very rarely conducted against entire macromolecules. The usual
practice is to dock small molecules in a much more confined region of interest. rDock
makes a clear distinction between the region the ligand is allowed to explore (known
here as the docking site), and the receptor atoms that need to be included in order to
calculate the score correctly. The former is controlled by the cavity mapping algorithm,
whilst the latter is scoring function dependent as it depends on the distance range of
each component term (for example, vdW range >> polar range). For this reason, it is
usual practice with rDock to prepare intact receptor files (rather than truncated
spheres around the region of interest), and to allow each scoring function term to
isolate the relevant receptor atoms within range of the docking site.

Dock provides two methods for defining the docking site:

- Two sphere method
- Reference ligand method

**Two sphere method.** The two sphere method aims to find cavities that are accessible to a small sphere
(of typical atomic or solvent radius) but are inaccessible to a larger sphere. The
larger sphere probe will eliminate flat and convex regions of the receptor surface,
and also shallow cavities. The regions that remain and are accessible to the small
sphere are likely to be the “nice well defined” cavities of interest for drug design
purposes.

**Procedure 4.1. Two sphere mapping algorithm**

1. A grid is placed over the cavity mapping region, encompassing a sphere of
   radius=RADIUS, center=CENTER. Cavity mapping is restricted to this sphere. All
   cavities located will be wholly within this sphere. Any cavity that would
   otherwise protrude beyond the cavity mapping sphere will be truncated at the
   periphery of the sphere.
2. Grid points within the volume occupied by the receptor are excluded (coloured
   red). The radii of the receptor atoms are increased temporarily by VOL\_INCR in
   this step.
3. Probes of radii LARGE\_SPHERE are placed on each remaining unallocated grid
   point and checked for clashes with receptor excluded volume. To eliminate edge
   effects, the grid is extended beyond the cavity mapping region by the diameter
   of the large sphere (for this step only). This allows the large probe to be
   placed on grid points outside of the cavity mapping region, yet partially
   protrude into the cavity mapping region.
4. All grid points within the cavity mapping region that are accessible to the
   large probe are excluded (coloured green).
5. Probes of radii SMALL\_SPHERE are placed on each remaining grid point and
   checked for clashes with receptor excluded volume (red) or large probe excluded
   volume (green)
6. All grid points that are accessible to the small probe are selected (yellow).
7. The final selection of cavity grid points is divided into distinct cavities
   (contiguous regions). In this example only one distinct cavity is found.
   User-defined filters of MIN\_VOLUME and MAX\_CAVITIES are applied at this stage to
   select a subset of cavities if required. Note that the filters will accept or
   reject intact cavities only.

**Reference ligand method.** The reference ligand method provides a much easier option to define a docking
volume of a given size around the binding mode of a known ligand, and is
particularly appropriate for large scale automated validation experiments.

1. Reference coordinates are read from REF\_MOL. A grid is placed over the cavity
   mapping region, encompassing overlapping spheres of radius=RADIUS, centered on
   each atom in REF\_MOL. Grid points outside of the overlapping spheres are
   excluded (coloured green). Grid points within the volume occupied by the
   receptor are excluded (coloured red). The vdW radii of the receptor atoms are
   increased by VOL\_INCR in this step.
2. Probes of radii SMALL\_SPHERE are placed on each remaining grid point and
   checked for clashes with red or green regions.
3. All grid points that are accessible to the small probe are selected (yellow).
4. The final selection of cavity grid points is divided into distinct cavities
   (contiguous regions). In this example only one distinct cavity is found.
   User-defined filters of MIN\_VOLUME and MAX\_CAVITIES are applied at this stage to
   select a subset of cavities if required. Note that the filters will accept or
   reject intact cavities only.

## Chapter 5. Common use cases

**Table of Contents**

Standard docking

Tethered scaffold docking

Docking with pharmacophore restraints

Docking with explicit waters

This section does not pretend to be a comprehensive User Guide. It does, however,
highlight the key steps the user must take for different docking strategies, and may
serve as a useful checklist in writing such a guide in the future.

## Standard docking

By standard docking, we refer to docking of a flexible, untethered ligand to a
receptor in the absence of explicit structural waters or any experimental
restraints.

**Procedure 5.1. Standard docking workflow**

1. Prepare a MOL2 file for the protein or nucleic acid target, taking into
   account the atom typing issues described above for MOL2 file parsing. The
   recommendation is to prepare an all-atom MOL2 file and allow rDock to remove
   the non-polar hydrogens on-the-fly.

   ### Important

   Make sure that any non-standard atom names and substructure names are
   defined in `$RBT_ROOT/data/sf/RbtIonicAtoms.prm` in
   order for the assignment of distributed formal charges to work
   correctly.

   Make sure that the Tripos atom types are set correctly. rDock uses the
   Tripos types to derive other critical atomic properties such as atomic
   number and hybridisation state.

   ### Note

   The rDock MOL2 parser was developed to read the CCDC/Astex
   `protein.mol2` files, therefore this validation
   set is the *de facto* standard reference. You should
   compare against the format of the CCDC/Astex MOL2 files if you are in
   doubt as to whether a particular MOL2 file is suitable for rDock
2. Prepare a system definition file. At a minimum, you need to define the
   receptor parameters, the cavity mapping parameters (`SECTION
   MAPPER`) and the cavity restraint penalty (`SECTION
   CAVITY`). Make sure you define the `RECEPTOR_FLEX`
   parameter if you wish to activate sampling of terminal OH and NH3+ groups in
   the vicinity of the docking site.
3. Generate the docking site (.as) file using `rbcavity`.
   You will require a reference bound ligand structure in the coordinate space
   of the receptor if you wish to use the reference ligand cavity mapping
   method.
4. Prepare the ligand SD files you wish to dock, taking into account the atom
   typing issues described above for SD file parsing. In particular, make sure
   that formal charges and formal bond order are defined coherently so that
   there are no formal valence errors in the file. rDock will report any
   perceived valence errors but will dock the structures anyway. Note that
   rDock never samples bond lengths, bond angles, ring conformations, or
   non-rotatable bonds during docking so initial conformations should be
   reasonable.
5. Run a small test calculation to check that the system is defined
   correctly. For example, run `rbdock` from the command
   line with a small ligand SD file, with the score-only protocol (`-p
   score.prm`) and with the `-T 2` option to
   generate verbose output. The output will include receptor atom properties,
   ligand atom properties, flexibility parameters, scoring function parameters
   and docking protocol parameters.
6. When satisfied, launch the full-scale calculations. A description of the
   various means of launching rDock is beyond the scope of this guide.

## Tethered scaffold docking

In tethered scaffold docking, the ligand poses are restricted and forced to
overlay the substructure coordinates of a reference ligand. The procedure is largely
as for standard docking, except that:

- Ligand SD files must be prepared with the
  `rbtether` utility to annotate each record with the
  matching substructure atom indices, and to transform the coordinates of
  each ligand so that the matching substructure coordinates are overlaid
  with the reference substructure coordinates. This requires a Daylight
  SMARTS toolkit license.
- The system definition file should contain a `SECTION
  LIGAND` to define which of the the ligand degrees of freedom
  should be tethering to their reference values. Tethering can be applied
  to position, orientation and dihedral degrees of freedom independently.
  Note that the tethers are applied directly within the chromosome
  representation used by the search engine (where they affect the
  randomisation and mutation operators), and therefore external restraint
  penalty functions to enforce the tethers are not required.

  ### Important

  The reference state values for each tethered degree of freedom are
  defined directly from the initial conformation of each ligand as
  read from the input SD file, and *not* from the
  reference SD file used by `rbtether`. This is why
  the ligand coordinates are transformed by
  `rbtether`, such that each ligand record can act
  as its own reference state. The reference SD file used by
  `rbtether` is not referred to by the docking
  calculation itself.

  It follows from the above that tethered ligand docking is
  inappropriate for input ligand SD files that have not already been
  transformed to the coordinate space of the docking site, either by
  `rbtether` or by some other means.

  **Example 5.1. Example ligand definition for tethered scaffold**

  This definition will tether the position and orientation of the
  tethered substructure, but will allow free sampling of ligand
  dihedrals.

  ```
   SECTION LIGAND
      TRANS_MODE TETHERED
      ROT_MODE TETHERED
      DIHEDRAL_MODE FREE
      MAX_TRANS 1.0
      MAX_ROT 30.0
  END_SECTION
  ```

## Docking with pharmacophore restraints

In pharmacophore restrained docking, ligand poses are biased to fit user-defined
pharmacophore points. The bias is introduced through the use of an external penalty
restraint, which penalises docking poses that do not match the pharmacophore
restraints. Unlike tethered scaffold docking, there is no modification to the
chromosome operators themselves, hence the search can be inefficient, particularly
for large numbers of restraints and/or for ligands with large numbers of matching
features. Pre-screening of ligands is based purely on feature counts, and not on
geometric match considerations.

The implementation supports both mandatory and optional pharmacophore restraints.
The penalty function is calculated over all mandatory restraints, and over (any
`NOPT` from `N`) of the optional restraints.
For example, you may wish to ensure that any 4 from 7 optional restraints are
satisfied in the generated poses.

The procedure is largely as for standard docking, except that:

- You should prepare separate pharmacophore restraint files for the
  mandatory and optional restraints. Note that optional restraints do not
  have to be defined, it is sufficient to only define at least one
  mandatory restraint.
- The system definition file should contain a `SECTION
  PHARMA` to add the pharmacophore restraint penalty to the
  scoring function.

## Docking with explicit waters

Explicit structural waters can be loaded from an external PDB file, independently
from the main receptor model, by adding a `SECTION SOLVENT` to the
system definition file. The user has fine control over the flexibility of each water
molecule. A total of 9 flexibility modes are possible, in which the translational
and rotational degrees of freedom of each water can be set independently to
`FIXED`, `TETHERED`, or
`FREE`. Thus, for example, it is possible to define a water with a
fixed oxygen coordinate (presumably at a crystallographically observed position),
but freely rotating such that the orientation of the water hydrogens can be
optimised by the search engine (and can be ligand-dependent).

### Note

In the current implementation, *solvent* refers
strictly to *water* molecules, and the format of the
water PDB file is very strictly defined. In future implementations it is
anticipated that other, larger (and possibly flexible) molecules will be
loadable as solvent, and that other file formats will be supported.

**Procedure 5.2. Explicit waters workflow**

1. Prepare a separate PDB file for the explicit waters according to the
   format prescribed (the section called “Water PDB file format”)
2. Add a `SECTION SOLVENT` to the system definition file
   and define the relevant flexibility parameters (Table 8.3, “Solvent definition parameters”). The minimal requirement is to define the
   `FILE` parameter.
3. Decide whether you wish to have different per-solvent flexibility modes
   (defined via the occupancy values and temperature factor values in the PDB
   file (Table 9.1, “Conversion of temperature factor values to solvent flexibility modes”)), or whether you wish to have a
   single flexibility mode applied to all waters (defined via the
   `TRANS_MODE` and `ROT_MODE` values in
   the `SECTION SOLVENT` of the receptor .prm file)

   ### Important

   If you wish to use per-solvent flexibility modes (that is, you
   wish to set different modes for different waters) make sure that you
   do not define `TRANS_MODE` or
   `ROT_MODE` entries in the `SECTION
   SOLVENT` as these values will override the per-solvent
   values derived from the temperature factors in the PDB
   file.
4. If you have defined any waters with `TETHERED`
   translational or rotational degrees of freedom, define
   `MAX_TRANS` and/or `MAX_ROT` values as
   appropriate (or accept the default values. The tethered ranges are applied
   to all tethered waters and can not be defined on a per-solvent basis at
   present.

## Chapter 6. Scoring function reference

Scoring functions for docking are constructed at run-time (by class
`RbtSFFactory`) from scoring function definition files (rDock
.prm format). The default location for scoring function definition files is
`$RBT_ROOT/data/sf/`.

The total score is an aggregate of intermolecular ligand-receptor and
ligand-solvent interactions (branch `SCORE.INTER`), intra-ligand
interactions (branch `SCORE.INTRA`), intra-receptor, intra-solvent and
receptor-solvent interactions (branch `SCORE.SYSTEM`), and external
restraint penalties (branch `SCORE.RESTR`).

The `SCORE.INTER`, `SCORE.INTRA` and
`SCORE.SYSTEM` branches consist of weighted sums of interaction
terms as shown below. Note that not all terms appear in all branches. See the rDock
draft paper for more details on the implementation of these terms.

**Table 6.1. Scoring function terms and C++ implementation classes**

| Term | Description | INTER | INTRA | SYSTEM |
| --- | --- | --- | --- | --- |
| VDW | van der Waals | `RbtVdwIdxSF` | `RbtVdwIntraSF` | `RbtVdwIdxSF` |
| VDW | van der Waals (grid-based) | `RbtVdwGridSF` | N/A | N/A |
| POLAR | Attractive polar | `RbtPolarIdxSF` | `RbtPolarIntraSF` | `RbtPolarIdxSF` |
| REPUL | Repulsive polar | `RbtPolarIdxSF` | `RbtPolarIntraSF` | `RbtPolarIdxSF` |
| SOLV | Desolvation | `RbtSAIdxSF` | `RbtSAIdxSF` | `RbtSAIdxSF` |
| DIHEDRAL | Dihedral potential | N/A | `RbtDihedralIntraSF` | `RbtDihedralTargetSF` |
| CONST | Translation/rotational binding entropy penalty | `RbtConstSF` | N/A | `RbtConstSF` |
| ROT | Torsional binding entropy penalty | `RbtRotSF` | N/A | N/A |

Two intermolecular scoring functions (`SCORE.INTER` branch) have
been validated. These are known informally as the standard scoring function and the
desolvation scoring function (referred to as SF3 and SF5 respectively in the rDock draft
paper). The standard intermolecular scoring function consists of `VDW`,
`POLAR` and `REPUL` terms. In the desolvation
scoring function, the `REPUL` term is replaced by a more finely
parameterised desolvation potential (`SOLV` term) based on a Weighted
Solvent-Accessible Surface Area (WSAS) model. The ligand intramolecular scoring function
(`SCORE.INTRA` branch) remains constant in both cases, and has the
same terms and weights as the standard intermolecular scoring function.

**Table 6.2. Scoring function data files**

| File | Description |
| --- | --- |
| RbtInterIdxSF.prm | Intermolecular scoring function definition (standard scoring function, SF3) |
| RbtInterGridSF.prm | As above, but vdW term uses a precalculated grid |
| RbtSolvIdxSF.prm | Intermolecular scoring function definition (desolvation scoring function, SF5) |
| RbtSolvGridSF.prm | As above, but vdW term uses a precalculated grid |
| RbtIntraSF.prm | Ligand intramolecular scoring function definition |
| RbtTargetSF.prm | System scoring function definition (just contains SCORE.SYSTEM.DIHEDRAL, see Notes below) |
| calcgrid\_vdw1.prm | vdW term only (`ECUT=1`), for calculating vdW grid (used by `rbcalcgrid`) |
| calcgrid\_vdw5.prm | vdW term only (`ECUT=5`), for calculating vdW grid (used by `rbcalcgrid`) |
| Tripos52\_vdw.prm | vdW term parameter file |
| Tripos52\_dihedrals.prm | Dihedral term parameter file |
| solvation\_asp.prm | Desolvation term parameter file |

### Note

External restraint penalty terms are defined by the user in the system
definition .prm file.

Originally, rDock did not support flexible receptor dihedrals or explicit
structural waters, and the overall scoring function consisted of just the
`SCORE.INTER` and `SCORE.INTRA` branches. At
that time, the intermolecular scoring function definition file (e.g.
`RbtInterIdxSF.prm`) represented precisely the SCORE.INTER
terms, and the intramolecular definition file (`RbtIntraSF.prm`)
represented precisely the SCORE.INTRA terms. With the introduction of receptor
flexibility and explicit structural waters (and hence the need for the
`SCORE.SYSTEM` branch), the situation is slightly more complex.
For implementation reasons, many of the terms reported under
`SCORE.SYSTEM` (with the exception of the dihedral term) are
calculated simultaneously with the terms reported under
`SCORE.INTER`, and hence their parameterisation is defined
implicitly in the intermolecular scoring function definition file. In contrast, the
ligand intramolecular scoring function terms can be controlled independently.

## Chapter 7. Docking protocol reference

Docking protocols are constructed at run-time (by class
`RbtTransformFactory`) from docking protocol definition files
(rDock .prm format). The default location for docking protocol files is
`$RBT_ROOT/data/scripts/`.

The docking protocol definition file defines the sequence of search algorithms that
constitute a single docking run for a single ligand record. Each search algorithm
component operates either on a single chromosome representing the system degrees of
freedom, or on a population of such chromosomes.

The chromosome is constructed (by `RbtChromFactory`) as an
aggregate of individual chromosome elements for the receptor, ligand and explicit
solvent degrees of freedom, as defined by the flexibility parameters in the system
definition file.

**Table 7.1. Chromsome elements**

| Element | Defined by | Class | Length |
| --- | --- | --- | --- |
| Position | Centre of mass | `RbtChromPositionElement` | 3 |
| Orientation | Euler angles for principal axes | `RbtChromPositionElement` | 3 |
| Dihedral | Dihedral angle for rotatable bond | `RbtChromDihedralElement` | 1 per bond |
| Occupancy | Explicit water occupancy state | `RbtChromOccupancylElement` | 1 per water |

**Table 7.2. Search algorithm components and C++ implementation classes**

| Component | Class | Operates on |
| --- | --- | --- |
| Randomise population | `RbtRandPopTransform` | Chromosome population |
| Genetic algorithm search | `RbtGATransform` | Chromosome population |
| Monte Carlo simulated annealing | `RbtSimAnnTransform` | Single chromosome |
| Simplex minimisation | `RbtSimplexTransform` | Single chromosome |
| Null operation | `RbtNullTransform` | N/A |

**Table 7.3. Docking protocol data files**

| File | Description |
| --- | --- |
| score.prm | Calculates score only for initial conformation (standard scoring function) |
| score\_solv.prm | As above, but uses desolvation scoring function |
| minimise.prm | Simplex minimisation of initial conformation (standard scoring function) |
| minimise\_solv.prm | As above, but uses desolvation scoring function |
| dock.prm | Full docking search (standard scoring function) |
| dock\_solv.prm | As above, but uses desolvation scoring function |
| dock\_grid.prm | Full docking search (standard scoring function, grid-based vdW term) |
| dock\_solv\_grid.prm | Full docking search (desolvation scoring function, grid-based vdW term) |

The standard rDock docking protocol consists of three phases of a Genetic Algorithm
search, followed by low-temperature Monte Carlo and Simplex minimisation. By way of
example, the `dock.prm` script is documented in detail. The other
scripts are very similar.

**Example 7.1. Standard rDock docking protocol (`dock.prm`)**

```
SECTION SCORE
    INTER    RbtInterIdxSF.prm
    INTRA    RbtIntraSF.prm
    SYSTEM   RbtTargetSF.prm
END_SECTION
```

The scoring function definition is referenced within the docking protocol
definition file itself, in the `SCORE` section. This section
contains entries for the `INTER`, `INTRA` and
`SYSTEM` scoring function definition files.

```
SECTION SETSLOPE_1
    TRANSFORM                       RbtNullTransform
    WEIGHT@SCORE.RESTR.CAVITY       5.0     # Dock with a high penalty for leaving the cavity
    WEIGHT@SCORE.INTRA.DIHEDRAL     0.1     # Gradually ramp up dihedral weight from 0.1->0.5
    ECUT@SCORE.INTER.VDW            1.0     # Gradually ramp up energy cutoff for switching to quadratic
    USE_4_8@SCORE.INTER.VDW         TRUE    # Start docking with a 4-8 vdW potential
    DA1MAX@SCORE.INTER.POLAR        180.0   # Broader angular dependence
    DA2MAX@SCORE.INTER.POLAR        180.0   # Broader angular dependence
    DR12MAX@SCORE.INTER.POLAR       1.5     # Broader distance range
END_SECTION
```

All sections that contain the `TRANSFORM` parameter are
interpreted as a search algorithm component. The value of the
`TRANSFORM` parameter is the C++ implementation class name for
that transform. An `RbtNullTransform` can be used to send
messages to the scoring function to modify key scoring function parameters in
order to increase search efficiency. All parameter names that contain the
`@` symbol are interpreted as scoring function messages,
where the string before the `@` is the scoring function
parameter name, the string after the `@` is the scoring
function term, and the parameter value is the new value for the scoring function
parameter. Messages are sent blind, with no success feedback, as the the docking
protocol has no knowledge of the composition of the scoring function terms.

Here, we start the docking with a soft 4-8 vdW potential, a reduced dihedral
potential, and extended polar ranges (distances and angles) for the
intermolecular polar potential. These changes are all designed to aid sampling
efficiency by not overpenalising bad contacts in the initial, randomised
population, and by encouraging the formation of intermolecular hydrogen bonds.

```
SECTION RANDOM_POP
    TRANSFORM                       RbtRandPopTransform
    POP_SIZE                        50
    SCALE_CHROM_LENGTH              TRUE
END_SECTION
```

Creates an initial, randomised chromosome population. If
`SCALE_CHROM_LENGTH` is false, the population is of fixed
size, defined by `POP_SIZE`. If
`SCALE_CHROM_LENGTH` is true, the population is proportional to
the overall chromosome length, defined by `POP_SIZE` multiplied
by the chromosome length.

```
SECTION GA_SLOPE1
    TRANSFORM                       RbtGATransform
    PCROSSOVER                      0.4     # Prob. of crossover
    XOVERMUT                        TRUE    # Cauchy mutation after each crossover
    CMUTATE                         FALSE   # True = Cauchy; False = Rectang. for regular mutations
    STEP_SIZE                       1.0     # Max relative mutation
END_SECTION
```

First round of GA.

```
SECTION SETSLOPE_3
    TRANSFORM                       RbtNullTransform
    WEIGHT@SCORE.INTRA.DIHEDRAL     0.2
    ECUT@SCORE.INTER.VDW            5.0
    DA1MAX@SCORE.INTER.POLAR        140.0
    DA2MAX@SCORE.INTER.POLAR        140.0
    DR12MAX@SCORE.INTER.POLAR       1.2
END_SECTION
```

Increases the ligand dihedral weight, increases the short-range intermolecular
vdW hardness (`ECUT`), and decreases the range of the
intermolecular polar distances and angles.

```
SECTION GA_SLOPE3
    TRANSFORM                       RbtGATransform
    PCROSSOVER                      0.4     # Prob. of crossover
    XOVERMUT                        TRUE    # Cauchy mutation after each crossover
    CMUTATE                         FALSE   # True = Cauchy; False = Rectang. for regular mutations
    STEP_SIZE                       1.0     # Max relative mutation
END_SECTION
```

Second round of GA with revised scoring function parameters.

```
SECTION SETSLOPE_5
    TRANSFORM                       RbtNullTransform
    WEIGHT@SCORE.INTRA.DIHEDRAL     0.3
    ECUT@SCORE.INTER.VDW            25.0
    USE_4_8@SCORE.INTER.VDW         FALSE   # Now switch to a convential 6-12 for final GA, MC, minimisation
    DA1MAX@SCORE.INTER.POLAR        120.0
    DA2MAX@SCORE.INTER.POLAR        120.0
    DR12MAX@SCORE.INTER.POLAR       0.9
END_SECTION
```

Further increases the ligand dihedral weight, further increases the
short-range intermolecular vdW hardness (`ECUT`), and further
decreases the range of the intermolecular polar distances and angles. Also
switches from softer 4-8 vdW potential to a harder 6-12 potential for final
round of GA, MC and minimisation.

```
SECTION GA_SLOPE5
    TRANSFORM                       RbtGATransform
    PCROSSOVER                      0.4     # Prob. of crossover
    XOVERMUT                        TRUE    # Cauchy mutation after each crossover
    CMUTATE                         FALSE   # True = Cauchy; False = Rectang. for regular mutations
    STEP_SIZE                       1.0     # Max relative mutation
END_SECTION
```

Final round of GA with revised scoring function parameters.

```
SECTION SETSLOPE_10
    TRANSFORM                       RbtNullTransform
    WEIGHT@SCORE.INTRA.DIHEDRAL     0.5     # Final dihedral weight matches SF file
    ECUT@SCORE.INTER.VDW            120.0   # Final ECUT matches SF file
    DA1MAX@SCORE.INTER.POLAR        80.0
    DA2MAX@SCORE.INTER.POLAR        100.0
    DR12MAX@SCORE.INTER.POLAR       0.6
END_SECTION
```

Resets all the modified scoring function parameters to their final values,
corresponding to the values in the scoring function definition files. It is
important that the final scoring function optimised by the docking search can be
compared directly with the score-only and minimisation-only protocols, in which
the scoring function parameters are not modified.

```
SECTION MC_10K
    TRANSFORM                       RbtSimAnnTransform
    START_T                         10.0
    FINAL_T                         10.0
    NUM_BLOCKS                      5
    STEP_SIZE                       0.1
    MIN_ACC_RATE                    0.25
    PARTITION_DIST                  8.0
    PARTITION_FREQ                  50
    HISTORY_FREQ                    0
END_SECTION
```

Low temperature Monte Carlo sampling, starting from fittest chromosome from
final round of GA.

```
SECTION SIMPLEX
    TRANSFORM                       RbtSimplexTransform
    MAX_CALLS                       200
    NCYCLES                         20
    STOPPING_STEP_LENGTH            10e-4
    PARTITION_DIST                  8.0
    STEP_SIZE                       1.0
    CONVERGENCE                     0.001
END_SECTION
```

Simplex minimisation, starting from fittest chromosome from low temperature
Monte Carlo sampling.

```
SECTION FINAL
    TRANSFORM                       RbtNullTransform
    WEIGHT@SCORE.RESTR.CAVITY       1.0     # revert to standard cavity penalty
END_SECTION
```

Finally, we reset the cavity restraint penalty to 1. The penalty has been held
at a value of 5 throughout the search, to strongly discourage the ligand from
leaving the docking site.

## Chapter 8. System definition file reference

**Table of Contents**

Receptor definition

Ligand definition

Solvent definition

Cavity mapping

Cavity restraint

Pharmacophore restraints

NMR restraints

Example system definition files

Although known previously as the *receptor* .prm file, the
system definition file has evolved to contain much more than the receptor information.
The system definition file is used to define:

- Receptor input files and flexibility parameters (the section called “Receptor definition”)
- Explicit solvent input file and flexibility parameters (the section called “Solvent definition”)
- Ligand flexibility parameters (the section called “Ligand definition”).
- External restraint terms to be added to the total scoring function (e.g.
  cavity restraint, pharmacophoric restraint)

## Receptor definition

The receptor can be loaded from a single MOL2 file, or from a combination of
Charmm PSF and CRD files. In the former case the MOL2 file provides the topology and
reference coordinates simultaneously, whereas in the latter case the topology is
loaded from the PSF file and the reference coordinates from the CRD file.

For historical compatibility reasons, receptor definition parameters are all
defined in the top-level namespace and should not be placed between
`SECTION`..`END_SECTION` pairs.

### Caution

If MOL2 and PSF/CRD parameters are defined together, the MOL2 parameters
take precedence and are used to load the receptor model.

**Table 8.1. Receptor definition parameters**

| Parameter | Description | Type | Default | Range of values |
| --- | --- | --- | --- | --- |
| Parameters specific to loading receptor in MOL2 file format. | | | | |
| `RECEPTOR_FILE` | Name of receptor MOL2 file | Filename string | No default value | Valid MOL2 filename |
| Parameters specific to loading receptor in Charmm PSF/CRD file format | | | | |
| `RECEPTOR_TOPOL_FILE` | Name of receptor Charmm PSF file | Filename string | No default value | Valid Charmm PSF filename |
| `RECEPTOR_COORD_FILE` | Name of receptor Charmm CRD file | Filename string | No default value | Valid Charmm CRD filename |
| `RECEPTOR_MASSES_FILE` | Name of rDock-annotated Charmm masses file | Filename string | No default value | `masses.rtf`; `top_all2_prot_na.inp` |
| General receptor parameters, applicable to either file format | | | | |
| `RECEPTOR_SEGMENT_NAME` | List of molecular segment names to read from either MOL2 or PSF/CRD file. If this parameter is defined, then any segment/chains not listed are not loaded. This provides a convenient way to remove cofactors, counterions and solvent without modifying the original file. | Comma-separated list of segment name strings (without any spaces) | Empty (i.e. all segments read from file) | Comma-separated list of segment name strings |
| `RECEPTOR_FLEX` | Defines terminal OH and NH3+ groups within this distance of docking volume as flexible. | float (Angstroms) | Undefined (rigid receptor) | > 0.0 (3.0 is a reasonable value) |
| Advanced parameters (should not need to be changed by the user) | | | | |
| `RECEPTOR_ALL_H` | Disables the removal of explicit non-polar hydrogens from the receptor model. *Not recommended* | boolean | FALSE | TRUE or FALSE |
| `DIHEDRAL_STEP` | Maximum mutation step size for receptor dihedral degrees of freedom | float (degrees) | 30.0 | >0.0 |

## Ligand definition

Ligand definition parameters need only be defined if you wish to introduce
tethering of some or all of the ligand degrees of freedom. If you are running
conventional free docking then this section is not required. All ligand definition
parameters should be defined in `SECTION LIGAND`. Note that the
ligand input SD file continues to be specified directly on the rbdock command-line
and not in the system definition file.

**Table 8.2. Ligand definition parameters**

| Parameter | Description | Type | Default | Range of values |
| --- | --- | --- | --- | --- |
| Main user parameters | | | | |
| `TRANS_MODE` | Sampling mode for ligand translational degrees of freedom | enumerated string literal | `FREE` | `FIXED`; `TETHERED`; `FREE` |
| `ROT_MODE` | Sampling mode for ligand whole-body rotational degrees of freedom | enumerated string literal | `FREE` | `FIXED`; `TETHERED`; `FREE` |
| `DIHEDRAL_MODE` | Sampling mode for ligand internal dihedral degrees of freedom | enumerated string literal | `FREE` | `FIXED`; `TETHERED`; `FREE` |
| `MAX_TRANS` | (for `TRANS_MODE` = `TETHERED` only) Maximum deviation allowed from reference centre of mass | float (Angstroms) | 1.0 | >0.0 |
| `MAX_ROT` | (for `ROT_MODE` = `TETHERED` only) Maximum deviation allowed from orientation of reference principle axes | float (degrees) | 30.0 | >0.0 - 180.0 |
| `MAX_DIHEDRAL` | (for `DIHEDRAL_MODE` = `TETHERED` only) Maximum deviation allowed from reference dihedral angles for any rotatable bond | float (degrees) | 30.0 | >0.0 - 180.0 |
| Advanced parameters (should not need to be changed by the user) | | | | |
| `TRANS_STEP` | Maximum mutation step size for ligand translational degrees of freedom | float (Angstroms) | 2.0 | >0.0 |
| `ROT_STEP` | Maximum mutation step size for ligand whole-body rotational degrees of freedom | float (degrees) | 30.0 | >0.0 |
| `DIHEDRAL_STEP` | Maximum mutation step size for ligand internal dihedral degrees of freedom | float (degrees) | 30.0 | >0.0 |

## Solvent definition

Solvent definition parameters need only be defined if you wish to introduce
explicit structural waters into the docking calculation, otherwise this section is
not required. All solvent definition parameters should be defined in
`SECTION SOLVENT`.

**Table 8.3. Solvent definition parameters**

| Parameter | Description | Type | Default | Range of values |
| --- | --- | --- | --- | --- |
| Main user parameters | | | | |
| `FILE` | Name of explicit solvent PDB file | File name string | No default value (mandatory parameter) | Valid PDB filename |
| `TRANS_MODE` | Sampling mode for solvent translational degrees of freedom. If defined here, the value overrides the per-solvent translational sampling modes defined in the solvent PDB file | enumerated string literal | `FREE` | `FIXED`; `TETHERED`; `FREE` |
| `ROT_MODE` | Sampling mode for solvent whole-body rotational degrees of freedom. If defined here, the value overrides the per-solvent rotational sampling modes defined in the solvent PDB file | enumerated string literal | `FREE` | `FIXED`; `TETHERED`; `FREE` |
| `MAX_TRANS` | (for `TRANS_MODE` = `TETHERED` waters only) Maximum deviation allowed from reference water oxygen positions. The same value is applied to all waters with `TRANS_MODE` = `TETHERED`; it is not possible currently to define per-solvent `MAX_TRANS` values. | float (Angstroms) | 1.0 | >0.0 |
| `MAX_ROT` | (for `ROT_MODE` = `TETHERED` waters only) Maximum deviation allowed from orientation of reference principal axes. The same value is applied to all waters with `ROT_MODE` = `TETHERED`; it is not possible currently to define per-solvent `MAX_ROT` values. | float (degrees) | 30.0 | >0.0 - 180.0 |
| `OCCUPANCY` | Controls occupany state sampling for all explicit solvent. If defined here, the value overrides the per-solvent occupancy states defined in the solvent PDB file. | float | 1.0 | 0.0 - 1.0 |
| Advanced parameters (should not need to be changed by the user) | | | | |
| `TRANS_STEP` | Maximum mutation step size for solvent translational degrees of freedom | float (Angstroms) | 2.0 | >0.0 |
| `ROT_STEP` | Maximum mutation step size for solvent whole-body rotational degrees of freedom | float (degrees) | 30.0 | >0.0 |
| `OCCUPANCY_STEP` | Maximum mutation step size for solvent occupancy state degrees of freedom. | float (degrees) | 1.0 | 0.0 - 1.0 |

**Solvent occupancy state sampling.** `OCCUPANCY` = 0 permanently disables all solvent;
`OCCUPANCY` = 1.0 permanently enables all solvent;
`OCCUPANCY` between 0 and 1 activates variable occupancy
state sampling, where the value represents the initial probability that the
solvent molecule is enabled. For example, `OCCUPANCY` = 0.5
means that the solvent is enabled in 50% of the initial GA population. However,
the probability that the solvent is actually enabled in the final docking
solution will depend on the particular ligand, the scoring function terms, and
on the penalty for solvent binding. The occupancy state chromosome value is
managed as a continuous variable between 0.0 and 1.0, with a nominal mutation
step size of 1.0. Chromosome values lower than the occupancy threshold (defined
as `1.0 - OCCUPANCY`) result in the solvent being disabled;
values higher than the threshold result in the solvent being enabled

## Cavity mapping

The cavity mapping section is mandatory. You should choose one of the mapping
algorithms shown below. All mapping parameters should be defined in `SECTION
MAPPER`.

**Table 8.4. Two sphere site mapping parameters**

| Parameter | Description | Type | Default | Range of values |
| --- | --- | --- | --- | --- |
| Main user parameters | | | | |
| `SITE_MAPPER` | Mapping algorithm specifier | string literal | `RbtSphereSiteMapper` | fixed |
| `CENTER` | (x,y,z) center of cavity mapping region | Bracketed cartesian coordinate string (x,y,z) | None | None |
| `RADIUS` | Radius of cavity mapping region | float (Angstroms) | 10.0 | > 0.0 (10.0-20.0 suggested range) |
| `SMALL_SPHERE` | Radius of small probe | float (Angstroms) | 1.5 | > 0.0 (1.0-2.0 suggested range) |
| `LARGE_SPHERE` | Radius of large probe | float (Angstroms) | 4.0 | > `SMALL_SPHERE` (3.5 - 6.0 suggested range) |
| `MAX_CAVITIES` | Maximum number of cavities to accept (in descending order of size) | integer | 99 | >0 |
| Advanced parameters (less frequently changed by the user) | | | | |
| `VOL_INCR` | Receptor atom radius increment for excluded volume | float (Angstroms) | 0.0 | >= 0.0 |
| `GRID_STEP` | Grid resolution for mapping | float (Angstroms) | 0.5 | >0.0 (0.3 - 0.8 suggested range) |
| `MIN_VOLUME` | Minimum cavity volume to accept (in A^3, not grid points) | float (Angstroms^3) | 100 | >0 (100-300 suggested range) |

**Table 8.5. Reference ligand site mapping parameters**

| Parameter | Description | Type | Default | Range of values |
| --- | --- | --- | --- | --- |
| Main user parameters | | | | |
| `SITE_MAPPER` | Mapping algorithm specifier | string literal | `RbtLigandSiteMapper` | fixed |
| `REF_MOL` | Reference ligand SD file name | string | ref.sd | None |
| `RADIUS` | Radius of cavity mapping region | float (Angstroms) | 10.0 | > 0.0 (10.0-20.0 suggested range) |
| `SMALL_SPHERE` | Radius of small probe | float (Angstroms) | 1.5 | > 0.0 (1.0-2.0 suggested range) |
| `LARGE_SPHERE` | Radius of large probe | float (Angstroms) | 4.0 | > `SMALL_SPHERE` (3.5 - 6.0 suggested range) |
| `MAX_CAVITIES` | Maximum number of cavities to accept (in descending order of size) | integer | 99 | >0 |
| Advanced parameters (less frequently changed by the user) | | | | |
| `VOL_INCR` | Receptor atom radius increment for excluded volume | float (Angstroms) | 0.0 | >= 0.0 |
| `GRID_STEP` | Grid resolution for mapping | float (Angstroms) | 0.5 | >0.0 (0.3 - 0.8 suggested range) |
| `MIN_VOLUME` | Minimum cavity volume to accept (in A^3, not grid points) | float (Angstroms^3) | 100 | >0 (100-300 suggested range) |

## Cavity restraint

The cavity restraint penalty function is mandatory and is designed to prevent the
ligand from exiting the docking site. The function is calculated over all
non-hydrogen atoms in the ligand (and over all explicit water oxygens that can
translate). The distance from each atom to the nearest cavity grid point is
calculated. If the distance exceeds the value of `RMAX`, a penalty
is imposed based on the value of (distance - `RMAX`). The penalty
can be either linear or quadratic depending on the value of the
`QUADRATIC` parameter.

It should not be necessary to change any the parameters in this section. Note that
the docking protocol itself will manipulate the `WEIGHT` parameter,
so any changes made to `WEIGHT` will have no effect.

```
SECTION CAVITY
    SCORING_FUNCTION        RbtCavityGridSF
    WEIGHT                  1.0
    RMAX                    0.1
    QUADRATIC               FALSE
END_SECTION
```

## Pharmacophore restraints

This section need only be defined if you wish to dock with pharmacophore
restraints. If you are running conventional free docking then this section is not
required. All pharmacophore definition parameters should be defined in
`SECTION PHARMA`.

**Table 8.6. Pharmacophore restraint parameters**

| Parameter | Description | Type | Default | Range of values |
| --- | --- | --- | --- | --- |
| `CONSTRAINTS_FILE` | Mandatory pharmacophore restraints file | File name string | None (mandatory parameter) | Valid file name |
| `OPTIONAL_FILE` | Optional pharmacophore restraints file | File name string | None (optional parameter) | Valid file name, or empty |
| NOPT | Number of optional restraints that should be met | Integer | 0 | Between 0 and number of restraints in `OPTIONAL_FILE` |
| WRITE\_ERRORS | Ligands with insufficient pharmacophore features to match the mandatory restraints are always removed prior to docking. If this parameter is true, the pre-filtered ligands are written to an error SD file with the same root name as the docked pose output SD file, but with an `_errors.sd` suffix. If false, the pre-filtered ligands are not written. | Boolean | `false` | `true` or `false` |
| WEIGHT | Overall weight for the pharmacophore penalty function | Float | 1.0 | >= 0.0 |

**Calculation of mandatory restraint penalty.** The list of ligand atoms that matches each restraint type in the mandatory
restraints file is precalculated for each ligand as it is loaded. If the ligand
contains insufficient features to satisfy all of the mandatory restraints the
ligand is rejected and is not docked. Note that the rejection is based purely on
feature counts and does not take into account the possible geometric
arrangements of the features. Rejected ligands are optionally written to an
error SD file. The penalty for each restraint is based on the distance from the
nearest matching ligand atom to the pharmacophore restraint centre. If the
distance is less than the defined tolerance (restraint sphere radius), the
penalty is zero. If the distance is greater than the defined tolerance a
quadratic penalty is applied, equal to (nearest distance - tolerance)^2.

**Calculation of optional restraint penalty.** The individual restraint penalties for each restraint in the optional
restraints file are calculated in the same way as for the mandatory penalties.
However, only the `NOPT` lowest scoring (least penalised)
restraints are summed for any given docking pose. Any remaining higher scoring
optional restraints are ignored and do not contribute to the total pharmacophore
restraint penalty.

**Calculation of overall restraint penalty.** The overall pharmacophore restraint penalty is the sum of the mandatory
restraint penalties and the `NOPT` lowest scoring optional
restraint penalties, multiplied by the `WEIGHT` parameter
value.

## NMR restraints

To be completed. However, this feature has rarely been used.

## Example system definition files

**Example 8.1. Full system definition file with all sections and common parameters
enumerated explicitly**

```
RBT_PARAMETER_FILE_V1.00
TITLE HSP90-PU3-lig-cavity, solvent flex=5

RECEPTOR_FILE PROT_W3_flex.mol2
RECEPTOR_SEGMENT_NAME PROT
RECEPTOR_FLEX 3.0

SECTION SOLVENT
    FILE PROT_W3_flex_5.pdb
    TRANS_MODE TETHERED
    ROT_MODE TETHERED
    MAX_TRANS 1.0
    MAX_ROT 30.0
    OCCUPANCY 0.5
END_SECTION

SECTION LIGAND
    TRANS_MODE FREE
    ROT_MODE FREE
    DIHEDRAL_MODE FREE
    MAX_TRANS 1.0
    MAX_ROT 30.0
    MAX_DIHEDRAL 30.0
 END_SECTION
                
SECTION MAPPER
    SITE_MAPPER RbtLigandSiteMapper
    REF_MOL ref.sd
    RADIUS 5.0
    SMALL_SPHERE 1.0
    MIN_VOLUME 100
    MAX_CAVITIES 1
    VOL_INCR 0.0
    GRIDSTEP 0.5
END_SECTION

SECTION CAVITY
    SCORING_FUNCTION RbtCavityGridSF
    WEIGHT 1.0
END_SECTION

SECTION PHARMA
    SCORING_FUNCTION RbtPharmaSF
    WEIGHT 1.0
    CONSTRAINTS_FILE mandatory.const
    OPTIONAL_FILE optional.const
    NOPT 3
    WRITE_ERRORS TRUE
END_SECTION
```

## Chapter 9. rDock file formats

**Table of Contents**

.prm file format

Water PDB file format

Pharmacophore restraints file format

## .prm file format

The .prm file format is an rDock-specific text format and is used for:

- system definition files (known previously as receptor .prm
  files)
- scoring function definition files
- search protocol definition files

The format is simple and allows for an arbitrary number of named
parameter/value pairs to be defined, optionally divided into named sections.
Sections provide a namespace for parameter names, to allow parameter names to be
duplicated within different sections.

The key features of the format are:

- The first line of the file must be
  `RBT_PARAMETER_FILE_V1.00` with no preceeding
  whitespace.
- Subsequent lines may contain either:

  1. comment lines
  2. reserved keywords `TITLE`,
     `SECTION`, or
     `END_SECTION`
  3. parameter name/value pairs
- Comment lines should start with a `#` character in
  the first column with no preceeding whitespace, and are
  ignored.
- The reserved words must start in the first column with no
  preceeding whitespace.
- The `TITLE` record should occur only once in the
  file and is used to provide a title string for display by various
  scripts such as run\_rbscreen.pl. The keyword should be followed by a
  single space character and then the title string, which may contain
  spaces. If the `TITLE` line occurs more than once, the
  last occurence is used.
- `SECTION` records can occur more than once, and
  should always be paired with a closing `END_SECTION`
  record. The keyword should be followed by a single space character and
  then the section name, which may NOT itself contain spaces. All section
  names must be unique within a .prm file. All parameter name/value pairs
  within the `SECTION` / `END_SECTION`
  block belong to that section.
- Parameter name/value pairs are read as free-format tokenised text
  and can have preceeding, trailing, and be separated by arbitrary
  whitespace. This implies that the parameter name and value strings
  themselves are not allowed to contain any spaces. The value strings are
  interpreted as numeric, string, or boolean values as appropriate for
  that parameter. Boolean values should be entered as
  `TRUE` or `FALSE` uppercase
  strings.

### Caution

The current implementation of the .prm file reader does not tolerate a
`TAB` character immediately following the
`TITLE` and `SECTION` keywords. It is very
important that the first character after the `SECTION` keyword
in particular is a true space character, otherwise the reserved word will not be
detected and the parameters for that section will be ignored.

**Example 9.1. Example .prm file**

In the following example, `RECEPTOR_FILE` is defined in the
top level namespace. The remaining parameters are defined in the
`MAPPER` and `CAVITY` namespaces. The
indentation is for readability, and has no significance in the format.

```
RBT_PARAMETER_FILE_V1.00
TITLE 4dfr oxido-reductase

RECEPTOR_FILE 4dfr.mol2

SECTION MAPPER
    SITE_MAPPER RbtLigandSiteMapper
    REF_MOL 4dfr_c.sd
    RADIUS 6.0
    SMALL_SPHERE 1.0
    MIN_VOLUME 100
    MAX_CAVITIES 1
    VOL_INCR 0.0
    GRIDSTEP 0.5
END_SECTION

SECTION CAVITY
    SCORING_FUNCTION RbtCavityGridSF
    WEIGHT 1.0
END_SECTION
```

## Water PDB file format

rDock requires explicit water PDB files to be in the style as output by the
Dowser program. In particular:

- Records can be HETATM or ATOM
- The atom names must be `OW`, `H1`
  and `H2`
- The atom records for each water molecule must belong to the same
  subunit ID
- The subunit IDs for different waters must be distinct, but do not
  have to be consecutive.
- The atom IDs are not used and do not have to be consecutive (they
  can even be duplicated)
- The order of the atom records within a subunit is
  unimportant
- The temperature factor field of the water oxygens can be used to
  define the per-solvent flexibility modes. The temperature factors of the
  water hydrogens are not used.

**Table 9.1. Conversion of temperature factor values to solvent flexibility modes**

| PDB temperature factor | Solvent translational flexibility | Solvent rotational flexibility |
| --- | --- | --- |
| 0 | `FIXED` | `FIXED` |
| 1 | `FIXED` | `TETHERED` |
| 2 | `FIXED` | `FREE` |
| 3 | `TETHERED` | `FIXED` |
| 4 | `TETHERED` | `TETHERED` |
| 5 | `TETHERED` | `FREE` |
| 6 | `FREE` | `FIXED` |
| 7 | `FREE` | `TETHERED` |
| 8 | `FREE` | `FREE` |

**Example 9.2. Valid rDock PDB file for explicit, flexible waters**

```
REMARK tmp_1YET.pdb xtal_hoh.pdb
HETATM 3540  OW  HOH W 106      28.929  12.684  20.864  1.00  1.0 
HETATM 3540  H1  HOH W 106      28.034  12.390  21.200  1.00
HETATM 3540  H2  HOH W 106      29.139  12.204  20.012  1.00
HETATM 3542  OW  HOH W 108      27.127  14.068  22.571  1.00  2.0 
HETATM 3542  H1  HOH W 108      26.632  13.344  23.052  1.00
HETATM 3542  H2  HOH W 108      27.636  13.673  21.806  1.00
HETATM 3679  OW  HOH W 245      27.208  10.345  27.250  1.00  3.0 
HETATM 3679  H1  HOH W 245      27.657  10.045  26.409  1.00
HETATM 3679  H2  HOH W 245      26.296  10.693  27.036  1.00
HETATM 3680  OW  HOH W 246      31.737  12.425  21.110  1.00  4.0 
HETATM 3680  H1  HOH W 246      31.831  12.448  22.106  1.00
HETATM 3680  H2  HOH W 246      30.775  12.535  20.863  1.00
```

## Pharmacophore restraints file format

Pharmacophore restraints are defined in a simple text file, with one restraint per
line. Each line should contain the following values, separated by commas or
whitespace:

*`x y z coords of restraint centre`*, *`tolerance
(in Angstroms)`*, *`restraint type string`*.

The supported restraint types are:

**Table 9.2. Pharmacophore restraint types**

| String | Description | Matches |
| --- | --- | --- |
| Any | Any atom | Any non-hydrogen atom |
| Don | H-bond donor | Any neutral donor hydrogen |
| Acc | H-bond acceptor | Any neutral acceptor |
| Aro | Aromatic | Any aromatic ring centre (pseudo atom) |
| Hyd | Hydrophobic | Any non-polar hydrogens (if present), any Csp3 or Ssp3, any C or S not bonded to Osp2, any Cl, Br, I |
| Hal | Hydrophobic, aliphatic | Subset of Hyd, sp3 atoms only |
| Har | Hydrophobic, aromatic | Subset of Hyd, aromatic atoms only |
| Ani | Anionic | Any atom with negative distributed formal charge |
| Cat | Cationic | Any atom with positive distributed formal charge |

## Chapter 10. rDock programs

**Table of Contents**

Executable (binary) programs

Scripts

Command-line reference

## Executable (binary) programs

**Table 10.1. Core rDock C++ executables**

| Executable | Used for | Description |
| --- | --- | --- |
| rbcavity | Preparation | Cavity mapping and preparation of docking site (.as) file |
| rbcalcgrid | Preparation | Calculation of vdW grid files (usually called by `make_grid.csh` wrapper script) |
| rbdock | Docking | The main rDock docking engine itself. |
| rbrms | Analysis | Calculation of ligand Root Mean Squared Displacement (RMSD) between reference and docked poses |

**Table 10.2. Auxiliary rDock C++ executables**

| Executable | Used for | Description |
| --- | --- | --- |
| rbtether | Preparation | Prepares a ligand SD file for tethered scaffold docking. Requires Daylight SMARTS license. Annotates ligand SD file with tethered substructure atom indices. |
| smart\_rms | Analysis | Calculation of ligand Root Mean Squared Displacement (RMSD) between reference and docked poses, taking into account ligand topological symmetry. Requires Daylight SMARTS license. |
| rbconvgrid | Analysis | Converts rDock vdW grids to InsightII grid format for visualisation |
| rbmoegrid | Analysis | Converts rDock vdW grids to MOE grid format for visualisation |
| rblist | Analysis | Outputs miscellaneous information for ligand SD file records |

## Scripts

**Table 10.3. Core scripts**

| Script | Used for | Description |
| --- | --- | --- |
| make\_grid.csh | Preparation | Creates the vdW grid files required for grid-based docking protocols (`dock_grid.prm` and `dock_solv_grid.prm`). Simple front-end to `rbcalcgrid`. |
| run\_rbdock.pl | Docking | Simple front-end to rbdock, suitable for small-scale docking experiments. Can create command files for a variety of queuing systems (Condor, SGE, NQS). |
| run\_rbscreen.pl | Docking | Front-end to rbdock for launching large-scale virtual screening campaigns. Creates a command file for Condor queuing system only. |
| sdfilter | Analysis | Utility for filtering SD files by arbitrary data field expressions. Useful for simple post-docking filtering by score components. |
| sdsort | Analysis | Utility for sorting SD files by arbitrary data field. Useful for simple post-docking filtering by score components. |
| sdreport | Analysis | Utility for reporting SD file data field values. Output in tab-delimited or csv format. |

**Table 10.4. Auxiliary scripts**

| Script | Used for | Description |
| --- | --- | --- |
| ht\_protocol\_finder.csh | Preparation | Used to optimise a high-throughput docking protocol from an initial exhaustive docking of a small representative ligand library. |
| sdsplit | Utility | Splits an SD file into multiple smaller SD files of fixed number of records. |
| sdmodify | Utility | Sets the molecule title line of each SD record equal to a given SD data field. |

## Command-line reference

## Name

rbdock — the rDock docking engine itself.

## Synopsis

`$RBT_ROOT/bin/rbdock`   
 {-i *`input ligand MDL SD file`*}  
 {-o *`output MDL SD file`*}  
 {-r *`system definition .prm file`*}  
 {-p *`docking protocol .prm file`*}  
 [-n *`number of docking
runs/ligand`*]  
 [-s *`random seed`*]  
 [-T *`debug trace level`*]  
 [[-t *`SCORE.INTER threshold`*] | [-t *`filter definition
file`*]]  
 [
`-ap`
`-an`
`-allH`
`-cont`
]

## Usage

**Simple exhaustive docking.** The minimum requirement for **rbdock** is to specify
the input (`-i`) and output (`-o`)
ligand SD file names, the system definition .prm file
(`-r`) and the docking protocol .prm file
(`-p`). This will perform one docking run per ligand
record in the input SD file and output all docked ligand poses to the
output SD file. Use `-n` to increase the number of
docking runs per ligand record.

**High-throughput docking 1.** The `-t` and `-cont` options can be
used to construct high-throughput protocols. If the argument following
`-t` is numeric it is interpreted as a threshold
value for `SCORE.INTER`, the total intermolecular score
between ligand and receptor/solvent. In the absence of
`-cont`, the threshold acts as an early termination
filter, and the docking runs for each ligand will be terminated early
once the threshold value has been exceeded. Note that the threshold is
applied only at the end of each individual docking run, not during the
runs themselves. If the `-cont` (continue) option is
specified as well, the threshold acts as an output pose filter instead
of a termination filter. The docking runs for each ligand run to
completion as in the exhaustive case, but only the docking poses that
exceed the threshold value of `SCORE.INTER` are written
to the output SD file.

**High throughput docking 2.** Alternatively, if the argument following `-t` is
non-numeric it is interpreted as a filter definition file. The filter
definition file can be used to define multiple termination filters and
multiple output pose filters in a generic way. Any docking score
component can be used in the filter definitions.
`run_rbscreen.pl` generates a filter definition
file for multi-stage, high-throughput docking, with progressive score
thresholds for early termination of poorly performing ligands. The use
of filter definition files is preferred over the more limited
`SCORE.INTER` filtering described above, whose use
is now deprecated.

**Automated ligand protonation/deprotonation.** The `-ap` option activates the automated protonation
of ligand positive ionisable centres, notably amines, guanidines,
imidazoles, and amidines. The `-an` option activates
the automated deprotonation of ligand negative ionisable centres,
notably carboxylic acids, phosphates, phosphonates, sulphates, and
sulphonates. The precise rules used by rDock for protonation and
deprotonation are quite crude, and are not user-customisable. Therefore
these flags are not recommended for detailed validation experiments, in
which care should be taken that the ligand protonation states are set
correctly in the input SD file. Note that rDock is not capable of
converting ionised centres back to the neutral form; these are
unidirectional transformations.

**Control of ligand non-polar hydrogens.** By default, rDock uses an implicit non-polar hydrogen model for
receptor and ligand, and all of the scoring function validation has been
performed on this basis. If the `-allH` option is not
defined (recommended), all explicit non-polar hydrogens encountered in
the ligand input SD file are removed, and only the polar hydrogens
(bonded to O, N, or S) are retained. If the `-allH`
option is defined (not recommended), no hydrogens are removed from the
ligand. Note that rDock is not capable of *adding*
explicit non-polar hydrogens, if none exist. In other words, the
`-allH` option disables hydrogen removal, it does
not activate hydrogen addition. You should always make sure that polar
hydrogens are defined explicitly. If the ligand input SD file contains
no explicit non-polar hydrogens, the `-allH` option has
no effect. Receptor protonation is controlled by the system definition
prm file.

---

## Name

rbcavity — Cavity mapping and preparation of docking site (.as) file
file

## Synopsis

`$RBT_ROOT/bin/rbcavity`   
 {-r *`system definition .prm file`*}  
 [
`-ras`
`-was`
`-d`
`-v`
`-s`
]  
 [-l *`distance from cavity`*]  
 [-b *`border`*]

## Usage

**Exploration of cavity mapping parameters.** 

```
rbcavity -r .prm file
```

You
can run `rbcavity` with just the `-r`
argument when first preparing a new receptor for docking. This allows
you to explore rapidly the impact of the cavity mapping parameters on
the generated cavities, whilst avoiding the overhead of actually writing
the docking site (.as) file to disk. The number of cavities and volume
of each cavity are written to standard output.

**Visualisation of cavities.** 

```
rbcavity -r .prm file -d
```

If
you have access to InsightII you can use the
`-d` option to dump the cavity volumes in InsightII
grid file format. There is no need to write the docking site (.as) file
first. The InsightII grid files should be loaded into the reference
coordinate space of the receptor and contoured at a contour level of
0.99.

**Writing the docking site (.as) file.** 

```
rbcavity -r .prm file -was
```

When
you are happy the mapping parameters, use the `-was`
option to write the docking site (.as) file to disk. The docking site
file is a binary file that contains the cavity volumes in a compact
format, and a pre-calculated cuboid grid extending over the cavities.
The grid represents the distance from each point in space to the nearest
cavity grid point, and is used by the cavity penalty scoring function.
Calculating the distance grid can take a long time (whereas the cavity
mapping itself is usually very fast), hence the `-was`
option should be used sparingly.

**Analysis of cavity atoms.** 

```
rbcavity -r .prm file -ras -l distance
```

Use the `-l` options to list the receptor atoms within
a given distance of any of the cavity volumes, for example to determine
which receptor OH/NH3+ groups should be flexible. This option requires
access to the pre-calculated distance grid embedded within the docking
site (.as) file, and is best used in combination with the
`-ras` option, which loads a previously generated
docking site file. This avoids the time consuming step of generating the
cavity distance grid again. If `-l` is used without
`-ras`, the cavity distance grid will be calculated
on-the-fly each time.

**Miscellaneous options.** The `-s` option writes out various statistics on the
cavity and on the receptor atoms in the vicinity of the cavity. These
values have been used in genetic programming model building for docking
pose false positive removal. The `-v` option writes out
the receptor coordinates in PSF/CRD format for use by the rDock Viewer
(not documented here). Note that the PSF/CRD files are not suitable for
simulation purposes, only for visualisation, as the atom types are not
set correctly. The `-b` option controls the size of the
cavity distance grid, and represents the border beyond the actual cavity
volumes. It should not be necessary to vary this parameter (default =
8A) unless longer-range scoring functions are implemented.

---

## Name

— Calculation of vdW grid files (usually called by
`make_grid.csh` wrapper script)

## Synopsis

`$RBT_ROOT/bin/rbcalcgrid`   
 {-r*`system definition file`*}  
 {-o*`output suffix for generated
grids`*}  
 {-p*`vdW scoring function prm file`*}  
 [-g*`grid step`*]  
 [-b*`border`*]

Note that, unlike `rbdock` and
`rbcavity`, spaces are not tolerated between the
command-line options and their corresponding arguments.

## Usage

See `$RBT_ROOT/bin/make_grid.csh` for common
usage.

---

## Name

rbrms — Calculation of ligand Root Mean Squared Displacement (RMSD) between
reference and docked poses

## Synopsis

`$RBT_ROOT/bin/rbrms` {
*`reference SD file`*
} {
*`input SD file`*
} [
*`output SD file`*
] [
*`RMSD threshold`*
]

## Usage

**With two arguments.** `rbrms` calculates the RMSD between each record in
the input SD file and the first record of the reference SD file. If
there is a mismatch in the number of atoms, the record is skipped and
the RMSD is not calculated. The RMSD is calculated over the heavy
(non-hydrogen) atoms only. Results are output to standard output.

**With three arguments.** As for two arguments, plus each record in the input SD file is written
to the output SD file with the calculated RMSD value stored as an SD
data field (`RMSD` field).

**With four arguments.** As for three arguments, with an RMSD filter applied to the output SD
file. Records are not written if they have an RMSD less than the
threshold value with any previous record in the input SD file. This
provides a crude way of removing duplicate poses.

---

## Name

rbtether — Prepares a ligand SD file for tethered scaffold docking. Requires
Daylight SMARTS license. Annotates ligand SD file with tethered substructure
atom indices.

## Synopsis

`$RBT_ROOT/bin/rbtether`   
 {-r*`reference ligand SD file`*}  
 {-i*`input ligand SD file`*}  
 {-s*`SMARTS query`*}  
 {-o*`output root`*}  
 [
`-ap`
`-an`
`-allH`
]

Note that, unlike `rbdock` and
`rbcavity`, spaces are not tolerated between the
command-line options and their corresponding arguments.

## Usage

**Standard options.** 

```
rbtether -rref -iinput -ooutput -squery
```

`rbtether` performs the following actions:

- Runs the SMARTS query against the reference SD file to
  determine the tethered substructure atom indices and
  coordinates.
- If more than one substructure match is retrieved (e.g.
  due to topological symmetry, or if the query is too simple)
  only the first substructure match is retained as the
  reference
- Runs the SMARTS query against each record of the input
  ligand SD file in turn.
- For each substructure match, the ligand coordinates are
  transformed such that the principal axes of the matching
  substructure coordinates are aligned with the reference
  substructure coordinates.
- In addition, an SD data field is added to the ligand
  record which lists the atom indicies of the substructure
  match, for later retrieval by
  `rbdock`.
- Each transformed ligand is written to the output SD
  file.
- Note that if the SMARTS query returns more than one
  substructure match for a ligand, that ligand is written
  multiple times to the output file, once for each match, each
  of which will be docked independently with different
  tethering information

**Determination of reference SMILES.** 

```
rbtether -rref
```

If
`rbtether` is run with only the
`-r` option (with no SMARTS query or ligand input or
output files) the SMILES string for the entire reference ligand is
reported to standard output and the program terminates with no further
action. The SMILES string can often be used as the SMARTS query if the
aim is to tether each ligand to the entire reference ligand
substructure.

**Miscellaneous options.** `-ap -an -allH` have the same meaning as described
for `rbdock`
