# Supplementary material for: rDock: A Fast, Versatile and Open Source Program for Docking Ligands to Proteins and Nucleic Acids
Source: PLoS Comput Biol. 2014 Apr 10;10(4):e1003571. doi: 10.1371/journal.pcbi.1003571 (PMC3983074; doi:10.1371/journal.pcbi.1003571)
Supplement: Software S1 — Compressed file with the source code of the rDock software for ligand docking to Proteins and Nucleic Acids. (GZ) [file pcbi.1003571.s010.gz › rDock_2013.1_src/docs/rDock_reference_guide.pdf]

rDock Reference Guide

Dr. David Morley

rDock Reference Guide

Dr. David Morley

Copyright © 2006 Vernalis

Table of Contents

Preface ... vii
Acknowledgements ... viii
1. Introduction ... 1
2. Configuration ... 2
3. Molecular files and atom typing ... 3
4. Cavity mapping ... 5
5. Common use cases ... 9
    Standard docking ... 9
    Tethered scaffold docking ... 10
    Docking with pharmacophore restraints ... 10
    Docking with explicit waters ... 11
6. Scoring function reference ... 12
7. Docking protocol reference ... 14
8. System definition file reference ... 18
    Receptor definition ... 18
    Ligand definition ... 19
    Solvent definition ... 20
    Cavity mapping ... 21
    Cavity restraint ... 23
    Pharmacophore restraints ... 23
    NMR restraints ... 24
    Example system definition files ... 24
9. rDock file formats ... 26
    .prm file format ... 26
    Water PDB file format ... 27
    Pharmacophore restraints file format ... 28
10. rDock programs ... 30
    Executable (binary) programs ... 30
    Scripts ... 30
    Command-line reference ... 31

List of Tables

6.1. Scoring function terms and C++ implementation classes 12
6.2. Scoring function data files 12
7.1. Chromsome elements 14
7.2. Search algorithm components and C++ implementation classes 14
7.3. Docking protocol data files 14
8.1. Receptor definition parameters 18
8.2. Ligand definition parameters 19
8.3. Solvent definition parameters 20
8.4. Two sphere site mapping parameters 22
8.5. Reference ligand site mapping parameters 22
8.6. Pharmacophore restraint parameters 23
9.1. Conversion of temperature factor values to solvent flexibility modes 27
9.2. Pharmacophore restraint types 28
10.1. Core rDock C++ executables 30
10.2. Auxiliary rDock C++ executables 30
10.3. Core scripts 30
10.4. Auxiliary scripts 31

List of Examples

5.1. Example ligand definition for tethered scaffold 10
7.1. Standard rDock docking protocol (`dock.prm`) 15
8.1. Full system definition file with all sections and common parameters enumerated explicitly 25
9.1. Example .prm file 27
9.2. Valid rDock PDB file for explicit, flexible waters 28

Preface

It is intended to develop this document into a full reference guide for the rDock platform, describing the software tools, parameter files, scoring functions, and search engines. The reader is encouraged to cross-reference the descriptions with the corresponding source code files to discover the finer implementation details.

Acknowledgements

Third-party source code. Two third-party C++ libraries are included within the rDock source code, to provide support for specific numerical calculations. The source code for each library can be distributed freely without licensing restrictions and we are grateful to the respective authors for their contributions.

- Nelder-Meads Simplex search, from Prof. Virginia Torczon's group, College of William and Mary, Department of Computer Science, VA. (http://www.cs.wm.edu/~va/software/)
- Template Numerical Toolkit, from Roldan Pozo, Mathematical and Computational Sciences Division, National Institute of Standards and Technology (http://math.nist.gov/tnt/)

Chapter 1. Introduction

The rDock platform is a suite of command-line tools for high-throughput docking and virtual screening. The programs and methods were developed and validated over a number of years at RiboTargets (more recently, Vernalis) for proprietary use. The original program (RiboDock) was designed for high-throughput virtual screening of large ligand libraries against RNA targets, in particular the bacterial ribosome. Since 2002 the programs have been substantially rewritten and validated for docking of drug-like ligands to protein and RNA targets. A variety of experimental restraints can be incorporated into the docking calculation, in support of an integrated Structure-Based Drug Design process.

Chapter 2. Configuration

Before launching rDock, make sure the following environment variable are defined. Precise details are likely to be site-specific.

RBT_ROOT environment variable. `$RBT_ROOT` environment variable should be defined to point to the rDock installation directory.

RBT_HOME environment variable. `$RBT_HOME` environment variable is optional, but can defined to to point to a user project directory containing rDock input files and customised data files.

PATH environment variable. `$RBT_ROOT/bin` should be added to the `$PATH` environment variable.

LD_LIBRARY_PATH. `$RBT_ROOT/lib` should be added to the `$LD_LIBRARY_PATH` environment variable.

Input file locations. The search path for the majority of input files for rDock is:

- Current working directory
- `$RBT_HOME`, if defined
- The appropriate subdirectory of `$RBT_ROOT/data/`. For example, the default location for scoring function files is `$RBT_ROOT/data/sf/`.

The exception is that input ligand SD files are always specified as an absolute path. If you wish to customise a scoring function or docking protocol, it is sufficient to copy the relevant file to the current working directory or to `$RBT_HOME`, and to modify the copied file.

Launching rDock. For small scale experimentation, the rDock executables can be launched directly from the command line. However, serious virtual screening campaigns will likely need access to a compute farm. In common with other docking tools, rDock uses the embarrassingly parallel approach to distributed computing. Large ligand libraries are split into smaller chunks, each of which is docked independently on a single machine. Docking jobs are controlled by a distributed resource manager (DRM) such as Condor. The RBT_TEST suite provides some examples of the generation of Condor command files for rDock.

Chapter 3. Molecular files and atom typing

Macromolecular targets (protein or RNA) are input from Tripos MOL2 files (`RbtMOL2FileSource`) or from pairs of Charmm PSF (`RbtPsfFileSource`) and CRD (`RbtCrdFileSource`) files. Ligands are input from MDL SD files (`RbtMdlFileSource`). Explicit structural waters are input optionally from PDB files (`RbtPdbFileSource`). Ligand docking poses are output to MDL SD files.

The rDock scoring functions have been defined and validated for implicit non-polar hydrogen (extended carbon) models only. If you provide all-atom models, be aware that the non-polar hydrogens will be removed automatically. Polar hydrogens must be defined explicitly in the molecular files, and are not added by rDock. Positive ionisable and negative ionisable groups can be automatically protonated and deprotonated respectively to create common charged groups such as guanidinium and carboxylic acid groups.

Tip

MOL2 is now the preferred file format for rDock as it eliminates many of the atom typing issues inherent in preparing and loading PSF files. The use of PSF/CRD files is strongly discouraged. The recommendation is to prepare an all-atom MOL2 file with correct Tripos atom types assigned, and allow rDock to remove non-polar hydrogens on-the-fly.

Atomic properties. rDock requires the following properties to be defined per atom. Depending on the file format, these properties may be loaded directly from the molecular input file, or derived internally once the model is loaded.

- Cartesian (x,y,z) coordinates
- Element (atomic number)
- Formal hybridisation state (sp, sp2, sp3, aromatic, trigonal planar)
- Formal charge
- Distributed formal charge (known informally as *group* charge)
- Tripos force field type (rDock uses a modified version of the Sybyl 5.2 types, extended to include carbon types with implicit non-polar hydrogens)
- Atom name
- Substructure (residue) name
- Atomic radius (assigned per element from `$RBT_ROOT/data/RbtElements.dat`)

Note

The rDock scoring functions do not use partial charges and therefore partial charges do not have to be defined. The atomic radii are simplified radii defined per element, and are used for cavity mapping and in the polar scoring function term, but are *not* used in the vdW scoring function term. The latter has its own indepedent parameterisation based on the Tripos force field types.

Difference between formal charge and distributed formal charge. The formal charge on an atom is always an integer. For example, a charged carboxylic acid group (`COO-`) can be defined formally as a formal double bond to a neutral oxygen sp2, and a formal single bond to a formally charged oxygen sp3. In reality of course, both oxygens are equivalent. rDock distributes the integer formal

charge across all equivalent atoms in the charged group that are topologically equivalent. In negatively charged acid groups, the formal charge is distributed equally between the acid oxygens. In positively charged amines, the formal charge is distributed equally between the hydrogens. In charged guanidinium, amidinium, and imidazole groups, the central carbon also receives an equal portion of the formal charge (in addition to the hydrogens). The distributed formal charge is also known as the group charge. The polar scoring functions in rDock use the distributed formal charge to scale the polar interaction strength of the polar interactions.

Parsing a MOL2 file. `MOLECULE`, `ATOM`, `BOND` and `SUBSTRUCTURE` records are parsed. The atom name, substructure name, Cartesian coordinates and Tripos atom type are read directly for each atom. The element type (atomic number) and formal hybridisation state are derived from the Tripos type using an internal lookup table. Formal charges are *not* read from the MOL2 file and do not have to be assigned correctly in the file. Distributed formal charges are assigned directly by rDock based on standard substructure and atom names as described below.

Parsing an SD file. Cartesian coordinates, element and formal charge are read directly for each atom. Formal bond orders are read for each bond. Atom names are derived from element name and atom ID (e.g. `C1`, `N2`, `C3` etc). The substructure name is `MOL`. Formal hybridisation states are derived internally for each atom based on connectivity patterns and formal bond orders. The Tripos types are asssigned using internal rules based on atomic number, formal hybridisation state and formal charges. The integer formal charges are distributed automatically across all topologically equivalent atoms in the charged group.

Assigning distributed formal charges to the receptor. rDock provides a file format independent method for assigning distributed formal charges directly to the receptor atoms, which is used by the MOL2 and PSF/CRD file readers. The method uses a lookup table based on standard substructure and atom names, and does not require the integer formal charges to be assigned to operate correctly. The lookup table file is `$RBT_ROOT/data/sf/RbtIonicAtoms.prm`. Each section name represents a substructure name that contains formally charged atoms. The entries within the section represent the atom names and distributed formal charges for that substructure name. The file provided with rDock contains entries for all standard amino acids and nucleic acids, common metals, and specific entries required for processing the GOLD CCDC/Astex validation sets.

Important

You may have to extend `RbtIonicAtoms.prm` if you are working with non-standard receptor substructure names and/or atom names, in order for the distributed formal charges to be assigned correctly.

Chapter 4. Cavity mapping

Virtual screening is very rarely conducted against entire macromolecules. The usual practice is to dock small molecules in a much more confined region of interest. rDock makes a clear distinction between the region the ligand is allowed to explore (known here as the docking site), and the receptor atoms that need to be included in order to calculate the score correctly. The former is controlled by the cavity mapping algorithm, whilst the latter is scoring function dependent as it depends on the distance range of each component term (for example, vdW range >> polar range). For this reason, it is usual practice with rDock to prepare intact receptor files (rather than truncated spheres around the region of interest), and to allow each scoring function term to isolate the relevant receptor atoms within range of the docking site.

Dock provides two methods for defining the docking site:

- Two sphere method
- Reference ligand method

Two sphere method. The two sphere method aims to find cavities that are accessible to a small sphere (of typical atomic or solvent radius) but are inaccessible to a larger sphere. The larger sphere probe will eliminate flat and convex regions of the receptor surface, and also shallow cavities. The regions that remain and are accessible to the small sphere are likely to be the “nice well defined” cavities of interest for drug design purposes.

Procedure 4.1. Two sphere mapping algorithm

1. A grid is placed over the cavity mapping region, encompassing a sphere of radius=RADIUS, center=CENTER. Cavity mapping is restricted to this sphere. All cavities located will be wholly within this sphere. Any cavity that would otherwise protrude beyond the cavity mapping sphere will be truncated at the periphery of the sphere.

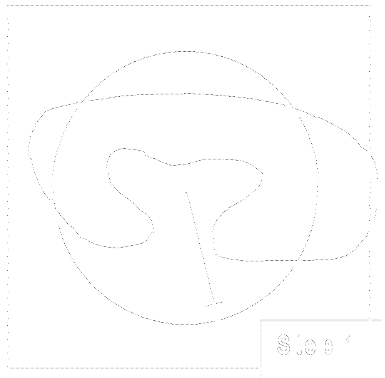

2. Grid points within the volume occupied by the receptor are excluded (coloured red). The radii of the receptor atoms are increased temporarily by VOL_INCR in this step.

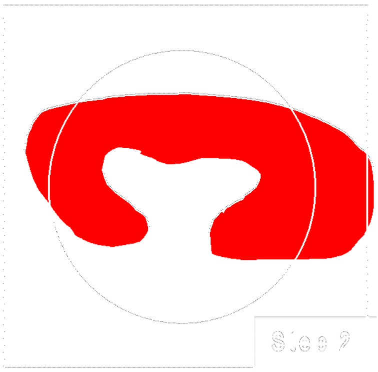

3. Probes of radii LARGE_SPHERE are placed on each remaining unallocated grid point and checked for clashes with receptor excluded volume. To eliminate edge effects, the grid is extended beyond the cavity mapping region by the diameter of the large sphere (for this step only). This allows the large probe to be placed on grid points outside of the cavity mapping region, yet partially protrude into the cavity mapping region.

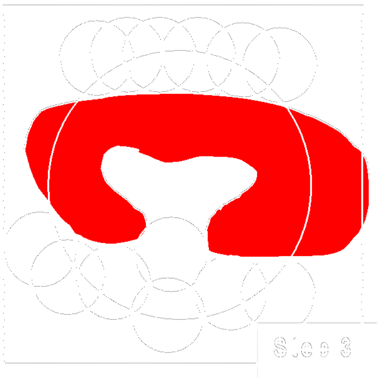

4. All grid points within the cavity mapping region that are accessible to the large probe are excluded (coloured green).

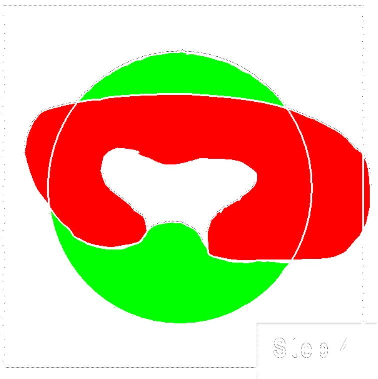

5. Probes of radii SMALL_SPHERE are placed on each remaining grid point and checked for clashes with receptor excluded volume (red) or large probe excluded volume (green)

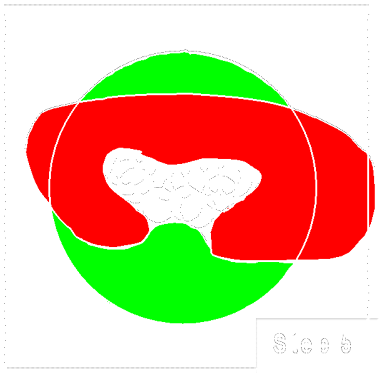

6. All grid points that are accessible to the small probe are selected (yellow).

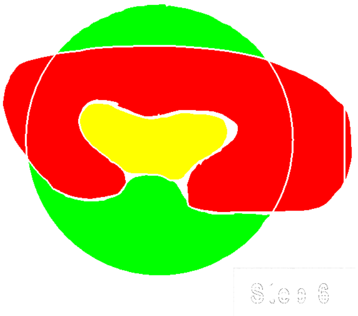

7. The final selection of cavity grid points is divided into distinct cavities (contiguous regions). In this example only one distinct cavity is found. User-defined filters of MIN_VOLUME and MAX_CAVITIES are applied at this stage to select a subset of cavities if required. Note that the filters will accept or reject intact cavities only.

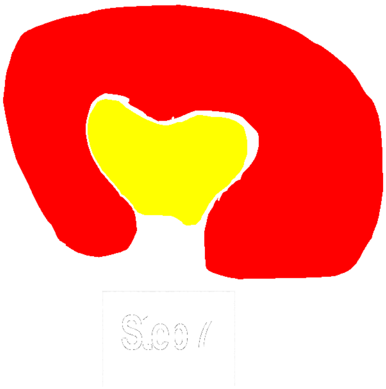

Reference ligand method. The reference ligand method provides a much easier option to define a docking volume of a given size around the binding mode of a known ligand, and is particularly appropriate for large scale automated validation experiments.

1. Reference coordinates are read from REF_MOL. A grid is placed over the cavity mapping region, encompassing overlapping spheres of radius=RADIUS, centered on each atom in REF_MOL. Grid points outside of the overlapping spheres are excluded (coloured green). Grid points within the volume occupied by the receptor are excluded (coloured red). The vdW radii of the receptor atoms are increased by VOL_INCR in this step.

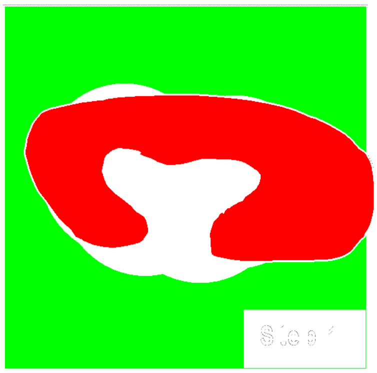

2. Probes of radii SMALL_SPHERE are placed on each remaining grid point and checked for clashes with red or green regions.

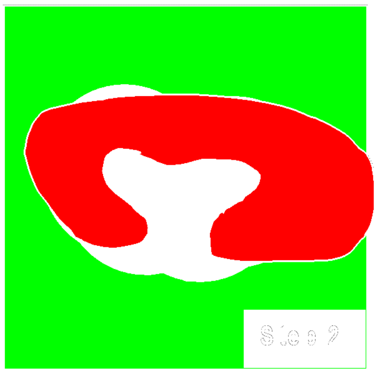

3. All grid points that are accessible to the small probe are selected (yellow).

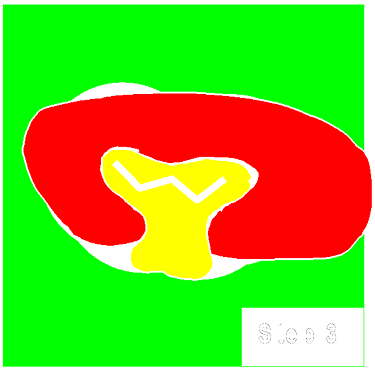

4. The final selection of cavity grid points is divided into distinct cavities (contiguous regions). In this example only one distinct cavity is found. User-defined filters of MIN_VOLUME and MAX_CAVITIES are applied at this stage to select a subset of cavities if required. Note that the filters will accept or reject intact cavities only.

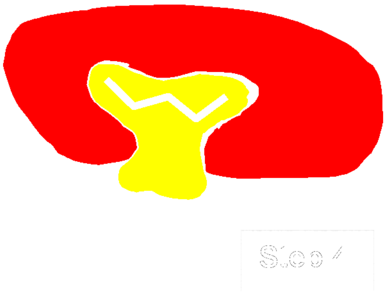

Chapter 5. Common use cases

This section does not pretend to be a comprehensive User Guide. It does, however, highlight the key steps the user must take for different docking strategies, and may serve as a useful checklist in writing such a guide in the future.

Standard docking

By standard docking, we refer to docking of a flexible, untethered ligand to a receptor in the absence of explicit structural waters or any experimental restraints.

Procedure 5.1. Standard docking workflow

1. Prepare a MOL2 file for the protein or nucleic acid target, taking into account the atom typing issues described above for MOL2 file parsing. The recommendation is to prepare an all-atom MOL2 file and allow rDock to remove the non-polar hydrogens on-the-fly.

   ## Important

   Make sure that any non-standard atom names and substructure names are defined in `$RBT_ROOT/data/sf/RbtIonicAtoms.prm` in order for the assignment of distributed formal charges to work correctly.

   Make sure that the Tripos atom types are set correctly. rDock uses the Tripos types to derive other critical atomic properties such as atomic number and hybridisation state.

   ## Note

   The rDock MOL2 parser was developed to read the CCDC/Astex `protein.mol2` files, therefore this validation set is the *de facto* standard reference. You should compare against the format of the CCDC/Astex MOL2 files if you are in doubt as to whether a particular MOL2 file is suitable for rDock

2. Prepare a system definition file. At a minimum, you need to define the receptor parameters, the cavity mapping parameters (`SECTION MAPPER`) and the cavity restraint penalty (`SECTION CAVITY`). Make sure you define the `RECEPTOR_FLEX` parameter if you wish to activate sampling of terminal OH and NH3+ groups in the vicinity of the docking site.

3. Generate the docking site (.as) file using `rbcavity`. You will require a reference bound ligand structure in the coordinate space of the receptor if you wish to use the reference ligand cavity mapping method.

4. Prepare the ligand SD files you wish to dock, taking into account the atom typing issues described above for SD file parsing. In particular, make sure that formal charges and formal bond order are defined coherently so that there are no formal valence errors in the file. rDock will report any perceived valence errors but will dock the structures anyway. Note that rDock never samples bond lengths, bond angles, ring conformations, or non-rotatable bonds during docking so initial conformations should be reasonable.

5. Run a small test calculation to check that the system is defined correctly. For example, run `rbdock` from the command line with a small ligand SD file, with the score-only protocol (`-p score.prm`) and with the `-T 2` option to generate verbose output. The output will include receptor atom properties, ligand atom properties, flexibility parameters, scoring function parameters and docking protocol parameters.

6. When satisfied, launch the full-scale calculations. A description of the various means of launching rDock is beyond the scope of this guide.

Tethered scaffold docking

In tethered scaffold docking, the ligand poses are restricted and forced to overlay the substructure coordinates of a reference ligand. The procedure is largely as for standard docking, except that:

- Ligand SD files must be prepared with the `rbtether` utility to annotate each record with the matching substructure atom indices, and to transform the coordinates of each ligand so that the matching substructure coordinates are overlaid with the reference substructure coordinates. This requires a Daylight SMARTS toolkit license.
- The system definition file should contain a `SECTION LIGAND` to define which of the the ligand degrees of freedom should be tethering to their reference values. Tethering can be applied to position, orientation and dihedral degrees of freedom independently. Note that the tethers are applied directly within the chromosome representation used by the search engine (where they affect the randomisation and mutation operators), and therefore external restraint penalty functions to enforce the tethers are not required.

Important

The reference state values for each tethered degree of freedom are defined directly from the initial conformation of each ligand as read from the input SD file, and *not* from the reference SD file used by `rbtether`. This is why the ligand coordinates are transformed by `rbtether`, such that each ligand record can act as its own reference state. The reference SD file used by `rbtether` is not referred to by the docking calculation itself.

It follows from the above that tethered ligand docking is inappropriate for input ligand SD files that have not already been transformed to the coordinate space of the docking site, either by `rbtether` or by some other means.

Example 5.1. Example ligand definition for tethered scaffold

This definition will tether the position and orientation of the tethered substructure, but will allow free sampling of ligand dihedrals.

```
 SECTION LIGAND
    TRANS_MODE TETHERED
    ROT_MODE TETHERED
    DIHEDRAL_MODE FREE
    MAX_TRANS 1.0
    MAX_ROT 30.0
END_SECTION
```

Docking with pharmacophore restraints

In pharmacophore restrained docking, ligand poses are biased to fit user-defined pharmacophore points. The bias is introduced through the use of an external penalty restraint, which penalises docking poses that do not match the pharmacophore restraints. Unlike tethered scaffold docking, there is no modification to the chromosome operators themselves, hence the search can be inefficient, particularly for large numbers of restraints and/or for ligands with large numbers of matching features. Pre-screening of ligands is based purely on feature counts, and not on geometric match considerations.

The implementation supports both mandatory and optional pharmacophore restraints. The penalty function is calculated over all mandatory restraints, and over (any `NOPT` from `N`) of the optional restraints. For example, you may wish to ensure that any 4 from 7 optional restraints are satisfied in the generated poses.

The procedure is largely as for standard docking, except that:

- You should prepare separate pharmacophore restraint files for the mandatory and optional restraints. Note that optional restraints do not have to be defined, it is sufficient to only define at least one mandatory restraint.
- The system definition file should contain a `SECTION PHARMA` to add the pharmacophore restraint penalty to the scoring function.

Docking with explicit waters

Explicit structural waters can be loaded from an external PDB file, independently from the main receptor model, by adding a `SECTION SOLVENT` to the system definition file. The user has fine control over the flexibility of each water molecule. A total of 9 flexibility modes are possible, in which the translational and rotational degrees of freedom of each water can be set independently to `FIXED`, `TETHERED`, or `FREE`. Thus, for example, it is possible to define a water with a fixed oxygen coordinate (presumably at a crystallographically observed position), but freely rotating such that the orientation of the water hydrogens can be optimised by the search engine (and can be ligand-dependent).

Note

In the current implementation, *solvent* refers strictly to *water* molecules, and the format of the water PDB file is very strictly defined. In future implementations it is anticipated that other, larger (and possibly flexible) molecules will be loadable as solvent, and that other file formats will be supported.

Procedure 5.2. Explicit waters workflow

1. Prepare a separate PDB file for the explicit waters according to the format prescribed (the section called “Water PDB file format”)
2. Add a `SECTION SOLVENT` to the system definition file and define the relevant flexibility parameters (Table 8.3, “Solvent definition parameters”). The minimal requirement is to define the `FILE` parameter.
3. Decide whether you wish to have different per-solvent flexibility modes (defined via the occupancy values and temperature factor values in the PDB file (Table 9.1, “Conversion of temperature factor values to solvent flexibility modes”)), or whether you wish to have a single flexibility mode applied to all waters (defined via the `TRANS_MODE` and `ROT_MODE` values in the `SECTION SOLVENT` of the receptor .prm file)

Important

If you wish to use per-solvent flexibility modes (that is, you wish to set different modes for different waters) make sure that you do not define `TRANS_MODE` or `ROT_MODE` entries in the `SECTION SOLVENT` as these values will override the per-solvent values derived from the temperature factors in the PDB file.

4. If you have defined any waters with `TETHERED` translational or rotational degrees of freedom, define `MAX_TRANS` and/or `MAX_ROT` values as appropriate (or accept the default values. The tethered ranges are applied to all tethered waters and can not be defined on a per-solvent basis at present.

Chapter 6. Scoring function reference

Scoring functions for docking are constructed at run-time (by class `RbtSFFactory`) from scoring function definition files (rDock .prm format). The default location for scoring function definition files is `$RBT_ROOT/data/sf/`.

The total score is an aggregate of intermolecular ligand-receptor and ligand-solvent interactions (branch `SCORE.INTER`), intra-ligand interactions (branch `SCORE.INTRA`), intra-receptor, intra-solvent and receptor-solvent interactions (branch `SCORE.SYSTEM`), and external restraint penalties (branch `SCORE.RESTR`).

The `SCORE.INTER`, `SCORE.INTRA` and `SCORE.SYSTEM` branches consist of weighted sums of interaction terms as shown below. Note that not all terms appear in all branches. See the rDock draft paper for more details on the implementation of these terms.

Table 6.1. Scoring function terms and C++ implementation classes

Term	Description	INTER	INTRA	SYSTEM
VDW	van der Waals	`RbtVdwIdxSF`	`RbtVdwIntraSF`	`RbtVdwIdxSF`
VDW	van der Waals (grid-based)	`RbtVdwGridSF`	N/A	N/A
POLAR	Attractive polar	`RbtPolarIdxSF`	`RbtPolarIntraSF`	`RbtPolarIdxSF`
REPUL	Repulsive polar	`RbtPolarIdxSF`	`RbtPolarIntraSF`	`RbtPolarIdxSF`
SOLV	Desolvation	`RbtSAIdxSF`	`RbtSAIdxSF`	`RbtSAIdxSF`
DIHEDRAL	Dihedral potential	N/A	`RbtDihedralIntraSF`	`RbtDihedralTargetSF`
CONST	Translation/rotational binding entropy penalty	`RbtConstSF`	N/A	`RbtConstSF`
ROT	Torsional binding entropy penalty	`RbtRotSF`	N/A	N/A

Two intermolecular scoring functions (`SCORE.INTER` branch) have been validated. These are known informally as the standard scoring function and the desolvation scoring function (referred to as SF3 and SF5 respectively in the rDock draft paper). The standard intermolecular scoring function consists of `VDW`, `POLAR` and `REPUL` terms. In the desolvation scoring function, the `REPUL` term is replaced by a more finely parameterised desolvation potential (`SOLV` term) based on a Weighted Solvent-Accessible Surface Area (WSAS) model. The ligand intramolecular scoring function (`SCORE.INTRA` branch) remains constant in both cases, and has the same terms and weights as the standard intermolecular scoring function.

Table 6.2. Scoring function data files

File	Description
RbtInterIdxSF.prm	Intermolecular scoring function definition (standard scoring function, SF3)
RbtInterGridSF.prm	As above, but vdW term uses a precalculated grid
RbtSolvIdxSF.prm	Intermolecular scoring function definition (desolvation scoring function, SF5)

File	Description
RbtSolvGridSF.prm	As above, but vdW term uses a precalculated grid
RbtIntraSF.prm	Ligand intramolecular scoring function definition
RbtTargetSF.prm	System scoring function definition (just contains SCORE.SYSTEM.DIHEDRAL, see Notes below)
calcgrid_vdw1.prm	vdW term only (`ECUT=1`), for calculating vdW grid (used by `rbcalcgrid`)
calcgrid_vdw5.prm	vdW term only (`ECUT=5`), for calculating vdW grid (used by `rbcalcgrid`)
Tripos52_vdw.prm	vdW term parameter file
Tripos52_dihedrals.prm	Dihedral term parameter file
solvation_asp.prm	Desolvation term parameter file

Note

External restraint penalty terms are defined by the user in the system definition .prm file.

Originally, rDock did not support flexible receptor dihedrals or explicit structural waters, and the overall scoring function consisted of just the `SCORE.INTER` and `SCORE.INTRA` branches. At that time, the intermolecular scoring function definition file (e.g. `RbtInterIdxSF.prm`) represented precisely the SCORE.INTER terms, and the intramolecular definition file (`RbtIntraSF.prm`) represented precisely the SCORE.INTRA terms. With the introduction of receptor flexibility and explicit structural waters (and hence the need for the `SCORE.SYSTEM` branch), the situation is slightly more complex. For implementation reasons, many of the terms reported under `SCORE.SYSTEM` (with the exception of the dihedral term) are calculated simultaneously with the terms reported under `SCORE.INTER`, and hence their parameterisation is defined implicitly in the intermolecular scoring function definition file. In contrast, the ligand intramolecular scoring function terms can be controlled independently.

Chapter 7. Docking protocol reference

Docking protocols are constructed at run-time (by class RbtTransformFactory) from docking protocol definition files (rDock .prm format). The default location for docking protocol files is $RBT_ROOT/data/scripts/.

The docking protocol definition file defines the sequence of search algorithms that constitute a single docking run for a single ligand record. Each search algorithm component operates either on a single chromosome representing the system degrees of freedom, or on a population of such chromosomes.

The chromosome is constructed (by RbtChromFactory) as an aggregate of individual chromosome elements for the receptor, ligand and explicit solvent degrees of freedom, as defined by the flexibility parameters in the system definition file.

Table 7.1. Chromsome elements

Element	Defined by	Class	Length
Position	Centre of mass	RbtChromPositionElement	3
Orientation	Euler angles for principal axes	RbtChromPositionElement	3
Dihedral	Dihedral angle for rotatable bond	RbtChromDihedralElement	1 per bond
Occupancy	Explicit water occupancy state	RbtChromOccupancylElement	1 per water

Table 7.2. Search algorithm components and C++ implementation classes

Component	Class	Operates on
Randomise population	RbtRandPopTransform	Chromosome population
Genetic algorithm search	RbtGATransform	Chromosome population
Monte Carlo simulated annealing	RbtSimAnnTransform	Single chromosome
Simplex minimisation	RbtSimplexTransform	Single chromosome
Null operation	RbtNullTransform	N/A

Table 7.3. Docking protocol data files

File	Description
score.prm	Calculates score only for initial conformation (standard scoring function)
score_solv.prm	As above, but uses desolvation scoring function
minimise.prm	Simplex minimisation of initial conformation (standard scoring function)
minimise_solv.prm	As above, but uses desolvation scoring function
dock.prm	Full docking search (standard scoring function)

File	Description
dock_solv.prm	As above, but uses desolvation scoring function
dock_grid.prm	Full docking search (standard scoring function, grid-based vdW term)
dock_solv_grid.prm	Full docking search (desolvation scoring function, grid-based vdW term)

The standard rDock docking protocol consists of three phases of a Genetic Algorithm search, followed by low-temperature Monte Carlo and Simplex minimisation. By way of example, the `dock.prm` script is documented in detail. The other scripts are very similar.

Example 7.1. Standard rDock docking protocol (`dock.prm`)

```
SECTION SCORE
    INTER    RbtInterIdxSF.prm
    INTRA    RbtIntraSF.prm
    SYSTEM   RbtTargetSF.prm
END_SECTION
```

The scoring function definition is referenced within the docking protocol definition file itself, in the `SCORE` section. This section contains entries for the `INTER`, `INTRA` and `SYSTEM` scoring function definition files.

```
SECTION SETSLOPE_1
    TRANSFORM                      RbtNullTransform
    WEIGHT@SCORE.RESTR.CAVITY      5.0     # Dock with a high penalty for leav
    WEIGHT@SCORE.INTRA.DIHEDRAL    0.1     # Gradually ramp up dihedral weight
    ECUT@SCORE.INTER.VDW           1.0     # Gradually ramp up energy cutoff fo
    USE_4_8@SCORE.INTER.VDW        TRUE    # Start docking with a 4-8 vdW poten
    DA1MAX@SCORE.INTER.POLAR       180.0   # Broader angular dependence
    DA2MAX@SCORE.INTER.POLAR       180.0   # Broader angular dependence
    DR12MAX@SCORE.INTER.POLAR      1.5     # Broader distance range
END_SECTION
```

All sections that contain the `TRANSFORM` parameter are interpreted as a search algorithm component. The value of the `TRANSFORM` parameter is the C++ implementation class name for that transform. An `RbtNullTransform` can be used to send messages to the scoring function to modify key scoring function parameters in order to increase search efficiency. All parameter names that contain the `@` symbol are interpreted as scoring function messages, where the string before the `@` is the scoring function parameter name, the string after the `@` is the scoring function term, and the parameter value is the new value for the scoring function parameter. Messages are sent blind, with no success feedback, as the the docking protocol has no knowledge of the composition of the scoring function terms.

Here, we start the docking with a soft 4-8 vdW potential, a reduced dihedral potential, and extended polar ranges (distances and angles) for the intermolecular polar potential. These changes are all designed to aid sampling efficiency by not overpenalising bad contacts in the initial, randomised population, and by encouraging the formation of intermolecular hydrogen bonds.

```
SECTION RANDOM_POP
    TRANSFORM                      RbtRandPopTransform
    POP_SIZE                       50
    SCALE_CHROM_LENGTH             TRUE
END_SECTION
```

Creates an initial, randomised chromosome population. If `SCALE_CHROM_LENGTH` is false, the

population is of fixed size, defined by POP_SIZE. If SCALE_CHROM_LENGTH is true, the population is proportional to the overall chromosome length, defined by POP_SIZE multiplied by the chromosome length.

```
SECTION GA_SLOPE1
    TRANSFORM                      RbtGATransform
    PCROSSOVER                     0.4     # Prob. of crossover
    XOVERMUT                       TRUE    # Cauchy mutation after each crosso
    CMUTATE                        FALSE   # True = Cauchy; False = Rectang. f
    STEP_SIZE                      1.0     # Max relative mutation
END_SECTION
```

First round of GA.

```
SECTION SETSLOPE_3
    TRANSFORM                      RbtNullTransform
    WEIGHT@SCORE.INTRA.DIHEDRAL    0.2
    ECUT@SCORE.INTER.VDW           5.0
    DA1MAX@SCORE.INTER.POLAR       140.0
    DA2MAX@SCORE.INTER.POLAR       140.0
    DR12MAX@SCORE.INTER.POLAR      1.2
END_SECTION
```

Increases the ligand dihedral weight, increases the short-range intermolecular vdW hardness (ECUT), and decreases the range of the intermolecular polar distances and angles.

```
SECTION GA_SLOPE3
    TRANSFORM                      RbtGATransform
    PCROSSOVER                     0.4     # Prob. of crossover
    XOVERMUT                       TRUE    # Cauchy mutation after each crosso
    CMUTATE                        FALSE   # True = Cauchy; False = Rectang. f
    STEP_SIZE                      1.0     # Max relative mutation
END_SECTION
```

Second round of GA with revised scoring function parameters.

```
SECTION SETSLOPE_5
    TRANSFORM                      RbtNullTransform
    WEIGHT@SCORE.INTRA.DIHEDRAL    0.3
    ECUT@SCORE.INTER.VDW           25.0
    USE_4_8@SCORE.INTER.VDW        FALSE   # Now switch to a convential 6-12 f
    DA1MAX@SCORE.INTER.POLAR       120.0
    DA2MAX@SCORE.INTER.POLAR       120.0
    DR12MAX@SCORE.INTER.POLAR      0.9
END_SECTION
```

Further increases the ligand dihedral weight, further increases the short-range intermolecular vdW hardness (ECUT), and further decreases the range of the intermolecular polar distances and angles. Also switches from softer 4-8 vdW potential to a harder 6-12 potential for final round of GA, MC and minimisation.

```
SECTION GA_SLOPE5
    TRANSFORM                      RbtGATransform
    PCROSSOVER                     0.4     # Prob. of crossover
    XOVERMUT                       TRUE    # Cauchy mutation after each crosso
    CMUTATE                        FALSE   # True = Cauchy; False = Rectang. f
    STEP_SIZE                      1.0     # Max relative mutation
END_SECTION
```

Final round of GA with revised scoring function parameters.

```
SECTION SETSLOPE_10
    TRANSFORM                       RbtNullTransform
    WEIGHT@SCORE.INTRA.DIHEDRAL     0.5     # Final dihedral weight matches SF f
    ECUT@SCORE.INTER.VDW            120.0   # Final ECUT matches SF file
    DA1MAX@SCORE.INTER.POLAR        80.0
    DA2MAX@SCORE.INTER.POLAR        100.0
    DR12MAX@SCORE.INTER.POLAR       0.6
END_SECTION
```

Resets all the modified scoring function parameters to their final values, corresponding to the values in the scoring function definition files. It is important that the final scoring function optimised by the docking search can be compared directly with the score-only and minimisation-only protocols, in which the scoring function parameters are not modified.

```
SECTION MC_10K
    TRANSFORM                       RbtSimAnnTransform
    START_T                         10.0
    FINAL_T                         10.0
    NUM_BLOCKS                      5
    STEP_SIZE                       0.1
    MIN_ACC_RATE                    0.25
    PARTITION_DIST                  8.0
    PARTITION_FREQ                  50
    HISTORY_FREQ                    0
END_SECTION
```

Low temperature Monte Carlo sampling, starting from fittest chromosome from final round of GA.

```
SECTION SIMPLEX
    TRANSFORM                       RbtSimplexTransform
    MAX_CALLS                       200
    NCYCLES                         20
    STOPPING_STEP_LENGTH            10e-4
    PARTITION_DIST                  8.0
    STEP_SIZE                       1.0
    CONVERGENCE                     0.001
END_SECTION
```

Simplex minimisation, starting from fittest chromosome from low temperature Monte Carlo sampling.

```
SECTION FINAL
    TRANSFORM                       RbtNullTransform
    WEIGHT@SCORE.RESTR.CAVITY       1.0     # revert to standard cavity penalty
END_SECTION
```

Finally, we reset the cavity restraint penalty to 1. The penalty has been held at a value of 5 throughout the search, to strongly discourage the ligand from leaving the docking site.

Chapter 8. System definition file reference

Although known previously as the *receptor* .prm file, the system definition file has evolved to contain much more than the receptor information. The system definition file is used to define:

- Receptor input files and flexibility parameters (the section called "Receptor definition")
- Explicit solvent input file and flexibility parameters (the section called "Solvent definition")
- Ligand flexibility parameters (the section called "Ligand definition").
- External restraint terms to be added to the total scoring function (e.g. cavity restraint, pharmacophoric restraint)

Receptor definition

The receptor can be loaded from a single MOL2 file, or from a combination of Charmm PSF and CRD files. In the former case the MOL2 file provides the topology and reference coordinates simultaneously, whereas in the latter case the topology is loaded from the PSF file and the reference coordinates from the CRD file.

For historical compatibility reasons, receptor definition parameters are all defined in the top-level namespace and should not be placed between `SECTION`..`END_SECTION` pairs.

Caution

If MOL2 and PSF/CRD parameters are defined together, the MOL2 parameters take precedence and are used to load the receptor model.

Table 8.1. Receptor definition parameters

Parameter	Description	Type	Default	Range of values
Parameters specific to loading receptor in MOL2 file format.				
`RECEPTOR_FILE`	Name of receptor MOL2 file	Filename string	No default value	Valid MOL2 filename
Parameters specific to loading receptor in Charmm PSF/CRD file format				
`RECEPTOR_TOPOL_FILE`	Name of receptor Charmm PSF file	Filename string	No default value	Valid Charmm PSF filename
`RECEPTOR_COORD_FILE`	Name of receptor Charmm CRD file	Filename string	No default value	Valid Charmm CRD filename
`RECEPTOR_MASSES_FILE`	Name of rDock-annotated Charmm masses file	Filename string	No default value	`masses.rtf` `top_all2_prot_na.inp`

Parameter	Description	Type	Default	Range of values
General receptor parameters, applicable to either file format				
RECEPTOR_SEGMENT_NAME	List of molecular segment names to read from either MOL2 or PSF/CRD file. If this parameter is defined, then any segment/chains not listed are not loaded. This provides a convenient way to remove cofactors, counterions and solvent without modifying the original file.	Comma-separated list of segment name strings (without any spaces)	Empty (i.e. all segments read from file)	Comma-separated list of segment name strings
RECEPTOR_FLEX	Defines terminal OH and NH3+ groups within this distance of docking volume as flexible.	float (Angstroms)	Undefined (rigid receptor)	> 0.0 (3.0 is a reasonable value)
Advanced parameters (should not need to be changed by the user)				
RECEPTOR_ALL_H	Disables the removal of explicit non-polar hydrogens from the receptor model. *Not recommended*	boolean	FALSE	TRUE or FALSE
DIHEDRAL_STEP	Maximum mutation step size for receptor dihedral degrees of freedom	float (degrees)	30.0	>0.0

Ligand definition

Ligand definition parameters need only be defined if you wish to introduce tethering of some or all of the ligand degrees of freedom. If you are running conventional free docking then this section is not required. All ligand definition parameters should be defined in `SECTION LIGAND`. Note that the ligand input SD file continues to be specified directly on the rbdock command-line and not in the system definition file.

Table 8.2. Ligand definition parameters

Parameter	Description	Type	Default	Range of values
Main user parameters				
TRANS_MODE	Sampling mode for ligand translational degrees of freedom	enumerated string literal	FREE	FIXED<br>TETHERED<br>FREE
ROT_MODE	Sampling mode for ligand whole-body rotational degrees of freedom	enumerated string literal	FREE	FIXED<br>TETHERED<br>FREE
DIHEDRAL_MODE	Sampling mode for ligand internal dihedral degrees of freedom	enumerated string literal	FREE	FIXED<br>TETHERED<br>FREE
MAX_TRANS	(for TRANS_MODE = TETHERED only) Maximum deviation allowed from reference centre of mass	float (Angstroms)	1.0	>0.0

Parameter	Description	Type	Default	Range of values
MAX_ROT	(for ROT_MODE = TETHERED only) Maximum deviation allowed from orientation of reference principle axes	float (degrees)	30.0	>0.0 - 180.0
MAX_DIHEDRAL	(for DIHEDRAL_MODE = TETHERED only) Maximum deviation allowed from reference dihedral angles for any rotatable bond	float (degrees)	30.0	>0.0 - 180.0
Advanced parameters (should not need to be changed by the user)				
TRANS_STEP	Maximum mutation step size for ligand translational degrees of freedom	float (Angstroms)	2.0	>0.0
ROT_STEP	Maximum mutation step size for ligand whole-body rotational degrees of freedom	float (degrees)	30.0	>0.0
DIHEDRAL_STEP	Maximum mutation step size for ligand internal dihedral degrees of freedom	float (degrees)	30.0	>0.0

Solvent definition

Solvent definition parameters need only be defined if you wish to introduce explicit structural waters into the docking calculation, otherwise this section is not required. All solvent definition parameters should be defined in `SECTION SOLVENT`.

Table 8.3. Solvent definition parameters

Parameter	Description	Type	Default	Range of values
Main user parameters				
FILE	Name of explicit solvent PDB file	File name string	No default value (mandatory parameter)	Valid PDB filename
TRANS_MODE	Sampling mode for solvent translational degrees of freedom. If defined here, the value overrides the per-solvent translational sampling modes defined in the solvent PDB file	enumerated string literal	FREE	FIXED<br>TETHERED<br>FREE
ROT_MODE	Sampling mode for solvent whole-body rotational degrees of freedom. If defined here, the value overrides the per-solvent rotational sampling modes defined in the solvent PDB file	enumerated string literal	FREE	FIXED<br>TETHERED<br>FREE
MAX_TRANS	(for TRANS_MODE = TETHERED waters only)	float (Angstroms)	1.0	>0.0

Parameter	Description	Type	Default	Range of values
	Maximum deviation allowed from reference water oxygen positions. The same value is applied to all waters with TRANS_MODE = TETHERED; it is not possible currently to define per-solvent MAX_TRANS values.			
MAX_ROT	(for ROT_MODE = TETHERED waters only) Maximum deviation allowed from orientation of reference principal axes. The same value is applied to all waters with ROT_MODE = TETHERED; it is not possible currently to define per-solvent MAX_ROT values.	float (degrees)	30.0	>0.0 - 180.0
OCCUPANCY	Controls occupany state sampling for all explicit solvent. If defined here, the value overrides the per-solvent occupancy states defined in the solvent PDB file.	float	1.0	0.0 - 1.0
Advanced parameters (should not need to be changed by the user)				
TRANS_STEP	Maximum mutation step size for solvent translational degrees of freedom	float (Angstroms)	2.0	>0.0
ROT_STEP	Maximum mutation step size for solvent whole-body rotational degrees of freedom	float (degrees)	30.0	>0.0
OCCUPANCY_STEP	Maximum mutation step size for solvent occupancy state degrees of freedom.	float (degrees)	1.0	0.0 - 1.0

Solvent occupancy state sampling. OCCUPANCY = 0 permanently disables all solvent; OCCUPANCY = 1.0 permanently enables all solvent; OCCUPANCY between 0 and 1 activates variable occupancy state sampling, where the value represents the initial probability that the solvent molecule is enabled. For example, OCCUPANCY = 0.5 means that the solvent is enabled in 50% of the initial GA population. However, the probability that the solvent is actually enabled in the final docking solution will depend on the particular ligand, the scoring function terms, and on the penalty for solvent binding. The occupancy state chromosome value is managed as a continuous variable between 0.0 and 1.0, with a nominal mutation step size of 1.0. Chromosome values lower than the occupancy threshold (defined as 1.0 - OCCUPANCY) result in the solvent being disabled; values higher than the threshold result in the solvent being enabled

Cavity mapping

The cavity mapping section is mandatory. You should choose one of the mapping algorithms shown below. All mapping parameters should be defined in SECTION MAPPER.

Table 8.4. Two sphere site mapping parameters

Parameter	Description	Type	Default	Range of values
Main user parameters				
SITE_MAPPER	Mapping algorithm specifier	string literal	RbtSphereSiteMapper	fixed
CENTER	(x,y,z) center of cavity mapping region	Bracketed cartesian coordinate string (x,y,z)	None	None
RADIUS	Radius of cavity mapping region	float (Angstroms)	10.0	> 0.0 (10.0-20.0 suggested range)
SMALL_SPHERE	Radius of small probe	float (Angstroms)	1.5	> 0.0 (1.0-2.0 suggested range)
LARGE_SPHERE	Radius of large probe	float (Angstroms)	4.0	> SMALL_SPHERE (3.5 - 6.0 suggested range)
MAX_CAVITIES	Maximum number of cavities to accept (in descending order of size)	integer	99	>0
Advanced parameters (less frequently changed by the user)				
VOL_INCR	Receptor atom radius increment for excluded volume	float (Angstroms)	0.0	>= 0.0
GRID_STEP	Grid resolution for mapping	float (Angstroms)	0.5	>0.0 (0.3 - 0.8 suggested range)
MIN_VOLUME	Minimum cavity volume to accept (in A^3, not grid points)	float (Angstroms^3)	100	>0 (100-300 suggested range)

Table 8.5. Reference ligand site mapping parameters

Parameter	Description	Type	Default	Range of values
Main user parameters				
SITE_MAPPER	Mapping algorithm specifier	string literal	RbtLigandSiteMapper	fixed
REF_MOL	Reference ligand SD file name	string	ref.sd	None
RADIUS	Radius of cavity mapping region	float (Angstroms)	10.0	> 0.0 (10.0-20.0 suggested range)

Parameter	Description	Type	Default	Range of values
SMALL_SPHERE	Radius of small probe	float (Angstroms)	1.5	> 0.0 (1.0-2.0 suggested range)
LARGE_SPHERE	Radius of large probe	float (Angstroms)	4.0	> SMALL_SPHERE (3.5 - 6.0 suggested range)
MAX_CAVITIES	Maximum number of cavities to accept (in descending order of size)	integer	99	>0
Advanced parameters (less frequently changed by the user)				
VOL_INCR	Receptor atom radius increment for excluded volume	float (Angstroms)	0.0	>= 0.0
GRID_STEP	Grid resolution for mapping	float (Angstroms)	0.5	>0.0 (0.3 - 0.8 suggested range)
MIN_VOLUME	Minimum cavity volume to accept (in A^3, not grid points)	float (Angstroms^3)	100	>0 (100-300 suggested range)

Cavity restraint

The cavity restraint penalty function is mandatory and is designed to prevent the ligand from exiting the docking site. The function is calculated over all non-hydrogen atoms in the ligand (and over all explicit water oxygens that can translate). The distance from each atom to the nearest cavity grid point is calculated. If the distance exceeds the value of `RMAX`, a penalty is imposed based on the value of (distance - `RMAX`). The penalty can be either linear or quadratic depending on the value of the `QUADRATIC` parameter.

It should not be necessary to change any the parameters in this section. Note that the docking protocol itself will manipulate the `WEIGHT` parameter, so any changes made to `WEIGHT` will have no effect.

```
SECTION CAVITY
    SCORING_FUNCTION        RbtCavityGridSF
    WEIGHT                  1.0
    RMAX                    0.1
    QUADRATIC               FALSE
END_SECTION
```

Pharmacophore restraints

This section need only be defined if you wish to dock with pharmacophore restraints. If you are running conventional free docking then this section is not required. All pharmacophore definition parameters should be defined in `SECTION PHARMA`.

Table 8.6. Pharmacophore restraint parameters

Parameter	Description	Type	Default	Range of values
CONSTRAINTS_FILE	Mandatory pharmacophore	File name	None	Valid file

Parameter	Description	Type	Default	Range of values
	restraints file	string	(mandatory parameter)	name
OPTIONAL_FILE	Optional pharmacophore restraints file	File name string	None (optional parameter)	Valid file name, or empty
NOPT	Number of optional restraints that should be met	Integer	0	Between 0 and number of restraints in OPTIONAL_FILE
WRITE_ERRORS	Ligands with insufficient pharmacophore features to match the mandatory restraints are always removed prior to docking. If this parameter is true, the pre-filtered ligands are written to an error SD file with the same root name as the docked pose output SD file, but with an _errors.sd suffix. If false, the pre-filtered ligands are not written.	Boolean	false	true or false
WEIGHT	Overall weight for the pharmacophore penalty function	Float	1.0	>= 0.0

Calculation of mandatory restraint penalty. The list of ligand atoms that matches each restraint type in the mandatory restraints file is precalculated for each ligand as it is loaded. If the ligand contains insufficient features to satisfy all of the mandatory restraints the ligand is rejected and is not docked. Note that the rejection is based purely on feature counts and does not take into account the possible geometric arrangements of the features. Rejected ligands are optionally written to an error SD file. The penalty for each restraint is based on the distance from the nearest matching ligand atom to the pharmacophore restraint centre. If the distance is less than the defined tolerance (restraint sphere radius), the penalty is zero. If the distance is greater than the defined tolerance a quadratic penalty is applied, equal to (nearest distance - tolerance)^2.

Calculation of optional restraint penalty. The individual restraint penalties for each restraint in the optional restraints file are calculated in the same way as for the mandatory penalties. However, only the NOPT lowest scoring (least penalised) restraints are summed for any given docking pose. Any remaining higher scoring optional restraints are ignored and do not contribute to the total pharmacophore restraint penalty.

Calculation of overall restraint penalty. The overall pharmacophore restraint penalty is the sum of the mandatory restraint penalties and the NOPT lowest scoring optional restraint penalties, multiplied by the WEIGHT parameter value.

NMR restraints

To be completed. However, this feature has rarely been used.

Example system definition files

Example 8.1. Full system definition file with all sections and common

parameters enumerated explicitly

```
RBT_PARAMETER_FILE_V1.00
TITLE HSP90-PU3-lig-cavity, solvent flex=5

RECEPTOR_FILE PROT_W3_flex.mol2
RECEPTOR_SEGMENT_NAME PROT
RECEPTOR_FLEX 3.0

SECTION SOLVENT
    FILE PROT_W3_flex_5.pdb
    TRANS_MODE TETHERED
    ROT_MODE TETHERED
    MAX_TRANS 1.0
    MAX_ROT 30.0
    OCCUPANCY 0.5
END_SECTION

SECTION LIGAND
    TRANS_MODE FREE
    ROT_MODE FREE
    DIHEDRAL_MODE FREE
    MAX_TRANS 1.0
    MAX_ROT 30.0
    MAX_DIHEDRAL 30.0
 END_SECTION

SECTION MAPPER
    SITE_MAPPER RbtLigandSiteMapper
    REF_MOL ref.sd
    RADIUS 5.0
    SMALL_SPHERE 1.0
    MIN_VOLUME 100
    MAX_CAVITIES 1
    VOL_INCR 0.0
    GRIDSTEP 0.5
END_SECTION

SECTION CAVITY
    SCORING_FUNCTION RbtCavityGridSF
    WEIGHT 1.0
END_SECTION

SECTION PHARMA
    SCORING_FUNCTION RbtPharmaSF
    WEIGHT 1.0
    CONSTRAINTS_FILE mandatory.const
    OPTIONAL_FILE optional.const
    NOPT 3
    WRITE_ERRORS TRUE
END_SECTION
```

Chapter 9. rDock file formats

.prm file format

The .prm file format is an rDock-specific text format and is used for:

- system definition files (known previously as receptor .prm files)
- scoring function definition files
- search protocol definition files

The format is simple and allows for an arbitrary number of named parameter/value pairs to be defined, optionally divided into named sections. Sections provide a namespace for parameter names, to allow parameter names to be duplicated within different sections.

The key features of the format are:

- The first line of the file must be `RBT_PARAMETER_FILE_V1.00` with no preceeding whitespace.
- Subsequent lines may contain either:
  1. comment lines
  2. reserved keywords `TITLE`, `SECTION`, or `END_SECTION`
  3. parameter name/value pairs
- Comment lines should start with a `#` character in the first column with no preceeding whitespace, and are ignored.
- The reserved words must start in the first column with no preceeding whitespace.
- The `TITLE` record should occur only once in the file and is used to provide a title string for display by various scripts such as run_rbscreen.pl. The keyword should be followed by a single space character and then the title string, which may contain spaces. If the `TITLE` line occurs more than once, the last occurence is used.
- `SECTION` records can occur more than once, and should always be paired with a closing `END_SECTION` record. The keyword should be followed by a single space character and then the section name, which may NOT itself contain spaces. All section names must be unique within a .prm file. All parameter name/value pairs within the `SECTION` / `END_SECTION` block belong to that section.
- Parameter name/value pairs are read as free-format tokenised text and can have preceeding, trailing, and be separated by arbitrary whitespace. This implies that the parameter name and value strings themselves are not allowed to contain any spaces. The value strings are interpreted as numeric, string, or boolean values as appropriate for that parameter. Boolean values should be entered as `TRUE` or `FALSE` uppercase strings.

Caution

The current implementation of the .prm file reader does not tolerate a `TAB` character immediately following the `TITLE` and `SECTION` keywords. It is very important that the first character after the `SECTION` keyword in particular is a true space character, otherwise the reserved word will not be detected and the parameters for that section will be ignored.

Example 9.1. Example .prm file

In the following example, `RECEPTOR_FILE` is defined in the top level namespace. The remaining parameters are defined in the `MAPPER` and `CAVITY` namespaces. The indentation is for readability, and has no significance in the format.

```
RBT_PARAMETER_FILE_V1.00
TITLE 4dfr oxido-reductase

RECEPTOR_FILE 4dfr.mol2

SECTION MAPPER
    SITE_MAPPER RbtLigandSiteMapper
    REF_MOL 4dfr_c.sd
    RADIUS 6.0
    SMALL_SPHERE 1.0
    MIN_VOLUME 100
    MAX_CAVITIES 1
    VOL_INCR 0.0
    GRIDSTEP 0.5
END_SECTION

SECTION CAVITY
    SCORING_FUNCTION RbtCavityGridSF
    WEIGHT 1.0
END_SECTION
```

Water PDB file format

rDock requires explicit water PDB files to be in the style as output by the Dowser program. In particular:

- Records can be HETATM or ATOM
- The atom names must be `OW`, `H1` and `H2`
- The atom records for each water molecule must belong to the same subunit ID
- The subunit IDs for different waters must be distinct, but do not have to be consecutive.
- The atom IDs are not used and do not have to be consecutive (they can even be duplicated)
- The order of the atom records within a subunit is unimportant
- The temperature factor field of the water oxygens can be used to define the per-solvent flexibility modes. The temperature factors of the water hydrogens are not used.

Table 9.1. Conversion of temperature factor values to solvent flexibility modes

PDB temperature factor	Solvent translational flexibility	Solvent rotational flexibility
0	`FIXED`	`FIXED`
1	`FIXED`	`TETHERED`
2	`FIXED`	`FREE`

PDB temperature factor	Solvent translational flexibility	Solvent rotational flexibility
3	TETHERED	FIXED
4	TETHERED	TETHERED
5	TETHERED	FREE
6	FREE	FIXED
7	FREE	TETHERED
8	FREE	FREE

Example 9.2. Valid rDock PDB file for explicit, flexible waters

```
REMARK tmp_1YET.pdb xtal_hoh.pdb
HETATM 3540  OW  HOH W 106      28.929  12.684  20.864  1.00  1.0
HETATM 3540  H1  HOH W 106      28.034  12.390  21.200  1.00
HETATM 3540  H2  HOH W 106      29.139  12.204  20.012  1.00
HETATM 3542  OW  HOH W 108      27.127  14.068  22.571  1.00  2.0
HETATM 3542  H1  HOH W 108      26.632  13.344  23.052  1.00
HETATM 3542  H2  HOH W 108      27.636  13.673  21.806  1.00
HETATM 3679  OW  HOH W 245      27.208  10.345  27.250  1.00  3.0
HETATM 3679  H1  HOH W 245      27.657  10.045  26.409  1.00
HETATM 3679  H2  HOH W 245      26.296  10.693  27.036  1.00
HETATM 3680  OW  HOH W 246      31.737  12.425  21.110  1.00  4.0
HETATM 3680  H1  HOH W 246      31.831  12.448  22.106  1.00
HETATM 3680  H2  HOH W 246      30.775  12.535  20.863  1.00
```

Pharmacophore restraints file format

Pharmacophore restraints are defined in a simple text file, with one restraint per line. Each line should contain the following values, separated by commas or whitespace:

`x y z coords of restraint centre`, *`tolerance (in Angstroms)`*, *`restraint type string`*.

The supported restraint types are:

Table 9.2. Pharmacophore restraint types

String	Description	Matches
Any	Any atom	Any non-hydrogen atom
Don	H-bond donor	Any neutral donor hydrogen
Acc	H-bond acceptor	Any neutral acceptor
Aro	Aromatic	Any aromatic ring centre (pseudo atom)
Hyd	Hydrophobic	Any non-polar hydrogens (if present), any Csp3 or Ssp3, any C or S not bonded to Osp2, any Cl, Br, I
Hal	Hydrophobic, aliphatic	Subset of Hyd, sp3 atoms only
Har	Hydrophobic, aromatic	Subset of Hyd, aromatic atoms only
Ani	Anionic	Any atom with negative distrib-

**String**	**Description**	**Matches**
		uted formal charge
Cat	Cationic	Any atom with positive distributed formal charge

Chapter 10. rDock programs

Executable (binary) programs

Table 10.1. Core rDock C++ executables

Executable	Used for	Description
rbcavity	Preparation	Cavity mapping and preparation of docking site (.as) file
rbcalcgrid	Preparation	Calculation of vdW grid files (usually called by `make_grid.csh` wrapper script)
rbdock	Docking	The main rDock docking engine itself.
rbrms	Analysis	Calculation of ligand Root Mean Squared Displacement (RMSD) between reference and docked poses

Table 10.2. Auxiliary rDock C++ executables

Executable	Used for	Description
rbtether	Preparation	Prepares a ligand SD file for tethered scaffold docking. Requires Daylight SMARTS license. Annotates ligand SD file with tethered substructure atom indices.
smart_rms	Analysis	Calculation of ligand Root Mean Squared Displacement (RMSD) between reference and docked poses, taking into account ligand topological symmetry. Requires Daylight SMARTS license.
rbconvgrid	Analysis	Converts rDock vdW grids to InsightII grid format for visualisation
rbmoegrid	Analysis	Converts rDock vdW grids to MOE grid format for visualisation
rblist	Analysis	Outputs miscellaneous information for ligand SD file records

Scripts

Table 10.3. Core scripts

Script	Used for	Description
make_grid.csh	Preparation	Creates the vdW grid files required for grid-based docking protocols (`dock_grid.prm` and `dock_solv_grid.prm`). Simple front-end to `rbcalcgrid`.
run_rbdock.pl	Docking	Simple front-end to rbdock, suitable for small-scale docking experiments. Can create command files for a variety of queuing systems (Condor, SGE, NQS).
run_rbscreen.pl	Docking	Front-end to rbdock for launching large-scale virtual screening campaigns. Creates a command file for Condor queuing system only.
sdfilter	Analysis	Utility for filtering SD files by arbitrary data field expressions. Useful for simple post-docking filtering by score

Script	Used for	Description
		components.
sdsort	Analysis	Utility for sorting SD files by arbitrary data field. Useful for simple post-docking filtering by score components.
sdreport	Analysis	Utility for reporting SD file data field values. Output in tab-delimited or csv format.

Table 10.4. Auxiliary scripts

Script	Used for	Description
ht_protocol_finder .csh	Preparation	Used to optimise a high-throughput docking protocol from an initial exhaustive docking of a small representative ligand library.
sdsplit	Utility	Splits an SD file into multiple smaller SD files of fixed number of records.
sdmodify	Utility	Sets the molecule title line of each SD record equal to a given SD data field.

Command-line reference

Name

rbdock -- the rDock docking engine itself.

```
$RBT_ROOT/bin/rbdock
{-i input ligand MDL SD file}
{-o output MDL SD file}
{-r system definition .prm file}
{-p docking protocol .prm file}
[-n number of docking runs/ligand]
[-s random seed]
[-T debug trace level]
[[-t SCORE.INTER threshold] | [-t filter definition file]]
[ -ap -an -allH -cont ]
```

Usage

Simple exhaustive docking. The minimum requirement for **rbdock** is to specify the input (`-i`) and output (`-o`) ligand SD file names, the system definition .prm file (`-r`) and the docking protocol .prm file (`-p`). This will perform one docking run per ligand record in the input SD file and output all docked ligand poses to the output SD file. Use `-n` to increase the number of docking runs per ligand record.

High-throughput docking 1. The `-t` and `-cont` options can be used to construct high-throughput protocols. If the argument following `-t` is numeric it is interpreted as a threshold value for `SCORE.INTER`, the total intermolecular score between ligand and receptor/solvent. In the absence of `-cont`, the threshold acts as an early termination filter, and the docking runs for each ligand will be terminated early once the threshold value has been exceeded. Note that the threshold is applied only at the end of each individual docking run, not during the runs themselves. If the `-cont` (continue) option is specified as well, the threshold acts as an output pose filter instead of a termination filter. The docking runs for each ligand run to completion as in the exhaustive case, but only the docking poses that exceed the threshold value of `SCORE.INTER` are written to the output SD file.

High throughput docking 2. Alternatively, if the argument following `-t` is non-numeric it is interpreted as a filter definition file. The filter definition file can be used to define multiple termination filters and multiple output pose filters in a generic way. Any docking score component can be used in the filter definitions. `run_rbscreen.pl` generates a filter definition file for multi-stage, high-throughput docking, with progressive score thresholds for early termination of poorly performing ligands. The use of filter definition files is preferred over the more limited `SCORE.INTER` filtering described above, whose use is now deprecated.

Automated ligand protonation/deprotonation. The `-ap` option activates the automated protonation of ligand positive ionisable centres, notably amines, guanidines, imidazoles, and amidines. The `-an` option activates the automated deprotonation of ligand negative ionisable centres, notably carboxylic acids, phosphates, phosphonates, sulphates, and sulphonates. The precise rules used by rDock for protonation and deprotonation are quite crude, and are not user-customisable. Therefore these flags are not recommended for detailed validation experiments, in which care should be taken that the ligand protonation states are set correctly in the input SD file. Note that rDock is not capable of converting ionised centres back to the neutral form; these are unidirectional transformations.

Control of ligand non-polar hydrogens. By default, rDock uses an implicit non-polar hydrogen model for receptor and ligand, and all of the scoring function validation has been performed on this basis. If the `-allH` option is not defined (recommended), all explicit non-polar hydrogens encountered in the ligand input SD file are removed, and only the polar hydrogens (bonded to O, N, or S) are retained. If the `-allH` option is defined (not recommended), no hydrogens are removed from the ligand. Note that rDock is not capable of *adding* explicit non-polar hydrogens, if none exist. In other words, the `-allH` option disables hydrogen removal, it does not activate hydrogen addition. You should always make sure that polar hydrogens are defined explicitly. If the ligand input SD file contains no explicit non-polar hydrogens, the `-allH` option has no effect. Receptor protonation is controlled by the system definition prm file.

Name

rbcavity -- Cavity mapping and preparation of docking site (.as) file file

`$RBT_ROOT/bin/rbcavity`
{-r *system definition .prm file*}
[`-ras` `-was` `-d` `-v` `-s`]
[-l *distance from cavity*]
[-b *border*]

Usage

Exploration of cavity mapping parameters.

`rbcavity -r` *.prm file*

You can run `rbcavity` with just the `-r` argument when first preparing a new receptor for docking. This allows you to explore rapidly the impact of the cavity mapping parameters on the generated cavities, whilst avoiding the overhead of actually writing the docking site (.as) file to disk. The number of cavities and volume of each cavity are written to standard output.

Visualisation of cavities.

`rbcavity -r` *.prm file* `-d`

If you have access to InsightII you can use the `-d` option to dump the cavity volumes in InsightII grid file format. There is no need to write the docking site (.as) file first. The InsightII grid files should be loaded into the reference coordinate space of the receptor and contoured at a contour level of 0.99.

Writing the docking site (.as) file.

`rbcavity -r` *.prm file* `-was`

When you are happy the mapping parameters, use the `-was` option to write the docking site (.as) file to disk. The docking site file is a binary file that contains the cavity volumes in a compact format, and a pre-calculated cuboid grid extending over the cavities. The grid represents the distance from each point in space to the nearest cavity grid point, and is used by the cavity penalty scoring function. Calculating the distance grid can take a long time (whereas the cavity mapping itself is usually very fast), hence the `-was` option should be used sparingly.

Analysis of cavity atoms.

`rbcavity -r` *.prm file* `-ras -l` *distance*

Use the `-l` options to list the receptor atoms within a given distance of any of the cavity volumes, for example to determine which receptor OH/NH3+ groups should be flexible. This option requires access to the pre-calculated distance grid embedded within the docking site (.as) file, and is best used in combination with the `-ras` option, which loads a previously generated docking site file. This avoids the time consuming step of generating the cavity distance grid again. If `-l` is used without `-ras`, the cavity distance grid will be calculated on-the-fly each time.

Miscellaneous options. The `-s` option writes out various statistics on the cavity and on the receptor atoms in the vicinity of the cavity. These values have been used in genetic programming model building for docking pose false positive removal. The `-v` option writes out the receptor coordinates in PSF/CRD format for use by the rDock Viewer (not documented here). Note that the PSF/CRD files are not suitable for simulation purposes, only for visualisation, as the atom types are not set correctly. The `-b` option controls the size of the cavity distance grid, and represents the border beyond the actual cavity volumes. It should not be necessary to vary this parameter (default = 8A) unless longer-range scoring functions are implemented.

Name

rbcalcgrid -- Calculation of vdW grid files (usually called by `make_grid.csh` wrapper script)

`$RBT_ROOT/bin/rbcalcgrid`
{-r*system definition file*}
{-o*output suffix for generated grids*}
{-p*vdW scoring function prm file*}
[-g*grid step*]
[-b*border*]

Note that, unlike `rbdock` and `rbcavity`, spaces are not tolerated between the command-line options and their corresponding arguments.

Usage

See `$RBT_ROOT/bin/make_grid.csh` for common usage.

Name

rbrms -- Calculation of ligand Root Mean Squared Displacement (RMSD) between reference and docked poses

`$RBT_ROOT/bin/rbrms` { *reference SD file* } { *input SD file* } [*output SD file*] [*RMSD threshold*]

Usage

With two arguments. `rbrms` calculates the RMSD between each record in the input SD file and the first record of the reference SD file. If there is a mismatch in the number of atoms, the record is skipped and the RMSD is not calculated. The RMSD is calculated over the heavy (non-hydrogen) atoms only. Results are output to standard output.

With three arguments. As for two arguments, plus each record in the input SD file is written to the output SD file with the calculated RMSD value stored as an SD data field (`RMSD` field).

With four arguments. As for three arguments, with an RMSD filter applied to the output SD file. Records are not written if they have an RMSD less than the threshold value with any previous record in the input SD file. This provides a crude way of removing duplicate poses.

Name

rbtether -- Prepares a ligand SD file for tethered scaffold docking. Requires Daylight SMARTS license. Annotates ligand SD file with tethered substructure atom indices.

```
$RBT_ROOT/bin/rbtether
{-rreference ligand SD file}
{-iinput ligand SD file}
{-sSMARTS query}
{-ooutput root}
[ -ap -an -allH ]
```

Note that, unlike `rbdock` and `rbcavity`, spaces are not tolerated between the command-line options and their corresponding arguments.

Usage

Standard options.

`rbtether -rref -iinput -ooutput -squery`

`rbtether` performs the following actions:

- Runs the SMARTS query against the reference SD file to determine the tethered substructure atom indices and coordinates.
- If more than one substructure match is retrieved (e.g. due to topological symmetry, or if the query is too simple) only the first substructure match is retained as the reference
- Runs the SMARTS query against each record of the input ligand SD file in turn.
- For each substructure match, the ligand coordinates are transformed such that the principal axes of the matching substructure coordinates are aligned with the reference substructure coordinates.
- In addition, an SD data field is added to the ligand record which lists the atom indicies of the substructure match, for later retrieval by `rbdock`.
- Each transformed ligand is written to the output SD file.
- Note that if the SMARTS query returns more than one substructure match for a ligand, that ligand is written multiple times to the output file, once for each match, each of which will be docked independently with different tethering information

Determination of reference SMILES.

`rbtether -rref`

If `rbtether` is run with only the `-r` option (with no SMARTS query or ligand input or output files) the SMILES string for the entire reference ligand is reported to standard output and the program terminates with no further action. The SMILES string can often be used as the SMARTS query if the aim is to tether each ligand to the entire reference ligand substructure.

Miscellaneous options. `-ap -an -allH` have the same meaning as described for `rbdock`
