# Supplementary material for: rDock: A Fast, Versatile and Open Source Program for Docking Ligands to Proteins and Nucleic Acids
Source: PLoS Comput Biol. 2014 Apr 10;10(4):e1003571. doi: 10.1371/journal.pcbi.1003571 (PMC3983074; doi:10.1371/journal.pcbi.1003571)
Supplement: Software S1 — Compressed file with the source code of the rDock software for ligand docking to Proteins and Nucleic Acids. (GZ) [file pcbi.1003571.s010.gz › rDock_2013.1_src/docs/rDock_getting_started.pdf]

Getting Started with rDock

Dr. David Morley

Getting Started with rDock

Dr. David Morley

Copyright © 2006 Vernalis

Table of Contents

Overview vi
1. Prerequisites 1
2. Unpacking the distribution files 2
3. Building rDock 4
4. Running a short validation experiment 6

List of Tables

1.1. Required packages for building and running rDock 1
2.1. rDock distribution files 2
3.1. Standard `tmake` build targets provided 4
4.1. Complexes included in the `cpu_benchmark` experiment 6
4.2. rDock score components output as SD data fields 7

Overview

rDock is a high-throughput molecular docking platform for protein and RNA targets. Under development at Vernalis (formerly RiboTargets) since 1998, the software (formerly known as RiboDock[1]), scoring functions, and search protocols have been refined continuously to meet the demands of in-house Structure-Based Drug Discovery (SBDD) projects.

The major components of the platform now include fast intermolecular scoring functions (van der Waals, polar, desolvation) validated against protein and RNA targets, a Genetic Algorithm-based stochastic search engine, a wide variety of external SBDD-derived restraint terms (tethered template, pharmacophore, noe distance restraints), and novel Genetic Programming-based post-docking filtering. A variety of scripts are provided to perform automated validation experiments and to launch virtual screening campaigns.

This introductory guide is aimed at new users of rDock. It describes the minimal set of steps required to build rDock from the source code distribution, and to run one of the automated validation experiments provided in the test suite distribution. The instructions assume that you are comfortable with simple Linux command line administration tasks, and with building Linux applications from make files. Once you are familiar with these steps you should proceed to the User and Reference Guide for more detailed documentation on the usage of rDock

[1] Validation of an empirical RNA-ligand scoring function for fast flexible docking using RiboDock, SD Morley and M Afshar, J. Comput.-Aided Mol. Des., 18 (2004) 189-208.

Chapter 1. Prerequisites

Compilers. rDock is supplied as source code, which means that you will have to compile the binary files (run-time libraries and executable programs) before you can use them. rDock has been developed largely on the Linux operating system, most recently with the GNU g++ compiler under SuSE 9.2 Pro. The code will almost certainly compile and run under other Linux distributions with little or no modification, but no other distributions or compilers have been tested extensively to date.

Condor. Although the rDock executables can be run directly from the command line, the validation experiment scripts use the Condor High Throughput Computing software for automating the process of distributing and managing individual docking jobs across a cluster of compute machines. Condor is available free of charge for a wide variety of platforms[1]. Installing and configuring Condor is beyond the scope of this guide as the Condor configuration is very site-dependent.

For full production use, you would typically compile rDock on a separate build machine and run the docking calculations on a cluster of compute machines. However, for the purposes of getting started, these instructions assume that you will be compiling rDock and running the initial validation experiments on the same machine, and that you have installed and configured a *Personal* Condor to manage the jobs on that machine.

Required packages. Make sure you have the following packages installed on your machine before you continue. The versions listed are appropriate for SuSE 9.2 Pro; other versions may be required for your particular Linux distribution.

Table 1.1. Required packages for building and running rDock

**Package**	**Description**	**Required at**	**Version**
gcc	GNU C compiler	Compile-time	3.3.4
g++	GNU C++ compiler	Compile-time	3.3.4
popt-devel	C++ command-line argument processing	Compile-time	1.7-190
cppunit	C++ unit test framework	Compile-time	1.10.2
popt	C++ command-line argument processing	Run-time	1.7-190
Condor	Distributed resource management	Run-time	6.6.10

[1]Condor is available from the University of Wisconsin (`http://www.cs.wisc.edu/condor`)

Chapter 2. Unpacking the distribution files

The rDock source files and test suite files are provided as independent gzipped tar (.tgz) distributions. Depending on your requirements, the two distributions can be unpacked to entirely separate locations, or can be unpacked under the same location. In this example they are unpacked under the same location.

Table 2.1. rDock distribution files

File	Description
rdock_[CODELINE]_[BUILDNUM]_src.tgz	rDock source distribution
RBT_TEST_[DATE].tgz	Test suite data files and scripts

where [CODELINE], [BUILDNUM], and [DATE] will vary depending on the release. [CODELINE] represents the major version string (for example, `2006.1`), [BUILDNUM] represents the minor version number (for example, `865`) and [DATE] represents a date string (for example, `20060313`).

Procedure 2.1. Example unpacking procedure

1. **Create a new directory for building rDock.**

   ```
   $ mkdir ~/dev
   ```

   The directory you created is referred to as [BUILDDIR] in the subsequents steps.

2. **Copy or download the distribution files to [BUILDDIR].**

   ```
   $ cp ~/mydownloads/rdock_2006.1_865_src.tgz ~/dev/
   $ cp ~/mydownloads/RBT_TEST_20060313.tgz ~/dev/
   ```

3. **Extract the distributions.**

   ```
   $ cd ~/dev/
    $ tar xvzf rdock_2006.1_865_src.tgz
    $ tar xvzf RBT_TEST_20060313.tgz
   ```

   The distributions contain files with relative path names, and you should find the following subdirectories created under [BUILDDIR]. Note that the `./rdock/2006.1` subdirectory may have a different name depending on the major version string (see above).

   ```
   $ find . -type d
   .
   ./rdock
   ./rdock/2006.1
   ./rdock/2006.1/fw
   ./rdock/2006.1/bin
   ./rdock/2006.1/lib
   ./rdock/2006.1/src
   ./rdock/2006.1/src/GP
   ./rdock/2006.1/src/exe
   ./rdock/2006.1/src/lib
   ```

```
./rdock/2006.1/src/daylight
./rdock/2006.1/data
./rdock/2006.1/data/sf
./rdock/2006.1/data/pmf
./rdock/2006.1/data/pmf/smoothed
./rdock/2006.1/data/scripts
./rdock/2006.1/data/filters
./rdock/2006.1/docs
./rdock/2006.1/docs/images
./rdock/2006.1/build
./rdock/2006.1/build/test
./rdock/2006.1/build/test/RBT_HOME
./rdock/2006.1/build/tmakelib
./rdock/2006.1/build/tmakelib/unix
./rdock/2006.1/build/tmakelib/linux-pathCC-64
./rdock/2006.1/build/tmakelib/linux-g++-64
./rdock/2006.1/build/tmakelib/linux-g++
./rdock/2006.1/import
./rdock/2006.1/import/nr
./rdock/2006.1/import/nr/src
./rdock/2006.1/import/nr/include
./rdock/2006.1/import/simplex
./rdock/2006.1/import/simplex/src
./rdock/2006.1/import/simplex/include
./rdock/2006.1/include
./rdock/2006.1/include/GP
./RBT_TEST
./RBT_TEST/na
./RBT_TEST/bin
./RBT_TEST/ccdc_astex
```

4. **Make a note of the following locations for later use.**

   The test suite root directory is `[BUILDDIR]/RBT_TEST/` and will be referred to as `[RBT_TEST]` in later instructions. In this example, `[RBT_TEST]` is `~/dev/RBT_TEST/`.

   The rDock root directory is `[BUILDDIR]/rdock/[CODELINE]` and will be referred to as `[RBT_ROOT]` in later instructions. In this example, `[RBT_ROOT]` is `~/dev/rdock/2006.1/`.

Chapter 3. Building rDock

rDock is written in C++ (with a small amount of C code from Numerical Recipes) and makes heavy use of the C++ Standard Template Library (STL). The majority of the source code is compiled into a single shared library (`libRbt.so`). The executable programs themselves are relatively light-weight command-line applications linked with `libRbt.so`.

The tmake build system (from Trolltech) is used to generate makefiles automatically for a particular build target (i.e. combination of operating system and compiler). The source distribution comes with tmake templates defining the compiler options and flags for three Linux build targets (`linux-g++`, `linux-g++-64`, and `linux-pathCC-64`). The build targets have been tested under SuSE 9.2 (2.6.8-24.18 kernel) with GNU g++ 3.3.4 and PathScale pathCC 2.1.

Table 3.1. Standard `tmake` build targets provided

Target name	Architecture	Compiler	Compiler flags (release build)
`linux-g++`	32-bit Intel	`g++`	`-m32 -O3 -ffast-math -march=pentium3 -mcpu=pentium3`
`linux-g++-64`	64-bit AMD Opteron	`g++`	`-m64 -O3 -ffast-math -march=k8 -mcpu=k8`
`linux-pathCC-64`	64-bit AMD Opteron	`pathcc`	`-Ofast -ffast-math -march=opteron -mtune=opteron`

Customising the tmake template for a build target. If none of the tmake templates are suitable for your machine, or if you wish to customise the compiler options, you should first customise one of the existing templates. The tmake template files are stored under `[RBT_ROOT]/build/tmakelib/`. Locate and edit the `tmake.conf` file for the build target you wish to customise. For example, to customise the `linux-g++` build target, edit `[RBT_ROOT]/build/tmakelib/linux-g++/tmake.conf` and localise the values to suit your compiler.

Procedure 3.1. rDock build procedure

To build rDock, first go to the `[RBT_ROOT]/build/` directory.

```
$ cd [RBT_ROOT]/build
```

1. **Compile**

   Make one of the build targets listed below.

   ```
   $ make linux-g++
   $ make linux-g++-64
   $ make linux-pathCC-64
   ```

2. **Test**

   Run the rDock unit tests to check build integrity. If no failed tests are reported you should be all set.

   ```
   $ make test
   ```

3. **Install (optional)**

   You can either run rDock directly from the build location (only recommended for initial testing), or install the binaries and data files to a new location (recommended for production use). To install in a new location [INSTALLDIR], create a binary distribution file, copy the distribution file to [INSTALLDIR], and extract the files.

```
$ make dist
$ mkdir -p [INSTALLDIR]
$ cp rdock_[CODELINE].tgz [INSTALLDIR]
$ cd [INSTALLDIR]
$ tar xvzf rdock_[CODELINE].tgz
```

4. **Cleanup (optional)**

   To remove all intermediate build files from `[RBT_ROOT]/build/`, leaving just the final executables (in `[RBT_ROOT]/bin/`) and shared libraries (in `[RBT_ROOT]/lib/`):

```
$ make clean
```

   To remove the final executables and shared libraries as well, returning to a source-only distribution:

```
$ make distclean
```

Chapter 4. Running a short validation experiment

Now that you have successfully built the rDock executables you should change your working directory away from the installed locations of [RBT_TEST] and [RBT_ROOT], for example to a personal project directory. It is not recommended to run validation experiments directly within the [RBT_TEST] and [RBT_ROOT] directory hierarchy.

The cpu_benchmark experiment. The `cpu_benchmark` experiment consists of nine protein-ligand complexes and two RNA-ligand complexes. It represents a small subset of the full rDock docking accuracy validation set and is presented here as the calculations should be short enough to run on a single workstation in a few hours. The full experiments require access to a cluster of machines to run in a tractable length of time. All ligands are non-covalently bound and were chosen to have a range of rotatable bond counts (from 0 to 8)

Table 4.1. Complexes included in the `cpu_benchmark` experiment

Type	PDB codes	File source
Protein-ligand	`1c1e 5abp 1d3h 1wap 1cil 6rnt 1mld 1mts 1lna`	Files used intact from CCDC/Astex "clean" high-resolution test set
RNA-ligand	`1koc 1byj`	Prepared by Vernalis

Configuring the environment. You need to define two environment variables to reflect the location of the test suite [RBT_TEST] and the rDock build you wish to test [RBT_ROOT]. Either customise the example `setup_validation` script (`bash` shell) provided in the [RBT_TEST] directory, and `source` the file, or define the environment variables from the command line. You should also add the [RBT_TEST]/bin and [RBT_ROOT]/bin directories to your path, and add [RBT_ROOT[/lib to your library path. For example, using the directory names used in the example unpacking procedure:

```
$ export RBT_TEST=~/dev/RBT_TEST
$ export RBT_ROOT=~/dev/rdock/2006.1
$ export PATH=${RBT_ROOT}/bin:${RBT_TEST}/bin:${PATH}
$ export LD_LIBRARY_PATH=${RBT_ROOT}/lib:${LD_LIBRARY_PATH}
```

Preparing the docking sites. rDock requires a docking site file (.as suffix) to be defined for each receptor. The .as file (also known as an active site file) defines a volume which represents the region of the receptor into which each ligand should be docked. This process is known as *cavity mapping* and needs be done only once for each receptor prior to running the experiments. The cavity mapping process is quite quick (from a few seconds to a few minutes per complex). The active site files (.as) themselves and associated rDock output log files are generated "in situ" in `$RBT_TEST/ccdc_astex/` and `$RBT_TEST/na/`. Make sure you have write-access to these directories. The only files you will see in your current working directory are the Condor .cmd and .log files. Wait until all Condor jobs have completed before moving to the next step.

```
$ make_cavities cpu_benchmark
$ condor_submit cav_cpu_benchmark.cmd
```

Running the experiment.

```
$ run_cpu_benchmark [TESTDIR]
```

where `[TESTDIR]` is the name of the subdirectory that will be created under your present working directory for the experiment output files. This command creates two Condor command files and

automatically submits the jobs:

`[TESTDIR]/SF3_100/cpu_benchmark.cmd`: 100 docking runs per complex with standard scoring function (SF3)
`[TESTDIR]/SF5_100/cpu_benchmark.cmd`: 100 docking runs per complex with solvation scoring function (SF5)
As a guide, the jobs require around 100 minutes total CPU time on an dual-processor AMD Opteron 248 with 2GB RAM, for an average of around three seconds per individual docking run.

Output files. After all Condor jobs have completed you should find the following output files for each complex in `[TESTDIR]/SF3_100/`and `[TESTDIR]/SF5_100`.

`s_[PDB].sd` Crystallographic reference ligand pose, with rDock scores (score-only protocol)
`m_[PDB].sd` Reference ligand pose minimised under rDock scoring function (minimisation protocol)
`x_[PDB].sd` 100 docked poses (dock protocol)
Each `.sd` file is accompanied by associated `.out` and `.err` files which contain the standard output (rDock output) and standard error for each calculation.

Table 4.2. rDock score components output as SD data fields

Data field	Description
SCORE	Total docking score = SCORE.INTER + SCORE.INTRA + SCORE.SYSTEM + SCORE.RESTR
SCORE.INTER	Total receptor-ligand intermolecular score
SCORE.INTRA	Total ligand intramolecular score
SCORE.SYSTEM	Total intra-receptor, intra-solvent, and receptor-solvent score
SCORE.RESTR	Total external restraint penalty score
SCORE.INTER/INTRA/SYSTEM are weighted sums of the individual scoring function terms listed below. Note that the individual scoring function terms are output as raw values, and must be multiplied by their respective weights (not shown here) in order to reconstitute the total scores.	
SCORE.*.VDW	vdW scores (truncated Tripos 6-12)
SCORE.*.POLAR	Attractive polar interactions (empirical geometric function of distances/angles)
SCORE.*.REPUL	Repulsive polar interactions (SF3 scoring function only)
SCORE.*.SOLV	Desolvation score (weighted SASA, SF5 scoring function only)
SCORE.*.DIHEDRAL	Dihedral score calculated over rotatable bonds only

Reporting the scores. The rDock scores for each pose are embedded within each SD data record as listed in Table 4.2, “rDock score components output as SD data fields”. You can use the `sdreport` script to output the tabulated scores for each pose. For example:

```
$ sdreport -t x_1koc.sd
```

will output the top-level score components (SCORE, SCORE.INTER, SCORE.INTRA, SCORE.RESTR - also SCORE.INTER.VDW) in tab-delimited format. Alternatively you can explicitly list the data fields you wish to output:

```
$ sdreport -tSCORE.INTER.POLAR,SCORE.INTER.VDW x_1koc.sd
```

Calculating the docking accuracy (RMSD). Use the `rmsreport` script to generate the SCORE vs RMSD data files for further analysis, where RMSD is the Root Mean Squared Deviation between a docked ligand pose and the crystallographic reference ligand pose (over non-hydrogens only). Run `rmsreport` within each experiment directory, with no arguments.

```
$ cd [TESTDIR]/SF3_100
$ rmsreport
```

```
$ cd [TESTDIR]/SF5_100
$ rmsreport
```

rmsreport generates a `[PDB].rmcs` file and an `rms[PDB].sd` file for each complex. `rms[PDB].sd` is a sorted, filtered copy of x_[PDB].sd with the RMSD value added as an additional data field. `[PDB].rmcs` is a tabulated text file with six columns where:

Col 1: pose number (sorted by total SCORE)
Col 2: SCORE
Col 3: SCORE.INTER
Col 4: SCORE.INTRA
Col 5: Root Mean Squared Deviation between pose and crystallographic reference (over non-hydrogens only)
Col 6: zero (not used)

Note

Poses that have an excessive cavity penalty (SCORE.RESTR.CAVITY > 1) are removed from the RMSD analysis by rmsreport. A cavity penalty indicates that the ligand pose is not entirely within the defined docking volume, and hence the reported scores can not be trusted.

Example results. View the contents of each .rmcs file. For example, to look at the top three poses for each complex:

```
$ head -3 *.rmcs
```

You should find that most of the top-scoring poses have an RMSD less than 2A. Sample results for the SF3 scoring function are shown below, although as rDock uses a stochastic search your results may differ.

```
== 1byj.rmcs ==
1       -40.955 -33.472 -7.482  2.855   0.000
2       -40.063 -38.091 -1.972  1.457   0.000
3       -38.215 -35.048 -3.168  7.630   0.000

== 1c1e.rmcs ==
1       -27.945 -23.581 0.000   0.265   0.000
2       -27.945 -23.582 0.000   0.264   0.000
3       -27.943 -23.578 0.000   0.263   0.000

== 1cil.rmcs ==
1       -32.052 -28.150 0.517   0.736   0.000
2       -31.312 -27.746 0.844   0.847   0.000
3       -31.116 -26.617 -0.077  1.293   0.000

== 1d3h.rmcs ==
1       -33.697 -22.345 -1.669  2.081   0.000
2       -33.691 -22.268 -1.752  2.059   0.000
3       -33.684 -22.191 -1.801  2.055   0.000

== 1koc.rmcs ==
1       -36.951 -37.164 -0.751  2.549   0.000
2       -36.562 -37.267 -0.341  2.452   0.000
3       -36.538 -37.317 -0.339  2.393   0.000

== 1lna.rmcs ==
1       -23.888 -24.112 0.224   1.255   0.000
2       -23.765 -23.956 0.191   1.114   0.000
3       -23.720 -23.496 -0.224  1.142   0.000

== 1mld.rmcs ==
1       -28.396 -28.905 0.957   1.697   0.000
2       -28.215 -28.151 0.410   1.246   0.000
3       -28.124 -28.232 0.436   1.508   0.000
```

```
== 1mts.rmcs ==
1       -38.309 -34.850 -0.727  1.313   0.000
2       -38.162 -35.351 -0.395  1.389   0.000
3       -37.852 -34.274 -0.869  1.157   0.000

== 1wap.rmcs ==
1       -42.289 -34.939 0.040   0.494   0.000
2       -41.818 -34.907 0.206   0.459   0.000
3       -41.769 -35.130 0.209   0.939   0.000

== 5abp.rmcs ==
1       -43.287 -38.021 0.370   0.886   0.000
2       -42.811 -37.664 0.367   0.893   0.000
3       -42.684 -37.426 0.361   0.907   0.000

== 6rnt.rmcs ==
1       -29.970 -26.189 -4.422  1.760   0.000
2       -29.368 -24.156 -5.368  1.356   0.000
3       -29.233 -24.059 -5.349  1.406   0.000
```
