# Supplementary material for: rDock: A Fast, Versatile and Open Source Program for Docking Ligands to Proteins and Nucleic Acids
Source: PLoS Comput Biol. 2014 Apr 10;10(4):e1003571. doi: 10.1371/journal.pcbi.1003571 (PMC3983074; doi:10.1371/journal.pcbi.1003571)
Supplement: Software S1 — Compressed file with the source code of the rDock software for ligand docking to Proteins and Nucleic Acids. (GZ) [file pcbi.1003571.s010.gz › rDock_2013.1_src/docs/newDocs/INSTALL.pdf]

RDock
How to install and compile

Peter Schmidtke

July 1, 2012

1 Introduction

1.1 Prerequisites

In order to be able to install RDock properly you need several pre-installed libraries or executables :

- gcc
- g++
- gettext (necessary for popt)
- popt (shipped with the RDock source code)
- cppunit (optional if you want to build the tests for RDock)

1.2 License & Warranty

This program is free software: you can redistribute it and/or modify it under the terms of the GNU General Public License as published by the Free Software Foundation, either version 3 of the License, or (at your option) any later version.
This program is distributed in the hope that it will be useful, but WITHOUT ANY WARRANTY; without even the implied warranty of MERCHANTABILITY or FITNESS FOR A PARTICULAR PURPOSE. See the GNU General Public License for more details.
You should have received a copy of the GNU General Public License along with this program. If not, see http://www.gnu.org/licenses.

1.3 Support & Contact

2 Installation

Rdock currently has several prerequisites, you have to install prior to Rdock.

2.1 Gettext

First you have to install gettext, which can be either done in different ways :

- Ubuntu : sudo apt-get install gettext
- OpenSuse : zypper install gettext
- Fedora/CentOs/Redhat : yum install gettext

In case you have another distribution or you prefer to compile gettext for your machine, you can do so. Gettext version 0.18 is shipped with the rdock source code. You can install by going to the rdock directory and triggering the following commands :

```
tar xzf gettext-0.18.tar.gz
cd gettext-0.18
./configure;
make;
sudo make install
```

2.2 Popt

The last mandatory dependency of Rdock is popt. popt is shipped with the Rdock code and can be installed the following way :
