# Supplementary material for: rDock: A Fast, Versatile and Open Source Program for Docking Ligands to Proteins and Nucleic Acids
Source: PLoS Comput Biol. 2014 Apr 10;10(4):e1003571. doi: 10.1371/journal.pcbi.1003571 (PMC3983074; doi:10.1371/journal.pcbi.1003571)
Supplement: Software S1 — Compressed file with the source code of the rDock software for ligand docking to Proteins and Nucleic Acids. (GZ) [file pcbi.1003571.s010.gz › rDock_2013.1_src/docs/rDock_getting_started.html]

Getting Started with rDock

# Getting Started with rDock

### Dr. David Morley

Enspiral Discovery Ltd.

`<d.morley@enspiral-discovery.com>`

Copyright © 2006 Vernalis

| **Revision History** | |
| --- | --- |
| Revision 2006.1 release | 20/09/2006 |
| Revision Initial draft | 16/03/2006 |

---

**Table of Contents**

Overview

1. Prerequisites

2. Unpacking the distribution files

3. Building rDock

4. Running a short validation experiment

**List of Tables**

1.1. Required packages for building and running rDock

2.1. rDock distribution files

3.1. Standard `tmake` build targets provided

4.1. Complexes included in the `cpu_benchmark` experiment

4.2. rDock score components output as SD data fields

## Overview

rDock is a high-throughput molecular docking platform for protein and RNA targets.
Under development at Vernalis (formerly RiboTargets) since 1998, the software (formerly
known as RiboDock^[1]^), scoring functions, and search protocols have been refined continuously to
meet the demands of in-house Structure-Based Drug Discovery (SBDD) projects.

The major components of the platform now include fast intermolecular scoring
functions (van der Waals, polar, desolvation) validated against protein and RNA targets,
a Genetic Algorithm-based stochastic search engine, a wide variety of external
SBDD-derived restraint terms (tethered template, pharmacophore, noe distance
restraints), and novel Genetic Programming-based post-docking filtering. A variety of
scripts are provided to perform automated validation experiments and to launch virtual
screening campaigns.

This introductory guide is aimed at new users of rDock. It describes the minimal
set of steps required to build rDock from the source code distribution, and to run one
of the automated validation experiments provided in the test suite distribution. The
instructions assume that you are comfortable with simple Linux command line
administration tasks, and with building Linux applications from make files. Once you are
familiar with these steps you should proceed to the User and Reference Guide for more
detailed documentation on the usage of rDock

---

^[1]^ Validation of an empirical RNA-ligand scoring function for fast flexible
docking using RiboDock, SD Morley and M Afshar, J. Comput.-Aided Mol. Des., 18
(2004) 189-208.

## Chapter 1. Prerequisites

**Compilers.** rDock is supplied as source code, which means that you will have to compile the
binary files (run-time libraries and executable programs) before you can use them.
rDock has been developed largely on the Linux operating system, most recently with
the GNU g++ compiler under SuSE 9.2 Pro. The code will almost certainly compile and
run under other Linux distributions with little or no modification, but no other
distributions or compilers have been tested extensively to date.

**Condor.** Although the rDock executables can be run directly from the command line, the
validation experiment scripts use the Condor High
Throughput Computing software for automating the process of distributing and
managing individual docking jobs across a cluster of compute machines. Condor is
available free of charge for a wide variety of platforms^[2]^. Installing and configuring Condor is beyond the scope of this guide as
the Condor configuration is very site-dependent.

For full production use, you would typically compile rDock on a separate build
machine and run the docking calculations on a cluster of compute machines. However, for
the purposes of getting started, these instructions assume that you will be compiling
rDock and running the initial validation experiments on the same machine, and that you
have installed and configured a *Personal* Condor to manage the jobs
on that machine.

**Required packages.** Make sure you have the following packages installed on your machine before you
continue. The versions listed are appropriate for SuSE 9.2 Pro; other versions may
be required for your particular Linux distribution.

**Table 1.1. Required packages for building and running rDock**

| Package | Description | Required at | Version |
| --- | --- | --- | --- |
| gcc | GNU C compiler | Compile-time | 3.3.4 |
| g++ | GNU C++ compiler | Compile-time | 3.3.4 |
| popt-devel | C++ command-line argument processing | Compile-time | 1.7-190 |
| cppunit | C++ unit test framework | Compile-time | 1.10.2 |
| popt | C++ command-line argument processing | Run-time | 1.7-190 |
| Condor | Distributed resource management | Run-time | 6.6.10 |

---

^[2]^ Condor is available from the University of Wisconsin
(`http://www.cs.wisc.edu/condor`)

## Chapter 2. Unpacking the distribution files

The rDock source files and test suite files are provided as independent gzipped
tar (.tgz) distributions. Depending on your requirements, the two distributions can be
unpacked to entirely separate locations, or can be unpacked under the same location. In
this example they are unpacked under the same location.

**Table 2.1. rDock distribution files**

| File | Description |
| --- | --- |
| rdock\_[CODELINE]\_[BUILDNUM]\_src.tgz | rDock source distribution |
| RBT\_TEST\_[DATE].tgz | Test suite data files and scripts |

where [CODELINE], [BUILDNUM], and [DATE] will vary depending on the release.
[CODELINE] represents the major version string (for example, `2006.1`),
[BUILDNUM] represents the minor version number (for example, `865`) and
[DATE] represents a date string (for example, `20060313`).

**Procedure 2.1. Example unpacking procedure**

1. **Create a new directory for building rDock.**

   ```
   $ mkdir ~/dev
   ```

   The directory you created is referred to as [BUILDDIR] in the subsequents
   steps.
2. **Copy or download the distribution files to [BUILDDIR].**

   ```
   $ cp ~/mydownloads/rdock_2006.1_865_src.tgz ~/dev/
   $ cp ~/mydownloads/RBT_TEST_20060313.tgz ~/dev/
   ```
3. **Extract the distributions.**

   ```
   $ cd ~/dev/
    $ tar xvzf rdock_2006.1_865_src.tgz
    $ tar xvzf RBT_TEST_20060313.tgz
   ```

   The distributions contain files with relative path names, and you should
   find the following subdirectories created under [BUILDDIR]. Note that the
   `./rdock/2006.1` subdirectory may have a different name
   depending on the major version string (see above).

   ```
   $ find . -type d
   .
   ./rdock
   ./rdock/2006.1
   ./rdock/2006.1/fw
   ./rdock/2006.1/bin
   ./rdock/2006.1/lib
   ./rdock/2006.1/src
   ./rdock/2006.1/src/GP
   ./rdock/2006.1/src/exe
   ./rdock/2006.1/src/lib
   ./rdock/2006.1/src/daylight
   ./rdock/2006.1/data
   ./rdock/2006.1/data/sf
   ./rdock/2006.1/data/pmf
   ./rdock/2006.1/data/pmf/smoothed
   ./rdock/2006.1/data/scripts
   ./rdock/2006.1/data/filters
   ./rdock/2006.1/docs
   ./rdock/2006.1/docs/images
   ./rdock/2006.1/build
   ./rdock/2006.1/build/test
   ./rdock/2006.1/build/test/RBT_HOME
   ./rdock/2006.1/build/tmakelib
   ./rdock/2006.1/build/tmakelib/unix
   ./rdock/2006.1/build/tmakelib/linux-pathCC-64
   ./rdock/2006.1/build/tmakelib/linux-g++-64
   ./rdock/2006.1/build/tmakelib/linux-g++
   ./rdock/2006.1/import
   ./rdock/2006.1/import/nr
   ./rdock/2006.1/import/nr/src
   ./rdock/2006.1/import/nr/include
   ./rdock/2006.1/import/simplex
   ./rdock/2006.1/import/simplex/src
   ./rdock/2006.1/import/simplex/include
   ./rdock/2006.1/include
   ./rdock/2006.1/include/GP
   ./RBT_TEST
   ./RBT_TEST/na
   ./RBT_TEST/bin
   ./RBT_TEST/ccdc_astex
   ```
4. **Make a note of the following locations for later use.**

   The test suite root directory is `[BUILDDIR]/RBT_TEST/`
   and will be referred to as `[RBT_TEST]` in later instructions.
   In this example, `[RBT_TEST]` is
   `~/dev/RBT_TEST/`.

   The rDock root directory is `[BUILDDIR]/rdock/[CODELINE]`
   and will be referred to as `[RBT_ROOT]` in later instructions.
   In this example, `[RBT_ROOT]` is
   `~/dev/rdock/2006.1/`.

## Chapter 3. Building rDock

rDock is written in C++ (with a small amount of C code from Numerical Recipes) and
makes heavy use of the C++ Standard Template Library (STL). The majority of the source
code is compiled into a single shared library (`libRbt.so`). The
executable programs themselves are relatively light-weight command-line applications
linked with `libRbt.so`.

The tmake build system (from Trolltech) is used to
generate makefiles automatically for a particular build target (i.e. combination of
operating system and compiler). The source distribution comes with
tmake templates defining the compiler options and flags for
three Linux build targets (`linux-g++`,
`linux-g++-64`, and `linux-pathCC-64`). The build
targets have been tested under SuSE 9.2 (2.6.8-24.18 kernel) with GNU g++ 3.3.4 and
PathScale pathCC 2.1.

**Table 3.1. Standard `tmake` build targets provided**

| Target name | Architecture | Compiler | Compiler flags (release build) |
| --- | --- | --- | --- |
| `linux-g++` | 32-bit Intel | `g++` | `-m32 -O3 -ffast-math -march=pentium3 -mcpu=pentium3` |
| `linux-g++-64` | 64-bit AMD Opteron | `g++` | `-m64 -O3 -ffast-math -march=k8 -mcpu=k8` |
| `linux-pathCC-64` | 64-bit AMD Opteron | `pathcc` | `-Ofast -ffast-math -march=opteron -mtune=opteron` |

**Customising the tmake template for a build target.** If none of the tmake templates are suitable for your machine, or if you wish to
customise the compiler options, you should first customise one of the existing
templates. The tmake template files are stored under
`[RBT_ROOT]/build/tmakelib/`. Locate and edit the
`tmake.conf` file for the build target you wish to customise.
For example, to customise the `linux-g++` build target, edit
`[RBT_ROOT]/build/tmakelib/linux-g++/tmake.conf` and localise
the values to suit your compiler.

**Procedure 3.1. rDock build procedure**

To build rDock, first go to the  `[RBT_ROOT]/build/`
directory.

```
$ cd [RBT_ROOT]/build
```

1. **Compile**

   Make one of the build targets listed below.

   ```
   $ make linux-g++
   $ make linux-g++-64
   $ make linux-pathCC-64
   ```
2. **Test**

   Run the rDock unit tests to check build integrity. If no failed tests are
   reported you should be all set.

   ```
   $ make test
   ```
3. **Install (optional)**

   You can either run rDock directly from the build location (only recommended
   for initial testing), or install the binaries and data files to a new location
   (recommended for production use). To install in a new location [INSTALLDIR],
   create a binary distribution file, copy the distribution file to [INSTALLDIR],
   and extract the files.

   ```
    $ make dist
    $ mkdir -p [INSTALLDIR]
    $ cp rdock_[CODELINE].tgz [INSTALLDIR]
    $ cd [INSTALLDIR]
    $ tar xvzf rdock_[CODELINE].tgz
   ```
4. **Cleanup (optional)**

   To remove all intermediate build files from
   `[RBT_ROOT]/build/`, leaving just the final executables (in
   `[RBT_ROOT]/bin/`) and shared libraries (in
   `[RBT_ROOT]/lib/`):

   ```
   $ make clean
   ```

   To remove the final executables and shared libraries as well, returning to
   a source-only distribution:

   ```
   $ make distclean
   ```

## Chapter 4. Running a short validation experiment

Now that you have successfully built the rDock executables you should change your
working directory away from the installed locations of [RBT\_TEST] and [RBT\_ROOT], for
example to a personal project directory. It is not recommended to run validation
experiments directly within the [RBT\_TEST] and [RBT\_ROOT] directory hierarchy.

**The cpu\_benchmark experiment.** The `cpu_benchmark` experiment consists of nine protein-ligand
complexes and two RNA-ligand complexes. It represents a small subset of the full
rDock docking accuracy validation set and is presented here as the calculations
should be short enough to run on a single workstation in a few hours. The full
experiments require access to a cluster of machines to run in a tractable length of
time. All ligands are non-covalently bound and were chosen to have a range of
rotatable bond counts (from 0 to 8)

**Table 4.1. Complexes included in the `cpu_benchmark` experiment**

| Type | PDB codes | File source |
| --- | --- | --- |
| Protein-ligand | `1c1e 5abp 1d3h 1wap 1cil 6rnt 1mld 1mts 1lna` | Files used intact from CCDC/Astex "clean" high-resolution test set |
| RNA-ligand | `1koc 1byj` | Prepared by Vernalis |

**Configuring the environment.** You need to define two environment variables to reflect the location of the test
suite [RBT\_TEST] and the rDock build you wish to test [RBT\_ROOT]. Either customise
the example `setup_validation` script (`bash`
shell) provided in the [RBT\_TEST] directory, and `source` the file,
or define the environment variables from the command line. You should also add the
[RBT\_TEST]/bin and [RBT\_ROOT]/bin directories to your path, and add [RBT\_ROOT[/lib
to your library path. For example, using the directory names used in the example
unpacking procedure:

```
$ export RBT_TEST=~/dev/RBT_TEST
$ export RBT_ROOT=~/dev/rdock/2006.1
$ export PATH=${RBT_ROOT}/bin:${RBT_TEST}/bin:${PATH}
$ export LD_LIBRARY_PATH=${RBT_ROOT}/lib:${LD_LIBRARY_PATH}
```

**Preparing the docking sites.** rDock requires a docking site file (.as suffix) to be defined for each receptor.
The .as file (also known as an active site file) defines a volume which represents
the region of the receptor into which each ligand should be docked. This process is
known as *cavity mapping* and needs be done only once for each
receptor prior to running the experiments. The cavity mapping process is quite quick
(from a few seconds to a few minutes per complex). The active site files (.as)
themselves and associated rDock output log files are generated "in situ" in
`$RBT_TEST/ccdc_astex/` and `$RBT_TEST/na/`.
Make sure you have write-access to these directories. The only files you will see in
your current working directory are the Condor .cmd and .log files. Wait until all
Condor jobs have completed before moving to the next step.

```
$ make_cavities cpu_benchmark
$ condor_submit cav_cpu_benchmark.cmd
```

**Running the experiment.** 

```
$ run_cpu_benchmark [TESTDIR]
```

where `[TESTDIR]` is
the name of the subdirectory that will be created under your present working
directory for the experiment output files. This command creates two Condor command
files and automatically submits the jobs:

`[TESTDIR]/SF3_100/cpu_benchmark.cmd`: 100 docking runs
per complex with standard scoring function (SF3)

`[TESTDIR]/SF5_100/cpu_benchmark.cmd`: 100 docking runs
per complex with solvation scoring function (SF5)

As a guide, the jobs require around 100 minutes total CPU time on an
dual-processor AMD Opteron 248 with 2GB RAM, for an average of around three seconds
per individual docking run.

**Output files.** After all Condor jobs have completed you should find the following output files
for each complex in `[TESTDIR]/SF3_100/`and
`[TESTDIR]/SF5_100`.

`s_[PDB].sd` Crystallographic reference ligand pose,
with rDock scores (score-only protocol)

`m_[PDB].sd` Reference ligand pose minimised under
rDock scoring function (minimisation protocol)

`x_[PDB].sd` 100 docked poses (dock protocol)

Each `.sd` file is accompanied by associated
`.out` and `.err` files which contain the
standard output (rDock output) and standard error for each calculation.

**Table 4.2. rDock score components output as SD data fields**

| Data field | Description |
| --- | --- |
| SCORE | Total docking score = SCORE.INTER + SCORE.INTRA + SCORE.SYSTEM + SCORE.RESTR |
| SCORE.INTER | Total receptor-ligand intermolecular score |
| SCORE.INTRA | Total ligand intramolecular score |
| SCORE.SYSTEM | Total intra-receptor, intra-solvent, and receptor-solvent score |
| SCORE.RESTR | Total external restraint penalty score |
| SCORE.INTER/INTRA/SYSTEM are weighted sums of the individual scoring function terms listed below. Note that the individual scoring function terms are output as raw values, and must be multiplied by their respective weights (not shown here) in order to reconstitute the total scores. | |
| SCORE.\*.VDW | vdW scores (truncated Tripos 6-12) |
| SCORE.\*.POLAR | Attractive polar interactions (empirical geometric function of distances/angles) |
| SCORE.\*.REPUL | Repulsive polar interactions (SF3 scoring function only) |
| SCORE.\*.SOLV | Desolvation score (weighted SASA, SF5 scoring function only) |
| SCORE.\*.DIHEDRAL | Dihedral score calculated over rotatable bonds only |

**Reporting the scores.** The rDock scores for each pose are embedded within each SD data record as listed
in Table 4.2, “rDock score components output as SD data fields”. You can use the `sdreport`
script to output the tabulated scores for each pose. For example:

```
$ sdreport -t x_1koc.sd
```

will output the top-level score components
(SCORE, SCORE.INTER, SCORE.INTRA, SCORE.RESTR - also SCORE.INTER.VDW) in
tab-delimited format. Alternatively you can explicitly list the data fields you wish
to output:

```
$ sdreport -tSCORE.INTER.POLAR,SCORE.INTER.VDW x_1koc.sd
```

**Calculating the docking accuracy (RMSD).** Use the `rmsreport` script to generate the SCORE vs RMSD data
files for further analysis, where RMSD is the Root Mean Squared Deviation between a
docked ligand pose and the crystallographic reference ligand pose (over
non-hydrogens only). Run `rmsreport` within each experiment
directory, with no
arguments.

```
$ cd [TESTDIR]/SF3_100
$ rmsreport
$ cd [TESTDIR]/SF5_100
$ rmsreport
```

rmsreport generates a `[PDB].rmcs` file and an
`rms[PDB].sd` file for each complex. `rms[PDB].sd`
is a sorted, filtered copy of x\_[PDB].sd with the RMSD value added as an additional
data field. `[PDB].rmcs` is a tabulated text file with six columns
where:

Col 1: pose number (sorted by total SCORE)

Col 2: SCORE

Col 3: SCORE.INTER

Col 4: SCORE.INTRA

Col 5: Root Mean Squared Deviation between pose and crystallographic
reference (over non-hydrogens only)

Col 6: zero (not used)

### Note

Poses that have an excessive cavity penalty (SCORE.RESTR.CAVITY > 1)
are removed from the RMSD analysis by rmsreport. A cavity penalty indicates
that the ligand pose is not entirely within the defined docking volume, and
hence the reported scores can not be trusted.

**Example results.** View the contents of each .rmcs file. For example, to look at the top three poses
for each complex:

```
$ head -3 *.rmcs
```

You should find that most of the
top-scoring poses have an RMSD less than 2A. Sample results for the SF3 scoring
function are shown below, although as rDock uses a stochastic search your results
may differ.

```
== 1byj.rmcs ==
1       -40.955 -33.472 -7.482  2.855   0.000
2       -40.063 -38.091 -1.972  1.457   0.000
3       -38.215 -35.048 -3.168  7.630   0.000

== 1c1e.rmcs ==
1       -27.945 -23.581 0.000   0.265   0.000
2       -27.945 -23.582 0.000   0.264   0.000
3       -27.943 -23.578 0.000   0.263   0.000

== 1cil.rmcs ==
1       -32.052 -28.150 0.517   0.736   0.000
2       -31.312 -27.746 0.844   0.847   0.000
3       -31.116 -26.617 -0.077  1.293   0.000

== 1d3h.rmcs ==
1       -33.697 -22.345 -1.669  2.081   0.000
2       -33.691 -22.268 -1.752  2.059   0.000
3       -33.684 -22.191 -1.801  2.055   0.000

== 1koc.rmcs ==
1       -36.951 -37.164 -0.751  2.549   0.000
2       -36.562 -37.267 -0.341  2.452   0.000
3       -36.538 -37.317 -0.339  2.393   0.000

== 1lna.rmcs ==
1       -23.888 -24.112 0.224   1.255   0.000
2       -23.765 -23.956 0.191   1.114   0.000
3       -23.720 -23.496 -0.224  1.142   0.000

== 1mld.rmcs ==
1       -28.396 -28.905 0.957   1.697   0.000
2       -28.215 -28.151 0.410   1.246   0.000
3       -28.124 -28.232 0.436   1.508   0.000

== 1mts.rmcs ==
1       -38.309 -34.850 -0.727  1.313   0.000
2       -38.162 -35.351 -0.395  1.389   0.000
3       -37.852 -34.274 -0.869  1.157   0.000

== 1wap.rmcs ==
1       -42.289 -34.939 0.040   0.494   0.000
2       -41.818 -34.907 0.206   0.459   0.000
3       -41.769 -35.130 0.209   0.939   0.000

== 5abp.rmcs ==
1       -43.287 -38.021 0.370   0.886   0.000
2       -42.811 -37.664 0.367   0.893   0.000
3       -42.684 -37.426 0.361   0.907   0.000

== 6rnt.rmcs ==
1       -29.970 -26.189 -4.422  1.760   0.000
2       -29.368 -24.156 -5.368  1.356   0.000
3       -29.233 -24.059 -5.349  1.406   0.000
```
