# Supplementary material for: rDock: A Fast, Versatile and Open Source Program for Docking Ligands to Proteins and Nucleic Acids
Source: PLoS Comput Biol. 2014 Apr 10;10(4):e1003571. doi: 10.1371/journal.pcbi.1003571 (PMC3983074; doi:10.1371/journal.pcbi.1003571)
Supplement: Software S1 — Compressed file with the source code of the rDock software for ligand docking to Proteins and Nucleic Acids. (GZ) [file pcbi.1003571.s010.gz › rDock_2013.1_src/gettext-0.18.tar.gz!/gettext-0.18/gettext-runtime/libasprintf/autosprintf_all.html]

GNU autosprintf


# GNU autosprintf, version 1.0

## Formatted Output to Strings in C++

Bruno Haible

---

# Table of Contents

- 1 Introduction- 2 The `autosprintf` class- 3 Using `autosprintf` in own programs

---

Copyright (C) 2002-2003, 2006-2007 Free Software Foundation, Inc.

This manual is free documentation. It is dually licensed under the
GNU FDL and the GNU GPL. This means that you can redistribute this
manual under either of these two licenses, at your choice.

This manual is covered by the GNU FDL. Permission is granted to copy,
distribute and/or modify this document under the terms of the
GNU Free Documentation License (FDL), either version 1.2 of the
License, or (at your option) any later version published by the
Free Software Foundation (FSF); with no Invariant Sections, with no
Front-Cover Text, and with no Back-Cover Texts.
A copy of the license is at http://www.gnu.org/licenses/fdl.html.

This manual is covered by the GNU GPL. You can redistribute it and/or
modify it under the terms of the GNU General Public License (GPL), either
version 2 of the License, or (at your option) any later version published
by the Free Software Foundation (FSF).
A copy of the license is at http://www.gnu.org/licenses/gpl.html.

# 1 Introduction

This package makes the C formatted output routines (`fprintf` et al.)
usable in C++ programs, for use with the `<string>` strings and the
`<iostream>` streams.

It allows to write code like

```
cerr << autosprintf ("syntax error in %s:%d: %s", filename, line, errstring);
```

instead of

```
cerr << "syntax error in " << filename << ":" << line << ": " << errstring;
```

The benefits of the autosprintf syntax are:

- It reuses the standard POSIX printf facility. Easy migration from C to C++.- English sentences are kept together.- It makes internationalization possible. Internationalization requires format
      strings, because in some cases the translator needs to change the order of a
      sentence, and more generally it is easier for the translator to work with a
      single string for a sentence than with multiple string pieces.- It reduces the risk of programming errors due to forgotten state in the
        output stream (e.g. `cout << hex;` not followed by `cout << dec;`).

# 2 The `autosprintf` class

An instance of class `autosprintf` just contains a string with the
formatted output result. Such an instance is usually allocated as an
automatic storage variable, i.e. on the stack, not with `new` on the
heap.

The constructor `autosprintf (const char *format, ...)` takes a format
string and additional arguments, like the C function `printf`.

Conversions to `char *` and `std::string` are defined that return
the encapsulated string. The conversion to `char *` returns a freshly
allocated copy of the encapsulated string; it needs to be freed using
`delete[]`. The conversion to `std::string` returns a copy of
the encapsulated string, with automatic memory management.

The destructor `~autosprintf ()` destroys the encapsulated string.

An `operator <<` is provided that outputs the encapsulated string to the
given `ostream`.

# 3 Using `autosprintf` in own programs

To use the `autosprintf` class in your programs, you need to add

```
#include "autosprintf.h"
using gnu::autosprintf;
```

to your source code.
The include file defines the class `autosprintf`, in a namespace called
`gnu`. The `‘using’` statement makes it possible to use the class
without the (otherwise natural) `gnu::` prefix.

When linking your program, you need to link with `libasprintf`, because
that's where the class is defined. In projects using GNU `autoconf`,
this means adding `‘AC_LIB_LINKFLAGS([asprintf])’` to `configure.in`
or `configure.ac`, and using the @LIBASPRINTF@ Makefile variable that
it provides.

---

This document was generated on 1 September 2007 using the
texi2html
translator version 1.52b.
