# Supplementary material for: rDock: A Fast, Versatile and Open Source Program for Docking Ligands to Proteins and Nucleic Acids
Source: PLoS Comput Biol. 2014 Apr 10;10(4):e1003571. doi: 10.1371/journal.pcbi.1003571 (PMC3983074; doi:10.1371/journal.pcbi.1003571)
Supplement: Software S1 — Compressed file with the source code of the rDock software for ligand docking to Proteins and Nucleic Acids. (GZ) [file pcbi.1003571.s010.gz › rDock_2013.1_src/gettext-0.18.tar.gz!/gettext-0.18/gettext-runtime/man/ngettext.3.html]

NGETTEXT


# NGETTEXT

NAME  
SYNOPSIS  
DESCRIPTION  
RETURN VALUE  
ERRORS  
BUGS  
SEE ALSO  


---


## NAME

|  |  |
| --- | --- |
|  | ngettext, dngettext, dcngettext − translate message and choose plural form |

## SYNOPSIS

|  |  |
| --- | --- |
|  | ``` #include <libintl.h>  char * ngettext (const char * msgid, const char * msgid_plural,                  unsigned long int n); char * dngettext (const char * domainname,                   const char * msgid, const char * msgid_plural,                   unsigned long int n); char * dcngettext (const char * domainname,                    const char * msgid, const char * msgid_plural,                    unsigned long int n, int category); ``` |

## DESCRIPTION

|  |  |
| --- | --- |
|  | The **ngettext**, **dngettext** and **dcngettext** functions attempt to translate a text string into the user’s native language, by looking up the appropriate plural form of the translation in a message catalog.  Plural forms are grammatical variants depending on the a number. Some languages have two forms, called singular and plural. Other languages have three forms, called singular, dual and plural. There are also languages with four forms.  The **ngettext**, **dngettext** and **dcngettext** functions work like the **gettext**, **dgettext** and **dcgettext** functions, respectively. Additionally, they choose the appropriate plural form, which depends on the number *n* and the language of the message catalog where the translation was found.  In the "C" locale, or if none of the used catalogs contain a translation for *msgid*, the **ngettext**, **dngettext** and **dcngettext** functions return *msgid* if *n* == 1, or *msgid\_plural* if *n* != 1. |

## RETURN VALUE

|  |  |
| --- | --- |
|  | If a translation was found in one of the specified catalogs, the appropriate plural form is converted to the locale’s codeset and returned. The resulting string is statically allocated and must not be modified or freed. Otherwise *msgid* or *msgid\_plural* is returned, as described above. |

## ERRORS

|  |  |
| --- | --- |
|  | **errno** is not modified. |

## BUGS

|  |  |
| --- | --- |
|  | The return type ought to be **const char \***, but is **char \*** to avoid warnings in C code predating ANSI C. |

## SEE ALSO

|  |  |
| --- | --- |
|  | **gettext**(3), **dgettext**(3), **dcgettext**(3) |

---
