# Supplementary material for: rDock: A Fast, Versatile and Open Source Program for Docking Ligands to Proteins and Nucleic Acids
Source: PLoS Comput Biol. 2014 Apr 10;10(4):e1003571. doi: 10.1371/journal.pcbi.1003571 (PMC3983074; doi:10.1371/journal.pcbi.1003571)
Supplement: Software S1 — Compressed file with the source code of the rDock software for ligand docking to Proteins and Nucleic Acids. (GZ) [file pcbi.1003571.s010.gz › rDock_2013.1_src/gettext-0.18.tar.gz!/gettext-0.18/gettext-runtime/man/bind_textdomain_codeset.3.html]

BIND\_TEXTDOMAIN\_CODESET


# BIND\_TEXTDOMAIN\_CODESET

NAME  
SYNOPSIS  
DESCRIPTION  
RETURN VALUE  
ERRORS  
BUGS  
SEE ALSO  


---


## NAME

|  |  |
| --- | --- |
|  | bind\_textdomain\_codeset − set encoding of message translations |

## SYNOPSIS

|  |  |
| --- | --- |
|  | ``` #include <libintl.h>  char * bind_textdomain_codeset (const char * domainname,                                 const char * codeset); ``` |

## DESCRIPTION

|  |  |
| --- | --- |
|  | The **bind\_textdomain\_codeset** function sets the output codeset for message catalogs for domain *domainname*.  A message domain is a set of translatable *msgid* messages. Usually, every software package has its own message domain.  By default, the **gettext** family of functions returns translated messages in the locale’s character encoding, which can be retrieved as **nl\_langinfo(CODESET)**. The need for calling **bind\_textdomain\_codeset** arises for programs which store strings in a locale independent way (e.g. UTF-8) and want to avoid an extra character set conversion on the returned translated messages.  *domainname* must be a non-empty string.  If *codeset* is not NULL, it must be a valid encoding name which can be used for the **iconv\_open** function. The **bind\_textdomain\_codeset** function sets the output codeset for message catalogs belonging to domain *domainname* to *codeset*. The function makes copies of the argument strings as needed.  If *codeset* is NULL, the function returns the previously set codeset for domain *domainname*. The default is NULL, denoting the locale’s character encoding. |

## RETURN VALUE

|  |  |
| --- | --- |
|  | If successful, the **bind\_textdomain\_codeset** function returns the current codeset for domain *domainname*, after possibly changing it. The resulting string is valid until the next **bind\_textdomain\_codeset** call for the same *domainname* and must not be modified or freed. If a memory allocation failure occurs, it sets **errno** to **ENOMEM** and returns NULL. If no codeset has been set for domain *domainname*, it returns NULL. |

## ERRORS

|  |  |
| --- | --- |
|  | The following error can occur, among others: |

|  |  |  |  |  |
| --- | --- | --- | --- | --- |
|  | **ENOMEM** |  | Not enough memory available. |  |

## BUGS

|  |  |
| --- | --- |
|  | The return type ought to be **const char \***, but is **char \*** to avoid warnings in C code predating ANSI C. |

## SEE ALSO

|  |  |
| --- | --- |
|  | **gettext**(3), **dgettext**(3), **dcgettext**(3), **ngettext**(3), **dngettext**(3), **dcngettext**(3), **textdomain**(3), **nl\_langinfo**(3), **iconv\_open**(3) |

---
