# Supplementary material for: rDock: A Fast, Versatile and Open Source Program for Docking Ligands to Proteins and Nucleic Acids
Source: PLoS Comput Biol. 2014 Apr 10;10(4):e1003571. doi: 10.1371/journal.pcbi.1003571 (PMC3983074; doi:10.1371/journal.pcbi.1003571)
Supplement: Software S1 — Compressed file with the source code of the rDock software for ligand docking to Proteins and Nucleic Acids. (GZ) [file pcbi.1003571.s010.gz › rDock_2013.1_src/gettext-0.18.tar.gz!/gettext-0.18/gettext-runtime/man/envsubst.1.html]

ENVSUBST


# ENVSUBST

NAME  
SYNOPSIS  
DESCRIPTION  
AUTHOR  
REPORTING BUGS  
COPYRIGHT  
SEE ALSO  


---


## NAME

|  |  |
| --- | --- |
|  | envsubst − substitutes environment variables in shell format strings |

## SYNOPSIS

|  |  |
| --- | --- |
|  | **envsubst** [*OPTION*] [*SHELL-FORMAT*] |

## DESCRIPTION

|  |  |
| --- | --- |
|  | Substitutes the values of environment variables. |

|  |  |
| --- | --- |
|  | **Operation mode:** |

|  |  |
| --- | --- |
|  | **−v**, **−−variables** |

|  |  |
| --- | --- |
|  | output the variables occurring in SHELL-FORMAT |

|  |  |
| --- | --- |
|  | **Informative output:** |

|  |  |
| --- | --- |
|  | **−h**, **−−help** |

|  |  |
| --- | --- |
|  | display this help and exit |

|  |  |
| --- | --- |
|  | **−V**, **−−version** |

|  |  |
| --- | --- |
|  | output version information and exit |

|  |  |
| --- | --- |
|  | In normal operation mode, standard input is copied to standard output, with references to environment variables of the form $VARIABLE or ${VARIABLE} being replaced with the corresponding values. If a SHELL-FORMAT is given, only those environment variables that are referenced in SHELL-FORMAT are substituted; otherwise all environment variables references occurring in standard input are substituted.  When **−−variables** is used, standard input is ignored, and the output consists of the environment variables that are referenced in SHELL-FORMAT, one per line. |

## AUTHOR

|  |  |
| --- | --- |
|  | Written by Bruno Haible. |

## REPORTING BUGS

|  |  |
| --- | --- |
|  | Report bugs to <bug-gnu-gettext@gnu.org>. |

## COPYRIGHT

|  |  |
| --- | --- |
|  | Copyright © 2003-2007 Free Software Foundation, Inc. License GPLv3+: GNU GPL version 3 or later <http://gnu.org/licenses/gpl.html>  This is free software: you are free to change and redistribute it. There is NO WARRANTY, to the extent permitted by law. |

## SEE ALSO

|  |  |
| --- | --- |
|  | The full documentation for **envsubst** is maintained as a Texinfo manual. If the **info** and **envsubst** programs are properly installed at your site, the command |

|  |  |
| --- | --- |
|  | **info envsubst** |

|  |  |
| --- | --- |
|  | should give you access to the complete manual. |

---
