# Supplementary material for: rDock: A Fast, Versatile and Open Source Program for Docking Ligands to Proteins and Nucleic Acids
Source: PLoS Comput Biol. 2014 Apr 10;10(4):e1003571. doi: 10.1371/journal.pcbi.1003571 (PMC3983074; doi:10.1371/journal.pcbi.1003571)
Supplement: Software S1 — Compressed file with the source code of the rDock software for ligand docking to Proteins and Nucleic Acids. (GZ) [file pcbi.1003571.s010.gz › rDock_2013.1_src/gettext-0.18.tar.gz!/gettext-0.18/gettext-runtime/man/gettext.3.html]

GETTEXT


# GETTEXT

NAME  
SYNOPSIS  
DESCRIPTION  
RETURN VALUE  
ERRORS  
BUGS  
SEE ALSO  


---


## NAME

|  |  |
| --- | --- |
|  | gettext, dgettext, dcgettext − translate message |

## SYNOPSIS

|  |  |
| --- | --- |
|  | ``` #include <libintl.h>  char * gettext (const char * msgid); char * dgettext (const char * domainname, const char * msgid); char * dcgettext (const char * domainname, const char * msgid,                   int category); ``` |

## DESCRIPTION

|  |  |
| --- | --- |
|  | The **gettext**, **dgettext** and **dcgettext** functions attempt to translate a text string into the user’s native language, by looking up the translation in a message catalog.  The *msgid* argument identifies the message to be translated. By convention, it is the English version of the message, with non-ASCII characters replaced by ASCII approximations. This choice allows the translators to work with message catalogs, called PO files, that contain both the English and the translated versions of each message, and can be installed using the **msgfmt** utility.  A message domain is a set of translatable *msgid* messages. Usually, every software package has its own message domain. The domain name is used to determine the message catalog where the translation is looked up; it must be a non-empty string. For the **gettext** function, it is specified through a preceding **textdomain** call. For the **dgettext** and **dcgettext** functions, it is passed as the *domainname* argument; if this argument is NULL, the domain name specified through a preceding **textdomain** call is used instead.  Translation lookup operates in the context of the current locale. For the **gettext** and **dgettext** functions, the **LC\_MESSAGES** locale facet is used. It is determined by a preceding call to the **setlocale** function. **setlocale(LC\_ALL,"")** initializes the **LC\_MESSAGES** locale based on the first nonempty value of the three environment variables **LC\_ALL**, **LC\_MESSAGES**, **LANG**; see **setlocale**(3). For the **dcgettext** function, the locale facet is determined by the *category* argument, which should be one of the **LC\_xxx** constants defined in the <locale.h> header, excluding **LC\_ALL**. In both cases, the functions also use the **LC\_CTYPE** locale facet in order to convert the translated message from the translator’s codeset to the current locale’s codeset, unless overridden by a prior call to the **bind\_textdomain\_codeset** function.  The message catalog used by the functions is at the pathname *dirname*/*locale*/*category*/*domainname*.mo. Here *dirname* is the directory specified through **bindtextdomain**. Its default is system and configuration dependent; typically it is *prefix*/share/locale, where *prefix* is the installation prefix of the package. *locale* is the name of the current locale facet; the GNU implementation also tries generalizations, such as the language name without the territory name. *category* is **LC\_MESSAGES** for the **gettext** and **dgettext** functions, or the argument passed to the **dcgettext** function.  If the **LANGUAGE** environment variable is set to a nonempty value, and the locale is not the "C" locale, the value of **LANGUAGE** is assumed to contain a colon separated list of locale names. The functions will attempt to look up a translation of *msgid* in each of the locales in turn. This is a GNU extension.  In the "C" locale, or if none of the used catalogs contain a translation for *msgid*, the **gettext**, **dgettext** and **dcgettext** functions return *msgid*. |

## RETURN VALUE

|  |  |
| --- | --- |
|  | If a translation was found in one of the specified catalogs, it is converted to the locale’s codeset and returned. The resulting string is statically allocated and must not be modified or freed. Otherwise *msgid* is returned. |

## ERRORS

|  |  |
| --- | --- |
|  | **errno** is not modified. |

## BUGS

|  |  |
| --- | --- |
|  | The return type ought to be **const char \***, but is **char \*** to avoid warnings in C code predating ANSI C.  When an empty string is used for *msgid*, the functions may return a nonempty string. |

## SEE ALSO

|  |  |
| --- | --- |
|  | **ngettext**(3), **dngettext**(3), **dcngettext**(3), **setlocale**(3), **textdomain**(3), **bindtextdomain**(3), **bind\_textdomain\_codeset**(3), **msgfmt**(1) |

---
