# Supplementary material for: rDock: A Fast, Versatile and Open Source Program for Docking Ligands to Proteins and Nucleic Acids
Source: PLoS Comput Biol. 2014 Apr 10;10(4):e1003571. doi: 10.1371/journal.pcbi.1003571 (PMC3983074; doi:10.1371/journal.pcbi.1003571)
Supplement: Software S1 — Compressed file with the source code of the rDock software for ligand docking to Proteins and Nucleic Acids. (GZ) [file pcbi.1003571.s010.gz › rDock_2013.1_src/gettext-0.18.tar.gz!/gettext-0.18/gettext-runtime/man/bindtextdomain.3.html]

BINDTEXTDOMAIN


# BINDTEXTDOMAIN

NAME  
SYNOPSIS  
DESCRIPTION  
RETURN VALUE  
ERRORS  
BUGS  
SEE ALSO  


---


## NAME

|  |  |
| --- | --- |
|  | bindtextdomain − set directory containing message catalogs |

## SYNOPSIS

|  |  |
| --- | --- |
|  | ``` #include <libintl.h>  char * bindtextdomain (const char * domainname, const char * dirname); ``` |

## DESCRIPTION

|  |  |
| --- | --- |
|  | The **bindtextdomain** function sets the base directory of the hierarchy containing message catalogs for a given message domain.  A message domain is a set of translatable *msgid* messages. Usually, every software package has its own message domain. The need for calling **bindtextdomain** arises because packages are not always installed with the same prefix as the <libintl.h> header and the libc/libintl libraries.  Message catalogs will be expected at the pathnames *dirname*/*locale*/*category*/*domainname*.mo, where *locale* is a locale name and *category* is a locale facet such as **LC\_MESSAGES**.  *domainname* must be a non-empty string.  If *dirname* is not NULL, the base directory for message catalogs belonging to domain *domainname* is set to *dirname*. The function makes copies of the argument strings as needed. If the program wishes to call the **chdir** function, it is important that *dirname* be an absolute pathname; otherwise it cannot be guaranteed that the message catalogs will be found.  If *dirname* is NULL, the function returns the previously set base directory for domain *domainname*. |

## RETURN VALUE

|  |  |
| --- | --- |
|  | If successful, the **bindtextdomain** function returns the current base directory for domain *domainname*, after possibly changing it. The resulting string is valid until the next **bindtextdomain** call for the same *domainname* and must not be modified or freed. If a memory allocation failure occurs, it sets **errno** to **ENOMEM** and returns NULL. |

## ERRORS

|  |  |
| --- | --- |
|  | The following error can occur, among others: |

|  |  |  |  |  |
| --- | --- | --- | --- | --- |
|  | **ENOMEM** |  | Not enough memory available. |  |

## BUGS

|  |  |
| --- | --- |
|  | The return type ought to be **const char \***, but is **char \*** to avoid warnings in C code predating ANSI C. |

## SEE ALSO

|  |  |
| --- | --- |
|  | **gettext**(3), **dgettext**(3), **dcgettext**(3), **ngettext**(3), **dngettext**(3), **dcngettext**(3), **textdomain**(3), **realpath**(3) |

---
