# Supplementary material for: rDock: A Fast, Versatile and Open Source Program for Docking Ligands to Proteins and Nucleic Acids
Source: PLoS Comput Biol. 2014 Apr 10;10(4):e1003571. doi: 10.1371/journal.pcbi.1003571 (PMC3983074; doi:10.1371/journal.pcbi.1003571)
Supplement: Software S1 — Compressed file with the source code of the rDock software for ligand docking to Proteins and Nucleic Acids. (GZ) [file pcbi.1003571.s010.gz › rDock_2013.1_src/gettext-0.18.tar.gz!/gettext-0.18/gettext-runtime/man/textdomain.3.html]

TEXTDOMAIN


# TEXTDOMAIN

NAME  
SYNOPSIS  
DESCRIPTION  
RETURN VALUE  
ERRORS  
BUGS  
SEE ALSO  


---


## NAME

|  |  |
| --- | --- |
|  | textdomain − set domain for future gettext() calls |

## SYNOPSIS

|  |  |
| --- | --- |
|  | ``` #include <libintl.h>  char * textdomain (const char * domainname); ``` |

## DESCRIPTION

|  |  |
| --- | --- |
|  | The **textdomain** function sets or retrieves the current message domain.  A message domain is a set of translatable *msgid* messages. Usually, every software package has its own message domain. The domain name is used to determine the message catalog where a translation is looked up; it must be a non-empty string.  The current message domain is used by the **gettext**, **ngettext** functions, and by the **dgettext**, **dcgettext**, **dngettext** and **dcngettext** functions when called with a NULL domainname argument.  If *domainname* is not NULL, the current message domain is set to *domainname*. The string the function stores internally is a copy of the *domainname* argument.  If *domainname* is NULL, the function returns the current message domain. |

## RETURN VALUE

|  |  |
| --- | --- |
|  | If successful, the **textdomain** function returns the current message domain, after possibly changing it. The resulting string is valid until the next **textdomain** call and must not be modified or freed. If a memory allocation failure occurs, it sets **errno** to **ENOMEM** and returns NULL. |

## ERRORS

|  |  |
| --- | --- |
|  | The following error can occur, among others: |

|  |  |  |  |  |
| --- | --- | --- | --- | --- |
|  | **ENOMEM** |  | Not enough memory available. |  |

## BUGS

|  |  |
| --- | --- |
|  | The return type ought to be **const char \***, but is **char \*** to avoid warnings in C code predating ANSI C. |

## SEE ALSO

|  |  |
| --- | --- |
|  | **gettext**(3), **ngettext**(3), **bindtextdomain**(3), **bind\_textdomain\_codeset**(3) |

---
