# Supplementary material for: rDock: A Fast, Versatile and Open Source Program for Docking Ligands to Proteins and Nucleic Acids
Source: PLoS Comput Biol. 2014 Apr 10;10(4):e1003571. doi: 10.1371/journal.pcbi.1003571 (PMC3983074; doi:10.1371/journal.pcbi.1003571)
Supplement: Software S1 — Compressed file with the source code of the rDock software for ligand docking to Proteins and Nucleic Acids. (GZ) [file pcbi.1003571.s010.gz › rDock_2013.1_src/gettext-0.18.tar.gz!/gettext-0.18/gettext-runtime/intl-csharp/csharpdoc/GNU_Gettext_GettextResourceSet.html]

GNU.Gettext.GettextResourceSet Class


### GNU.Gettext.GettextResourceSet Class

> ```
> public class GettextResourceSet: System.Resources.ResourceSet
> ```

#### Base Types

> System.Resources.ResourceSet  
>   GettextResourceSet

#### Library

> GNU.Gettext

#### Summary

> Each instance of this class encapsulates a single PO file.
>
> This API of this class is not meant to be used directly; use
> `GettextResourceManager` instead.

#### See Also

> GNU.Gettext Namespace

#### Members

> GettextResourceSet Constructors
>
> GettextResourceSet() Constructor  
> GettextResourceSet(System.Resources.IResourceReader) Constructor  
> GettextResourceSet(System.IO.Stream) Constructor  
> GettextResourceSet(System.String) Constructor  
>
> GettextResourceSet Methods
>
> GettextResourceSet.GetPluralString Method  
> GettextResourceSet.GetString(System.String) Method  
> GettextResourceSet.GetString(System.String, bool) Method  
> GettextResourceSet.PluralEval Method  
>
> GettextResourceSet Properties
>
> GettextResourceSet.Keys Property

---


### GettextResourceSet() Constructor

> ```
> protected GettextResourceSet();
> ```

#### Summary

> Creates a new message catalog. When using this constructor, you
> must override the `ReadResources` method, in order to initialize
> the `Table` property. The message catalog will support plural
> forms only if the `ReadResources` method installs values of type
> `String[]` and if the `PluralEval` method is overridden.

#### See Also

> GNU.Gettext.GettextResourceSet Class, GNU.Gettext Namespace

---


### GettextResourceSet(System.Resources.IResourceReader) Constructor

> ```
> public GettextResourceSet(System.Resources.IResourceReader reader);
> ```

#### Summary

> Creates a new message catalog, by reading the string/value pairs from
> the given *reader*. The message catalog will support
> plural forms only if the reader can produce values of type
> `String[]` and if the `PluralEval` method is overridden.

#### See Also

> GNU.Gettext.GettextResourceSet Class, GNU.Gettext Namespace

---


### GettextResourceSet(System.IO.Stream) Constructor

> ```
> public GettextResourceSet(System.IO.Stream stream);
> ```

#### Summary

> Creates a new message catalog, by reading the string/value pairs from
> the given *stream*, which should have the format of
> a `.resources` file. The message catalog will not support plural
> forms.

#### See Also

> GNU.Gettext.GettextResourceSet Class, GNU.Gettext Namespace

---


### GettextResourceSet(System.String) Constructor

> ```
> public GettextResourceSet(System.String fileName);
> ```

#### Summary

> Creates a new message catalog, by reading the string/value pairs from
> the file with the given *fileName*. The file should
> be in the format of a `.resources` file. The message catalog will
> not support plural forms.

#### See Also

> GNU.Gettext.GettextResourceSet Class, GNU.Gettext Namespace

---


### GettextResourceSet.GetPluralString Method

> ```
> public virtual System.String GetPluralString(System.String msgid, System.String msgidPlural, long n);
> ```

#### Summary

> Returns the translation of *msgid* and
> *msgidPlural*, choosing the right plural form
> depending on the number *n*.

#### Parameters

> msgid
> :   the key string to be translated, an ASCII
>     string
>
> msgidPlural
> :   the English plural of *msgid*,
>     an ASCII string
>
> n
> :   the number, should be >= 0

#### Return Value

> the translation, or `null` if none is found

#### See Also

> GNU.Gettext.GettextResourceSet Class, GNU.Gettext Namespace

---


### GettextResourceSet.GetString(System.String) Method

> ```
> public override System.String GetString(System.String msgid);
> ```

#### Summary

> Returns the translation of *msgid*.

#### Parameters

> msgid
> :   the key string to be translated, an ASCII
>     string

#### Return Value

> the translation of *msgid*, or `null` if
> none is found

#### See Also

> GNU.Gettext.GettextResourceSet Class, GNU.Gettext Namespace

---


### GettextResourceSet.GetString(System.String, bool) Method

> ```
> public override System.String GetString(System.String msgid, bool ignoreCase);
> ```

#### Summary

> Returns the translation of *msgid*, with possibly
> case-insensitive lookup.

#### Parameters

> msgid
> :   the key string to be translated, an ASCII
>     string

#### Return Value

> the translation of *msgid*, or `null` if
> none is found

#### See Also

> GNU.Gettext.GettextResourceSet Class, GNU.Gettext Namespace

---


### GettextResourceSet.PluralEval Method

> ```
> protected virtual long PluralEval(long n);
> ```

#### Summary

> Returns the index of the plural form to be chosen for a given number.
> The default implementation is the Germanic plural formula:
> zero for *n* == 1, one for *n* != 1.

#### See Also

> GNU.Gettext.GettextResourceSet Class, GNU.Gettext Namespace

---


### GettextResourceSet.Keys Property

> ```
> public virtual System.Collections.ICollection Keys { get; }
> ```

#### Summary

> Returns the keys of this resource set, i.e. the strings for which
> `GetObject()` can return a non-null value.

#### See Also

> GNU.Gettext.GettextResourceSet Class, GNU.Gettext Namespace
