# Supplementary material for: rDock: A Fast, Versatile and Open Source Program for Docking Ligands to Proteins and Nucleic Acids
Source: PLoS Comput Biol. 2014 Apr 10;10(4):e1003571. doi: 10.1371/journal.pcbi.1003571 (PMC3983074; doi:10.1371/journal.pcbi.1003571)
Supplement: Software S1 — Compressed file with the source code of the rDock software for ligand docking to Proteins and Nucleic Acids. (GZ) [file pcbi.1003571.s010.gz › rDock_2013.1_src/gettext-0.18.tar.gz!/gettext-0.18/gettext-runtime/intl-csharp/csharpdoc/GNU_Gettext_GettextResourceManager.html]

GNU.Gettext.GettextResourceManager Class


### GNU.Gettext.GettextResourceManager Class

> ```
> public class GettextResourceManager: System.Resources.ResourceManager
> ```

#### Base Types

> System.Resources.ResourceManager  
>   GettextResourceManager

#### Library

> GNU.Gettext

#### Summary

> Each instance of this class can be used to lookup translations for a
> given resource name. For each `CultureInfo`, it performs the lookup
> in several assemblies, from most specific over territory-neutral to
> language-neutral.

#### See Also

> GNU.Gettext Namespace

#### Members

> GettextResourceManager Constructors
>
> GettextResourceManager(System.String) Constructor  
> GettextResourceManager(System.String, System.Reflection.Assembly) Constructor  
>
> GettextResourceManager Methods
>
> GettextResourceManager.GetPluralString(System.String, System.String, long, System.Globalization.CultureInfo) Method  
> GettextResourceManager.GetPluralString(System.String, System.String, long) Method  
> GettextResourceManager.GetString(System.String, System.Globalization.CultureInfo) Method  
> GettextResourceManager.GetString(System.String) Method

---


### GettextResourceManager(System.String) Constructor

> ```
> public GettextResourceManager(System.String baseName);
> ```

#### Summary

> Constructor.

#### Parameters

> baseName
> :   the resource name, also the assembly base
>     name

#### See Also

> GNU.Gettext.GettextResourceManager Class, GNU.Gettext Namespace

---


### GettextResourceManager(System.String, System.Reflection.Assembly) Constructor

> ```
> public GettextResourceManager(System.String baseName, System.Reflection.Assembly assembly);
> ```

#### Summary

> Constructor.

#### Parameters

> baseName
> :   the resource name, also the assembly base
>     name

#### See Also

> GNU.Gettext.GettextResourceManager Class, GNU.Gettext Namespace

---


### GettextResourceManager.GetPluralString(System.String, System.String, long, System.Globalization.CultureInfo) Method

> ```
> public virtual System.String GetPluralString(System.String msgid, System.String msgidPlural, long n, System.Globalization.CultureInfo culture);
> ```

#### Summary

> Returns the translation of *msgid* and
> *msgidPlural* in a given culture, choosing the right
> plural form depending on the number *n*.

#### Parameters

> msgid
> :   the key string to be translated, an ASCII
>     string
>
> msgidPlural
> :   the English plural of *msgid*,
>     an ASCII string
>
> n
> :   the number, should be >= 0

#### Return Value

> the translation, or *msgid* or
> *msgidPlural* if none is found

#### See Also

> GNU.Gettext.GettextResourceManager Class, GNU.Gettext Namespace

---


### GettextResourceManager.GetPluralString(System.String, System.String, long) Method

> ```
> public virtual System.String GetPluralString(System.String msgid, System.String msgidPlural, long n);
> ```

#### Summary

> Returns the translation of *msgid* and
> *msgidPlural* in the current culture, choosing the
> right plural form depending on the number *n*.

#### Parameters

> msgid
> :   the key string to be translated, an ASCII
>     string
>
> msgidPlural
> :   the English plural of *msgid*,
>     an ASCII string
>
> n
> :   the number, should be >= 0

#### Return Value

> the translation, or *msgid* or
> *msgidPlural* if none is found

#### See Also

> GNU.Gettext.GettextResourceManager Class, GNU.Gettext Namespace

---


### GettextResourceManager.GetString(System.String, System.Globalization.CultureInfo) Method

> ```
> public override System.String GetString(System.String msgid, System.Globalization.CultureInfo culture);
> ```

#### Summary

> Returns the translation of *msgid* in a given culture.

#### Parameters

> msgid
> :   the key string to be translated, an ASCII
>     string

#### Return Value

> the translation of *msgid*, or
> *msgid* if none is found

#### See Also

> GNU.Gettext.GettextResourceManager Class, GNU.Gettext Namespace

---


### GettextResourceManager.GetString(System.String) Method

> ```
> public override System.String GetString(System.String msgid);
> ```

#### Summary

> Returns the translation of *msgid* in the current
> culture.

#### Parameters

> msgid
> :   the key string to be translated, an ASCII
>     string

#### Return Value

> the translation of *msgid*, or
> *msgid* if none is found

#### See Also

> GNU.Gettext.GettextResourceManager Class, GNU.Gettext Namespace
