# Supplementary material for: rDock: A Fast, Versatile and Open Source Program for Docking Ligands to Proteins and Nucleic Acids
Source: PLoS Comput Biol. 2014 Apr 10;10(4):e1003571. doi: 10.1371/journal.pcbi.1003571 (PMC3983074; doi:10.1371/journal.pcbi.1003571)
Supplement: Software S1 — Compressed file with the source code of the rDock software for ligand docking to Proteins and Nucleic Acids. (GZ) [file pcbi.1003571.s010.gz › rDock_2013.1_src/gettext-0.18.tar.gz!/gettext-0.18/gettext-runtime/intl-java/javadoc2/index-all.html]

: Index


|  |  |  |
| --- | --- | --- |
| Class \| **Tree** \| **Deprecated** \| **Index** \| **Help** | |  |
| PREV   NEXT | **FRAMES**    **NO FRAMES** |  |

G N V 

---


## **G**

**gettext(ResourceBundle, String)** - Static method in class gnu.gettext.GettextResource: Returns the translation of msgid. **GettextResource** - class gnu.gettext.GettextResource.: This class implements the main GNU libintl functions in Java. **GettextResource()** - Constructor for class gnu.gettext.GettextResource

---


## **N**

**ngettext(ResourceBundle, String, String, long)** - Static method in class gnu.gettext.GettextResource: Returns the plural form for n of the translation of msgid.

---


## **V**

**verbose** - Static variable in class gnu.gettext.GettextResource

---

G N V


|  |  |  |
| --- | --- | --- |
| Class \| **Tree** \| **Deprecated** \| **Index** \| **Help** | |  |
| PREV   NEXT | **FRAMES**    **NO FRAMES** |  |


---
