# Supplementary material for: rDock: A Fast, Versatile and Open Source Program for Docking Ligands to Proteins and Nucleic Acids
Source: PLoS Comput Biol. 2014 Apr 10;10(4):e1003571. doi: 10.1371/journal.pcbi.1003571 (PMC3983074; doi:10.1371/journal.pcbi.1003571)
Supplement: Software S1 — Compressed file with the source code of the rDock software for ligand docking to Proteins and Nucleic Acids. (GZ) [file pcbi.1003571.s010.gz › rDock_2013.1_src/gettext-0.18.tar.gz!/gettext-0.18/gettext-runtime/intl-java/javadoc2/gnu/gettext/GettextResource.html]

: Class GettextResource


|  |  |  |
| --- | --- | --- |
| **Class** \| **Tree** \| **Deprecated** \| **Index** \| **Help** | |  |
| PREV CLASS   NEXT CLASS | **FRAMES**    **NO FRAMES** |  |
| SUMMARY:  INNER \| FIELD \| CONSTR \| METHOD | DETAIL:  FIELD \| CONSTR \| METHOD |  |


---


## gnu.gettext Class GettextResource

```
java.lang.Object
  |
  +--java.util.ResourceBundle
        |
        +--gnu.gettext.GettextResource
```

---

public abstract class **GettextResource** extends java.util.ResourceBundle

This class implements the main GNU libintl functions in Java.

Using the GNU gettext approach, compiled message catalogs are normal
Java ResourceBundle classes and are thus interoperable with standard
ResourceBundle based code.

The main differences between the Sun ResourceBundle approach and the
GNU gettext approach are:

- In the Sun approach, the keys are abstract textual shortcuts.
  In the GNU gettext approach, the keys are the English/ASCII version
  of the messages.- In the Sun approach, the translation files are called
    "Resource\_locale.properties" and have non-ASCII
    characters encoded in the Java
    `\``unnnn` syntax. Very few editors
    can natively display international characters in this format. In the
    GNU gettext approach, the translation files are called
    "Resource.locale.po"
    and are in the encoding the translator has chosen. Many editors
    can be used. There are at least three GUI translating tools
    (Emacs PO mode, KDE KBabel, GNOME gtranslator).- In the Sun approach, the function
      `ResourceBundle.getString` throws a
      `MissingResourceException` when no translation is found.
      In the GNU gettext approach, the `gettext` function
      returns the (English) message key in that case.- In the Sun approach, there is no support for plural handling.
        Even the most elaborate MessageFormat strings cannot provide decent
        plural handling. In the GNU gettext approach, we have the
        `ngettext` function.

To compile GNU gettext message catalogs into Java ResourceBundle classes,
the `msgfmt` program can be used.

---

|  |  |
| --- | --- |
| **Field Summary** | |
| `static boolean` | `verbose` |

**Fields inherited from class java.util.ResourceBundle**

`parent`


**Constructor Summary**

`GettextResource()`


|  |  |
| --- | --- |
| **Method Summary** | |
| `static java.lang.String` | `gettext(java.util.ResourceBundle catalog, java.lang.String msgid)`             Returns the translation of msgid. |
| `static java.lang.String` | `ngettext(java.util.ResourceBundle catalog, java.lang.String msgid, java.lang.String msgid_plural, long n)`             Returns the plural form for n of the translation of msgid. |

**Methods inherited from class java.util.ResourceBundle**

`getBundle, getBundle, getBundle, getKeys, getLocale, getObject, getString, getStringArray, handleGetObject, setParent`

**Methods inherited from class java.lang.Object**

`clone, equals, finalize, getClass, hashCode, notify, notifyAll, toString, wait, wait, wait`

**Field Detail**

### verbose

```
public static boolean verbose
```


**Constructor Detail**

### GettextResource

```
public GettextResource()
```


**Method Detail**

### gettext

```
public static java.lang.String gettext(java.util.ResourceBundle catalog,
                                       java.lang.String msgid)
```

:   Returns the translation of msgid.:   **Parameters:**: `catalog` - a ResourceBundle: `msgid` - the key string to be translated, an ASCII string **Returns:**: the translation of msgid, or msgid if none is found

---


### ngettext

```
public static java.lang.String ngettext(java.util.ResourceBundle catalog,
                                        java.lang.String msgid,
                                        java.lang.String msgid_plural,
                                        long n)
```

:   Returns the plural form for n of the translation of
    msgid.:   **Parameters:**: `catalog` - a ResourceBundle: `msgid` - the key string to be translated, an ASCII string: `msgid_plural` - its English plural form **Returns:**: the translation of msgid depending on n, or msgid or msgid\_plural if none is found


---


|  |  |  |
| --- | --- | --- |
| **Class** \| **Tree** \| **Deprecated** \| **Index** \| **Help** | |  |
| PREV CLASS   NEXT CLASS | **FRAMES**    **NO FRAMES** |  |
| SUMMARY:  INNER \| FIELD \| CONSTR \| METHOD | DETAIL:  FIELD \| CONSTR \| METHOD |  |


---
