# Supplementary material for: rDock: A Fast, Versatile and Open Source Program for Docking Ligands to Proteins and Nucleic Acids
Source: PLoS Comput Biol. 2014 Apr 10;10(4):e1003571. doi: 10.1371/journal.pcbi.1003571 (PMC3983074; doi:10.1371/journal.pcbi.1003571)
Supplement: Software S1 — Compressed file with the source code of the rDock software for ligand docking to Proteins and Nucleic Acids. (GZ) [file pcbi.1003571.s010.gz › rDock_2013.1_src/gettext-0.18.tar.gz!/gettext-0.18/gettext-runtime/intl-java/javadoc2/gnu/gettext/package-summary.html]

: Package gnu.gettext


|  |  |  |
| --- | --- | --- |
| **Package** \| Class \| **Tree** \| **Deprecated** \| **Index** \| **Help** | |  |
| PREV PACKAGE   NEXT PACKAGE | **FRAMES**    **NO FRAMES** |  |


---

## Package gnu.gettext

|  |  |
| --- | --- |
| **Class Summary** | |
| **GettextResource** | This class implements the main GNU libintl functions in Java. |

---


|  |  |  |
| --- | --- | --- |
| **Package** \| Class \| **Tree** \| **Deprecated** \| **Index** \| **Help** | |  |
| PREV PACKAGE   NEXT PACKAGE | **FRAMES**    **NO FRAMES** |  |


---
