# Supplementary material for: rDock: A Fast, Versatile and Open Source Program for Docking Ligands to Proteins and Nucleic Acids
Source: PLoS Comput Biol. 2014 Apr 10;10(4):e1003571. doi: 10.1371/journal.pcbi.1003571 (PMC3983074; doi:10.1371/journal.pcbi.1003571)
Supplement: Software S1 — Compressed file with the source code of the rDock software for ligand docking to Proteins and Nucleic Acids. (GZ) [file pcbi.1003571.s010.gz › rDock_2013.1_src/gettext-0.18.tar.gz!/gettext-0.18/gettext-tools/projects/TP/teams.html]

National translation teams


# Translation Project

**Welcome**  
**Changes**

**Overview**

**The Packages**  
**The Teams**  
**The Disclaimer**  
**The Robot**

**Info for...**

**Maintainers**  
**Coordinators**  
**Translators**

**Documents**

**Contributors**  
**File List**  
**Tools**

# National translation teams

| Code | Language | Team address |
| --- | --- | --- |
| af | Afrikaans | i18n@af.org.za |
| sq | Albanian | translation-team-sq@lists.sourceforge.net |
| am | Amharic | locales@geez.org |
| ar | Arabic | doc@arabeyes.org |
| es\_AR | Argentinian | translation-es-AR@lists.sourceforge.net |
| hy | Armenian | translation-team-hy@lists.sourceforge.net |
| ast | Asturian | xspuente@asturies.org |
| az | Azerbaijani | translation-team-az@lists.sourceforge.net |
| eu | Basque | translation-team-eu@lists.sourceforge.net |
| be | Belarusian | i18n@mova.org |
| bs | Bosnian | mtodorov@alu.hr |
| pt\_BR | Brazilian Portuguese | ldp-br@bazar.conectiva.com.br |
| bg | Bulgarian | dict@fsa-bg.org |
| ca | Catalan | ca@dodds.net |
| zh\_HK | Chinese (Hong Kong) | community@linuxhall.org |
| zh\_CN | Chinese (simplified) | translation-team-zh-cn@lists.sourceforge.net |
| zh\_TW | Chinese (traditional) | zh-l10n@linux.org.tw |
| hr | Croatian | lokalizacija@linux.hr |
| cs | Czech | translation-team-cs@lists.sourceforge.net |
| da | Danish | dansk@dansk-gruppen.dk |
| nl | Dutch | vertaling@vrijschrift.org |
| dz | Dzongkha | dzongkha-l10n@lists.sourceforge.net |
| en | English | en@translate.freefriends.org |
| en\_GB | English (British) | en\_gb@li.org |
| eo | Esperanto | translation-team-eo@lists.sourceforge.net |
| et | Estonian | linux-ee@lists.eenet.ee |
| fi | Finnish | translation-team-fi@lists.sourceforge.net |
| fr | French | traduc@traduc.org |
| gl | Galician | gpul-traduccion@ceu.fi.udc.es |
| ka | Georgian | ka@li.org |
| de | German | translation-team-de@lists.sourceforge.net |
| el | Greek | nls@tux.hellug.gr |
| gu | Gujarati | indianoss-gujarati@lists.sourceforge.net |
| he | Hebrew | eliz@gnu.org |
| hi | Hindi | fedora-trans-hi@redhat.com |
| hu | Hungarian | translation-team-hu@lists.sourceforge.net |
| is | Icelandic | kde-isl@mmedia.is |
| id | Indonesian | translation-team-id@lists.sourceforge.net |
| ga | Irish | gaeilge-gnulinux@lists.sourceforge.net |
| it | Italian | tp@lists.linux.it |
| ja | Japanese | translation-team-ja@lists.sourceforge.net |
| rw | Kinyarwanda | translation-team-rw@lists.sourceforge.net |
| ky | Kirghiz | i18n-team-ky-kyrgyz@lists.sourceforge.net |
| ko | Korean | translation-team-ko@lists.sourceforge.net |
| ku | Kurdish | ku@li.org |
| lv | Latvian | lv@li.org |
| lt | Lithuanian | komp\_lt@konferencijos.lt |
| art-lojban | Lojban | translation-art-lojban@lists.sourceforge.net |
| lg | Luganda | kompyuta@kizito.freeuk.com |
| mk | Macedonian | ufo@linux.net.mk |
| mg | Malagasy | dikateny@linuxmg.org |
| ms | Malay | translation-team-ms@lists.sourceforge.net |
| mt | Maltese | l10n@linux.org.mt |
| mn | Mongolian | openmn-translation@lists.sourceforge.net |
| ne | Nepali | translation-team-ne@lists.sourceforge.net |
| es\_NI | Nicaraguian | eichwalk@iro.umontreal.ca |
| nso | Northern Sotho | sipedi@translate.org.za |
| no | Norwegian | i18n-nb@lister.ping.uio.no |
| nb | Norwegian Bokmaal | i18n-nb@lister.ping.uio.no |
| nn | Norwegian Nynorsk | i18n-nn@lister.ping.uio.no |
| no@nynorsk | Norwegian Nynorsk | i18n-nn@lister.ping.uio.no |
| or | Oriya | gora\_mohanty@yahoo.co.in |
| fa | Persian | translation-team-fa@lists.sourceforge.net |
| pl | Polish | translation-team-pl@lists.sourceforge.net |
| pt | Portuguese | translation-team-pt@lists.sourceforge.net |
| pa | Punjabi | fedora-trans-pa@redhat.com |
| rm | Rhaeto-Romance | gnu-rumantsch@guglielmtux.ch |
| ro | Romanian | translation-team-ro@lists.sourceforge.net |
| ru | Russian | ru@li.org |
| sr | Serbian | gnu@prevod.org |
| si | Sinhalese | ananda.ruhunuhewa@inalco.fr |
| sk | Slovak | sk-i18n@lists.linux.sk |
| sl | Slovenian | translation-team-sl@lists.sourceforge.net |
| st | Sotho | sesotho@translate.org.za |
| es | Spanish | es@li.org |
| es\_IC | Spanish (Canary Islands) | es\_ic@eListas.net |
| sw | Swahili | translation-team-sw@lists.sourceforge.net |
| ss | Swati | swati@translate.org.za |
| sv | Swedish | tp-sv@listor.tp-sv.se |
| tg | Tajik | translations@tajikngo.org |
| ta | Tamil | translation-team-ta@lists.sourceforge.net |
| th | Thai | thailang@buraphalinux.org |
| tr | Turkish | gnu-tr-u12a@lists.sourceforge.net |
| tk | Turkmen | kakilikgroup@yahoo.com |
| uk | Ukrainian | translation-team-uk@lists.sourceforge.net |
| ven | Venda | venda@translate.org.za |
| vi | Vietnamese | vi-VN@googlegroups.com |
| wa | Walloon | linux-wa@walon.org |
| cy | Welsh | cy@pengwyn.linux.org.uk |
| xh | Xhosa | xhosa@translate.org.za |
| zu | Zulu | zulu@translate.org.za |

(Last updated on 2007-06-27 11:28 +0200.)

Your comments
are welcome.
