# Supplementary material for: rDock: A Fast, Versatile and Open Source Program for Docking Ligands to Proteins and Nucleic Acids
Source: PLoS Comput Biol. 2014 Apr 10;10(4):e1003571. doi: 10.1371/journal.pcbi.1003571 (PMC3983074; doi:10.1371/journal.pcbi.1003571)
Supplement: Software S1 — Compressed file with the source code of the rDock software for ligand docking to Proteins and Nucleic Acids. (GZ) [file pcbi.1003571.s010.gz › rDock_2013.1_src/gettext-0.18.tar.gz!/gettext-0.18/gettext-tools/projects/KDE/teams.html]

 

The KDE Translation Teams


# The KDE Translation Teams

Many KDE applications have been translated to **more than 50
languages** already and the number of both the translated applications and
the supported languages is still growing. For information on how complete the
translation of the current KDE user interface to the individual languages
really is please see the respective statistics page. (Documentation translation is still in
its first stages for many teams.) The languages distributed with the last
official KDE version are listed on a separate
page.

Almost all language teams are looking for additional
translators. And, of course, there are still a lot of languages missing in KDE.
So if you have some spare time and a good knowledge of English, please consider
joining one of the teams and help translating KDE to your native language. Just
write to the appropriate team coordinator in the list below. If there's no
coordinator listed for your language, take a look at the
Translation HOWTO for more information on
how new languages are introduced to KDE and subscribe to the
mailinglist
for translators and documenters.

The team table below is sorted according to the official lists
of ISO 639 language codes. (See
http://lcweb.loc.g
ov/standards/iso639-2/englangn.html). We are
presently switching from the 2-letter codes to the 3-letter codes.) In addition
to the basic team data like the **coordinator(s)**, the
**mailing list**, and the **team's web site**, the
list now contains links to:

- compressed **archives of all GUI and documentation
  files** that were already translated by the respective language team;
  these files are usually of the type ".po" (GNU gettext) and ".docbook"
  (XML)
- text files that contain **all message strings of the
  translated .po** files -- these files are sometimes called
  **"compendia"** and are used for instance by specialized
  translation programs like KBabel (the
  recommended one for KDE translation).
- the **"highscore list"** for each team: an
  overview which .po files were already translated but are in need of an
  overhaul.
- the **doc statistics**: an overview of the
  current status if there are any doc translations available

|  |  |
| --- | --- |
|  |  |
| af | Afrikaans team |
|  | - team coordinator(s): Frikkie Thirion - mailing list: send an empty mail to   lia-subscribe@af.org.za - web site: kde.af.org.za - .po and doc archive:   i18n.kde.org/translation\_archive/kde-i18n-af.tar.bz2     (size:  1.7M) - .po messages translated:   i18n.kde.org/po\_overview/af.messages.bz2    (size:  464k) - .po files in need of a revision:   i18n.kde.org/stats/gui/af-highscore.html - doc statistics: -- |
| ara | Arabic team |
|  | - team coordinator(s): Mohammed Elzubeir - mailing list: doc@arabeyes.org (subscribe via   www.arabeyes.org/cgi-bin/mailman/listinfo/doc) - web site: www.arabeyes.org - .po and doc archive:   i18n.kde.org/translation\_archive/kde-i18n-ara.tar.bz2     (size:  429k) - .po messages translated:   i18n.kde.org/po\_overview/ara.messages.bz2    (size:  85k) - .po files in need of a revision:   i18n.kde.org/stats/gui/ara-highscore.html - doc statistics: -- |
| az | Azerbaijani team |
|  | - team coordinator(s): Vasif Ismailoglu - mailing list: -- - web site: www.linuxaz.com - .po and doc archive:   i18n.kde.org/translation\_archive/kde-i18n-az.tar.bz2     (size:  1.7M) - .po messages translated:   i18n.kde.org/po\_overview/az.messages.bz2    (size:  480k) - .po files in need of a revision:   i18n.kde.org/stats/gui/az-highscore.html - doc statistics: -- |
| bg | Bulgarian team |
|  | - team coordinator(s):   Vesselina Nikolova - mailing list: -- - web site:   www.galgenberg.net/~vesseli   na/bg.html - .po and doc archive:   i18n.kde.org/translation\_archive/kde-i18n-bg.tar.bz2     (size:  1.5M) - .po messages translated:   i18n.kde.org/po\_overview/bg.messages.bz2    (size:  12k) - .po files in need of a revision:   i18n.kde.org/stats/gui/bg-highscore.html - doc statistics: -- |
| bn | Bengali team |
|  | - team coordinator(s):   Ahmed, Taneem - mailing list: -- - web site: -- - .po and doc archive:-- - .po messages translated: -- - .po files in need of a revision: -- - doc statistics: -- |
| bo | Tibetan team |
|  | - team coordinator(s): Katrin Norbu - mailing list: -- - web site: -- - .po and doc archive:-- - .po messages translated: -- - .po files in need of a revision: -- - doc statistics: -- |
| br | Breton team |
|  | - team coordinator(s):   Jañ-Mai Drapier - mailing list: -- - web site: perso.club-internet.fr/jdrapier - .po and doc archive:   i18n.kde.org/translation\_archive/kde-i18n-br.tar.bz2     (size:  548k) - .po messages translated:   i18n.kde.org/po\_overview/br.messages.bz2    (size:  62k) - .po files in need of a revision:   i18n.kde.org/stats/gui/br-highscore.html - doc statistics: -- |
| bs | Bosnian team |
|  | - coordinator(s): Neshiren   Armin, Amila Akagic - mailing list: Send mail to   kde-subscribe@lugbih.org - web site: kde.lugbih.org - .po and doc archive:   i18n.kde.org/translation\_archive/kde-i18n-bs.tar.bz2     (size:  274k) - .po messages translated:   i18n.kde.org/po\_overview/bs.messages.bz2     (size:  28k) - .po files in need of a revision:   i18n.kde.org/stats/gui/bs-highscore.html - doc statistics: -- |
| ca | Catalan team |
|  | - team coordinator(s): Sebastià Pla - mailing list: send a mail with "subscribe" in the subject   line to: linux-ca-request@chanae.alpha   net.ch - web site:   linux.softcatala.org/projecte   s/kde/ - .po and doc archive:   i18n.kde.org/translation\_archive/kde-i18n-ca.tar.bz2    (size:  920k) - .po messages translated:   i18n.kde.org/po\_overview/ca.messages.bz2    (size:  183k) - .po files in need of a revision:   i18n.kde.org/stats/gui/ca-highscore.html - doc statistics: -- |
| cs | Czech team |
|  | - coordinator(s): Miroslav   Flídr (GUI), Lukáš   Tinkl (documentation) - mailing list: kde-czech-apps@lists.sourceforge.net - web site: kde-czech.sourceforge.net - (Subscription via   lists.sourceforge.net/lists/listinfo/kde-czech-apps) - .po and doc archive:   i18n.kde.org/translation\_archive/kde-i18n-cs.tar.bz2     (size:  1.9M) - .po messages translated:   i18n.kde.org/po\_overview/cs.messages.bz2     (size:  549k) - .po files in need of a revision:   i18n.kde.org/stats/gui/cs-highscore.html - doc statistics:   i18n.kde.org/stats/doc/index.php?team=cs |
| cy | Welsh team |
|  | - coordinator(s): Elfed   Lewis - mailing list: -- - web site: -- - .po and doc archive:   i18n.kde.org/translation\_archive/kde-i18n-cy.tar.bz2    (size:  205k) - .po messages translated:   i18n.kde.org/po\_overview/cy.messages.bz2     (size:  12k) - .po files in need of a revision:   i18n.kde.org/stats/gui/cy-highscore.html - doc statistics: -- |
| da | Danish team |
|  | - coordinator(s): Erik   Kjær Pedersen - mailing list: -- - web site: -- - .po and doc archive:   i18n.kde.org/translation\_archive/kde-i18n-da.tar.bz2    (size:  4.5M) - .po messages translated:   i18n.kde.org/po\_overview/da.messages.bz2     (size:  1.1M) - .po files in need of a revision:   i18n.kde.org/stats/gui/da-highscore.html - doc statistics:   i18n.kde.org/stats/doc/index.php?team=da |
| de | German team |
|  | - coordinator(s): Thomas Diehl   (GUI and documentation) Malte   Knörr (web site) - mailing lists: There are separate KDE and web translation   lists. To get on the KDE translation list (GUI and documentation) send mail   with "subscribe" in the subject line to   kde-i18n-de-request@kde.org.    To get on the web site translation list send mail with "subscribe" in the   subject line to www-de-request@kde.org - team web site: i18n.kde.org/teams/de/ - user web site:   www.kde.org/international/germ   any/ - web forum: www.dtp-service.com/discus\_d/ - .po and doc archive:   i18n.kde.org/translation\_archive/kde-i18n-de.tar.bz2     (size:  7.6M) - .po messages translated:   i18n.kde.org/po\_overview/de.messages.bz2     (size:  1.3M) - .po files in need of a revision:   i18n.kde.org/stats/gui/de-highscore.html - doc statistics:   i18n.kde.org/stats/doc/index.php?team=de |
| el | Greek team |
|  | - coordinator(s): Dimitris Kamenopoulos - mailing list: kdei18ngr@hellug.gr (to subscribe send mail to   majordomo@hellug.gr with "subscribe   kdei18ngr" in the message body) - web site: i18n.kde.org/teams/el/ - .po and doc archive:   i18n.kde.org/translation\_archive/kde-i18n-el.tar.bz2    (size:  934k) - .po messages translated:   i18n.kde.org/po\_overview/el.messages.bz2     (size:  8k) - .po files in need of a revision:   i18n.kde.org/stats/gui/el-highscore.html - doc statistics: -- |
| en\_GB | British English Team |
|  | - team coordinator(s):   John Knight - mailing list: -- - web site: -- - .po and doc archive:   i18n.kde.org/translation\_archive/kde-i18n-en\_GB.tar.bz2    (size:  1.6M) - .po messages translated:   i18n.kde.org/po\_overview/en\_GB.message   s.bz2  (size:  466k) - .po files in need of a revision:   i18n.kde.org/stats/gui/highscore-en\_GB   .html - doc statistics: -- |
| eo | Esperanto team |
|  | - team coordinator(s): Wolfram Diestel - mailing list on translating Linux/GNU-applications (not   focussed on KDE): eo@li.org; send mail to    majordomo@li.org with "subscribe eo" in the message body - web site:   www.uni-leipzi   g.de/esperanto/material/kde/kde.html - .po and doc archive:   i18n.kde.org/translation\_archive/kde-i18n-eo.tar.bz2    (size:  1.4M) - .po messages translated:   i18n.kde.org/po\_overview/eo.messages.bz2     (size:  414k) - .po files in need of a revision:   i18n.kde.org/stats/gui/eo-highscore.html - doc statistics: -- |
| es | Spanish team |
|  | - team coordinators: Jaime   Robles - mailing list: send message to   majordomo@kybs.de with the words   "subscribe kde-es" in the body - web site: i18n.kde.org/teams/es/ - .po and doc archive:   i18n.kde.org/translation\_archive/kde-i18n-es.tar.bz2    (size:  4.2M) - .po messages translated:   i18n.kde.org/po\_overview/es.messages.bz2     (size:  38k) - .po files in need of a revision:   i18n.kde.org/stats/gui/es-highscore.html - doc statistics:   i18n.kde.org/stats/doc/index.php?team=es |
| et | Estonian team |
|  | - coordinator(s): Hasso   Tepper - mailing list: linux-ee@eenet.ee (for subscription send mail   to listproc@lists.edu.ee with body   "subscribe linux-ee firstname lastname") - web site: -- - .po and doc archive:   i18n.kde.org/translation\_archive/kde-i18n-et.tar.bz2    (size:  1.6M) - .po messages translated:   i18n.kde.org/po\_overview/et.messages.bz2    (size:  506k) - .po files in need of a revision:   i18n.kde.org/stats/gui/et-highscore.html - doc statistics: -- |
| eu | Basque team |
|  | - coordinator(s): Marcos Goienetxe - mailing list: send mail with "subscribe" in the subject line   to: linux-eu-request@chanae.alphanet.c - web site: kde.eu.euskal-linux.org - .po and doc archive:   i18n.kde.org/translation\_archive/kde-i18n-eu.tar.bz2    (size:  567k) - .po messages translated:   i18n.kde.org/po\_overview/eu.messages.bz2    (size:  86k) - .po files in need of a revision:   i18n.kde.org/stats/gui/eu-highscore.html - doc statistics: -- |
| fa | Farsi (Persian) team |
|  | - coordinator(s): Arash   Zeini - mailing list: subscribe via   lists.sharif.edu/mailman/listinfo/farsikde - web site: www.farsikde.com - .po and doc archive: -- - .po messages translated: -- - .po files in need of a revision: -- - doc statistics: -- |
| fi | Finnish team |
|  | - coordinator(s): Kim   Enkovaara - mailing list: -- - web site: kde-i18n-fi.sourceforge.net - .po and doc archive:   i18n.kde.org/translation\_archive/kde-i18n-fi.tar.bz2    (size:  1.5M) - .po messages translated:   i18n.kde.org/po\_overview/fi.messages.bz2    (size:  86k) - .po files in need of a revision:   i18n.kde.org/stats/gui/fi-highscore.html - doc statistics: -- |
| fo | Faroese team |
|  | - coordinator(s): Aki Nielsen - mailing list: -- - web site: -- - .po and doc archive: -- - .po messages translated: -- - .po files in need of a revision: -- - doc statistics: -- |
| fr | French team |
|  | - team coordinator(s):   Thibaut Cousin (GUI translation)    Ludovic Grossard   (documentation translation)  Serge   'Sergio' Tellene (web server translation). - mailing list: send an empty mail to   kde-francophone-subscribe@kde.org - team web site: i18n.kde.org/teams/fr/ - user web site: www.kde.org/fr - .po and doc archive:   i18n.kde.org/translation\_archive/kde-i18n-fr.tar.bz2    (size:  6.9M) - .po messages translated:   i18n.kde.org/po\_overview/fr.messages.bz2    (size:  26k) - .po files in need of a revision:   i18n.kde.org/stats/gui/fr-highscore.html - doc statistics:   i18n.kde.org/stats/doc/index.php?team=fr |
| ga | Irish Gaelic team |
|  | - coordinator(s): Sean V. Kelley - mailing list: see   lists.sourceforge.net/mailman/listinfo/gaeilge-gnulinux - web site: gaeilge.sourceforge.net - .po and doc archive:   i18n.kde.org/translation\_archive/kde-i18n-ga.tar.bz2    (size:  218k) - .po messages translated:   i18n.kde.org/po\_overview/ga.messages.bz2    (size:  2k) - .po files in need of a revision:   i18n.kde.org/stats/gui/ga-highscore.html - doc statistics: -- |
| gl | Gallegan team |
|  | - coordinator(s): Alejandro   Pose Rey, Jesús Bravo   Álvarez - mailing list: trasno@ceu.fi.udc.es (for information about how   to subscribe, visit trasno.gpul.org/axuda/lista.php) - web site: trasno.gpul.org/kde/ - .po and doc archive: -- - .po messages translated:   i18n.kde.org/po\_overview/gl.messages.bz2    (size:  64k) - .po files in need of a revision:   i18n.kde.org/stats/gui/gl-highscore.html - doc statistics: -- |
| gu | Gujarati team |
|  | - coordinator(s): Apu   Shah - mailing list: -- - web site: -- - .po and doc archive: -- - .po messages translated: -- - .po files in need of a revision: -- - doc statistics: -- |
| he | Hebrew team |
|  | - coordinator(s): Meni   Livne - mailing list: groups.yahoo.com/group/kde-il - web site: www.kde.org/il/hebrew - .po and doc archive:   i18n.kde.org/translation\_archive/kde-i18n-he.tar.bz2    (size:  1.5M) - .po messages translated:   i18n.kde.org/po\_overview/he.messages.bz2    (size:  381k) - .po files in need of a revision:   i18n.kde.org/stats/gui/he-highscore.html - doc statistics: -- |
| hi | Hindi team |
|  | - coordinator(s): Harsh   Kumar - mailing list: -- - web site: -- - .po and doc archive: -- - .po messages translated: -- - .po files in need of a revision: -- - doc statistics: -- |
| hr | Croatian team |
|  | - team coordinator(s):   Vlatko Kosturjak - mailing list: send mail to   lokalizacija-subscribe@linux.hr - web site: lokalizacija.linux.hr/kde/ - .po and doc archive:   i18n.kde.org/translation\_archive/kde-i18n-hr.tar.bz2    (size:  894k) - .po messages translated:   i18n.kde.org/po\_overview/hr.messages.bz2    (size:  143k) - .po files in need of a revision:   i18n.kde.org/stats/gui/hr-highscore.html - doc statistics: -- |
| hu | Hungarian team |
|  | - coordinator(s): Marcell   Lengyel - mailing list: kde-lista@sophia.jpte.hu (to get in send mail   with "subscribe" in the body to   kde-lista-request@sophia.jpte.hu) - web site: www.kde.hu - .po and doc archive:   i18n.kde.org/translation\_archive/kde-i18n-hu.tar.bz2    (size:  2.7M) - .po messages translated:   i18n.kde.org/po\_overview/hu.messages.bz2    (size: [an error occurred while processing this directive]) - .po files in need of a revision:   i18n.kde.org/stats/gui/hu-highscore.html - doc statistics:   i18n.kde.org/stats/doc/index.php?team=hu |
| id/ind | Indonesian team |
|  | - coordinator(s):   I Made Wiryana,   Haris Fauzi - mailing list: -- - web site: merdeka.trustix.co.id/kde - .po and doc archive:   i18n.kde.org/translation\_archive/kde-i18n-ind.tar.bz2     (size:  1.5M) - .po messages translated:   i18n.kde.org/po\_overview/ind.messages.bz2    (size:  390k) - .po files in need of a revision:   i18n.kde.org/stats/gui/ind-highscore.html - doc statistics: -- |
| is | Icelandic team |
|  | - team coordinator(s): Logi   Ragnarsson, Thorarinn R.   Einarsson, Bjarni R. Einarsson - mailing list: kde-isl@mmedia.is (no subscription info   available yet) - web site: www.is.kde.org - .po and doc archive:   i18n.kde.org/translation\_archive/kde-i18n-is.tar.bz2    (size:  1.2M) - .po messages translated:   i18n.kde.org/po\_overview/is.messages.bz2    (size:  1k) - .po files in need of a revision:   i18n.kde.org/stats/gui/is-highscore.html - doc statistics: -- |
| it | Italian team |
|  | - coordinator(s): Andrea   Rizzi - mailing list: send mail with "subscribe" in the subject line   to: kde-i18n-it-request@kde.org - web site: i18n.kde.org/teams/it/ - .po and doc archive:   i18n.kde.org/translation\_archive/kde-i18n-it.tar.bz2    (size:  1.8M) - .po messages translated:   i18n.kde.org/po\_overview/it.messages.bz2     (size:  520k) - .po files in need of a revision:   i18n.kde.org/stats/gui/it-highscore.html - doc statistics:   i18n.kde.org/stats/doc/index.php?team=it |
| ja | Japanese team |
|  | - coordinator(s): Taiki   Komoda - mailing list: send mail to   Kuser-ctl@kde.gr.jp with "subscribe"   in the message body. - web site: www.kde.gr.jp - .po and doc archive:   i18n.kde.org/translation\_archive/kde-i18n-ja.tar.bz2    (size:  1.5M) - .po messages translated:   i18n.kde.org/po\_overview/ja.messages.bz2    (size: [an error occurred while processing this directive]) - .po files in need of a revision:   i18n.kde.org/stats/gui/ja-highscore.html - doc statistics:   i18n.kde.org/stats/doc/index.php?team=ja |
| km | Khmer (Cambodian) team |
|  | - coordinator(s): Neang,   Savun - mailing list: kdelist@khmermp3.com (no subscription info   available yet) - web site: kde.khmermp3.com - .po and doc archive: -- - .po messages translated: -- - .po files in need of a revision: -- - doc statistics: -- |
| ko | Korean team |
|  | - coordinator(s): KIM   KyungHeon - mailing list: kde-i18n@kldp.org (use the web page at   list.kldp.org/mailman/listinfo/kde-i18n/   to subscribe) - team web site: www.kde.or.kr - user web site: kde.kldp.org - .po and doc archive:   i18n.kde.org/translation\_archive/kde-i18n-ko.tar.bz2    (size:  991k) - .po messages translated:   i18n.kde.org/po\_overview/ko.messages.bz2    (size:  10k) - .po files in need of a revision:   i18n.kde.org/stats/gui/ko-highscore.html - doc statistics: -- |
| kur | Kurdish team |
|  | - team coordinator(s): Abou   Gerdi - mailing list: -- - web site: -- - .po and doc archive:   i18n.kde.org/translation\_archive/kde-i18n-kur.tar.bz2     (size:  232k) - .po messages translated:   i18n.kde.org/po\_overview/kur.messages.bz2    (size:  33k) - .po files in need of a revision:   i18n.kde.org/stats/gui/kur-highscore.html - doc statistics: -- |
| lt | Lithuanian team |
|  | - coordinator(s): Richard   Cepas - mailing list: -- - web site: -- - .po and doc archive:   i18n.kde.org/translation\_archive/kde-i18n-lt.tar.bz2    (size:  948k) - .po messages translated:   i18n.kde.org/po\_overview/lt.messages.bz2    (size:  270k) - .po files in need of a revision:   i18n.kde.org/stats/gui/lt-highscore.html - doc statistics: -- |
| lv | Latvian team |
|  | - coordinator(s): Andris   Maziks - mailing list: -- - web site: -- - .po and doc archive: -- - .po messages translated:   i18n.kde.org/po\_overview/lv.messages.bz2    (size:  247k) - .po files in need of a revision:   i18n.kde.org/stats/gui/lv-highscore.html - doc statistics: -- |
| mi | Maori team |
|  | - coordinator(s): **unmaintained** - mailing list: -- - web site: -- - .po and doc archive:   i18n.kde.org/translation\_archive/kde-i18n-mi.tar.bz2    (size:  230k) - .po messages translated:   i18n.kde.org/po\_overview/mi.messages.bz2    (size:  9k) - .po files in need of a revision:   i18n.kde.org/stats/gui/mi-highscore.html - doc statistics: -- |
| mk | Macedonian team |
|  | - coordinator(s): Danko   Ilik - mailing list: -- - web site: slavej.pmf.ukim.edu.mk - .po and doc archive:   i18n.kde.org/translation\_archive/kde-i18n-mk.tar.bz2    (size:  700k) - .po messages translated:   i18n.kde.org/po\_overview/mk.messages.bz2    (size:  122k) - .po files in need of a revision:   i18n.kde.org/stats/gui/mk-highscore.html - doc statistics: -- |
| mr | Marathi team |
|  | - coordinator(s): Rahul   Palkar - mailing list: -- - web site: -- - .po and doc archive: -- - .po messages translated: -- - .po files in need of a revision: -- - doc statistics: -- |
| mt | Maltese team |
|  | - coordinator(s): Ramon   Casha - mailing list: -- - web site: -- - .po and doc archive: -- - .po messages translated: -- - .po files in need of a revision: -- - doc statistics: -- |
| nl | Dutch team |
|  | - team coordinator(s): Rinse de   Vries, see also contact list at www.kde.nl/nl/contact.html - mailing list: i18n@kde.nl (send an empty message to   kde-nl-subscribe@egroups.com   to subscribe) - mailing list archive:   www.egroups.com/group/kde-nl - web site: www.kde.nl/nl - user web site: www.kde.nl - .po and doc archive:   i18n.kde.org/translation\_archive/kde-i18n-nl.tar.bz2    (size:  2.8M) - .po messages translated:   i18n.kde.org/po\_overview/nl.messages.bz2    (size:  741k) - .po files in need of a revision:   i18n.kde.org/stats/gui/nl-highscore.html - doc statistics:   i18n.kde.org/stats/doc/index.php?team=nl |
| no | Norwegian (Bokmål) team |
|  | - coordinator(s): Christian A. Strømmen - web site: -- - mailing list: -- - .po and doc archive:   i18n.kde.org/translation\_archive/kde-i18n-no.tar.bz2    (size:  1.7M) - .po messages translated:   i18n.kde.org/po\_overview/no.messages.bz2    (size:  89k) - .po files in need of a revision:   i18n.kde.org/stats/gui/no-highscore.html - doc statistics: -- |
| no\_NY | Norwegian (Nynorsk) team |
|  | - coordinator(s): Gaute   Hvoslef Kvalnes - web site: i18n.kde.org/teams/no\_NY - mailing list: -- - .po and doc archive:   i18n.kde.org/translation\_archive/kde-i18n-no\_NY.tar.bz2    (size:  1.6M) - .po messages translated:   i18n.kde.org/po\_overview/no\_NY.messages.bz2    (size:  466k) - .po files in need of a revision:   i18n.kde.org/stats/gui/no\_NY-highscore.html - doc statistics: -- |
| oc | Occitan team |
|  | - coordinator(s): Joan Luc Labòrda - web site: -- - .po and doc archive:   i18n.kde.org/translation\_archive/kde-i18n-oc.tar.bz2    (size:  306k) - .po messages translated:   i18n.kde.org/po\_overview/oc.messages.bz2    (size:  34k) - .po files in need of a revision:   i18n.kde.org/stats/gui/oc-highscore.html - doc statistics: -- |
| pl | Polish team |
|  | - coordinator(s):   Jacek Stolarczyk - mailing list: kde-transl@mer.chemia.polsl.gliwice.pl (to   subscribe send an empty mail to   kde-transl-request@mer.chemia.polsl.gliwice.pl) - web site:   mer.chemia.polsl.gliwice.pl/kde/transl/ - .po and doc archive:   i18n.kde.org/translation\_archive/kde-i18n-pl.tar.bz2    (size:  1.5M) - .po messages translated:   i18n.kde.org/po\_overview/pl.messages.bz2    (size:  10k) - .po files in need of a revision:   i18n.kde.org/stats/gui/pl-highscore.html (more   info at mer.chemia.polsl.gliwice.pl/kde/transl/pliki-po/) - doc statistics: -- |
| pt | Portuguese team |
|  | - coordinator(s): Pedro   Morais - mailing list: kde@poli.org (for subscription send mail to   Pedro Morais) - web site: kde.poli.org - .po and doc archive:   i18n.kde.org/translation\_archive/kde-i18n-pt.tar.bz2    (size:  2.1M) - .po messages translated:   i18n.kde.org/po\_overview/pt.messages.bz2    (size:  648k) - .po files in need of a revision:   i18n.kde.org/stats/gui/pt-highscore.html - doc statistics:   i18n.kde.org/stats/doc/index.php?team=pt |
| pt\_BR | Brazilian Portuguese team |
|  | - team coordinator(s):   Roberto Teixeira - mailing list: ldp-br@bazar.conectiva.com.br (see the   web site for subscription   info) - web site: ldp-br.conectiva.com.br - .po and doc archive:   i18n.kde.org/translation\_archive/kde-i18n-pt\_BR.tar.bz2    (size:  1.4M) - .po messages translated:   i18n.kde.org/po\_overview/pt\_BR.messages.bz2    (size:  28k) - .po files in need of a revision:   i18n.kde.org/stats/gui/pt\_BR-highscore.html - doc statistics: -- |
| ro | Romanian team |
|  | - team coordinator(s): Claudiu Costin,   Florin Boariu - mailing list: See   www.egroups.com/group/ro-kde - web site: www.ro.kde.org - .po and doc archive:   i18n.kde.org/translation\_archive/kde-i18n-ro.tar.bz2    (size:  1.7M) - .po messages translated:   i18n.kde.org/po\_overview/ro.messages.bz2    (size:  422k) - .po files in need of a revision:   i18n.kde.org/stats/gui/ro-highscore.html - doc statistics: i18n.kde.org/stats/doc/html/doc-ro.html |
| ru | Russian team |
|  | - coordinator(s): Gregory   Mokhin - mailing list: subscribe by visiting:   maillist.mscom.ru/mailman/listinfo.cgi/kde-i18n/   or by sending "subscribe" to   kde-i18n-request@maillist.mscom.ru.   Archives are available at   maillist.mscom.ru/pipermail/kde-i18n/ - web site: -- - .po and doc archive:   i18n.kde.org/translation\_archive/kde-i18n-ru.tar.bz2    (size:  2.3M) - .po messages translated:   i18n.kde.org/po\_overview/ru.messages.bz2    (size:  696k) - .po files in need of a revision:   i18n.kde.org/stats/gui/ru-highscore.html - doc statistics:   http://i18n.kde.org/stats/doc/index.php?team=ru |
| sk | Slovak team |
|  | - coordinator(s): Juraj   Bednar - mailing list: subscribe via:   rak.isternet.sk/mailman/listinfo/sk-i18n   or by sending "subscribe" to sk-i18n-request@rak.isternet.sk.   Archives are available at rak.isternet.sk/pipermail/sk-i18n. - web site: ski18n.linux.sk - .po and doc archive:   i18n.kde.org/translation\_archive/kde-i18n-sk.tar.bz2    (size:  3.7M) - .po messages translated:   i18n.kde.org/po\_overview/sk.messages.bz2    (size:  867k) - .po files in need of a revision:   i18n.kde.org/stats/gui/sk-highscore.html - doc statistics:   i18n.kde.org/stats/doc/index.php?team=sk |
| sl | Slovenian team |
|  | - coordinator(s): Roman   Maurer - mailing list:   lugos-slo - team web site: i18n.kde.org/teams/sl/ - user web site: www.lugos.si/delo/slo/KDE-sl/ - .po and doc archive:   i18n.kde.org/translation\_archive/kde-i18n-sl.tar.bz2    (size:  5.1M) - .po messages translated:   i18n.kde.org/po\_overview/sl.messages.bz2    (size:  1.0M) - .po files in need of a revision:   i18n.kde.org/stats/gui/sl-highscore.html - doc statistics:   i18n.kde.org/stats/doc/index.php?team=sl |
| sr | Serbian team |
|  | - team coordinator(s): Marko Rosic,   Slobodan Markovic - mailing list: send an empty mail to   kde-yu-subscribe@egroups.com - web site: www.kde.org.yu - .po and doc archive:   i18n.kde.org/translation\_archive/kde-i18n-sr.tar.bz2    (size:  1.1M) - .po messages translated:   i18n.kde.org/po\_overview/sr.messages.bz2    (size:  282k) - .po files in need of a revision:   i18n.kde.org/stats/gui/sr-highscore.html - doc statistics: i18n.kde.org/stats/doc/html/doc-sr.html |
| sv | Swedish team |
|  | - coordinator(s): Anders   Widell, Karl Backström - mailing list:   http://lists.lysator.liu.se/mailman/listinfo/swedoc - web site: i18n.kde.org/teams/sv/ - .po and doc archive:   i18n.kde.org/translation\_archive/kde-i18n-sv.tar.bz2    (size:  4.9M) - .po messages translated:   i18n.kde.org/po\_overview/sv.messages.bz2    (size:  1.4M) - .po files in need of a revision:   i18n.kde.org/stats/gui/sv-highscore.html - doc statistics:   i18n.kde.org/stats/doc/index.php?team=sv&date=2001-08-13 |
| ta | Tamil team |
|  | - coordinator(s): Sivakumar   Shanmugasundaram - mailing list: -- - web site: www.tamillinux.org - .po and doc archive:   i18n.kde.org/translation\_archive/kde-i18n-ta.tar.bz2    (size:  1.0M) - .po messages translated:   i18n.kde.org/po\_overview/ta.messages.bz2    (size:  293k) - .po files in need of a revision:   i18n.kde.org/stats/gui/ta-highscore.html - doc statistics: -- |
| tg | Tajik team |
|  | - coordinator(s):   Roger Kovacs - mailing list: -- - web site: www.khujandcomptech.dyn.tj - .po and doc archive:-- - .po messages translated: -- - .po files in need of a revision: -- - doc statistics: -- |
| th | Thai team |
|  | - coordinator(s): Thaweesak   Taekratok - mailing list: -- - web site: -- - .po and doc archive:   i18n.kde.org/translation\_archive/kde-i18n-th.tar.bz2    (size:  1.1M) - .po messages translated:   i18n.kde.org/po\_overview/th.messages.bz2    (size:  1k) - .po files in need of a revision:   i18n.kde.org/stats/gui/th-highscore.html - doc statistics: -- |
| tr | Turkish team |
|  | - coordinator(s): Gorkem   Cetin - mailing list: kde-i18n-tr@kde.org (to get in send mail with   "subscribe" in the subject line to kde-i18n-tr-request@kde.org) - web site: www.tr.i18n.kde.org - .po and doc archive:   i18n.kde.org/translation\_archive/kde-i18n-tr.tar.bz2    (size:  1.7M) - .po messages translated:   i18n.kde.org/po\_overview/tr.messages.bz2    (size: [an error occurred while processing this directive]) - .po files in need of a revision:   i18n.kde.org/stats/gui/tr-highscore.html - doc statistics: -- |
| uk | Ukrainian team |
|  | - coordinator(s): Mike   Futerko, Andy Rysin - mailing list: linux@linux.org.ua (to get in send mail with   "subscribe linux your\_address" to majordomo@linux.org.ua) - web site: kde.alphadiz.com - .po and doc archive:   i18n.kde.org/translation\_archive/kde-i18n-uk.tar.bz2    (size:  1.4M) - .po messages translated:   i18n.kde.org/po\_overview/uk.messages.bz2    (size: [an error occurred while processing this directive]) - .po files in need of a revision:   i18n.kde.org/stats/gui/uk-highscore.html - doc statistics: -- |
| vi | Vietnamese team |
|  | - team coordinator(s): Nguyen   Hung.Vu - mailing list: to subcribe send email to:   KDE-i18n-VN-subscribe@yahoogroups.com to   unsubscribe send email to: KDE-i18n-VN-unsubscribe@yahoogroups.com to post a   message send email to: KDE-i18n-VN@yahoogroups.com - web site:   groups.yahoo.com/group/KDE-i18n-VN - .po and doc archive: -- - .po messages translated: -- - .po files in need of a revision: -- - doc statistics: -- |
| wa | Walloon team |
|  | - team coordinators: **unmaintained** (former maintainer:   Lorint Hendschel) - mailing list: -- - web site: users.skynet.be/bs302306/linux/ - .po and doc archive:   i18n.kde.org/translation\_archive/kde-i18n-wa.tar.bz2    (size:  471k) - .po messages translated:   i18n.kde.org/po\_overview/wa.messages.bz2    (size: [an error occurred while processing this directive]) - .po files in need of a revision:   i18n.kde.org/stats/gui/wa-highscore.html - doc statistics: -- |
| xh | Xhosa team |
|  | - team coordinator(s): Dwayne Bailey - mailing list: -- - web site: translate.org.za - .po and doc archive: -- - .po messages translated: -- - .po files in need of a revision: -- - doc statistics: -- |
| zh\_CN.GB2312 | Simplified Chinese team |
|  | - coordinator(s): Wang   Jian - mailing list: i18n-translation@lists.linux.net.cn (for   subscription send email with body set to one line "subscribe i18n-translation"   sympa@lists.linux.net.cn or visit   lists.linux.net.cn/wws/subscribe/i18n-translation) - web site: i18n.linux.net.cn - .po and doc archive:   i18n.kde.org/translation\_archive/kde-i18n-zh\_CN.GB2312.tar.bz2    (size:  1.3M) - .po messages translated:   i18n.kde.org/po\_overview/zh\_CN.GB2312.messages.bz2    (size:  326k) - .po files in need of a revision:   i18n.kde.org/stats/gui/zh\_CN.GB2312-highscore.html - doc statistics: -- |
| zh\_TW.Big5 | Traditional Chinese team |
|  | - coordinator(s): Kenduest Lee - mailing list: zh-l10n@linux.org.tw (Send email to   majordomo@linux.org.tw with mail   body "subscribe zh-l10n") - web site: i18n.linux.org.tw - .po and doc archive:   i18n.kde.org/translation\_archive/kde-i18n-   zh\_TW.Big5.tar.bz2  (size:  1.7M) - .po messages translated:   i18n.kde.org/po\_overview/zh\_TW   .Big5.messages.bz2  (size: [an error occurred while processing this directive]) - .po files in need of a revision:   i18n.kde.org/stats/gui/zh\_TW.Big5-highscore - doc statistics: -- |

 

---

 Page maintained by
Thomas Diehl. Last update 11 Oktober
2001
