# Supplementary material for: rDock: A Fast, Versatile and Open Source Program for Docking Ligands to Proteins and Nucleic Acids
Source: PLoS Comput Biol. 2014 Apr 10;10(4):e1003571. doi: 10.1371/journal.pcbi.1003571 (PMC3983074; doi:10.1371/journal.pcbi.1003571)
Supplement: Software S1 — Compressed file with the source code of the rDock software for ligand docking to Proteins and Nucleic Acids. (GZ) [file pcbi.1003571.s010.gz › rDock_2013.1_src/gettext-0.18.tar.gz!/gettext-0.18/gettext-tools/projects/GNOME/teams.html]

xml version="1.0" encoding="UTF-8"?


The GNOME Translation Project - Teams


## Teams:

Translation teams already exist for many languages.
To join a team, contact the team leader.  
If a team does not exist for your language, consider starting one.

|  |  |  |
| --- | --- | --- |
| **Language** | **Coordinator** | **Web Page** |
| Afrikaans | Petri Jooste | https://savannah.gnu.org/projects/gnomeaf/ |
| Albanian | Laurent Dhima | http://gnome-albanian.sourceforge.net/ |
| Amharic | Daniel Yacob | http://gnome.geez.org/Amharic/ |
| Arabic | Arafat Medini | http://www.arabeyes.org/ |
| Armenian | Norayr Chilingaryan |  |
| Assamese | Sunaram Patir |  |
| Australian English | Peter Lawler |  |
| Azerbaijani | Metin Amiroff | http://www.linux.gen.az/ |
| Basque | Iñaki Larrañaga Murgoitio |  |
| Belarusian | Ales Nyakhaychyk | http://www.i18n.linux.by/ |
| Bengali | Taneem Ahmed | http://www.bengalinux.org/projects/gnome/ |
| Bosnian | Amila Akagić | http://projekti.linux.org.ba/ |
| Brazilian Portugese | Gustavo Maciel Dias Vieira | http://gnome-br.sourceforge.net/ |
| Breton | Francis Tyers |  |
| Bulgarian | Alexander Shopov | http://gnome.cult.bg/ |
| Burmese | Zaw Win Aung |  |
| British English | Bastien Nocera | http://live.gnome.org/BritishEnglish |
| Canadian English | Adam Weinberger | http://www.vectors.cx/en\_ca.html |
| Catalan | Jordi Mas | http://www.softcatala.org/ |
| Chinese (simplified) | Wang Jian | Chinese Linux i18n |
| Chinese (traditional) | Abel Cheung | Temporary GNOME trad. Chinese translation mailing list |
| Croatian | Robert Sedak | http://lokalizacija.linux.hr/ |
| Czech | Stanislav Brabec |  |
| Danish | Ole Laursen | Oversættelse af Gnome |
| Dutch | Vincent van Adrighem | GNOME Nederlands |
| Dzongkha | Pema Geyleg | http://savannah.nongnu.org/projects/dzongkha-gnome/ |
| Esperanto | Artem Popov |  |
| Estonian | Priit Laes | http://et-gnome.sourceforge.net/ |
| Fijian | Timoci Tora |  |
| Finnish | Ilkka Tuohela | http://www.gnome-fi.org/ |
| French | Christophe Merlet (RedFox) | http://gnomefr.traduc.org/ |
| Galician | Ignacio Casal Quinteiro | http://trisquel.uvigo.es/traduccion/ |
| Georgian | Aiet Kolkhi | http://aiet.qartuli.net/projects/gnome\_en.php |
| German | Hendrik Richter | http://www.gnome.de/ |
| Greek | Simos Xenitellis | Hellenic GNOME Localisation Project |
| Gujarati | Nirav Mehta | http://www.utkarsh.org/ |
| Hausa | Bamiyo Odumah |  |
| Hebrew | Yair Hershkovitz | http://gnome-il.sourceforge.net/ |
| Hindi | Guntupalli Karunakar | http://www.indlinux.org/hindi/gnome/ |
| Hungarian | Gábor Kelemen | A GNOME Projekt |
| Hyam | Uchenna Agbim | http://zitt.sourceforge.net/ |
| Icelandic | Samúel Jón Gunnarsson | http://www.techattack.nu/~gnome/ |
| Igbo | Nwabunwanne | http://igbo.sourceforge.net/ |
| Indonesian | Mohammad Damt | http://gnome.linux.or.id/ |
| Iranian Azerbaijani | Amir Hedayaty | http://www.farsiweb.info/wiki/Main/GNOME |
| Irish | Paul Duffy |  |
| Italian | Alessio Frusciante | Progetto di traduzione italiana |
| Japanese | Takeshi Aihana | Japan GNOME Users Group |
| Jju | Uchenna Agbim | http://zitt.sourceforge.net/ |
| Kagoma | Uchenna Agbim | http://zitt.sourceforge.net/ |
| Kannada | Pramod R | http://kannada.sourceforge.net/ |
| Kazakh | Amrenov Kanat | http://linuxoid.spl.kz/gnome/ |
| Khmer | Javier Sola |  |
| Kinyarwanda | Steve Murphy |  |
| Kirghiz | Timur Jamakeev |  |
| Korean | Changwoo Ryu |  |
| Kurdish | Abdullah Ulas | http://gnome-kurdi.sourceforge.net/ |
| Lao | Anousak Souphavanh | http://opensource.muanglao.com/ |
| Latin | Mark Polo |  |
| Latvian | Artis Trops | http://ll10nt.laka.lv/ |
| Limburgish | Kenneth Rohde Christiansen |  |
| Lithuanian | Žygimantas Beručka | http://www.lietuvybe.org/gnome/ |
| Low Saxon | Jürgen Lüters |  |
| Macedonian | Arangel Angov | http://gnomk.sourceforge.net/ |
| Maithili | Rajesh Ranjan |  |
| Malay | Hasbullah bin Pit | http://gabai.sourceforge.net/ |
| Malayalam | Sajith V.K | http://www.keralaindustry.org/malayalam/ |
| Maltese | Ramon Casha | http://linux.org.mt/lingwa/ |
| Maori | John C Barstow | http://www.nzlinux.org.nz/l10n/ |
| Marathi | Jitendra Shah | http://www.indictrans.org/ |
| Mayan | Daniel Espinosa | http://www.lenmex.org/ |
| Mongolian | Sanlig Badral |  |
| Nahuatl | Daniel Espinosa | http://www.lenmex.org/ |
| Nepali | Pawan Chitrakar |  |
| Northern Sotho | Dwayne Bailey | http://translate.org.za/ |
| Norwegian | Kjartan Maraas |  |
| Occitan | Joan Luc Labòrda |  |
| Old English | James R. Johnson | http://home.comcast.net/~modean52/oeme\_gnome.htm |
| Oriya | Gora Mohanty | http://oriya.sarovar.org/ |
| Páez | Santiago José Ruano Rincón |  |
| Persian | Roozbeh Pournader | http://www.farsiweb.info/wiki/Main/GNOME |
| Polish | Artur Flinta | http://www.gnome.pl/ |
| Portuguese | Duarte Loreto |  |
| Punjabi | Amanpreet Singh Alam | http://punlinux.sourceforge.net/ |
| Pushto | Ismail Maskani |  |
| Quechua | Angel Javier Chulve |  |
| Romanian | Mugurel Tudor | Proiectul de traducere GNOME in romana |
| Russian | Dmitry G. Mastrukov | http://www.gnome.org.ru/ |
| Sanskrit | Mukesh G |  |
| Serbian | Daneelo Shegun | http://www.prevod.org/ |
| Sinhala | Anuradha Ratnaweera | http://sinhala.linux.lk/ |
| Slovak | Marcel Telka | Preklad Linuxu do slovenèiny |
| Slovenian | Andraz Tori | http://www.lugos.si/delo/slo/GNOME-sl/ |
| Spanish | Francisco Javier F. Serrador | GNOME Hispano |
| Swahili | Mturi Elias |  |
| Swedish | Christian Rose | http://www.gnome.se/sv/ |
| Tagalog | Eric Pareja |  |
| Tamil | Dinesh Nadarajah | http://tamilgnome.sourceforge.net/ |
| Telugu | Sunil Mohan | http://www.indlinux.org/telugu/ |
| Tetum | Peter Gossner | http://www.gnu.org/software/tetum/ |
| Thai | Krich Nasingkun and Paisa Seeluangsawat | http://gnome-th.sourceforge.net/ |
| Tibetan | Christopher Fynn | http://iris.lib.virginia.edu/tibet/tools/linux/gui/gnome/ |
| Tigrinya | Simon Mebrahtu | http://tinux.sourceforge.net/ |
| Turkish | Fatih Demir, Görkem Çetin | http://www.gnome.org.tr/ |
| Turkmen | Ghorban M. Tavakoly | http://kakilik.sourceforge.net/ |
| Tyap | Uchenna Agbim | http://zitt.sourceforge.net/ |
| Uighur | Abdurixit Abduxukur | http://groups.yahoo.com/group/gnome-uighur/ |
| Ukrainian | Maxim V. Dziumanenko |  |
| Urdu | Shahzad Chohan |  |
| Uzbek | Mashrab Kuvatov | http://freax.homelinux.net/ |
| Vietnamese | Nguyen Thai Ngoc Duy | http://gnomevi.sourceforge.net/ |
| Walloon | Pablo Saratxaga |  |
| Welsh | Dafydd Harries | http://muse.19inch.net/~daf/gnome-cy/ |
| Xhosa | Adi Attar |  |
| Yiddish | Raphael Finkel |  |
| Yoruba | Abayomi Mobolaji Onibudo | http://www.wazobiasoft.org/gnome/yoruba.htm |
| Zapotec | Daniel Espinosa | http://www.lenmex.org/ |
| Zulu | Dwayne Bailey | http://translate.org.za/ |

### Starting a new team

If no team for your language is listed here and you want to start a new one,
please send an email to announce the new team
to the mailing list.  
It should have a subject like "New team for [your language name] ([your language code])"
and contain the name and the email of the person who is the new coordinator for that language
(And if one exists, the URL of the Team's page).  
(The webmaster will add this team to this page shortly after)

### Project Web Pages and Mailing Lists

We would like to encourage translation teams to have web pages,
even if they are simple ones, and mailing lists. For many teams,
the easiest and best way to do this is to use
Savannah. Savannah
will allow you to create a project for your team, providing you
use it to advance open source development, which you are doing.
It will give you a place to set up web pages, have a mailing list,
and place files for people to download. You can even set up CVS
if you find that helpful. It also allows you to give access to
multiple team members so you can each update the web pages,
downloadable files, etc.

### Errors

If there are any errors in the contact/web address of a team
please write to the mailing list.

Navigation

- Home
- News
- Getting Involved
- Release Planning
- Architecture
- Projects
  - Accessibility
  - Bugsquad
  - Documentation
  - Packaging
  - Translation
    - Tasks
    - Status
    - Resources
    - Teams
    - Joining
    - Contacting
    - Related Projects
  - Usability
  - Webhackers
- Documentation
- Feature Articles
- Development Tools
- External Resources

Users ·
**Developers** ·
Bonsai ·
ViewCVS ·
FTP ·
Bugzilla ·
Software Map ·
Contact

© 2003-2004 by The GNOME Project
  
Last modified 2005/07/26 22:13:21 UTC.
  
Optimised
for standards.
Hosted by Red Hat.
