# Supplementary material for: rDock: A Fast, Versatile and Open Source Program for Docking Ligands to Proteins and Nucleic Acids
Source: PLoS Comput Biol. 2014 Apr 10;10(4):e1003571. doi: 10.1371/journal.pcbi.1003571 (PMC3983074; doi:10.1371/journal.pcbi.1003571)
Supplement: Software S1 — Compressed file with the source code of the rDock software for ligand docking to Proteins and Nucleic Acids. (GZ) [file pcbi.1003571.s010.gz › rDock_2013.1_src/gettext-0.18.tar.gz!/gettext-0.18/gettext-tools/man/xgettext.1.html]

XGETTEXT


# XGETTEXT

NAME  
SYNOPSIS  
DESCRIPTION  
AUTHOR  
REPORTING BUGS  
COPYRIGHT  
SEE ALSO  


---


## NAME

|  |  |
| --- | --- |
|  | xgettext − extract gettext strings from source |

## SYNOPSIS

|  |  |
| --- | --- |
|  | **xgettext** [*OPTION*] [*INPUTFILE*]... |

## DESCRIPTION

|  |  |
| --- | --- |
|  | Extract translatable strings from given input files.  Mandatory arguments to long options are mandatory for short options too. Similarly for optional arguments. |

|  |  |
| --- | --- |
|  | **Input file location:** |

|  |  |
| --- | --- |
|  | INPUTFILE ... |

|  |  |
| --- | --- |
|  | input files |

|  |  |
| --- | --- |
|  | **−f**, **−−files−from**=*FILE* |

|  |  |
| --- | --- |
|  | get list of input files from FILE |

|  |  |
| --- | --- |
|  | **−D**, **−−directory**=*DIRECTORY* |

|  |  |
| --- | --- |
|  | add DIRECTORY to list for input files search |

|  |  |
| --- | --- |
|  | If input file is -, standard input is read. |

|  |  |
| --- | --- |
|  | **Output file location:** |

|  |  |
| --- | --- |
|  | **−d**, **−−default−domain**=*NAME* |

|  |  |
| --- | --- |
|  | use NAME.po for output (instead of messages.po) |

|  |  |
| --- | --- |
|  | **−o**, **−−output**=*FILE* |

|  |  |
| --- | --- |
|  | write output to specified file |

|  |  |
| --- | --- |
|  | **−p**, **−−output−dir**=*DIR* |

|  |  |
| --- | --- |
|  | output files will be placed in directory DIR |

|  |  |
| --- | --- |
|  | If output file is -, output is written to standard output. |

|  |  |
| --- | --- |
|  | **Choice of input file language:** |

|  |  |
| --- | --- |
|  | **−L**, **−−language**=*NAME* |

|  |  |
| --- | --- |
|  | recognise the specified language (C, C++, ObjectiveC, PO, Shell, Python, Lisp, EmacsLisp, librep, Scheme, Smalltalk, Java, JavaProperties, C#, awk, YCP, Tcl, Perl, PHP, GCC-source, NXStringTable, RST, Glade) |

|  |  |
| --- | --- |
|  | **−C**, **−−c**++ |

|  |  |
| --- | --- |
|  | shorthand for **−−language**=*C*++ |

|  |  |
| --- | --- |
|  | By default the language is guessed depending on the input file name extension. |

|  |  |
| --- | --- |
|  | **Input file interpretation:** |

|  |  |
| --- | --- |
|  | **−−from−code**=*NAME* |

|  |  |
| --- | --- |
|  | encoding of input files (except for Python, Tcl, Glade) |

|  |  |
| --- | --- |
|  | By default the input files are assumed to be in ASCII. |

|  |  |
| --- | --- |
|  | **Operation mode:** |

|  |  |
| --- | --- |
|  | **−j**, **−−join−existing** |

|  |  |
| --- | --- |
|  | join messages with existing file |

|  |  |
| --- | --- |
|  | **−x**, **−−exclude−file**=*FILE*.po |

|  |  |
| --- | --- |
|  | entries from FILE.po are not extracted |

|  |  |
| --- | --- |
|  | **−cTAG**, **−−add−comments**=*TAG* |

|  |  |
| --- | --- |
|  | place comment blocks starting with TAG and preceding keyword lines in output file |

|  |  |
| --- | --- |
|  | **−c**, **−−add−comments** |

|  |  |
| --- | --- |
|  | place all comment blocks preceding keyword lines in output file |

|  |  |
| --- | --- |
|  | **Language specific options:** |

|  |  |
| --- | --- |
|  | **−a**, **−−extract−all** |

|  |  |
| --- | --- |
|  | extract all strings (only languages C, C++, ObjectiveC, Shell, Python, Lisp, EmacsLisp, librep, Scheme, Java, C#, awk, Tcl, Perl, PHP, GCC-source, Glade) |

|  |  |
| --- | --- |
|  | **−kWORD**, **−−keyword**=*WORD* |

|  |  |
| --- | --- |
|  | look for WORD as an additional keyword |

|  |  |
| --- | --- |
|  | **−k**, **−−keyword** |

|  |  |
| --- | --- |
|  | do not to use default keywords (only languages C, C++, ObjectiveC, Shell, Python, Lisp, EmacsLisp, librep, Scheme, Java, C#, awk, Tcl, Perl, PHP, GCC-source, Glade) |

|  |  |
| --- | --- |
|  | **−−flag**=*WORD*:ARG:FLAG |

|  |  |
| --- | --- |
|  | additional flag for strings inside the argument number ARG of keyword WORD |

|  |  |
| --- | --- |
|  | (only languages C, C++, ObjectiveC, Shell, Python, Lisp, EmacsLisp, librep, Scheme, Java, C#, awk, YCP, Tcl, Perl, PHP, GCC-source) |

|  |  |
| --- | --- |
|  | **−T**, **−−trigraphs** |

|  |  |
| --- | --- |
|  | understand ANSI C trigraphs for input (only languages C, C++, ObjectiveC) |

|  |  |  |  |  |  |  |  |
| --- | --- | --- | --- | --- | --- | --- | --- |
|  | **−−qt** |  | recognize Qt format strings (only language C++) ||  | **−−kde** |  | recognize KDE 4 format strings (only language C++) |

|  |  |
| --- | --- |
|  | **−−boost** |

|  |  |
| --- | --- |
|  | recognize Boost format strings (only language C++) |

|  |  |
| --- | --- |
|  | **−−debug** |

|  |  |
| --- | --- |
|  | more detailed formatstring recognition result |

|  |  |
| --- | --- |
|  | **Output details:** |

|  |  |
| --- | --- |
|  | **−−color** |

|  |  |
| --- | --- |
|  | use colors and other text attributes always |

|  |  |
| --- | --- |
|  | **−−color**=*WHEN* |

|  |  |
| --- | --- |
|  | use colors and other text attributes if WHEN. WHEN may be ’always’, ’never’, ’auto’, or ’html’. |

|  |  |
| --- | --- |
|  | **−−style**=*STYLEFILE* |

|  |  |
| --- | --- |
|  | specify CSS style rule file for **−−color** |

|  |  |
| --- | --- |
|  | **−e**, **−−no−escape** |

|  |  |
| --- | --- |
|  | do not use C escapes in output (default) |

|  |  |
| --- | --- |
|  | **−E**, **−−escape** |

|  |  |
| --- | --- |
|  | use C escapes in output, no extended chars |

|  |  |
| --- | --- |
|  | **−−force−po** |

|  |  |
| --- | --- |
|  | write PO file even if empty |

|  |  |
| --- | --- |
|  | **−i**, **−−indent** |

|  |  |
| --- | --- |
|  | write the .po file using indented style |

|  |  |
| --- | --- |
|  | **−−no−location** |

|  |  |
| --- | --- |
|  | do not write ’#: filename:line’ lines |

|  |  |
| --- | --- |
|  | **−n**, **−−add−location** |

|  |  |
| --- | --- |
|  | generate ’#: filename:line’ lines (default) |

|  |  |
| --- | --- |
|  | **−−strict** |

|  |  |
| --- | --- |
|  | write out strict Uniforum conforming .po file |

|  |  |
| --- | --- |
|  | **−−properties−output** |

|  |  |
| --- | --- |
|  | write out a Java .properties file |

|  |  |
| --- | --- |
|  | **−−stringtable−output** |

|  |  |
| --- | --- |
|  | write out a NeXTstep/GNUstep .strings file |

|  |  |
| --- | --- |
|  | **−w**, **−−width**=*NUMBER* |

|  |  |
| --- | --- |
|  | set output page width |

|  |  |
| --- | --- |
|  | **−−no−wrap** |

|  |  |
| --- | --- |
|  | do not break long message lines, longer than the output page width, into several lines |

|  |  |
| --- | --- |
|  | **−s**, **−−sort−output** |

|  |  |
| --- | --- |
|  | generate sorted output |

|  |  |
| --- | --- |
|  | **−F**, **−−sort−by−file** |

|  |  |
| --- | --- |
|  | sort output by file location |

|  |  |
| --- | --- |
|  | **−−omit−header** |

|  |  |
| --- | --- |
|  | don’t write header with ‘msgid ""’ entry |

|  |  |
| --- | --- |
|  | **−−copyright−holder**=*STRING* |

|  |  |
| --- | --- |
|  | set copyright holder in output |

|  |  |
| --- | --- |
|  | **−−foreign−user** |

|  |  |
| --- | --- |
|  | omit FSF copyright in output for foreign user |

|  |  |
| --- | --- |
|  | **−−package−name**=*PACKAGE* |

|  |  |
| --- | --- |
|  | set package name in output |

|  |  |
| --- | --- |
|  | **−−package−version**=*VERSION* |

|  |  |
| --- | --- |
|  | set package version in output |

|  |  |
| --- | --- |
|  | **−−msgid−bugs−address**=*EMAIL*@ADDRESS |

|  |  |
| --- | --- |
|  | set report address for msgid bugs |

|  |  |
| --- | --- |
|  | **−m[STRING]**, **−−msgstr−prefix**[=*STRING*] |

|  |  |
| --- | --- |
|  | use STRING or "" as prefix for msgstr values |

|  |  |
| --- | --- |
|  | **−M[STRING]**, **−−msgstr−suffix**[=*STRING*] |

|  |  |
| --- | --- |
|  | use STRING or "" as suffix for msgstr values |

|  |  |
| --- | --- |
|  | **Informative output:** |

|  |  |
| --- | --- |
|  | **−h**, **−−help** |

|  |  |
| --- | --- |
|  | display this help and exit |

|  |  |
| --- | --- |
|  | **−V**, **−−version** |

|  |  |
| --- | --- |
|  | output version information and exit |

## AUTHOR

|  |  |
| --- | --- |
|  | Written by Ulrich Drepper. |

## REPORTING BUGS

|  |  |
| --- | --- |
|  | Report bugs to <bug-gnu-gettext@gnu.org>. |

## COPYRIGHT

|  |  |
| --- | --- |
|  | Copyright © 1995-1998, 2000-2010 Free Software Foundation, Inc. License GPLv3+: GNU GPL version 3 or later <http://gnu.org/licenses/gpl.html>  This is free software: you are free to change and redistribute it. There is NO WARRANTY, to the extent permitted by law. |

## SEE ALSO

|  |  |
| --- | --- |
|  | The full documentation for **xgettext** is maintained as a Texinfo manual. If the **info** and **xgettext** programs are properly installed at your site, the command |

|  |  |
| --- | --- |
|  | **info xgettext** |

|  |  |
| --- | --- |
|  | should give you access to the complete manual. |

---
