# Supplementary material for: rDock: A Fast, Versatile and Open Source Program for Docking Ligands to Proteins and Nucleic Acids
Source: PLoS Comput Biol. 2014 Apr 10;10(4):e1003571. doi: 10.1371/journal.pcbi.1003571 (PMC3983074; doi:10.1371/journal.pcbi.1003571)
Supplement: Software S1 — Compressed file with the source code of the rDock software for ligand docking to Proteins and Nucleic Acids. (GZ) [file pcbi.1003571.s010.gz › rDock_2013.1_src/gettext-0.18.tar.gz!/gettext-0.18/gettext-tools/man/msgfmt.1.html]

MSGFMT


# MSGFMT

NAME  
SYNOPSIS  
DESCRIPTION  
AUTHOR  
REPORTING BUGS  
COPYRIGHT  
SEE ALSO  


---


## NAME

|  |  |
| --- | --- |
|  | msgfmt − compile message catalog to binary format |

## SYNOPSIS

|  |  |
| --- | --- |
|  | **msgfmt** [*OPTION*] *filename.po* ... |

## DESCRIPTION

|  |  |
| --- | --- |
|  | Generate binary message catalog from textual translation description.  Mandatory arguments to long options are mandatory for short options too. Similarly for optional arguments. |

|  |  |
| --- | --- |
|  | **Input file location:** |

|  |  |
| --- | --- |
|  | filename.po ... |

|  |  |
| --- | --- |
|  | input files |

|  |  |
| --- | --- |
|  | **−D**, **−−directory**=*DIRECTORY* |

|  |  |
| --- | --- |
|  | add DIRECTORY to list for input files search |

|  |  |
| --- | --- |
|  | If input file is -, standard input is read. |

|  |  |
| --- | --- |
|  | **Operation mode:** |

|  |  |
| --- | --- |
|  | **−j**, **−−java** |

|  |  |
| --- | --- |
|  | Java mode: generate a Java ResourceBundle class |

|  |  |
| --- | --- |
|  | **−−java2** |

|  |  |
| --- | --- |
|  | like **−−java**, and assume Java2 (JDK 1.2 or higher) |

|  |  |
| --- | --- |
|  | **−−csharp** |

|  |  |
| --- | --- |
|  | C# mode: generate a .NET .dll file |

|  |  |
| --- | --- |
|  | **−−csharp−resources** |

|  |  |
| --- | --- |
|  | C# resources mode: generate a .NET .resources file |

|  |  |  |  |  |  |  |  |  |  |
| --- | --- | --- | --- | --- | --- | --- | --- | --- | --- |
|  | **−−tcl** |  | Tcl mode: generate a tcl/msgcat .msg file |  ||  | **−−qt** |  | Qt mode: generate a Qt .qm file |  |

|  |  |
| --- | --- |
|  | **Output file location:** |

|  |  |
| --- | --- |
|  | **−o**, **−−output−file**=*FILE* |

|  |  |
| --- | --- |
|  | write output to specified file |

|  |  |
| --- | --- |
|  | **−−strict** |

|  |  |
| --- | --- |
|  | enable strict Uniforum mode |

|  |  |
| --- | --- |
|  | If output file is -, output is written to standard output. |

|  |  |
| --- | --- |
|  | **Output file location in Java mode:** |

|  |  |
| --- | --- |
|  | **−r**, **−−resource**=*RESOURCE* |

|  |  |
| --- | --- |
|  | resource name |

|  |  |
| --- | --- |
|  | **−l**, **−−locale**=*LOCALE* |

|  |  |
| --- | --- |
|  | locale name, either language or language\_COUNTRY |

|  |  |
| --- | --- |
|  | **−d** DIRECTORY |

|  |  |
| --- | --- |
|  | base directory of classes directory hierarchy |

|  |  |
| --- | --- |
|  | The class name is determined by appending the locale name to the resource name, separated with an underscore. The **−d** option is mandatory. The class is written under the specified directory. |

|  |  |
| --- | --- |
|  | **Output file location in C# mode:** |

|  |  |
| --- | --- |
|  | **−r**, **−−resource**=*RESOURCE* |

|  |  |
| --- | --- |
|  | resource name |

|  |  |
| --- | --- |
|  | **−l**, **−−locale**=*LOCALE* |

|  |  |
| --- | --- |
|  | locale name, either language or language\_COUNTRY |

|  |  |
| --- | --- |
|  | **−d** DIRECTORY |

|  |  |
| --- | --- |
|  | base directory for locale dependent .dll files |

|  |  |
| --- | --- |
|  | The **−l** and **−d** options are mandatory. The .dll file is written in a subdirectory of the specified directory whose name depends on the locale. |

|  |  |
| --- | --- |
|  | **Output file location in Tcl mode:** |

|  |  |
| --- | --- |
|  | **−l**, **−−locale**=*LOCALE* |

|  |  |
| --- | --- |
|  | locale name, either language or language\_COUNTRY |

|  |  |
| --- | --- |
|  | **−d** DIRECTORY |

|  |  |
| --- | --- |
|  | base directory of .msg message catalogs |

|  |  |
| --- | --- |
|  | The **−l** and **−d** options are mandatory. The .msg file is written in the specified directory. |

|  |  |
| --- | --- |
|  | **Input file syntax:** |

|  |  |
| --- | --- |
|  | **−P**, **−−properties−input** |

|  |  |
| --- | --- |
|  | input files are in Java .properties syntax |

|  |  |
| --- | --- |
|  | **−−stringtable−input** |

|  |  |
| --- | --- |
|  | input files are in NeXTstep/GNUstep .strings syntax |

|  |  |
| --- | --- |
|  | **Input file interpretation:** |

|  |  |
| --- | --- |
|  | **−c**, **−−check** |

|  |  |
| --- | --- |
|  | perform all the checks implied by **−−check−format**, **−−check−header**, **−−check−domain** |

|  |  |
| --- | --- |
|  | **−−check−format** |

|  |  |
| --- | --- |
|  | check language dependent format strings |

|  |  |
| --- | --- |
|  | **−−check−header** |

|  |  |
| --- | --- |
|  | verify presence and contents of the header entry |

|  |  |
| --- | --- |
|  | **−−check−domain** |

|  |  |
| --- | --- |
|  | check for conflicts between domain directives and the **−−output−file** option |

|  |  |
| --- | --- |
|  | **−C**, **−−check−compatibility** |

|  |  |
| --- | --- |
|  | check that GNU msgfmt behaves like X/Open msgfmt |

|  |  |
| --- | --- |
|  | **−−check−accelerators**[=*CHAR*] |

|  |  |
| --- | --- |
|  | check presence of keyboard accelerators for menu items |

|  |  |
| --- | --- |
|  | **−f**, **−−use−fuzzy** |

|  |  |
| --- | --- |
|  | use fuzzy entries in output |

|  |  |
| --- | --- |
|  | **Output details:** |

|  |  |
| --- | --- |
|  | **−a**, **−−alignment**=*NUMBER* |

|  |  |
| --- | --- |
|  | align strings to NUMBER bytes (default: 1) |

|  |  |
| --- | --- |
|  | **−−no−hash** |

|  |  |
| --- | --- |
|  | binary file will not include the hash table |

|  |  |
| --- | --- |
|  | **Informative output:** |

|  |  |
| --- | --- |
|  | **−h**, **−−help** |

|  |  |
| --- | --- |
|  | display this help and exit |

|  |  |
| --- | --- |
|  | **−V**, **−−version** |

|  |  |
| --- | --- |
|  | output version information and exit |

|  |  |
| --- | --- |
|  | **−−statistics** |

|  |  |
| --- | --- |
|  | print statistics about translations |

|  |  |
| --- | --- |
|  | **−v**, **−−verbose** |

|  |  |
| --- | --- |
|  | increase verbosity level |

## AUTHOR

|  |  |
| --- | --- |
|  | Written by Ulrich Drepper. |

## REPORTING BUGS

|  |  |
| --- | --- |
|  | Report bugs to <bug-gnu-gettext@gnu.org>. |

## COPYRIGHT

|  |  |
| --- | --- |
|  | Copyright © 1995-1998, 2000-2010 Free Software Foundation, Inc. License GPLv3+: GNU GPL version 3 or later <http://gnu.org/licenses/gpl.html>  This is free software: you are free to change and redistribute it. There is NO WARRANTY, to the extent permitted by law. |

## SEE ALSO

|  |  |
| --- | --- |
|  | The full documentation for **msgfmt** is maintained as a Texinfo manual. If the **info** and **msgfmt** programs are properly installed at your site, the command |

|  |  |
| --- | --- |
|  | **info msgfmt** |

|  |  |
| --- | --- |
|  | should give you access to the complete manual. |

---
