# Supplementary material for: rDock: A Fast, Versatile and Open Source Program for Docking Ligands to Proteins and Nucleic Acids
Source: PLoS Comput Biol. 2014 Apr 10;10(4):e1003571. doi: 10.1371/journal.pcbi.1003571 (PMC3983074; doi:10.1371/journal.pcbi.1003571)
Supplement: Software S1 — Compressed file with the source code of the rDock software for ligand docking to Proteins and Nucleic Acids. (GZ) [file pcbi.1003571.s010.gz › rDock_2013.1_src/gettext-0.18.tar.gz!/gettext-0.18/gettext-tools/man/msgmerge.1.html]

MSGMERGE


# MSGMERGE

NAME  
SYNOPSIS  
DESCRIPTION  
AUTHOR  
REPORTING BUGS  
COPYRIGHT  
SEE ALSO  


---


## NAME

|  |  |
| --- | --- |
|  | msgmerge − merge message catalog and template |

## SYNOPSIS

|  |  |
| --- | --- |
|  | **msgmerge** [*OPTION*] *def.po ref.pot* |

## DESCRIPTION

|  |  |
| --- | --- |
|  | Merges two Uniforum style .po files together. The def.po file is an existing PO file with translations which will be taken over to the newly created file as long as they still match; comments will be preserved, but extracted comments and file positions will be discarded. The ref.pot file is the last created PO file with up-to-date source references but old translations, or a PO Template file (generally created by xgettext); any translations or comments in the file will be discarded, however dot comments and file positions will be preserved. Where an exact match cannot be found, fuzzy matching is used to produce better results.  Mandatory arguments to long options are mandatory for short options too. |

|  |  |
| --- | --- |
|  | **Input file location:** |

|  |  |  |  |  |
| --- | --- | --- | --- | --- |
|  | def.po |  | translations referring to old sources |  |

|  |  |
| --- | --- |
|  | ref.pot |

|  |  |
| --- | --- |
|  | references to new sources |

|  |  |
| --- | --- |
|  | **−D**, **−−directory**=*DIRECTORY* |

|  |  |
| --- | --- |
|  | add DIRECTORY to list for input files search |

|  |  |
| --- | --- |
|  | **−C**, **−−compendium**=*FILE* |

|  |  |
| --- | --- |
|  | additional library of message translations, may be specified more than once |

|  |  |
| --- | --- |
|  | **Operation mode:** |

|  |  |
| --- | --- |
|  | **−U**, **−−update** |

|  |  |
| --- | --- |
|  | update def.po, do nothing if def.po already up to date |

|  |  |
| --- | --- |
|  | **Output file location:** |

|  |  |
| --- | --- |
|  | **−o**, **−−output−file**=*FILE* |

|  |  |
| --- | --- |
|  | write output to specified file |

|  |  |
| --- | --- |
|  | The results are written to standard output if no output file is specified or if it is -.  Output file location in update mode: The result is written back to def.po. |

|  |  |
| --- | --- |
|  | **−−backup**=*CONTROL* |

|  |  |
| --- | --- |
|  | make a backup of def.po |

|  |  |
| --- | --- |
|  | **−−suffix**=*SUFFIX* |

|  |  |
| --- | --- |
|  | override the usual backup suffix |

|  |  |
| --- | --- |
|  | The version control method may be selected via the **−−backup** option or through the VERSION\_CONTROL environment variable. Here are the values: |

|  |  |
| --- | --- |
|  | none, off |

|  |  |
| --- | --- |
|  | never make backups (even if **−−backup** is given) |

|  |  |
| --- | --- |
|  | numbered, t |

|  |  |
| --- | --- |
|  | make numbered backups |

|  |  |
| --- | --- |
|  | existing, nil |

|  |  |
| --- | --- |
|  | numbered if numbered backups exist, simple otherwise |

|  |  |
| --- | --- |
|  | simple, never |

|  |  |
| --- | --- |
|  | always make simple backups |

|  |  |
| --- | --- |
|  | The backup suffix is ‘~’, unless set with **−−suffix** or the SIMPLE\_BACKUP\_SUFFIX environment variable. |

|  |  |
| --- | --- |
|  | **Operation modifiers:** |

|  |  |
| --- | --- |
|  | **−m**, **−−multi−domain** |

|  |  |
| --- | --- |
|  | apply ref.pot to each of the domains in def.po |

|  |  |
| --- | --- |
|  | **−N**, **−−no−fuzzy−matching** |

|  |  |
| --- | --- |
|  | do not use fuzzy matching |

|  |  |
| --- | --- |
|  | **−−previous** |

|  |  |
| --- | --- |
|  | keep previous msgids of translated messages |

|  |  |
| --- | --- |
|  | **Input file syntax:** |

|  |  |
| --- | --- |
|  | **−P**, **−−properties−input** |

|  |  |
| --- | --- |
|  | input files are in Java .properties syntax |

|  |  |
| --- | --- |
|  | **−−stringtable−input** |

|  |  |
| --- | --- |
|  | input files are in NeXTstep/GNUstep .strings syntax |

|  |  |
| --- | --- |
|  | **Output details:** |

|  |  |
| --- | --- |
|  | **−−lang**=*CATALOGNAME* |

|  |  |
| --- | --- |
|  | set ’Language’ field in the header entry |

|  |  |
| --- | --- |
|  | **−−color** |

|  |  |
| --- | --- |
|  | use colors and other text attributes always |

|  |  |
| --- | --- |
|  | **−−color**=*WHEN* |

|  |  |
| --- | --- |
|  | use colors and other text attributes if WHEN. WHEN may be ’always’, ’never’, ’auto’, or ’html’. |

|  |  |
| --- | --- |
|  | **−−style**=*STYLEFILE* |

|  |  |
| --- | --- |
|  | specify CSS style rule file for **−−color** |

|  |  |
| --- | --- |
|  | **−e**, **−−no−escape** |

|  |  |
| --- | --- |
|  | do not use C escapes in output (default) |

|  |  |
| --- | --- |
|  | **−E**, **−−escape** |

|  |  |
| --- | --- |
|  | use C escapes in output, no extended chars |

|  |  |
| --- | --- |
|  | **−−force−po** |

|  |  |
| --- | --- |
|  | write PO file even if empty |

|  |  |
| --- | --- |
|  | **−i**, **−−indent** |

|  |  |
| --- | --- |
|  | indented output style |

|  |  |
| --- | --- |
|  | **−−no−location** |

|  |  |
| --- | --- |
|  | suppress ’#: filename:line’ lines |

|  |  |
| --- | --- |
|  | **−−add−location** |

|  |  |
| --- | --- |
|  | preserve ’#: filename:line’ lines (default) |

|  |  |
| --- | --- |
|  | **−−strict** |

|  |  |
| --- | --- |
|  | strict Uniforum output style |

|  |  |
| --- | --- |
|  | **−p**, **−−properties−output** |

|  |  |
| --- | --- |
|  | write out a Java .properties file |

|  |  |
| --- | --- |
|  | **−−stringtable−output** |

|  |  |
| --- | --- |
|  | write out a NeXTstep/GNUstep .strings file |

|  |  |
| --- | --- |
|  | **−w**, **−−width**=*NUMBER* |

|  |  |
| --- | --- |
|  | set output page width |

|  |  |
| --- | --- |
|  | **−−no−wrap** |

|  |  |
| --- | --- |
|  | do not break long message lines, longer than the output page width, into several lines |

|  |  |
| --- | --- |
|  | **−s**, **−−sort−output** |

|  |  |
| --- | --- |
|  | generate sorted output |

|  |  |
| --- | --- |
|  | **−F**, **−−sort−by−file** |

|  |  |
| --- | --- |
|  | sort output by file location |

|  |  |
| --- | --- |
|  | **Informative output:** |

|  |  |
| --- | --- |
|  | **−h**, **−−help** |

|  |  |
| --- | --- |
|  | display this help and exit |

|  |  |
| --- | --- |
|  | **−V**, **−−version** |

|  |  |
| --- | --- |
|  | output version information and exit |

|  |  |
| --- | --- |
|  | **−v**, **−−verbose** |

|  |  |
| --- | --- |
|  | increase verbosity level |

|  |  |
| --- | --- |
|  | **−q**, **−−quiet**, **−−silent** |

|  |  |
| --- | --- |
|  | suppress progress indicators |

## AUTHOR

|  |  |
| --- | --- |
|  | Written by Peter Miller. |

## REPORTING BUGS

|  |  |
| --- | --- |
|  | Report bugs to <bug-gnu-gettext@gnu.org>. |

## COPYRIGHT

|  |  |
| --- | --- |
|  | Copyright © 1995-1998, 2000-2010 Free Software Foundation, Inc. License GPLv3+: GNU GPL version 3 or later <http://gnu.org/licenses/gpl.html>  This is free software: you are free to change and redistribute it. There is NO WARRANTY, to the extent permitted by law. |

## SEE ALSO

|  |  |
| --- | --- |
|  | The full documentation for **msgmerge** is maintained as a Texinfo manual. If the **info** and **msgmerge** programs are properly installed at your site, the command |

|  |  |
| --- | --- |
|  | **info msgmerge** |

|  |  |
| --- | --- |
|  | should give you access to the complete manual. |

---
