# Supplementary material for: rDock: A Fast, Versatile and Open Source Program for Docking Ligands to Proteins and Nucleic Acids
Source: PLoS Comput Biol. 2014 Apr 10;10(4):e1003571. doi: 10.1371/journal.pcbi.1003571 (PMC3983074; doi:10.1371/journal.pcbi.1003571)
Supplement: Software S1 — Compressed file with the source code of the rDock software for ligand docking to Proteins and Nucleic Acids. (GZ) [file pcbi.1003571.s010.gz › rDock_2013.1_src/gettext-0.18.tar.gz!/gettext-0.18/gettext-tools/man/autopoint.1.html]

AUTOPOINT


# AUTOPOINT

NAME  
SYNOPSIS  
DESCRIPTION  
OPTIONS  
AUTHOR  
REPORTING BUGS  
SEE ALSO  


---


## NAME

|  |  |
| --- | --- |
|  | autopoint − copies standard gettext infrastructure |

## SYNOPSIS

|  |  |
| --- | --- |
|  | **autopoint** [*OPTION*]... |

## DESCRIPTION

|  |  |
| --- | --- |
|  | Copies standard gettext infrastructure files into a source package. |

## OPTIONS

|  |  |  |  |  |
| --- | --- | --- | --- | --- |
|  | **−−help** |  | print this help and exit |  |

|  |  |
| --- | --- |
|  | **−−version** |

|  |  |
| --- | --- |
|  | print version information and exit |

|  |  |
| --- | --- |
|  | **−f**, **−−force** |

|  |  |
| --- | --- |
|  | force overwriting of files that already exist |

|  |  |
| --- | --- |
|  | **−n**, **−−dry−run** |

|  |  |
| --- | --- |
|  | print modifications but don’t perform them |

## AUTHOR

|  |  |
| --- | --- |
|  | Written by Bruno Haible |

## REPORTING BUGS

|  |  |
| --- | --- |
|  | Report bugs to <bug-gnu-gettext@gnu.org>.  Uses a versions archive in cvs format. Copyright (C) 2002-2010 Free Software Foundation, Inc. License GPLv3+: GNU GPL version 3 or later <http://gnu.org/licenses/gpl.html> This is free software: you are free to change and redistribute it. There is NO WARRANTY, to the extent permitted by law. |

## SEE ALSO

|  |  |
| --- | --- |
|  | The full documentation for **autopoint** is maintained as a Texinfo manual. If the **info** and **autopoint** programs are properly installed at your site, the command |

|  |  |
| --- | --- |
|  | **info autopoint** |

|  |  |
| --- | --- |
|  | should give you access to the complete manual. |

---
