# Supplementary material for: rDock: A Fast, Versatile and Open Source Program for Docking Ligands to Proteins and Nucleic Acids
Source: PLoS Comput Biol. 2014 Apr 10;10(4):e1003571. doi: 10.1371/journal.pcbi.1003571 (PMC3983074; doi:10.1371/journal.pcbi.1003571)
Supplement: Software S1 — Compressed file with the source code of the rDock software for ligand docking to Proteins and Nucleic Acids. (GZ) [file pcbi.1003571.s010.gz › rDock_2013.1_src/gettext-0.18.tar.gz!/gettext-0.18/gettext-tools/man/msggrep.1.html]

MSGGREP


# MSGGREP

NAME  
SYNOPSIS  
DESCRIPTION  
AUTHOR  
REPORTING BUGS  
COPYRIGHT  
SEE ALSO  


---


## NAME

|  |  |
| --- | --- |
|  | msggrep − pattern matching on message catalog |

## SYNOPSIS

|  |  |
| --- | --- |
|  | **msggrep** [*OPTION*] [*INPUTFILE*] |

## DESCRIPTION

|  |  |
| --- | --- |
|  | Extracts all messages of a translation catalog that match a given pattern or belong to some given source files.  Mandatory arguments to long options are mandatory for short options too. |

|  |  |
| --- | --- |
|  | **Input file location:** |

|  |  |
| --- | --- |
|  | INPUTFILE |

|  |  |
| --- | --- |
|  | input PO file |

|  |  |
| --- | --- |
|  | **−D**, **−−directory**=*DIRECTORY* |

|  |  |
| --- | --- |
|  | add DIRECTORY to list for input files search |

|  |  |
| --- | --- |
|  | If no input file is given or if it is -, standard input is read. |

|  |  |
| --- | --- |
|  | **Output file location:** |

|  |  |
| --- | --- |
|  | **−o**, **−−output−file**=*FILE* |

|  |  |
| --- | --- |
|  | write output to specified file |

|  |  |
| --- | --- |
|  | The results are written to standard output if no output file is specified or if it is -. |

|  |  |
| --- | --- |
|  | **Message selection:** |

|  |  |
| --- | --- |
|  | [-N SOURCEFILE]... [-M DOMAINNAME]... [-J MSGCTXT-PATTERN] [-K MSGID-PATTERN] [-T MSGSTR-PATTERN] [-C COMMENT-PATTERN] [-X EXTRACTED-COMMENT-PATTERN] |

|  |  |
| --- | --- |
|  | A message is selected if it comes from one of the specified source files, or if it comes from one of the specified domains, or if **−J** is given and its context (msgctxt) matches MSGCTXT-PATTERN, or if **−K** is given and its key (msgid or msgid\_plural) matches MSGID-PATTERN, or if **−T** is given and its translation (msgstr) matches MSGSTR-PATTERN, or if **−C** is given and the translator’s comment matches COMMENT-PATTERN, or if **−X** is given and the extracted comment matches EXTRACTED-COMMENT-PATTERN.  When more than one selection criterion is specified, the set of selected messages is the union of the selected messages of each criterion.  MSGCTXT-PATTERN or MSGID-PATTERN or MSGSTR-PATTERN or COMMENT-PATTERN or EXTRACTED-COMMENT-PATTERN syntax: |

|  |  |
| --- | --- |
|  | [-E \| **−F]** [-e PATTERN \| **−f** FILE]... |

|  |  |
| --- | --- |
|  | PATTERNs are basic regular expressions by default, or extended regular expressions if **−E** is given, or fixed strings if **−F** is given. |

|  |  |
| --- | --- |
|  | **−N**, **−−location**=*SOURCEFILE* |

|  |  |
| --- | --- |
|  | select messages extracted from SOURCEFILE |

|  |  |
| --- | --- |
|  | **−M**, **−−domain**=*DOMAINNAME* |

|  |  |
| --- | --- |
|  | select messages belonging to domain DOMAINNAME |

|  |  |
| --- | --- |
|  | **−J**, **−−msgctxt** |

|  |  |
| --- | --- |
|  | start of patterns for the msgctxt |

|  |  |
| --- | --- |
|  | **−K**, **−−msgid** |

|  |  |
| --- | --- |
|  | start of patterns for the msgid |

|  |  |
| --- | --- |
|  | **−T**, **−−msgstr** |

|  |  |
| --- | --- |
|  | start of patterns for the msgstr |

|  |  |
| --- | --- |
|  | **−C**, **−−comment** |

|  |  |
| --- | --- |
|  | start of patterns for the translator’s comment |

|  |  |
| --- | --- |
|  | **−X**, **−−extracted−comment** |

|  |  |
| --- | --- |
|  | start of patterns for the extracted comment |

|  |  |
| --- | --- |
|  | **−E**, **−−extended−regexp** |

|  |  |
| --- | --- |
|  | PATTERN is an extended regular expression |

|  |  |
| --- | --- |
|  | **−F**, **−−fixed−strings** |

|  |  |
| --- | --- |
|  | PATTERN is a set of newline-separated strings |

|  |  |
| --- | --- |
|  | **−e**, **−−regexp**=*PATTERN* |

|  |  |
| --- | --- |
|  | use PATTERN as a regular expression |

|  |  |
| --- | --- |
|  | **−f**, **−−file**=*FILE* |

|  |  |
| --- | --- |
|  | obtain PATTERN from FILE |

|  |  |
| --- | --- |
|  | **−i**, **−−ignore−case** |

|  |  |
| --- | --- |
|  | ignore case distinctions |

|  |  |
| --- | --- |
|  | **−v**, **−−invert−match** |

|  |  |
| --- | --- |
|  | output only the messages that do not match any selection criterion |

|  |  |
| --- | --- |
|  | **Input file syntax:** |

|  |  |
| --- | --- |
|  | **−P**, **−−properties−input** |

|  |  |
| --- | --- |
|  | input file is in Java .properties syntax |

|  |  |
| --- | --- |
|  | **−−stringtable−input** |

|  |  |
| --- | --- |
|  | input file is in NeXTstep/GNUstep .strings syntax |

|  |  |
| --- | --- |
|  | **Output details:** |

|  |  |
| --- | --- |
|  | **−−color** |

|  |  |
| --- | --- |
|  | use colors and other text attributes always |

|  |  |
| --- | --- |
|  | **−−color**=*WHEN* |

|  |  |
| --- | --- |
|  | use colors and other text attributes if WHEN. WHEN may be ’always’, ’never’, ’auto’, or ’html’. |

|  |  |
| --- | --- |
|  | **−−style**=*STYLEFILE* |

|  |  |
| --- | --- |
|  | specify CSS style rule file for **−−color** |

|  |  |
| --- | --- |
|  | **−−no−escape** |

|  |  |
| --- | --- |
|  | do not use C escapes in output (default) |

|  |  |
| --- | --- |
|  | **−−escape** |

|  |  |
| --- | --- |
|  | use C escapes in output, no extended chars |

|  |  |
| --- | --- |
|  | **−−force−po** |

|  |  |
| --- | --- |
|  | write PO file even if empty |

|  |  |
| --- | --- |
|  | **−−indent** |

|  |  |
| --- | --- |
|  | indented output style |

|  |  |
| --- | --- |
|  | **−−no−location** |

|  |  |
| --- | --- |
|  | suppress ’#: filename:line’ lines |

|  |  |
| --- | --- |
|  | **−−add−location** |

|  |  |
| --- | --- |
|  | preserve ’#: filename:line’ lines (default) |

|  |  |
| --- | --- |
|  | **−−strict** |

|  |  |
| --- | --- |
|  | strict Uniforum output style |

|  |  |
| --- | --- |
|  | **−p**, **−−properties−output** |

|  |  |
| --- | --- |
|  | write out a Java .properties file |

|  |  |
| --- | --- |
|  | **−−stringtable−output** |

|  |  |
| --- | --- |
|  | write out a NeXTstep/GNUstep .strings file |

|  |  |
| --- | --- |
|  | **−w**, **−−width**=*NUMBER* |

|  |  |
| --- | --- |
|  | set output page width |

|  |  |
| --- | --- |
|  | **−−no−wrap** |

|  |  |
| --- | --- |
|  | do not break long message lines, longer than the output page width, into several lines |

|  |  |
| --- | --- |
|  | **−−sort−output** |

|  |  |
| --- | --- |
|  | generate sorted output |

|  |  |
| --- | --- |
|  | **−−sort−by−file** |

|  |  |
| --- | --- |
|  | sort output by file location |

|  |  |
| --- | --- |
|  | **Informative output:** |

|  |  |
| --- | --- |
|  | **−h**, **−−help** |

|  |  |
| --- | --- |
|  | display this help and exit |

|  |  |
| --- | --- |
|  | **−V**, **−−version** |

|  |  |
| --- | --- |
|  | output version information and exit |

## AUTHOR

|  |  |
| --- | --- |
|  | Written by Bruno Haible. |

## REPORTING BUGS

|  |  |
| --- | --- |
|  | Report bugs to <bug-gnu-gettext@gnu.org>. |

## COPYRIGHT

|  |  |
| --- | --- |
|  | Copyright © 2001-2010 Free Software Foundation, Inc. License GPLv3+: GNU GPL version 3 or later <http://gnu.org/licenses/gpl.html>  This is free software: you are free to change and redistribute it. There is NO WARRANTY, to the extent permitted by law. |

## SEE ALSO

|  |  |
| --- | --- |
|  | The full documentation for **msggrep** is maintained as a Texinfo manual. If the **info** and **msggrep** programs are properly installed at your site, the command |

|  |  |
| --- | --- |
|  | **info msggrep** |

|  |  |
| --- | --- |
|  | should give you access to the complete manual. |

---
