# Supplementary material for: rDock: A Fast, Versatile and Open Source Program for Docking Ligands to Proteins and Nucleic Acids
Source: PLoS Comput Biol. 2014 Apr 10;10(4):e1003571. doi: 10.1371/journal.pcbi.1003571 (PMC3983074; doi:10.1371/journal.pcbi.1003571)
Supplement: Software S1 — Compressed file with the source code of the rDock software for ligand docking to Proteins and Nucleic Acids. (GZ) [file pcbi.1003571.s010.gz › rDock_2013.1_src/gettext-0.18.tar.gz!/gettext-0.18/gettext-tools/man/msguniq.1.html]

MSGUNIQ


# MSGUNIQ

NAME  
SYNOPSIS  
DESCRIPTION  
AUTHOR  
REPORTING BUGS  
COPYRIGHT  
SEE ALSO  


---


## NAME

|  |  |
| --- | --- |
|  | msguniq − unify duplicate translations in message catalog |

## SYNOPSIS

|  |  |
| --- | --- |
|  | **msguniq** [*OPTION*] [*INPUTFILE*] |

## DESCRIPTION

|  |  |
| --- | --- |
|  | Unifies duplicate translations in a translation catalog. Finds duplicate translations of the same message ID. Such duplicates are invalid input for other programs like msgfmt, msgmerge or msgcat. By default, duplicates are merged together. When using the **−−repeated** option, only duplicates are output, and all other messages are discarded. Comments and extracted comments will be cumulated, except that if **−−use−first** is specified, they will be taken from the first translation. File positions will be cumulated. When using the **−−unique** option, duplicates are discarded.  Mandatory arguments to long options are mandatory for short options too. |

|  |  |
| --- | --- |
|  | **Input file location:** |

|  |  |
| --- | --- |
|  | INPUTFILE |

|  |  |
| --- | --- |
|  | input PO file |

|  |  |
| --- | --- |
|  | **−D**, **−−directory**=*DIRECTORY* |

|  |  |
| --- | --- |
|  | add DIRECTORY to list for input files search |

|  |  |
| --- | --- |
|  | If no input file is given or if it is -, standard input is read. |

|  |  |
| --- | --- |
|  | **Output file location:** |

|  |  |
| --- | --- |
|  | **−o**, **−−output−file**=*FILE* |

|  |  |
| --- | --- |
|  | write output to specified file |

|  |  |
| --- | --- |
|  | The results are written to standard output if no output file is specified or if it is -. |

|  |  |
| --- | --- |
|  | **Message selection:** |

|  |  |
| --- | --- |
|  | **−d**, **−−repeated** |

|  |  |
| --- | --- |
|  | print only duplicates |

|  |  |
| --- | --- |
|  | **−u**, **−−unique** |

|  |  |
| --- | --- |
|  | print only unique messages, discard duplicates |

|  |  |
| --- | --- |
|  | **Input file syntax:** |

|  |  |
| --- | --- |
|  | **−P**, **−−properties−input** |

|  |  |
| --- | --- |
|  | input file is in Java .properties syntax |

|  |  |
| --- | --- |
|  | **−−stringtable−input** |

|  |  |
| --- | --- |
|  | input file is in NeXTstep/GNUstep .strings syntax |

|  |  |
| --- | --- |
|  | **Output details:** |

|  |  |
| --- | --- |
|  | **−t**, **−−to−code**=*NAME* |

|  |  |
| --- | --- |
|  | encoding for output |

|  |  |
| --- | --- |
|  | **−−use−first** |

|  |  |
| --- | --- |
|  | use first available translation for each message, don’t merge several translations |

|  |  |
| --- | --- |
|  | **−−color** |

|  |  |
| --- | --- |
|  | use colors and other text attributes always |

|  |  |
| --- | --- |
|  | **−−color**=*WHEN* |

|  |  |
| --- | --- |
|  | use colors and other text attributes if WHEN. WHEN may be ’always’, ’never’, ’auto’, or ’html’. |

|  |  |
| --- | --- |
|  | **−−style**=*STYLEFILE* |

|  |  |
| --- | --- |
|  | specify CSS style rule file for **−−color** |

|  |  |
| --- | --- |
|  | **−e**, **−−no−escape** |

|  |  |
| --- | --- |
|  | do not use C escapes in output (default) |

|  |  |
| --- | --- |
|  | **−E**, **−−escape** |

|  |  |
| --- | --- |
|  | use C escapes in output, no extended chars |

|  |  |
| --- | --- |
|  | **−−force−po** |

|  |  |
| --- | --- |
|  | write PO file even if empty |

|  |  |
| --- | --- |
|  | **−i**, **−−indent** |

|  |  |
| --- | --- |
|  | write the .po file using indented style |

|  |  |
| --- | --- |
|  | **−−no−location** |

|  |  |
| --- | --- |
|  | do not write ’#: filename:line’ lines |

|  |  |
| --- | --- |
|  | **−n**, **−−add−location** |

|  |  |
| --- | --- |
|  | generate ’#: filename:line’ lines (default) |

|  |  |
| --- | --- |
|  | **−−strict** |

|  |  |
| --- | --- |
|  | write out strict Uniforum conforming .po file |

|  |  |
| --- | --- |
|  | **−p**, **−−properties−output** |

|  |  |
| --- | --- |
|  | write out a Java .properties file |

|  |  |
| --- | --- |
|  | **−−stringtable−output** |

|  |  |
| --- | --- |
|  | write out a NeXTstep/GNUstep .strings file |

|  |  |
| --- | --- |
|  | **−w**, **−−width**=*NUMBER* |

|  |  |
| --- | --- |
|  | set output page width |

|  |  |
| --- | --- |
|  | **−−no−wrap** |

|  |  |
| --- | --- |
|  | do not break long message lines, longer than the output page width, into several lines |

|  |  |
| --- | --- |
|  | **−s**, **−−sort−output** |

|  |  |
| --- | --- |
|  | generate sorted output |

|  |  |
| --- | --- |
|  | **−F**, **−−sort−by−file** |

|  |  |
| --- | --- |
|  | sort output by file location |

|  |  |
| --- | --- |
|  | **Informative output:** |

|  |  |
| --- | --- |
|  | **−h**, **−−help** |

|  |  |
| --- | --- |
|  | display this help and exit |

|  |  |
| --- | --- |
|  | **−V**, **−−version** |

|  |  |
| --- | --- |
|  | output version information and exit |

## AUTHOR

|  |  |
| --- | --- |
|  | Written by Bruno Haible. |

## REPORTING BUGS

|  |  |
| --- | --- |
|  | Report bugs to <bug-gnu-gettext@gnu.org>. |

## COPYRIGHT

|  |  |
| --- | --- |
|  | Copyright © 2001-2010 Free Software Foundation, Inc. License GPLv3+: GNU GPL version 3 or later <http://gnu.org/licenses/gpl.html>  This is free software: you are free to change and redistribute it. There is NO WARRANTY, to the extent permitted by law. |

## SEE ALSO

|  |  |
| --- | --- |
|  | The full documentation for **msguniq** is maintained as a Texinfo manual. If the **info** and **msguniq** programs are properly installed at your site, the command |

|  |  |
| --- | --- |
|  | **info msguniq** |

|  |  |
| --- | --- |
|  | should give you access to the complete manual. |

---
