# Supplementary material for: rDock: A Fast, Versatile and Open Source Program for Docking Ligands to Proteins and Nucleic Acids
Source: PLoS Comput Biol. 2014 Apr 10;10(4):e1003571. doi: 10.1371/journal.pcbi.1003571 (PMC3983074; doi:10.1371/journal.pcbi.1003571)
Supplement: Software S1 — Compressed file with the source code of the rDock software for ligand docking to Proteins and Nucleic Acids. (GZ) [file pcbi.1003571.s010.gz › rDock_2013.1_src/gettext-0.18.tar.gz!/gettext-0.18/gettext-tools/man/msgattrib.1.html]

MSGATTRIB


# MSGATTRIB

NAME  
SYNOPSIS  
DESCRIPTION  
AUTHOR  
REPORTING BUGS  
COPYRIGHT  
SEE ALSO  


---


## NAME

|  |  |
| --- | --- |
|  | msgattrib − attribute matching and manipulation on message catalog |

## SYNOPSIS

|  |  |
| --- | --- |
|  | **msgattrib** [*OPTION*] [*INPUTFILE*] |

## DESCRIPTION

|  |  |
| --- | --- |
|  | Filters the messages of a translation catalog according to their attributes, and manipulates the attributes.  Mandatory arguments to long options are mandatory for short options too. |

|  |  |
| --- | --- |
|  | **Input file location:** |

|  |  |
| --- | --- |
|  | INPUTFILE |

|  |  |
| --- | --- |
|  | input PO file |

|  |  |
| --- | --- |
|  | **−D**, **−−directory**=*DIRECTORY* |

|  |  |
| --- | --- |
|  | add DIRECTORY to list for input files search |

|  |  |
| --- | --- |
|  | If no input file is given or if it is -, standard input is read. |

|  |  |
| --- | --- |
|  | **Output file location:** |

|  |  |
| --- | --- |
|  | **−o**, **−−output−file**=*FILE* |

|  |  |
| --- | --- |
|  | write output to specified file |

|  |  |
| --- | --- |
|  | The results are written to standard output if no output file is specified or if it is -. |

|  |  |
| --- | --- |
|  | **Message selection:** |

|  |  |
| --- | --- |
|  | **−−translated** |

|  |  |
| --- | --- |
|  | keep translated, remove untranslated messages |

|  |  |
| --- | --- |
|  | **−−untranslated** |

|  |  |
| --- | --- |
|  | keep untranslated, remove translated messages |

|  |  |
| --- | --- |
|  | **−−no−fuzzy** |

|  |  |
| --- | --- |
|  | remove ’fuzzy’ marked messages |

|  |  |
| --- | --- |
|  | **−−only−fuzzy** |

|  |  |
| --- | --- |
|  | keep ’fuzzy’ marked messages |

|  |  |
| --- | --- |
|  | **−−no−obsolete** |

|  |  |
| --- | --- |
|  | remove obsolete #~ messages |

|  |  |
| --- | --- |
|  | **−−only−obsolete** |

|  |  |
| --- | --- |
|  | keep obsolete #~ messages |

|  |  |
| --- | --- |
|  | **Attribute manipulation:** |

|  |  |
| --- | --- |
|  | **−−set−fuzzy** |

|  |  |
| --- | --- |
|  | set all messages ’fuzzy’ |

|  |  |
| --- | --- |
|  | **−−clear−fuzzy** |

|  |  |
| --- | --- |
|  | set all messages non-’fuzzy’ |

|  |  |
| --- | --- |
|  | **−−set−obsolete** |

|  |  |
| --- | --- |
|  | set all messages obsolete |

|  |  |
| --- | --- |
|  | **−−clear−obsolete** |

|  |  |
| --- | --- |
|  | set all messages non-obsolete |

|  |  |
| --- | --- |
|  | **−−clear−previous** |

|  |  |
| --- | --- |
|  | remove the "previous msgid" from all messages |

|  |  |
| --- | --- |
|  | **−−only−file**=*FILE*.po |

|  |  |
| --- | --- |
|  | manipulate only entries listed in FILE.po |

|  |  |
| --- | --- |
|  | **−−ignore−file**=*FILE*.po |

|  |  |
| --- | --- |
|  | manipulate only entries not listed in FILE.po |

|  |  |
| --- | --- |
|  | **−−fuzzy** |

|  |  |
| --- | --- |
|  | synonym for **−−only−fuzzy −−clear−fuzzy** |

|  |  |
| --- | --- |
|  | **−−obsolete** |

|  |  |
| --- | --- |
|  | synonym for **−−only−obsolete −−clear−obsolete** |

|  |  |
| --- | --- |
|  | **Input file syntax:** |

|  |  |
| --- | --- |
|  | **−P**, **−−properties−input** |

|  |  |
| --- | --- |
|  | input file is in Java .properties syntax |

|  |  |
| --- | --- |
|  | **−−stringtable−input** |

|  |  |
| --- | --- |
|  | input file is in NeXTstep/GNUstep .strings syntax |

|  |  |
| --- | --- |
|  | **Output details:** |

|  |  |
| --- | --- |
|  | **−−color** |

|  |  |
| --- | --- |
|  | use colors and other text attributes always |

|  |  |
| --- | --- |
|  | **−−color**=*WHEN* |

|  |  |
| --- | --- |
|  | use colors and other text attributes if WHEN. WHEN may be ’always’, ’never’, ’auto’, or ’html’. |

|  |  |
| --- | --- |
|  | **−−style**=*STYLEFILE* |

|  |  |
| --- | --- |
|  | specify CSS style rule file for **−−color** |

|  |  |
| --- | --- |
|  | **−e**, **−−no−escape** |

|  |  |
| --- | --- |
|  | do not use C escapes in output (default) |

|  |  |
| --- | --- |
|  | **−E**, **−−escape** |

|  |  |
| --- | --- |
|  | use C escapes in output, no extended chars |

|  |  |
| --- | --- |
|  | **−−force−po** |

|  |  |
| --- | --- |
|  | write PO file even if empty |

|  |  |
| --- | --- |
|  | **−i**, **−−indent** |

|  |  |
| --- | --- |
|  | write the .po file using indented style |

|  |  |
| --- | --- |
|  | **−−no−location** |

|  |  |
| --- | --- |
|  | do not write ’#: filename:line’ lines |

|  |  |
| --- | --- |
|  | **−n**, **−−add−location** |

|  |  |
| --- | --- |
|  | generate ’#: filename:line’ lines (default) |

|  |  |
| --- | --- |
|  | **−−strict** |

|  |  |
| --- | --- |
|  | write out strict Uniforum conforming .po file |

|  |  |
| --- | --- |
|  | **−p**, **−−properties−output** |

|  |  |
| --- | --- |
|  | write out a Java .properties file |

|  |  |
| --- | --- |
|  | **−−stringtable−output** |

|  |  |
| --- | --- |
|  | write out a NeXTstep/GNUstep .strings file |

|  |  |
| --- | --- |
|  | **−w**, **−−width**=*NUMBER* |

|  |  |
| --- | --- |
|  | set output page width |

|  |  |
| --- | --- |
|  | **−−no−wrap** |

|  |  |
| --- | --- |
|  | do not break long message lines, longer than the output page width, into several lines |

|  |  |
| --- | --- |
|  | **−s**, **−−sort−output** |

|  |  |
| --- | --- |
|  | generate sorted output |

|  |  |
| --- | --- |
|  | **−F**, **−−sort−by−file** |

|  |  |
| --- | --- |
|  | sort output by file location |

|  |  |
| --- | --- |
|  | **Informative output:** |

|  |  |
| --- | --- |
|  | **−h**, **−−help** |

|  |  |
| --- | --- |
|  | display this help and exit |

|  |  |
| --- | --- |
|  | **−V**, **−−version** |

|  |  |
| --- | --- |
|  | output version information and exit |

## AUTHOR

|  |  |
| --- | --- |
|  | Written by Bruno Haible. |

## REPORTING BUGS

|  |  |
| --- | --- |
|  | Report bugs to <bug-gnu-gettext@gnu.org>. |

## COPYRIGHT

|  |  |
| --- | --- |
|  | Copyright © 2001-2010 Free Software Foundation, Inc. License GPLv3+: GNU GPL version 3 or later <http://gnu.org/licenses/gpl.html>  This is free software: you are free to change and redistribute it. There is NO WARRANTY, to the extent permitted by law. |

## SEE ALSO

|  |  |
| --- | --- |
|  | The full documentation for **msgattrib** is maintained as a Texinfo manual. If the **info** and **msgattrib** programs are properly installed at your site, the command |

|  |  |
| --- | --- |
|  | **info msgattrib** |

|  |  |
| --- | --- |
|  | should give you access to the complete manual. |

---
