# Supplementary material for: rDock: A Fast, Versatile and Open Source Program for Docking Ligands to Proteins and Nucleic Acids
Source: PLoS Comput Biol. 2014 Apr 10;10(4):e1003571. doi: 10.1371/journal.pcbi.1003571 (PMC3983074; doi:10.1371/journal.pcbi.1003571)
Supplement: Software S1 — Compressed file with the source code of the rDock software for ligand docking to Proteins and Nucleic Acids. (GZ) [file pcbi.1003571.s010.gz › rDock_2013.1_src/gettext-0.18.tar.gz!/gettext-0.18/gettext-tools/man/msgcat.1.html]

MSGCAT


# MSGCAT

NAME  
SYNOPSIS  
DESCRIPTION  
AUTHOR  
REPORTING BUGS  
COPYRIGHT  
SEE ALSO  


---


## NAME

|  |  |
| --- | --- |
|  | msgcat − combines several message catalogs |

## SYNOPSIS

|  |  |
| --- | --- |
|  | **msgcat** [*OPTION*] [*INPUTFILE*]... |

## DESCRIPTION

|  |  |
| --- | --- |
|  | Concatenates and merges the specified PO files. Find messages which are common to two or more of the specified PO files. By using the **−−more−than** option, greater commonality may be requested before messages are printed. Conversely, the **−−less−than** option may be used to specify less commonality before messages are printed (i.e. **−−less−than**=*2* will only print the unique messages). Translations, comments and extract comments will be cumulated, except that if **−−use−first** is specified, they will be taken from the first PO file to define them. File positions from all PO files will be cumulated.  Mandatory arguments to long options are mandatory for short options too. |

|  |  |
| --- | --- |
|  | **Input file location:** |

|  |  |
| --- | --- |
|  | INPUTFILE ... |

|  |  |
| --- | --- |
|  | input files |

|  |  |
| --- | --- |
|  | **−f**, **−−files−from**=*FILE* |

|  |  |
| --- | --- |
|  | get list of input files from FILE |

|  |  |
| --- | --- |
|  | **−D**, **−−directory**=*DIRECTORY* |

|  |  |
| --- | --- |
|  | add DIRECTORY to list for input files search |

|  |  |
| --- | --- |
|  | If input file is -, standard input is read. |

|  |  |
| --- | --- |
|  | **Output file location:** |

|  |  |
| --- | --- |
|  | **−o**, **−−output−file**=*FILE* |

|  |  |
| --- | --- |
|  | write output to specified file |

|  |  |
| --- | --- |
|  | The results are written to standard output if no output file is specified or if it is -. |

|  |  |
| --- | --- |
|  | **Message selection:** |

|  |  |
| --- | --- |
|  | -<, **−−less−than**=*NUMBER* |

|  |  |
| --- | --- |
|  | print messages with less than this many definitions, defaults to infinite if not set |

|  |  |
| --- | --- |
|  | ->, **−−more−than**=*NUMBER* |

|  |  |
| --- | --- |
|  | print messages with more than this many definitions, defaults to 0 if not set |

|  |  |
| --- | --- |
|  | **−u**, **−−unique** |

|  |  |
| --- | --- |
|  | shorthand for **−−less−than**=*2*, requests that only unique messages be printed |

|  |  |
| --- | --- |
|  | **Input file syntax:** |

|  |  |
| --- | --- |
|  | **−P**, **−−properties−input** |

|  |  |
| --- | --- |
|  | input files are in Java .properties syntax |

|  |  |
| --- | --- |
|  | **−−stringtable−input** |

|  |  |
| --- | --- |
|  | input files are in NeXTstep/GNUstep .strings syntax |

|  |  |
| --- | --- |
|  | **Output details:** |

|  |  |
| --- | --- |
|  | **−t**, **−−to−code**=*NAME* |

|  |  |
| --- | --- |
|  | encoding for output |

|  |  |
| --- | --- |
|  | **−−use−first** |

|  |  |
| --- | --- |
|  | use first available translation for each message, don’t merge several translations |

|  |  |
| --- | --- |
|  | **−−lang**=*CATALOGNAME* |

|  |  |
| --- | --- |
|  | set ’Language’ field in the header entry |

|  |  |
| --- | --- |
|  | **−−color** |

|  |  |
| --- | --- |
|  | use colors and other text attributes always |

|  |  |
| --- | --- |
|  | **−−color**=*WHEN* |

|  |  |
| --- | --- |
|  | use colors and other text attributes if WHEN. WHEN may be ’always’, ’never’, ’auto’, or ’html’. |

|  |  |
| --- | --- |
|  | **−−style**=*STYLEFILE* |

|  |  |
| --- | --- |
|  | specify CSS style rule file for **−−color** |

|  |  |
| --- | --- |
|  | **−e**, **−−no−escape** |

|  |  |
| --- | --- |
|  | do not use C escapes in output (default) |

|  |  |
| --- | --- |
|  | **−E**, **−−escape** |

|  |  |
| --- | --- |
|  | use C escapes in output, no extended chars |

|  |  |
| --- | --- |
|  | **−−force−po** |

|  |  |
| --- | --- |
|  | write PO file even if empty |

|  |  |
| --- | --- |
|  | **−i**, **−−indent** |

|  |  |
| --- | --- |
|  | write the .po file using indented style |

|  |  |
| --- | --- |
|  | **−−no−location** |

|  |  |
| --- | --- |
|  | do not write ’#: filename:line’ lines |

|  |  |
| --- | --- |
|  | **−n**, **−−add−location** |

|  |  |
| --- | --- |
|  | generate ’#: filename:line’ lines (default) |

|  |  |
| --- | --- |
|  | **−−strict** |

|  |  |
| --- | --- |
|  | write out strict Uniforum conforming .po file |

|  |  |
| --- | --- |
|  | **−p**, **−−properties−output** |

|  |  |
| --- | --- |
|  | write out a Java .properties file |

|  |  |
| --- | --- |
|  | **−−stringtable−output** |

|  |  |
| --- | --- |
|  | write out a NeXTstep/GNUstep .strings file |

|  |  |
| --- | --- |
|  | **−w**, **−−width**=*NUMBER* |

|  |  |
| --- | --- |
|  | set output page width |

|  |  |
| --- | --- |
|  | **−−no−wrap** |

|  |  |
| --- | --- |
|  | do not break long message lines, longer than the output page width, into several lines |

|  |  |
| --- | --- |
|  | **−s**, **−−sort−output** |

|  |  |
| --- | --- |
|  | generate sorted output |

|  |  |
| --- | --- |
|  | **−F**, **−−sort−by−file** |

|  |  |
| --- | --- |
|  | sort output by file location |

|  |  |
| --- | --- |
|  | **Informative output:** |

|  |  |
| --- | --- |
|  | **−h**, **−−help** |

|  |  |
| --- | --- |
|  | display this help and exit |

|  |  |
| --- | --- |
|  | **−V**, **−−version** |

|  |  |
| --- | --- |
|  | output version information and exit |

## AUTHOR

|  |  |
| --- | --- |
|  | Written by Bruno Haible. |

## REPORTING BUGS

|  |  |
| --- | --- |
|  | Report bugs to <bug-gnu-gettext@gnu.org>. |

## COPYRIGHT

|  |  |
| --- | --- |
|  | Copyright © 2001-2010 Free Software Foundation, Inc. License GPLv3+: GNU GPL version 3 or later <http://gnu.org/licenses/gpl.html>  This is free software: you are free to change and redistribute it. There is NO WARRANTY, to the extent permitted by law. |

## SEE ALSO

|  |  |
| --- | --- |
|  | The full documentation for **msgcat** is maintained as a Texinfo manual. If the **info** and **msgcat** programs are properly installed at your site, the command |

|  |  |
| --- | --- |
|  | **info msgcat** |

|  |  |
| --- | --- |
|  | should give you access to the complete manual. |

---
