# Supplementary material for: rDock: A Fast, Versatile and Open Source Program for Docking Ligands to Proteins and Nucleic Acids
Source: PLoS Comput Biol. 2014 Apr 10;10(4):e1003571. doi: 10.1371/journal.pcbi.1003571 (PMC3983074; doi:10.1371/journal.pcbi.1003571)
Supplement: Software S1 — Compressed file with the source code of the rDock software for ligand docking to Proteins and Nucleic Acids. (GZ) [file pcbi.1003571.s010.gz › rDock_2013.1_src/gettext-0.18.tar.gz!/gettext-0.18/gettext-tools/man/msgcmp.1.html]

MSGCMP


# MSGCMP

NAME  
SYNOPSIS  
DESCRIPTION  
AUTHOR  
REPORTING BUGS  
COPYRIGHT  
SEE ALSO  


---


## NAME

|  |  |
| --- | --- |
|  | msgcmp − compare message catalog and template |

## SYNOPSIS

|  |  |
| --- | --- |
|  | **msgcmp** [*OPTION*] *def.po ref.pot* |

## DESCRIPTION

|  |  |
| --- | --- |
|  | Compare two Uniforum style .po files to check that both contain the same set of msgid strings. The def.po file is an existing PO file with the translations. The ref.pot file is the last created PO file, or a PO Template file (generally created by xgettext). This is useful for checking that you have translated each and every message in your program. Where an exact match cannot be found, fuzzy matching is used to produce better diagnostics.  Mandatory arguments to long options are mandatory for short options too. |

|  |  |
| --- | --- |
|  | **Input file location:** |

|  |  |  |  |  |
| --- | --- | --- | --- | --- |
|  | def.po |  | translations |  |

|  |  |
| --- | --- |
|  | ref.pot |

|  |  |
| --- | --- |
|  | references to the sources |

|  |  |
| --- | --- |
|  | **−D**, **−−directory**=*DIRECTORY* |

|  |  |
| --- | --- |
|  | add DIRECTORY to list for input files search |

|  |  |
| --- | --- |
|  | **Operation modifiers:** |

|  |  |
| --- | --- |
|  | **−m**, **−−multi−domain** |

|  |  |
| --- | --- |
|  | apply ref.pot to each of the domains in def.po |

|  |  |
| --- | --- |
|  | **−N**, **−−no−fuzzy−matching** |

|  |  |
| --- | --- |
|  | do not use fuzzy matching |

|  |  |
| --- | --- |
|  | **−−use−fuzzy** |

|  |  |
| --- | --- |
|  | consider fuzzy entries |

|  |  |
| --- | --- |
|  | **−−use−untranslated** |

|  |  |
| --- | --- |
|  | consider untranslated entries |

|  |  |
| --- | --- |
|  | **Input file syntax:** |

|  |  |
| --- | --- |
|  | **−P**, **−−properties−input** |

|  |  |
| --- | --- |
|  | input files are in Java .properties syntax |

|  |  |
| --- | --- |
|  | **−−stringtable−input** |

|  |  |
| --- | --- |
|  | input files are in NeXTstep/GNUstep .strings syntax |

|  |  |
| --- | --- |
|  | **Informative output:** |

|  |  |
| --- | --- |
|  | **−h**, **−−help** |

|  |  |
| --- | --- |
|  | display this help and exit |

|  |  |
| --- | --- |
|  | **−V**, **−−version** |

|  |  |
| --- | --- |
|  | output version information and exit |

## AUTHOR

|  |  |
| --- | --- |
|  | Written by Peter Miller. |

## REPORTING BUGS

|  |  |
| --- | --- |
|  | Report bugs to <bug-gnu-gettext@gnu.org>. |

## COPYRIGHT

|  |  |
| --- | --- |
|  | Copyright © 1995-1998, 2000-2010 Free Software Foundation, Inc. License GPLv3+: GNU GPL version 3 or later <http://gnu.org/licenses/gpl.html>  This is free software: you are free to change and redistribute it. There is NO WARRANTY, to the extent permitted by law. |

## SEE ALSO

|  |  |
| --- | --- |
|  | The full documentation for **msgcmp** is maintained as a Texinfo manual. If the **info** and **msgcmp** programs are properly installed at your site, the command |

|  |  |
| --- | --- |
|  | **info msgcmp** |

|  |  |
| --- | --- |
|  | should give you access to the complete manual. |

---
