# Supplementary material for: rDock: A Fast, Versatile and Open Source Program for Docking Ligands to Proteins and Nucleic Acids
Source: PLoS Comput Biol. 2014 Apr 10;10(4):e1003571. doi: 10.1371/journal.pcbi.1003571 (PMC3983074; doi:10.1371/journal.pcbi.1003571)
Supplement: Software S1 — Compressed file with the source code of the rDock software for ligand docking to Proteins and Nucleic Acids. (GZ) [file pcbi.1003571.s010.gz › rDock_2013.1_src/gettext-0.18.tar.gz!/gettext-0.18/gettext-tools/doc/gettext_5.html]

GNU gettext utilities - 5 Making the PO Template File

Go to the first, previous, next, last section, table of contents.

---

# 5 Making the PO Template File

After preparing the sources, the programmer creates a PO template file.
This section explains how to use `xgettext` for this purpose.

`xgettext` creates a file named ‘domainname.po’. You
should then rename it to ‘domainname.pot’. (Why doesn't
`xgettext` create it under the name ‘domainname.pot’
right away? The answer is: for historical reasons. When `xgettext`
was specified, the distinction between a PO file and PO file template
was fuzzy, and the suffix `‘.pot’` wasn't in use at that time.)

## 5.1 Invoking the `xgettext` Program

```
xgettext [option] [inputfile] ...
```

The `xgettext` program extracts translatable strings from given
input files.

### 5.1.1 Input file location

`‘inputfile ...’`: Input files. `‘-f file’`: `‘--files-from=file’`: Read the names of the input files from file instead of getting them from the command line. `‘-D directory’`: `‘--directory=directory’`: Add directory to the list of directories. Source files are searched relative to this list of directories. The resulting ‘.po’ file will be written relative to the current directory, though.

If inputfile is `‘-’`, standard input is read.

### 5.1.2 Output file location

`‘-d name’`: `‘--default-domain=name’`: Use ‘name.po’ for output (instead of ‘messages.po’). `‘-o file’`: `‘--output=file’`: Write output to specified file (instead of ‘name.po’ or ‘messages.po’). `‘-p dir’`: `‘--output-dir=dir’`: Output files will be placed in directory dir.

If the output file is `‘-’` or `‘/dev/stdout’`, the output
is written to standard output.

### 5.1.3 Choice of input file language

`‘-L name’`: `‘--language=name’`: Specifies the language of the input files. The supported languages are `C`, `C++`, `ObjectiveC`, `PO`, `Python`, `Lisp`, `EmacsLisp`, `librep`, `Scheme`, `Smalltalk`, `Java`, `JavaProperties`, `C#`, `awk`, `YCP`, `Tcl`, `Perl`, `PHP`, `GCC-source`, `NXStringTable`, `RST`, `Glade`. `‘-C’`: `‘--c++’`: This is a shorthand for `--language=C++`.

By default the language is guessed depending on the input file name
extension.

### 5.1.4 Input file interpretation

`‘--from-code=name’`: Specifies the encoding of the input files. This option is needed only if some untranslated message strings or their corresponding comments contain non-ASCII characters. Note that Tcl and Glade input files are always assumed to be in UTF-8, regardless of this option.

By default the input files are assumed to be in ASCII.

### 5.1.5 Operation mode

`‘-j’`: `‘--join-existing’`: Join messages with existing file. `‘-x file’`: `‘--exclude-file=file’`: Entries from file are not extracted. file should be a PO or POT file. `‘-c[tag]’`: `‘--add-comments[=tag]’`: Place comment blocks starting with tag and preceding keyword lines in the output file. Without a tag, the option means to put *all* comment blocks preceding keyword lines in the output file.

### 5.1.6 Language specific options

`‘-a’`: `‘--extract-all’`: Extract all strings. This option has an effect with most languages, namely C, C++, ObjectiveC, Shell, Python, Lisp, EmacsLisp, librep, Java, C#, awk, Tcl, Perl, PHP, GCC-source, Glade. `‘-k[keywordspec]’`: `‘--keyword[=keywordspec]’`: Specify keywordspec as an additional keyword to be looked for. Without a keywordspec, the option means to not use default keywords. If keywordspec is a C identifier id, `xgettext` looks for strings in the first argument of each call to the function or macro id. If keywordspec is of the form `‘id:argnum’`, `xgettext` looks for strings in the argnumth argument of the call. If keywordspec is of the form `‘id:argnum1,argnum2’`, `xgettext` looks for strings in the argnum1st argument and in the argnum2nd argument of the call, and treats them as singular/plural variants for a message with plural handling. Also, if keywordspec is of the form `‘id:contextargnumc,argnum’` or `‘id:argnum,contextargnumc’`, `xgettext` treats strings in the contextargnumth argument as a context specifier. And, as a special-purpose support for GNOME, if keywordspec is of the form `‘id:argnumg’`, `xgettext` recognizes the argnumth argument as a string with context, using the GNOME `glib` syntax `‘"msgctxt|msgid"’`. Furthermore, if keywordspec is of the form `‘id:...,totalnumargst’`, `xgettext` recognizes this argument specification only if the number of actual arguments is equal to totalnumargs. This is useful for disambiguating overloaded function calls in C++. Finally, if keywordspec is of the form `‘id:argnum...,"xcomment"’`, `xgettext`, when extracting a message from the specified argument strings, adds an extracted comment xcomment to the message. Note that when used through a normal shell command line, the double-quotes around the xcomment need to be escaped. This option has an effect with most languages, namely C, C++, ObjectiveC, Shell, Python, Lisp, EmacsLisp, librep, Java, C#, awk, Tcl, Perl, PHP, GCC-source, Glade. The default keyword specifications, which are always looked for if not explicitly disabled, are language dependent. They are: - For C, C++, and GCC-source: `gettext`, `dgettext:2`, `dcgettext:2`, `ngettext:1,2`, `dngettext:2,3`, `dcngettext:2,3`, `gettext_noop`, and `pgettext:1c,2`, `dpgettext:2c,3`, `dcpgettext:2c,3`, `npgettext:1c,2,3`, `dnpgettext:2c,3,4`, `dcnpgettext:2c,3,4`.- For Objective C: Like for C, and also `NSLocalizedString`, `_`, `NSLocalizedStaticString`, `__`.- For Shell scripts: `gettext`, `ngettext:1,2`, `eval_gettext`, `eval_ngettext:1,2`.- For Python: `gettext`, `ugettext`, `dgettext:2`, `ngettext:1,2`, `ungettext:1,2`, `dngettext:2,3`, `_`.- For Lisp: `gettext`, `ngettext:1,2`, `gettext-noop`.- For EmacsLisp: `_`.- For librep: `_`.- For Scheme: `gettext`, `ngettext:1,2`, `gettext-noop`.- For Java: `GettextResource.gettext:2`, `GettextResource.ngettext:2,3`, `GettextResource.pgettext:2c,3`, `GettextResource.npgettext:2c,3,4`, `gettext`, `ngettext:1,2`, `pgettext:1c,2`, `npgettext:1c,2,3`, `getString`.- For C#: `GetString`, `GetPluralString:1,2`, `GetParticularString:1c,2`, `GetParticularPluralString:1c,2,3`.- For awk: `dcgettext`, `dcngettext:1,2`.- For Tcl: `::msgcat::mc`.- For Perl: `gettext`, `%gettext`, `$gettext`, `dgettext:2`, `dcgettext:2`, `ngettext:1,2`, `dngettext:2,3`, `dcngettext:2,3`, `gettext_noop`.- For PHP: `_`, `gettext`, `dgettext:2`, `dcgettext:2`, `ngettext:1,2`, `dngettext:2,3`, `dcngettext:2,3`.- For Glade 1: `label`, `title`, `text`, `format`, `copyright`, `comments`, `preview_text`, `tooltip`. To disable the default keyword specifications, the option `‘-k’` or `‘--keyword’` or `‘--keyword=’`, without a keywordspec, can be used. `‘--flag=word:arg:flag’`: Specifies additional flags for strings occurring as part of the argth argument of the function word. The possible flags are the possible format string indicators, such as `‘c-format’`, and their negations, such as `‘no-c-format’`, possibly prefixed with `‘pass-’`. The meaning of `--flag=function:arg:lang-format` is that in language lang, the specified function expects as argth argument a format string. (For those of you familiar with GCC function attributes, `--flag=function:arg:c-format` is roughly equivalent to the declaration `‘__attribute__ ((__format__ (__printf__, arg, ...)))’` attached to function in a C source file.) For example, if you use the `‘error’` function from GNU libc, you can specify its behaviour through `--flag=error:3:c-format`. The effect of this specification is that `xgettext` will mark as format strings all `gettext` invocations that occur as argth argument of function. This is useful when such strings contain no format string directives: together with the checks done by `‘msgfmt -c’` it will ensure that translators cannot accidentally use format string directives that would lead to a crash at runtime. The meaning of `--flag=function:arg:pass-lang-format` is that in language lang, if the function call occurs in a position that must yield a format string, then its argth argument must yield a format string of the same type as well. (If you know GCC function attributes, the `--flag=function:arg:pass-c-format` option is roughly equivalent to the declaration `‘__attribute__ ((__format_arg__ (arg)))’` attached to function in a C source file.) For example, if you use the `‘_’` shortcut for the `gettext` function, you should use `--flag=_:1:pass-c-format`. The effect of this specification is that `xgettext` will propagate a format string requirement for a `_("string")` call to its first argument, the literal `"string"`, and thus mark it as a format string. This is useful when such strings contain no format string directives: together with the checks done by `‘msgfmt -c’` it will ensure that translators cannot accidentally use format string directives that would lead to a crash at runtime. This option has an effect with most languages, namely C, C++, ObjectiveC, Shell, Python, Lisp, EmacsLisp, librep, Scheme, Java, C#, awk, YCP, Tcl, Perl, PHP, GCC-source. `‘-T’`: `‘--trigraphs’`: Understand ANSI C trigraphs for input. This option has an effect only with the languages C, C++, ObjectiveC. `‘--qt’`: Recognize Qt format strings. This option has an effect only with the language C++. `‘--kde’`: Recognize KDE 4 format strings. This option has an effect only with the language C++. `‘--boost’`: Recognize Boost format strings. This option has an effect only with the language C++. `‘--debug’`: Use the flags `c-format` and `possible-c-format` to show who was responsible for marking a message as a format string. The latter form is used if the `xgettext` program decided, the format form is used if the programmer prescribed it. By default only the `c-format` form is used. The translator should not have to care about these details.

This implementation of `xgettext` is able to process a few awkward
cases, like strings in preprocessor macros, ANSI concatenation of
adjacent strings, and escaped end of lines for continued strings.

### 5.1.7 Output details

`‘--color’`: `‘--color=when’`: Specify whether or when to use colors and other text attributes. See section 9.11.1 The `--color` option for details. `‘--style=style_file’`: Specify the CSS style rule file to use for `--color`. See section 9.11.3 The `--style` option for details. `‘--force-po’`: Always write an output file even if no message is defined. `‘-i’`: `‘--indent’`: Write the .po file using indented style. `‘--no-location’`: Do not write `‘#: filename:line’` lines. Note that using this option makes it harder for technically skilled translators to understand each message's context. `‘-n’`: `‘--add-location’`: Generate `‘#: filename:line’` lines (default). `‘--strict’`: Write out a strict Uniforum conforming PO file. Note that this Uniforum format should be avoided because it doesn't support the GNU extensions. `‘--properties-output’`: Write out a Java ResourceBundle in Java `.properties` syntax. Note that this file format doesn't support plural forms and silently drops obsolete messages. `‘--stringtable-output’`: Write out a NeXTstep/GNUstep localized resource file in `.strings` syntax. Note that this file format doesn't support plural forms. `‘-w number’`: `‘--width=number’`: Set the output page width. Long strings in the output files will be split across multiple lines in order to ensure that each line's width (= number of screen columns) is less or equal to the given number. `‘--no-wrap’`: Do not break long message lines. Message lines whose width exceeds the output page width will not be split into several lines. Only file reference lines which are wider than the output page width will be split. `‘-s’`: `‘--sort-output’`: Generate sorted output. Note that using this option makes it much harder for the translator to understand each message's context. `‘-F’`: `‘--sort-by-file’`: Sort output by file location. `‘--omit-header’`: Don't write header with `‘msgid ""’` entry. This is useful for testing purposes because it eliminates a source of variance for generated `.gmo` files. With `--omit-header`, two invocations of `xgettext` on the same files with the same options at different times are guaranteed to produce the same results. Note that using this option will lead to an error if the resulting file would not entirely be in ASCII. `‘--copyright-holder=string’`: Set the copyright holder in the output. string should be the copyright holder of the surrounding package. (Note that the msgstr strings, extracted from the package's sources, belong to the copyright holder of the package.) Translators are expected to transfer or disclaim the copyright for their translations, so that package maintainers can distribute them without legal risk. If string is empty, the output files are marked as being in the public domain; in this case, the translators are expected to disclaim their copyright, again so that package maintainers can distribute them without legal risk. The default value for string is the Free Software Foundation, Inc., simply because `xgettext` was first used in the GNU project. `‘--foreign-user’`: Omit FSF copyright in output. This option is equivalent to `‘--copyright-holder=”’`. It can be useful for packages outside the GNU project that want their translations to be in the public domain. `‘--package-name=package’`: Set the package name in the header of the output. `‘--package-version=version’`: Set the package version in the header of the output. This option has an effect only if the `‘--package-name’` option is also used. `‘--msgid-bugs-address=email@address’`: Set the reporting address for msgid bugs. This is the email address or URL to which the translators shall report bugs in the untranslated strings: - Strings which are not entire sentences, see the maintainer guidelines in section 4.3 Preparing Translatable Strings.- Strings which use unclear terms or require additional context to be understood.- Strings which make invalid assumptions about notation of date, time or money.- Pluralisation problems.- Incorrect English spelling.- Incorrect formatting. It can be your email address, or a mailing list address where translators can write to without being subscribed, or the URL of a web page through which the translators can contact you. The default value is empty, which means that translators will be clueless! Don't forget to specify this option. `‘-m[string]’`: `‘--msgstr-prefix[=string]’`: Use string (or "" if not specified) as prefix for msgstr values. `‘-M[string]’`: `‘--msgstr-suffix[=string]’`: Use string (or "" if not specified) as suffix for msgstr values.

### 5.1.8 Informative output

`‘-h’`: `‘--help’`: Display this help and exit. `‘-V’`: `‘--version’`: Output version information and exit.

---

Go to the first, previous, next, last section, table of contents.
