# Supplementary material for: rDock: A Fast, Versatile and Open Source Program for Docking Ligands to Proteins and Nucleic Acids
Source: PLoS Comput Biol. 2014 Apr 10;10(4):e1003571. doi: 10.1371/journal.pcbi.1003571 (PMC3983074; doi:10.1371/journal.pcbi.1003571)
Supplement: Software S1 — Compressed file with the source code of the rDock software for ligand docking to Proteins and Nucleic Acids. (GZ) [file pcbi.1003571.s010.gz › rDock_2013.1_src/gettext-0.18.tar.gz!/gettext-0.18/gettext-tools/doc/gettext_1.html]

GNU gettext utilities - 1 Introduction

Go to the first, previous, next, last section, table of contents.

---

# 1 Introduction

This chapter explains the goals sought in the creation
of GNU `gettext` and the free Translation Project.
Then, it explains a few broad concepts around
Native Language Support, and positions message translation with regard
to other aspects of national and cultural variance, as they apply
to programs. It also surveys those files used to convey the
translations. It explains how the various tools interact in the
initial generation of these files, and later, how the maintenance
cycle should usually operate.

In this manual, we use *he* when speaking of the programmer or
maintainer, *she* when speaking of the translator, and *they*
when speaking of the installers or end users of the translated program.
This is only a convenience for clarifying the documentation. It is
*absolutely* not meant to imply that some roles are more appropriate
to males or females. Besides, as you might guess, GNU `gettext`
is meant to be useful for people using computers, whatever their sex,
race, religion or nationality!

Please send suggestions and corrections to:

```
Internet address:
    bug-gnu-gettext@gnu.org
```

Please include the manual's edition number and update date in your messages.

## 1.1 The Purpose of GNU `gettext`

Usually, programs are written and documented in English, and use
English at execution time to interact with users. This is true
not only of GNU software, but also of a great deal of proprietary
and free software. Using a common language is quite handy for
communication between developers, maintainers and users from all
countries. On the other hand, most people are less comfortable with
English than with their own native language, and would prefer to
use their mother tongue for day to day's work, as far as possible.
Many would simply *love* to see their computer screen showing
a lot less of English, and far more of their own language.

However, to many people, this dream might appear so far fetched that
they may believe it is not even worth spending time thinking about
it. They have no confidence at all that the dream might ever
become true. Yet some have not lost hope, and have organized themselves.
The Translation Project is a formalization of this hope into a
workable structure, which has a good chance to get all of us nearer
the achievement of a truly multi-lingual set of programs.

GNU `gettext` is an important step for the Translation Project,
as it is an asset on which we may build many other steps. This package
offers to programmers, translators and even users, a well integrated
set of tools and documentation. Specifically, the GNU `gettext`
utilities are a set of tools that provides a framework within which
other free packages may produce multi-lingual messages. These tools
include

- A set of conventions about how programs should be written to support
  message catalogs.- A directory and file naming organization for the message catalogs
    themselves.- A runtime library supporting the retrieval of translated messages.- A few stand-alone programs to massage in various ways the sets of
        translatable strings, or already translated strings.- A library supporting the parsing and creation of files containing
          translated messages.- A special mode for Emacs(1) which helps preparing these sets
            and bringing them up to date.

GNU `gettext` is designed to minimize the impact of
internationalization on program sources, keeping this impact as small
and hardly noticeable as possible. Internationalization has better
chances of succeeding if it is very light weighted, or at least,
appear to be so, when looking at program sources.

The Translation Project also uses the GNU `gettext` distribution
as a vehicle for documenting its structure and methods. This goes
beyond the strict technicalities of documenting the GNU `gettext`
proper. By so doing, translators will find in a single place, as
far as possible, all they need to know for properly doing their
translating work. Also, this supplemental documentation might also
help programmers, and even curious users, in understanding how GNU
`gettext` is related to the remainder of the Translation
Project, and consequently, have a glimpse at the *big picture*.

## 1.2 I18n, L10n, and Such

Two long words appear all the time when we discuss support of native
language in programs, and these words have a precise meaning, worth
being explained here, once and for all in this document. The words are
*internationalization* and *localization*. Many people,
tired of writing these long words over and over again, took the
habit of writing *i18n* and *l10n* instead, quoting the first
and last letter of each word, and replacing the run of intermediate
letters by a number merely telling how many such letters there are.
But in this manual, in the sake of clarity, we will patiently write
the names in full, each time...

By *internationalization*, one refers to the operation by which a
program, or a set of programs turned into a package, is made aware of and
able to support multiple languages. This is a generalization process,
by which the programs are untied from calling only English strings or
other English specific habits, and connected to generic ways of doing
the same, instead. Program developers may use various techniques to
internationalize their programs. Some of these have been standardized.
GNU `gettext` offers one of these standards. See section 11 The Programmer's View.

By *localization*, one means the operation by which, in a set
of programs already internationalized, one gives the program all
needed information so that it can adapt itself to handle its input
and output in a fashion which is correct for some native language and
cultural habits. This is a particularisation process, by which generic
methods already implemented in an internationalized program are used
in specific ways. The programming environment puts several functions
to the programmers disposal which allow this runtime configuration.
The formal description of specific set of cultural habits for some
country, together with all associated translations targeted to the
same native language, is called the *locale* for this language
or country. Users achieve localization of programs by setting proper
values to special environment variables, prior to executing those
programs, identifying which locale should be used.

In fact, locale message support is only one component of the cultural
data that makes up a particular locale. There are a whole host of
routines and functions provided to aid programmers in developing
internationalized software and which allow them to access the data
stored in a particular locale. When someone presently refers to a
particular locale, they are obviously referring to the data stored
within that particular locale. Similarly, if a programmer is referring
to “accessing the locale routines”, they are referring to the
complete suite of routines that access all of the locale's information.

One uses the expression *Native Language Support*, or merely NLS,
for speaking of the overall activity or feature encompassing both
internationalization and localization, allowing for multi-lingual
interactions in a program. In a nutshell, one could say that
internationalization is the operation by which further localizations
are made possible.

Also, very roughly said, when it comes to multi-lingual messages,
internationalization is usually taken care of by programmers, and
localization is usually taken care of by translators.

## 1.3 Aspects in Native Language Support

For a totally multi-lingual distribution, there are many things to
translate beyond output messages.

- As of today, GNU `gettext` offers a complete toolset for
  translating messages output by C programs. Perl scripts and shell
  scripts will also need to be translated. Even if there are today some hooks
  by which this can be done, these hooks are not integrated as well as they
  should be.- Some programs, like `autoconf` or `bison`, are able
    to produce other programs (or scripts). Even if the generating
    programs themselves are internationalized, the generated programs they
    produce may need internationalization on their own, and this indirect
    internationalization could be automated right from the generating
    program. In fact, quite usually, generating and generated programs
    could be internationalized independently, as the effort needed is
    fairly orthogonal.- A few programs include textual tables which might need translation
      themselves, independently of the strings contained in the program
      itself. For example, RFC 1345 gives an English description for each
      character which the `recode` program is able to reconstruct at execution.
      Since these descriptions are extracted from the RFC by mechanical means,
      translating them properly would require a prior translation of the RFC
      itself.- Almost all programs accept options, which are often worded out so to
        be descriptive for the English readers; one might want to consider
        offering translated versions for program options as well.- Many programs read, interpret, compile, or are somewhat driven by
          input files which are texts containing keywords, identifiers, or
          replies which are inherently translatable. For example, one may want
          `gcc` to allow diacriticized characters in identifiers or use
          translated keywords; `‘rm -i’` might accept something else than
          `‘y’` or `‘n’` for replies, etc. Even if the program will
          eventually make most of its output in the foreign languages, one has
          to decide whether the input syntax, option values, etc., are to be
          localized or not.- The manual accompanying a package, as well as all documentation files
            in the distribution, could surely be translated, too. Translating a
            manual, with the intent of later keeping up with updates, is a major
            undertaking in itself, generally.

As we already stressed, translation is only one aspect of locales.
Other internationalization aspects are system services and are handled
in GNU `libc`. There
are many attributes that are needed to define a country's cultural
conventions. These attributes include beside the country's native
language, the formatting of the date and time, the representation of
numbers, the symbols for currency, etc. These local *rules* are
termed the country's locale. The locale represents the knowledge
needed to support the country's native attributes.

There are a few major areas which may vary between countries and
hence, define what a locale must describe. The following list helps
putting multi-lingual messages into the proper context of other tasks
related to locales. See the GNU `libc` manual for details.

*Characters and Codesets*: The codeset most commonly used through out the USA and most English speaking parts of the world is the ASCII codeset. However, there are many characters needed by various locales that are not found within this codeset. The 8-bit ISO 8859-1 code set has most of the special characters needed to handle the major European languages. However, in many cases, choosing ISO 8859-1 is nevertheless not adequate: it doesn't even handle the major European currency. Hence each locale will need to specify which codeset they need to use and will need to have the appropriate character handling routines to cope with the codeset. *Currency*: The symbols used vary from country to country as does the position used by the symbol. Software needs to be able to transparently display currency figures in the native mode for each locale. *Dates*: The format of date varies between locales. For example, Christmas day in 1994 is written as 12/25/94 in the USA and as 25/12/94 in Australia. Other countries might use ISO 8601 dates, etc. Time of the day may be noted as hh:mm, hh.mm, or otherwise. Some locales require time to be specified in 24-hour mode rather than as AM or PM. Further, the nature and yearly extent of the Daylight Saving correction vary widely between countries. *Numbers*: Numbers can be represented differently in different locales. For example, the following numbers are all written correctly for their respective locales: ``` 12,345.67 English 12.345,67 German 12345,67 French 1,2345.67 Asia ``` Some programs could go further and use different unit systems, like English units or Metric units, or even take into account variants about how numbers are spelled in full. *Messages*: The most obvious area is the language support within a locale. This is where GNU `gettext` provides the means for developers and users to easily change the language that the software uses to communicate to the user.

These areas of cultural conventions are called *locale categories*.
It is an unfortunate term; *locale aspects* or *locale feature
categories* would be a better term, because each “locale category”
describes an area or task that requires localization. The concrete data
that describes the cultural conventions for such an area and for a particular
culture is also called a *locale category*. In this sense, a locale
is composed of several locale categories: the locale category describing
the codeset, the locale category describing the formatting of numbers,
the locale category containing the translated messages, and so on.

Components of locale outside of message handling are standardized in
the ISO C standard and the POSIX:2001 standard (also known as the SUSV3
specification). GNU `libc`
fully implements this, and most other modern systems provide a more
or less reasonable support for at least some of the missing components.

## 1.4 Files Conveying Translations

The letters PO in ‘.po’ files means Portable Object, to
distinguish it from ‘.mo’ files, where MO stands for Machine
Object. This paradigm, as well as the PO file format, is inspired
by the NLS standard developed by Uniforum, and first implemented by
Sun in their Solaris system.

PO files are meant to be read and edited by humans, and associate each
original, translatable string of a given package with its translation
in a particular target language. A single PO file is dedicated to
a single target language. If a package supports many languages,
there is one such PO file per language supported, and each package
has its own set of PO files. These PO files are best created by
the `xgettext` program, and later updated or refreshed through
the `msgmerge` program. Program `xgettext` extracts all
marked messages from a set of C files and initializes a PO file with
empty translations. Program `msgmerge` takes care of adjusting
PO files between releases of the corresponding sources, commenting
obsolete entries, initializing new ones, and updating all source
line references. Files ending with ‘.pot’ are kind of base
translation files found in distributions, in PO file format.

MO files are meant to be read by programs, and are binary in nature.
A few systems already offer tools for creating and handling MO files
as part of the Native Language Support coming with the system, but the
format of these MO files is often different from system to system,
and non-portable. The tools already provided with these systems don't
support all the features of GNU `gettext`. Therefore GNU
`gettext` uses its own format for MO files. Files ending with
‘.gmo’ are really MO files, when it is known that these files use
the GNU format.

## 1.5 Overview of GNU `gettext`

The following diagram summarizes the relation between the files
handled by GNU `gettext` and the tools acting on these files.
It is followed by somewhat detailed explanations, which you should
read while keeping an eye on the diagram. Having a clear understanding
of these interrelations will surely help programmers, translators
and maintainers.

@example
@group
Original C Sources ───> Preparation ───> Marked C Sources ───╮
│
╭─────────<─── GNU gettext Library │
╭─── make <───┤ │
│ ╰─────────<────────────────────┬───────────────╯
│ │
│ ╭─────<─── PACKAGE.pot <─── xgettext <───╯ ╭───<─── PO Compendium
│ │ │ ↑
│ │ ╰───╮ │
│ ╰───╮ ├───> PO editor ───╮
│ ├────> msgmerge ──────> LANG.po ────>────────╯ │
│ ╭───╯ │
│ │ │
│ ╰─────────────<───────────────╮ │
│ ├─── New LANG.po <────────────────────╯
│ ╭─── LANG.gmo <─── msgfmt <───╯
│ │
│ ╰───> install ───> /.../LANG/PACKAGE.mo ───╮
│ ├───> "Hello world!"
╰───────> install ───> /.../bin/PROGRAM ───────╯
@end group
@end example

As a programmer, the first step to bringing GNU `gettext`
into your package is identifying, right in the C sources, those strings
which are meant to be translatable, and those which are untranslatable.
This tedious job can be done a little more comfortably using emacs PO
mode, but you can use any means familiar to you for modifying your
C sources. Beside this some other simple, standard changes are needed to
properly initialize the translation library. See section 4 Preparing Program Sources, for
more information about all this.

For newly written software the strings of course can and should be
marked while writing it. The `gettext` approach makes this
very easy. Simply put the following lines at the beginning of each file
or in a central header file:

```
#define _(String) (String)
#define N_(String) String
#define textdomain(Domain)
#define bindtextdomain(Package, Directory)
```

Doing this allows you to prepare the sources for internationalization.
Later when you feel ready for the step to use the `gettext` library
simply replace these definitions by the following:

```
#include <libintl.h>
#define _(String) gettext (String)
#define gettext_noop(String) String
#define N_(String) gettext_noop (String)
```

and link against ‘libintl.a’ or ‘libintl.so’. Note that on
GNU systems, you don't need to link with `libintl` because the
`gettext` library functions are already contained in GNU libc.
That is all you have to change.

Once the C sources have been modified, the `xgettext` program
is used to find and extract all translatable strings, and create a
PO template file out of all these. This ‘package.pot’ file
contains all original program strings. It has sets of pointers to
exactly where in C sources each string is used. All translations
are set to empty. The letter `t` in ‘.pot’ marks this as
a Template PO file, not yet oriented towards any particular language.
See section 5.1 Invoking the `xgettext` Program, for more details about how one calls the
`xgettext` program. If you are *really* lazy, you might
be interested at working a lot more right away, and preparing the
whole distribution setup (see section 13 The Maintainer's View). By doing so, you
spare yourself typing the `xgettext` command, as `make`
should now generate the proper things automatically for you!

The first time through, there is no ‘lang.po’ yet, so the
`msgmerge` step may be skipped and replaced by a mere copy of
‘package.pot’ to ‘lang.po’, where lang
represents the target language. See section 6 Creating a New PO File for details.

Then comes the initial translation of messages. Translation in
itself is a whole matter, still exclusively meant for humans,
and whose complexity far overwhelms the level of this manual.
Nevertheless, a few hints are given in some other chapter of this
manual (see section 12 The Translator's View). You will also find there indications
about how to contact translating teams, or becoming part of them,
for sharing your translating concerns with others who target the same
native language.

While adding the translated messages into the ‘lang.po’
PO file, if you are not using one of the dedicated PO file editors
(see section 8 Editing PO Files), you are on your own
for ensuring that your efforts fully respect the PO file format, and quoting
conventions (see section 3 The Format of PO Files). This is surely not an impossible task,
as this is the way many people have handled PO files around 1995.
On the other hand, by using a PO file editor, most details
of PO file format are taken care of for you, but you have to acquire
some familiarity with PO file editor itself.

If some common translations have already been saved into a compendium
PO file, translators may use PO mode for initializing untranslated
entries from the compendium, and also save selected translations into
the compendium, updating it (see section 8.4 Using Translation Compendia). Compendium files
are meant to be exchanged between members of a given translation team.

Programs, or packages of programs, are dynamic in nature: users write
bug reports and suggestion for improvements, maintainers react by
modifying programs in various ways. The fact that a package has
already been internationalized should not make maintainers shy
of adding new strings, or modifying strings already translated.
They just do their job the best they can. For the Translation
Project to work smoothly, it is important that maintainers do not
carry translation concerns on their already loaded shoulders, and that
translators be kept as free as possible of programming concerns.

The only concern maintainers should have is carefully marking new
strings as translatable, when they should be, and do not otherwise
worry about them being translated, as this will come in proper time.
Consequently, when programs and their strings are adjusted in various
ways by maintainers, and for matters usually unrelated to translation,
`xgettext` would construct ‘package.pot’ files which are
evolving over time, so the translations carried by ‘lang.po’
are slowly fading out of date.

It is important for translators (and even maintainers) to understand
that package translation is a continuous process in the lifetime of a
package, and not something which is done once and for all at the start.
After an initial burst of translation activity for a given package,
interventions are needed once in a while, because here and there,
translated entries become obsolete, and new untranslated entries
appear, needing translation.

The `msgmerge` program has the purpose of refreshing an already
existing ‘lang.po’ file, by comparing it with a newer
‘package.pot’ template file, extracted by `xgettext`
out of recent C sources. The refreshing operation adjusts all
references to C source locations for strings, since these strings
move as programs are modified. Also, `msgmerge` comments out as
obsolete, in ‘lang.po’, those already translated entries
which are no longer used in the program sources (see section 8.3.8 Obsolete Entries). It finally discovers new strings and inserts them in
the resulting PO file as untranslated entries (see section 8.3.7 Untranslated Entries). See section 7.1 Invoking the `msgmerge` Program, for more information about what
`msgmerge` really does.

Whatever route or means taken, the goal is to obtain an updated
‘lang.po’ file offering translations for all strings.

The temporal mobility, or fluidity of PO files, is an integral part of
the translation game, and should be well understood, and accepted.
People resisting it will have a hard time participating in the
Translation Project, or will give a hard time to other participants! In
particular, maintainers should relax and include all available official
PO files in their distributions, even if these have not recently been
updated, without exerting pressure on the translator teams to get the
job done. The pressure should rather come
from the community of users speaking a particular language, and
maintainers should consider themselves fairly relieved of any concern
about the adequacy of translation files. On the other hand, translators
should reasonably try updating the PO files they are responsible for,
while the package is undergoing pretest, prior to an official
distribution.

Once the PO file is complete and dependable, the `msgfmt` program
is used for turning the PO file into a machine-oriented format, which
may yield efficient retrieval of translations by the programs of the
package, whenever needed at runtime (see section 10.3 The Format of GNU MO Files). See section 10.1 Invoking the `msgfmt` Program, for more information about all modes of execution
for the `msgfmt` program.

Finally, the modified and marked C sources are compiled and linked
with the GNU `gettext` library, usually through the operation of
`make`, given a suitable ‘Makefile’ exists for the project,
and the resulting executable is installed somewhere users will find it.
The MO files themselves should also be properly installed. Given the
appropriate environment variables are set (see section 2.3 Setting the Locale through Environment Variables),
the program should localize itself automatically, whenever it executes.

The remainder of this manual has the purpose of explaining in depth the various
steps outlined above.

---

Go to the first, previous, next, last section, table of contents.
