# Supplementary material for: rDock: A Fast, Versatile and Open Source Program for Docking Ligands to Proteins and Nucleic Acids
Source: PLoS Comput Biol. 2014 Apr 10;10(4):e1003571. doi: 10.1371/journal.pcbi.1003571 (PMC3983074; doi:10.1371/journal.pcbi.1003571)
Supplement: Software S1 — Compressed file with the source code of the rDock software for ligand docking to Proteins and Nucleic Acids. (GZ) [file pcbi.1003571.s010.gz › rDock_2013.1_src/gettext-0.18.tar.gz!/gettext-0.18/gettext-tools/doc/gettext_23.html]

GNU gettext utilities - PO Mode Index

Go to the first, previous, next, last section, table of contents.

---

# PO Mode Index

Jump to:
&
-
a
-
c
-
e
-
f
-
i
-
l
-
m
-
o
-
p
-
s
-
u

## &

- ‘.emacs’ customizations- ‘TAGS’, and marking translatable strings

## a

- auxiliary PO file

## c

- commands- comment out PO file entry- consulting program sources- consulting translations to other languages- current entry of a PO file- cut and paste for translated strings

## e

- editing comments- editing multiple entries- editing translations- `etags`, using for marking strings- exiting PO subedit

## f

- find source fragment for a PO file entry

## i

- installing PO mode

## l

- looking at the source to aid translation

## m

- marking strings for translation- moving by fuzzy entries- moving by obsolete entries- moving by translated entries- moving by untranslated entries- moving through a PO file

## o

- obsolete active entry

## p

- pending subedits

## s

- starting a string translation- string normalization in entries- subedit minor mode

## u

- use the source, Luke- using obsolete translations to make new entries- using translation compendia

---

Go to the first, previous, next, last section, table of contents.
