# Supplementary material for: rDock: A Fast, Versatile and Open Source Program for Docking Ligands to Proteins and Nucleic Acids
Source: PLoS Comput Biol. 2014 Apr 10;10(4):e1003571. doi: 10.1371/journal.pcbi.1003571 (PMC3983074; doi:10.1371/journal.pcbi.1003571)
Supplement: Software S1 — Compressed file with the source code of the rDock software for ligand docking to Proteins and Nucleic Acids. (GZ) [file pcbi.1003571.s010.gz › rDock_2013.1_src/gettext-0.18.tar.gz!/gettext-0.18/gettext-tools/doc/gettext_24.html]

GNU gettext utilities - Autoconf Macro Index

Go to the first, previous, next, last section, table of contents.

---

# Autoconf Macro Index

Jump to:
a

## a

- AM\_GNU\_GETTEXT- AM\_GNU\_GETTEXT\_INTL\_SUBDIR- AM\_GNU\_GETTEXT\_NEED- AM\_GNU\_GETTEXT\_VERSION- AM\_ICONV- AM\_PO\_SUBDIRS- AM\_XGETTEXT\_OPTION

---

Go to the first, previous, next, last section, table of contents.
