# Supplementary material for: rDock: A Fast, Versatile and Open Source Program for Docking Ligands to Proteins and Nucleic Acids
Source: PLoS Comput Biol. 2014 Apr 10;10(4):e1003571. doi: 10.1371/journal.pcbi.1003571 (PMC3983074; doi:10.1371/journal.pcbi.1003571)
Supplement: Software S1 — Compressed file with the source code of the rDock software for ligand docking to Proteins and Nucleic Acids. (GZ) [file pcbi.1003571.s010.gz › rDock_2013.1_src/gettext-0.18.tar.gz!/gettext-0.18/gettext-tools/doc/gettext_14.html]

GNU gettext utilities - 14 The Installer's and Distributor's View

Go to the first, previous, next, last section, table of contents.

---

# 14 The Installer's and Distributor's View

By default, packages fully using GNU `gettext`, internally,
are installed in such a way that they to allow translation of
messages. At *configuration* time, those packages should
automatically detect whether the underlying host system already provides
the GNU `gettext` functions. If not,
the GNU `gettext` library should be automatically prepared
and used. Installers may use special options at configuration
time for changing this behavior. The command `‘./configure
--with-included-gettext’` bypasses system `gettext` to
use the included GNU `gettext` instead,
while `‘./configure --disable-nls’`
produces programs totally unable to translate messages.

Internationalized packages have usually many ‘ll.po’
files. Unless
translations are disabled, all those available are installed together
with the package. However, the environment variable `LINGUAS`
may be set, prior to configuration, to limit the installed set.
`LINGUAS` should then contain a space separated list of two-letter
codes, stating which languages are allowed.

---

Go to the first, previous, next, last section, table of contents.
