# Supplementary material for: rDock: A Fast, Versatile and Open Source Program for Docking Ligands to Proteins and Nucleic Acids
Source: PLoS Comput Biol. 2014 Apr 10;10(4):e1003571. doi: 10.1371/journal.pcbi.1003571 (PMC3983074; doi:10.1371/journal.pcbi.1003571)
Supplement: Software S1 — Compressed file with the source code of the rDock software for ligand docking to Proteins and Nucleic Acids. (GZ) [file pcbi.1003571.s010.gz › rDock_2013.1_src/gettext-0.18.tar.gz!/gettext-0.18/gettext-tools/doc/gettext_foot.html]

GNU gettext utilities - Footnotes


# GNU gettext tools, version 0.18

## Native Language Support Library and Tools

## Edition 0.18, 25 April 2010

Ulrich Drepper
Jim Meyering
François Pinard
Bruno Haible

---

### (1)

In this manual, all mentions of Emacs
refers to either GNU Emacs or to XEmacs, which people sometimes call FSF
Emacs and Lucid Emacs, respectively.

### (2)

This
limitation is not imposed by GNU `gettext`, but is for compatibility
with the `msgfmt` implementation on Solaris.

### (3)

Some
system, e.g. mingw, don't have `LC_MESSAGES`. Here we use a more or
less arbitrary value for it, namely 1729, the smallest positive integer
which can be represented in two different ways as the sum of two cubes.

### (4)

When the system does not support `setlocale` its behavior
in setting the locale values is simulated by looking at the environment
variables.

### (5)

Additions are welcome. Send appropriate information to
bug-gnu-gettext@gnu.org and bug-glibc-manual@gnu.org.

---

This document was generated on 9 May 2010 using the
texi2html
translator version 1.52b.
