# Supplementary material for: rDock: A Fast, Versatile and Open Source Program for Docking Ligands to Proteins and Nucleic Acids
Source: PLoS Comput Biol. 2014 Apr 10;10(4):e1003571. doi: 10.1371/journal.pcbi.1003571 (PMC3983074; doi:10.1371/journal.pcbi.1003571)
Supplement: Software S1 — Compressed file with the source code of the rDock software for ligand docking to Proteins and Nucleic Acids. (GZ) [file pcbi.1003571.s010.gz › rDock_2013.1_src/gettext-0.18.tar.gz!/gettext-0.18/gettext-tools/doc/tutorial.html]

A tutorial on Native Language Support using GNU gettext


# A tutorial on Native Language Support using GNU gettext

**G. Mohanty**

**Revision 0.3: 24 July 2004**

### Abstract:

The use of the GNU gettext utilities to implement support for native
languages is described here. Though, the language to be supported is
considered to be Oriya, the method is generally applicable. Likewise, while
Linux was used as the platform here, any system using GNU gettext should work
in a similar fashion.

We go through a step-by-step description of how to make on-screen messages
from a toy program to appear in Oriya instead of English; starting from the
programming and ending with the user's viewpoint. Some discussion is also made
of how to go about the task of translation.

# Introduction

Currently, both commercial and free computer software is typically written and
documented in English. Till recently, little effort was expended towards
allowing them to interact with the user in languages other than English, thus
leaving the non-English speaking world at a disadvantage. However, that
changed with the release of the GNU gettext utilities, and nowadays most GNU
programs are written within a framework that allows easy translation of the
program message to languages other than English. Provided that translations
are available, the language used by the program to interact with the user can
be set at the time of running it. gettext manages to achieve this seemingly
miraculous task in a manner that simplifies the work of both the programmer
and the translator, and, more importantly, allows them to work independently
of each other.

This article describes how to support native languages under a system using
the GNU gettext utilities. While it should be applicable to other versions of
gettext, the one actually used for the examples here is version
0.12.1. Another system, called catgets, described in the X/Open
Portability Guide, is also in use, but we shall not discuss that here.

# A simple example

Our first example of using gettext will be the good old Hello World program,
whose sole function is to print the phrase ``Hello, world!'' to the terminal.
The internationalized version of this program might be saved in hello.c as:

```
1    #include <libintl.h>
2    #include <locale.h>
3    #include <stdio.h>
4    #include <stdlib.h>
5    int main(void)
6    {
7      setlocale( LC_ALL, "" );
8      bindtextdomain( "hello", "/usr/share/locale" );
9      textdomain( "hello" );
10      printf( gettext( "Hello, world!\n" ) );
11      exit(0);
12    }
```

Of course, a real program would check the return values of the functions and
try to deal with any errors, but we have omitted that part of the code for
clarity. Compile as usual with gcc -o hello hello.c. The program should
be linked to the GNU libintl library, but as this is part of the GNU C
library, this is done automatically for you under Linux, and other systems
using glibc.

## The programmer's viewpoint

As expected, when the hello executable is run under the default locale
(usually the C locale) it prints ``Hello, world!'' in the terminal. Besides
some initial setup work, the only additional burden faced by the programmer is
to replace any string to be printed with gettext(string), i.e., to
instead pass the string as an argument to the gettext function. For lazy
people like myself, the amount of extra typing can be reduced even further by
a CPP macro, e.g., put this at the beginning of the source code file,

```
  #define _(STRING)    gettext(STRING)
```

and then use \_(string) instead of gettext(string).

Let us dissect the program line-by-line.

1. locale.h defines C data structures used to hold locale
   information, and is needed by the setlocale function. libintl.h
   prototypes the GNU text utilities functions, and is needed here by
   bindtextdomain, gettext, and textdomain.
2. The call to setlocale () on line 7, with LC\_ALL as the first argument
   and an empty string as the second one, initializes the entire current locale
   of the program as per environment variables set by the user. In other words,
   the program locale is initialized to match that of the user. For details see
   ``man setlocale.''
3. The bindtextdomain function on line 8 sets the base directory for the
   message catalogs for a given message domain. A message domain is a set of
   translatable messages, with every software package typically having its own
   domain. Here, we have used ``hello'' as the name of the message domain for
   our toy program. As the second argument, /usr/share/locale, is the default
   system location for message catalogs, what we are saying here is that we are
   going to place the message catalog in the default system directory. Thus, we
   could have dispensed with the call to bindtextdomain here, and this
   function is useful only if the message catalogs are installed in a
   non-standard place, e.g., a packaged software distribution might have
   the catalogs under a po/ directory under its own main directory. See ``man
   bindtextdomain'' for details.
4. The textdomain call on line 9 sets the message domain of the current
   program to ``hello,'' i.e., the name that we are using for our example
   program. ``man textdomain'' will give usage details for the function.
5. Finally, on line 10, we have replaced what would normally have been,

   ```
     printf( "Hello, world!\n" );
   ```

   with,

   ```
     printf( gettext( "Hello, world!\n" ) );
   ```

   (If you are unfamiliar with C, the 
   \n at the end of the string
   produces a newline at the end of the output.) This simple modification to all
   translatable strings allows the translator to work independently from the
   programmer. gettextize eases the task of the programmer in adapting a
   package to use GNU gettext for the first time, or to upgrade to a newer
   version of gettext.

## Extracting translatable strings

Now, it is time to extract the strings to be translated from the program
source code. This is achieved with xgettext, which can be invoked as follows:

```
  xgettext -d hello -o hello.pot hello.c
```

This processes the source code in hello.c, saving the output in hello.pot (the
argument to the -o option).
The message domain for the program should be specified as the argument
to the -d option, and should match the domain specified in the call to
textdomain (on line 9 of the program source). Other details on how to use
gettext can be found from ``man gettext.''

A .pot (portable object template) file is used as the basis for translating
program messages into any language. To start translation, one can simply copy
hello.pot to oriya.po (this preserves the template file for later translation
into a different language). However, the preferred way to do this is by
use of the msginit program, which takes care of correctly setting up some
default values,

```
  msginit -l or_IN -o oriya.po -i hello.pot
```

Here, the -l option defines the locale (an Oriya locale should have been
installed on your system), and the -i and -o options define the input and
output files, respectively. If there is only a single .pot file in the
directory, it will be used as the input file, and the -i option can be
omitted. For me, the oriya.po file produced by msginit would look like:

```
  # Oriya translations for PACKAGE package.
  # Copyright (C) 2004 THE PACKAGE'S COPYRIGHT HOLDER
  # This file is distributed under the same license as the PACKAGE package.
  # Gora Mohanty <gora_mohanty@yahoo.co.in>, 2004.
  #
  msgid ""
  msgstr ""
  "Project-Id-Version: PACKAGE VERSION\n"
  "Report-Msgid-Bugs-To: \n"
  "POT-Creation-Date: 2004-06-22 02:22+0530\n"
  "PO-Revision-Date: 2004-06-22 02:38+0530\n"
  "Last-Translator: Gora Mohanty <gora_mohanty@yahoo.co.in>\n"
  "Language-Team: Oriya\n"
  "MIME-Version: 1.0\n"
  "Content-Type: text/plain; charset=UTF-8\n"
  "Content-Transfer-Encoding: 8bit\n"
 
  #: hello.c:10
  msgid "Hello, world!\n"
  msgstr ""
```

msginit prompted for my email address, and probably obtained my real name
from the system password file. It also filled in values such as the revision
date, language, character set, presumably using information from the or\_IN
locale.

It is important to respect the format of the entries in the .po (portable
object) file. Each entry has the following structure:

```
  WHITE-SPACE
  #  TRANSLATOR-COMMENTS
  #. AUTOMATIC-COMMENTS
  #: REFERENCE...
  #, FLAG...
  msgid UNTRANSLATED-STRING
  msgstr TRANSLATED-STRING
```

where, the initial white-space (spaces, tabs, newlines,...), and all
comments might or might not exist for a particular entry. Comment lines start
with a '#' as the first character, and there are two kinds: (i) manually
added translator comments, that have some white-space immediately following the
'#,' and (ii) automatic comments added and maintained by the gettext tools,
with a non-white-space character after the '#.' The msgid line contains
the untranslated (English) string, if there is one for that PO file entry, and
the msgstr line is where the translated string is to be entered. More on
this later. For details on the format of PO files see gettext::Basics::PO
Files:: in the Emacs info-browser (see Appdx. A for an
introduction to using the info-browser in Emacs).

## Making translations

The oriya.po file can then be edited to add the translated Oriya
strings. While the editing can be carried out in any editor if one is careful
to follow the PO file format, there are several editors that ease the task of
editing PO files, among them being po-mode in Emacs, kbabel, gtranslator,
poedit, etc. Appdx. B describes features of some of
these editors.

The first thing to do is fill in the comments at the beginning and the header
entry, parts of which have already been filled in by msginit. The lines in
the header entry are pretty much self-explanatory, and details can be found in
the gettext::Creating::Header Entry:: info node. After that, the remaining
work consists of typing the Oriya text that is to serve as translations for
the corresponding English string. For the msgstr line in each of the
remaining entries, add the translated Oriya text between the double quotes;
the translation corresponding to the English phrase in the msgid string
for the entry. For example, for the phrase ``Hello world!
\n'' in
oriya.po, we could enter ``ନମସ୍କାର
\n''. The final
oriya.po file might look like:

```
  # Oriya translations for hello example package.
  # Copyright (C) 2004 Gora Mohanty
  # This file is distributed under the same license as the hello example package.
  # Gora Mohanty <gora_mohanty@yahoo.co.in>, 2004.
  #
  msgid ""
  msgstr ""
  "Project-Id-Version: oriya\n"
  "Report-Msgid-Bugs-To: \n"
  "POT-Creation-Date: 2004-06-22 02:22+0530\n"
  "PO-Revision-Date: 2004-06-22 10:54+0530\n"
  "Last-Translator: Gora Mohanty <gora_mohanty@yahoo.co.in>\n"
  "Language-Team: Oriya\n"
  "MIME-Version: 1.0\n"
  "Content-Type: text/plain; charset=UTF-8\n"
  "Content-Transfer-Encoding: 8bit\n"
  "X-Generator: KBabel 1.3\n"

  #: hello.c:10
  msgid "Hello, world!\n"
  msgstr "ନମସ୍କାର\n"
```

For editing PO files, I have found the kbabel editor suits me the best. The
only problem is that while Oriya text can be entered directly into kbabel
using the xkb Oriya keyboard layouts [1] and the entries
are saved properly, the text is not displayed correctly in the kbabel window
if it includes conjuncts. Emacs po-mode is a little restrictive, but strictly
enforces conformance with the PO file format. The main problem with it is that
it does not seem currently possible to edit Oriya text in Emacs. yudit
is the best at editing Oriya text, but does not ensure that the PO file format
is followed. You can play around a bit with these editors to find one that
suits your personal preferences. One possibility might be to first edit the
header entry with kbabel or Emacs po-mode, and then use yudit to enter
the Oriya text on the msgstr lines.

## Message catalogs

After completing the translations in the oriya.po file, it must be compiled to
a binary format that can be quickly loaded by the gettext tools. To do that,
use:

```
  msgfmt -c -v -o hello.mo oriya.po
```

The -c option does detailed checking of the PO file format, -v makes the
program verbose, and the output filename is given by the argument to the -o
option. Note that the base of the output filename should match the message
domain given in the first arguments to bindtextdomain and textdomain on
lines 8 and 9 of the example program in Sec. 2. The .mo
(machine object) file should be stored in the location whose base directory is
given by the second argument to bindtextdomain. The final location of the
file will be in the sub-directory LL/LC\_MESSAGES or LL\_CC/LC\_MESSAGES under
the base directory, where LL stands for a language, and CC for a country. For
example, as we have chosen the standard location, /usr/share/locale, for our
base directory, and for us the language and country strings are ``or'' and
``IN,'' respectively, we will place hello.mo in /usr/share/locale/or\_IN. Note
that you will need super-user privilege to copy hello.mo to this system
directory. Thus,

```
  mkdir -p /usr/share/locale/or_IN/LC_MESSAGES
  cp hello.mo /usr/share/locale/or_IN/LC_MESSAGES
```

## The user's viewpoint

Once the message catalogs have been properly installed, any user on the system
can use the Oriya version of the Hello World program, provided an Oriya locale
is available. First, change your locale with,

```
  echo $LANG
  export LANG=or_IN
```

The first statement shows you the current setting of your locale (this is
usually en\_US, and you will need it to reset the default locale at the end),
while the second one sets it to an Oriya locale.

A Unicode-capable terminal emulator is needed to view Oriya output
directly. The new versions of both gnome-terminal and konsole (the KDE
terminal emulator) are Unicode-aware. I will focus on gnome-terminal as it
seems to have better support for internationalization. gnome-terminal needs to
be told that the bytes arriving are UTF-8 encoded multibyte sequences. This
can be done by (a) choosing Terminal -> Character Coding ->
Unicode (UTF-8), or (b) typing ``/bin/echo -n -e
'
\033%
\G''' in the terminal, or (c) by running
/bin/unicode\_start. Likewise, you can revert to the default locale by (a)
choosing Terminal -> Character Coding -> Current Locale
(ISO-8859-1), or (b) ``/bin/echo -n -e '
\033%
\@','' or
(c) by running /bin/unicode\_stop. Now, running the example program (after
compiling with gcc as described in Sec. 2) with,

```
  ./hello
```

should give you output in Oriya. Please note that conjuncts will most likely
be displayed with a ``halant'' as the terminal probably does not render Indian
language fonts correctly. Also, as most terminal emulators assume fixed-width
fonts, the results are hardly likely to be aesthetically appealing.

An alternative is to save the program output in a file, and view it with
yudit which will render the glyphs correctly. Thus,

```
  ./hello > junk
  yudit junk
```

Do not forget to reset the locale before resuming usual work in the
terminal. Else, your English characters might look funny.

While all this should give the average user some pleasure in being able to see
Oriya output from a program without a whole lot of work, it should be kept in
mind that we are still far from our desired goal. Hopefully, one day the
situation will be such that rather than deriving special pleasure from it,
users take it for granted that Oriya should be available and are upset
otherwise.

# Adding complications: program upgrade

The previous section presented a simple example of how Oriya language support
could be added to a C program. Like all programs, we might now wish to further
enhance it. For example, we could include a greeting to the user by adding
another printf statement after the first one. Our new hello.c source
code might look like this:

```
1    #include <libintl.h>
2    #include <locale.h>
3    #include <stdio.h>
4    #include <stdlib.h>
5    int main(void)
6    {
7      setlocale( LC_ALL, "" );
8      bindtextdomain( "hello", "/usr/share/locale" );
9      textdomain( "hello" );
10      printf( gettext( "Hello, world!\n" ) );
11      printf( gettext( "How are you\n" ) );
12      exit(0);
13    }
```

For such a small change, it would be simple enough to just repeat the above
cycle of extracting the relevant English text, translating it to Oriya, and
preparing a new message catalog. We can even simplify the work by cutting and
pasting most of the old oriya.po file into the new one. However, real programs
will have thousands of such strings, and we would like to be able to translate
only the changed strings, and have the gettext utilities handle the drudgery
of combining the new translations with the old ones. This is indeed possible.

## Merging old and new translations

As before, extract the translatable strings from hello.c to a new portable
object template file, hello-new.pot, using xgettext,

```
  xgettext -d hello -o hello-new.pot hello.c
```

Now, we use a new program, msgmerge, to merge the existing .po file with
translations into the new template file, viz.,

```
  msgmerge -U oriya.po hello-new.pot
```

The -U option updates the existing
.po file, oriya.po. We could have chosen to instead create a new .po file by
using ``-o <filename>'' instead of -U. The updated .po file will still
have the old translations embedded in it, and new entries with untranslated
msgid lines. For us, the new lines in oriya.po will look like,

```
  #: hello.c:11
  msgid "How are you?\n"
  msgstr ""
```

For the new translation, we could use, ``ଆପଣ
କିପରି ଅଛନ୍ତି?'' in
place of the English phrase ``How are you?'' The updated oriya.po file,
including the translation might look like:

```
  # Oriya translations for hello example package.
  # Copyright (C) 2004 Gora Mohanty
  # This file is distributed under the same license as the hello examplepackage.
  # Gora Mohanty <gora_mohanty@yahoo.co.in>, 2004.
  #
  msgid ""
  msgstr ""
  "Project-Id-Version: oriya\n"
  "Report-Msgid-Bugs-To: \n"
  "POT-Creation-Date: 2004-06-23 14:30+0530\n"
  "PO-Revision-Date: 2004-06-22 10:54+0530\n"
  "Last-Translator: Gora Mohanty <gora_mohanty@yahoo.co.in>\n"
  "Language-Team: Oriya\n"
  "MIME-Version: 1.0\n"
  "Content-Type: text/plain; charset=UTF-8\n"
  "Content-Transfer-Encoding: 8bit\n"
  "X-Generator: KBabel 1.3\n"
  
  #: hello.c:10
  msgid "Hello, world!\n"
  msgstr "ନମସ୍କାର\n"

  #: hello.c:11
  msgid "How are you?\n"
  msgstr "ଆପଣ କିପରି ଅଛନ୍ତି?\n"
```

Compile oriya.po to a machine object file, and install in the appropriate
place as in Sec. 2.4. Thus,

```
  msgfmt -c -v -o hello.mo oriya.po
  mkdir -p /usr/share/locale/or_IN/LC_MESSAGES
  cp hello.mo /usr/share/locale/or_IN/LC_MESSAGES
```

You can test the Oriya output as above, after recompiling hello.c and running
it in an Oriya locale.

# More about gettext

The GNU gettext info pages provide a well-organized and complete description
of the gettext utilities and their usage for enabling Native Language
Support. One should, at the very least, read the introductory material at
gettext::Introduction::, and the suggested references in
gettext::Conclusion::References::. Besides the gettext utilities described in
this document, various other programs to manipulate .po files are discussed in
gettext:Manipulating::. Finally, support for programming languages other than
C/C++ is discussed in gettext::Programming Languages::.

# The work of translation

Besides the obvious program message strings that have been the sole focus of
our discussion here, there are many other things that require translation,
including GUI messages, command-line option strings, configuration files,
program documentation, etc. Besides these obvious aspects, there are a
significant number of programs and/or scripts that are automatically generated
by other programs. These generated programs might also themselves require
translation. So, in any effort to provide support for a given native language,
carrying out the translation and keeping up with program updates becomes a
major part of the undertaking, requiring a continuing commitment from the
language team. A plan has been outlined for the Oriya localization
project [2].

# Acknowledgments

Extensive use has obviously been made of the GNU gettext manual in preparing
this document. I have also been helped by an article in the Linux
Journal [3].

This work is part of the project for enabling the use of Oriya under Linux. I
thank my uncle, N. M. Pattnaik, for conceiving of the project. We have all
benefited from the discussions amidst the group of people working on this
project. On the particular issue of translation, the help of H. R. Pansari,
A. Nayak, and M. Chand is much appreciated.

# The Emacs info browser

You can start up Emacs from the command-line by typing ``emacs,'' or ``emacs
<filename>.'' It can be started from the menu in some desktops, e.g., on
my GNOME desktop, it is under Main Menu -> Programming ->
Emacs. If you are unfamiliar with Emacs, a tutorial can be started by typing
``C-h t'' in an Emacs window, or from the Help item in the menubar at the
top. Emacs makes extensive use of the Control (sometimes labelled as ``CTRL''
or ``CTL'') and Meta (sometimes labelled as ``Edit'' or ``Alt'') keys. In
Emacs parlance, a hyphenated sequence, such as ``C-h'' means to press the
Control and `h' key simultaneously, while ``C-h t'' would mean to press the
Control and `h' key together, release them, and press the `t' key. Similarly,
``M-x'' is used to indicate that the Meta and `x' keys should be pressed at
the same time.

The info browser can be started by typing ``C-h i'' in Emacs. The first time
you do this, it will briefly list some commands available inside the info
browser, and present you with a menu of major topics. Each menu item, or
cross-reference is hyperlinked to the appropriate node, and you can visit that
node either by moving the cursor to the item and pressing Enter, or by
clicking on it with the middle mouse button. To get to the gettext menu items,
you can either scroll down to the line,

```
  * gettext: (gettext).                          GNU gettext utilities.
```

and visit that node. Or, as it is several pages down, you can locate it using
``I-search.'' Type ``C-s'' to enter ``I-search'' which will then prompt you
for a string in the mini-buffer at the bottom of the window. This is an
incremental search, so that Emacs will keep moving you forward through the
buffer as you are entering your search string. If you have reached the last
occurrence of the search string in the current buffer, you will get a message
saying ``Failing I-search: ...'' on pressing ``C-s.'' At that point, press
``C-s'' again to resume the search at the beginning of the buffer. Likewise,
``C-r'' incrementally searches backwards from the present location.

Info nodes are listed in this document with a ``::'' separator, so
that one can go to the gettext::Creating::Header Entry:: by visiting the
``gettext'' node from the main info menu, navigating to the ``Creating''
node, and following that to the ``Header Entry'' node.

A stand-alone info browser, independent of Emacs, is also available on many
systems. Thus, the gettext info page can also be accessed by typing
``info gettext'' in a terminal. xinfo is an X application serving as an
info browser, so that if it is installed, typing ``xinfo gettext'' from the
command line will open a new browser window with the gettext info page.

# PO file editors

While the yudit editor is adequate for our present purposes, and we are
planning on using that as it is platform-independent, and currently the best
at rendering Oriya. This section describes some features of some editors that
are specialized for editing PO files under Linux. This is still work in
progress, as I am in the process of trying out different editors before
settling on one. The ones considered here are: Emacs in po-mode, poedit,
kbabel, and gtranslator.

## Emacs PO mode

Emacs should automatically enter po-mode when you load a .po file, as
indicated by ``PO'' in the modeline at the bottom. The window is made
read-only, so that you can edit the .po file only through special commands. A
description of Emacs po-mode can be found under the gettext::Basics info node,
or type `h' or `?' in a po-mode window for a list of available commands. While
I find Emacs po-mode quite restrictive, this is probably due to unfamiliarity
with it. Its main advantage is that it imposes rigid conformance to the PO
file format, and checks the file format when closing the .po file
buffer. Emacs po-mode is not useful for Oriya translation, as I know of no way
to directly enter Oriya text under Emacs.

## poedit

XXX: in preparation.

## KDE: the kbabel editor

kbabel [4] is a more user-friendly and configurable editor than
either of Emacs po-mode or poedit. It is integrated into KDE, and offers
extensive contextual help. Besides support for various PO file features, it
has a plugin framework for dictionaries, that allows consistency checks and
translation suggestions.

## GNOME: the gtranslator editor

XXX: in preparation.

## Bibliography

:   1: G. Mohanty, A practical primer for using Oriya under Linux, v0.3, http://oriya.sarovar.org/docs/getting\_started/index.html, 2004, Sec. 6.2 describes the xkb layouts for Oriya. 2: G. Mohanty, A plan for Oriya localization, v0.1, http://oriya.sarovar.org/docs/translation\_plan/index.html, 2004. 3: Linux Journal article on internationalization, http://www.linuxjournal.com/article.php?sid=3023. 4: Features of the kbabel editor, http://i18n.kde.org/tools/kbabel/features.html.

# About this document ...

**A tutorial on Native Language Support using GNU gettext**

This document was generated using the
**LaTeX**2HTML translator Version 2002-2-1 (1.70)

Copyright © 1993, 1994, 1995, 1996,
Nikos Drakos,
Computer Based Learning Unit, University of Leeds.
  
Copyright © 1997, 1998, 1999,
Ross Moore,
Mathematics Department, Macquarie University, Sydney.

The command line arguments were:   
**latex2html** -no\_math -html\_version 4.0,math,unicode,i18n,tables -split 0 memo

The translation was initiated by Gora Mohanty on 2004-07-24

---


Gora Mohanty
2004-07-24
