# Supplementary material for: rDock: A Fast, Versatile and Open Source Program for Docking Ligands to Proteins and Nucleic Acids
Source: PLoS Comput Biol. 2014 Apr 10;10(4):e1003571. doi: 10.1371/journal.pcbi.1003571 (PMC3983074; doi:10.1371/journal.pcbi.1003571)
Supplement: Software S1 — Compressed file with the source code of the rDock software for ligand docking to Proteins and Nucleic Acids. (GZ) [file pcbi.1003571.s010.gz › rDock_2013.1_src/gettext-0.18.tar.gz!/gettext-0.18/gettext-tools/doc/gettext_4.html]

GNU gettext utilities - 4 Preparing Program Sources

Go to the first, previous, next, last section, table of contents.

---

# 4 Preparing Program Sources

For the programmer, changes to the C source code fall into three
categories. First, you have to make the localization functions
known to all modules needing message translation. Second, you should
properly trigger the operation of GNU `gettext` when the program
initializes, usually from the `main` function. Last, you should
identify, adjust and mark all constant strings in your program
needing translation.

## 4.1 Importing the `gettext` declaration

Presuming that your set of programs, or package, has been adjusted
so all needed GNU `gettext` files are available, and your
‘Makefile’ files are adjusted (see section 13 The Maintainer's View), each C module
having translated C strings should contain the line:

```
#include <libintl.h>
```

Similarly, each C module containing `printf()`/`fprintf()`/...
calls with a format string that could be a translated C string (even if
the C string comes from a different C module) should contain the line:

```
#include <libintl.h>
```

## 4.2 Triggering `gettext` Operations

The initialization of locale data should be done with more or less
the same code in every program, as demonstrated below:

```
int
main (int argc, char *argv[])
{
  ...
  setlocale (LC_ALL, "");
  bindtextdomain (PACKAGE, LOCALEDIR);
  textdomain (PACKAGE);
  ...
}
```

PACKAGE and LOCALEDIR should be provided either by
‘config.h’ or by the Makefile. For now consult the `gettext`
or `hello` sources for more information.

The use of `LC_ALL` might not be appropriate for you.
`LC_ALL` includes all locale categories and especially
`LC_CTYPE`. This latter category is responsible for determining
character classes with the `isalnum` etc. functions from
‘ctype.h’ which could especially for programs, which process some
kind of input language, be wrong. For example this would mean that a
source code using the ç (c-cedilla character) is runnable in
France but not in the U.S.

Some systems also have problems with parsing numbers using the
`scanf` functions if an other but the `LC_ALL` locale category is
used. The standards say that additional formats but the one known in the
`"C"` locale might be recognized. But some systems seem to reject
numbers in the `"C"` locale format. In some situation, it might
also be a problem with the notation itself which makes it impossible to
recognize whether the number is in the `"C"` locale or the local
format. This can happen if thousands separator characters are used.
Some locales define this character according to the national
conventions to `'.'` which is the same character used in the
`"C"` locale to denote the decimal point.

So it is sometimes necessary to replace the `LC_ALL` line in the
code above by a sequence of `setlocale` lines

```
{
  ...
  setlocale (LC_CTYPE, "");
  setlocale (LC_MESSAGES, "");
  ...
}
```

On all POSIX conformant systems the locale categories `LC_CTYPE`,
`LC_MESSAGES`, `LC_COLLATE`, `LC_MONETARY`,
`LC_NUMERIC`, and `LC_TIME` are available. On some systems
which are only ISO C compliant, `LC_MESSAGES` is missing, but
a substitute for it is defined in GNU gettext's `<libintl.h>` and
in GNU gnulib's `<locale.h>`.

Note that changing the `LC_CTYPE` also affects the functions
declared in the `<ctype.h>` standard header and some functions
declared in the `<string.h>` and `<stdlib.h>` standard headers.
If this is not
desirable in your application (for example in a compiler's parser),
you can use a set of substitute functions which hardwire the C locale,
such as found in the modules `‘c-ctype’`, `‘c-strcase’`,
`‘c-strcasestr’`, `‘c-strtod’`, `‘c-strtold’` in the GNU gnulib
source distribution.

It is also possible to switch the locale forth and back between the
environment dependent locale and the C locale, but this approach is
normally avoided because a `setlocale` call is expensive,
because it is tedious to determine the places where a locale switch
is needed in a large program's source, and because switching a locale
is not multithread-safe.

## 4.3 Preparing Translatable Strings

Before strings can be marked for translations, they sometimes need to
be adjusted. Usually preparing a string for translation is done right
before marking it, during the marking phase which is described in the
next sections. What you have to keep in mind while doing that is the
following.

- Decent English style.- Entire sentences.- Split at paragraphs.- Use format strings instead of string concatenation.- Avoid unusual markup and unusual control characters.

Let's look at some examples of these guidelines.

Translatable strings should be in good English style. If slang language
with abbreviations and shortcuts is used, often translators will not
understand the message and will produce very inappropriate translations.

```
"%s: is parameter\n"
```

This is nearly untranslatable: Is the displayed item *a* parameter or
*the* parameter?

```
"No match"
```

The ambiguity in this message makes it unintelligible: Is the program
attempting to set something on fire? Does it mean "The given object does
not match the template"? Does it mean "The template does not fit for any
of the objects"?

In both cases, adding more words to the message will help both the
translator and the English speaking user.

Translatable strings should be entire sentences. It is often not possible
to translate single verbs or adjectives in a substitutable way.

```
printf ("File %s is %s protected", filename, rw ? "write" : "read");
```

Most translators will not look at the source and will thus only see the
string `"File %s is %s protected"`, which is unintelligible. Change
this to

```
printf (rw ? "File %s is write protected" : "File %s is read protected",
        filename);
```

This way the translator will not only understand the message, she will
also be able to find the appropriate grammatical construction. A French
translator for example translates "write protected" like "protected
against writing".

Entire sentences are also important because in many languages, the
declination of some word in a sentence depends on the gender or the
number (singular/plural) of another part of the sentence. There are
usually more interdependencies between words than in English. The
consequence is that asking a translator to translate two half-sentences
and then combining these two half-sentences through dumb string concatenation
will not work, for many languages, even though it would work for English.
That's why translators need to handle entire sentences.

Often sentences don't fit into a single line. If a sentence is output
using two subsequent `printf` statements, like this

```
printf ("Locale charset \"%s\" is different from\n", lcharset);
printf ("input file charset \"%s\".\n", fcharset);
```

the translator would have to translate two half sentences, but nothing
in the POT file would tell her that the two half sentences belong together.
It is necessary to merge the two `printf` statements so that the
translator can handle the entire sentence at once and decide at which
place to insert a line break in the translation (if at all):

```
printf ("Locale charset \"%s\" is different from\n\
input file charset \"%s\".\n", lcharset, fcharset);
```

You may now ask: how about two or more adjacent sentences? Like in this case:

```
puts ("Apollo 13 scenario: Stack overflow handling failed.");
puts ("On the next stack overflow we will crash!!!");
```

Should these two statements merged into a single one? I would recommend to
merge them if the two sentences are related to each other, because then it
makes it easier for the translator to understand and translate both. On
the other hand, if one of the two messages is a stereotypic one, occurring
in other places as well, you will do a favour to the translator by not
merging the two. (Identical messages occurring in several places are
combined by xgettext, so the translator has to handle them once only.)

Translatable strings should be limited to one paragraph; don't let a
single message be longer than ten lines. The reason is that when the
translatable string changes, the translator is faced with the task of
updating the entire translated string. Maybe only a single word will
have changed in the English string, but the translator doesn't see that
(with the current translation tools), therefore she has to proofread
the entire message.

Many GNU programs have a `‘--help’` output that extends over several
screen pages. It is a courtesy towards the translators to split such a
message into several ones of five to ten lines each. While doing that,
you can also attempt to split the documented options into groups,
such as the input options, the output options, and the informative
output options. This will help every user to find the option he is
looking for.

Hardcoded string concatenation is sometimes used to construct English
strings:

```
strcpy (s, "Replace ");
strcat (s, object1);
strcat (s, " with ");
strcat (s, object2);
strcat (s, "?");
```

In order to present to the translator only entire sentences, and also
because in some languages the translator might want to swap the order
of `object1` and `object2`, it is necessary to change this
to use a format string:

```
sprintf (s, "Replace %s with %s?", object1, object2);
```

A similar case is compile time concatenation of strings. The ISO C 99
include file `<inttypes.h>` contains a macro `PRId64` that
can be used as a formatting directive for outputting an `‘int64_t’`
integer through `printf`. It expands to a constant string, usually
"d" or "ld" or "lld" or something like this, depending on the platform.
Assume you have code like

```
printf ("The amount is %0" PRId64 "\n", number);
```

The `gettext` tools and library have special support for these
`<inttypes.h>` macros. You can therefore simply write

```
printf (gettext ("The amount is %0" PRId64 "\n"), number);
```

The PO file will contain the string "The amount is %0<PRId64>\n".
The translators will provide a translation containing "%0<PRId64>"
as well, and at runtime the `gettext` function's result will
contain the appropriate constant string, "d" or "ld" or "lld".

This works only for the predefined `<inttypes.h>` macros. If
you have defined your own similar macros, let's say `‘MYPRId64’`,
that are not known to `xgettext`, the solution for this problem
is to change the code like this:

```
char buf1[100];
sprintf (buf1, "%0" MYPRId64, number);
printf (gettext ("The amount is %s\n"), buf1);
```

This means, you put the platform dependent code in one statement, and the
internationalization code in a different statement. Note that a buffer length
of 100 is safe, because all available hardware integer types are limited to
128 bits, and to print a 128 bit integer one needs at most 54 characters,
regardless whether in decimal, octal or hexadecimal.

All this applies to other programming languages as well. For example, in
Java and C#, string concatenation is very frequently used, because it is a
compiler built-in operator. Like in C, in Java, you would change

```
System.out.println("Replace "+object1+" with "+object2+"?");
```

into a statement involving a format string:

```
System.out.println(
    MessageFormat.format("Replace {0} with {1}?",
                         new Object[] { object1, object2 }));
```

Similarly, in C#, you would change

```
Console.WriteLine("Replace "+object1+" with "+object2+"?");
```

into a statement involving a format string:

```
Console.WriteLine(
    String.Format("Replace {0} with {1}?", object1, object2));
```

Unusual markup or control characters should not be used in translatable
strings. Translators will likely not understand the particular meaning
of the markup or control characters.

For example, if you have a convention that `‘|’` delimits the
left-hand and right-hand part of some GUI elements, translators will
often not understand it without specific comments. It might be
better to have the translator translate the left-hand and right-hand
part separately.

Another example is the `‘argp’` convention to use a single `‘\v’`
(vertical tab) control character to delimit two sections inside a
string. This is flawed. Some translators may convert it to a simple
newline, some to blank lines. With some PO file editors it may not be
easy to even enter a vertical tab control character. So, you cannot
be sure that the translation will contain a `‘\v’` character, at the
corresponding position. The solution is, again, to let the translator
translate two separate strings and combine at run-time the two translated
strings with the `‘\v’` required by the convention.

HTML markup, however, is common enough that it's probably ok to use in
translatable strings. But please bear in mind that the GNU gettext tools
don't verify that the translations are well-formed HTML.

## 4.4 How Marks Appear in Sources

All strings requiring translation should be marked in the C sources. Marking
is done in such a way that each translatable string appears to be
the sole argument of some function or preprocessor macro. There are
only a few such possible functions or macros meant for translation,
and their names are said to be marking keywords. The marking is
attached to strings themselves, rather than to what we do with them.
This approach has more uses. A blatant example is an error message
produced by formatting. The format string needs translation, as
well as some strings inserted through some `‘%s’` specification
in the format, while the result from `sprintf` may have so many
different instances that it is impractical to list them all in some
`‘error_string_out()’` routine, say.

This marking operation has two goals. The first goal of marking
is for triggering the retrieval of the translation, at run time.
The keyword is possibly resolved into a routine able to dynamically
return the proper translation, as far as possible or wanted, for the
argument string. Most localizable strings are found in executable
positions, that is, attached to variables or given as parameters to
functions. But this is not universal usage, and some translatable
strings appear in structured initializations. See section 4.7 Special Cases of Translatable Strings.

The second goal of the marking operation is to help `xgettext`
at properly extracting all translatable strings when it scans a set
of program sources and produces PO file templates.

The canonical keyword for marking translatable strings is
`‘gettext’`, it gave its name to the whole GNU `gettext`
package. For packages making only light use of the `‘gettext’`
keyword, macro or function, it is easily used *as is*. However,
for packages using the `gettext` interface more heavily, it
is usually more convenient to give the main keyword a shorter, less
obtrusive name. Indeed, the keyword might appear on a lot of strings
all over the package, and programmers usually do not want nor need
their program sources to remind them forcefully, all the time, that they
are internationalized. Further, a long keyword has the disadvantage
of using more horizontal space, forcing more indentation work on
sources for those trying to keep them within 79 or 80 columns.

Many packages use `‘_’` (a simple underline) as a keyword,
and write `‘_("Translatable string")’` instead of `‘gettext
("Translatable string")’`. Further, the coding rule, from GNU standards,
wanting that there is a space between the keyword and the opening
parenthesis is relaxed, in practice, for this particular usage.
So, the textual overhead per translatable string is reduced to
only three characters: the underline and the two parentheses.
However, even if GNU `gettext` uses this convention internally,
it does not offer it officially. The real, genuine keyword is truly
`‘gettext’` indeed. It is fairly easy for those wanting to use
`‘_’` instead of `‘gettext’` to declare:

```
#include <libintl.h>
#define _(String) gettext (String)
```

instead of merely using `‘#include <libintl.h>’`.

The marking keywords `‘gettext’` and `‘_’` take the translatable
string as sole argument. It is also possible to define marking functions
that take it at another argument position. It is even possible to make
the marked argument position depend on the total number of arguments of
the function call; this is useful in C++. All this is achieved using
`xgettext`'s `‘--keyword’` option. How to pass such an option
to `xgettext`, assuming that `gettextize` is used, is described
in section 13.4.3 ‘Makevars’ in ‘po/’ and section 13.5.6 AM\_XGETTEXT\_OPTION in ‘po.m4’.

Note also that long strings can be split across lines, into multiple
adjacent string tokens. Automatic string concatenation is performed
at compile time according to ISO C and ISO C++; `xgettext` also
supports this syntax.

Later on, the maintenance is relatively easy. If, as a programmer,
you add or modify a string, you will have to ask yourself if the
new or altered string requires translation, and include it within
`‘_()’` if you think it should be translated. For example, `‘"%s"’`
is an example of string *not* requiring translation. But
`‘"%s: %d"’` *does* require translation, because in French, unlike
in English, it's customary to put a space before a colon.

## 4.5 Marking Translatable Strings

In PO mode, one set of features is meant more for the programmer than
for the translator, and allows him to interactively mark which strings,
in a set of program sources, are translatable, and which are not.
Even if it is a fairly easy job for a programmer to find and mark
such strings by other means, using any editor of his choice, PO mode
makes this work more comfortable. Further, this gives translators
who feel a little like programmers, or programmers who feel a little
like translators, a tool letting them work at marking translatable
strings in the program sources, while simultaneously producing a set of
translation in some language, for the package being internationalized.

The set of program sources, targeted by the PO mode commands describe
here, should have an Emacs tags table constructed for your project,
prior to using these PO file commands. This is easy to do. In any
shell window, change the directory to the root of your project, then
execute a command resembling:

```
etags src/*.[hc] lib/*.[hc]
```

presuming here you want to process all ‘.h’ and ‘.c’ files
from the ‘src/’ and ‘lib/’ directories. This command will
explore all said files and create a ‘TAGS’ file in your root
directory, somewhat summarizing the contents using a special file
format Emacs can understand.

For packages following the GNU coding standards, there is
a make goal `tags` or `TAGS` which constructs the tag files in
all directories and for all files containing source code.

Once your ‘TAGS’ file is ready, the following commands assist
the programmer at marking translatable strings in his set of sources.
But these commands are necessarily driven from within a PO file
window, and it is likely that you do not even have such a PO file yet.
This is not a problem at all, as you may safely open a new, empty PO
file, mainly for using these commands. This empty PO file will slowly
fill in while you mark strings as translatable in your program sources.

`,`: Search through program sources for a string which looks like a candidate for translation (`po-tags-search`). `M-,`: Mark the last string found with `‘_()’` (`po-mark-translatable`). `M-.`: Mark the last string found with a keyword taken from a set of possible keywords. This command with a prefix allows some management of these keywords (`po-select-mark-and-mark`).

The `,` (`po-tags-search`) command searches for the next
occurrence of a string which looks like a possible candidate for
translation, and displays the program source in another Emacs window,
positioned in such a way that the string is near the top of this other
window. If the string is too big to fit whole in this window, it is
positioned so only its end is shown. In any case, the cursor
is left in the PO file window. If the shown string would be better
presented differently in different native languages, you may mark it
using `M-,` or `M-.`. Otherwise, you might rather ignore it
and skip to the next string by merely repeating the `,` command.

A string is a good candidate for translation if it contains a sequence
of three or more letters. A string containing at most two letters in
a row will be considered as a candidate if it has more letters than
non-letters. The command disregards strings containing no letters,
or isolated letters only. It also disregards strings within comments,
or strings already marked with some keyword PO mode knows (see below).

If you have never told Emacs about some ‘TAGS’ file to use, the
command will request that you specify one from the minibuffer, the
first time you use the command. You may later change your ‘TAGS’
file by using the regular Emacs command `M-x visit-tags-table`,
which will ask you to name the precise ‘TAGS’ file you want
to use. See section ‘Tag Tables’ in The Emacs Editor.

Each time you use the `,` command, the search resumes from where it was
left by the previous search, and goes through all program sources,
obeying the ‘TAGS’ file, until all sources have been processed.
However, by giving a prefix argument to the command (`C-u
,)`, you may request that the search be restarted all over again
from the first program source; but in this case, strings that you
recently marked as translatable will be automatically skipped.

Using this `,` command does not prevent using of other regular
Emacs tags commands. For example, regular `tags-search` or
`tags-query-replace` commands may be used without disrupting the
independent `,` search sequence. However, as implemented, the
*initial* `,` command (or the `,` command is used with a
prefix) might also reinitialize the regular Emacs tags searching to the
first tags file, this reinitialization might be considered spurious.

The `M-,` (`po-mark-translatable`) command will mark the
recently found string with the `‘_’` keyword. The `M-.`
(`po-select-mark-and-mark`) command will request that you type
one keyword from the minibuffer and use that keyword for marking
the string. Both commands will automatically create a new PO file
untranslated entry for the string being marked, and make it the
current entry (making it easy for you to immediately proceed to its
translation, if you feel like doing it right away). It is possible
that the modifications made to the program source by `M-,` or
`M-.` render some source line longer than 80 columns, forcing you
to break and re-indent this line differently. You may use the `O`
command from PO mode, or any other window changing command from
Emacs, to break out into the program source window, and do any
needed adjustments. You will have to use some regular Emacs command
to return the cursor to the PO file window, if you want command
`,` for the next string, say.

The `M-.` command has a few built-in speedups, so you do not
have to explicitly type all keywords all the time. The first such
speedup is that you are presented with a *preferred* keyword,
which you may accept by merely typing `RET` at the prompt.
The second speedup is that you may type any non-ambiguous prefix of the
keyword you really mean, and the command will complete it automatically
for you. This also means that PO mode has to *know* all
your possible keywords, and that it will not accept mistyped keywords.

If you reply `?` to the keyword request, the command gives a
list of all known keywords, from which you may choose. When the
command is prefixed by an argument (`C-u M-.`), it inhibits
updating any program source or PO file buffer, and does some simple
keyword management instead. In this case, the command asks for a
keyword, written in full, which becomes a new allowed keyword for
later `M-.` commands. Moreover, this new keyword automatically
becomes the *preferred* keyword for later commands. By typing
an already known keyword in response to `C-u M-.`, one merely
changes the *preferred* keyword and does nothing more.

All keywords known for `M-.` are recognized by the `,` command
when scanning for strings, and strings already marked by any of those
known keywords are automatically skipped. If many PO files are opened
simultaneously, each one has its own independent set of known keywords.
There is no provision in PO mode, currently, for deleting a known
keyword, you have to quit the file (maybe using `q`) and reopen
it afresh. When a PO file is newly brought up in an Emacs window, only
`‘gettext’` and `‘_’` are known as keywords, and `‘gettext’`
is preferred for the `M-.` command. In fact, this is not useful to
prefer `‘_’`, as this one is already built in the `M-,` command.

## 4.6 Special Comments preceding Keywords

In C programs strings are often used within calls of functions from the
`printf` family. The special thing about these format strings is
that they can contain format specifiers introduced with `%`. Assume
we have the code

```
printf (gettext ("String `%s' has %d characters\n"), s, strlen (s));
```

A possible German translation for the above string might be:

```
"%d Zeichen lang ist die Zeichenkette `%s'"
```

A C programmer, even if he cannot speak German, will recognize that
there is something wrong here. The order of the two format specifiers
is changed but of course the arguments in the `printf` don't have.
This will most probably lead to problems because now the length of the
string is regarded as the address.

To prevent errors at runtime caused by translations the `msgfmt`
tool can check statically whether the arguments in the original and the
translation string match in type and number. If this is not the case
and the `‘-c’` option has been passed to `msgfmt`, `msgfmt`
will give an error and refuse to produce a MO file. Thus consequent
use of `‘msgfmt -c’` will catch the error, so that it cannot cause
cause problems at runtime.

If the word order in the above German translation would be correct one
would have to write

```
"%2$d Zeichen lang ist die Zeichenkette `%1$s'"
```

The routines in `msgfmt` know about this special notation.

Because not all strings in a program must be format strings it is not
useful for `msgfmt` to test all the strings in the ‘.po’ file.
This might cause problems because the string might contain what looks
like a format specifier, but the string is not used in `printf`.

Therefore the `xgettext` adds a special tag to those messages it
thinks might be a format string. There is no absolute rule for this,
only a heuristic. In the ‘.po’ file the entry is marked using the
`c-format` flag in the `#,` comment line (see section 3 The Format of PO Files).

The careful reader now might say that this again can cause problems.
The heuristic might guess it wrong. This is true and therefore
`xgettext` knows about a special kind of comment which lets
the programmer take over the decision. If in the same line as or
the immediately preceding line to the `gettext` keyword
the `xgettext` program finds a comment containing the words
`xgettext:c-format`, it will mark the string in any case with
the `c-format` flag. This kind of comment should be used when
`xgettext` does not recognize the string as a format string but
it really is one and it should be tested. Please note that when the
comment is in the same line as the `gettext` keyword, it must be
before the string to be translated.

This situation happens quite often. The `printf` function is often
called with strings which do not contain a format specifier. Of course
one would normally use `fputs` but it does happen. In this case
`xgettext` does not recognize this as a format string but what
happens if the translation introduces a valid format specifier? The
`printf` function will try to access one of the parameters but none
exists because the original code does not pass any parameters.

`xgettext` of course could make a wrong decision the other way
round, i.e. a string marked as a format string actually is not a format
string. In this case the `msgfmt` might give too many warnings and
would prevent translating the ‘.po’ file. The method to prevent
this wrong decision is similar to the one used above, only the comment
to use must contain the string `xgettext:no-c-format`.

If a string is marked with `c-format` and this is not correct the
user can find out who is responsible for the decision. See
section 5.1 Invoking the `xgettext` Program to see how the `--debug` option can be
used for solving this problem.

## 4.7 Special Cases of Translatable Strings

The attentive reader might now point out that it is not always possible
to mark translatable string with `gettext` or something like this.
Consider the following case:

```
{
  static const char *messages[] = {
    "some very meaningful message",
    "and another one"
  };
  const char *string;
  ...
  string
    = index > 1 ? "a default message" : messages[index];

  fputs (string);
  ...
}
```

While it is no problem to mark the string `"a default message"` it
is not possible to mark the string initializers for `messages`.
What is to be done? We have to fulfill two tasks. First we have to mark the
strings so that the `xgettext` program (see section 5.1 Invoking the `xgettext` Program)
can find them, and second we have to translate the string at runtime
before printing them.

The first task can be fulfilled by creating a new keyword, which names a
no-op. For the second we have to mark all access points to a string
from the array. So one solution can look like this:

```
#define gettext_noop(String) String

{
  static const char *messages[] = {
    gettext_noop ("some very meaningful message"),
    gettext_noop ("and another one")
  };
  const char *string;
  ...
  string
    = index > 1 ? gettext ("a default message") : gettext (messages[index]);

  fputs (string);
  ...
}
```

Please convince yourself that the string which is written by
`fputs` is translated in any case. How to get `xgettext` know
the additional keyword `gettext_noop` is explained in section 5.1 Invoking the `xgettext` Program.

The above is of course not the only solution. You could also come along
with the following one:

```
#define gettext_noop(String) String

{
  static const char *messages[] = {
    gettext_noop ("some very meaningful message",
    gettext_noop ("and another one")
  };
  const char *string;
  ...
  string
    = index > 1 ? gettext_noop ("a default message") : messages[index];

  fputs (gettext (string));
  ...
}
```

But this has a drawback. The programmer has to take care that
he uses `gettext_noop` for the string `"a default message"`.
A use of `gettext` could have in rare cases unpredictable results.

One advantage is that you need not make control flow analysis to make
sure the output is really translated in any case. But this analysis is
generally not very difficult. If it should be in any situation you can
use this second method in this situation.

## 4.8 Letting Users Report Translation Bugs

Code sometimes has bugs, but translations sometimes have bugs too. The
users need to be able to report them. Reporting translation bugs to the
programmer or maintainer of a package is not very useful, since the
maintainer must never change a translation, except on behalf of the
translator. Hence the translation bugs must be reported to the
translators.

Here is a way to organize this so that the maintainer does not need to
forward translation bug reports, nor even keep a list of the addresses of
the translators or their translation teams.

Every program has a place where is shows the bug report address. For
GNU programs, it is the code which handles the “--help” option,
typically in a function called “usage”. In this place, instruct the
translator to add her own bug reporting address. For example, if that
code has a statement

```
printf (_("Report bugs to <%s>.\n"), PACKAGE_BUGREPORT);
```

you can add some translator instructions like this:

```
/* TRANSLATORS: The placeholder indicates the bug-reporting address
   for this package.  Please add _another line_ saying
   "Report translation bugs to <...>\n" with the address for translation
   bugs (typically your translation team's web or email address).  */
printf (_("Report bugs to <%s>.\n"), PACKAGE_BUGREPORT);
```

These will be extracted by `‘xgettext’`, leading to a .pot file that
contains this:

```
#. TRANSLATORS: The placeholder indicates the bug-reporting address
#. for this package.  Please add _another line_ saying
#. "Report translation bugs to <...>\n" with the address for translation
#. bugs (typically your translation team's web or email address).
#: src/hello.c:178
#, c-format
msgid "Report bugs to <%s>.\n"
msgstr ""
```

## 4.9 Marking Proper Names for Translation

Should names of persons, cities, locations etc. be marked for translation
or not? People who only know languages that can be written with Latin
letters (English, Spanish, French, German, etc.) are tempted to say “no”,
because names usually do not change when transported between these languages.
However, in general when translating from one script to another, names
are translated too, usually phonetically or by transliteration. For
example, Russian or Greek names are converted to the Latin alphabet when
being translated to English, and English or French names are converted
to the Katakana script when being translated to Japanese. This is
necessary because the speakers of the target language in general cannot
read the script the name is originally written in.

As a programmer, you should therefore make sure that names are marked
for translation, with a special comment telling the translators that it
is a proper name and how to pronounce it. In its simple form, it looks
like this:

```
printf (_("Written by %s.\n"),
        /* TRANSLATORS: This is a proper name.  See the gettext
           manual, section Names.  Note this is actually a non-ASCII
           name: The first name is (with Unicode escapes)
           "Fran\u00e7ois" or (with HTML entities) "Fran&ccedil;ois".
           Pronunciation is like "fraa-swa pee-nar".  */
        _("Francois Pinard"));
```

The GNU gnulib library offers a module `‘propername’`
(http://www.gnu.org/software/gnulib/MODULES.html#module=propername)
which takes care to automatically append the original name, in parentheses,
to the translated name. For names that cannot be written in ASCII, it
also frees the translator from the task of entering the appropriate non-ASCII
characters if no script change is needed. In this more comfortable form,
it looks like this:

```
printf (_("Written by %s and %s.\n"),
        proper_name ("Ulrich Drepper"),
        /* TRANSLATORS: This is a proper name.  See the gettext
           manual, section Names.  Note this is actually a non-ASCII
           name: The first name is (with Unicode escapes)
           "Fran\u00e7ois" or (with HTML entities) "Fran&ccedil;ois".
           Pronunciation is like "fraa-swa pee-nar".  */
        proper_name_utf8 ("Francois Pinard", "Fran\303\247ois Pinard"));
```

You can also write the original name directly in Unicode (rather than with
Unicode escapes or HTML entities) and denote the pronunciation using the
International Phonetic Alphabet (see
http://www.wikipedia.org/wiki/International\_Phonetic\_Alphabet).

As a translator, you should use some care when translating names, because
it is frustrating if people see their names mutilated or distorted.

If your language uses the Latin script, all you need to do is to reproduce
the name as perfectly as you can within the usual character set of your
language. In this particular case, this means to provide a translation
containing the c-cedilla character. If your language uses a different
script and the people speaking it don't usually read Latin words, it means
transliteration. If the programmer used the simple case, you should still
give, in parentheses, the original writing of the name -- for the sake of
the people that do read the Latin script. If the programmer used the
`‘propername’` module mentioned above, you don't need to give the original
writing of the name in parentheses, because the program will already do so.
Here is an example, using Greek as the target script:

```
#. This is a proper name.  See the gettext
#. manual, section Names.  Note this is actually a non-ASCII
#. name: The first name is (with Unicode escapes)
#. "Fran\u00e7ois" or (with HTML entities) "Fran&ccedil;ois".
#. Pronunciation is like "fraa-swa pee-nar".
msgid "Francois Pinard"
msgstr "\phi\rho\alpha\sigma\omicron\alpha \pi\iota\nu\alpha\rho"
       " (Francois Pinard)"
```

Because translation of names is such a sensitive domain, it is a good
idea to test your translation before submitting it.

## 4.10 Preparing Library Sources

When you are preparing a library, not a program, for the use of
`gettext`, only a few details are different. Here we assume that
the library has a translation domain and a POT file of its own. (If
it uses the translation domain and POT file of the main program, then
the previous sections apply without changes.)

1. The library code doesn't call `setlocale (LC_ALL, "")`. It's the
   responsibility of the main program to set the locale. The library's
   documentation should mention this fact, so that developers of programs
   using the library are aware of it.- The library code doesn't call `textdomain (PACKAGE)`, because it
     would interfere with the text domain set by the main program.- The initialization code for a program was

       ```
         setlocale (LC_ALL, "");
         bindtextdomain (PACKAGE, LOCALEDIR);
         textdomain (PACKAGE);
       ```

       For a library it is reduced to

       ```
         bindtextdomain (PACKAGE, LOCALEDIR);
       ```

       If your library's API doesn't already have an initialization function,
       you need to create one, containing at least the `bindtextdomain`
       invocation. However, you usually don't need to export and document this
       initialization function: It is sufficient that all entry points of the
       library call the initialization function if it hasn't been called before.
       The typical idiom used to achieve this is a static boolean variable that
       indicates whether the initialization function has been called. Like this:

       ```
       static bool libfoo_initialized;

       static void
       libfoo_initialize (void)
       {
         bindtextdomain (PACKAGE, LOCALEDIR);
         libfoo_initialized = true;
       }

       /* This function is part of the exported API.  */
       struct foo *
       create_foo (...)
       {
         /* Must ensure the initialization is performed.  */
         if (!libfoo_initialized)
           libfoo_initialize ();
         ...
       }

       /* This function is part of the exported API.  The argument must be
          non-NULL and have been created through create_foo().  */
       int
       foo_refcount (struct foo *argument)
       {
         /* No need to invoke the initialization function here, because
            create_foo() must already have been called before.  */
         ...
       }
       ```

       - The usual declaration of the `‘_’` macro in each source file was

         ```
         #include <libintl.h>
         #define _(String) gettext (String)
         ```

         for a program. For a library, which has its own translation domain,
         it reads like this:

         ```
         #include <libintl.h>
         #define _(String) dgettext (PACKAGE, String)
         ```

         In other words, `dgettext` is used instead of `gettext`.
         Similarly, the `dngettext` function should be used in place of the
         `ngettext` function.

---

Go to the first, previous, next, last section, table of contents.
