# Supplementary material for: rDock: A Fast, Versatile and Open Source Program for Docking Ligands to Proteins and Nucleic Acids
Source: PLoS Comput Biol. 2014 Apr 10;10(4):e1003571. doi: 10.1371/journal.pcbi.1003571 (PMC3983074; doi:10.1371/journal.pcbi.1003571)
Supplement: Software S1 — Compressed file with the source code of the rDock software for ligand docking to Proteins and Nucleic Acids. (GZ) [file pcbi.1003571.s010.gz › rDock_2013.1_src/gettext-0.18.tar.gz!/gettext-0.18/gettext-tools/doc/gettext_6.html]

GNU gettext utilities - 6 Creating a New PO File

Go to the first, previous, next, last section, table of contents.

---

# 6 Creating a New PO File

When starting a new translation, the translator creates a file called
‘LANG.po’, as a copy of the ‘package.pot’ template
file with modifications in the initial comments (at the beginning of the file)
and in the header entry (the first entry, near the beginning of the file).

The easiest way to do so is by use of the `‘msginit’` program.
For example:

```
$ cd PACKAGE-VERSION
$ cd po
$ msginit
```

The alternative way is to do the copy and modifications by hand.
To do so, the translator copies ‘package.pot’ to
‘LANG.po’. Then she modifies the initial comments and
the header entry of this file.

## 6.1 Invoking the `msginit` Program

```
msginit [option]
```

The `msginit` program creates a new PO file, initializing the meta
information with values from the user's environment.

### 6.1.1 Input file location

`‘-i inputfile’`: `‘--input=inputfile’`: Input POT file.

If no inputfile is given, the current directory is searched for the
POT file. If it is `‘-’`, standard input is read.

### 6.1.2 Output file location

`‘-o file’`: `‘--output-file=file’`: Write output to specified PO file.

If no output file is given, it depends on the `‘--locale’` option or the
user's locale setting. If it is `‘-’`, the results are written to
standard output.

### 6.1.3 Input file syntax

`‘-P’`: `‘--properties-input’`: Assume the input file is a Java ResourceBundle in Java `.properties` syntax, not in PO file syntax. `‘--stringtable-input’`: Assume the input file is a NeXTstep/GNUstep localized resource file in `.strings` syntax, not in PO file syntax.

### 6.1.4 Output details

`‘-l ll_CC’`: `‘--locale=ll_CC’`: Set target locale. ll should be a language code, and CC should be a country code. The command `‘locale -a’` can be used to output a list of all installed locales. The default is the user's locale setting. `‘--no-translator’`: Declares that the PO file will not have a human translator and is instead automatically generated. `‘--color’`: `‘--color=when’`: Specify whether or when to use colors and other text attributes. See section 9.11.1 The `--color` option for details. `‘--style=style_file’`: Specify the CSS style rule file to use for `--color`. See section 9.11.3 The `--style` option for details. `‘-p’`: `‘--properties-output’`: Write out a Java ResourceBundle in Java `.properties` syntax. Note that this file format doesn't support plural forms and silently drops obsolete messages. `‘--stringtable-output’`: Write out a NeXTstep/GNUstep localized resource file in `.strings` syntax. Note that this file format doesn't support plural forms. `‘-w number’`: `‘--width=number’`: Set the output page width. Long strings in the output files will be split across multiple lines in order to ensure that each line's width (= number of screen columns) is less or equal to the given number. `‘--no-wrap’`: Do not break long message lines. Message lines whose width exceeds the output page width will not be split into several lines. Only file reference lines which are wider than the output page width will be split.

### 6.1.5 Informative output

`‘-h’`: `‘--help’`: Display this help and exit. `‘-V’`: `‘--version’`: Output version information and exit.

## 6.2 Filling in the Header Entry

The initial comments "SOME DESCRIPTIVE TITLE", "YEAR" and
"FIRST AUTHOR <EMAIL@ADDRESS>, YEAR" ought to be replaced by sensible
information. This can be done in any text editor; if Emacs is used
and it switched to PO mode automatically (because it has recognized
the file's suffix), you can disable it by typing `M-x fundamental-mode`.

Modifying the header entry can already be done using PO mode: in Emacs,
type `M-x po-mode RET` and then `RET` again to start editing the
entry. You should fill in the following fields.

Project-Id-Version: This is the name and version of the package. Fill it in if it has not already been filled in by `xgettext`. Report-Msgid-Bugs-To: This has already been filled in by `xgettext`. It contains an email address or URL where you can report bugs in the untranslated strings: - Strings which are not entire sentences, see the maintainer guidelines in section 4.3 Preparing Translatable Strings.- Strings which use unclear terms or require additional context to be understood.- Strings which make invalid assumptions about notation of date, time or money.- Pluralisation problems.- Incorrect English spelling.- Incorrect formatting. POT-Creation-Date: This has already been filled in by `xgettext`. PO-Revision-Date: You don't need to fill this in. It will be filled by the PO file editor when you save the file. Last-Translator: Fill in your name and email address (without double quotes). Language-Team: Fill in the English name of the language, and the email address or homepage URL of the language team you are part of. Before starting a translation, it is a good idea to get in touch with your translation team, not only to make sure you don't do duplicated work, but also to coordinate difficult linguistic issues. In the Free Translation Project, each translation team has its own mailing list. The up-to-date list of teams can be found at the Free Translation Project's homepage, http://translationproject.org/, in the "Teams" area. Language: Fill in the language code of the language. This can be in one of three forms: - `‘ll’`, an ISO 639 two-letter language code (lowercase). See section A Language Codes for the list of codes.- `‘ll_CC’`, where `‘ll’` is an ISO 639 two-letter language code (lowercase) and `‘CC’` is an ISO 3166 two-letter country code (uppercase). The country code specification is not redundant: Some languages have dialects in different countries. For example, `‘de_AT’` is used for Austria, and `‘pt_BR’` for Brazil. The country code serves to distinguish the dialects. See section A Language Codes and section B Country Codes for the lists of codes.- `‘ll_CC@variant’`, where `‘ll’` is an ISO 639 two-letter language code (lowercase), `‘CC’` is an ISO 3166 two-letter country code (uppercase), and `‘variant’` is a variant designator. The variant designator (lowercase) can be a script designator, such as `‘latin’` or `‘cyrillic’`. The naming convention `‘ll_CC’` is also the way locales are named on systems based on GNU libc. But there are three important differences: - In this PO file field, but not in locale names, `‘ll_CC’` combinations denoting a language's main dialect are abbreviated as `‘ll’`. For example, `‘de’` is equivalent to `‘de_DE’` (German as spoken in Germany), and `‘pt’` to `‘pt_PT’` (Portuguese as spoken in Portugal) in this context.- In this PO file field, suffixes like `‘.encoding’` are not used.- In this PO file field, variant designators that are not relevant to message translation, such as `‘@euro’`, are not used. So, if your locale name is `‘de_DE.UTF-8’`, the language specification in PO files is just `‘de’`. Content-Type: Replace `‘CHARSET’` with the character encoding used for your language, in your locale, or UTF-8. This field is needed for correct operation of the `msgmerge` and `msgfmt` programs, as well as for users whose locale's character encoding differs from yours (see section 11.2.4 How to specify the output character set `gettext` uses). You get the character encoding of your locale by running the shell command `‘locale charmap’`. If the result is `‘C’` or `‘ANSI_X3.4-1968’`, which is equivalent to `‘ASCII’` (= `‘US-ASCII’`), it means that your locale is not correctly configured. In this case, ask your translation team which charset to use. `‘ASCII’` is not usable for any language except Latin. Because the PO files must be portable to operating systems with less advanced internationalization facilities, the character encodings that can be used are limited to those supported by both GNU `libc` and GNU `libiconv`. These are: `ASCII`, `ISO-8859-1`, `ISO-8859-2`, `ISO-8859-3`, `ISO-8859-4`, `ISO-8859-5`, `ISO-8859-6`, `ISO-8859-7`, `ISO-8859-8`, `ISO-8859-9`, `ISO-8859-13`, `ISO-8859-14`, `ISO-8859-15`, `KOI8-R`, `KOI8-U`, `KOI8-T`, `CP850`, `CP866`, `CP874`, `CP932`, `CP949`, `CP950`, `CP1250`, `CP1251`, `CP1252`, `CP1253`, `CP1254`, `CP1255`, `CP1256`, `CP1257`, `GB2312`, `EUC-JP`, `EUC-KR`, `EUC-TW`, `BIG5`, `BIG5-HKSCS`, `GBK`, `GB18030`, `SHIFT_JIS`, `JOHAB`, `TIS-620`, `VISCII`, `GEORGIAN-PS`, `UTF-8`. In the GNU system, the following encodings are frequently used for the corresponding languages. - `ISO-8859-1` for Afrikaans, Albanian, Basque, Breton, Catalan, Cornish, Danish, Dutch, English, Estonian, Faroese, Finnish, French, Galician, German, Greenlandic, Icelandic, Indonesian, Irish, Italian, Malay, Manx, Norwegian, Occitan, Portuguese, Spanish, Swedish, Tagalog, Uzbek, Walloon,- `ISO-8859-2` for Bosnian, Croatian, Czech, Hungarian, Polish, Romanian, Serbian, Slovak, Slovenian,- `ISO-8859-3` for Maltese,- `ISO-8859-5` for Macedonian, Serbian,- `ISO-8859-6` for Arabic,- `ISO-8859-7` for Greek,- `ISO-8859-8` for Hebrew,- `ISO-8859-9` for Turkish,- `ISO-8859-13` for Latvian, Lithuanian, Maori,- `ISO-8859-14` for Welsh,- `ISO-8859-15` for Basque, Catalan, Dutch, English, Finnish, French, Galician, German, Irish, Italian, Portuguese, Spanish, Swedish, Walloon,- `KOI8-R` for Russian,- `KOI8-U` for Ukrainian,- `KOI8-T` for Tajik,- `CP1251` for Bulgarian, Belarusian,- `GB2312`, `GBK`, `GB18030` for simplified writing of Chinese,- `BIG5`, `BIG5-HKSCS` for traditional writing of Chinese,- `EUC-JP` for Japanese,- `EUC-KR` for Korean,- `TIS-620` for Thai,- `GEORGIAN-PS` for Georgian,- `UTF-8` for any language, including those listed above. When single quote characters or double quote characters are used in translations for your language, and your locale's encoding is one of the ISO-8859-\* charsets, it is best if you create your PO files in UTF-8 encoding, instead of your locale's encoding. This is because in UTF-8 the real quote characters can be represented (single quote characters: U+2018, U+2019, double quote characters: U+201C, U+201D), whereas none of ISO-8859-\* charsets has them all. Users in UTF-8 locales will see the real quote characters, whereas users in ISO-8859-\* locales will see the vertical apostrophe and the vertical double quote instead (because that's what the character set conversion will transliterate them to). To enter such quote characters under X11, you can change your keyboard mapping using the `xmodmap` program. The X11 names of the quote characters are "leftsinglequotemark", "rightsinglequotemark", "leftdoublequotemark", "rightdoublequotemark", "singlelowquotemark", "doublelowquotemark". Note that only recent versions of GNU Emacs support the UTF-8 encoding: Emacs 20 with Mule-UCS, and Emacs 21. As of January 2001, XEmacs doesn't support the UTF-8 encoding. The character encoding name can be written in either upper or lower case. Usually upper case is preferred. Content-Transfer-Encoding: Set this to `8bit`. Plural-Forms: This field is optional. It is only needed if the PO file has plural forms. You can find them by searching for the `‘msgid_plural’` keyword. The format of the plural forms field is described in section 11.2.6 Additional functions for plural forms and section 12.6 Translating plural forms.

---

Go to the first, previous, next, last section, table of contents.
