# Supplementary material for: rDock: A Fast, Versatile and Open Source Program for Docking Ligands to Proteins and Nucleic Acids
Source: PLoS Comput Biol. 2014 Apr 10;10(4):e1003571. doi: 10.1371/journal.pcbi.1003571 (PMC3983074; doi:10.1371/journal.pcbi.1003571)
Supplement: Software S1 — Compressed file with the source code of the rDock software for ligand docking to Proteins and Nucleic Acids. (GZ) [file pcbi.1003571.s010.gz › rDock_2013.1_src/gettext-0.18.tar.gz!/gettext-0.18/gettext-tools/doc/gettext_18.html]

GNU gettext utilities - B Country Codes

Go to the first, previous, next, last section, table of contents.

---

# B Country Codes

The ISO 3166 standard defines two character codes for many countries
and territories. All abbreviations for countries used in the Translation
Project should come from this standard.

`‘AD’`: Andorra. `‘AE’`: United Arab Emirates. `‘AF’`: Afghanistan. `‘AG’`: Antigua and Barbuda. `‘AI’`: Anguilla. `‘AL’`: Albania. `‘AM’`: Armenia. `‘AN’`: Netherlands Antilles. `‘AO’`: Angola. `‘AQ’`: Antarctica. `‘AR’`: Argentina. `‘AS’`: Samoa (American). `‘AT’`: Austria. `‘AU’`: Australia. `‘AW’`: Aruba. `‘AX’`: Aaland Islands. `‘AZ’`: Azerbaijan. `‘BA’`: Bosnia and Herzegovina. `‘BB’`: Barbados. `‘BD’`: Bangladesh. `‘BE’`: Belgium. `‘BF’`: Burkina Faso. `‘BG’`: Bulgaria. `‘BH’`: Bahrain. `‘BI’`: Burundi. `‘BJ’`: Benin. `‘BM’`: Bermuda. `‘BN’`: Brunei. `‘BO’`: Bolivia. `‘BR’`: Brazil. `‘BS’`: Bahamas. `‘BT’`: Bhutan. `‘BV’`: Bouvet Island. `‘BW’`: Botswana. `‘BY’`: Belarus. `‘BZ’`: Belize. `‘CA’`: Canada. `‘CC’`: Cocos (Keeling) Islands. `‘CD’`: Congo (Dem. Rep.). `‘CF’`: Central African Republic. `‘CG’`: Congo (Rep.). `‘CH’`: Switzerland. `‘CI’`: Côte d'Ivoire. `‘CK’`: Cook Islands. `‘CL’`: Chile. `‘CM’`: Cameroon. `‘CN’`: China. `‘CO’`: Colombia. `‘CR’`: Costa Rica. `‘CU’`: Cuba. `‘CV’`: Cape Verde. `‘CX’`: Christmas Island. `‘CY’`: Cyprus. `‘CZ’`: Czech Republic. `‘DE’`: Germany. `‘DJ’`: Djibouti. `‘DK’`: Denmark. `‘DM’`: Dominica. `‘DO’`: Dominican Republic. `‘DZ’`: Algeria. `‘EC’`: Ecuador. `‘EE’`: Estonia. `‘EG’`: Egypt. `‘EH’`: Western Sahara. `‘ER’`: Eritrea. `‘ES’`: Spain. `‘ET’`: Ethiopia. `‘FI’`: Finland. `‘FJ’`: Fiji. `‘FK’`: Falkland Islands. `‘FM’`: Micronesia. `‘FO’`: Faeroe Islands. `‘FR’`: France. `‘GA’`: Gabon. `‘GB’`: Britain (United Kingdom). `‘GD’`: Grenada. `‘GE’`: Georgia. `‘GF’`: French Guiana. `‘GG’`: Guernsey. `‘GH’`: Ghana. `‘GI’`: Gibraltar. `‘GL’`: Greenland. `‘GM’`: Gambia. `‘GN’`: Guinea. `‘GP’`: Guadeloupe. `‘GQ’`: Equatorial Guinea. `‘GR’`: Greece. `‘GS’`: South Georgia and the South Sandwich Islands. `‘GT’`: Guatemala. `‘GU’`: Guam. `‘GW’`: Guinea-Bissau. `‘GY’`: Guyana. `‘HK’`: Hong Kong. `‘HM’`: Heard Island and McDonald Islands. `‘HN’`: Honduras. `‘HR’`: Croatia. `‘HT’`: Haiti. `‘HU’`: Hungary. `‘ID’`: Indonesia. `‘IE’`: Ireland. `‘IL’`: Israel. `‘IM’`: Isle of Man. `‘IN’`: India. `‘IO’`: British Indian Ocean Territory. `‘IQ’`: Iraq. `‘IR’`: Iran. `‘IS’`: Iceland. `‘IT’`: Italy. `‘JE’`: Jersey. `‘JM’`: Jamaica. `‘JO’`: Jordan. `‘JP’`: Japan. `‘KE’`: Kenya. `‘KG’`: Kyrgyzstan. `‘KH’`: Cambodia. `‘KI’`: Kiribati. `‘KM’`: Comoros. `‘KN’`: St Kitts and Nevis. `‘KP’`: Korea (North). `‘KR’`: Korea (South). `‘KW’`: Kuwait. `‘KY’`: Cayman Islands. `‘KZ’`: Kazakhstan. `‘LA’`: Laos. `‘LB’`: Lebanon. `‘LC’`: St Lucia. `‘LI’`: Liechtenstein. `‘LK’`: Sri Lanka. `‘LR’`: Liberia. `‘LS’`: Lesotho. `‘LT’`: Lithuania. `‘LU’`: Luxembourg. `‘LV’`: Latvia. `‘LY’`: Libya. `‘MA’`: Morocco. `‘MC’`: Monaco. `‘MD’`: Moldova. `‘ME’`: Montenegro. `‘MG’`: Madagascar. `‘MH’`: Marshall Islands. `‘MK’`: Macedonia. `‘ML’`: Mali. `‘MM’`: Myanmar (Burma). `‘MN’`: Mongolia. `‘MO’`: Macao. `‘MP’`: Northern Mariana Islands. `‘MQ’`: Martinique. `‘MR’`: Mauritania. `‘MS’`: Montserrat. `‘MT’`: Malta. `‘MU’`: Mauritius. `‘MV’`: Maldives. `‘MW’`: Malawi. `‘MX’`: Mexico. `‘MY’`: Malaysia. `‘MZ’`: Mozambique. `‘NA’`: Namibia. `‘NC’`: New Caledonia. `‘NE’`: Niger. `‘NF’`: Norfolk Island. `‘NG’`: Nigeria. `‘NI’`: Nicaragua. `‘NL’`: Netherlands. `‘NO’`: Norway. `‘NP’`: Nepal. `‘NR’`: Nauru. `‘NU’`: Niue. `‘NZ’`: New Zealand. `‘OM’`: Oman. `‘PA’`: Panama. `‘PE’`: Peru. `‘PF’`: French Polynesia. `‘PG’`: Papua New Guinea. `‘PH’`: Philippines. `‘PK’`: Pakistan. `‘PL’`: Poland. `‘PM’`: St Pierre and Miquelon. `‘PN’`: Pitcairn. `‘PR’`: Puerto Rico. `‘PS’`: Palestine. `‘PT’`: Portugal. `‘PW’`: Palau. `‘PY’`: Paraguay. `‘QA’`: Qatar. `‘RE’`: Reunion. `‘RO’`: Romania. `‘RS’`: Serbia. `‘RU’`: Russia. `‘RW’`: Rwanda. `‘SA’`: Saudi Arabia. `‘SB’`: Solomon Islands. `‘SC’`: Seychelles. `‘SD’`: Sudan. `‘SE’`: Sweden. `‘SG’`: Singapore. `‘SH’`: St Helena. `‘SI’`: Slovenia. `‘SJ’`: Svalbard and Jan Mayen. `‘SK’`: Slovakia. `‘SL’`: Sierra Leone. `‘SM’`: San Marino. `‘SN’`: Senegal. `‘SO’`: Somalia. `‘SR’`: Suriname. `‘ST’`: Sao Tome and Principe. `‘SV’`: El Salvador. `‘SY’`: Syria. `‘SZ’`: Swaziland. `‘TC’`: Turks and Caicos Islands. `‘TD’`: Chad. `‘TF’`: French Southern and Antarctic Lands. `‘TG’`: Togo. `‘TH’`: Thailand. `‘TJ’`: Tajikistan. `‘TK’`: Tokelau. `‘TL’`: Timor-Leste. `‘TM’`: Turkmenistan. `‘TN’`: Tunisia. `‘TO’`: Tonga. `‘TR’`: Turkey. `‘TT’`: Trinidad and Tobago. `‘TV’`: Tuvalu. `‘TW’`: Taiwan. `‘TZ’`: Tanzania. `‘UA’`: Ukraine. `‘UG’`: Uganda. `‘UM’`: US minor outlying islands. `‘US’`: United States. `‘UY’`: Uruguay. `‘UZ’`: Uzbekistan. `‘VA’`: Vatican City. `‘VC’`: St Vincent and the Grenadines. `‘VE’`: Venezuela. `‘VG’`: Virgin Islands (UK). `‘VI’`: Virgin Islands (US). `‘VN’`: Vietnam. `‘VU’`: Vanuatu. `‘WF’`: Wallis and Futuna. `‘WS’`: Samoa (Western). `‘YE’`: Yemen. `‘YT’`: Mayotte. `‘ZA’`: South Africa. `‘ZM’`: Zambia. `‘ZW’`: Zimbabwe.

---

Go to the first, previous, next, last section, table of contents.
