# Supplementary material for: rDock: A Fast, Versatile and Open Source Program for Docking Ligands to Proteins and Nucleic Acids
Source: PLoS Comput Biol. 2014 Apr 10;10(4):e1003571. doi: 10.1371/journal.pcbi.1003571 (PMC3983074; doi:10.1371/journal.pcbi.1003571)
Supplement: Software S1 — Compressed file with the source code of the rDock software for ligand docking to Proteins and Nucleic Acids. (GZ) [file pcbi.1003571.s010.gz › rDock_2013.1_src/gettext-0.18.tar.gz!/gettext-0.18/gettext-tools/doc/gettext_13.html]

GNU gettext utilities - 13 The Maintainer's View

Go to the first, previous, next, last section, table of contents.

---

# 13 The Maintainer's View

The maintainer of a package has many responsibilities. One of them
is ensuring that the package will install easily on many platforms,
and that the magic we described earlier (see section 2 The User's View) will work
for installers and end users.

Of course, there are many possible ways by which GNU `gettext`
might be integrated in a distribution, and this chapter does not cover
them in all generality. Instead, it details one possible approach which
is especially adequate for many free software distributions following GNU
standards, or even better, Gnits standards, because GNU `gettext`
is purposely for helping the internationalization of the whole GNU
project, and as many other good free packages as possible. So, the
maintainer's view presented here presumes that the package already has
a ‘configure.ac’ file and uses GNU Autoconf.

Nevertheless, GNU `gettext` may surely be useful for free packages
not following GNU standards and conventions, but the maintainers of such
packages might have to show imagination and initiative in organizing
their distributions so `gettext` work for them in all situations.
There are surely many, out there.

Even if `gettext` methods are now stabilizing, slight adjustments
might be needed between successive `gettext` versions, so you
should ideally revise this chapter in subsequent releases, looking
for changes.

## 13.1 Flat or Non-Flat Directory Structures

Some free software packages are distributed as `tar` files which unpack
in a single directory, these are said to be *flat* distributions.
Other free software packages have a one level hierarchy of subdirectories, using
for example a subdirectory named ‘doc/’ for the Texinfo manual and
man pages, another called ‘lib/’ for holding functions meant to
replace or complement C libraries, and a subdirectory ‘src/’ for
holding the proper sources for the package. These other distributions
are said to be *non-flat*.

We cannot say much about flat distributions. A flat
directory structure has the disadvantage of increasing the difficulty
of updating to a new version of GNU `gettext`. Also, if you have
many PO files, this could somewhat pollute your single directory.
Also, GNU `gettext`'s libintl sources consist of C sources, shell
scripts, `sed` scripts and complicated Makefile rules, which don't
fit well into an existing flat structure. For these reasons, we
recommend to use non-flat approach in this case as well.

Maybe because GNU `gettext` itself has a non-flat structure,
we have more experience with this approach, and this is what will be
described in the remaining of this chapter. Some maintainers might
use this as an opportunity to unflatten their package structure.

## 13.2 Prerequisite Works

There are some works which are required for using GNU `gettext`
in one of your package. These works have some kind of generality
that escape the point by point descriptions used in the remainder
of this chapter. So, we describe them here.

- Before attempting to use `gettextize` you should install some
  other packages first.
  Ensure that recent versions of GNU `m4`, GNU Autoconf and GNU
  `gettext` are already installed at your site, and if not, proceed
  to do this first. If you get to install these things, beware that
  GNU `m4` must be fully installed before GNU Autoconf is even
  *configured*.
  To further ease the task of a package maintainer the `automake`
  package was designed and implemented. GNU `gettext` now uses this
  tool and the ‘Makefile’s in the ‘intl/’ and ‘po/’
  therefore know about all the goals necessary for using `automake`
  and ‘libintl’ in one project.
  Those four packages are only needed by you, as a maintainer; the
  installers of your own package and end users do not really need any of
  GNU `m4`, GNU Autoconf, GNU `gettext`, or GNU `automake`
  for successfully installing and running your package, with messages
  properly translated. But this is not completely true if you provide
  internationalized shell scripts within your own package: GNU
  `gettext` shall then be installed at the user site if the end users
  want to see the translation of shell script messages.- Your package should use Autoconf and have a ‘configure.ac’ or
    ‘configure.in’ file.
    If it does not, you have to learn how. The Autoconf documentation
    is quite well written, it is a good idea that you print it and get
    familiar with it.- Your C sources should have already been modified according to
      instructions given earlier in this manual. See section 4 Preparing Program Sources.- Your ‘po/’ directory should receive all PO files submitted to you
        by the translator teams, each having ‘ll.po’ as a name.
        This is not usually easy to get translation
        work done before your package gets internationalized and available!
        Since the cycle has to start somewhere, the easiest for the maintainer
        is to start with absolutely no PO files, and wait until various
        translator teams get interested in your package, and submit PO files.

It is worth adding here a few words about how the maintainer should
ideally behave with PO files submissions. As a maintainer, your role is
to authenticate the origin of the submission as being the representative
of the appropriate translating teams of the Translation Project (forward
the submission to ‘coordinator@translationproject.org’ in case of doubt),
to ensure that the PO file format is not severely broken and does not
prevent successful installation, and for the rest, to merely put these
PO files in ‘po/’ for distribution.

As a maintainer, you do not have to take on your shoulders the
responsibility of checking if the translations are adequate or
complete, and should avoid diving into linguistic matters. Translation
teams drive themselves and are fully responsible of their linguistic
choices for the Translation Project. Keep in mind that translator teams are *not*
driven by maintainers. You can help by carefully redirecting all
communications and reports from users about linguistic matters to the
appropriate translation team, or explain users how to reach or join
their team. The simplest might be to send them the ‘ABOUT-NLS’ file.

Maintainers should *never ever* apply PO file bug reports
themselves, short-cutting translation teams. If some translator has
difficulty to get some of her points through her team, it should not be
an option for her to directly negotiate translations with maintainers.
Teams ought to settle their problems themselves, if any. If you, as
a maintainer, ever think there is a real problem with a team, please
never try to *solve* a team's problem on your own.

## 13.3 Invoking the `gettextize` Program

The `gettextize` program is an interactive tool that helps the
maintainer of a package internationalized through GNU `gettext`.
It is used for two purposes:

- As a wizard, when a package is modified to use GNU `gettext` for
  the first time.- As a migration tool, for upgrading the GNU `gettext` support in
    a package from a previous to a newer version of GNU `gettext`.

This program performs the following tasks:

- It copies into the package some files that are consistently and
  identically needed in every package internationalized through
  GNU `gettext`.- It performs as many of the tasks mentioned in the next section
    section 13.4 Files You Must Create or Alter as can be performed automatically.- It removes obsolete files and idioms used for previous GNU
      `gettext` versions to the form recommended for the current GNU
      `gettext` version.- It prints a summary of the tasks that ought to be done manually
        and could not be done automatically by `gettextize`.

It can be invoked as follows:

```
gettextize [ option... ] [ directory ]
```

and accepts the following options:

`‘-f’`: `‘--force’`: Force replacement of files which already exist. `‘--intl’`: Install the libintl sources in a subdirectory named ‘intl/’. This libintl will be used to provide internationalization on systems that don't have GNU libintl installed. If this option is omitted, the call to `AM_GNU_GETTEXT` in ‘configure.ac’ should read: `‘AM_GNU_GETTEXT([external])’`, and internationalization will not be enabled on systems lacking GNU gettext. `‘--po-dir=dir’`: Specify a directory containing PO files. Such a directory contains the translations into various languages of a particular POT file. This option can be specified multiple times, once for each translation domain. If it is not specified, the directory named ‘po/’ is updated. `‘--no-changelog’`: Don't update or create ChangeLog files. By default, `gettextize` logs all changes (file additions, modifications and removals) in a file called `‘ChangeLog’` in each affected directory. `‘--symlink’`: Make symbolic links instead of copying the needed files. This can be useful to save a few kilobytes of disk space, but it requires extra effort to create self-contained tarballs, it may disturb some mechanism the maintainer applies to the sources, and it is likely to introduce bugs when a newer version of `gettext` is installed on the system. `‘-n’`: `‘--dry-run’`: Print modifications but don't perform them. All actions that `gettextize` would normally execute are inhibited and instead only listed on standard output. `‘--help’`: Display this help and exit. `‘--version’`: Output version information and exit.

If directory is given, this is the top level directory of a
package to prepare for using GNU `gettext`. If not given, it
is assumed that the current directory is the top level directory of
such a package.

The program `gettextize` provides the following files. However,
no existing file will be replaced unless the option `--force`
(`-f`) is specified.

1. The ‘ABOUT-NLS’ file is copied in the main directory of your package,
   the one being at the top level. This file gives the main indications
   about how to install and use the Native Language Support features
   of your program. You might elect to use a more recent copy of this
   ‘ABOUT-NLS’ file than the one provided through `gettextize`,
   if you have one handy. You may also fetch a more recent copy of file
   ‘ABOUT-NLS’ from Translation Project sites, and from most GNU
   archive sites.- A ‘po/’ directory is created for eventually holding
     all translation files, but initially only containing the file
     ‘po/Makefile.in.in’ from the GNU `gettext` distribution
     (beware the double `‘.in’` in the file name) and a few auxiliary
     files. If the ‘po/’ directory already exists, it will be preserved
     along with the files it contains, and only ‘Makefile.in.in’ and
     the auxiliary files will be overwritten.
     If `‘--po-dir’` has been specified, this holds for every directory
     specified through `‘--po-dir’`, instead of ‘po/’.- Only if `‘--intl’` has been specified:
       A ‘intl/’ directory is created and filled with most of the files
       originally in the ‘intl/’ directory of the GNU `gettext`
       distribution. Also, if option `--force` (`-f`) is given,
       the ‘intl/’ directory is emptied first.- The file ‘config.rpath’ is copied into the directory containing
         configuration support files. It is needed by the `AM_GNU_GETTEXT`
         autoconf macro.- Only if the project is using GNU `automake`:
           A set of `autoconf` macro files is copied into the package's
           `autoconf` macro repository, usually in a directory called ‘m4/’.

If your site support symbolic links, `gettextize` will not
actually copy the files into your package, but establish symbolic
links instead. This avoids duplicating the disk space needed in
all packages. Merely using the `‘-h’` option while creating the
`tar` archive of your distribution will resolve each link by an
actual copy in the distribution archive. So, to insist, you really
should use `‘-h’` option with `tar` within your `dist`
goal of your main ‘Makefile.in’.

Furthermore, `gettextize` will update all ‘Makefile.am’ files
in each affected directory, as well as the top level ‘configure.ac’
or ‘configure.in’ file.

It is interesting to understand that most new files for supporting
GNU `gettext` facilities in one package go in ‘intl/’,
‘po/’ and ‘m4/’ subdirectories. One distinction between
‘intl/’ and the two other directories is that ‘intl/’ is
meant to be completely identical in all packages using GNU `gettext`,
while the other directories will mostly contain package dependent
files.

The `gettextize` program makes backup files for all files it
replaces or changes, and also write ChangeLog entries about these
changes. This way, the careful maintainer can check after running
`gettextize` whether its changes are acceptable to him, and
possibly adjust them. An exception to this rule is the ‘intl/’
directory, which is added or replaced or removed as a whole.

It is important to understand that `gettextize` can not do the
entire job of adapting a package for using GNU `gettext`. The
amount of remaining work depends on whether the package uses GNU
`automake` or not. But in any case, the maintainer should still
read the section section 13.4 Files You Must Create or Alter after invoking `gettextize`.

In particular, if after using `‘gettexize’`, you get an error
`‘AC_COMPILE_IFELSE was called before AC_GNU_SOURCE’` or
`‘AC_RUN_IFELSE was called before AC_GNU_SOURCE’`, you can fix it
by modifying ‘configure.ac’, as described in section 13.4.5 ‘configure.ac’ at top level.

It is also important to understand that `gettextize` is not part
of the GNU build system, in the sense that it should not be invoked
automatically, and not be invoked by someone who doesn't assume the
responsibilities of a package maintainer. For the latter purpose, a
separate tool is provided, see section 13.6.3 Invoking the `autopoint` Program.

## 13.4 Files You Must Create or Alter

Besides files which are automatically added through `gettextize`,
there are many files needing revision for properly interacting with
GNU `gettext`. If you are closely following GNU standards for
Makefile engineering and auto-configuration, the adaptations should
be easier to achieve. Here is a point by point description of the
changes needed in each.

So, here comes a list of files, each one followed by a description of
all alterations it needs. Many examples are taken out from the GNU
`gettext` 0.18 distribution itself, or from the GNU
`hello` distribution (http://www.franken.de/users/gnu/ke/hello
or http://www.gnu.franken.de/ke/hello/) You may indeed
refer to the source code of the GNU `gettext` and GNU `hello`
packages, as they are intended to be good examples for using GNU
gettext functionality.

### 13.4.1 ‘POTFILES.in’ in ‘po/’

The ‘po/’ directory should receive a file named
‘POTFILES.in’. This file tells which files, among all program
sources, have marked strings needing translation. Here is an example
of such a file:

```
# List of source files containing translatable strings.
# Copyright (C) 1995 Free Software Foundation, Inc.

# Common library files
lib/error.c
lib/getopt.c
lib/xmalloc.c

# Package source files
src/gettext.c
src/msgfmt.c
src/xgettext.c
```

Hash-marked comments and white lines are ignored. All other lines
list those source files containing strings marked for translation
(see section 4.4 How Marks Appear in Sources), in a notation relative to the top level
of your whole distribution, rather than the location of the
‘POTFILES.in’ file itself.

When a C file is automatically generated by a tool, like `flex` or
`bison`, that doesn't introduce translatable strings by itself,
it is recommended to list in ‘po/POTFILES.in’ the real source file
(ending in ‘.l’ in the case of `flex`, or in ‘.y’ in the
case of `bison`), not the generated C file.

### 13.4.2 ‘LINGUAS’ in ‘po/’

The ‘po/’ directory should also receive a file named
‘LINGUAS’. This file contains the list of available translations.
It is a whitespace separated list. Hash-marked comments and white lines
are ignored. Here is an example file:

```
# Set of available languages.
de fr
```

This example means that German and French PO files are available, so
that these languages are currently supported by your package. If you
want to further restrict, at installation time, the set of installed
languages, this should not be done by modifying the ‘LINGUAS’ file,
but rather by using the `LINGUAS` environment variable
(see section 14 The Installer's and Distributor's View).

It is recommended that you add the "languages" `‘en@quot’` and
`‘en@boldquot’` to the `LINGUAS` file. `en@quot` is a
variant of English message catalogs (`en`) which uses real quotation
marks instead of the ugly looking asymmetric ASCII substitutes `` ‘`’ ``
and `‘'’`. `en@boldquot` is a variant of `en@quot` that
additionally outputs quoted pieces of text in a bold font, when used in
a terminal emulator which supports the VT100 escape sequences (such as
`xterm` or the Linux console, but not Emacs in `M-x shell` mode).

These extra message catalogs `‘en@quot’` and `‘en@boldquot’`
are constructed automatically, not by translators; to support them, you
need the files ‘Rules-quot’, ‘quot.sed’, ‘boldquot.sed’,
‘en@quot.header’, ‘en@boldquot.header’, ‘insert-header.sin’
in the ‘po/’ directory. You can copy them from GNU gettext's ‘po/’
directory; they are also installed by running `gettextize`.

### 13.4.3 ‘Makevars’ in ‘po/’

The ‘po/’ directory also has a file named ‘Makevars’. It
contains variables that are specific to your project. ‘po/Makevars’
gets inserted into the ‘po/Makefile’ when the latter is created.
The variables thus take effect when the POT file is created or updated,
and when the message catalogs get installed.

The first three variables can be left unmodified if your package has a
single message domain and, accordingly, a single ‘po/’ directory.
Only packages which have multiple ‘po/’ directories at different
locations need to adjust the three first variables defined in
‘Makevars’.

As an alternative to the `XGETTEXT_OPTIONS` variables, it is also
possible to specify `xgettext` options through the
`AM_XGETTEXT_OPTION` autoconf macro. See section 13.5.6 AM\_XGETTEXT\_OPTION in ‘po.m4’.

### 13.4.4 Extending ‘Makefile’ in ‘po/’

All files called ‘Rules-\*’ in the ‘po/’ directory get appended to
the ‘po/Makefile’ when it is created. They present an opportunity to
add rules for special PO files to the Makefile, without needing to mess
with ‘po/Makefile.in.in’.

GNU gettext comes with a ‘Rules-quot’ file, containing rules for
building catalogs ‘en@quot.po’ and ‘en@boldquot.po’. The
effect of ‘en@quot.po’ is that people who set their `LANGUAGE`
environment variable to `‘en@quot’` will get messages with proper
looking symmetric Unicode quotation marks instead of abusing the ASCII
grave accent and the ASCII apostrophe for indicating quotations. To
enable this catalog, simply add `en@quot` to the ‘po/LINGUAS’
file. The effect of ‘en@boldquot.po’ is that people who set
`LANGUAGE` to `‘en@boldquot’` will get not only proper quotation
marks, but also the quoted text will be shown in a bold font on terminals
and consoles. This catalog is useful only for command-line programs, not
GUI programs. To enable it, similarly add `en@boldquot` to the
‘po/LINGUAS’ file.

Similarly, you can create rules for building message catalogs for the
‘sr@latin’ locale -- Serbian written with the Latin alphabet --
from those for the ‘sr’ locale -- Serbian written with Cyrillic
letters. See section 9.4 Invoking the `msgfilter` Program.

### 13.4.5 ‘configure.ac’ at top level

‘configure.ac’ or ‘configure.in’ - this is the source from which
`autoconf` generates the ‘configure’ script.

1. Declare the package and version.
   This is done by a set of lines like these:

   ```
   PACKAGE=gettext
   VERSION=0.18
   AC_DEFINE_UNQUOTED(PACKAGE, "$PACKAGE")
   AC_DEFINE_UNQUOTED(VERSION, "$VERSION")
   AC_SUBST(PACKAGE)
   AC_SUBST(VERSION)
   ```

   or, if you are using GNU `automake`, by a line like this:

   ```
   AM_INIT_AUTOMAKE(gettext, 0.18)
   ```

   Of course, you replace `‘gettext’` with the name of your package,
   and `‘0.18’` by its version numbers, exactly as they
   should appear in the packaged `tar` file name of your distribution
   (‘gettext-0.18.tar.gz’, here).- Check for internationalization support.
     Here is the main `m4` macro for triggering internationalization
     support. Just add this line to ‘configure.ac’:

     ```
     AM_GNU_GETTEXT
     ```

     This call is purposely simple, even if it generates a lot of configure
     time checking and actions.
     If you have suppressed the ‘intl/’ subdirectory by calling
     `gettextize` without `‘--intl’` option, this call should read

     ```
     AM_GNU_GETTEXT([external])
     ```

     - Have output files created.
       The `AC_OUTPUT` directive, at the end of your ‘configure.ac’
       file, needs to be modified in two ways:

       ```
       AC_OUTPUT([existing configuration files intl/Makefile po/Makefile.in],
       [existing additional actions])
       ```

       The modification to the first argument to `AC_OUTPUT` asks
       for substitution in the ‘intl/’ and ‘po/’ directories.
       Note the `‘.in’` suffix used for ‘po/’ only. This is because
       the distributed file is really ‘po/Makefile.in.in’.
       If you have suppressed the ‘intl/’ subdirectory by calling
       `gettextize` without `‘--intl’` option, then you don't need to
       add `intl/Makefile` to the `AC_OUTPUT` line.

If, after doing the recommended modifications, a command like
`‘aclocal -I m4’` or `‘autoconf’` or `‘autoreconf’` fails with
a trace similar to this:

```
configure.ac:44: warning: AC_COMPILE_IFELSE was called before AC_GNU_SOURCE
../../lib/autoconf/specific.m4:335: AC_GNU_SOURCE is expanded from...
m4/lock.m4:224: gl_LOCK is expanded from...
m4/gettext.m4:571: gt_INTL_SUBDIR_CORE is expanded from...
m4/gettext.m4:472: AM_INTL_SUBDIR is expanded from...
m4/gettext.m4:347: AM_GNU_GETTEXT is expanded from...
configure.ac:44: the top level
configure.ac:44: warning: AC_RUN_IFELSE was called before AC_GNU_SOURCE
```

you need to add an explicit invocation of `‘AC_GNU_SOURCE’` in the
‘configure.ac’ file - after `‘AC_PROG_CC’` but before
`‘AM_GNU_GETTEXT’`, most likely very close to the `‘AC_PROG_CC’`
invocation. This is necessary because of ordering restrictions imposed
by GNU autoconf.

### 13.4.6 ‘config.guess’, ‘config.sub’ at top level

If you haven't suppressed the ‘intl/’ subdirectory,
you need to add the GNU ‘config.guess’ and ‘config.sub’ files
to your distribution. They are needed because the ‘intl/’ directory
has platform dependent support for determining the locale's character
encoding and therefore needs to identify the platform.

You can obtain the newest version of ‘config.guess’ and
‘config.sub’ from the CVS of the `‘config’` project at
‘http://savannah.gnu.org/’. The commands to fetch them are

```
$ wget 'http://savannah.gnu.org/cgi-bin/viewcvs/*checkout*/config/config/config.guess'
$ wget 'http://savannah.gnu.org/cgi-bin/viewcvs/*checkout*/config/config/config.sub'
```

Less recent versions are also contained in the GNU `automake` and
GNU `libtool` packages.

Normally, ‘config.guess’ and ‘config.sub’ are put at the
top level of a distribution. But it is also possible to put them in a
subdirectory, altogether with other configuration support files like
‘install-sh’, ‘ltconfig’, ‘ltmain.sh’ or ‘missing’.
All you need to do, other than moving the files, is to add the following line
to your ‘configure.ac’.

```
AC_CONFIG_AUX_DIR([subdir])
```

### 13.4.7 ‘mkinstalldirs’ at top level

With earlier versions of GNU gettext, you needed to add the GNU
‘mkinstalldirs’ script to your distribution. This is not needed any
more. You can remove it if you not also using an automake version older than
automake 1.9.

### 13.4.8 ‘aclocal.m4’ at top level

If you do not have an ‘aclocal.m4’ file in your distribution,
the simplest is to concatenate the files ‘codeset.m4’, ‘fcntl-o.m4’,
‘gettext.m4’, ‘glibc2.m4’, ‘glibc21.m4’, ‘iconv.m4’,
‘intdiv0.m4’, ‘intl.m4’, ‘intldir.m4’, ‘intlmacosx.m4’,
‘intmax.m4’, ‘inttypes\_h.m4’, ‘inttypes-pri.m4’,
‘lcmessage.m4’, ‘lib-ld.m4’, ‘lib-link.m4’,
‘lib-prefix.m4’, ‘lock.m4’, ‘longlong.m4’, ‘nls.m4’,
‘po.m4’, ‘printf-posix.m4’, ‘progtest.m4’, ‘size\_max.m4’,
‘stdint\_h.m4’, ‘threadlib.m4’, ‘uintmax\_t.m4’,
‘visibility.m4’, ‘wchar\_t.m4’, ‘wint\_t.m4’, ‘xsize.m4’
from GNU `gettext`'s
‘m4/’ directory into a single file. If you have suppressed the
‘intl/’ directory, only ‘gettext.m4’, ‘iconv.m4’,
‘lib-ld.m4’, ‘lib-link.m4’, ‘lib-prefix.m4’,
‘nls.m4’, ‘po.m4’, ‘progtest.m4’ need to be concatenated.

If you are not using GNU `automake` 1.8 or newer, you will need to
add a file ‘mkdirp.m4’ from a newer automake distribution to the
list of files above.

If you already have an ‘aclocal.m4’ file, then you will have
to merge the said macro files into your ‘aclocal.m4’. Note that if
you are upgrading from a previous release of GNU `gettext`, you
should most probably *replace* the macros (`AM_GNU_GETTEXT`,
etc.), as they usually
change a little from one release of GNU `gettext` to the next.
Their contents may vary as we get more experience with strange systems
out there.

If you are using GNU `automake` 1.5 or newer, it is enough to put
these macro files into a subdirectory named ‘m4/’ and add the line

```
ACLOCAL_AMFLAGS = -I m4
```

to your top level ‘Makefile.am’.

These macros check for the internationalization support functions
and related informations. Hopefully, once stabilized, these macros
might be integrated in the standard Autoconf set, because this
piece of `m4` code will be the same for all projects using GNU
`gettext`.

### 13.4.9 ‘acconfig.h’ at top level

Earlier GNU `gettext` releases required to put definitions for
`ENABLE_NLS`, `HAVE_GETTEXT` and `HAVE_LC_MESSAGES`,
`HAVE_STPCPY`, `PACKAGE` and `VERSION` into an
‘acconfig.h’ file. This is not needed any more; you can remove
them from your ‘acconfig.h’ file unless your package uses them
independently from the ‘intl/’ directory.

### 13.4.10 ‘config.h.in’ at top level

The include file template that holds the C macros to be defined by
`configure` is usually called ‘config.h.in’ and may be
maintained either manually or automatically.

If `gettextize` has created an ‘intl/’ directory, this file
must be called ‘config.h.in’ and must be at the top level. If,
however, you have suppressed the ‘intl/’ directory by calling
`gettextize` without `‘--intl’` option, then you can choose the
name of this file and its location freely.

If it is maintained automatically, by use of the `‘autoheader’`
program, you need to do nothing about it. This is the case in particular
if you are using GNU `automake`.

If it is maintained manually, and if `gettextize` has created an
‘intl/’ directory, you should switch to using `‘autoheader’`.
The list of C macros to be added for the sake of the ‘intl/’
directory is just too long to be maintained manually; it also changes
between different versions of GNU `gettext`.

If it is maintained manually, and if on the other hand you have
suppressed the ‘intl/’ directory by calling `gettextize`
without `‘--intl’` option, then you can get away by adding the
following lines to ‘config.h.in’:

```
/* Define to 1 if translation of program messages to the user's
   native language is requested. */
#undef ENABLE_NLS
```

### 13.4.11 ‘Makefile.in’ at top level

Here are a few modifications you need to make to your main, top-level
‘Makefile.in’ file.

1. Add the following lines near the beginning of your ‘Makefile.in’,
   so the `‘dist:’` goal will work properly (as explained further down):

   ```
   PACKAGE = @PACKAGE@
   VERSION = @VERSION@
   ```

   - Add file ‘ABOUT-NLS’ to the `DISTFILES` definition, so the file gets
     distributed.- Wherever you process subdirectories in your ‘Makefile.in’, be sure
       you also process the subdirectories `‘intl’` and `‘po’`. Special
       rules in the ‘Makefiles’ take care for the case where no
       internationalization is wanted.
       If you are using Makefiles, either generated by automake, or hand-written
       so they carefully follow the GNU coding standards, the effected goals for
       which the new subdirectories must be handled include `‘installdirs’`,
       `‘install’`, `‘uninstall’`, `‘clean’`, `‘distclean’`.
       Here is an example of a canonical order of processing. In this
       example, we also define `SUBDIRS` in `Makefile.in` for it
       to be further used in the `‘dist:’` goal.

       ```
       SUBDIRS = doc intl lib src po
       ```

       Note that you must arrange for `‘make’` to descend into the
       `intl` directory before descending into other directories containing
       code which make use of the `libintl.h` header file. For this
       reason, here we mention `intl` before `lib` and `src`.- A delicate point is the `‘dist:’` goal, as both
         ‘intl/Makefile’ and ‘po/Makefile’ will later assume that the
         proper directory has been set up from the main ‘Makefile’. Here is
         an example at what the `‘dist:’` goal might look like:

         ```
         distdir = $(PACKAGE)-$(VERSION)
         dist: Makefile
         	rm -fr $(distdir)
         	mkdir $(distdir)
         	chmod 777 $(distdir)
         	for file in $(DISTFILES); do \
         	  ln $$file $(distdir) 2>/dev/null || cp -p $$file $(distdir); \
         	done
         	for subdir in $(SUBDIRS); do \
         	  mkdir $(distdir)/$$subdir || exit 1; \
         	  chmod 777 $(distdir)/$$subdir; \
         	  (cd $$subdir && $(MAKE) $@) || exit 1; \
         	done
         	tar chozf $(distdir).tar.gz $(distdir)
         	rm -fr $(distdir)
         ```

Note that if you are using GNU `automake`, ‘Makefile.in’ is
automatically generated from ‘Makefile.am’, and all needed changes
to ‘Makefile.am’ are already made by running `‘gettextize’`.

### 13.4.12 ‘Makefile.in’ in ‘src/’

Some of the modifications made in the main ‘Makefile.in’ will
also be needed in the ‘Makefile.in’ from your package sources,
which we assume here to be in the ‘src/’ subdirectory. Here are
all the modifications needed in ‘src/Makefile.in’:

1. In view of the `‘dist:’` goal, you should have these lines near the
   beginning of ‘src/Makefile.in’:

   ```
   PACKAGE = @PACKAGE@
   VERSION = @VERSION@
   ```

   - If not done already, you should guarantee that `top_srcdir`
     gets defined. This will serve for `cpp` include files. Just add
     the line:

     ```
     top_srcdir = @top_srcdir@
     ```

     - You might also want to define `subdir` as `‘src’`, later
       allowing for almost uniform `‘dist:’` goals in all your
       ‘Makefile.in’. At list, the `‘dist:’` goal below assume that
       you used:

       ```
       subdir = src
       ```

       - The `main` function of your program will normally call
         `bindtextdomain` (see see section 4.2 Triggering `gettext` Operations), like this:

         ```
         bindtextdomain (PACKAGE, LOCALEDIR);
         textdomain (PACKAGE);
         ```

         To make LOCALEDIR known to the program, add the following lines to
         ‘Makefile.in’ if you are using Autoconf version 2.60 or newer:

         ```
         datadir = @datadir@
         datarootdir= @datarootdir@
         localedir = @localedir@
         DEFS = -DLOCALEDIR=\"$(localedir)\" @DEFS@
         ```

         or these lines if your version of Autoconf is older than 2.60:

         ```
         datadir = @datadir@
         localedir = $(datadir)/locale
         DEFS = -DLOCALEDIR=\"$(localedir)\" @DEFS@
         ```

         Note that `@datadir@` defaults to `‘$(prefix)/share’`, thus
         `$(localedir)` defaults to `‘$(prefix)/share/locale’`.- You should ensure that the final linking will use `@LIBINTL@` or
           `@LTLIBINTL@` as a library. `@LIBINTL@` is for use without
           `libtool`, `@LTLIBINTL@` is for use with `libtool`. An
           easy way to achieve this is to manage that it gets into `LIBS`, like
           this:

           ```
           LIBS = @LIBINTL@ @LIBS@
           ```

           In most packages internationalized with GNU `gettext`, one will
           find a directory ‘lib/’ in which a library containing some helper
           functions will be build. (You need at least the few functions which the
           GNU `gettext` Library itself needs.) However some of the functions
           in the ‘lib/’ also give messages to the user which of course should be
           translated, too. Taking care of this, the support library (say
           ‘libsupport.a’) should be placed before `@LIBINTL@` and
           `@LIBS@` in the above example. So one has to write this:

           ```
           LIBS = ../lib/libsupport.a @LIBINTL@ @LIBS@
           ```

           - You should also ensure that directory ‘intl/’ will be searched for
             C preprocessor include files in all circumstances. So, you have to
             manage so both `‘-I../intl’` and `‘-I$(top_srcdir)/intl’` will
             be given to the C compiler.- Your `‘dist:’` goal has to conform with others. Here is a
               reasonable definition for it:

               ```
               distdir = ../$(PACKAGE)-$(VERSION)/$(subdir)
               dist: Makefile $(DISTFILES)
               	for file in $(DISTFILES); do \
               	  ln $$file $(distdir) 2>/dev/null || cp -p $$file $(distdir) || exit 1; \
               	done
               ```

Note that if you are using GNU `automake`, ‘Makefile.in’ is
automatically generated from ‘Makefile.am’, and the first three
changes and the last change are not necessary. The remaining needed
‘Makefile.am’ modifications are the following:

1. To make LOCALEDIR known to the program, add the following to
   ‘Makefile.am’:

   ```
   <module>_CPPFLAGS = -DLOCALEDIR=\"$(localedir)\"
   ```

   for each specific module or compilation unit, or

   ```
   AM_CPPFLAGS = -DLOCALEDIR=\"$(localedir)\"
   ```

   for all modules and compilation units together. Furthermore, if you are
   using an Autoconf version older then 2.60, add this line to define
   `‘localedir’`:

   ```
   localedir = $(datadir)/locale
   ```

   - To ensure that the final linking will use `@LIBINTL@` or
     `@LTLIBINTL@` as a library, add the following to
     ‘Makefile.am’:

     ```
     <program>_LDADD = @LIBINTL@
     ```

     for each specific program, or

     ```
     LDADD = @LIBINTL@
     ```

     for all programs together. Remember that when you use `libtool`
     to link a program, you need to use @LTLIBINTL@ instead of @LIBINTL@
     for that program.- If you have an ‘intl/’ directory, whose contents is created by
       `gettextize`, then to ensure that it will be searched for
       C preprocessor include files in all circumstances, add something like
       this to ‘Makefile.am’:

       ```
       AM_CPPFLAGS = -I../intl -I$(top_srcdir)/intl
       ```

### 13.4.13 ‘gettext.h’ in ‘lib/’

Internationalization of packages, as provided by GNU `gettext`, is
optional. It can be turned off in two situations:

- When the installer has specified `‘./configure --disable-nls’`. This
  can be useful when small binaries are more important than features, for
  example when building utilities for boot diskettes. It can also be useful
  in order to get some specific C compiler warnings about code quality with
  some older versions of GCC (older than 3.0).- When the package does not include the `intl/` subdirectory, and the
    libintl.h header (with its associated libintl library, if any) is not
    already installed on the system, it is preferable that the package builds
    without internationalization support, rather than to give a compilation
    error.

A C preprocessor macro can be used to detect these two cases. Usually,
when `libintl.h` was found and not explicitly disabled, the
`ENABLE_NLS` macro will be defined to 1 in the autoconf generated
configuration file (usually called ‘config.h’). In the two negative
situations, however, this macro will not be defined, thus it will evaluate
to 0 in C preprocessor expressions.

‘gettext.h’ is a convenience header file for conditional use of
‘<libintl.h>’, depending on the `ENABLE_NLS` macro. If
`ENABLE_NLS` is set, it includes ‘<libintl.h>’; otherwise it
defines no-op substitutes for the libintl.h functions. We recommend
the use of `"gettext.h"` over direct use of ‘<libintl.h>’,
so that portability to older systems is guaranteed and installers can
turn off internationalization if they want to. In the C code, you will
then write

```
#include "gettext.h"
```

instead of

```
#include <libintl.h>
```

The location of `gettext.h` is usually in a directory containing
auxiliary include files. In many GNU packages, there is a directory
‘lib/’ containing helper functions; ‘gettext.h’ fits there.
In other packages, it can go into the ‘src’ directory.

Do not install the `gettext.h` file in public locations. Every
package that needs it should contain a copy of it on its own.

## 13.5 Autoconf macros for use in ‘configure.ac’

GNU `gettext` installs macros for use in a package's
‘configure.ac’ or ‘configure.in’.
See section ‘Introduction’ in The Autoconf Manual.
The primary macro is, of course, `AM_GNU_GETTEXT`.

### 13.5.1 AM\_GNU\_GETTEXT in ‘gettext.m4’

The `AM_GNU_GETTEXT` macro tests for the presence of the GNU gettext
function family in either the C library or a separate `libintl`
library (shared or static libraries are both supported) or in the package's
‘intl/’ directory. It also invokes `AM_PO_SUBDIRS`, thus preparing
the ‘po/’ directories of the package for building.

`AM_GNU_GETTEXT` accepts up to three optional arguments. The general
syntax is

```
AM_GNU_GETTEXT([intlsymbol], [needsymbol], [intldir])
```

intlsymbol can be `‘external’` or `‘no-libtool’`. The default
(if it is not specified or empty) is `‘no-libtool’`. intlsymbol
should be `‘external’` for packages with no ‘intl/’ directory.
For packages with an ‘intl/’ directory, you can either use an
intlsymbol equal to `‘no-libtool’`, or you can use `‘external’`
and override by using the macro `AM_GNU_GETTEXT_INTL_SUBDIR` elsewhere.
The two ways to specify the existence of an ‘intl/’ directory are
equivalent. At build time, a static library
`$(top_builddir)/intl/libintl.a` will then be created.

If needsymbol is specified and is `‘need-ngettext’`, then GNU
gettext implementations (in libc or libintl) without the `ngettext()`
function will be ignored. If needsymbol is specified and is
`‘need-formatstring-macros’`, then GNU gettext implementations that don't
support the ISO C 99 ‘<inttypes.h>’ formatstring macros will be ignored.
Only one needsymbol can be specified. These requirements can also be
specified by using the macro `AM_GNU_GETTEXT_NEED` elsewhere. To specify
more than one requirement, just specify the strongest one among them, or
invoke the `AM_GNU_GETTEXT_NEED` macro several times. The hierarchy
among the various alternatives is as follows: `‘need-formatstring-macros’`
implies `‘need-ngettext’`.

intldir is used to find the intl libraries. If empty, the value
`‘$(top_builddir)/intl/’` is used.

The `AM_GNU_GETTEXT` macro determines whether GNU gettext is
available and should be used. If so, it sets the `USE_NLS` variable
to `‘yes’`; it defines `ENABLE_NLS` to 1 in the autoconf
generated configuration file (usually called ‘config.h’); it sets
the variables `LIBINTL` and `LTLIBINTL` to the linker options
for use in a Makefile (`LIBINTL` for use without libtool,
`LTLIBINTL` for use with libtool); it adds an `‘-I’` option to
`CPPFLAGS` if necessary. In the negative case, it sets
`USE_NLS` to `‘no’`; it sets `LIBINTL` and `LTLIBINTL`
to empty and doesn't change `CPPFLAGS`.

The complexities that `AM_GNU_GETTEXT` deals with are the following:

- Some operating systems have `gettext` in the C library, for example
  glibc. Some have it in a separate library `libintl`. GNU `libintl`
  might have been installed as part of the GNU `gettext` package.- GNU `libintl`, if installed, is not necessarily already in the search
    path (`CPPFLAGS` for the include file search path, `LDFLAGS` for
    the library search path).- Except for glibc, the operating system's native `gettext` cannot
      exploit the GNU mo files, doesn't have the necessary locale dependency
      features, and cannot convert messages from the catalog's text encoding
      to the user's locale encoding.- GNU `libintl`, if installed, is not necessarily already in the
        run time library search path. To avoid the need for setting an environment
        variable like `LD_LIBRARY_PATH`, the macro adds the appropriate
        run time search path options to the `LIBINTL` and `LTLIBINTL`
        variables. This works on most systems, but not on some operating systems
        with limited shared library support, like SCO.- GNU `libintl` relies on POSIX/XSI `iconv`. The macro checks for
          linker options needed to use iconv and appends them to the `LIBINTL`
          and `LTLIBINTL` variables.

### 13.5.2 AM\_GNU\_GETTEXT\_VERSION in ‘gettext.m4’

The `AM_GNU_GETTEXT_VERSION` macro declares the version number of
the GNU gettext infrastructure that is used by the package.

The use of this macro is optional; only the `autopoint` program makes
use of it (see section 13.6 Integrating with CVS).

### 13.5.3 AM\_GNU\_GETTEXT\_NEED in ‘gettext.m4’

The `AM_GNU_GETTEXT_NEED` macro declares a constraint regarding the
GNU gettext implementation. The syntax is

```
AM_GNU_GETTEXT_NEED([needsymbol])
```

If needsymbol is `‘need-ngettext’`, then GNU gettext implementations
(in libc or libintl) without the `ngettext()` function will be ignored.
If needsymbol is `‘need-formatstring-macros’`, then GNU gettext
implementations that don't support the ISO C 99 ‘<inttypes.h>’
formatstring macros will be ignored.

The optional second argument of `AM_GNU_GETTEXT` is also taken into
account.

The `AM_GNU_GETTEXT_NEED` invocations can occur before or after
the `AM_GNU_GETTEXT` invocation; the order doesn't matter.

### 13.5.4 AM\_GNU\_GETTEXT\_INTL\_SUBDIR in ‘intldir.m4’

The `AM_GNU_GETTEXT_INTL_SUBDIR` macro specifies that the
`AM_GNU_GETTEXT` macro, although invoked with the first argument
`‘external’`, should also prepare for building the ‘intl/’
subdirectory.

The `AM_GNU_GETTEXT_INTL_SUBDIR` invocation can occur before or after
the `AM_GNU_GETTEXT` invocation; the order doesn't matter.

The use of this macro requires GNU automake 1.10 or newer and
GNU autoconf 2.61 or newer.

### 13.5.5 AM\_PO\_SUBDIRS in ‘po.m4’

The `AM_PO_SUBDIRS` macro prepares the ‘po/’ directories of the
package for building. This macro should be used in internationalized
programs written in other programming languages than C, C++, Objective C,
for example `sh`, `Python`, `Lisp`. See section 15 Other Programming Languages for a list of programming languages that support localization
through PO files.

The `AM_PO_SUBDIRS` macro determines whether internationalization
should be used. If so, it sets the `USE_NLS` variable to `‘yes’`,
otherwise to `‘no’`. It also determines the right values for Makefile
variables in each ‘po/’ directory.

### 13.5.6 AM\_XGETTEXT\_OPTION in ‘po.m4’

The `AM_XGETTEXT_OPTION` macro registers a command-line option to be
used in the invocations of `xgettext` in the ‘po/’ directories
of the package.

For example, if you have a source file that defines a function
`‘error_at_line’` whose fifth argument is a format string, you can use

```
AM_XGETTEXT_OPTION([--flag=error_at_line:5:c-format])
```

to instruct `xgettext` to mark all translatable strings in `‘gettext’`
invocations that occur as fifth argument to this function as `‘c-format’`.

See section 5.1 Invoking the `xgettext` Program for the list of options that `xgettext`
accepts.

The use of this macro is an alternative to the use of the
`‘XGETTEXT_OPTIONS’` variable in ‘po/Makevars’.

### 13.5.7 AM\_ICONV in ‘iconv.m4’

The `AM_ICONV` macro tests for the presence of the POSIX/XSI
`iconv` function family in either the C library or a separate
`libiconv` library. If found, it sets the `am_cv_func_iconv`
variable to `‘yes’`; it defines `HAVE_ICONV` to 1 in the autoconf
generated configuration file (usually called ‘config.h’); it defines
`ICONV_CONST` to `‘const’` or to empty, depending on whether the
second argument of `iconv()` is of type `‘const char **’` or
`‘char **’`; it sets the variables `LIBICONV` and
`LTLIBICONV` to the linker options for use in a Makefile
(`LIBICONV` for use without libtool, `LTLIBICONV` for use with
libtool); it adds an `‘-I’` option to `CPPFLAGS` if
necessary. If not found, it sets `LIBICONV` and `LTLIBICONV` to
empty and doesn't change `CPPFLAGS`.

The complexities that `AM_ICONV` deals with are the following:

- Some operating systems have `iconv` in the C library, for example
  glibc. Some have it in a separate library `libiconv`, for example
  OSF/1 or FreeBSD. Regardless of the operating system, GNU `libiconv`
  might have been installed. In that case, it should be used instead of the
  operating system's native `iconv`.- GNU `libiconv`, if installed, is not necessarily already in the search
    path (`CPPFLAGS` for the include file search path, `LDFLAGS` for
    the library search path).- GNU `libiconv` is binary incompatible with some operating system's
      native `iconv`, for example on FreeBSD. Use of an ‘iconv.h’
      and ‘libiconv.so’ that don't fit together would produce program
      crashes.- GNU `libiconv`, if installed, is not necessarily already in the
        run time library search path. To avoid the need for setting an environment
        variable like `LD_LIBRARY_PATH`, the macro adds the appropriate
        run time search path options to the `LIBICONV` variable. This works
        on most systems, but not on some operating systems with limited shared
        library support, like SCO.

‘iconv.m4’ is distributed with the GNU gettext package because
‘gettext.m4’ relies on it.

## 13.6 Integrating with CVS

Many projects use CVS for distributed development, version control and
source backup. This section gives some advice how to manage the uses
of `cvs`, `gettextize`, `autopoint` and `autoconf`.

### 13.6.1 Avoiding version mismatch in distributed development

In a project development with multiple developers, using CVS, there
should be a single developer who occasionally - when there is desire to
upgrade to a new `gettext` version - runs `gettextize` and
performs the changes listed in section 13.4 Files You Must Create or Alter, and then commits
his changes to the CVS.

It is highly recommended that all developers on a project use the same
version of GNU `gettext` in the package. In other words, if a
developer runs `gettextize`, he should go the whole way, make the
necessary remaining changes and commit his changes to the CVS.
Otherwise the following damages will likely occur:

- Apparent version mismatch between developers. Since some `gettext`
  specific portions in ‘configure.ac’, ‘configure.in’ and
  `Makefile.am`, `Makefile.in` files depend on the `gettext`
  version, the use of infrastructure files belonging to different
  `gettext` versions can easily lead to build errors.- Hidden version mismatch. Such version mismatch can also lead to
    malfunctioning of the package, that may be undiscovered by the developers.
    The worst case of hidden version mismatch is that internationalization
    of the package doesn't work at all.- Release risks. All developers implicitly perform constant testing on
      a package. This is important in the days and weeks before a release.
      If the guy who makes the release tar files uses a different version
      of GNU `gettext` than the other developers, the distribution will
      be less well tested than if all had been using the same `gettext`
      version. For example, it is possible that a platform specific bug goes
      undiscovered due to this constellation.

### 13.6.2 Files to put under CVS version control

There are basically three ways to deal with generated files in the
context of a CVS repository, such as ‘configure’ generated from
‘configure.ac’, `parser.c` generated from
`parser.y`, or `po/Makefile.in.in` autoinstalled by
`gettextize` or `autopoint`.

1. All generated files are always committed into the repository.- All generated files are committed into the repository occasionally,
     for example each time a release is made.- Generated files are never committed into the repository.

Each of these three approaches has different advantages and drawbacks.

1. The advantage is that anyone can check out the CVS at any moment and
   gets a working build. The drawbacks are: 1a. It requires some frequent
   "cvs commit" actions by the maintainers. 1b. The repository grows in size
   quite fast.- The advantage is that anyone can check out the CVS, and the usual
     "./configure; make" will work. The drawbacks are: 2a. The one who
     checks out the repository needs tools like GNU `automake`,
     GNU `autoconf`, GNU `m4` installed in his PATH; sometimes
     he even needs particular versions of them. 2b. When a release is made
     and a commit is made on the generated files, the other developers get
     conflicts on the generated files after doing "cvs update". Although
     these conflicts are easy to resolve, they are annoying.- The advantage is less work for the maintainers. The drawback is that
       anyone who checks out the CVS not only needs tools like GNU `automake`,
       GNU `autoconf`, GNU `m4` installed in his PATH, but also that
       he needs to perform a package specific pre-build step before being able
       to "./configure; make".

For the first and second approach, all files modified or brought in
by the occasional `gettextize` invocation and update should be
committed into the CVS.

For the third approach, the maintainer can omit from the CVS repository
all the files that `gettextize` mentions as "copy". Instead, he
adds to the ‘configure.ac’ or ‘configure.in’ a line of the
form

```
AM_GNU_GETTEXT_VERSION(0.18)
```

and adds to the package's pre-build script an invocation of
`‘autopoint’`. For everyone who checks out the CVS, this
`autopoint` invocation will copy into the right place the
`gettext` infrastructure files that have been omitted from the CVS.

The version number used as argument to `AM_GNU_GETTEXT_VERSION` is
the version of the `gettext` infrastructure that the package wants
to use. It is also the minimum version number of the `‘autopoint’`
program. So, if you write `AM_GNU_GETTEXT_VERSION(0.11.5)` then the
developers can have any version >= 0.11.5 installed; the package will work
with the 0.11.5 infrastructure in all developers' builds. When the
maintainer then runs gettextize from, say, version 0.12.1 on the package,
the occurrence of `AM_GNU_GETTEXT_VERSION(0.11.5)` will be changed
into `AM_GNU_GETTEXT_VERSION(0.12.1)`, and all other developers that
use the CVS will henceforth need to have GNU `gettext` 0.12.1 or newer
installed.

### 13.6.3 Invoking the `autopoint` Program

```
autopoint [option]...
```

The `autopoint` program copies standard gettext infrastructure files
into a source package. It extracts from a macro call of the form
`AM_GNU_GETTEXT_VERSION(version)`, found in the package's
‘configure.in’ or ‘configure.ac’ file, the gettext version
used by the package, and copies the infrastructure files belonging to
this version into the package.

#### 13.6.3.1 Options

`‘-f’`: `‘--force’`: Force overwriting of files that already exist. `‘-n’`: `‘--dry-run’`: Print modifications but don't perform them. All file copying actions that `autopoint` would normally execute are inhibited and instead only listed on standard output.

#### 13.6.3.2 Informative output

`‘--help’`: Display this help and exit. `‘--version’`: Output version information and exit.

`autopoint` supports the GNU `gettext` versions from 0.10.35 to
the current one, 0.18. In order to apply `autopoint` to
a package using a `gettext` version newer than 0.18, you
need to install this same version of GNU `gettext` at least.

In packages using GNU `automake`, an invocation of `autopoint`
should be followed by invocations of `aclocal` and then `autoconf`
and `autoheader`. The reason is that `autopoint` installs some
autoconf macro files, which are used by `aclocal` to create
‘aclocal.m4’, and the latter is used by `autoconf` to create the
package's ‘configure’ script and by `autoheader` to create the
package's ‘config.h.in’ include file template.

The name `‘autopoint’` is an abbreviation of `‘auto-po-intl-m4’`;
the tool copies or updates mostly files in the ‘po’, ‘intl’,
‘m4’ directories.

## 13.7 Creating a Distribution Tarball

In projects that use GNU `automake`, the usual commands for creating
a distribution tarball, `‘make dist’` or `‘make distcheck’`,
automatically update the PO files as needed.

If GNU `automake` is not used, the maintainer needs to perform this
update before making a release:

```
$ ./configure
$ (cd po; make update-po)
$ make distclean
```

---

Go to the first, previous, next, last section, table of contents.
