# Supplementary material for: rDock: A Fast, Versatile and Open Source Program for Docking Ligands to Proteins and Nucleic Acids
Source: PLoS Comput Biol. 2014 Apr 10;10(4):e1003571. doi: 10.1371/journal.pcbi.1003571 (PMC3983074; doi:10.1371/journal.pcbi.1003571)
Supplement: Software S1 — Compressed file with the source code of the rDock software for ligand docking to Proteins and Nucleic Acids. (GZ) [file pcbi.1003571.s010.gz › rDock_2013.1_src/gettext-0.18.tar.gz!/gettext-0.18/gettext-tools/doc/gettext_17.html]

GNU gettext utilities - A Language Codes

Go to the first, previous, next, last section, table of contents.

---

# A Language Codes

The ISO 639 standard defines two-letter codes for many languages, and
three-letter codes for more rarely used languages.
All abbreviations for languages used in the Translation Project should
come from this standard.

## A.1 Usual Language Codes

For the commonly used languages, the ISO 639-1 standard defines two-letter
codes.

`‘aa’`: Afar. `‘ab’`: Abkhazian. `‘ae’`: Avestan. `‘af’`: Afrikaans. `‘ak’`: Akan. `‘am’`: Amharic. `‘an’`: Aragonese. `‘ar’`: Arabic. `‘as’`: Assamese. `‘av’`: Avaric. `‘ay’`: Aymara. `‘az’`: Azerbaijani. `‘ba’`: Bashkir. `‘be’`: Belarusian. `‘bg’`: Bulgarian. `‘bh’`: Bihari. `‘bi’`: Bislama. `‘bm’`: Bambara. `‘bn’`: Bengali; Bangla. `‘bo’`: Tibetan. `‘br’`: Breton. `‘bs’`: Bosnian. `‘ca’`: Catalan. `‘ce’`: Chechen. `‘ch’`: Chamorro. `‘co’`: Corsican. `‘cr’`: Cree. `‘cs’`: Czech. `‘cu’`: Church Slavic. `‘cv’`: Chuvash. `‘cy’`: Welsh. `‘da’`: Danish. `‘de’`: German. `‘dv’`: Divehi; Maldivian. `‘dz’`: Dzongkha; Bhutani. `‘ee’`: 'Ew'e. `‘el’`: Greek. `‘en’`: English. `‘eo’`: Esperanto. `‘es’`: Spanish. `‘et’`: Estonian. `‘eu’`: Basque. `‘fa’`: Persian. `‘ff’`: Fulah. `‘fi’`: Finnish. `‘fj’`: Fijian; Fiji. `‘fo’`: Faroese. `‘fr’`: French. `‘fy’`: Western Frisian. `‘ga’`: Irish. `‘gd’`: Scottish Gaelic. `‘gl’`: Galician. `‘gn’`: Guarani. `‘gu’`: Gujarati. `‘gv’`: Manx. `‘ha’`: Hausa. `‘he’`: Hebrew (formerly iw). `‘hi’`: Hindi. `‘ho’`: Hiri Motu. `‘hr’`: Croatian. `‘ht’`: Haitian; Haitian Creole. `‘hu’`: Hungarian. `‘hy’`: Armenian. `‘hz’`: Herero. `‘ia’`: Interlingua. `‘id’`: Indonesian (formerly in). `‘ie’`: Interlingue; Occidental. `‘ig’`: Igbo. `‘ii’`: Sichuan Yi; Nuosu. `‘ik’`: Inupiak; Inupiaq. `‘io’`: Ido. `‘is’`: Icelandic. `‘it’`: Italian. `‘iu’`: Inuktitut. `‘ja’`: Japanese. `‘jv’`: Javanese. `‘ka’`: Georgian. `‘kg’`: Kongo. `‘ki’`: Kikuyu; Gikuyu. `‘kj’`: Kuanyama; Kwanyama. `‘kk’`: Kazakh. `‘kl’`: Kalaallisut; Greenlandic. `‘km’`: Central Khmer; Cambodian. `‘kn’`: Kannada. `‘ko’`: Korean. `‘kr’`: Kanuri. `‘ks’`: Kashmiri. `‘ku’`: Kurdish. `‘kv’`: Komi. `‘kw’`: Cornish. `‘ky’`: Kirghiz. `‘la’`: Latin. `‘lb’`: Letzeburgesch; Luxembourgish. `‘lg’`: Ganda. `‘li’`: Limburgish; Limburger; Limburgan. `‘ln’`: Lingala. `‘lo’`: Lao; Laotian. `‘lt’`: Lithuanian. `‘lu’`: Luba-Katanga. `‘lv’`: Latvian; Lettish. `‘mg’`: Malagasy. `‘mh’`: Marshallese. `‘mi’`: Maori. `‘mk’`: Macedonian. `‘ml’`: Malayalam. `‘mn’`: Mongolian. `‘mo’`: Moldavian. `‘mr’`: Marathi. `‘ms’`: Malay. `‘mt’`: Maltese. `‘my’`: Burmese. `‘na’`: Nauru. `‘nb’`: Norwegian Bokmål. `‘nd’`: Ndebele, North. `‘ne’`: Nepali. `‘ng’`: Ndonga. `‘nl’`: Dutch. `‘nn’`: Norwegian Nynorsk. `‘no’`: Norwegian. `‘nr’`: Ndebele, South. `‘nv’`: Navajo; Navaho. `‘ny’`: Chichewa; Nyanja. `‘oc’`: Occitan; Provençal. `‘oj’`: Ojibwa. `‘om’`: (Afan) Oromo. `‘or’`: Oriya. `‘os’`: Ossetian; Ossetic. `‘pa’`: Panjabi; Punjabi. `‘pi’`: Pali. `‘pl’`: Polish. `‘ps’`: Pashto; Pushto. `‘pt’`: Portuguese. `‘qu’`: Quechua. `‘rm’`: Romansh. `‘rn’`: Rundi; Kirundi. `‘ro’`: Romanian. `‘ru’`: Russian. `‘rw’`: Kinyarwanda. `‘sa’`: Sanskrit. `‘sc’`: Sardinian. `‘sd’`: Sindhi. `‘se’`: Northern Sami. `‘sg’`: Sango; Sangro. `‘si’`: Sinhala; Sinhalese. `‘sk’`: Slovak. `‘sl’`: Slovenian. `‘sm’`: Samoan. `‘sn’`: Shona. `‘so’`: Somali. `‘sq’`: Albanian. `‘sr’`: Serbian. `‘ss’`: Swati; Siswati. `‘st’`: Sesotho; Sotho, Southern. `‘su’`: Sundanese. `‘sv’`: Swedish. `‘sw’`: Swahili. `‘ta’`: Tamil. `‘te’`: Telugu. `‘tg’`: Tajik. `‘th’`: Thai. `‘ti’`: Tigrinya. `‘tk’`: Turkmen. `‘tl’`: Tagalog. `‘tn’`: Tswana; Setswana. `‘to’`: Tonga. `‘tr’`: Turkish. `‘ts’`: Tsonga. `‘tt’`: Tatar. `‘tw’`: Twi. `‘ty’`: Tahitian. `‘ug’`: Uighur. `‘uk’`: Ukrainian. `‘ur’`: Urdu. `‘uz’`: Uzbek. `‘ve’`: Venda. `‘vi’`: Vietnamese. `‘vo’`: Volapük; Volapuk. `‘wa’`: Walloon. `‘wo’`: Wolof. `‘xh’`: Xhosa. `‘yi’`: Yiddish (formerly ji). `‘yo’`: Yoruba. `‘za’`: Zhuang. `‘zh’`: Chinese. `‘zu’`: Zulu.

## A.2 Rare Language Codes

For rarely used languages, the ISO 639-2 standard defines three-letter
codes. Here is the current list, reduced to only living languages with at least
one million of speakers.

`‘ace’`: Achinese. `‘awa’`: Awadhi. `‘bal’`: Baluchi. `‘ban’`: Balinese. `‘bej’`: Beja; Bedawiyet. `‘bem’`: Bemba. `‘bho’`: Bhojpuri. `‘bik’`: Bikol. `‘bin’`: Bini; Edo. `‘bug’`: Buginese. `‘ceb’`: Cebuano. `‘din’`: Dinka. `‘doi’`: Dogri. `‘fil’`: Filipino; Pilipino. `‘fon’`: Fon. `‘gon’`: Gondi. `‘gsw’`: Swiss German; Alemannic; Alsatian. `‘hil’`: Hiligaynon. `‘hmn’`: Hmong. `‘ilo’`: Iloko. `‘kab’`: Kabyle. `‘kam’`: Kamba. `‘kbd’`: Kabardian. `‘kmb’`: Kimbundu. `‘kok’`: Konkani. `‘kru’`: Kurukh. `‘lua’`: Luba-Lulua. `‘luo’`: Luo (Kenya and Tanzania). `‘mad’`: Madurese. `‘mag’`: Magahi. `‘mai’`: Maithili. `‘mak’`: Makasar. `‘man’`: Mandingo. `‘men’`: Mende. `‘min’`: Minangkabau. `‘mni’`: Manipuri. `‘mos’`: Mossi. `‘mwr’`: Marwari. `‘nap’`: Neapolitan. `‘nso’`: Pedi; Sepedi; Northern Sotho. `‘nym’`: Nyamwezi. `‘nyn’`: Nyankole. `‘pag’`: Pangasinan. `‘pam’`: Pampanga; Kapampangan. `‘raj’`: Rajasthani. `‘sas’`: Sasak. `‘sat’`: Santali. `‘scn’`: Sicilian. `‘shn’`: Shan. `‘sid’`: Sidamo. `‘srr’`: Serer. `‘suk’`: Sukuma. `‘sus’`: Susu. `‘tem’`: Timne. `‘tiv’`: Tiv. `‘tum’`: Tumbuka. `‘umb’`: Umbundu. `‘wal’`: Walamo. `‘war’`: Waray. `‘yao’`: Yao.

---

Go to the first, previous, next, last section, table of contents.
