# Supplementary material for: rDock: A Fast, Versatile and Open Source Program for Docking Ligands to Proteins and Nucleic Acids
Source: PLoS Comput Biol. 2014 Apr 10;10(4):e1003571. doi: 10.1371/journal.pcbi.1003571 (PMC3983074; doi:10.1371/journal.pcbi.1003571)
Supplement: Software S1 — Compressed file with the source code of the rDock software for ligand docking to Proteins and Nucleic Acids. (GZ) [file pcbi.1003571.s010.gz › rDock_2013.1_src/gettext-0.18.tar.gz!/gettext-0.18/gettext-tools/doc/gettext_2.html]

GNU gettext utilities - 2 The User's View

Go to the first, previous, next, last section, table of contents.

---

# 2 The User's View

Nowadays, when users log into a computer, they usually find that all
their programs show messages in their native language -- at least for
users of languages with an active free software community, like French or
German; to a lesser extent for languages with a smaller participation in
free software and the GNU project, like Hindi and Filipino.

How does this work? How can the user influence the language that is used
by the programs? This chapter will answer it.

## 2.1 Operating System Installation

The default language is often already specified during operating system
installation. When the operating system is installed, the installer
typically asks for the language used for the installation process and,
separately, for the language to use in the installed system. Some OS
installers only ask for the language once.

This determines the system-wide default language for all users. But the
installers often give the possibility to install extra localizations for
additional languages. For example, the localizations of KDE (the K
Desktop Environment) and OpenOffice.org are often bundled separately,
as one installable package per language.

At this point it is good to consider the intended use of the machine: If
it is a machine designated for personal use, additional localizations are
probably not necessary. If, however, the machine is in use in an
organization or company that has international relationships, one can
consider the needs of guest users. If you have a guest from abroad, for
a week, what could be his preferred locales? It may be worth installing
these additional localizations ahead of time, since they cost only a bit
of disk space at this point.

The system-wide default language is the locale configuration that is used
when a new user account is created. But the user can have his own locale
configuration that is different from the one of the other users of the
same machine. He can specify it, typically after the first login, as
described in the next section.

## 2.2 Setting the Locale Used by GUI Programs

The immediately available programs in a user's desktop come from a group
of programs called a “desktop environment”; it usually includes the window
manager, a web browser, a text editor, and more. The most common free
desktop environments are KDE, GNOME, and Xfce.

The locale used by GUI programs of the desktop environment can be specified
in a configuration screen called “control center”, “language settings”
or “country settings”.

Individual GUI programs that are not part of the desktop environment can
have their locale specified either in a settings panel, or through environment
variables.

For some programs, it is possible to specify the locale through environment
variables, possibly even to a different locale than the desktop's locale.
This means, instead of starting a program through a menu or from the file
system, you can start it from the command-line, after having set some
environment variables. The environment variables can be those specified
in the next section (section 2.3 Setting the Locale through Environment Variables); for some versions of
KDE, however, the locale is specified through a variable `KDE_LANG`,
rather than `LANG` or `LC_ALL`.

## 2.3 Setting the Locale through Environment Variables

As a user, if your language has been installed for this package, in the
simplest case, you only have to set the `LANG` environment variable
to the appropriate `‘ll_CC’` combination. For example,
let's suppose that you speak German and live in Germany. At the shell
prompt, merely execute
`‘setenv LANG de_DE’` (in `csh`),
`‘export LANG; LANG=de_DE’` (in `sh`) or
`‘export LANG=de_DE’` (in `bash`). This can be done from your
‘.login’ or ‘.profile’ file, once and for all.

### 2.3.1 Locale Names

A locale name usually has the form `‘ll_CC’`. Here
`‘ll’` is an ISO 639 two-letter language code, and
`‘CC’` is an ISO 3166 two-letter country code. For example,
for German in Germany, ll is `de`, and CC is `DE`.
You find a list of the language codes in appendix section A Language Codes and
a list of the country codes in appendix section B Country Codes.

You might think that the country code specification is redundant. But in
fact, some languages have dialects in different countries. For example,
`‘de_AT’` is used for Austria, and `‘pt_BR’` for Brazil. The country
code serves to distinguish the dialects.

Many locale names have an extended syntax
`‘ll_CC.encoding’` that also specifies the character
encoding. These are in use because between 2000 and 2005, most users have
switched to locales in UTF-8 encoding. For example, the German locale on
glibc systems is nowadays `‘de_DE.UTF-8’`. The older name `‘de_DE’`
still refers to the German locale as of 2000 that stores characters in
ISO-8859-1 encoding -- a text encoding that cannot even accomodate the Euro
currency sign.

Some locale names use `‘ll_CC.@variant’` instead of
`‘ll_CC’`. The `‘@variant’` can denote any kind of
characteristics that is not already implied by the language ll and
the country CC. It can denote a particular monetary unit. For example,
on glibc systems, `‘de_DE@euro’` denotes the locale that uses the Euro
currency, in contrast to the older locale `‘de_DE’` which implies the use
of the currency before 2002. It can also denote a dialect of the language,
or the script used to write text (for example, `‘sr_RS@latin’` uses the
Latin script, whereas `‘sr_RS’` uses the Cyrillic script to write Serbian),
or the orthography rules, or similar.

On other systems, some variations of this scheme are used, such as
`‘ll’`. You can get the list of locales supported by your system
for your language by running the command `‘locale -a | grep '^ll'’`.

There is also a special locale, called `‘C’`.
When it is used, it disables all localization: in this locale, all programs
standardized by POSIX use English messages and an unspecified character
encoding (often US-ASCII, but sometimes also ISO-8859-1 or UTF-8, depending on
the operating system).

### 2.3.2 Locale Environment Variables

A locale is composed of several *locale categories*, see section 1.3 Aspects in Native Language Support.
When a program looks up locale dependent values, it does this according to
the following environment variables, in priority order:

1. `LANGUAGE`

   - `LC_ALL`


     - `LC_xxx`, according to selected locale category:
       `LC_CTYPE`, `LC_NUMERIC`, `LC_TIME`, `LC_COLLATE`,
       `LC_MONETARY`, `LC_MESSAGES`, ...
       - `LANG`

Variables whose value is set but is empty are ignored in this lookup.

`LANG` is the normal environment variable for specifying a locale.
As a user, you normally set this variable (unless some of the other variables
have already been set by the system, in ‘/etc/profile’ or similar
initialization files).

`LC_CTYPE`, `LC_NUMERIC`, `LC_TIME`, `LC_COLLATE`,
`LC_MONETARY`, `LC_MESSAGES`, and so on, are the environment
variables meant to override `LANG` and affecting a single locale
category only. For example, assume you are a Swedish user in Spain, and you
want your programs to handle numbers and dates according to Spanish
conventions, and only the messages should be in Swedish. Then you could
create a locale named `‘sv_ES’` or `‘sv_ES.UTF-8’` by use of the
`localedef` program. But it is simpler, and achieves the same effect,
to set the `LANG` variable to `es_ES.UTF-8` and the
`LC_MESSAGES` variable to `sv_SE.UTF-8`; these two locales come
already preinstalled with the operating system.

`LC_ALL` is an environment variable that overrides all of these.
It is typically used in scripts that run particular programs. For example,
`configure` scripts generated by GNU autoconf use `LC_ALL` to make
sure that the configuration tests don't operate in locale dependent ways.

Some systems, unfortunately, set `LC_ALL` in ‘/etc/profile’ or in
similar initialization files. As a user, you therefore have to unset this
variable if you want to set `LANG` and optionally some of the other
`LC_xxx` variables.

The `LANGUAGE` variable is described in the next subsection.

### 2.3.3 Specifying a Priority List of Languages

Not all programs have translations for all languages. By default, an
English message is shown in place of a nonexistent translation. If you
understand other languages, you can set up a priority list of languages.
This is done through a different environment variable, called
`LANGUAGE`. GNU `gettext` gives preference to `LANGUAGE`
over `LC_ALL` and `LANG` for the purpose of message handling,
but you still need to have `LANG` (or `LC_ALL`) set to the primary
language; this is required by other parts of the system libraries.
For example, some Swedish users who would rather read translations in
German than English for when Swedish is not available, set `LANGUAGE`
to `‘sv:de’` while leaving `LANG` to `‘sv_SE’`.

Special advice for Norwegian users: The language code for Norwegian
bokm@ringaccent{a}l changed from `‘no’` to `‘nb’` recently (in 2003).
During the transition period, while some message catalogs for this language
are installed under `‘nb’` and some older ones under `‘no’`, it is
recommended for Norwegian users to set `LANGUAGE` to `‘nb:no’` so that
both newer and older translations are used.

In the `LANGUAGE` environment variable, but not in the other
environment variables, `‘ll_CC’` combinations can be
abbreviated as `‘ll’` to denote the language's main dialect.
For example, `‘de’` is equivalent to `‘de_DE’` (German as spoken in
Germany), and `‘pt’` to `‘pt_PT’` (Portuguese as spoken in Portugal)
in this context.

Note: The variable `LANGUAGE` is ignored if the locale is set to
`‘C’`. In other words, you have to first enable localization, by setting
`LANG` (or `LC_ALL`) to a value other than `‘C’`, before you can
use a language priority list through the `LANGUAGE` variable.

## 2.4 Installing Translations for Particular Programs

Languages are not equally well supported in all packages using GNU
`gettext`, and more translations are added over time. Usually, you
use the translations that are shipped with the operating system
or with particular packages that you install afterwards. But you can also
install newer localizations directly. For doing this, you will need an
understanding where each localization file is stored on the file system.

For programs that participate in the Translation Project, you can start
looking for translations here:
http://translationproject.org/team/index.html.
A snapshot of this information is also found in the ‘ABOUT-NLS’ file
that is shipped with GNU gettext.

For programs that are part of the KDE project, the starting point is:
http://i18n.kde.org/.

For programs that are part of the GNOME project, the starting point is:
http://www.gnome.org/i18n/.

For other programs, you may check whether the program's source code package
contains some ‘ll.po’ files; often they are kept together in a
directory called ‘po/’. Each ‘ll.po’ file contains the
message translations for the language whose abbreviation of ll.

---

Go to the first, previous, next, last section, table of contents.
