# Supplementary material for: rDock: A Fast, Versatile and Open Source Program for Docking Ligands to Proteins and Nucleic Acids
Source: PLoS Comput Biol. 2014 Apr 10;10(4):e1003571. doi: 10.1371/journal.pcbi.1003571 (PMC3983074; doi:10.1371/journal.pcbi.1003571)
Supplement: Software S1 — Compressed file with the source code of the rDock software for ligand docking to Proteins and Nucleic Acids. (GZ) [file pcbi.1003571.s010.gz › rDock_2013.1_src/gettext-0.18.tar.gz!/gettext-0.18/gettext-tools/doc/FAQ.html]

GNU gettext FAQ


# Frequently Asked Questions for GNU gettext

# Questions

### General

- Where is the mailing list?
- Where is the newest gettext source?
- I want to be notified of new gettext
  releases.

### Problems building GNU gettext

- On Solaris, I get a build
  error “text relocations remain” in the libasprintf subdirectory
- “make install” fails

### Problems integrating GNU gettext

- How do I make use of gettext() in my package?
- I get a linker error “undefined
  reference to libintl\_gettext”
- gettextize adds multiple
  references to the same directories/files
  to Makefile.am and configure.ac
- My program compiles and links fine,
  but doesn't output translated
  strings.

### GNU gettext on Windows

- What does Woe32 mean?
- How do I compile, link and run a program
  that uses the gettext()
  function?
- Setting the LANG
  environment variable doesn't have any effect

### Other

- What does this mean: “`msgid' and `msgstr'
  entries do not both
  end with '\n'”
- German umlauts are displayed like “ge"andert”
  instead of
  “geändert”
- The LANGUAGE
  environment variable is ignored after I set LANG=en
- I use accented characters in my
  source code. How do I tell the
  C/C++ compiler in which encoding it is (like xgettext's --from-code option)?

# Answers

### General

#### Where is the mailing list?

Three mailing lists are available:   

- bug-gnu-gettext@gnu.org  
  This mailing list is for discussion of features and bugs of the GNU
  gettext software, including
  libintl, the gettext-tools, and its autoconf macros.
- translation-i18n@lists.sourceforge.net  
  This mailing list is for methodology questions around
  internationalization, and for discussions of translator tools,
  including but not limited to GNU gettext.
- coordinator@translationproject.org  
  This is the email address of the Free Translation Project,
  that is the project which manages the translated message
  catalogs for many free software packages. Note that KDE and GNOME
  packages are not part of this project; they have their own translation
  projects: i18n.kde.org and gtp.

The bug-gnu-gettext list
is archived as part of the bug-gnu-utils archives. bug-gnu-gettext cannot be
subscribed on its own; to receive its contents by mail, subscribe to bug-gnu-utils.  

#### Where is the newest gettext source?

The newest gettext release is available on ftp.gnu.org and its mirrors, in
http://ftp.gnu.org/gnu/gettext/.  
  
Prereleases are announced on the autotools-announce mailing list.
Note that prereleases are meant for testing and not meant for use in
production environments. Please don't use the “gettextize” program of a
prerelease on projects which you share with other programmers via CVS.  
  
If you want to live on the bleeding edge, you can also use the
development sources. Instructions for retrieving the gettext CVS are
found here.
Note that building from CVS requires special tools (autoconf, automake,
m4, groff, bison, etc.) and requires that you pay attention to the README-alpha and autogen.sh files in the CVS.  

#### I want to be notified of new gettext releases.

If you are interested in stable gettext releases, you can follow the info-gnu mailing list. It
is also available as a newsgroup gmane.org.fsf.announce
through gmane.org.  
  
You can also periodically check the download location.  
  
If you are interested in testing prereleases as well, you can subscribe
to the autotools-announce mailing
list.  

### Problems building GNU gettext

#### On Solaris, I get a build error “text relocations remain” in the libasprintf subdirectory

libtool (or more precisely, the version of libtool that was available
at the time the gettext release waas made) doesn't support linking C++
libraries with some versions of GCC. As a workaround, you can configure
gettext with the option --disable-libasprintf.  

#### “make install” fails

“make install DESTDIR=/some/tempdir” can fail with
an error message relating to libgettextlib
or libgettextsrc, or can
silently fail to install libgettextsrc.
On some platforms, this is due to limitations of libtool regarding DESTDIR. On other platforms, it
is due to the way the system handles shared libraries, and libtool
cannot work around it. Fortunately, on Linux and other glibc based
systems, DESTDIR is
supported if no different version of gettext is already installed (i.e.
it works if you uninstall the older gettext before building and
installing the newer one, or if you do a plain “make install” before “make install DESTDIR=/some/tempdir”). On other
systems, when  DESTDIR
does not work, you can still do “make
install” and copy the installed files to /some/tempdir
afterwards.  
  
If “make install” without DESTDIR fails, it's a bug which
you are welcome to report to the usual bug report address.

### Problems integrating GNU gettext

#### How do I make use of gettext() in my package?

It's not as difficult as it sounds. Here's the recipe for C or C++
based packages.  

- Add an invocation of AM\_GNU\_GETTEXT([external])
  to the package's configure.{ac,in}
  file.
- Invoke “gettextize --copy”.
  It will do most of the autoconf/automake related work for you.
- Add the gettext.h
  file to the package's source directory, and include it in all source
  files that contain translatable strings or do output via printf or fprintf.
- In the source file defining the main() function of the program,
  add these lines to the header  

  `#include <locale.h>  
  #include "gettext.h"`

  and these lines near the beginning of the main() function:  

  `setlocale (LC_ALL, "");  
  bindtextdomain (PACKAGE,
  LOCALEDIR);  
  textdomain (PACKAGE);`
- Mark all strings that should be translated with \_(), like this: \_("No errors found."). While
  doing this, try to turn the strings into good English, one entire
  sentence per string, not more than one paragraph per string, and use
  format strings instead of string concatenation. This is needed so that
  the translators can provide accurate translations.
- In every source file containing translatable strings, add these lines
  to the header:  

  `#include "gettext.h"  
  #define _(string) gettext (string)`
- In the freshly created po/
  directory, set up the POTFILES.in
  file, and do a “make update-po”.
  Then distribute the generated .pot
  file to your nearest translation project.
- Shortly before a release, integrate the translators' .po files into the po/ directory and do “make update-po” again.

You find detailed descriptions of how this all works in the GNU gettext
manual, chapters “The Maintainer's View” and “Preparing Program
Sources”.

#### I get a linker error “undefined reference to libintl\_gettext”

This error means that the program uses the gettext() function after having
included the <libintl.h>
file from GNU gettext (which remaps it to libintl\_gettext()), however at
link time a function of this name could not be linked in. (It is
expected to come from the libintl
library, installed by GNU gettext.)  
  
There are many possible reasons for this error, but in any case you
should consider the -I, -L and -l options passed to the
compiler. In packages using autoconf
generated configure scripts, -I
options come from the CFLAGS
and CPPFLAGS variables
(in Makefiles also DEFS
and INCLUDES), -L options come from the LDFLAGS variable, and -l options come from the LIBS variable. The first thing
you should check are the values of these variables in your environment
and in the  package's config.status
autoconfiguration result.  
  
To find the cause of the error, a little analysis is needed. Does the
program's final link command contains the option “-lintl”?  

- If yes:  
  Find out where the libintl
  comes from. To do this, you have to check for libintl.a and libintl.so\* (libintl.dylib on MacOS X) in
  each directory given as a -L option, as well as in the compiler's
  implicit search directories. (You get these implicit search directories
  for gcc by using “gcc -v”
  instead of “gcc” in the
  final link command line; compilers other than GCC usually look in /usr/lib and /lib.) A shell command like  

  `$ for d in /usr/local/lib
  /usr/lib /lib; do ls -l $d/libintl.*; done`

  will show where the libintl
  comes from. By looking at the dates and whether each library defines libintl\_gettext (via “nm path/libintl.so
  | grep libintl\_gettext”) you can now distinguish three possible
  causes of the error:  
  - Some older libintl is used instead of the newer one. The fix
    is to remove the old library or to reorganize your -L options.
  - The used libintl is the new one, and it doesn't contain
    libintl\_gettext. This would be a bug in gettext. If this is the case,
    please report it to the usual bug report address.
  - The used libintl is a static library (libintl.a), there are
    no uses of gettext in .o files before the “-lintl” but there are some
    after the “-lintl”. In this case the fix is to move the “-lintl” to the
    end or near the end of the link command line. The only libintl
    dependency that needs to be mentioned after “-lintl” is “-liconv”.
- If no:  
  In this case it's likely a bug in the package you are building: The
  package's Makefiles should make sure that “-lintl” is used where needed.  
  Test whether libintl was found by configure. You can check this by doing  

  `$ grep
  '\(INTLLIBS\|LIBINTL\)' config.status`

  and looking whether the value of this autoconf variable is non-empty.  
  - If yes: It should be the responsibility of the Makefile to
    use the value of this variable in the link command line. Does the
    Makefile.in rule for linking the program use @INTLLIBS@ or @LIBINTL@?  
    - If no: It's a Makefile.am/in bug.
    - If yes: Something strange is going on. You need to dig
      deeper.
    Note that @INTLLIBS@ is
    for gettext.m4 versions
    <= 0.10.40 and @LIBINTL@
    is for gettext.m4
    versions >= 0.11, depending on which gettext.m4 was used to build
    the package's configure -
    regardless of which gettext you have now installed.
  - If no: So libintl was not found.  
    Take a look at the package's configure.in/ac.
    Does it invoke AM\_GNU\_GETTEXT?  
    - If no: The gettext maintainers take no responsibilities for
      lookalikes named CY\_GNU\_GETTEXT, AM\_GLIB\_GNU\_GETTEXT, AM\_GNOME\_GETTEXT
      and similar, or for homebrewn autoconf checks. Complain to the package
      maintainer.
    - If yes: It looks like the -I and -L options were inconsistent.
      You should have a -Isomedir/include in the CFLAGS or CPPFLAGS if and only if you
      also have a -Lsomedir/lib in the LDFLAGS. And somedir/include
      should contain a libintl.h
      if and only if somedir/lib contains libintl.{a,so}.  
      This case can also happen if you have configured a GCC < 3.2 with
      the same --prefix option
      as you used for GNU libiconv or GNU gettext. This is fatal, because
      these versions of GCC implicitly use -Lprefix/lib
      but not  
      -Iprefix/include. The
      workaround is to use a different --prefix
      for GCC.

#### gettextize adds multiple references to the same directories/files to Makefile.am and configure.ac

If gettextize is used on
a package, then the po/, intl/, m4/ directories of the package
are removed, and then gettextize
is invoked on the package again, it will re-add the po/, intl/, m4/ directories and change Makefile.am, configure.ac and ChangeLog accordingly. This is
normal. The second use of gettextize
here is an abuse of the program. gettextize
is a wizard intended to transform a working
source package into a working
source package that uses the newest version of gettext. If you
start out from a nonfunctional source package (it is nonfunctional
since you have omitted some directories), you cannot expect that gettextize corrects it.  
  
Often this question arises in packages that use CVS. See the section
“CVS Issues / Integrating with CVS” of the GNU gettext documentation.
This section mentions a program autopoint
which is designed to reconstruct those files and directories created by
gettextize that can be
omitted from a CVS repository.  

#### My program compiles and links fine, but doesn't output translated strings.

There are several possible reasons. Here is a checklist that allows you
to determine the cause.  

1. Check that the environment variables LC\_ALL, LC\_MESSAGES,
   LC\_CTYPE, LANG, LANGUAGE together specify a valid locale and language.  
   To check this, run the commands  

   `$ gettext --version`  
   `$ gettext --help`

   You should see at least some output in your desired language. If not,
   either  
   - You have chosen a too exotic language. gettext is localized to 33
     languages. Choose a less exotic language, such as Galician or
     Ukrainian. Or
   - There is a problem with your environment variables. Possibly
     LC\_ALL points to a locale that is not installed, or LC\_MESSAGES and
     LC\_CTYPE are inconsistent.
2. Check that your program contains a setlocale call.  
   To check this, run your program under ltrace. For example,  

   `$ ltrace ./myprog`  
   `...`  
   `setlocale(6,
   "")                 
   = "de_DE.UTF-8"`

   If you have no ltrace, you can also do this check by running your
   program under the debugger. For example,  

   `$ gdb ./myprog`  
   `(gdb) break main`  
   `(gdb) run`  
   `Breakpoint 1, main ()`  
   `(gdb) break setlocale`  
   `(gdb) continue`  
   `Breakpoint 2, setlocale ()`  
   `;; OK, the breakpoint has been hit, setlocale() is being
   called.`

   Either way, check that the return value of setlocale() is non-NULL. A NULL
   return value indicates a failure.
3. Check that your program contains a textdomain call, a bindtextdomain call referring
   to the same message domain, and then really calls the gettext, dgettext or dcgettext function.  
   To check this, run the program under ltrace. For example,  

   `$ ltrace ./myprog`  
   `...`  
   `textdomain("hello-c")                            
   = "hello-c"`  
   `bindtextdomain("hello-c", "/opt/share"...) = "/opt/share"...`  
   `dcgettext(0, 0x08048691, 5, 0x0804a200, 0x08048689) =
   0x4001721f`

   If you have no ltrace, you can also do this check by running your
   program under the debugger. For example,  

   `$ gdb ./myprog`  
   `(gdb) break main`  
   `(gdb) run`  
   `Breakpoint 1, main ()`  
   `(gdb) break textdomain`  
   `(gdb) break bindtextdomain`  
   `(gdb) break gettext`  
   `(gdb) break dgettext`  
   `(gdb) break dcgettext`  
   `(gdb) continue`  
   `Breakpoint 2, textdomain ()`  
   `(gdb) continue`  
   `Breakpoint 3, bindtextdomain ()`  
   `(gdb) continue`  
   `Breakpoint 6, dcgettext ()`

   Note that here dcgettext()
   is called instead of the gettext()
   function mentioned in the source code; this is due to an optimization
   in <libintl.h>.  
   When using libintl on a non-glibc system, you have to add a prefix “libintl\_” to all the function
   names mentioned here, because that's what the functions are really
   named, under the hood.  
   If gettext/dgettext/dcgettext is not called at all,
   the possible cause might be that some autoconf or Makefile macrology
   has turned off internationalization entirely (like the --disable-nls configuration
   option usually does).
4. Check that the .mo
   file that contains the translation is really there where the program
   expects it.  
   To check this, run the program under strace and look at the open() calls. For example,  

   `$ strace ./myprog 2>&1
   | grep '^open('`  
   `open("/etc/ld.so.preload", O_RDONLY)    = -1
   ENOENT (No such file or directory)`  
   `open("/etc/ld.so.cache",
   O_RDONLY)      = 5`  
   `open("/lib/libc.so.6",
   O_RDONLY)        = 5`  
   `open("/usr/lib/locale/locale-archive", O_RDONLY|O_LARGEFILE)
   = 5`  
   `open("/usr/share/locale/locale.alias", O_RDONLY) = 5`  
   `open("/opt/share/locale/de/LC_MESSAGES/hello-c.mo", O_RDONLY)
   = 5`  
   `...`

   A nonnegative open()
   return value means that the file has been found.  
   If you have no strace, you can also guess the .mo file's location: it is  

   localedir/lang/LC\_MESSAGES/domain.mo

   where domain is the argument
   passed to textdomain(), localedir is the second argument
   passed to bindtextdomain(),
   and lang is the language (LL) or language and territory (LL\_CC),
   depending on the environment variables checked in step 1.
5. Check that the .mo file contains a translation for the string
   that is being asked for.  
   To do this, you need to convert the .mo file back to PO file format,
   through the command  

   `$ msgunfmt` localedir/lang/LC\_MESSAGES/domain.mo

   and look for an msgid
   that matches the given string.

### GNU gettext on Windows

#### What does Woe32 mean?

“Woe32” denotes the Windows 32-bit operating systems for x86: Windows
NT/2000/XP/Vista and Windows 95/98/ME. Microsoft uses the term “Win32” to
denote these; this is a psychological trick in order to make everyone
believe that these OSes are a “win” for the user. However, for most
users and developers, they are a source of woes, which is why I call
them “Woe32”.  

#### How do I compile, link and run a program that uses the gettext() function?

When you use RedHat's cygwin environment, it's as on Unix:  

- You need to add an -I
  option to the compilation command line, so that the compiler finds the libintl.h include file, and
- You need to add an -L
  option to the link command line, so that the linker finds the libintl library.

When you use the Mingw environment (either from within cygwin, with CC="gcc -mno-cygwin", or from
MSYS, with CC="gcc"), I
don't know the details.  
  
When you use the Microsoft Visual C/C++ (MSVC) compiler, you will
likely use the precompiled Woe32 binaries. For running a program that
uses gettext(), one needs the .bin.woe32.zip
packages of gettext-runtime
and libiconv. As a
developer, you'll also need the xgettext
and msgfmt programs that
are contained in the .bin.woe32.zip
package of gettext-tools.
Then  

- You need to add an -MD
  option to all compilation and link command lines. MSVC has six
  different, mutually incompatible, compilation models (-ML, -MT, -MD, -MLd, -MTd, -MDd); the default is -ML. intl.dll uses the -MD model, therefore the rest
  of the program must use -MD
  as well.
- You need to add an -I
  option to the compilation command line, so that the compiler finds the libintl.h include file.
- You need to add an -L
  option to the link command line, so that the linker finds the intl.lib library.
- You need to copy the intl.dll
  and iconv.dll to the
  directory where your .exe
  files are created, so that they will be found at runtime.

#### Setting the LANG environment variable doesn't have any effect

If neither LC\_ALL, LC\_MESSAGES nor LANGUAGES is set, it's the LANG
environment variable which determines the language into which gettext()
translates the messages.  
  
You can test your program by setting the LANG environment variable from
outside the program. In a Windows command interpreter:  

`set LANG=de_DE`  
`.\myprog.exe`

Or in a Cygwin shell:  

`$ env LANG=de_DE ./myprog.exe`

  
If this test fails, look at the question “My program compiles and links
fine, but doesn't output translated
strings.” above.  
  
If this test succeeds, the problem is related in the way you set the
environment variable. Here is a checklist:  

- Check that you are using the -MD option in all compilation
  and link command lines. Otherwise you might end up calling the putenv() function from
  Microsoft's libc.lib,
  whereas intl.dll is using
  the getenv() function
  from Mictosoft's msvcrt.lib.
- Check that you set the environment variable using both SetEnvironmentVariable() and putenv(). A convenient way to
  do so, and to deal with the fact that some Unix systems have setenv() and some don't, is the
  following function.  
    

  `#include <string.h>`  
  `#include <stdlib.h>`  
  `#if defined _WIN32`  
  `# include <windows.h>`  
  `#endif`  
    
  `int my_setenv (const char * name, const char * value) {`  
    `size_t namelen = strlen(name);`  
    `size_t valuelen = (value==NULL ? 0 : strlen(value));`  
  `#if defined _WIN32`  
    `/* On Woe32, each process has two copies of the
  environment variables,`  
       `one managed by the OS and one
  managed by the C library. We set`  
       `the value in both locations, so that
  other software that looks in`  
       `one place or the other is guaranteed
  to see the value. Even if it's`  
       `a bit slow. See also`  
       `<http://article.gmane.org/gmane.comp.gnu.mingw.user/8272>`  
       `<http://article.gmane.org/gmane.comp.gnu.mingw.user/8273>`  
       `<http://www.cygwin.com/ml/cygwin/1999-04/msg00478.html>
  */`  
    `if (!SetEnvironmentVariableA(name,value))`  
      `return -1;`   
  `#endif`  
  `#if defined(HAVE_PUTENV)`  
    `char* buffer = (char*)malloc(namelen+1+valuelen+1);`  
    `if (!buffer)`  
      `return -1; /* no need to set errno =
  ENOMEM */`  
    `memcpy(buffer,name,namelen);`  
    `if (value != NULL) {`  
      `buffer[namelen] = '=';`  
      `memcpy(buffer+namelen+1,value,valuelen);`  
      `buffer[namelen+1+valuelen] = 0;`  
    `} else`  
      `buffer[namelen] = 0;`  
    `return putenv(buffer);`  
  `#elif defined(HAVE_SETENV)`  
    `return setenv(name,value,1);`  
  `#else`  
    `/* Uh oh, neither putenv() nor setenv() ... */`  
    `return -1;`  
  `#endif`  
  `}`

### Other

#### What does this mean: “`msgid' and `msgstr' entries do not both end with '\n'”

It means that when the original string ends in a newline, your
translation must also end in a newline. And if the original string does
not end in a newline, then your translation should likewise not have a
newline at the end.  

#### German umlauts are displayed like “ge"andert” instead of “geändert”

This symptom occurs when the LC\_CTYPE
facet of the locale is not set; then gettext() doesn't know which
character set to use, and converts all messages to ASCII, as far as
possible.  
  
If the program is doing  
 `setlocale (LC_MESSAGES, "");` then change it to  
 `setlocale (LC_CTYPE, "");  
setlocale (LC_MESSAGES, "");`   
or do both of these in a single call:  
 `setlocale (LC_ALL, "");`   
If the program is already doing  
 `setlocale (LC_ALL, "");`   
then the symptom can still occur if the user has not set LANG, but instead has set LC\_MESSAGES to a valid locale
and has set LC\_CTYPE to
nothing or an invalid locale. The fix for the user is then to set LANG instead of LC\_MESSAGES.  

#### The LANGUAGE environment variable is ignored after I set LANG=en

This is because “en” is a language name, but not a valid locale name.
The ABOUT-NLS  file
says:  
> In the LANGUAGE
> environment variable, but not in the LANG environment variable, LL\_CC combinations can be
> abbreviated as LL to
> denote the language's main dialect.

Why is LANG=en not
allowed? Because LANG is
a setting for the entire locale, including monetary information, and
this depends on the country: en\_GB, en\_AU, en\_ZA all have different
currencies.  

#### I use accented characters in my source code. How do I tell the C/C++ compiler in which encoding it is (like xgettext's --from-code option)?

Short answer: If you want your program to be useful to other people,
then don't use accented characters
(or other non-ASCII characters) in string literals in the source code. Instead, use
only ASCII for string literals, and use gettext() to retrieve their
display-ready form.  
  
Long explanation:  
The reason is that the ISO C standard specifies that the character set
at compilation time can be different from the character set at
execution time.  
The character encoding at compilation time is the one which determines
how the source files are interpreted and also how string literals are
stored in the compiled code. This character encoding is generally
unspecified; for recent versions of GCC, it depends on the LC\_CTYPE
locale in effect during the compilation process.  
The character encoding at execution time is the one which determines
how standard functions like isprint(),
wcwidth() etc. work and
how strings written to standard output should be encoded. This
character encoding is specified by POSIX to depend on the LC\_CTYPE
locale in effect when the program is executed; see also the description
in the ABOUT-NLS file.  
Strings in the compiled code are not magically converted between the
time the program is compiled and the time it is run.  
  
Therefore what could you do to get accented characters to work?  
  
Can you ensure that the execution character set is the same as the
compilation character set? Even if your program is to be used only in a
single country, this is not realistically possible. For example, in
Germany there are currently three character encodings in use: UTF-8,
ISO-8859-15 and ISO-8859-1. Therefore you would have to explicitly
convert the accented strings from the compilation character set to the
execution character set at runtime, for example through iconv().  
  
Can you ensure that the compilation character set is the one in which
your source files are stored? This is not realistically possible
either: For compilers other than GCC, there is no way to specify the
compilation character set. So let's assume for a moment that everyone
uses GCC; then you will specify the LC\_CTYPE or LC\_ALL environment
variable in the Makefile. But for this you have to assume that everyone
has a locale in a given encoding. Be it UTF-8 or ISO-8859-1 - this is
not realistic. People often have no locale installed besides the one
they use.  
  
Use of wide strings L"..."
doesn't help solving the problem, because on systems like FreeBSD or
Solaris, the way how wide string literals are stored in compiled code
depends on the compilation  character set, just as it does for
narrow strings "...".
Moreover, wide strings have problems of their own.  
  
Use of ISO C 99 Unicode escapes "\uxxxx"
doesn't help either because these characters are converted to the
compilation character set at compile time; so again, since you can't
guarantee that the compilation character set is not ASCII, you're
risking compilation errors just as if the real character had been used
in the source instead of the Unicode escape.  
  
So, in summary, there is no way to make accented characters in string
literals work in C/C++.  
  
You might then wonder what xgettext's
--from-code option is good
for. The answer is  

1. For the comments in C/C++ source code. The compiler ignores them.
2. For other programming languages like Java, for which the compiler
   converts all string literals to UTF-8.

  


---

GNU gettext FAQ  
Bruno Haible <bruno@clisp.org>

Last modified: 24 February 2004
