# Supplementary material for: rDock: A Fast, Versatile and Open Source Program for Docking Ligands to Proteins and Nucleic Acids
Source: PLoS Comput Biol. 2014 Apr 10;10(4):e1003571. doi: 10.1371/journal.pcbi.1003571 (PMC3983074; doi:10.1371/journal.pcbi.1003571)
Supplement: Software S1 — Compressed file with the source code of the rDock software for ligand docking to Proteins and Nucleic Acids. (GZ) [file pcbi.1003571.s010.gz › rDock_2013.1_src/gettext-0.18.tar.gz!/gettext-0.18/gettext-tools/doc/gettext_8.html]

GNU gettext utilities - 8 Editing PO Files

Go to the first, previous, next, last section, table of contents.

---

# 8 Editing PO Files

## 8.1 KDE's PO File Editor

## 8.2 GNOME's PO File Editor

## 8.3 Emacs's PO File Editor

For those of you being
the lucky users of Emacs, PO mode has been specifically created
for providing a cozy environment for editing or modifying PO files.
While editing a PO file, PO mode allows for the easy browsing of
auxiliary and compendium PO files, as well as for following references into
the set of C program sources from which PO files have been derived.
It has a few special features, among which are the interactive marking
of program strings as translatable, and the validation of PO files
with easy repositioning to PO file lines showing errors.

For the beginning, besides main PO mode commands
(see section 8.3.2 Main PO mode Commands), you should know how to move between entries
(see section 8.3.3 Entry Positioning), and how to handle untranslated entries
(see section 8.3.7 Untranslated Entries).

### 8.3.1 Completing GNU `gettext` Installation

Once you have received, unpacked, configured and compiled the GNU
`gettext` distribution, the `‘make install’` command puts in
place the programs `xgettext`, `msgfmt`, `gettext`, and
`msgmerge`, as well as their available message catalogs. To
top off a comfortable installation, you might also want to make the
PO mode available to your Emacs users.

During the installation of the PO mode, you might want to modify your
file ‘.emacs’, once and for all, so it contains a few lines looking
like:

```
(setq auto-mode-alist
      (cons '("\\.po\\'\\|\\.po\\." . po-mode) auto-mode-alist))
(autoload 'po-mode "po-mode" "Major mode for translators to edit PO files" t)
```

Later, whenever you edit some ‘.po’
file, or any file having the string `‘.po.’` within its name,
Emacs loads ‘po-mode.elc’ (or ‘po-mode.el’) as needed, and
automatically activates PO mode commands for the associated buffer.
The string *PO* appears in the mode line for any buffer for
which PO mode is active. Many PO files may be active at once in a
single Emacs session.

If you are using Emacs version 20 or newer, and have already installed
the appropriate international fonts on your system, you may also tell
Emacs how to determine automatically the coding system of every PO file.
This will often (but not always) cause the necessary fonts to be loaded
and used for displaying the translations on your Emacs screen. For this
to happen, add the lines:

```
(modify-coding-system-alist 'file "\\.po\\'\\|\\.po\\."
                            'po-find-file-coding-system)
(autoload 'po-find-file-coding-system "po-mode")
```

to your ‘.emacs’ file. If, with this, you still see boxes instead
of international characters, try a different font set (via Shift Mouse
button 1).

### 8.3.2 Main PO mode Commands

After setting up Emacs with something similar to the lines in
section 8.3.1 Completing GNU `gettext` Installation, PO mode is activated for a window when Emacs finds a
PO file in that window. This puts the window read-only and establishes a
po-mode-map, which is a genuine Emacs mode, in a way that is not derived
from text mode in any way. Functions found on `po-mode-hook`,
if any, will be executed.

When PO mode is active in a window, the letters `‘PO’` appear
in the mode line for that window. The mode line also displays how
many entries of each kind are held in the PO file. For example,
the string `‘132t+3f+10u+2o’` would tell the translator that the
PO mode contains 132 translated entries (see section 8.3.5 Translated Entries,
3 fuzzy entries (see section 8.3.6 Fuzzy Entries), 10 untranslated entries
(see section 8.3.7 Untranslated Entries) and 2 obsolete entries (see section 8.3.8 Obsolete Entries). Zero-coefficients items are not shown. So, in this example, if
the fuzzy entries were unfuzzied, the untranslated entries were translated
and the obsolete entries were deleted, the mode line would merely display
`‘145t’` for the counters.

The main PO commands are those which do not fit into the other categories of
subsequent sections. These allow for quitting PO mode or for managing windows
in special ways.

`_`: Undo last modification to the PO file (`po-undo`). `Q`: Quit processing and save the PO file (`po-quit`). `q`: Quit processing, possibly after confirmation (`po-confirm-and-quit`). `0`: Temporary leave the PO file window (`po-other-window`). `?`: `h`: Show help about PO mode (`po-help`). `=`: Give some PO file statistics (`po-statistics`). `V`: Batch validate the format of the whole PO file (`po-validate`).

The command `_` (`po-undo`) interfaces to the Emacs
*undo* facility. See section ‘Undoing Changes’ in The Emacs Editor. Each time `_` is typed, modifications which the translator
did to the PO file are undone a little more. For the purpose of
undoing, each PO mode command is atomic. This is especially true for
the `RET` command: the whole edition made by using a single
use of this command is undone at once, even if the edition itself
implied several actions. However, while in the editing window, one
can undo the edition work quite parsimoniously.

The commands `Q` (`po-quit`) and `q`
(`po-confirm-and-quit`) are used when the translator is done with the
PO file. The former is a bit less verbose than the latter. If the file
has been modified, it is saved to disk first. In both cases, and prior to
all this, the commands check if any untranslated messages remain in the
PO file and, if so, the translator is asked if she really wants to leave
off working with this PO file. This is the preferred way of getting rid
of an Emacs PO file buffer. Merely killing it through the usual command
`C-x k` (`kill-buffer`) is not the tidiest way to proceed.

The command `0` (`po-other-window`) is another, softer way,
to leave PO mode, temporarily. It just moves the cursor to some other
Emacs window, and pops one if necessary. For example, if the translator
just got PO mode to show some source context in some other, she might
discover some apparent bug in the program source that needs correction.
This command allows the translator to change sex, become a programmer,
and have the cursor right into the window containing the program she
(or rather *he*) wants to modify. By later getting the cursor back
in the PO file window, or by asking Emacs to edit this file once again,
PO mode is then recovered.

The command `h` (`po-help`) displays a summary of all available PO
mode commands. The translator should then type any character to resume
normal PO mode operations. The command `?` has the same effect
as `h`.

The command `=` (`po-statistics`) computes the total number of
entries in the PO file, the ordinal of the current entry (counted from
1), the number of untranslated entries, the number of obsolete entries,
and displays all these numbers.

The command `V` (`po-validate`) launches `msgfmt` in
checking and verbose
mode over the current PO file. This command first offers to save the
current PO file on disk. The `msgfmt` tool, from GNU `gettext`,
has the purpose of creating a MO file out of a PO file, and PO mode uses
the features of this program for checking the overall format of a PO file,
as well as all individual entries.

The program `msgfmt` runs asynchronously with Emacs, so the
translator regains control immediately while her PO file is being studied.
Error output is collected in the Emacs `‘*compilation*’` buffer,
displayed in another window. The regular Emacs command `` C-x` ``
(`next-error`), as well as other usual compile commands, allow the
translator to reposition quickly to the offending parts of the PO file.
Once the cursor is on the line in error, the translator may decide on
any PO mode action which would help correcting the error.

### 8.3.3 Entry Positioning

The cursor in a PO file window is almost always part of
an entry. The only exceptions are the special case when the cursor
is after the last entry in the file, or when the PO file is
empty. The entry where the cursor is found to be is said to be the
current entry. Many PO mode commands operate on the current entry,
so moving the cursor does more than allowing the translator to browse
the PO file, this also selects on which entry commands operate.

Some PO mode commands alter the position of the cursor in a specialized
way. A few of those special purpose positioning are described here,
the others are described in following sections (for a complete list try
`C-h m`):

`.`: Redisplay the current entry (`po-current-entry`). `n`: Select the entry after the current one (`po-next-entry`). `p`: Select the entry before the current one (`po-previous-entry`). `<`: Select the first entry in the PO file (`po-first-entry`). `>`: Select the last entry in the PO file (`po-last-entry`). `m`: Record the location of the current entry for later use (`po-push-location`). `r`: Return to a previously saved entry location (`po-pop-location`). `x`: Exchange the current entry location with the previously saved one (`po-exchange-location`).

Any Emacs command able to reposition the cursor may be used
to select the current entry in PO mode, including commands which
move by characters, lines, paragraphs, screens or pages, and search
commands. However, there is a kind of standard way to display the
current entry in PO mode, which usual Emacs commands moving
the cursor do not especially try to enforce. The command `.`
(`po-current-entry`) has the sole purpose of redisplaying the
current entry properly, after the current entry has been changed by
means external to PO mode, or the Emacs screen otherwise altered.

It is yet to be decided if PO mode helps the translator, or otherwise
irritates her, by forcing a rigid window disposition while she
is doing her work. We originally had quite precise ideas about
how windows should behave, but on the other hand, anyone used to
Emacs is often happy to keep full control. Maybe a fixed window
disposition might be offered as a PO mode option that the translator
might activate or deactivate at will, so it could be offered on an
experimental basis. If nobody feels a real need for using it, or
a compulsion for writing it, we should drop this whole idea.
The incentive for doing it should come from translators rather than
programmers, as opinions from an experienced translator are surely
more worth to me than opinions from programmers *thinking* about
how *others* should do translation.

The commands `n` (`po-next-entry`) and `p`
(`po-previous-entry`) move the cursor the entry following,
or preceding, the current one. If `n` is given while the
cursor is on the last entry of the PO file, or if `p`
is given while the cursor is on the first entry, no move is done.

The commands `<` (`po-first-entry`) and `>`
(`po-last-entry`) move the cursor to the first entry, or last
entry, of the PO file. When the cursor is located past the last
entry in a PO file, most PO mode commands will return an error saying
`‘After last entry’`. Moreover, the commands `<` and `>`
have the special property of being able to work even when the cursor
is not into some PO file entry, and one may use them for nicely
correcting this situation. But even these commands will fail on a
truly empty PO file. There are development plans for the PO mode for it
to interactively fill an empty PO file from sources. See section 4.5 Marking Translatable Strings.

The translator may decide, before working at the translation of
a particular entry, that she needs to browse the remainder of the
PO file, maybe for finding the terminology or phraseology used
in related entries. She can of course use the standard Emacs idioms
for saving the current cursor location in some register, and use that
register for getting back, or else, use the location ring.

PO mode offers another approach, by which cursor locations may be saved
onto a special stack. The command `m` (`po-push-location`)
merely adds the location of current entry to the stack, pushing
the already saved locations under the new one. The command
`r` (`po-pop-location`) consumes the top stack element and
repositions the cursor to the entry associated with that top element.
This position is then lost, for the next `r` will move the cursor
to the previously saved location, and so on until no locations remain
on the stack.

If the translator wants the position to be kept on the location stack,
maybe for taking a look at the entry associated with the top
element, then go elsewhere with the intent of getting back later, she
ought to use `m` immediately after `r`.

The command `x` (`po-exchange-location`) simultaneously
repositions the cursor to the entry associated with the top element of
the stack of saved locations, and replaces that top element with the
location of the current entry before the move. Consequently, repeating
the `x` command toggles alternatively between two entries.
For achieving this, the translator will position the cursor on the
first entry, use `m`, then position to the second entry, and
merely use `x` for making the switch.

### 8.3.4 Normalizing Strings in Entries

There are many different ways for encoding a particular string into a
PO file entry, because there are so many different ways to split and
quote multi-line strings, and even, to represent special characters
by backslashed escaped sequences. Some features of PO mode rely on
the ability for PO mode to scan an already existing PO file for a
particular string encoded into the `msgid` field of some entry.
Even if PO mode has internally all the built-in machinery for
implementing this recognition easily, doing it fast is technically
difficult. To facilitate a solution to this efficiency problem,
we decided on a canonical representation for strings.

A conventional representation of strings in a PO file is currently
under discussion, and PO mode experiments with a canonical representation.
Having both `xgettext` and PO mode converging towards a uniform
way of representing equivalent strings would be useful, as the internal
normalization needed by PO mode could be automatically satisfied
when using `xgettext` from GNU `gettext`. An explicit
PO mode normalization should then be only necessary for PO files
imported from elsewhere, or for when the convention itself evolves.

So, for achieving normalization of at least the strings of a given
PO file needing a canonical representation, the following PO mode
command is available:

`M-x po-normalize`: Tidy the whole PO file by making entries more uniform.

The special command `M-x po-normalize`, which has no associated
keys, revises all entries, ensuring that strings of both original
and translated entries use uniform internal quoting in the PO file.
It also removes any crumb after the last entry. This command may be
useful for PO files freshly imported from elsewhere, or if we ever
improve on the canonical quoting format we use. This canonical format
is not only meant for getting cleaner PO files, but also for greatly
speeding up `msgid` string lookup for some other PO mode commands.

`M-x po-normalize` presently makes three passes over the entries.
The first implements heuristics for converting PO files for GNU
`gettext` 0.6 and earlier, in which `msgid` and `msgstr`
fields were using K&R style C string syntax for multi-line strings.
These heuristics may fail for comments not related to obsolete
entries and ending with a backslash; they also depend on subsequent
passes for finalizing the proper commenting of continued lines for
obsolete entries. This first pass might disappear once all oldish PO
files would have been adjusted. The second and third pass normalize
all `msgid` and `msgstr` strings respectively. They also
clean out those trailing backslashes used by XView's `msgfmt`
for continued lines.

Having such an explicit normalizing command allows for importing PO
files from other sources, but also eases the evolution of the current
convention, evolution driven mostly by aesthetic concerns, as of now.
It is easy to make suggested adjustments at a later time, as the
normalizing command and eventually, other GNU `gettext` tools
should greatly automate conformance. A description of the canonical
string format is given below, for the particular benefit of those not
having Emacs handy, and who would nevertheless want to handcraft
their PO files in nice ways.

Right now, in PO mode, strings are single line or multi-line. A string
goes multi-line if and only if it has *embedded* newlines, that
is, if it matches `‘[^\n]\n+[^\n]’`. So, we would have:

```
msgstr "\n\nHello, world!\n\n\n"
```

but, replacing the space by a newline, this becomes:

```
msgstr ""
"\n"
"\n"
"Hello,\n"
"world!\n"
"\n"
"\n"
```

We are deliberately using a caricatural example, here, to make the
point clearer. Usually, multi-lines are not that bad looking.
It is probable that we will implement the following suggestion.
We might lump together all initial newlines into the empty string,
and also all newlines introducing empty lines (that is, for n
> 1, the n-1'th last newlines would go together on a separate
string), so making the previous example appear:

```
msgstr "\n\n"
"Hello,\n"
"world!\n"
"\n\n"
```

There are a few yet undecided little points about string normalization,
to be documented in this manual, once these questions settle.

### 8.3.5 Translated Entries

Each PO file entry for which the `msgstr` field has been filled with
a translation, and which is not marked as fuzzy (see section 8.3.6 Fuzzy Entries),
is said to be a *translated* entry. Only translated entries will
later be compiled by GNU `msgfmt` and become usable in programs.
Other entry types will be excluded; translation will not occur for them.

Some commands are more specifically related to translated entry processing.

`t`: Find the next translated entry (`po-next-translated-entry`). `T`: Find the previous translated entry (`po-previous-translated-entry`).

The commands `t` (`po-next-translated-entry`) and `T`
(`po-previous-translated-entry`) move forwards or backwards, chasing
for an translated entry. If none is found, the search is extended and
wraps around in the PO file buffer.

Translated entries usually result from the translator having edited in
a translation for them, section 8.3.9 Modifying Translations. However, if the
variable `po-auto-fuzzy-on-edit` is not `nil`, the entry having
received a new translation first becomes a fuzzy entry, which ought to
be later unfuzzied before becoming an official, genuine translated entry.
See section 8.3.6 Fuzzy Entries.

### 8.3.6 Fuzzy Entries

Each PO file entry may have a set of *attributes*, which are
qualities given a name and explicitly associated with the translation,
using a special system comment. One of these attributes
has the name `fuzzy`, and entries having this attribute are said
to have a fuzzy translation. They are called fuzzy entries, for short.

Fuzzy entries, even if they account for translated entries for
most other purposes, usually call for revision by the translator.
Those may be produced by applying the program `msgmerge` to
update an older translated PO files according to a new PO template
file, when this tool hypothesises that some new `msgid` has
been modified only slightly out of an older one, and chooses to pair
what it thinks to be the old translation for the new modified entry.
The slight alteration in the original string (the `msgid` string)
should often be reflected in the translated string, and this requires
the intervention of the translator. For this reason, `msgmerge`
might mark some entries as being fuzzy.

Also, the translator may decide herself to mark an entry as fuzzy
for her own convenience, when she wants to remember that the entry
has to be later revisited. So, some commands are more specifically
related to fuzzy entry processing.

`f`: Find the next fuzzy entry (`po-next-fuzzy-entry`). `F`: Find the previous fuzzy entry (`po-previous-fuzzy-entry`). `TAB`: Remove the fuzzy attribute of the current entry (`po-unfuzzy`).

The commands `f` (`po-next-fuzzy-entry`) and `F`
(`po-previous-fuzzy-entry`) move forwards or backwards, chasing for
a fuzzy entry. If none is found, the search is extended and wraps
around in the PO file buffer.

The command `TAB` (`po-unfuzzy`) removes the fuzzy
attribute associated with an entry, usually leaving it translated.
Further, if the variable `po-auto-select-on-unfuzzy` has not
the `nil` value, the `TAB` command will automatically chase
for another interesting entry to work on. The initial value of
`po-auto-select-on-unfuzzy` is `nil`.

The initial value of `po-auto-fuzzy-on-edit` is `nil`. However,
if the variable `po-auto-fuzzy-on-edit` is set to `t`, any entry
edited through the `RET` command is marked fuzzy, as a way to
ensure some kind of double check, later. In this case, the usual paradigm
is that an entry becomes fuzzy (if not already) whenever the translator
modifies it. If she is satisfied with the translation, she then uses
`TAB` to pick another entry to work on, clearing the fuzzy attribute
on the same blow. If she is not satisfied yet, she merely uses `SPC`
to chase another entry, leaving the entry fuzzy.

The translator may also use the `DEL` command
(`po-fade-out-entry`) over any translated entry to mark it as being
fuzzy, when she wants to easily leave a trace she wants to later return
working at this entry.

Also, when time comes to quit working on a PO file buffer with the `q`
command, the translator is asked for confirmation, if fuzzy string
still exists.

### 8.3.7 Untranslated Entries

When `xgettext` originally creates a PO file, unless told
otherwise, it initializes the `msgid` field with the untranslated
string, and leaves the `msgstr` string to be empty. Such entries,
having an empty translation, are said to be *untranslated* entries.
Later, when the programmer slightly modifies some string right in
the program, this change is later reflected in the PO file
by the appearance of a new untranslated entry for the modified string.

The usual commands moving from entry to entry consider untranslated
entries on the same level as active entries. Untranslated entries
are easily recognizable by the fact they end with `‘msgstr ""’`.

The work of the translator might be (quite naively) seen as the process
of seeking for an untranslated entry, editing a translation for
it, and repeating these actions until no untranslated entries remain.
Some commands are more specifically related to untranslated entry
processing.

`u`: Find the next untranslated entry (`po-next-untranslated-entry`). `U`: Find the previous untranslated entry (`po-previous-untransted-entry`). `k`: Turn the current entry into an untranslated one (`po-kill-msgstr`).

The commands `u` (`po-next-untranslated-entry`) and `U`
(`po-previous-untransted-entry`) move forwards or backwards,
chasing for an untranslated entry. If none is found, the search is
extended and wraps around in the PO file buffer.

An entry can be turned back into an untranslated entry by
merely emptying its translation, using the command `k`
(`po-kill-msgstr`). See section 8.3.9 Modifying Translations.

Also, when time comes to quit working on a PO file buffer
with the `q` command, the translator is asked for confirmation,
if some untranslated string still exists.

### 8.3.8 Obsolete Entries

By *obsolete* PO file entries, we mean those entries which are
commented out, usually by `msgmerge` when it found that the
translation is not needed anymore by the package being localized.

The usual commands moving from entry to entry consider obsolete
entries on the same level as active entries. Obsolete entries are
easily recognizable by the fact that all their lines start with
`#`, even those lines containing `msgid` or `msgstr`.

Commands exist for emptying the translation or reinitializing it
to the original untranslated string. Commands interfacing with the
kill ring may force some previously saved text into the translation.
The user may interactively edit the translation. All these commands
may apply to obsolete entries, carefully leaving the entry obsolete
after the fact.

Moreover, some commands are more specifically related to obsolete
entry processing.

`o`: Find the next obsolete entry (`po-next-obsolete-entry`). `O`: Find the previous obsolete entry (`po-previous-obsolete-entry`). `DEL`: Make an active entry obsolete, or zap out an obsolete entry (`po-fade-out-entry`).

The commands `o` (`po-next-obsolete-entry`) and `O`
(`po-previous-obsolete-entry`) move forwards or backwards,
chasing for an obsolete entry. If none is found, the search is
extended and wraps around in the PO file buffer.

PO mode does not provide ways for un-commenting an obsolete entry
and making it active, because this would reintroduce an original
untranslated string which does not correspond to any marked string
in the program sources. This goes with the philosophy of never
introducing useless `msgid` values.

However, it is possible to comment out an active entry, so making
it obsolete. GNU `gettext` utilities will later react to the
disappearance of a translation by using the untranslated string.
The command `DEL` (`po-fade-out-entry`) pushes the current entry
a little further towards annihilation. If the entry is active (it is a
translated entry), then it is first made fuzzy. If it is already fuzzy,
then the entry is merely commented out, with confirmation. If the entry
is already obsolete, then it is completely deleted from the PO file.
It is easy to recycle the translation so deleted into some other PO file
entry, usually one which is untranslated. See section 8.3.9 Modifying Translations.

Here is a quite interesting problem to solve for later development of
PO mode, for those nights you are not sleepy. The idea would be that
PO mode might become bright enough, one of these days, to make good
guesses at retrieving the most probable candidate, among all obsolete
entries, for initializing the translation of a newly appeared string.
I think it might be a quite hard problem to do this algorithmically, as
we have to develop good and efficient measures of string similarity.
Right now, PO mode completely lets the decision to the translator,
when the time comes to find the adequate obsolete translation, it
merely tries to provide handy tools for helping her to do so.

### 8.3.9 Modifying Translations

PO mode prevents direct modification of the PO file, by the usual
means Emacs gives for altering a buffer's contents. By doing so,
it pretends helping the translator to avoid little clerical errors
about the overall file format, or the proper quoting of strings,
as those errors would be easily made. Other kinds of errors are
still possible, but some may be caught and diagnosed by the batch
validation process, which the translator may always trigger by the
`V` command. For all other errors, the translator has to rely on
her own judgment, and also on the linguistic reports submitted to her
by the users of the translated package, having the same mother tongue.

When the time comes to create a translation, correct an error diagnosed
mechanically or reported by a user, the translators have to resort to
using the following commands for modifying the translations.

`RET`: Interactively edit the translation (`po-edit-msgstr`). `LFD`: `C-j`: Reinitialize the translation with the original, untranslated string (`po-msgid-to-msgstr`). `k`: Save the translation on the kill ring, and delete it (`po-kill-msgstr`). `w`: Save the translation on the kill ring, without deleting it (`po-kill-ring-save-msgstr`). `y`: Replace the translation, taking the new from the kill ring (`po-yank-msgstr`).

The command `RET` (`po-edit-msgstr`) opens a new Emacs
window meant to edit in a new translation, or to modify an already existing
translation. The new window contains a copy of the translation taken from
the current PO file entry, all ready for edition, expunged of all quoting
marks, fully modifiable and with the complete extent of Emacs modifying
commands. When the translator is done with her modifications, she may use
`C-c C-c` to close the subedit window with the automatically requoted
results, or `C-c C-k` to abort her modifications. See section 8.3.11 Details of Sub Edition,
for more information.

The command `LFD` (`po-msgid-to-msgstr`) initializes, or
reinitializes the translation with the original string. This command is
normally used when the translator wants to redo a fresh translation of
the original string, disregarding any previous work.

It is possible to arrange so, whenever editing an untranslated
entry, the `LFD` command be automatically executed. If you set
`po-auto-edit-with-msgid` to `t`, the translation gets
initialised with the original string, in case none exists already.
The default value for `po-auto-edit-with-msgid` is `nil`.

In fact, whether it is best to start a translation with an empty
string, or rather with a copy of the original string, is a matter of
taste or habit. Sometimes, the source language and the
target language are so different that is simply best to start writing
on an empty page. At other times, the source and target languages
are so close that it would be a waste to retype a number of words
already being written in the original string. A translator may also
like having the original string right under her eyes, as she will
progressively overwrite the original text with the translation, even
if this requires some extra editing work to get rid of the original.

The command `k` (`po-kill-msgstr`) merely empties the
translation string, so turning the entry into an untranslated
one. But while doing so, its previous contents is put apart in
a special place, known as the kill ring. The command `w`
(`po-kill-ring-save-msgstr`) has also the effect of taking a
copy of the translation onto the kill ring, but it otherwise leaves
the entry alone, and does *not* remove the translation from the
entry. Both commands use exactly the Emacs kill ring, which is shared
between buffers, and which is well known already to Emacs lovers.

The translator may use `k` or `w` many times in the course
of her work, as the kill ring may hold several saved translations.
From the kill ring, strings may later be reinserted in various
Emacs buffers. In particular, the kill ring may be used for moving
translation strings between different entries of a single PO file
buffer, or if the translator is handling many such buffers at once,
even between PO files.

To facilitate exchanges with buffers which are not in PO mode, the
translation string put on the kill ring by the `k` command is fully
unquoted before being saved: external quotes are removed, multi-line
strings are concatenated, and backslash escaped sequences are turned
into their corresponding characters. In the special case of obsolete
entries, the translation is also uncommented prior to saving.

The command `y` (`po-yank-msgstr`) completely replaces the
translation of the current entry by a string taken from the kill ring.
Following Emacs terminology, we then say that the replacement
string is *yanked* into the PO file buffer.
See section ‘Yanking’ in The Emacs Editor.
The first time `y` is used, the translation receives the value of
the most recent addition to the kill ring. If `y` is typed once
again, immediately, without intervening keystrokes, the translation
just inserted is taken away and replaced by the second most recent
addition to the kill ring. By repeating `y` many times in a row,
the translator may travel along the kill ring for saved strings,
until she finds the string she really wanted.

When a string is yanked into a PO file entry, it is fully and
automatically requoted for complying with the format PO files should
have. Further, if the entry is obsolete, PO mode then appropriately
push the inserted string inside comments. Once again, translators
should not burden themselves with quoting considerations besides, of
course, the necessity of the translated string itself respective to
the program using it.

Note that `k` or `w` are not the only commands pushing strings
on the kill ring, as almost any PO mode command replacing translation
strings (or the translator comments) automatically saves the old string
on the kill ring. The main exceptions to this general rule are the
yanking commands themselves.

To better illustrate the operation of killing and yanking, let's
use an actual example, taken from a common situation. When the
programmer slightly modifies some string right in the program, his
change is later reflected in the PO file by the appearance
of a new untranslated entry for the modified string, and the fact
that the entry translating the original or unmodified string becomes
obsolete. In many cases, the translator might spare herself some work
by retrieving the unmodified translation from the obsolete entry,
then initializing the untranslated entry `msgstr` field with
this retrieved translation. Once this done, the obsolete entry is
not wanted anymore, and may be safely deleted.

When the translator finds an untranslated entry and suspects that a
slight variant of the translation exists, she immediately uses `m`
to mark the current entry location, then starts chasing obsolete
entries with `o`, hoping to find some translation corresponding
to the unmodified string. Once found, she uses the `DEL` command
for deleting the obsolete entry, knowing that `DEL` also *kills*
the translation, that is, pushes the translation on the kill ring.
Then, `r` returns to the initial untranslated entry, and `y`
then *yanks* the saved translation right into the `msgstr`
field. The translator is then free to use `RET` for fine
tuning the translation contents, and maybe to later use `u`,
then `m` again, for going on with the next untranslated string.

When some sequence of keys has to be typed over and over again, the
translator may find it useful to become better acquainted with the Emacs
capability of learning these sequences and playing them back under request.
See section ‘Keyboard Macros’ in The Emacs Editor.

### 8.3.10 Modifying Comments

Any translation work done seriously will raise many linguistic
difficulties, for which decisions have to be made, and the choices
further documented. These documents may be saved within the
PO file in form of translator comments, which the translator
is free to create, delete, or modify at will. These comments may
be useful to herself when she returns to this PO file after a while.

Comments not having whitespace after the initial `‘#’`, for example,
those beginning with `‘#.’` or `‘#:’`, are *not* translator
comments, they are exclusively created by other `gettext` tools.
So, the commands below will never alter such system added comments,
they are not meant for the translator to modify. See section 3 The Format of PO Files.

The following commands are somewhat similar to those modifying translations,
so the general indications given for those apply here. See section 8.3.9 Modifying Translations.

`#`: Interactively edit the translator comments (`po-edit-comment`). `K`: Save the translator comments on the kill ring, and delete it (`po-kill-comment`). `W`: Save the translator comments on the kill ring, without deleting it (`po-kill-ring-save-comment`). `Y`: Replace the translator comments, taking the new from the kill ring (`po-yank-comment`).

These commands parallel PO mode commands for modifying the translation
strings, and behave much the same way as they do, except that they handle
this part of PO file comments meant for translator usage, rather
than the translation strings. So, if the descriptions given below are
slightly succinct, it is because the full details have already been given.
See section 8.3.9 Modifying Translations.

The command `#` (`po-edit-comment`) opens a new Emacs window
containing a copy of the translator comments on the current PO file entry.
If there are no such comments, PO mode understands that the translator wants
to add a comment to the entry, and she is presented with an empty screen.
Comment marks (`#`) and the space following them are automatically
removed before edition, and reinstated after. For translator comments
pertaining to obsolete entries, the uncommenting and recommenting operations
are done twice. Once in the editing window, the keys `C-c C-c`
allow the translator to tell she is finished with editing the comment.
See section 8.3.11 Details of Sub Edition, for further details.

Functions found on `po-subedit-mode-hook`, if any, are executed after
the string has been inserted in the edit buffer.

The command `K` (`po-kill-comment`) gets rid of all
translator comments, while saving those comments on the kill ring.
The command `W` (`po-kill-ring-save-comment`) takes
a copy of the translator comments on the kill ring, but leaves
them undisturbed in the current entry. The command `Y`
(`po-yank-comment`) completely replaces the translator comments
by a string taken at the front of the kill ring. When this command
is immediately repeated, the comments just inserted are withdrawn,
and replaced by other strings taken along the kill ring.

On the kill ring, all strings have the same nature. There is no
distinction between *translation* strings and *translator
comments* strings. So, for example, let's presume the translator
has just finished editing a translation, and wants to create a new
translator comment to document why the previous translation was
not good, just to remember what was the problem. Foreseeing that she
will do that in her documentation, the translator may want to quote
the previous translation in her translator comments. To do so, she
may initialize the translator comments with the previous translation,
still at the head of the kill ring. Because editing already pushed the
previous translation on the kill ring, she merely has to type `M-w`
prior to `#`, and the previous translation will be right there,
all ready for being introduced by some explanatory text.

On the other hand, presume there are some translator comments already
and that the translator wants to add to those comments, instead
of wholly replacing them. Then, she should edit the comment right
away with `#`. Once inside the editing window, she can use the
regular Emacs commands `C-y` (`yank`) and `M-y`
(`yank-pop`) to get the previous translation where she likes.

### 8.3.11 Details of Sub Edition

The PO subedit minor mode has a few peculiarities worth being described
in fuller detail. It installs a few commands over the usual editing set
of Emacs, which are described below.

`C-c C-c`: Complete edition (`po-subedit-exit`). `C-c C-k`: Abort edition (`po-subedit-abort`). `C-c C-a`: Consult auxiliary PO files (`po-subedit-cycle-auxiliary`).

The window's contents represents a translation for a given message,
or a translator comment. The translator may modify this window to
her heart's content. Once this is done, the command `C-c C-c`
(`po-subedit-exit`) may be used to return the edited translation into
the PO file, replacing the original translation, even if it moved out of
sight or if buffers were switched.

If the translator becomes unsatisfied with her translation or comment,
to the extent she prefers keeping what was existent prior to the
`RET` or `#` command, she may use the command `C-c C-k`
(`po-subedit-abort`) to merely get rid of edition, while preserving
the original translation or comment. Another way would be for her to exit
normally with `C-c C-c`, then type `U` once for undoing the
whole effect of last edition.

The command `C-c C-a` (`po-subedit-cycle-auxiliary`)
allows for glancing through translations
already achieved in other languages, directly while editing the current
translation. This may be quite convenient when the translator is fluent
at many languages, but of course, only makes sense when such completed
auxiliary PO files are already available to her (see section 8.3.13 Consulting Auxiliary PO Files).

Functions found on `po-subedit-mode-hook`, if any, are executed after
the string has been inserted in the edit buffer.

While editing her translation, the translator should pay attention to not
inserting unwanted `RET` (newline) characters at the end of
the translated string if those are not meant to be there, or to removing
such characters when they are required. Since these characters are not
visible in the editing buffer, they are easily introduced by mistake.
To help her, `RET` automatically puts the character `<`
at the end of the string being edited, but this `<` is not really
part of the string. On exiting the editing window with `C-c C-c`,
PO mode automatically removes such `<` and all whitespace added after
it. If the translator adds characters after the terminating `<`, it
looses its delimiting property and integrally becomes part of the string.
If she removes the delimiting `<`, then the edited string is taken
*as is*, with all trailing newlines, even if invisible. Also, if
the translated string ought to end itself with a genuine `<`, then
the delimiting `<` may not be removed; so the string should appear,
in the editing window, as ending with two `<` in a row.

When a translation (or a comment) is being edited, the translator may move
the cursor back into the PO file buffer and freely move to other entries,
browsing at will. If, with an edition pending, the translator wanders in the
PO file buffer, she may decide to start modifying another entry. Each entry
being edited has its own subedit buffer. It is possible to simultaneously
edit the translation *and* the comment of a single entry, or to
edit entries in different PO files, all at once. Typing `RET`
on a field already being edited merely resumes that particular edit. Yet,
the translator should better be comfortable at handling many Emacs windows!

Pending subedits may be completed or aborted in any order, regardless
of how or when they were started. When many subedits are pending and the
translator asks for quitting the PO file (with the `q` command), subedits
are automatically resumed one at a time, so she may decide for each of them.

### 8.3.12 C Sources Context

PO mode is particularly powerful when used with PO files
created through GNU `gettext` utilities, as those utilities
insert special comments in the PO files they generate.
Some of these special comments relate the PO file entry to
exactly where the untranslated string appears in the program sources.

When the translator gets to an untranslated entry, she is fairly
often faced with an original string which is not as informative as
it normally should be, being succinct, cryptic, or otherwise ambiguous.
Before choosing how to translate the string, she needs to understand
better what the string really means and how tight the translation has
to be. Most of the time, when problems arise, the only way left to make
her judgment is looking at the true program sources from where this
string originated, searching for surrounding comments the programmer
might have put in there, and looking around for helping clues of
*any* kind.

Surely, when looking at program sources, the translator will receive
more help if she is a fluent programmer. However, even if she is
not versed in programming and feels a little lost in C code, the
translator should not be shy at taking a look, once in a while.
It is most probable that she will still be able to find some of the
hints she needs. She will learn quickly to not feel uncomfortable
in program code, paying more attention to programmer's comments,
variable and function names (if he dared choosing them well), and
overall organization, than to the program code itself.

The following commands are meant to help the translator at getting
program source context for a PO file entry.

`s`: Resume the display of a program source context, or cycle through them (`po-cycle-source-reference`). `M-s`: Display of a program source context selected by menu (`po-select-source-reference`). `S`: Add a directory to the search path for source files (`po-consider-source-path`). `M-S`: Delete a directory from the search path for source files (`po-ignore-source-path`).

The commands `s` (`po-cycle-source-reference`) and `M-s`
(`po-select-source-reference`) both open another window displaying
some source program file, and already positioned in such a way that
it shows an actual use of the string to be translated. By doing
so, the command gives source program context for the string. But if
the entry has no source context references, or if all references
are unresolved along the search path for program sources, then the
command diagnoses this as an error.

Even if `s` (or `M-s`) opens a new window, the cursor stays
in the PO file window. If the translator really wants to
get into the program source window, she ought to do it explicitly,
maybe by using command `O`.

When `s` is typed for the first time, or for a PO file entry which
is different of the last one used for getting source context, then the
command reacts by giving the first context available for this entry,
if any. If some context has already been recently displayed for the
current PO file entry, and the translator wandered off to do other
things, typing `s` again will merely resume, in another window,
the context last displayed. In particular, if the translator moved
the cursor away from the context in the source file, the command will
bring the cursor back to the context. By using `s` many times
in a row, with no other commands intervening, PO mode will cycle to
the next available contexts for this particular entry, getting back
to the first context once the last has been shown.

The command `M-s` behaves differently. Instead of cycling through
references, it lets the translator choose a particular reference among
many, and displays that reference. It is best used with completion,
if the translator types `TAB` immediately after `M-s`, in
response to the question, she will be offered a menu of all possible
references, as a reminder of which are the acceptable answers.
This command is useful only where there are really many contexts
available for a single string to translate.

Program source files are usually found relative to where the PO
file stands. As a special provision, when this fails, the file is
also looked for, but relative to the directory immediately above it.
Those two cases take proper care of most PO files. However, it might
happen that a PO file has been moved, or is edited in a different
place than its normal location. When this happens, the translator
should tell PO mode in which directory normally sits the genuine PO
file. Many such directories may be specified, and all together, they
constitute what is called the *search path* for program sources.
The command `S` (`po-consider-source-path`) is used to interactively
enter a new directory at the front of the search path, and the command
`M-S` (`po-ignore-source-path`) is used to select, with completion,
one of the directories she does not want anymore on the search path.

### 8.3.13 Consulting Auxiliary PO Files

PO mode is able to help the knowledgeable translator, being fluent in
many languages, at taking advantage of translations already achieved
in other languages she just happens to know. It provides these other
language translations as additional context for her own work. Moreover,
it has features to ease the production of translations for many languages
at once, for translators preferring to work in this way.

An *auxiliary* PO file is an existing PO file meant for the same
package the translator is working on, but targeted to a different mother
tongue language. Commands exist for declaring and handling auxiliary
PO files, and also for showing contexts for the entry under work.

Here are the auxiliary file commands available in PO mode.

`a`: Seek auxiliary files for another translation for the same entry (`po-cycle-auxiliary`). `C-c C-a`: Switch to a particular auxiliary file (`po-select-auxiliary`). `A`: Declare this PO file as an auxiliary file (`po-consider-as-auxiliary`). `M-A`: Remove this PO file from the list of auxiliary files (`po-ignore-as-auxiliary`).

Command `A` (`po-consider-as-auxiliary`) adds the current
PO file to the list of auxiliary files, while command `M-A`
(`po-ignore-as-auxiliary` just removes it.

The command `a` (`po-cycle-auxiliary`) seeks all auxiliary PO
files, round-robin, searching for a translated entry in some other language
having an `msgid` field identical as the one for the current entry.
The found PO file, if any, takes the place of the current PO file in
the display (its window gets on top). Before doing so, the current PO
file is also made into an auxiliary file, if not already. So, `a`
in this newly displayed PO file will seek another PO file, and so on,
so repeating `a` will eventually yield back the original PO file.

The command `C-c C-a` (`po-select-auxiliary`) asks the translator
for her choice of a particular auxiliary file, with completion, and
then switches to that selected PO file. The command also checks if
the selected file has an `msgid` field identical as the one for
the current entry, and if yes, this entry becomes current. Otherwise,
the cursor of the selected file is left undisturbed.

For all this to work fully, auxiliary PO files will have to be normalized,
in that way that `msgid` fields should be written *exactly*
the same way. It is possible to write `msgid` fields in various
ways for representing the same string, different writing would break the
proper behaviour of the auxiliary file commands of PO mode. This is not
expected to be much a problem in practice, as most existing PO files have
their `msgid` entries written by the same GNU `gettext` tools.

However, PO files initially created by PO mode itself, while marking
strings in source files, are normalised differently. So are PO
files resulting of the `‘M-x normalize’` command. Until these
discrepancies between PO mode and other GNU `gettext` tools get
fully resolved, the translator should stay aware of normalisation issues.

## 8.4 Using Translation Compendia

A *compendium* is a special PO file containing a set of
translations recurring in many different packages. The translator can
use gettext tools to build a new compendium, to add entries to her
compendium, and to initialize untranslated entries, or to update
already translated entries, from translations kept in the compendium.

### 8.4.1 Creating Compendia

Basically every PO file consisting of translated entries only can be
declared as a valid compendium. Often the translator wants to have
special compendia; let's consider two cases: concatenating PO
files and extracting a message subset from a PO file.

#### 8.4.1.1 Concatenate PO Files

To concatenate several valid PO files into one compendium file you can
use `‘msgcomm’` or `‘msgcat’` (the latter preferred):

```
msgcat -o compendium.po file1.po file2.po
```

By default, `msgcat` will accumulate divergent translations
for the same string. Those occurrences will be marked as `fuzzy`
and highly visible decorated; calling `msgcat` on
‘file1.po’:

```
#: src/hello.c:200
#, c-format
msgid "Report bugs to <%s>.\n"
msgstr "Comunicar `bugs' a <%s>.\n"
```

and ‘file2.po’:

```
#: src/bye.c:100
#, c-format
msgid "Report bugs to <%s>.\n"
msgstr "Comunicar \"bugs\" a <%s>.\n"
```

will result in:

```
#: src/hello.c:200 src/bye.c:100
#, fuzzy, c-format
msgid "Report bugs to <%s>.\n"
msgstr ""
"#-#-#-#-#  file1.po  #-#-#-#-#\n"
"Comunicar `bugs' a <%s>.\n"
"#-#-#-#-#  file2.po  #-#-#-#-#\n"
"Comunicar \"bugs\" a <%s>.\n"
```

The translator will have to resolve this “conflict” manually; she
has to decide whether the first or the second version is appropriate
(or provide a new translation), to delete the “marker lines”, and
finally to remove the `fuzzy` mark.

If the translator knows in advance the first found translation of a
message is always the best translation she can make use to the
`‘--use-first’` switch:

```
msgcat --use-first -o compendium.po file1.po file2.po
```

A good compendium file must not contain `fuzzy` or untranslated
entries. If input files are “dirty” you must preprocess the input
files or postprocess the result using `‘msgattrib --translated --no-fuzzy’`.

#### 8.4.1.2 Extract a Message Subset from a PO File

Nobody wants to translate the same messages again and again; thus you
may wish to have a compendium file containing ‘getopt.c’ messages.

To extract a message subset (e.g., all ‘getopt.c’ messages) from an
existing PO file into one compendium file you can use `‘msggrep’`:

```
msggrep --location src/getopt.c -o compendium.po file.po
```

### 8.4.2 Using Compendia

You can use a compendium file to initialize a translation from scratch
or to update an already existing translation.

#### 8.4.2.1 Initialize a New Translation File

Since a PO file with translations does not exist the translator can
merely use ‘/dev/null’ to fake the “old” translation file.

```
msgmerge --compendium compendium.po -o file.po /dev/null file.pot
```

#### 8.4.2.2 Update an Existing Translation File

Concatenate the compendium file(s) and the existing PO, merge the
result with the POT file and remove the obsolete entries (optional,
here done using `‘sed’`):

```
msgcat --use-first -o update.po compendium1.po compendium2.po file.po
msgmerge update.po file.pot | msgattrib --no-obsolete > file.po
```

---

Go to the first, previous, next, last section, table of contents.
