# Supplementary material for: rDock: A Fast, Versatile and Open Source Program for Docking Ligands to Proteins and Nucleic Acids
Source: PLoS Comput Biol. 2014 Apr 10;10(4):e1003571. doi: 10.1371/journal.pcbi.1003571 (PMC3983074; doi:10.1371/journal.pcbi.1003571)
Supplement: Software S1 — Compressed file with the source code of the rDock software for ligand docking to Proteins and Nucleic Acids. (GZ) [file pcbi.1003571.s010.gz › rDock_2013.1_src/gettext-0.18.tar.gz!/gettext-0.18/gettext-tools/doc/gettext_11.html]

GNU gettext utilities - 11 The Programmer's View

Go to the first, previous, next, last section, table of contents.

---

# 11 The Programmer's View

One aim of the current message catalog implementation provided by
GNU `gettext` was to use the system's message catalog handling, if the
installer wishes to do so. So we perhaps should first take a look at
the solutions we know about. The people in the POSIX committee did not
manage to agree on one of the semi-official standards which we'll
describe below. In fact they couldn't agree on anything, so they decided
only to include an example of an interface. The major Unix vendors
are split in the usage of the two most important specifications: X/Open's
catgets vs. Uniforum's gettext interface. We'll describe them both and
later explain our solution of this dilemma.

## 11.1 About `catgets`

The `catgets` implementation is defined in the X/Open Portability
Guide, Volume 3, XSI Supplementary Definitions, Chapter 5. But the
process of creating this standard seemed to be too slow for some of
the Unix vendors so they created their implementations on preliminary
versions of the standard. Of course this leads again to problems while
writing platform independent programs: even the usage of `catgets`
does not guarantee a unique interface.

Another, personal comment on this that only a bunch of committee members
could have made this interface. They never really tried to program
using this interface. It is a fast, memory-saving implementation, an
user can happily live with it. But programmers hate it (at least I and
some others do...)

But we must not forget one point: after all the trouble with transferring
the rights on Unix(tm) they at last came to X/Open, the very same who
published this specification. This leads me to making the prediction
that this interface will be in future Unix standards (e.g. Spec1170) and
therefore part of all Unix implementation (implementations, which are
*allowed* to wear this name).

### 11.1.1 The Interface

The interface to the `catgets` implementation consists of three
functions which correspond to those used in file access: `catopen`
to open the catalog for using, `catgets` for accessing the message
tables, and `catclose` for closing after work is done. Prototypes
for the functions and the needed definitions are in the
`<nl_types.h>` header file.

`catopen` is used like in this:

```
nl_catd catd = catopen ("catalog_name", 0);
```

The function takes as the argument the name of the catalog. This usual
refers to the name of the program or the package. The second parameter
is not further specified in the standard. I don't even know whether it
is implemented consistently among various systems. So the common advice
is to use `0` as the value. The return value is a handle to the
message catalog, equivalent to handles to file returned by `open`.

This handle is of course used in the `catgets` function which can
be used like this:

```
char *translation = catgets (catd, set_no, msg_id, "original string");
```

The first parameter is this catalog descriptor. The second parameter
specifies the set of messages in this catalog, in which the message
described by `msg_id` is obtained. `catgets` therefore uses a
three-stage addressing:

```
catalog name => set number => message ID => translation
```

The fourth argument is not used to address the translation. It is given
as a default value in case when one of the addressing stages fail. One
important thing to remember is that although the return type of catgets
is `char *` the resulting string *must not* be changed. It
should better be `const char *`, but the standard is published in
1988, one year before ANSI C.

The last of these functions is used and behaves as expected:

```
catclose (catd);
```

After this no `catgets` call using the descriptor is legal anymore.

### 11.1.2 Problems with the `catgets` Interface?!

Now that this description seemed to be really easy -- where are the
problems we speak of? In fact the interface could be used in a
reasonable way, but constructing the message catalogs is a pain. The
reason for this lies in the third argument of `catgets`: the unique
message ID. This has to be a numeric value for all messages in a single
set. Perhaps you could imagine the problems keeping such a list while
changing the source code. Add a new message here, remove one there. Of
course there have been developed a lot of tools helping to organize this
chaos but one as the other fails in one aspect or the other. We don't
want to say that the other approach has no problems but they are far
more easy to manage.

## 11.2 About `gettext`

The definition of the `gettext` interface comes from a Uniforum
proposal. It was submitted there by Sun, who had implemented the
`gettext` function in SunOS 4, around 1990. Nowadays, the
`gettext` interface is specified by the OpenI18N standard.

The main point about this solution is that it does not follow the
method of normal file handling (open-use-close) and that it does not
burden the programmer with so many tasks, especially the unique key handling.
Of course here also a unique key is needed, but this key is the message
itself (how long or short it is). See section 11.3 Comparing the Two Interfaces for a more
detailed comparison of the two methods.

The following section contains a rather detailed description of the
interface. We make it that detailed because this is the interface
we chose for the GNU `gettext` Library. Programmers interested
in using this library will be interested in this description.

### 11.2.1 The Interface

The minimal functionality an interface must have is a) to select a
domain the strings are coming from (a single domain for all programs is
not reasonable because its construction and maintenance is difficult,
perhaps impossible) and b) to access a string in a selected domain.

This is principally the description of the `gettext` interface. It
has a global domain which unqualified usages reference. Of course this
domain is selectable by the user.

```
char *textdomain (const char *domain_name);
```

This provides the possibility to change or query the current status of
the current global domain of the `LC_MESSAGE` category. The
argument is a null-terminated string, whose characters must be legal in
the use in filenames. If the domain\_name argument is `NULL`,
the function returns the current value. If no value has been set
before, the name of the default domain is returned: *messages*.
Please note that although the return value of `textdomain` is of
type `char *` no changing is allowed. It is also important to know
that no checks of the availability are made. If the name is not
available you will see this by the fact that no translations are provided.

To use a domain set by `textdomain` the function

```
char *gettext (const char *msgid);
```

is to be used. This is the simplest reasonable form one can imagine.
The translation of the string msgid is returned if it is available
in the current domain. If it is not available, the argument itself is
returned. If the argument is `NULL` the result is undefined.

One thing which should come into mind is that no explicit dependency to
the used domain is given. The current value of the domain is used.
If this changes between two
executions of the same `gettext` call in the program, both calls
reference a different message catalog.

For the easiest case, which is normally used in internationalized
packages, once at the beginning of execution a call to `textdomain`
is issued, setting the domain to a unique name, normally the package
name. In the following code all strings which have to be translated are
filtered through the gettext function. That's all, the package speaks
your language.

### 11.2.2 Solving Ambiguities

While this single name domain works well for most applications there
might be the need to get translations from more than one domain. Of
course one could switch between different domains with calls to
`textdomain`, but this is really not convenient nor is it fast. A
possible situation could be one case subject to discussion during this
writing: all
error messages of functions in the set of common used functions should
go into a separate domain `error`. By this mean we would only need
to translate them once.
Another case are messages from a library, as these *have* to be
independent of the current domain set by the application.

For this reasons there are two more functions to retrieve strings:

```
char *dgettext (const char *domain_name, const char *msgid);
char *dcgettext (const char *domain_name, const char *msgid,
                 int category);
```

Both take an additional argument at the first place, which corresponds
to the argument of `textdomain`. The third argument of
`dcgettext` allows to use another locale category but `LC_MESSAGES`.
But I really don't know where this can be useful. If the
domain\_name is `NULL` or category has an value beside
the known ones, the result is undefined. It should also be noted that
this function is not part of the second known implementation of this
function family, the one found in Solaris.

A second ambiguity can arise by the fact, that perhaps more than one
domain has the same name. This can be solved by specifying where the
needed message catalog files can be found.

```
char *bindtextdomain (const char *domain_name,
                      const char *dir_name);
```

Calling this function binds the given domain to a file in the specified
directory (how this file is determined follows below). Especially a
file in the systems default place is not favored against the specified
file anymore (as it would be by solely using `textdomain`). A
`NULL` pointer for the dir\_name parameter returns the binding
associated with domain\_name. If domain\_name itself is
`NULL` nothing happens and a `NULL` pointer is returned. Here
again as for all the other functions is true that none of the return
value must be changed!

It is important to remember that relative path names for the
dir\_name parameter can be trouble. Since the path is always
computed relative to the current directory different results will be
achieved when the program executes a `chdir` command. Relative
paths should always be avoided to avoid dependencies and
unreliabilities.

### 11.2.3 Locating Message Catalog Files

Because many different languages for many different packages have to be
stored we need some way to add these information to file message catalog
files. The way usually used in Unix environments is have this encoding
in the file name. This is also done here. The directory name given in
`bindtextdomain`s second argument (or the default directory),
followed by the name of the locale, the locale category, and the domain name
are concatenated:

```
dir_name/locale/LC_category/domain_name.mo
```

The default value for dir\_name is system specific. For the GNU
library, and for packages adhering to its conventions, it's:

```
/usr/local/share/locale
```

locale is the name of the locale category which is designated by
`LC_category`. For `gettext` and `dgettext` this
`LC_category` is always `LC_MESSAGES`.(3)
The name of the locale category is determined through
`setlocale (LC_category, NULL)`.
(4)
When using the function `dcgettext`, you can specify the locale category
through the third argument.

### 11.2.4 How to specify the output character set `gettext` uses

`gettext` not only looks up a translation in a message catalog. It
also converts the translation on the fly to the desired output character
set. This is useful if the user is working in a different character set
than the translator who created the message catalog, because it avoids
distributing variants of message catalogs which differ only in the
character set.

The output character set is, by default, the value of `nl_langinfo
(CODESET)`, which depends on the `LC_CTYPE` part of the current
locale. But programs which store strings in a locale independent way
(e.g. UTF-8) can request that `gettext` and related functions
return the translations in that encoding, by use of the
`bind_textdomain_codeset` function.

Note that the msgid argument to `gettext` is not subject to
character set conversion. Also, when `gettext` does not find a
translation for msgid, it returns msgid unchanged --
independently of the current output character set. It is therefore
recommended that all msgids be US-ASCII strings.

Function: char \* **bind\_textdomain\_codeset** *(const char \*domainname, const char \*codeset)*: The `bind_textdomain_codeset` function can be used to specify the output character set for message catalogs for domain domainname. The codeset argument must be a valid codeset name which can be used for the `iconv_open` function, or a null pointer.

If the codeset parameter is the null pointer,
`bind_textdomain_codeset` returns the currently selected codeset
for the domain with the name domainname. It returns `NULL` if
no codeset has yet been selected.

The `bind_textdomain_codeset` function can be used several times.
If used multiple times with the same domainname argument, the
later call overrides the settings made by the earlier one.

The `bind_textdomain_codeset` function returns a pointer to a
string containing the name of the selected codeset. The string is
allocated internally in the function and must not be changed by the
user. If the system went out of core during the execution of
`bind_textdomain_codeset`, the return value is `NULL` and the
global variable errno is set accordingly.

### 11.2.5 Using contexts for solving ambiguities

One place where the `gettext` functions, if used normally, have big
problems is within programs with graphical user interfaces (GUIs). The
problem is that many of the strings which have to be translated are very
short. They have to appear in pull-down menus which restricts the
length. But strings which are not containing entire sentences or at
least large fragments of a sentence may appear in more than one
situation in the program but might have different translations. This is
especially true for the one-word strings which are frequently used in
GUI programs.

As a consequence many people say that the `gettext` approach is
wrong and instead `catgets` should be used which indeed does not
have this problem. But there is a very simple and powerful method to
handle this kind of problems with the `gettext` functions.

Contexts can be added to strings to be translated. A context dependent
translation lookup is when a translation for a given string is searched,
that is limited to a given context. The translation for the same string
in a different context can be different. The different translations of
the same string in different contexts can be stored in the in the same
MO file, and can be edited by the translator in the same PO file.

The ‘gettext.h’ include file contains the lookup macros for strings
with contexts. They are implemented as thin macros and inline functions
over the functions from `<libintl.h>`.

```
const char *pgettext (const char *msgctxt, const char *msgid);
```

In a call of this macro, msgctxt and msgid must be string
literals. The macro returns the translation of msgid, restricted
to the context given by msgctxt.

The msgctxt string is visible in the PO file to the translator.
You should try to make it somehow canonical and never changing. Because
every time you change an msgctxt, the translator will have to review
the translation of msgid.

Finding a canonical msgctxt string that doesn't change over time can
be hard. But you shouldn't use the file name or class name containing the
`pgettext` call -- because it is a common development task to rename
a file or a class, and it shouldn't cause translator work. Also you shouldn't
use a comment in the form of a complete English sentence as msgctxt --
because orthography or grammar changes are often applied to such sentences,
and again, it shouldn't force the translator to do a review.

The `‘p’` in `‘pgettext’` stands for “particular”: `pgettext`
fetches a particular translation of the msgid.

```
const char *dpgettext (const char *domain_name,
                       const char *msgctxt, const char *msgid);
const char *dcpgettext (const char *domain_name,
                        const char *msgctxt, const char *msgid,
                        int category);
```

These are generalizations of `pgettext`. They behave similarly to
`dgettext` and `dcgettext`, respectively. The domain\_name
argument defines the translation domain. The category argument
allows to use another locale category than `LC_MESSAGES`.

As as example consider the following fictional situation. A GUI program
has a menu bar with the following entries:

```
+------------+------------+--------------------------------------+
| File       | Printer    |                                      |
+------------+------------+--------------------------------------+
| Open     | | Select   |
| New      | | Open     |
+----------+ | Connect  |
             +----------+
```

To have the strings `File`, `Printer`, `Open`,
`New`, `Select`, and `Connect` translated there has to be
at some point in the code a call to a function of the `gettext`
family. But in two places the string passed into the function would be
`Open`. The translations might not be the same and therefore we
are in the dilemma described above.

What distinguishes the two places is the menu path from the menu root to
the particular menu entries:

```
Menu|File
Menu|Printer
Menu|File|Open
Menu|File|New
Menu|Printer|Select
Menu|Printer|Open
Menu|Printer|Connect
```

The context is thus the menu path without its last part. So, the calls
look like this:

```
pgettext ("Menu|", "File")
pgettext ("Menu|", "Printer")
pgettext ("Menu|File|", "Open")
pgettext ("Menu|File|", "New")
pgettext ("Menu|Printer|", "Select")
pgettext ("Menu|Printer|", "Open")
pgettext ("Menu|Printer|", "Connect")
```

Whether or not to use the `‘|’` character at the end of the context is a
matter of style.

For more complex cases, where the msgctxt or msgid are not
string literals, more general macros are available:

```
const char *pgettext_expr (const char *msgctxt, const char *msgid);
const char *dpgettext_expr (const char *domain_name,
                            const char *msgctxt, const char *msgid);
const char *dcpgettext_expr (const char *domain_name,
                             const char *msgctxt, const char *msgid,
                             int category);
```

Here msgctxt and msgid can be arbitrary string-valued expressions.
These macros are more general. But in the case that both argument expressions
are string literals, the macros without the `‘_expr’` suffix are more
efficient.

### 11.2.6 Additional functions for plural forms

The functions of the `gettext` family described so far (and all the
`catgets` functions as well) have one problem in the real world
which have been neglected completely in all existing approaches. What
is meant here is the handling of plural forms.

Looking through Unix source code before the time anybody thought about
internationalization (and, sadly, even afterwards) one can often find
code similar to the following:

```
   printf ("%d file%s deleted", n, n == 1 ? "" : "s");
```

After the first complaints from people internationalizing the code people
either completely avoided formulations like this or used strings like
`"file(s)"`. Both look unnatural and should be avoided. First
tries to solve the problem correctly looked like this:

```
   if (n == 1)
     printf ("%d file deleted", n);
   else
     printf ("%d files deleted", n);
```

But this does not solve the problem. It helps languages where the
plural form of a noun is not simply constructed by adding an
‘s’
but that is all. Once again people fell into the trap of believing the
rules their language is using are universal. But the handling of plural
forms differs widely between the language families. For example,
Rafal Maszkowski `<rzm@mat.uni.torun.pl>` reports:

> In Polish we use e.g. plik (file) this way:
>
> ```
> 1 plik
> 2,3,4 pliki
> 5-21 pliko'w
> 22-24 pliki
> 25-31 pliko'w
> ```
>
> and so on (o' means 8859-2 oacute which should be rather okreska,
> similar to aogonek).

There are two things which can differ between languages (and even inside
language families);

- The form how plural forms are built differs. This is a problem with
  languages which have many irregularities. German, for instance, is a
  drastic case. Though English and German are part of the same language
  family (Germanic), the almost regular forming of plural noun forms
  (appending an
  ‘s’)
  is hardly found in German.- The number of plural forms differ. This is somewhat surprising for
    those who only have experiences with Romanic and Germanic languages
    since here the number is the same (there are two).
    But other language families have only one form or many forms. More
    information on this in an extra section.

The consequence of this is that application writers should not try to
solve the problem in their code. This would be localization since it is
only usable for certain, hardcoded language environments. Instead the
extended `gettext` interface should be used.

These extra functions are taking instead of the one key string two
strings and a numerical argument. The idea behind this is that using
the numerical argument and the first string as a key, the implementation
can select using rules specified by the translator the right plural
form. The two string arguments then will be used to provide a return
value in case no message catalog is found (similar to the normal
`gettext` behavior). In this case the rules for Germanic language
is used and it is assumed that the first string argument is the singular
form, the second the plural form.

This has the consequence that programs without language catalogs can
display the correct strings only if the program itself is written using
a Germanic language. This is a limitation but since the GNU C library
(as well as the GNU `gettext` package) are written as part of the
GNU package and the coding standards for the GNU project require program
being written in English, this solution nevertheless fulfills its
purpose.

Function: char \* **ngettext** *(const char \*msgid1, const char \*msgid2, unsigned long int n)*: The `ngettext` function is similar to the `gettext` function as it finds the message catalogs in the same way. But it takes two extra arguments. The msgid1 parameter must contain the singular form of the string to be converted. It is also used as the key for the search in the catalog. The msgid2 parameter is the plural form. The parameter n is used to determine the plural form. If no message catalog is found msgid1 is returned if `n == 1`, otherwise `msgid2`.

An example for the use of this function is:

```
printf (ngettext ("%d file removed", "%d files removed", n), n);
```

Please note that the numeric value n has to be passed to the
`printf` function as well. It is not sufficient to pass it only to
`ngettext`.

In the English singular case, the number -- always 1 -- can be replaced with
"one":

```
printf (ngettext ("One file removed", "%d files removed", n), n);
```

This works because the `‘printf’` function discards excess arguments that
are not consumed by the format string.

If this function is meant to yield a format string that takes two or more
arguments, you can not use it like this:

```
printf (ngettext ("%d file removed from directory %s",
                  "%d files removed from directory %s",
                  n, dir),
        n);
```

because in many languages the translators want to replace the `‘%d’`
with an explicit word in the singular case, just like “one” in English,
and C format strings cannot consume the second argument but skip the first
argument. Instead, you have to reorder the arguments so that `‘n’`
comes last:

```
printf (ngettext ("%$2d file removed from directory %$1s",
                  "%$2d files removed from directory %$1s",
                  dir, n),
        n);
```

See section 15.3.1 C Format Strings for details about this argument reordering syntax.

When you know that the value of `n` is within a given range, you can
specify it as a comment directed to the `xgettext` tool. This
information may help translators to use more adequate translations. Like
this:

```
if (days > 7 && days < 14)
  /* xgettext: range: 1..6 */
  printf (ngettext ("one week and one day", "one week and %d days",
                    days - 7),
          days - 7);
```

It is also possible to use this function when the strings don't contain a
cardinal number:

```
puts (ngettext ("Delete the selected file?",
                "Delete the selected files?",
                n));
```

In this case the number n is only used to choose the plural form.

Function: char \* **dngettext** *(const char \*domain, const char \*msgid1, const char \*msgid2, unsigned long int n)*: The `dngettext` is similar to the `dgettext` function in the way the message catalog is selected. The difference is that it takes two extra parameter to provide the correct plural form. These two parameters are handled in the same way `ngettext` handles them.

Function: char \* **dcngettext** *(const char \*domain, const char \*msgid1, const char \*msgid2, unsigned long int n, int category)*: The `dcngettext` is similar to the `dcgettext` function in the way the message catalog is selected. The difference is that it takes two extra parameter to provide the correct plural form. These two parameters are handled in the same way `ngettext` handles them.

Now, how do these functions solve the problem of the plural forms?
Without the input of linguists (which was not available) it was not
possible to determine whether there are only a few different forms in
which plural forms are formed or whether the number can increase with
every new supported language.

Therefore the solution implemented is to allow the translator to specify
the rules of how to select the plural form. Since the formula varies
with every language this is the only viable solution except for
hardcoding the information in the code (which still would require the
possibility of extensions to not prevent the use of new languages).

The information about the plural form selection has to be stored in the
header entry of the PO file (the one with the empty `msgid` string).
The plural form information looks like this:

```
Plural-Forms: nplurals=2; plural=n == 1 ? 0 : 1;
```

The `nplurals` value must be a decimal number which specifies how
many different plural forms exist for this language. The string
following `plural` is an expression which is using the C language
syntax. Exceptions are that no negative numbers are allowed, numbers
must be decimal, and the only variable allowed is `n`. Spaces are
allowed in the expression, but backslash-newlines are not; in the
examples below the backslash-newlines are present for formatting purposes
only. This expression will be evaluated whenever one of the functions
`ngettext`, `dngettext`, or `dcngettext` is called. The
numeric value passed to these functions is then substituted for all uses
of the variable `n` in the expression. The resulting value then
must be greater or equal to zero and smaller than the value given as the
value of `nplurals`.

The following rules are known at this point. The language with families
are listed. But this does not necessarily mean the information can be
generalized for the whole family (as can be easily seen in the table
below).(5)

Only one form:: Some languages only require one single form. There is no distinction between the singular and plural form. An appropriate header entry would look like this: ``` Plural-Forms: nplurals=1; plural=0; ``` Languages with this property include: Asian family: Japanese, Vietnamese, Korean Two forms, singular used for one only: This is the form used in most existing programs since it is what English is using. A header entry would look like this: ``` Plural-Forms: nplurals=2; plural=n != 1; ``` (Note: this uses the feature of C expressions that boolean expressions have to value zero or one.) Languages with this property include: Germanic family: English, German, Dutch, Swedish, Danish, Norwegian, Faroese Romanic family: Spanish, Portuguese, Italian, Bulgarian Latin/Greek family: Greek Finno-Ugric family: Finnish, Estonian Semitic family: Hebrew Artificial: Esperanto Other languages using the same header entry are: Finno-Ugric family: Hungarian Turkic/Altaic family: Turkish Hungarian does not appear to have a plural if you look at sentences involving cardinal numbers. For example, “1 apple” is “1 alma”, and “123 apples” is “123 alma”. But when the number is not explicit, the distinction between singular and plural exists: “the apple” is “az alma”, and “the apples” is “az alm'{a}k”. Since `ngettext` has to support both types of sentences, it is classified here, under “two forms”. The same holds for Turkish: “1 apple” is “1 elma”, and “123 apples” is “123 elma”. But when the number is omitted, the distinction between singular and plural exists: “the apple” is “elma”, and “the apples” is “elmalar”. Two forms, singular used for zero and one: Exceptional case in the language family. The header entry would be: ``` Plural-Forms: nplurals=2; plural=n>1; ``` Languages with this property include: Romanic family: Brazilian Portuguese, French Three forms, special case for zero: The header entry would be: ``` Plural-Forms: nplurals=3; plural=n%10==1 && n%100!=11 ? 0 : n != 0 ? 1 : 2; ``` Languages with this property include: Baltic family: Latvian Three forms, special cases for one and two: The header entry would be: ``` Plural-Forms: nplurals=3; plural=n==1 ? 0 : n==2 ? 1 : 2; ``` Languages with this property include: Celtic: Gaeilge (Irish) Three forms, special case for numbers ending in 00 or [2-9][0-9]: The header entry would be: ``` Plural-Forms: nplurals=3; \ plural=n==1 ? 0 : (n==0 || (n%100 > 0 && n%100 < 20)) ? 1 : 2; ``` Languages with this property include: Romanic family: Romanian Three forms, special case for numbers ending in 1[2-9]: The header entry would look like this: ``` Plural-Forms: nplurals=3; \ plural=n%10==1 && n%100!=11 ? 0 : \ n%10>=2 && (n%100<10 || n%100>=20) ? 1 : 2; ``` Languages with this property include: Baltic family: Lithuanian Three forms, special cases for numbers ending in 1 and 2, 3, 4, except those ending in 1[1-4]: The header entry would look like this: ``` Plural-Forms: nplurals=3; \ plural=n%10==1 && n%100!=11 ? 0 : \ n%10>=2 && n%10<=4 && (n%100<10 || n%100>=20) ? 1 : 2; ``` Languages with this property include: Slavic family: Russian, Ukrainian, Serbian, Croatian Three forms, special cases for 1 and 2, 3, 4: The header entry would look like this: ``` Plural-Forms: nplurals=3; \ plural=(n==1) ? 0 : (n>=2 && n<=4) ? 1 : 2; ``` Languages with this property include: Slavic family: Czech, Slovak Three forms, special case for one and some numbers ending in 2, 3, or 4: The header entry would look like this: ``` Plural-Forms: nplurals=3; \ plural=n==1 ? 0 : \ n%10>=2 && n%10<=4 && (n%100<10 || n%100>=20) ? 1 : 2; ``` Languages with this property include: Slavic family: Polish Four forms, special case for one and all numbers ending in 02, 03, or 04: The header entry would look like this: ``` Plural-Forms: nplurals=4; \ plural=n%100==1 ? 0 : n%100==2 ? 1 : n%100==3 || n%100==4 ? 2 : 3; ``` Languages with this property include: Slavic family: Slovenian

You might now ask, `ngettext` handles only numbers n of type
`‘unsigned long’`. What about larger integer types? What about negative
numbers? What about floating-point numbers?

About larger integer types, such as `‘uintmax_t’` or
`‘unsigned long long’`: they can be handled by reducing the value to a
range that fits in an `‘unsigned long’`. Simply casting the value to
`‘unsigned long’` would not do the right thing, since it would treat
`ULONG_MAX + 1` like zero, `ULONG_MAX + 2` like singular, and
the like. Here you can exploit the fact that all mentioned plural form
formulas eventually become periodic, with a period that is a divisor of 100
(or 1000 or 1000000). So, when you reduce a large value to another one in
the range [1000000, 1999999] that ends in the same 6 decimal digits, you
can assume that it will lead to the same plural form selection. This code
does this:

```
#include <inttypes.h>
uintmax_t nbytes = ...;
printf (ngettext ("The file has %"PRIuMAX" byte.",
                  "The file has %"PRIuMAX" bytes.",
                  (nbytes > ULONG_MAX
                   ? (nbytes % 1000000) + 1000000
                   : nbytes)),
        nbytes);
```

Negative and floating-point values usually represent physical entities for
which singular and plural don't clearly apply. In such cases, there is no
need to use `ngettext`; a simple `gettext` call with a form suitable
for all values will do. For example:

```
printf (gettext ("Time elapsed: %.3f seconds"),
        num_milliseconds * 0.001);
```

Even if num\_milliseconds happens to be a multiple of 1000, the output

```
Time elapsed: 1.000 seconds
```

is acceptable in English, and similarly for other languages.

The translators' perspective regarding plural forms is explained in
section 12.6 Translating plural forms.

### 11.2.7 Optimization of the \*gettext functions

At this point of the discussion we should talk about an advantage of the
GNU `gettext` implementation. Some readers might have pointed out
that an internationalized program might have a poor performance if some
string has to be translated in an inner loop. While this is unavoidable
when the string varies from one run of the loop to the other it is
simply a waste of time when the string is always the same. Take the
following example:

```
{
  while (...)
    {
      puts (gettext ("Hello world"));
    }
}
```

When the locale selection does not change between two runs the resulting
string is always the same. One way to use this is:

```
{
  str = gettext ("Hello world");
  while (...)
    {
      puts (str);
    }
}
```

But this solution is not usable in all situation (e.g. when the locale
selection changes) nor does it lead to legible code.

For this reason, GNU `gettext` caches previous translation results.
When the same translation is requested twice, with no new message
catalogs being loaded in between, `gettext` will, the second time,
find the result through a single cache lookup.

## 11.3 Comparing the Two Interfaces

The following discussion is perhaps a little bit colored. As said
above we implemented GNU `gettext` following the Uniforum
proposal and this surely has its reasons. But it should show how we
came to this decision.

First we take a look at the developing process. When we write an
application using NLS provided by `gettext` we proceed as always.
Only when we come to a string which might be seen by the users and thus
has to be translated we use `gettext("...")` instead of
`"..."`. At the beginning of each source file (or in a central
header file) we define

```
#define gettext(String) (String)
```

Even this definition can be avoided when the system supports the
`gettext` function in its C library. When we compile this code the
result is the same as if no NLS code is used. When you take a look at
the GNU `gettext` code you will see that we use `_("...")`
instead of `gettext("...")`. This reduces the number of
additional characters per translatable string to *3* (in words:
three).

When now a production version of the program is needed we simply replace
the definition

```
#define _(String) (String)
```

by

```
#include <libintl.h>
#define _(String) gettext (String)
```

Additionally we run the program ‘xgettext’ on all source code file
which contain translatable strings and that's it: we have a running
program which does not depend on translations to be available, but which
can use any that becomes available.

The same procedure can be done for the `gettext_noop` invocations
(see section 4.7 Special Cases of Translatable Strings). One usually defines `gettext_noop` as a
no-op macro. So you should consider the following code for your project:

```
#define gettext_noop(String) String
#define N_(String) gettext_noop (String)
```

`N_` is a short form similar to `_`. The ‘Makefile’ in
the ‘po/’ directory of GNU `gettext` knows by default both of the
mentioned short forms so you are invited to follow this proposal for
your own ease.

Now to `catgets`. The main problem is the work for the
programmer. Every time he comes to a translatable string he has to
define a number (or a symbolic constant) which has also be defined in
the message catalog file. He also has to take care for duplicate
entries, duplicate message IDs etc. If he wants to have the same
quality in the message catalog as the GNU `gettext` program
provides he also has to put the descriptive comments for the strings and
the location in all source code files in the message catalog. This is
nearly a Mission: Impossible.

But there are also some points people might call advantages speaking for
`catgets`. If you have a single word in a string and this string
is used in different contexts it is likely that in one or the other
language the word has different translations. Example:

```
printf ("%s: %d", gettext ("number"), number_of_errors)

printf ("you should see %d %s", number_count,
        number_count == 1 ? gettext ("number") : gettext ("numbers"))
```

Here we have to translate two times the string `"number"`. Even
if you do not speak a language beside English it might be possible to
recognize that the two words have a different meaning. In German the
first appearance has to be translated to `"Anzahl"` and the second
to `"Zahl"`.

Now you can say that this example is really esoteric. And you are
right! This is exactly how we felt about this problem and decide that
it does not weight that much. The solution for the above problem could
be very easy:

```
printf ("%s %d", gettext ("number:"), number_of_errors)

printf (number_count == 1 ? gettext ("you should see %d number")
                          : gettext ("you should see %d numbers"),
        number_count)
```

We believe that we can solve all conflicts with this method. If it is
difficult one can also consider changing one of the conflicting string a
little bit. But it is not impossible to overcome.

`catgets` allows same original entry to have different translations,
but `gettext` has another, scalable approach for solving ambiguities
of this kind: See section 11.2.2 Solving Ambiguities.

## 11.4 Using libintl.a in own programs

Starting with version 0.9.4 the library `libintl.h` should be
self-contained. I.e., you can use it in your own programs without
providing additional functions. The ‘Makefile’ will put the header
and the library in directories selected using the `$(prefix)`.

## 11.5 Being a `gettext` grok

**NOTE:**  This documentation section is outdated and needs to be
revised.

To fully exploit the functionality of the GNU `gettext` library it
is surely helpful to read the source code. But for those who don't want
to spend that much time in reading the (sometimes complicated) code here
is a list comments:

- Changing the language at runtime
  For interactive programs it might be useful to offer a selection of the
  used language at runtime. To understand how to do this one need to know
  how the used language is determined while executing the `gettext`
  function. The method which is presented here only works correctly
  with the GNU implementation of the `gettext` functions.
  In the function `dcgettext` at every call the current setting of
  the highest priority environment variable is determined and used.
  Highest priority means here the following list with decreasing
  priority:
  1. `LANGUAGE`

     - `LC_ALL`


       - `LC_xxx`, according to selected locale category
         - `LANG`
  Afterwards the path is constructed using the found value and the
  translation file is loaded if available.
  What happens now when the value for, say, `LANGUAGE` changes? According
  to the process explained above the new value of this variable is found
  as soon as the `dcgettext` function is called. But this also means
  the (perhaps) different message catalog file is loaded. In other
  words: the used language is changed.
  But there is one little hook. The code for gcc-2.7.0 and up provides
  some optimization. This optimization normally prevents the calling of
  the `dcgettext` function as long as no new catalog is loaded. But
  if `dcgettext` is not called the program also cannot find the
  `LANGUAGE` variable be changed (see section 11.2.7 Optimization of the \*gettext functions). A
  solution for this is very easy. Include the following code in the
  language switching function.

  ```
    /* Change language.  */
    setenv ("LANGUAGE", "fr", 1);

    /* Make change known.  */
    {
      extern int  _nl_msg_cat_cntr;
      ++_nl_msg_cat_cntr;
    }
  ```

  The variable `_nl_msg_cat_cntr` is defined in ‘loadmsgcat.c’.
  You don't need to know what this is for. But it can be used to detect
  whether a `gettext` implementation is GNU gettext and not non-GNU
  system's native gettext implementation.

## 11.6 Temporary Notes for the Programmers Chapter

**NOTE:**  This documentation section is outdated and needs to be
revised.

### 11.6.1 Temporary - Two Possible Implementations

There are two competing methods for language independent messages:
the X/Open `catgets` method, and the Uniforum `gettext`
method. The `catgets` method indexes messages by integers; the
`gettext` method indexes them by their English translations.
The `catgets` method has been around longer and is supported
by more vendors. The `gettext` method is supported by Sun,
and it has been heard that the COSE multi-vendor initiative is
supporting it. Neither method is a POSIX standard; the POSIX.1
committee had a lot of disagreement in this area.

Neither one is in the POSIX standard. There was much disagreement
in the POSIX.1 committee about using the `gettext` routines
vs. `catgets` (XPG). In the end the committee couldn't
agree on anything, so no messaging system was included as part
of the standard. I believe the informative annex of the standard
includes the XPG3 messaging interfaces, “...as an example of
a messaging system that has been implemented...”

They were very careful not to say anywhere that you should use one
set of interfaces over the other. For more on this topic please
see the Programming for Internationalization FAQ.

### 11.6.2 Temporary - About `catgets`

There have been a few discussions of late on the use of
`catgets` as a base. I think it important to present both
sides of the argument and hence am opting to play devil's advocate
for a little bit.

I'll not deny the fact that `catgets` could have been designed
a lot better. It currently has quite a number of limitations and
these have already been pointed out.

However there is a great deal to be said for consistency and
standardization. A common recurring problem when writing Unix
software is the myriad portability problems across Unix platforms.
It seems as if every Unix vendor had a look at the operating system
and found parts they could improve upon. Undoubtedly, these
modifications are probably innovative and solve real problems.
However, software developers have a hard time keeping up with all
these changes across so many platforms.

And this has prompted the Unix vendors to begin to standardize their
systems. Hence the impetus for Spec1170. Every major Unix vendor
has committed to supporting this standard and every Unix software
developer waits with glee the day they can write software to this
standard and simply recompile (without having to use autoconf)
across different platforms.

As I understand it, Spec1170 is roughly based upon version 4 of the
X/Open Portability Guidelines (XPG4). Because `catgets` and
friends are defined in XPG4, I'm led to believe that `catgets`
is a part of Spec1170 and hence will become a standardized component
of all Unix systems.

### 11.6.3 Temporary - Why a single implementation

Now it seems kind of wasteful to me to have two different systems
installed for accessing message catalogs. If we do want to remedy
`catgets` deficiencies why don't we try to expand `catgets`
(in a compatible manner) rather than implement an entirely new system.
Otherwise, we'll end up with two message catalog access systems installed
with an operating system - one set of routines for packages using GNU
`gettext` for their internationalization, and another set of routines
(catgets) for all other software. Bloated?

Supposing another catalog access system is implemented. Which do
we recommend? At least for Linux, we need to attract as many
software developers as possible. Hence we need to make it as easy
for them to port their software as possible. Which means supporting
`catgets`. We will be implementing the `libintl` code
within our `libc`, but does this mean we also have to incorporate
another message catalog access scheme within our `libc` as well?
And what about people who are going to be using the `libintl`
+ non-`catgets` routines. When they port their software to
other platforms, they're now going to have to include the front-end
(`libintl`) code plus the back-end code (the non-`catgets`
access routines) with their software instead of just including the
`libintl` code with their software.

Message catalog support is however only the tip of the iceberg.
What about the data for the other locale categories? They also have
a number of deficiencies. Are we going to abandon them as well and
develop another duplicate set of routines (should `libintl`
expand beyond message catalog support)?

Like many parts of Unix that can be improved upon, we're stuck with balancing
compatibility with the past with useful improvements and innovations for
the future.

### 11.6.4 Temporary - Notes

X/Open agreed very late on the standard form so that many
implementations differ from the final form. Both of my system (old
Linux catgets and Ultrix-4) have a strange variation.

OK. After incorporating the last changes I have to spend some time on
making the GNU/Linux `libc` `gettext` functions. So in future
Solaris is not the only system having `gettext`.

---

Go to the first, previous, next, last section, table of contents.
