# Supplementary material for: rDock: A Fast, Versatile and Open Source Program for Docking Ligands to Proteins and Nucleic Acids
Source: PLoS Comput Biol. 2014 Apr 10;10(4):e1003571. doi: 10.1371/journal.pcbi.1003571 (PMC3983074; doi:10.1371/journal.pcbi.1003571)
Supplement: Software S1 — Compressed file with the source code of the rDock software for ligand docking to Proteins and Nucleic Acids. (GZ) [file pcbi.1003571.s010.gz › rDock_2013.1_src/gettext-0.18.tar.gz!/gettext-0.18/gettext-tools/doc/gettext_19.html]

GNU gettext utilities - C Licenses

Go to the first, previous, next, last section, table of contents.

---

# C Licenses

The files of this package are covered by the licenses indicated in each
particular file or directory. Here is a summary:

- The `libintl` and `libasprintf` libraries are covered by the
  GNU Library General Public License (LGPL).
  A copy of the license is included in section C.2 GNU LESSER GENERAL PUBLIC LICENSE.- The executable programs of this package and the `libgettextpo` library
    are covered by the GNU General Public License (GPL).
    A copy of the license is included in section C.1 GNU GENERAL PUBLIC LICENSE.- This manual is free documentation. It is dually licensed under the
      GNU FDL and the GNU GPL. This means that you can redistribute this
      manual under either of these two licenses, at your choice.
        
      This manual is covered by the GNU FDL. Permission is granted to copy,
      distribute and/or modify this document under the terms of the
      GNU Free Documentation License (FDL), either version 1.2 of the
      License, or (at your option) any later version published by the
      Free Software Foundation (FSF); with no Invariant Sections, with no
      Front-Cover Text, and with no Back-Cover Texts.
      A copy of the license is included in section C.3 GNU Free Documentation License.
        
      This manual is covered by the GNU GPL. You can redistribute it and/or
      modify it under the terms of the GNU General Public License (GPL), either
      version 2 of the License, or (at your option) any later version published
      by the Free Software Foundation (FSF).
      A copy of the license is included in section C.1 GNU GENERAL PUBLIC LICENSE.

## C.1 GNU GENERAL PUBLIC LICENSE

Version 2, June 1991

```
Copyright (C) 1989, 1991 Free Software Foundation, Inc.
51 Franklin Street, Fifth Floor, Boston, MA  02110-1301, USA

Everyone is permitted to copy and distribute verbatim copies
of this license document, but changing it is not allowed.
```

### Preamble

The licenses for most software are designed to take away your
freedom to share and change it. By contrast, the GNU General Public
License is intended to guarantee your freedom to share and change free
software--to make sure the software is free for all its users. This
General Public License applies to most of the Free Software
Foundation's software and to any other program whose authors commit to
using it. (Some other Free Software Foundation software is covered by
the GNU Library General Public License instead.) You can apply it to
your programs, too.

When we speak of free software, we are referring to freedom, not
price. Our General Public Licenses are designed to make sure that you
have the freedom to distribute copies of free software (and charge for
this service if you wish), that you receive source code or can get it
if you want it, that you can change the software or use pieces of it
in new free programs; and that you know you can do these things.

To protect your rights, we need to make restrictions that forbid
anyone to deny you these rights or to ask you to surrender the rights.
These restrictions translate to certain responsibilities for you if you
distribute copies of the software, or if you modify it.

For example, if you distribute copies of such a program, whether
gratis or for a fee, you must give the recipients all the rights that
you have. You must make sure that they, too, receive or can get the
source code. And you must show them these terms so they know their
rights.

We protect your rights with two steps: (1) copyright the software, and
(2) offer you this license which gives you legal permission to copy,
distribute and/or modify the software.

Also, for each author's protection and ours, we want to make certain
that everyone understands that there is no warranty for this free
software. If the software is modified by someone else and passed on, we
want its recipients to know that what they have is not the original, so
that any problems introduced by others will not reflect on the original
authors' reputations.

Finally, any free program is threatened constantly by software
patents. We wish to avoid the danger that redistributors of a free
program will individually obtain patent licenses, in effect making the
program proprietary. To prevent this, we have made it clear that any
patent must be licensed for everyone's free use or not licensed at all.

The precise terms and conditions for copying, distribution and
modification follow.

1. This License applies to any program or other work which contains
   a notice placed by the copyright holder saying it may be distributed
   under the terms of this General Public License. The “Program”, below,
   refers to any such program or work, and a “work based on the Program”
   means either the Program or any derivative work under copyright law:
   that is to say, a work containing the Program or a portion of it,
   either verbatim or with modifications and/or translated into another
   language. (Hereinafter, translation is included without limitation in
   the term “modification”.) Each licensee is addressed as “you”.
   Activities other than copying, distribution and modification are not
   covered by this License; they are outside its scope. The act of
   running the Program is not restricted, and the output from the Program
   is covered only if its contents constitute a work based on the
   Program (independent of having been made by running the Program).
   Whether that is true depends on what the Program does.- You may copy and distribute verbatim copies of the Program's
     source code as you receive it, in any medium, provided that you
     conspicuously and appropriately publish on each copy an appropriate
     copyright notice and disclaimer of warranty; keep intact all the
     notices that refer to this License and to the absence of any warranty;
     and give any other recipients of the Program a copy of this License
     along with the Program.
     You may charge a fee for the physical act of transferring a copy, and
     you may at your option offer warranty protection in exchange for a fee.- You may modify your copy or copies of the Program or any portion
       of it, thus forming a work based on the Program, and copy and
       distribute such modifications or work under the terms of Section 1
       above, provided that you also meet all of these conditions:
       1. You must cause the modified files to carry prominent notices
          stating that you changed the files and the date of any change.- You must cause any work that you distribute or publish, that in
            whole or in part contains or is derived from the Program or any
            part thereof, to be licensed as a whole at no charge to all third
            parties under the terms of this License.- If the modified program normally reads commands interactively
              when run, you must cause it, when started running for such
              interactive use in the most ordinary way, to print or display an
              announcement including an appropriate copyright notice and a
              notice that there is no warranty (or else, saying that you provide
              a warranty) and that users may redistribute the program under
              these conditions, and telling the user how to view a copy of this
              License. (Exception: if the Program itself is interactive but
              does not normally print such an announcement, your work based on
              the Program is not required to print an announcement.)
       These requirements apply to the modified work as a whole. If
       identifiable sections of that work are not derived from the Program,
       and can be reasonably considered independent and separate works in
       themselves, then this License, and its terms, do not apply to those
       sections when you distribute them as separate works. But when you
       distribute the same sections as part of a whole which is a work based
       on the Program, the distribution of the whole must be on the terms of
       this License, whose permissions for other licensees extend to the
       entire whole, and thus to each and every part regardless of who wrote it.
       Thus, it is not the intent of this section to claim rights or contest
       your rights to work written entirely by you; rather, the intent is to
       exercise the right to control the distribution of derivative or
       collective works based on the Program.
       In addition, mere aggregation of another work not based on the Program
       with the Program (or with a work based on the Program) on a volume of
       a storage or distribution medium does not bring the other work under
       the scope of this License.- You may copy and distribute the Program (or a work based on it,
         under Section 2) in object code or executable form under the terms of
         Sections 1 and 2 above provided that you also do one of the following:
         1. Accompany it with the complete corresponding machine-readable
            source code, which must be distributed under the terms of Sections
            1 and 2 above on a medium customarily used for software interchange; or,- Accompany it with a written offer, valid for at least three
              years, to give any third party, for a charge no more than your
              cost of physically performing source distribution, a complete
              machine-readable copy of the corresponding source code, to be
              distributed under the terms of Sections 1 and 2 above on a medium
              customarily used for software interchange; or,- Accompany it with the information you received as to the offer
                to distribute corresponding source code. (This alternative is
                allowed only for noncommercial distribution and only if you
                received the program in object code or executable form with such
                an offer, in accord with Subsection b above.)
         The source code for a work means the preferred form of the work for
         making modifications to it. For an executable work, complete source
         code means all the source code for all modules it contains, plus any
         associated interface definition files, plus the scripts used to
         control compilation and installation of the executable. However, as a
         special exception, the source code distributed need not include
         anything that is normally distributed (in either source or binary
         form) with the major components (compiler, kernel, and so on) of the
         operating system on which the executable runs, unless that component
         itself accompanies the executable.
         If distribution of executable or object code is made by offering
         access to copy from a designated place, then offering equivalent
         access to copy the source code from the same place counts as
         distribution of the source code, even though third parties are not
         compelled to copy the source along with the object code.- You may not copy, modify, sublicense, or distribute the Program
           except as expressly provided under this License. Any attempt
           otherwise to copy, modify, sublicense or distribute the Program is
           void, and will automatically terminate your rights under this License.
           However, parties who have received copies, or rights, from you under
           this License will not have their licenses terminated so long as such
           parties remain in full compliance.- You are not required to accept this License, since you have not
             signed it. However, nothing else grants you permission to modify or
             distribute the Program or its derivative works. These actions are
             prohibited by law if you do not accept this License. Therefore, by
             modifying or distributing the Program (or any work based on the
             Program), you indicate your acceptance of this License to do so, and
             all its terms and conditions for copying, distributing or modifying
             the Program or works based on it.- Each time you redistribute the Program (or any work based on the
               Program), the recipient automatically receives a license from the
               original licensor to copy, distribute or modify the Program subject to
               these terms and conditions. You may not impose any further
               restrictions on the recipients' exercise of the rights granted herein.
               You are not responsible for enforcing compliance by third parties to
               this License.- If, as a consequence of a court judgment or allegation of patent
                 infringement or for any other reason (not limited to patent issues),
                 conditions are imposed on you (whether by court order, agreement or
                 otherwise) that contradict the conditions of this License, they do not
                 excuse you from the conditions of this License. If you cannot
                 distribute so as to satisfy simultaneously your obligations under this
                 License and any other pertinent obligations, then as a consequence you
                 may not distribute the Program at all. For example, if a patent
                 license would not permit royalty-free redistribution of the Program by
                 all those who receive copies directly or indirectly through you, then
                 the only way you could satisfy both it and this License would be to
                 refrain entirely from distribution of the Program.
                 If any portion of this section is held invalid or unenforceable under
                 any particular circumstance, the balance of the section is intended to
                 apply and the section as a whole is intended to apply in other
                 circumstances.
                 It is not the purpose of this section to induce you to infringe any
                 patents or other property right claims or to contest validity of any
                 such claims; this section has the sole purpose of protecting the
                 integrity of the free software distribution system, which is
                 implemented by public license practices. Many people have made
                 generous contributions to the wide range of software distributed
                 through that system in reliance on consistent application of that
                 system; it is up to the author/donor to decide if he or she is willing
                 to distribute software through any other system and a licensee cannot
                 impose that choice.
                 This section is intended to make thoroughly clear what is believed to
                 be a consequence of the rest of this License.- If the distribution and/or use of the Program is restricted in
                   certain countries either by patents or by copyrighted interfaces, the
                   original copyright holder who places the Program under this License
                   may add an explicit geographical distribution limitation excluding
                   those countries, so that distribution is permitted only in or among
                   countries not thus excluded. In such case, this License incorporates
                   the limitation as if written in the body of this License.- The Free Software Foundation may publish revised and/or new versions
                     of the General Public License from time to time. Such new versions will
                     be similar in spirit to the present version, but may differ in detail to
                     address new problems or concerns.
                     Each version is given a distinguishing version number. If the Program
                     specifies a version number of this License which applies to it and “any
                     later version”, you have the option of following the terms and conditions
                     either of that version or of any later version published by the Free
                     Software Foundation. If the Program does not specify a version number of
                     this License, you may choose any version ever published by the Free Software
                     Foundation.- If you wish to incorporate parts of the Program into other free
                       programs whose distribution conditions are different, write to the author
                       to ask for permission. For software which is copyrighted by the Free
                       Software Foundation, write to the Free Software Foundation; we sometimes
                       make exceptions for this. Our decision will be guided by the two goals
                       of preserving the free status of all derivatives of our free software and
                       of promoting the sharing and reuse of software generally.- BECAUSE THE PROGRAM IS LICENSED FREE OF CHARGE, THERE IS NO WARRANTY
                         FOR THE PROGRAM, TO THE EXTENT PERMITTED BY APPLICABLE LAW. EXCEPT WHEN
                         OTHERWISE STATED IN WRITING THE COPYRIGHT HOLDERS AND/OR OTHER PARTIES
                         PROVIDE THE PROGRAM “AS IS” WITHOUT WARRANTY OF ANY KIND, EITHER EXPRESSED
                         OR IMPLIED, INCLUDING, BUT NOT LIMITED TO, THE IMPLIED WARRANTIES OF
                         MERCHANTABILITY AND FITNESS FOR A PARTICULAR PURPOSE. THE ENTIRE RISK AS
                         TO THE QUALITY AND PERFORMANCE OF THE PROGRAM IS WITH YOU. SHOULD THE
                         PROGRAM PROVE DEFECTIVE, YOU ASSUME THE COST OF ALL NECESSARY SERVICING,
                         REPAIR OR CORRECTION.- IN NO EVENT UNLESS REQUIRED BY APPLICABLE LAW OR AGREED TO IN WRITING
                           WILL ANY COPYRIGHT HOLDER, OR ANY OTHER PARTY WHO MAY MODIFY AND/OR
                           REDISTRIBUTE THE PROGRAM AS PERMITTED ABOVE, BE LIABLE TO YOU FOR DAMAGES,
                           INCLUDING ANY GENERAL, SPECIAL, INCIDENTAL OR CONSEQUENTIAL DAMAGES ARISING
                           OUT OF THE USE OR INABILITY TO USE THE PROGRAM (INCLUDING BUT NOT LIMITED
                           TO LOSS OF DATA OR DATA BEING RENDERED INACCURATE OR LOSSES SUSTAINED BY
                           YOU OR THIRD PARTIES OR A FAILURE OF THE PROGRAM TO OPERATE WITH ANY OTHER
                           PROGRAMS), EVEN IF SUCH HOLDER OR OTHER PARTY HAS BEEN ADVISED OF THE
                           POSSIBILITY OF SUCH DAMAGES.

### Appendix: How to Apply These Terms to Your New Programs

If you develop a new program, and you want it to be of the greatest
possible use to the public, the best way to achieve this is to make it
free software which everyone can redistribute and change under these terms.

To do so, attach the following notices to the program. It is safest
to attach them to the start of each source file to most effectively
convey the exclusion of warranty; and each file should have at least
the “copyright” line and a pointer to where the full notice is found.

```
one line to give the program's name and a brief idea of what it does.
Copyright (C) yyyy  name of author

This program is free software; you can redistribute it and/or modify
it under the terms of the GNU General Public License as published by
the Free Software Foundation; either version 2 of the License, or
(at your option) any later version.

This program is distributed in the hope that it will be useful,
but WITHOUT ANY WARRANTY; without even the implied warranty of
MERCHANTABILITY or FITNESS FOR A PARTICULAR PURPOSE.  See the
GNU General Public License for more details.

You should have received a copy of the GNU General Public License
along with this program; if not, write to the Free Software
Foundation, Inc., 51 Franklin Street, Fifth Floor, Boston, MA  02110-1301, USA.
```

Also add information on how to contact you by electronic and paper mail.

If the program is interactive, make it output a short notice like this
when it starts in an interactive mode:

```
Gnomovision version 69, Copyright (C) 19yy name of author
Gnomovision comes with ABSOLUTELY NO WARRANTY; for details type `show w'.
This is free software, and you are welcome to redistribute it
under certain conditions; type `show c' for details.
```

The hypothetical commands `‘show w’` and `‘show c’` should show
the appropriate parts of the General Public License. Of course, the
commands you use may be called something other than `‘show w’` and
`‘show c’`; they could even be mouse-clicks or menu items--whatever
suits your program.

You should also get your employer (if you work as a programmer) or your
school, if any, to sign a “copyright disclaimer” for the program, if
necessary. Here is a sample; alter the names:

```
Yoyodyne, Inc., hereby disclaims all copyright interest in the program
`Gnomovision' (which makes passes at compilers) written by James Hacker.

signature of Ty Coon, 1 April 1989
Ty Coon, President of Vice
```

This General Public License does not permit incorporating your program into
proprietary programs. If your program is a subroutine library, you may
consider it more useful to permit linking proprietary applications with the
library. If this is what you want to do, use the GNU Library General
Public License instead of this License.

## C.2 GNU LESSER GENERAL PUBLIC LICENSE

Version 2.1, February 1999

```
Copyright (C) 1991, 1999 Free Software Foundation, Inc.
51 Franklin St -- Fifth Floor, Boston, MA 02110-1301, USA

Everyone is permitted to copy and distribute verbatim copies
of this license document, but changing it is not allowed.

[This is the first released version of the Lesser GPL.  It also counts
as the successor of the GNU Library Public License, version 2, hence the
version number 2.1.]
```

### Preamble

The licenses for most software are designed to take away your
freedom to share and change it. By contrast, the GNU General Public
Licenses are intended to guarantee your freedom to share and change
free software--to make sure the software is free for all its users.

This license, the Lesser General Public License, applies to some
specially designated software--typically libraries--of the Free
Software Foundation and other authors who decide to use it. You can use
it too, but we suggest you first think carefully about whether this
license or the ordinary General Public License is the better strategy to
use in any particular case, based on the explanations below.

When we speak of free software, we are referring to freedom of use,
not price. Our General Public Licenses are designed to make sure that
you have the freedom to distribute copies of free software (and charge
for this service if you wish); that you receive source code or can get
it if you want it; that you can change the software and use pieces of it
in new free programs; and that you are informed that you can do these
things.

To protect your rights, we need to make restrictions that forbid
distributors to deny you these rights or to ask you to surrender these
rights. These restrictions translate to certain responsibilities for
you if you distribute copies of the library or if you modify it.

For example, if you distribute copies of the library, whether gratis
or for a fee, you must give the recipients all the rights that we gave
you. You must make sure that they, too, receive or can get the source
code. If you link other code with the library, you must provide
complete object files to the recipients, so that they can relink them
with the library after making changes to the library and recompiling
it. And you must show them these terms so they know their rights.

We protect your rights with a two-step method: (1) we copyright the
library, and (2) we offer you this license, which gives you legal
permission to copy, distribute and/or modify the library.

To protect each distributor, we want to make it very clear that
there is no warranty for the free library. Also, if the library is
modified by someone else and passed on, the recipients should know
that what they have is not the original version, so that the original
author's reputation will not be affected by problems that might be
introduced by others.

Finally, software patents pose a constant threat to the existence of
any free program. We wish to make sure that a company cannot
effectively restrict the users of a free program by obtaining a
restrictive license from a patent holder. Therefore, we insist that
any patent license obtained for a version of the library must be
consistent with the full freedom of use specified in this license.

Most GNU software, including some libraries, is covered by the
ordinary GNU General Public License. This license, the GNU Lesser
General Public License, applies to certain designated libraries, and
is quite different from the ordinary General Public License. We use
this license for certain libraries in order to permit linking those
libraries into non-free programs.

When a program is linked with a library, whether statically or using
a shared library, the combination of the two is legally speaking a
combined work, a derivative of the original library. The ordinary
General Public License therefore permits such linking only if the
entire combination fits its criteria of freedom. The Lesser General
Public License permits more lax criteria for linking other code with
the library.

We call this license the *Lesser* General Public License because it
does *Less* to protect the user's freedom than the ordinary General
Public License. It also provides other free software developers Less
of an advantage over competing non-free programs. These disadvantages
are the reason we use the ordinary General Public License for many
libraries. However, the Lesser license provides advantages in certain
special circumstances.

For example, on rare occasions, there may be a special need to
encourage the widest possible use of a certain library, so that it becomes
a de-facto standard. To achieve this, non-free programs must be
allowed to use the library. A more frequent case is that a free
library does the same job as widely used non-free libraries. In this
case, there is little to gain by limiting the free library to free
software only, so we use the Lesser General Public License.

In other cases, permission to use a particular library in non-free
programs enables a greater number of people to use a large body of
free software. For example, permission to use the GNU C Library in
non-free programs enables many more people to use the whole GNU
operating system, as well as its variant, the GNU/Linux operating
system.

Although the Lesser General Public License is Less protective of the
users' freedom, it does ensure that the user of a program that is
linked with the Library has the freedom and the wherewithal to run
that program using a modified version of the Library.

The precise terms and conditions for copying, distribution and
modification follow. Pay close attention to the difference between a
“work based on the library” and a “work that uses the library”. The
former contains code derived from the library, whereas the latter must
be combined with the library in order to run.

1. This License Agreement applies to any software library or other program
   which contains a notice placed by the copyright holder or other
   authorized party saying it may be distributed under the terms of this
   Lesser General Public License (also called “this License”). Each
   licensee is addressed as “you”.
   A “library” means a collection of software functions and/or data
   prepared so as to be conveniently linked with application programs
   (which use some of those functions and data) to form executables.
   The “Library”, below, refers to any such software library or work
   which has been distributed under these terms. A “work based on the
   Library” means either the Library or any derivative work under
   copyright law: that is to say, a work containing the Library or a
   portion of it, either verbatim or with modifications and/or translated
   straightforwardly into another language. (Hereinafter, translation is
   included without limitation in the term “modification”.)
   “Source code” for a work means the preferred form of the work for
   making modifications to it. For a library, complete source code means
   all the source code for all modules it contains, plus any associated
   interface definition files, plus the scripts used to control compilation
   and installation of the library.
   Activities other than copying, distribution and modification are not
   covered by this License; they are outside its scope. The act of
   running a program using the Library is not restricted, and output from
   such a program is covered only if its contents constitute a work based
   on the Library (independent of the use of the Library in a tool for
   writing it). Whether that is true depends on what the Library does
   and what the program that uses the Library does.- You may copy and distribute verbatim copies of the Library's
     complete source code as you receive it, in any medium, provided that
     you conspicuously and appropriately publish on each copy an
     appropriate copyright notice and disclaimer of warranty; keep intact
     all the notices that refer to this License and to the absence of any
     warranty; and distribute a copy of this License along with the
     Library.
     You may charge a fee for the physical act of transferring a copy,
     and you may at your option offer warranty protection in exchange for a
     fee.- You may modify your copy or copies of the Library or any portion
       of it, thus forming a work based on the Library, and copy and
       distribute such modifications or work under the terms of Section 1
       above, provided that you also meet all of these conditions:
       1. The modified work must itself be a software library.- You must cause the files modified to carry prominent notices
            stating that you changed the files and the date of any change.- You must cause the whole of the work to be licensed at no
              charge to all third parties under the terms of this License.- If a facility in the modified Library refers to a function or a
                table of data to be supplied by an application program that uses
                the facility, other than as an argument passed when the facility
                is invoked, then you must make a good faith effort to ensure that,
                in the event an application does not supply such function or
                table, the facility still operates, and performs whatever part of
                its purpose remains meaningful.
                (For example, a function in a library to compute square roots has
                a purpose that is entirely well-defined independent of the
                application. Therefore, Subsection 2d requires that any
                application-supplied function or table used by this function must
                be optional: if the application does not supply it, the square
                root function must still compute square roots.)
       These requirements apply to the modified work as a whole. If
       identifiable sections of that work are not derived from the Library,
       and can be reasonably considered independent and separate works in
       themselves, then this License, and its terms, do not apply to those
       sections when you distribute them as separate works. But when you
       distribute the same sections as part of a whole which is a work based
       on the Library, the distribution of the whole must be on the terms of
       this License, whose permissions for other licensees extend to the
       entire whole, and thus to each and every part regardless of who wrote
       it.
       Thus, it is not the intent of this section to claim rights or contest
       your rights to work written entirely by you; rather, the intent is to
       exercise the right to control the distribution of derivative or
       collective works based on the Library.
       In addition, mere aggregation of another work not based on the Library
       with the Library (or with a work based on the Library) on a volume of
       a storage or distribution medium does not bring the other work under
       the scope of this License.- You may opt to apply the terms of the ordinary GNU General Public
         License instead of this License to a given copy of the Library. To do
         this, you must alter all the notices that refer to this License, so
         that they refer to the ordinary GNU General Public License, version 2,
         instead of to this License. (If a newer version than version 2 of the
         ordinary GNU General Public License has appeared, then you can specify
         that version instead if you wish.) Do not make any other change in
         these notices.
         Once this change is made in a given copy, it is irreversible for
         that copy, so the ordinary GNU General Public License applies to all
         subsequent copies and derivative works made from that copy.
         This option is useful when you wish to copy part of the code of
         the Library into a program that is not a library.- You may copy and distribute the Library (or a portion or
           derivative of it, under Section 2) in object code or executable form
           under the terms of Sections 1 and 2 above provided that you accompany
           it with the complete corresponding machine-readable source code, which
           must be distributed under the terms of Sections 1 and 2 above on a
           medium customarily used for software interchange.
           If distribution of object code is made by offering access to copy
           from a designated place, then offering equivalent access to copy the
           source code from the same place satisfies the requirement to
           distribute the source code, even though third parties are not
           compelled to copy the source along with the object code.- A program that contains no derivative of any portion of the
             Library, but is designed to work with the Library by being compiled or
             linked with it, is called a “work that uses the Library”. Such a
             work, in isolation, is not a derivative work of the Library, and
             therefore falls outside the scope of this License.
             However, linking a “work that uses the Library” with the Library
             creates an executable that is a derivative of the Library (because it
             contains portions of the Library), rather than a “work that uses the
             library”. The executable is therefore covered by this License.
             Section 6 states terms for distribution of such executables.
             When a “work that uses the Library” uses material from a header file
             that is part of the Library, the object code for the work may be a
             derivative work of the Library even though the source code is not.
             Whether this is true is especially significant if the work can be
             linked without the Library, or if the work is itself a library. The
             threshold for this to be true is not precisely defined by law.
             If such an object file uses only numerical parameters, data
             structure layouts and accessors, and small macros and small inline
             functions (ten lines or less in length), then the use of the object
             file is unrestricted, regardless of whether it is legally a derivative
             work. (Executables containing this object code plus portions of the
             Library will still fall under Section 6.)
             Otherwise, if the work is a derivative of the Library, you may
             distribute the object code for the work under the terms of Section 6.
             Any executables containing that work also fall under Section 6,
             whether or not they are linked directly with the Library itself.- As an exception to the Sections above, you may also combine or
               link a “work that uses the Library” with the Library to produce a
               work containing portions of the Library, and distribute that work
               under terms of your choice, provided that the terms permit
               modification of the work for the customer's own use and reverse
               engineering for debugging such modifications.
               You must give prominent notice with each copy of the work that the
               Library is used in it and that the Library and its use are covered by
               this License. You must supply a copy of this License. If the work
               during execution displays copyright notices, you must include the
               copyright notice for the Library among them, as well as a reference
               directing the user to the copy of this License. Also, you must do one
               of these things:
               1. Accompany the work with the complete corresponding
                  machine-readable source code for the Library including whatever
                  changes were used in the work (which must be distributed under
                  Sections 1 and 2 above); and, if the work is an executable linked
                  with the Library, with the complete machine-readable “work that
                  uses the Library”, as object code and/or source code, so that the
                  user can modify the Library and then relink to produce a modified
                  executable containing the modified Library. (It is understood
                  that the user who changes the contents of definitions files in the
                  Library will not necessarily be able to recompile the application
                  to use the modified definitions.)- Use a suitable shared library mechanism for linking with the Library. A
                    suitable mechanism is one that (1) uses at run time a copy of the
                    library already present on the user's computer system, rather than
                    copying library functions into the executable, and (2) will operate
                    properly with a modified version of the library, if the user installs
                    one, as long as the modified version is interface-compatible with the
                    version that the work was made with.- Accompany the work with a written offer, valid for at
                      least three years, to give the same user the materials
                      specified in Subsection 6a, above, for a charge no more
                      than the cost of performing this distribution.- If distribution of the work is made by offering access to copy
                        from a designated place, offer equivalent access to copy the above
                        specified materials from the same place.- Verify that the user has already received a copy of these
                          materials or that you have already sent this user a copy.
               For an executable, the required form of the “work that uses the
               Library” must include any data and utility programs needed for
               reproducing the executable from it. However, as a special exception,
               the materials to be distributed need not include anything that is
               normally distributed (in either source or binary form) with the major
               components (compiler, kernel, and so on) of the operating system on
               which the executable runs, unless that component itself accompanies the
               executable.
               It may happen that this requirement contradicts the license
               restrictions of other proprietary libraries that do not normally
               accompany the operating system. Such a contradiction means you cannot
               use both them and the Library together in an executable that you
               distribute.- You may place library facilities that are a work based on the
                 Library side-by-side in a single library together with other library
                 facilities not covered by this License, and distribute such a combined
                 library, provided that the separate distribution of the work based on
                 the Library and of the other library facilities is otherwise
                 permitted, and provided that you do these two things:
                 1. Accompany the combined library with a copy of the same work
                    based on the Library, uncombined with any other library
                    facilities. This must be distributed under the terms of the
                    Sections above.- Give prominent notice with the combined library of the fact
                      that part of it is a work based on the Library, and explaining
                      where to find the accompanying uncombined form of the same work.
                 - You may not copy, modify, sublicense, link with, or distribute
                   the Library except as expressly provided under this License. Any
                   attempt otherwise to copy, modify, sublicense, link with, or
                   distribute the Library is void, and will automatically terminate your
                   rights under this License. However, parties who have received copies,
                   or rights, from you under this License will not have their licenses
                   terminated so long as such parties remain in full compliance.- You are not required to accept this License, since you have not
                     signed it. However, nothing else grants you permission to modify or
                     distribute the Library or its derivative works. These actions are
                     prohibited by law if you do not accept this License. Therefore, by
                     modifying or distributing the Library (or any work based on the
                     Library), you indicate your acceptance of this License to do so, and
                     all its terms and conditions for copying, distributing or modifying
                     the Library or works based on it.- Each time you redistribute the Library (or any work based on the
                       Library), the recipient automatically receives a license from the
                       original licensor to copy, distribute, link with or modify the Library
                       subject to these terms and conditions. You may not impose any further
                       restrictions on the recipients' exercise of the rights granted herein.
                       You are not responsible for enforcing compliance by third parties with
                       this License.- If, as a consequence of a court judgment or allegation of patent
                         infringement or for any other reason (not limited to patent issues),
                         conditions are imposed on you (whether by court order, agreement or
                         otherwise) that contradict the conditions of this License, they do not
                         excuse you from the conditions of this License. If you cannot
                         distribute so as to satisfy simultaneously your obligations under this
                         License and any other pertinent obligations, then as a consequence you
                         may not distribute the Library at all. For example, if a patent
                         license would not permit royalty-free redistribution of the Library by
                         all those who receive copies directly or indirectly through you, then
                         the only way you could satisfy both it and this License would be to
                         refrain entirely from distribution of the Library.
                         If any portion of this section is held invalid or unenforceable under any
                         particular circumstance, the balance of the section is intended to apply,
                         and the section as a whole is intended to apply in other circumstances.
                         It is not the purpose of this section to induce you to infringe any
                         patents or other property right claims or to contest validity of any
                         such claims; this section has the sole purpose of protecting the
                         integrity of the free software distribution system which is
                         implemented by public license practices. Many people have made
                         generous contributions to the wide range of software distributed
                         through that system in reliance on consistent application of that
                         system; it is up to the author/donor to decide if he or she is willing
                         to distribute software through any other system and a licensee cannot
                         impose that choice.
                         This section is intended to make thoroughly clear what is believed to
                         be a consequence of the rest of this License.- If the distribution and/or use of the Library is restricted in
                           certain countries either by patents or by copyrighted interfaces, the
                           original copyright holder who places the Library under this License may add
                           an explicit geographical distribution limitation excluding those countries,
                           so that distribution is permitted only in or among countries not thus
                           excluded. In such case, this License incorporates the limitation as if
                           written in the body of this License.- The Free Software Foundation may publish revised and/or new
                             versions of the Lesser General Public License from time to time.
                             Such new versions will be similar in spirit to the present version,
                             but may differ in detail to address new problems or concerns.
                             Each version is given a distinguishing version number. If the Library
                             specifies a version number of this License which applies to it and
                             “any later version”, you have the option of following the terms and
                             conditions either of that version or of any later version published by
                             the Free Software Foundation. If the Library does not specify a
                             license version number, you may choose any version ever published by
                             the Free Software Foundation.- If you wish to incorporate parts of the Library into other free
                               programs whose distribution conditions are incompatible with these,
                               write to the author to ask for permission. For software which is
                               copyrighted by the Free Software Foundation, write to the Free
                               Software Foundation; we sometimes make exceptions for this. Our
                               decision will be guided by the two goals of preserving the free status
                               of all derivatives of our free software and of promoting the sharing
                               and reuse of software generally.- BECAUSE THE LIBRARY IS LICENSED FREE OF CHARGE, THERE IS NO
                                 WARRANTY FOR THE LIBRARY, TO THE EXTENT PERMITTED BY APPLICABLE LAW.
                                 EXCEPT WHEN OTHERWISE STATED IN WRITING THE COPYRIGHT HOLDERS AND/OR
                                 OTHER PARTIES PROVIDE THE LIBRARY “AS IS” WITHOUT WARRANTY OF ANY
                                 KIND, EITHER EXPRESSED OR IMPLIED, INCLUDING, BUT NOT LIMITED TO, THE
                                 IMPLIED WARRANTIES OF MERCHANTABILITY AND FITNESS FOR A PARTICULAR
                                 PURPOSE. THE ENTIRE RISK AS TO THE QUALITY AND PERFORMANCE OF THE
                                 LIBRARY IS WITH YOU. SHOULD THE LIBRARY PROVE DEFECTIVE, YOU ASSUME
                                 THE COST OF ALL NECESSARY SERVICING, REPAIR OR CORRECTION.- IN NO EVENT UNLESS REQUIRED BY APPLICABLE LAW OR AGREED TO IN
                                   WRITING WILL ANY COPYRIGHT HOLDER, OR ANY OTHER PARTY WHO MAY MODIFY
                                   AND/OR REDISTRIBUTE THE LIBRARY AS PERMITTED ABOVE, BE LIABLE TO YOU
                                   FOR DAMAGES, INCLUDING ANY GENERAL, SPECIAL, INCIDENTAL OR
                                   CONSEQUENTIAL DAMAGES ARISING OUT OF THE USE OR INABILITY TO USE THE
                                   LIBRARY (INCLUDING BUT NOT LIMITED TO LOSS OF DATA OR DATA BEING
                                   RENDERED INACCURATE OR LOSSES SUSTAINED BY YOU OR THIRD PARTIES OR A
                                   FAILURE OF THE LIBRARY TO OPERATE WITH ANY OTHER SOFTWARE), EVEN IF
                                   SUCH HOLDER OR OTHER PARTY HAS BEEN ADVISED OF THE POSSIBILITY OF SUCH
                                   DAMAGES.

### How to Apply These Terms to Your New Libraries

If you develop a new library, and you want it to be of the greatest
possible use to the public, we recommend making it free software that
everyone can redistribute and change. You can do so by permitting
redistribution under these terms (or, alternatively, under the terms of the
ordinary General Public License).

To apply these terms, attach the following notices to the library. It is
safest to attach them to the start of each source file to most effectively
convey the exclusion of warranty; and each file should have at least the
“copyright” line and a pointer to where the full notice is found.

```
one line to give the library's name and an idea of what it does.
Copyright (C) year  name of author

This library is free software; you can redistribute it and/or modify it
under the terms of the GNU Lesser General Public License as published by
the Free Software Foundation; either version 2.1 of the License, or (at
your option) any later version.

This library is distributed in the hope that it will be useful, but
WITHOUT ANY WARRANTY; without even the implied warranty of
MERCHANTABILITY or FITNESS FOR A PARTICULAR PURPOSE.  See the GNU
Lesser General Public License for more details.

You should have received a copy of the GNU Lesser General Public
License along with this library; if not, write to the Free Software
Foundation, Inc., 51 Franklin Street, Fifth Floor, Boston, MA 02110-1301,
USA.
```

Also add information on how to contact you by electronic and paper mail.

You should also get your employer (if you work as a programmer) or your
school, if any, to sign a “copyright disclaimer” for the library, if
necessary. Here is a sample; alter the names:

```
Yoyodyne, Inc., hereby disclaims all copyright interest in the library
`Frob' (a library for tweaking knobs) written by James Random Hacker.

signature of Ty Coon, 1 April 1990
Ty Coon, President of Vice
```

That's all there is to it!

## C.3 GNU Free Documentation License

Version 1.2, November 2002

```
Copyright (C) 2000,2001,2002 Free Software Foundation, Inc.
51 Franklin Street, Fifth Floor, Boston, MA  02110-1301, USA

Everyone is permitted to copy and distribute verbatim copies
of this license document, but changing it is not allowed.
```

1. PREAMBLE
   The purpose of this License is to make a manual, textbook, or other
   functional and useful document *free* in the sense of freedom: to
   assure everyone the effective freedom to copy and redistribute it,
   with or without modifying it, either commercially or noncommercially.
   Secondarily, this License preserves for the author and publisher a way
   to get credit for their work, while not being considered responsible
   for modifications made by others.
   This License is a kind of “copyleft”, which means that derivative
   works of the document must themselves be free in the same sense. It
   complements the GNU General Public License, which is a copyleft
   license designed for free software.
   We have designed this License in order to use it for manuals for free
   software, because free software needs free documentation: a free
   program should come with manuals providing the same freedoms that the
   software does. But this License is not limited to software manuals;
   it can be used for any textual work, regardless of subject matter or
   whether it is published as a printed book. We recommend this License
   principally for works whose purpose is instruction or reference.- APPLICABILITY AND DEFINITIONS
     This License applies to any manual or other work, in any medium, that
     contains a notice placed by the copyright holder saying it can be
     distributed under the terms of this License. Such a notice grants a
     world-wide, royalty-free license, unlimited in duration, to use that
     work under the conditions stated herein. The “Document”, below,
     refers to any such manual or work. Any member of the public is a
     licensee, and is addressed as “you”. You accept the license if you
     copy, modify or distribute the work in a way requiring permission
     under copyright law.
     A “Modified Version” of the Document means any work containing the
     Document or a portion of it, either copied verbatim, or with
     modifications and/or translated into another language.
     A “Secondary Section” is a named appendix or a front-matter section
     of the Document that deals exclusively with the relationship of the
     publishers or authors of the Document to the Document's overall
     subject (or to related matters) and contains nothing that could fall
     directly within that overall subject. (Thus, if the Document is in
     part a textbook of mathematics, a Secondary Section may not explain
     any mathematics.) The relationship could be a matter of historical
     connection with the subject or with related matters, or of legal,
     commercial, philosophical, ethical or political position regarding
     them.
     The “Invariant Sections” are certain Secondary Sections whose titles
     are designated, as being those of Invariant Sections, in the notice
     that says that the Document is released under this License. If a
     section does not fit the above definition of Secondary then it is not
     allowed to be designated as Invariant. The Document may contain zero
     Invariant Sections. If the Document does not identify any Invariant
     Sections then there are none.
     The “Cover Texts” are certain short passages of text that are listed,
     as Front-Cover Texts or Back-Cover Texts, in the notice that says that
     the Document is released under this License. A Front-Cover Text may
     be at most 5 words, and a Back-Cover Text may be at most 25 words.
     A “Transparent” copy of the Document means a machine-readable copy,
     represented in a format whose specification is available to the
     general public, that is suitable for revising the document
     straightforwardly with generic text editors or (for images composed of
     pixels) generic paint programs or (for drawings) some widely available
     drawing editor, and that is suitable for input to text formatters or
     for automatic translation to a variety of formats suitable for input
     to text formatters. A copy made in an otherwise Transparent file
     format whose markup, or absence of markup, has been arranged to thwart
     or discourage subsequent modification by readers is not Transparent.
     An image format is not Transparent if used for any substantial amount
     of text. A copy that is not “Transparent” is called “Opaque”.
     Examples of suitable formats for Transparent copies include plain
     ASCII without markup, Texinfo input format, LaTeX input
     format, SGML or XML using a publicly available
     DTD, and standard-conforming simple HTML,
     PostScript or PDF designed for human modification. Examples
     of transparent image formats include PNG, XCF and
     JPG. Opaque formats include proprietary formats that can be
     read and edited only by proprietary word processors, SGML or
     XML for which the DTD and/or processing tools are
     not generally available, and the machine-generated HTML,
     PostScript or PDF produced by some word processors for
     output purposes only.
     The “Title Page” means, for a printed book, the title page itself,
     plus such following pages as are needed to hold, legibly, the material
     this License requires to appear in the title page. For works in
     formats which do not have any title page as such, “Title Page” means
     the text near the most prominent appearance of the work's title,
     preceding the beginning of the body of the text.
     A section “Entitled XYZ” means a named subunit of the Document whose
     title either is precisely XYZ or contains XYZ in parentheses following
     text that translates XYZ in another language. (Here XYZ stands for a
     specific section name mentioned below, such as “Acknowledgements”,
     “Dedications”, “Endorsements”, or “History”.) To “Preserve the Title”
     of such a section when you modify the Document means that it remains a
     section “Entitled XYZ” according to this definition.
     The Document may include Warranty Disclaimers next to the notice which
     states that this License applies to the Document. These Warranty
     Disclaimers are considered to be included by reference in this
     License, but only as regards disclaiming warranties: any other
     implication that these Warranty Disclaimers may have is void and has
     no effect on the meaning of this License.- VERBATIM COPYING
       You may copy and distribute the Document in any medium, either
       commercially or noncommercially, provided that this License, the
       copyright notices, and the license notice saying this License applies
       to the Document are reproduced in all copies, and that you add no other
       conditions whatsoever to those of this License. You may not use
       technical measures to obstruct or control the reading or further
       copying of the copies you make or distribute. However, you may accept
       compensation in exchange for copies. If you distribute a large enough
       number of copies you must also follow the conditions in section 3.
       You may also lend copies, under the same conditions stated above, and
       you may publicly display copies.- COPYING IN QUANTITY
         If you publish printed copies (or copies in media that commonly have
         printed covers) of the Document, numbering more than 100, and the
         Document's license notice requires Cover Texts, you must enclose the
         copies in covers that carry, clearly and legibly, all these Cover
         Texts: Front-Cover Texts on the front cover, and Back-Cover Texts on
         the back cover. Both covers must also clearly and legibly identify
         you as the publisher of these copies. The front cover must present
         the full title with all words of the title equally prominent and
         visible. You may add other material on the covers in addition.
         Copying with changes limited to the covers, as long as they preserve
         the title of the Document and satisfy these conditions, can be treated
         as verbatim copying in other respects.
         If the required texts for either cover are too voluminous to fit
         legibly, you should put the first ones listed (as many as fit
         reasonably) on the actual cover, and continue the rest onto adjacent
         pages.
         If you publish or distribute Opaque copies of the Document numbering
         more than 100, you must either include a machine-readable Transparent
         copy along with each Opaque copy, or state in or with each Opaque copy
         a computer-network location from which the general network-using
         public has access to download using public-standard network protocols
         a complete Transparent copy of the Document, free of added material.
         If you use the latter option, you must take reasonably prudent steps,
         when you begin distribution of Opaque copies in quantity, to ensure
         that this Transparent copy will remain thus accessible at the stated
         location until at least one year after the last time you distribute an
         Opaque copy (directly or through your agents or retailers) of that
         edition to the public.
         It is requested, but not required, that you contact the authors of the
         Document well before redistributing any large number of copies, to give
         them a chance to provide you with an updated version of the Document.- MODIFICATIONS
           You may copy and distribute a Modified Version of the Document under
           the conditions of sections 2 and 3 above, provided that you release
           the Modified Version under precisely this License, with the Modified
           Version filling the role of the Document, thus licensing distribution
           and modification of the Modified Version to whoever possesses a copy
           of it. In addition, you must do these things in the Modified Version:
           1. Use in the Title Page (and on the covers, if any) a title distinct
              from that of the Document, and from those of previous versions
              (which should, if there were any, be listed in the History section
              of the Document). You may use the same title as a previous version
              if the original publisher of that version gives permission.- List on the Title Page, as authors, one or more persons or entities
                responsible for authorship of the modifications in the Modified
                Version, together with at least five of the principal authors of the
                Document (all of its principal authors, if it has fewer than five),
                unless they release you from this requirement.- State on the Title page the name of the publisher of the
                  Modified Version, as the publisher.- Preserve all the copyright notices of the Document.- Add an appropriate copyright notice for your modifications
                      adjacent to the other copyright notices.- Include, immediately after the copyright notices, a license notice
                        giving the public permission to use the Modified Version under the
                        terms of this License, in the form shown in the Addendum below.- Preserve in that license notice the full lists of Invariant Sections
                          and required Cover Texts given in the Document's license notice.- Include an unaltered copy of this License.- Preserve the section Entitled “History”, Preserve its Title, and add
                              to it an item stating at least the title, year, new authors, and
                              publisher of the Modified Version as given on the Title Page. If
                              there is no section Entitled “History” in the Document, create one
                              stating the title, year, authors, and publisher of the Document as
                              given on its Title Page, then add an item describing the Modified
                              Version as stated in the previous sentence.- Preserve the network location, if any, given in the Document for
                                public access to a Transparent copy of the Document, and likewise
                                the network locations given in the Document for previous versions
                                it was based on. These may be placed in the “History” section.
                                You may omit a network location for a work that was published at
                                least four years before the Document itself, or if the original
                                publisher of the version it refers to gives permission.- For any section Entitled “Acknowledgements” or “Dedications”, Preserve
                                  the Title of the section, and preserve in the section all the
                                  substance and tone of each of the contributor acknowledgements and/or
                                  dedications given therein.- Preserve all the Invariant Sections of the Document,
                                    unaltered in their text and in their titles. Section numbers
                                    or the equivalent are not considered part of the section titles.- Delete any section Entitled “Endorsements”. Such a section
                                      may not be included in the Modified Version.- Do not retitle any existing section to be Entitled “Endorsements” or
                                        to conflict in title with any Invariant Section.- Preserve any Warranty Disclaimers.
           If the Modified Version includes new front-matter sections or
           appendices that qualify as Secondary Sections and contain no material
           copied from the Document, you may at your option designate some or all
           of these sections as invariant. To do this, add their titles to the
           list of Invariant Sections in the Modified Version's license notice.
           These titles must be distinct from any other section titles.
           You may add a section Entitled “Endorsements”, provided it contains
           nothing but endorsements of your Modified Version by various
           parties--for example, statements of peer review or that the text has
           been approved by an organization as the authoritative definition of a
           standard.
           You may add a passage of up to five words as a Front-Cover Text, and a
           passage of up to 25 words as a Back-Cover Text, to the end of the list
           of Cover Texts in the Modified Version. Only one passage of
           Front-Cover Text and one of Back-Cover Text may be added by (or
           through arrangements made by) any one entity. If the Document already
           includes a cover text for the same cover, previously added by you or
           by arrangement made by the same entity you are acting on behalf of,
           you may not add another; but you may replace the old one, on explicit
           permission from the previous publisher that added the old one.
           The author(s) and publisher(s) of the Document do not by this License
           give permission to use their names for publicity for or to assert or
           imply endorsement of any Modified Version.- COMBINING DOCUMENTS
             You may combine the Document with other documents released under this
             License, under the terms defined in section 4 above for modified
             versions, provided that you include in the combination all of the
             Invariant Sections of all of the original documents, unmodified, and
             list them all as Invariant Sections of your combined work in its
             license notice, and that you preserve all their Warranty Disclaimers.
             The combined work need only contain one copy of this License, and
             multiple identical Invariant Sections may be replaced with a single
             copy. If there are multiple Invariant Sections with the same name but
             different contents, make the title of each such section unique by
             adding at the end of it, in parentheses, the name of the original
             author or publisher of that section if known, or else a unique number.
             Make the same adjustment to the section titles in the list of
             Invariant Sections in the license notice of the combined work.
             In the combination, you must combine any sections Entitled “History”
             in the various original documents, forming one section Entitled
             “History”; likewise combine any sections Entitled “Acknowledgements”,
             and any sections Entitled “Dedications”. You must delete all
             sections Entitled “Endorsements.”- COLLECTIONS OF DOCUMENTS
               You may make a collection consisting of the Document and other documents
               released under this License, and replace the individual copies of this
               License in the various documents with a single copy that is included in
               the collection, provided that you follow the rules of this License for
               verbatim copying of each of the documents in all other respects.
               You may extract a single document from such a collection, and distribute
               it individually under this License, provided you insert a copy of this
               License into the extracted document, and follow this License in all
               other respects regarding verbatim copying of that document.- AGGREGATION WITH INDEPENDENT WORKS
                 A compilation of the Document or its derivatives with other separate
                 and independent documents or works, in or on a volume of a storage or
                 distribution medium, is called an “aggregate” if the copyright
                 resulting from the compilation is not used to limit the legal rights
                 of the compilation's users beyond what the individual works permit.
                 When the Document is included in an aggregate, this License does not
                 apply to the other works in the aggregate which are not themselves
                 derivative works of the Document.
                 If the Cover Text requirement of section 3 is applicable to these
                 copies of the Document, then if the Document is less than one half of
                 the entire aggregate, the Document's Cover Texts may be placed on
                 covers that bracket the Document within the aggregate, or the
                 electronic equivalent of covers if the Document is in electronic form.
                 Otherwise they must appear on printed covers that bracket the whole
                 aggregate.- TRANSLATION
                   Translation is considered a kind of modification, so you may
                   distribute translations of the Document under the terms of section 4.
                   Replacing Invariant Sections with translations requires special
                   permission from their copyright holders, but you may include
                   translations of some or all Invariant Sections in addition to the
                   original versions of these Invariant Sections. You may include a
                   translation of this License, and all the license notices in the
                   Document, and any Warranty Disclaimers, provided that you also include
                   the original English version of this License and the original versions
                   of those notices and disclaimers. In case of a disagreement between
                   the translation and the original version of this License or a notice
                   or disclaimer, the original version will prevail.
                   If a section in the Document is Entitled “Acknowledgements”,
                   “Dedications”, or “History”, the requirement (section 4) to Preserve
                   its Title (section 1) will typically require changing the actual
                   title.- TERMINATION
                     You may not copy, modify, sublicense, or distribute the Document except
                     as expressly provided for under this License. Any other attempt to
                     copy, modify, sublicense or distribute the Document is void, and will
                     automatically terminate your rights under this License. However,
                     parties who have received copies, or rights, from you under this
                     License will not have their licenses terminated so long as such
                     parties remain in full compliance.- FUTURE REVISIONS OF THIS LICENSE
                       The Free Software Foundation may publish new, revised versions
                       of the GNU Free Documentation License from time to time. Such new
                       versions will be similar in spirit to the present version, but may
                       differ in detail to address new problems or concerns. See
                       http://www.gnu.org/copyleft/.
                       Each version of the License is given a distinguishing version number.
                       If the Document specifies that a particular numbered version of this
                       License “or any later version” applies to it, you have the option of
                       following the terms and conditions either of that specified version or
                       of any later version that has been published (not as a draft) by the
                       Free Software Foundation. If the Document does not specify a version
                       number of this License, you may choose any version ever published (not
                       as a draft) by the Free Software Foundation.

### ADDENDUM: How to use this License for your documents

To use this License in a document you have written, include a copy of
the License in the document and put the following copyright and
license notices just after the title page:

```
  Copyright (C)  year  your name.
  Permission is granted to copy, distribute and/or modify this document
  under the terms of the GNU Free Documentation License, Version 1.2
  or any later version published by the Free Software Foundation;
  with no Invariant Sections, no Front-Cover Texts, and no Back-Cover
  Texts.  A copy of the license is included in the section entitled ``GNU
  Free Documentation License''.
```

If you have Invariant Sections, Front-Cover Texts and Back-Cover Texts,
replace the “with...Texts.” line with this:

```
    with the Invariant Sections being list their titles, with
    the Front-Cover Texts being list, and with the Back-Cover Texts
    being list.
```

If you have Invariant Sections without Cover Texts, or some other
combination of the three, merge those two alternatives to suit the
situation.

If your document contains nontrivial examples of program code, we
recommend releasing these examples in parallel under your choice of
free software license, such as the GNU General Public License,
to permit their use in free software.

---

Go to the first, previous, next, last section, table of contents.
