# Supplementary material for: rDock: A Fast, Versatile and Open Source Program for Docking Ligands to Proteins and Nucleic Acids
Source: PLoS Comput Biol. 2014 Apr 10;10(4):e1003571. doi: 10.1371/journal.pcbi.1003571 (PMC3983074; doi:10.1371/journal.pcbi.1003571)
Supplement: Software S1 — Compressed file with the source code of the rDock software for ligand docking to Proteins and Nucleic Acids. (GZ) [file pcbi.1003571.s010.gz › rDock_2013.1_src/gettext-0.18.tar.gz!/gettext-0.18/gettext-tools/doc/gettext_20.html]

GNU gettext utilities - Program Index

Go to the first, previous, next, last section, table of contents.

---

# Program Index

Jump to:
a
-
e
-
g
-
m
-
n
-
r
-
x

## a

- autopoint

## e

- envsubst

## g

- gettext, gettext- gettextize

## m

- msgattrib- msgcat- msgcmp- msgcomm- msgconv- msgen- msgexec- msgfilter- msgfmt- msggrep- msginit- msgmerge- msgunfmt- msguniq

## n

- ngettext, ngettext

## r

- recode-sr-latin

## x

- xgettext

---

Go to the first, previous, next, last section, table of contents.
