# Supplementary material for: rDock: A Fast, Versatile and Open Source Program for Docking Ligands to Proteins and Nucleic Acids
Source: PLoS Comput Biol. 2014 Apr 10;10(4):e1003571. doi: 10.1371/journal.pcbi.1003571 (PMC3983074; doi:10.1371/journal.pcbi.1003571)
Supplement: Software S1 — Compressed file with the source code of the rDock software for ligand docking to Proteins and Nucleic Acids. (GZ) [file pcbi.1003571.s010.gz › rDock_2013.1_src/gettext-0.18.tar.gz!/gettext-0.18/gettext-tools/doc/gettext_12.html]

GNU gettext utilities - 12 The Translator's View

Go to the first, previous, next, last section, table of contents.

---

# 12 The Translator's View

## 12.1 Introduction 0

**NOTE:**  This documentation section is outdated and needs to be
revised.

Free software is going international! The Translation Project is a way
to get maintainers, translators and users all together, so free software
will gradually become able to speak many native languages.

The GNU `gettext` tool set contains *everything* maintainers
need for internationalizing their packages for messages. It also
contains quite useful tools for helping translators at localizing
messages to their native language, once a package has already been
internationalized.

To achieve the Translation Project, we need many interested
people who like their own language and write it well, and who are also
able to synergize with other translators speaking the same language.
If you'd like to volunteer to *work* at translating messages,
please send mail to your translating team.

Each team has its own mailing list, courtesy of Linux
International. You may reach your translating team at the address
‘ll@li.org’, replacing ll by the two-letter ISO 639
code for your language. Language codes are *not* the same as
country codes given in ISO 3166. The following translating teams
exist:

> Chinese `zh`, Czech `cs`, Danish `da`, Dutch `nl`,
> Esperanto `eo`, Finnish `fi`, French `fr`, Irish
> `ga`, German `de`, Greek `el`, Italian `it`,
> Japanese `ja`, Indonesian `in`, Norwegian `no`, Polish
> `pl`, Portuguese `pt`, Russian `ru`, Spanish `es`,
> Swedish `sv` and Turkish `tr`.

For example, you may reach the Chinese translating team by writing to
‘zh@li.org’. When you become a member of the translating team
for your own language, you may subscribe to its list. For example,
Swedish people can send a message to ‘sv-request@li.org’,
having this message body:

```
subscribe
```

Keep in mind that team members should be interested in *working*
at translations, or at solving translational difficulties, rather than
merely lurking around. If your team does not exist yet and you want to
start one, please write to ‘coordinator@translationproject.org’;
you will then reach the coordinator for all translator teams.

A handful of GNU packages have already been adapted and provided
with message translations for several languages. Translation
teams have begun to organize, using these packages as a starting
point. But there are many more packages and many languages for
which we have no volunteer translators. If you would like to
volunteer to work at translating messages, please send mail to
‘coordinator@translationproject.org’ indicating what language(s)
you can work on.

## 12.2 Introduction 1

**NOTE:**  This documentation section is outdated and needs to be
revised.

This is now official, GNU is going international! Here is the
announcement submitted for the January 1995 GNU Bulletin:

> A handful of GNU packages have already been adapted and provided
> with message translations for several languages. Translation
> teams have begun to organize, using these packages as a starting
> point. But there are many more packages and many languages
> for which we have no volunteer translators. If you'd like to
> volunteer to work at translating messages, please send mail to
> `‘coordinator@translationproject.org’` indicating what language(s)
> you can work on.

This document should answer many questions for those who are curious about
the process or would like to contribute. Please at least skim over it,
hoping to cut down a little of the high volume of e-mail generated by this
collective effort towards internationalization of free software.

Most free programming which is widely shared is done in English, and
currently, English is used as the main communicating language between
national communities collaborating to free software. This very document
is written in English. This will not change in the foreseeable future.

However, there is a strong appetite from national communities for
having more software able to write using national language and habits,
and there is an on-going effort to modify free software in such a way
that it becomes able to do so. The experiments driven so far raised
an enthusiastic response from pretesters, so we believe that
internationalization of free software is dedicated to succeed.

For suggestion clarifications, additions or corrections to this
document, please e-mail to ‘coordinator@translationproject.org’.

## 12.3 Discussions

**NOTE:**  This documentation section is outdated and needs to be
revised.

Facing this internationalization effort, a few users expressed their
concerns. Some of these doubts are presented and discussed, here.

- Smaller groups
  Some languages are not spoken by a very large number of people, so people
  speaking them sometimes consider that there may not be all that much
  demand such versions of free software packages. Moreover, many people
  being *into computers*, in some countries, generally seem to prefer
  English versions of their software.
  On the other end, people might enjoy their own language a lot, and be
  very motivated at providing to themselves the pleasure of having their
  beloved free software speaking their mother tongue. They do themselves
  a personal favor, and do not pay that much attention to the number of
  people benefiting of their work.- Misinterpretation
    Other users are shy to push forward their own language, seeing in this
    some kind of misplaced propaganda. Someone thought there must be some
    users of the language over the networks pestering other people with it.
    But any spoken language is worth localization, because there are
    people behind the language for whom the language is important and
    dear to their hearts.- Odd translations
      The biggest problem is to find the right translations so that
      everybody can understand the messages. Translations are usually a
      little odd. Some people get used to English, to the extent they may
      find translations into their own language “rather pushy, obnoxious
      and sometimes even hilarious.” As a French speaking man, I have
      the experience of those instruction manuals for goods, so poorly
      translated in French in Korea or Taiwan...
      The fact is that we sometimes have to create a kind of national
      computer culture, and this is not easy without the collaboration of
      many people liking their mother tongue. This is why translations are
      better achieved by people knowing and loving their own language, and
      ready to work together at improving the results they obtain.- Dependencies over the GPL or LGPL
        Some people wonder if using GNU `gettext` necessarily brings their
        package under the protective wing of the GNU General Public License or
        the GNU Library General Public License, when they do not want to make
        their program free, or want other kinds of freedom. The simplest
        answer is “normally not”.
        The `gettext-runtime` part of GNU `gettext`, i.e. the
        contents of `libintl`, is covered by the GNU Library General Public
        License. The `gettext-tools` part of GNU `gettext`, i.e. the
        rest of the GNU `gettext` package, is covered by the GNU General
        Public License.
        The mere marking of localizable strings in a package, or conditional
        inclusion of a few lines for initialization, is not really including
        GPL'ed or LGPL'ed code. However, since the localization routines in
        `libintl` are under the LGPL, the LGPL needs to be considered.
        It gives the right to distribute the complete unmodified source of
        `libintl` even with non-free programs. It also gives the right
        to use `libintl` as a shared library, even for non-free programs.
        But it gives the right to use `libintl` as a static library or
        to incorporate `libintl` into another library only to free
        software.

## 12.4 Organization

**NOTE:**  This documentation section is outdated and needs to be
revised.

On a larger scale, the true solution would be to organize some kind of
fairly precise set up in which volunteers could participate. I gave
some thought to this idea lately, and realize there will be some
touchy points. I thought of writing to Richard Stallman to launch
such a project, but feel it might be good to shake out the ideas
between ourselves first. Most probably that Linux International has
some experience in the field already, or would like to orchestrate
the volunteer work, maybe. Food for thought, in any case!

I guess we have to setup something early, somehow, that will help
many possible contributors of the same language to interlock and avoid
work duplication, and further be put in contact for solving together
problems particular to their tongue (in most languages, there are many
difficulties peculiar to translating technical English). My Swedish
contributor acknowledged these difficulties, and I'm well aware of
them for French.

This is surely not a technical issue, but we should manage so the
effort of locale contributors be maximally useful, despite the national
team layer interface between contributors and maintainers.

The Translation Project needs some setup for coordinating language
coordinators. Localizing evolving programs will surely
become a permanent and continuous activity in the free software community,
once well started.
The setup should be minimally completed and tested before GNU
`gettext` becomes an official reality. The e-mail address
‘coordinator@translationproject.org’ has been set up for receiving
offers from volunteers and general e-mail on these topics. This address
reaches the Translation Project coordinator.

### 12.4.1 Central Coordination

I also think GNU will need sooner than it thinks, that someone set up
a way to organize and coordinate these groups. Some kind of group
of groups. My opinion is that it would be good that GNU delegates
this task to a small group of collaborating volunteers, shortly.
Perhaps in ‘gnu.announce’ a list of this national committee's
can be published.

My role as coordinator would simply be to refer to Ulrich any German
speaking volunteer interested to localization of free software packages, and
maybe helping national groups to initially organize, while maintaining
national registries for until national groups are ready to take over.
In fact, the coordinator should ease volunteers to get in contact with
one another for creating national teams, which should then select
one coordinator per language, or country (regionalized language).
If well done, the coordination should be useful without being an
overwhelming task, the time to put delegations in place.

### 12.4.2 National Teams

I suggest we look for volunteer coordinators/editors for individual
languages. These people will scan contributions of translation files
for various programs, for their own languages, and will ensure high
and uniform standards of diction.

From my current experience with other people in these days, those who
provide localizations are very enthusiastic about the process, and are
more interested in the localization process than in the program they
localize, and want to do many programs, not just one. This seems
to confirm that having a coordinator/editor for each language is a
good idea.

We need to choose someone who is good at writing clear and concise
prose in the language in question. That is hard--we can't check
it ourselves. So we need to ask a few people to judge each others'
writing and select the one who is best.

I announce my prerelease to a few dozen people, and you would not
believe all the discussions it generated already. I shudder to think
what will happen when this will be launched, for true, officially,
world wide. Who am I to arbitrate between two Czekolsovak users
contradicting each other, for example?

I assume that your German is not much better than my French so that
I would not be able to judge about these formulations. What I would
suggest is that for each language there is a group for people who
maintain the PO files and judge about changes. I suspect there will
be cultural differences between how such groups of people will behave.
Some will have relaxed ways, reach consensus easily, and have anyone
of the group relate to the maintainers, while others will fight to
death, organize heavy administrations up to national standards, and
use strict channels.

The German team is putting out a good example. Right now, they are
maybe half a dozen people revising translations of each other and
discussing the linguistic issues. I do not even have all the names.
Ulrich Drepper is taking care of coordinating the German team.
He subscribed to all my pretest lists, so I do not even have to warn
him specifically of incoming releases.

I'm sure, that is a good idea to get teams for each language working
on translations. That will make the translations better and more
consistent.

#### 12.4.2.1 Sub-Cultures

Taking French for example, there are a few sub-cultures around computers
which developed diverging vocabularies. Picking volunteers here and
there without addressing this problem in an organized way, soon in the
project, might produce a distasteful mix of internationalized programs,
and possibly trigger endless quarrels among those who really care.

Keeping some kind of unity in the way French localization of
internationalized programs is achieved is a difficult (and delicate) job.
Knowing the latin character of French people (:-), if we take this
the wrong way, we could end up nowhere, or spoil a lot of energies.
Maybe we should begin to address this problem seriously *before*
GNU `gettext` become officially published. And I suspect that this
means soon!

#### 12.4.2.2 Organizational Ideas

I expect the next big changes after the official release. Please note
that I use the German translation of the short GPL message. We need
to set a few good examples before the localization goes out for true
in the free software community. Here are a few points to discuss:

- Each group should have one FTP server (at least one master).- The files on the server should reflect the latest version (of
    course!) and it should also contain a RCS directory with the
    corresponding archives (I don't have this now).- There should also be a ChangeLog file (this is more useful than the
      RCS archive but can be generated automatically from the later by
      Emacs).- A *core group* should judge about questionable changes (for now
        this group consists solely by me but I ask some others occasionally;
        this also seems to work).

### 12.4.3 Mailing Lists

If we get any inquiries about GNU `gettext`, send them on to:

```
‘coordinator@translationproject.org’
```

The ‘\*-pretest’ lists are quite useful to me, maybe the idea could
be generalized to many GNU, and non-GNU packages. But each maintainer
his/her way!

François, we have a mechanism in place here at
‘gnu.ai.mit.edu’ to track teams, support mailing lists for
them and log members. We have a slight preference that you use it.
If this is OK with you, I can get you clued in.

Things are changing! A few years ago, when Daniel Fekete and I
asked for a mailing list for GNU localization, nested at the FSF, we
were politely invited to organize it anywhere else, and so did we.
For communicating with my pretesters, I later made a handful of
mailing lists located at iro.umontreal.ca and administrated by
`majordomo`. These lists have been *very* dependable
so far...

I suspect that the German team will organize itself a mailing list
located in Germany, and so forth for other countries. But before they
organize for true, it could surely be useful to offer mailing lists
located at the FSF to each national team. So yes, please explain me
how I should proceed to create and handle them.

We should create temporary mailing lists, one per country, to help
people organize. Temporary, because once regrouped and structured, it
would be fair the volunteers from country bring back *their* list
in there and manage it as they want. My feeling is that, in the long
run, each team should run its own list, from within their country.
There also should be some central list to which all teams could
subscribe as they see fit, as long as each team is represented in it.

## 12.5 Information Flow

**NOTE:**  This documentation section is outdated and needs to be
revised.

There will surely be some discussion about this messages after the
packages are finally released. If people now send you some proposals
for better messages, how do you proceed? Jim, please note that
right now, as I put forward nearly a dozen of localizable programs, I
receive both the translations and the coordination concerns about them.

If I put one of my things to pretest, Ulrich receives the announcement
and passes it on to the German team, who make last minute revisions.
Then he submits the translation files to me *as the maintainer*.
For free packages I do not maintain, I would not even hear about it.
This scheme could be made to work for the whole Translation Project,
I think. For security reasons, maybe Ulrich (national coordinators,
in fact) should update central registry kept at the Translation Project
(Jim, me, or Len's recruits) once in a while.

In December/January, I was aggressively ready to internationalize
all of GNU, giving myself the duty of one small GNU package per week
or so, taking many weeks or months for bigger packages. But it does
not work this way. I first did all the things I'm responsible for.
I've nothing against some missionary work on other maintainers, but
I'm also loosing a lot of energy over it--same debates over again.

And when the first localized packages are released we'll get a lot of
responses about ugly translations :-). Surely, and we need to have
beforehand a fairly good idea about how to handle the information
flow between the national teams and the package maintainers.

Please start saving somewhere a quick history of each PO file. I know
for sure that the file format will change, allowing for comments.
It would be nice that each file has a kind of log, and references for
those who want to submit comments or gripes, or otherwise contribute.
I sent a proposal for a fast and flexible format, but it is not
receiving acceptance yet by the GNU deciders. I'll tell you when I
have more information about this.

## 12.6 Translating plural forms

Suppose you are translating a PO file, and it contains an entry like this:

```
#, c-format
msgid "One file removed"
msgid_plural "%d files removed"
msgstr[0] ""
msgstr[1] ""
```

What does this mean? How do you fill it in?

Such an entry denotes a message with plural forms, that is, a message where
the text depends on a cardinal number. The general form of the message,
in English, is the `msgid_plural` line. The `msgid` line is the
English singular form, that is, the form for when the number is equal to 1.
More details about plural forms are explained in section 11.2.6 Additional functions for plural forms.

The first thing you need to look at is the `Plural-Forms` line in the
header entry of the PO file. It contains the number of plural forms and a
formula. If the PO file does not yet have such a line, you have to add it.
It only depends on the language into which you are translating. You can
get this info by using the `msginit` command (see section 6 Creating a New PO File) --
it contains a database of known plural formulas -- or by asking other
members of your translation team.

Suppose the line looks as follows:

```
"Plural-Forms: nplurals=3; plural=n%10==1 && n%100!=11 ? 0 : n%10>=2 && n"
"%10<=4 && (n%100<10 || n%100>=20) ? 1 : 2;\n"
```

It's logically one line; recall that the PO file formatting is allowed to
break long lines so that each physical line fits in 80 monospaced columns.

The value of `nplurals` here tells you that there are three plural
forms. The first thing you need to do is to ensure that the entry contains
an `msgstr` line for each of the forms:

```
#, c-format
msgid "One file removed"
msgid_plural "%d files removed"
msgstr[0] ""
msgstr[1] ""
msgstr[2] ""
```

Then translate the `msgid_plural` line and fill it in into each
`msgstr` line:

```
#, c-format
msgid "One file removed"
msgid_plural "%d files removed"
msgstr[0] "%d slika uklonjenih"
msgstr[1] "%d slika uklonjenih"
msgstr[2] "%d slika uklonjenih"
```

Now you can refine the translation so that it matches the plural form.
According to the formula above, `msgstr[0]` is used when the number
ends in 1 but does not end in 11; `msgstr[1]` is used when the number
ends in 2, 3, 4, but not in 12, 13, 14; and `msgstr[2]` is used in
all other cases. With this knowledge, you can refine the translations:

```
#, c-format
msgid "One file removed"
msgid_plural "%d files removed"
msgstr[0] "%d slika je uklonjena"
msgstr[1] "%d datoteke uklonjenih"
msgstr[2] "%d slika uklonjenih"
```

You noticed that in the English singular form (`msgid`) the number
placeholder could be omitted and replaced by the numeral word “one”.
Can you do this in your translation as well?

```
msgstr[0] "jednom datotekom je uklonjen"
```

Well, it depends on whether `msgstr[0]` applies only to the number 1,
or to other numbers as well. If, according to the plural formula,
`msgstr[0]` applies only to `n == 1`, then you can use the
specialized translation without the number placeholder. In our case,
however, `msgstr[0]` also applies to the numbers 21, 31, 41, etc.,
and therefore you cannot omit the placeholder.

## 12.7 Prioritizing messages: How to determine which messages to translate first

A translator sometimes has only a limited amount of time per week to
spend on a package, and some packages have quite large message catalogs
(over 1000 messages). Therefore she wishes to translate the messages
first that are the most visible to the user, or that occur most frequently.
This section describes how to determine these "most urgent" messages.
It also applies to determine the "next most urgent" messages after the
message catalog has already been partially translated.

In a first step, she uses the programs like a user would do. While she
does this, the GNU `gettext` library logs into a file the not yet
translated messages for which a translation was requested from the program.

In a second step, she uses the PO mode to translate precisely this set
of messages.

Here a more details. The GNU `libintl` library (but not the
corresponding functions in GNU `libc`) supports an environment variable
`GETTEXT_LOG_UNTRANSLATED`. The GNU `libintl` library will
log into this file the messages for which `gettext()` and related
functions couldn't find the translation. If the file doesn't exist, it
will be created as needed. On systems with GNU `libc` a shared library
`‘preloadable_libintl.so’` is provided that can be used with the ELF
`‘LD_PRELOAD’` mechanism.

So, in the first step, the translator uses these commands on systems with
GNU `libc`:

```
$ LD_PRELOAD=/usr/local/lib/preloadable_libintl.so
$ export LD_PRELOAD
$ GETTEXT_LOG_UNTRANSLATED=$HOME/gettextlogused
$ export GETTEXT_LOG_UNTRANSLATED
```

and these commands on other systems:

```
$ GETTEXT_LOG_UNTRANSLATED=$HOME/gettextlogused
$ export GETTEXT_LOG_UNTRANSLATED
```

Then she uses and peruses the programs. (It is a good and recommended
practice to use the programs for which you provide translations: it
gives you the needed context.) When done, she removes the environment
variables:

```
$ unset LD_PRELOAD
$ unset GETTEXT_LOG_UNTRANSLATED
```

The second step starts with removing duplicates:

```
$ msguniq $HOME/gettextlogused > missing.po
```

The result is a PO file, but needs some preprocessing before a PO file editor
can be used with it. First, it is a multi-domain PO file, containing
messages from many translation domains. Second, it lacks all translator
comments and source references. Here is how to get a list of the affected
translation domains:

```
$ sed -n -e 's,^domain "\(.*\)"$,\1,p' < missing.po | sort | uniq
```

Then the translator can handle the domains one by one. For simplicity,
let's use environment variables to denote the language, domain and source
package.

```
$ lang=nl             # your language
$ domain=coreutils    # the name of the domain to be handled
$ package=/usr/src/gnu/coreutils-4.5.4   # the package where it comes from
```

She takes the latest copy of ‘$lang.po’ from the Translation Project,
or from the package (in most cases, ‘$package/po/$lang.po’), or
creates a fresh one if she's the first translator (see section 6 Creating a New PO File).
She then uses the following commands to mark the not urgent messages as
"obsolete". (This doesn't mean that these messages - translated and
untranslated ones - will go away. It simply means that the PO file editor
will ignore them in the following editing session.)

```
$ msggrep --domain=$domain missing.po | grep -v '^domain' \
  > $domain-missing.po
$ msgattrib --set-obsolete --ignore-file $domain-missing.po $domain.$lang.po \
  > $domain.$lang-urgent.po
```

The she translates ‘$domain.$lang-urgent.po’ by use of a PO file editor
(see section 8 Editing PO Files).
(FIXME: I don't know whether `KBabel` and `gtranslator` also
preserve obsolete messages, as they should.)
Finally she restores the not urgent messages (with their earlier
translations, for those which were already translated) through this command:

```
$ msgmerge --no-fuzzy-matching $domain.$lang-urgent.po $package/po/$domain.pot \
  > $domain.$lang.po
```

Then she can submit ‘$domain.$lang.po’ and proceed to the next domain.

---

Go to the first, previous, next, last section, table of contents.
