# Supplementary material for: rDock: A Fast, Versatile and Open Source Program for Docking Ligands to Proteins and Nucleic Acids
Source: PLoS Comput Biol. 2014 Apr 10;10(4):e1003571. doi: 10.1371/journal.pcbi.1003571 (PMC3983074; doi:10.1371/journal.pcbi.1003571)
Supplement: Software S1 — Compressed file with the source code of the rDock software for ligand docking to Proteins and Nucleic Acids. (GZ) [file pcbi.1003571.s010.gz › rDock_2013.1_src/gettext-0.18.tar.gz!/gettext-0.18/gettext-tools/doc/gettext_9.html]

GNU gettext utilities - 9 Manipulating PO Files

Go to the first, previous, next, last section, table of contents.

---

# 9 Manipulating PO Files

Sometimes it is necessary to manipulate PO files in a way that is better
performed automatically than by hand. GNU `gettext` includes a
complete set of tools for this purpose.

When merging two packages into a single package, the resulting POT file
will be the concatenation of the two packages' POT files. Thus the
maintainer must concatenate the two existing package translations into
a single translation catalog, for each language. This is best performed
using `‘msgcat’`. It is then the translators' duty to deal with any
possible conflicts that arose during the merge.

When a translator takes over the translation job from another translator,
but she uses a different character encoding in her locale, she will
convert the catalog to her character encoding. This is best done through
the `‘msgconv’` program.

When a maintainer takes a source file with tagged messages from another
package, he should also take the existing translations for this source
file (and not let the translators do the same job twice). One way to do
this is through `‘msggrep’`, another is to create a POT file for
that source file and use `‘msgmerge’`.

When a translator wants to adjust some translation catalog for a special
dialect or orthography -- for example, German as written in Switzerland
versus German as written in Germany -- she needs to apply some text
processing to every message in the catalog. The tool for doing this is
`‘msgfilter’`.

Another use of `msgfilter` is to produce approximately the POT file for
which a given PO file was made. This can be done through a filter command
like `‘msgfilter sed -e d | sed -e '/^# /d'’`. Note that the original
POT file may have had different comments and different plural message counts,
that's why it's better to use the original POT file if available.

When a translator wants to check her translations, for example according
to orthography rules or using a non-interactive spell checker, she can do
so using the `‘msgexec’` program.

When third party tools create PO or POT files, sometimes duplicates cannot
be avoided. But the GNU `gettext` tools give an error when they
encounter duplicate msgids in the same file and in the same domain.
To merge duplicates, the `‘msguniq’` program can be used.

`‘msgcomm’` is a more general tool for keeping or throwing away
duplicates, occurring in different files.

`‘msgcmp’` can be used to check whether a translation catalog is
completely translated.

`‘msgattrib’` can be used to select and extract only the fuzzy
or untranslated messages of a translation catalog.

`‘msgen’` is useful as a first step for preparing English translation
catalogs. It copies each message's msgid to its msgstr.

Finally, for those applications where all these various programs are not
sufficient, a library `‘libgettextpo’` is provided that can be used to
write other specialized programs that process PO files.

## 9.1 Invoking the `msgcat` Program

```
msgcat [option] [inputfile]...
```

The `msgcat` program concatenates and merges the specified PO files.
It finds messages which are common to two or more of the specified PO files.
By using the `--more-than` option, greater commonality may be requested
before messages are printed. Conversely, the `--less-than` option may be
used to specify less commonality before messages are printed (i.e.
`‘--less-than=2’` will only print the unique messages). Translations,
comments and extract comments will be cumulated, except that if
`--use-first` is specified, they will be taken from the first PO file
to define them. File positions from all PO files will be cumulated.

### 9.1.1 Input file location

`‘inputfile ...’`: Input files. `‘-f file’`: `‘--files-from=file’`: Read the names of the input files from file instead of getting them from the command line. `‘-D directory’`: `‘--directory=directory’`: Add directory to the list of directories. Source files are searched relative to this list of directories. The resulting ‘.po’ file will be written relative to the current directory, though.

If inputfile is `‘-’`, standard input is read.

### 9.1.2 Output file location

`‘-o file’`: `‘--output-file=file’`: Write output to specified file.

The results are written to standard output if no output file is specified
or if it is `‘-’`.

### 9.1.3 Message selection

`‘-< number’`: `‘--less-than=number’`: Print messages with less than number definitions, defaults to infinite if not set. `‘-> number’`: `‘--more-than=number’`: Print messages with more than number definitions, defaults to 0 if not set. `‘-u’`: `‘--unique’`: Shorthand for `‘--less-than=2’`. Requests that only unique messages be printed.

### 9.1.4 Input file syntax

`‘-P’`: `‘--properties-input’`: Assume the input files are Java ResourceBundles in Java `.properties` syntax, not in PO file syntax. `‘--stringtable-input’`: Assume the input files are NeXTstep/GNUstep localized resource files in `.strings` syntax, not in PO file syntax.

### 9.1.5 Output details

`‘-t’`: `‘--to-code=name’`: Specify encoding for output. `‘--use-first’`: Use first available translation for each message. Don't merge several translations into one. `‘--lang=catalogname’`: Specify the `‘Language’` field to be used in the header entry. See section 6.2 Filling in the Header Entry for the meaning of this field. Note: The `‘Language-Team’` and `‘Plural-Forms’` fields are left unchanged. `‘--color’`: `‘--color=when’`: Specify whether or when to use colors and other text attributes. See section 9.11.1 The `--color` option for details. `‘--style=style_file’`: Specify the CSS style rule file to use for `--color`. See section 9.11.3 The `--style` option for details. `‘--force-po’`: Always write an output file even if it contains no message. `‘-i’`: `‘--indent’`: Write the .po file using indented style. `‘--no-location’`: Do not write `‘#: filename:line’` lines. `‘-n’`: `‘--add-location’`: Generate `‘#: filename:line’` lines (default). `‘--strict’`: Write out a strict Uniforum conforming PO file. Note that this Uniforum format should be avoided because it doesn't support the GNU extensions. `‘-p’`: `‘--properties-output’`: Write out a Java ResourceBundle in Java `.properties` syntax. Note that this file format doesn't support plural forms and silently drops obsolete messages. `‘--stringtable-output’`: Write out a NeXTstep/GNUstep localized resource file in `.strings` syntax. Note that this file format doesn't support plural forms. `‘-w number’`: `‘--width=number’`: Set the output page width. Long strings in the output files will be split across multiple lines in order to ensure that each line's width (= number of screen columns) is less or equal to the given number. `‘--no-wrap’`: Do not break long message lines. Message lines whose width exceeds the output page width will not be split into several lines. Only file reference lines which are wider than the output page width will be split. `‘-s’`: `‘--sort-output’`: Generate sorted output. Note that using this option makes it much harder for the translator to understand each message's context. `‘-F’`: `‘--sort-by-file’`: Sort output by file location.

### 9.1.6 Informative output

`‘-h’`: `‘--help’`: Display this help and exit. `‘-V’`: `‘--version’`: Output version information and exit.

## 9.2 Invoking the `msgconv` Program

```
msgconv [option] [inputfile]
```

The `msgconv` program converts a translation catalog to a different
character encoding.

### 9.2.1 Input file location

`‘inputfile’`: Input PO file. `‘-D directory’`: `‘--directory=directory’`: Add directory to the list of directories. Source files are searched relative to this list of directories. The resulting ‘.po’ file will be written relative to the current directory, though.

If no inputfile is given or if it is `‘-’`, standard input is read.

### 9.2.2 Output file location

`‘-o file’`: `‘--output-file=file’`: Write output to specified file.

The results are written to standard output if no output file is specified
or if it is `‘-’`.

### 9.2.3 Conversion target

`‘-t’`: `‘--to-code=name’`: Specify encoding for output.

The default encoding is the current locale's encoding.

### 9.2.4 Input file syntax

`‘-P’`: `‘--properties-input’`: Assume the input file is a Java ResourceBundle in Java `.properties` syntax, not in PO file syntax. `‘--stringtable-input’`: Assume the input file is a NeXTstep/GNUstep localized resource file in `.strings` syntax, not in PO file syntax.

### 9.2.5 Output details

`‘--color’`: `‘--color=when’`: Specify whether or when to use colors and other text attributes. See section 9.11.1 The `--color` option for details. `‘--style=style_file’`: Specify the CSS style rule file to use for `--color`. See section 9.11.3 The `--style` option for details. `‘--force-po’`: Always write an output file even if it contains no message. `‘-i’`: `‘--indent’`: Write the .po file using indented style. `‘--no-location’`: Do not write `‘#: filename:line’` lines. `‘--add-location’`: Generate `‘#: filename:line’` lines (default). `‘--strict’`: Write out a strict Uniforum conforming PO file. Note that this Uniforum format should be avoided because it doesn't support the GNU extensions. `‘-p’`: `‘--properties-output’`: Write out a Java ResourceBundle in Java `.properties` syntax. Note that this file format doesn't support plural forms and silently drops obsolete messages. `‘--stringtable-output’`: Write out a NeXTstep/GNUstep localized resource file in `.strings` syntax. Note that this file format doesn't support plural forms. `‘-w number’`: `‘--width=number’`: Set the output page width. Long strings in the output files will be split across multiple lines in order to ensure that each line's width (= number of screen columns) is less or equal to the given number. `‘--no-wrap’`: Do not break long message lines. Message lines whose width exceeds the output page width will not be split into several lines. Only file reference lines which are wider than the output page width will be split. `‘-s’`: `‘--sort-output’`: Generate sorted output. Note that using this option makes it much harder for the translator to understand each message's context. `‘-F’`: `‘--sort-by-file’`: Sort output by file location.

### 9.2.6 Informative output

`‘-h’`: `‘--help’`: Display this help and exit. `‘-V’`: `‘--version’`: Output version information and exit.

## 9.3 Invoking the `msggrep` Program

```
msggrep [option] [inputfile]
```

The `msggrep` program extracts all messages of a translation catalog
that match a given pattern or belong to some given source files.

### 9.3.1 Input file location

`‘inputfile’`: Input PO file. `‘-D directory’`: `‘--directory=directory’`: Add directory to the list of directories. Source files are searched relative to this list of directories. The resulting ‘.po’ file will be written relative to the current directory, though.

If no inputfile is given or if it is `‘-’`, standard input is read.

### 9.3.2 Output file location

`‘-o file’`: `‘--output-file=file’`: Write output to specified file.

The results are written to standard output if no output file is specified
or if it is `‘-’`.

### 9.3.3 Message selection

```
  [-N sourcefile]... [-M domainname]...
  [-J msgctxt-pattern] [-K msgid-pattern] [-T msgstr-pattern]
  [-C comment-pattern]
```

A message is selected if

- it comes from one of the specified source files,- or if it comes from one of the specified domains,- or if `‘-J’` is given and its context (msgctxt) matches
      msgctxt-pattern,- or if `‘-K’` is given and its key (msgid or msgid\_plural) matches
        msgid-pattern,- or if `‘-T’` is given and its translation (msgstr) matches
          msgstr-pattern,- or if `‘-C’` is given and the translator's comment matches
            comment-pattern.

When more than one selection criterion is specified, the set of selected
messages is the union of the selected messages of each criterion.

msgctxt-pattern or msgid-pattern or msgstr-pattern syntax:

```
  [-E | -F] [-e pattern | -f file]...
```

patterns are basic regular expressions by default, or extended regular
expressions if -E is given, or fixed strings if -F is given.

`‘-N sourcefile’`: `‘--location=sourcefile’`: Select messages extracted from sourcefile. sourcefile can be either a literal file name or a wildcard pattern. `‘-M domainname’`: `‘--domain=domainname’`: Select messages belonging to domain domainname. `‘-J’`: `‘--msgctxt’`: Start of patterns for the msgctxt. `‘-K’`: `‘--msgid’`: Start of patterns for the msgid. `‘-T’`: `‘--msgstr’`: Start of patterns for the msgstr. `‘-C’`: `‘--comment’`: Start of patterns for the translator's comment. `‘-X’`: `‘--extracted-comment’`: Start of patterns for the extracted comments. `‘-E’`: `‘--extended-regexp’`: Specify that pattern is an extended regular expression. `‘-F’`: `‘--fixed-strings’`: Specify that pattern is a set of newline-separated strings. `‘-e pattern’`: `‘--regexp=pattern’`: Use pattern as a regular expression. `‘-f file’`: `‘--file=file’`: Obtain pattern from file. `‘-i’`: `‘--ignore-case’`: Ignore case distinctions. `‘-v’`: `‘--invert-match’`: Output only the messages that do not match any selection criterion, instead of the messages that match a selection criterion.

### 9.3.4 Input file syntax

`‘-P’`: `‘--properties-input’`: Assume the input file is a Java ResourceBundle in Java `.properties` syntax, not in PO file syntax. `‘--stringtable-input’`: Assume the input file is a NeXTstep/GNUstep localized resource file in `.strings` syntax, not in PO file syntax.

### 9.3.5 Output details

`‘--color’`: `‘--color=when’`: Specify whether or when to use colors and other text attributes. See section 9.11.1 The `--color` option for details. `‘--style=style_file’`: Specify the CSS style rule file to use for `--color`. See section 9.11.3 The `--style` option for details. `‘--force-po’`: Always write an output file even if it contains no message. `‘--indent’`: Write the .po file using indented style. `‘--no-location’`: Do not write `‘#: filename:line’` lines. `‘--add-location’`: Generate `‘#: filename:line’` lines (default). `‘--strict’`: Write out a strict Uniforum conforming PO file. Note that this Uniforum format should be avoided because it doesn't support the GNU extensions. `‘-p’`: `‘--properties-output’`: Write out a Java ResourceBundle in Java `.properties` syntax. Note that this file format doesn't support plural forms and silently drops obsolete messages. `‘--stringtable-output’`: Write out a NeXTstep/GNUstep localized resource file in `.strings` syntax. Note that this file format doesn't support plural forms. `‘-w number’`: `‘--width=number’`: Set the output page width. Long strings in the output files will be split across multiple lines in order to ensure that each line's width (= number of screen columns) is less or equal to the given number. `‘--no-wrap’`: Do not break long message lines. Message lines whose width exceeds the output page width will not be split into several lines. Only file reference lines which are wider than the output page width will be split. `‘--sort-output’`: Generate sorted output. Note that using this option makes it much harder for the translator to understand each message's context. `‘--sort-by-file’`: Sort output by file location.

### 9.3.6 Informative output

`‘-h’`: `‘--help’`: Display this help and exit. `‘-V’`: `‘--version’`: Output version information and exit.

### 9.3.7 Examples

To extract the messages that come from the source files
`gnulib-lib/error.c` and `gnulib-lib/getopt.c`:

```
msggrep -N gnulib-lib/error.c -N gnulib-lib/getopt.c input.po
```

To extract the messages that contain the string “Please specify” in the
original string:

```
msggrep --msgid -F -e 'Please specify' input.po
```

To extract the messages that have a context specifier of either “Menu>File”
or “Menu>Edit” or a submenu of them:

```
msggrep --msgctxt -E -e '^Menu>(File|Edit)' input.po
```

To extract the messages whose translation contains one of the strings in the
file `wordlist.txt`:

```
msggrep --msgstr -F -f wordlist.txt input.po
```

## 9.4 Invoking the `msgfilter` Program

```
msgfilter [option] filter [filter-option]
```

The `msgfilter` program applies a filter to all translations of a
translation catalog.

During each filter invocation, the environment variable
`MSGFILTER_MSGID` is bound to the message's msgid, and the environment
variable `MSGFILTER_LOCATION` is bound to the location in the PO file
of the message. If the message has a context, the environment variable
`MSGFILTER_MSGCTXT` is bound to the message's msgctxt, otherwise it is
unbound.

### 9.4.1 Input file location

`‘-i inputfile’`: `‘--input=inputfile’`: Input PO file. `‘-D directory’`: `‘--directory=directory’`: Add directory to the list of directories. Source files are searched relative to this list of directories. The resulting ‘.po’ file will be written relative to the current directory, though.

If no inputfile is given or if it is `‘-’`, standard input is read.

### 9.4.2 Output file location

`‘-o file’`: `‘--output-file=file’`: Write output to specified file.

The results are written to standard output if no output file is specified
or if it is `‘-’`.

### 9.4.3 The filter

The filter can be any program that reads a translation from standard
input and writes a modified translation to standard output. A frequently
used filter is `‘sed’`. A few particular built-in filters are also
recognized.

Note: If the filter is not a built-in filter, you have to care about encodings:
It is your responsibility to ensure that the filter can cope
with input encoded in the translation catalog's encoding. If the
filter wants input in a particular encoding, you can in a first step
convert the translation catalog to that encoding using the `‘msgconv’`
program, before invoking `‘msgfilter’`. If the filter wants input
in the locale's encoding, but you want to avoid the locale's encoding, then
you can first convert the translation catalog to UTF-8 using the
`‘msgconv’` program and then make `‘msgfilter’` work in an UTF-8
locale, by using the `LC_ALL` environment variable.

Note: Most translations in a translation catalog don't end with a newline
character. For this reason, it is important that the filter
recognizes its last input line even if it ends without a newline, and that
it doesn't add an undesired trailing newline at the end. The `‘sed’`
program on some platforms is known to ignore the last line of input if it
is not terminated with a newline. You can use GNU `sed` instead; it
does not have this limitation.

### 9.4.4 Useful filter-options when the filter is `‘sed’`

`‘-e script’`: `‘--expression=script’`: Add script to the commands to be executed. `‘-f scriptfile’`: `‘--file=scriptfile’`: Add the contents of scriptfile to the commands to be executed. `‘-n’`: `‘--quiet’`: `‘--silent’`: Suppress automatic printing of pattern space.

### 9.4.5 Built-in filters

The filter `‘recode-sr-latin’` is recognized as a built-in filter.
The command `‘recode-sr-latin’` converts Serbian text, written in the
Cyrillic script, to the Latin script.
The command `‘msgfilter recode-sr-latin’` applies this conversion to the
translations of a PO file. Thus, it can be used to convert an ‘sr.po’
file to an ‘sr@latin.po’ file.

The use of built-in filters is not sensitive to the current locale's encoding.
Moreover, when used with a built-in filter, `‘msgfilter’` can automatically
convert the message catalog to the UTF-8 encoding when needed.

### 9.4.6 Input file syntax

`‘-P’`: `‘--properties-input’`: Assume the input file is a Java ResourceBundle in Java `.properties` syntax, not in PO file syntax. `‘--stringtable-input’`: Assume the input file is a NeXTstep/GNUstep localized resource file in `.strings` syntax, not in PO file syntax.

### 9.4.7 Output details

`‘--color’`: `‘--color=when’`: Specify whether or when to use colors and other text attributes. See section 9.11.1 The `--color` option for details. `‘--style=style_file’`: Specify the CSS style rule file to use for `--color`. See section 9.11.3 The `--style` option for details. `‘--force-po’`: Always write an output file even if it contains no message. `‘--indent’`: Write the .po file using indented style. `‘--keep-header’`: Keep the header entry, i.e. the message with `‘msgid ""’`, unmodified, instead of filtering it. By default, the header entry is subject to filtering like any other message. `‘--no-location’`: Do not write `‘#: filename:line’` lines. `‘--add-location’`: Generate `‘#: filename:line’` lines (default). `‘--strict’`: Write out a strict Uniforum conforming PO file. Note that this Uniforum format should be avoided because it doesn't support the GNU extensions. `‘-p’`: `‘--properties-output’`: Write out a Java ResourceBundle in Java `.properties` syntax. Note that this file format doesn't support plural forms and silently drops obsolete messages. `‘--stringtable-output’`: Write out a NeXTstep/GNUstep localized resource file in `.strings` syntax. Note that this file format doesn't support plural forms. `‘-w number’`: `‘--width=number’`: Set the output page width. Long strings in the output files will be split across multiple lines in order to ensure that each line's width (= number of screen columns) is less or equal to the given number. `‘--no-wrap’`: Do not break long message lines. Message lines whose width exceeds the output page width will not be split into several lines. Only file reference lines which are wider than the output page width will be split. `‘-s’`: `‘--sort-output’`: Generate sorted output. Note that using this option makes it much harder for the translator to understand each message's context. `‘-F’`: `‘--sort-by-file’`: Sort output by file location.

### 9.4.8 Informative output

`‘-h’`: `‘--help’`: Display this help and exit. `‘-V’`: `‘--version’`: Output version information and exit.

### 9.4.9 Examples

To convert German translations to Swiss orthography (in an UTF-8 locale):

```
msgconv -t UTF-8 de.po | msgfilter sed -e 's/ß/ss/g'
```

To convert Serbian translations in Cyrillic script to Latin script:

```
msgfilter recode-sr-latin < sr.po
```

## 9.5 Invoking the `msguniq` Program

```
msguniq [option] [inputfile]
```

The `msguniq` program unifies duplicate translations in a translation
catalog. It finds duplicate translations of the same message ID. Such
duplicates are invalid input for other programs like `msgfmt`,
`msgmerge` or `msgcat`. By default, duplicates are merged
together. When using the `‘--repeated’` option, only duplicates are
output, and all other messages are discarded. Comments and extracted
comments will be cumulated, except that if `‘--use-first’` is
specified, they will be taken from the first translation. File positions
will be cumulated. When using the `‘--unique’` option, duplicates are
discarded.

### 9.5.1 Input file location

`‘inputfile’`: Input PO file. `‘-D directory’`: `‘--directory=directory’`: Add directory to the list of directories. Source files are searched relative to this list of directories. The resulting ‘.po’ file will be written relative to the current directory, though.

If no inputfile is given or if it is `‘-’`, standard input is read.

### 9.5.2 Output file location

`‘-o file’`: `‘--output-file=file’`: Write output to specified file.

The results are written to standard output if no output file is specified
or if it is `‘-’`.

### 9.5.3 Message selection

`‘-d’`: `‘--repeated’`: Print only duplicates. `‘-u’`: `‘--unique’`: Print only unique messages, discard duplicates.

### 9.5.4 Input file syntax

`‘-P’`: `‘--properties-input’`: Assume the input file is a Java ResourceBundle in Java `.properties` syntax, not in PO file syntax. `‘--stringtable-input’`: Assume the input file is a NeXTstep/GNUstep localized resource file in `.strings` syntax, not in PO file syntax.

### 9.5.5 Output details

`‘-t’`: `‘--to-code=name’`: Specify encoding for output. `‘--use-first’`: Use first available translation for each message. Don't merge several translations into one. `‘--color’`: `‘--color=when’`: Specify whether or when to use colors and other text attributes. See section 9.11.1 The `--color` option for details. `‘--style=style_file’`: Specify the CSS style rule file to use for `--color`. See section 9.11.3 The `--style` option for details. `‘--force-po’`: Always write an output file even if it contains no message. `‘-i’`: `‘--indent’`: Write the .po file using indented style. `‘--no-location’`: Do not write `‘#: filename:line’` lines. `‘-n’`: `‘--add-location’`: Generate `‘#: filename:line’` lines (default). `‘--strict’`: Write out a strict Uniforum conforming PO file. Note that this Uniforum format should be avoided because it doesn't support the GNU extensions. `‘-p’`: `‘--properties-output’`: Write out a Java ResourceBundle in Java `.properties` syntax. Note that this file format doesn't support plural forms and silently drops obsolete messages. `‘--stringtable-output’`: Write out a NeXTstep/GNUstep localized resource file in `.strings` syntax. Note that this file format doesn't support plural forms. `‘-w number’`: `‘--width=number’`: Set the output page width. Long strings in the output files will be split across multiple lines in order to ensure that each line's width (= number of screen columns) is less or equal to the given number. `‘--no-wrap’`: Do not break long message lines. Message lines whose width exceeds the output page width will not be split into several lines. Only file reference lines which are wider than the output page width will be split. `‘-s’`: `‘--sort-output’`: Generate sorted output. Note that using this option makes it much harder for the translator to understand each message's context. `‘-F’`: `‘--sort-by-file’`: Sort output by file location.

### 9.5.6 Informative output

`‘-h’`: `‘--help’`: Display this help and exit. `‘-V’`: `‘--version’`: Output version information and exit.

## 9.6 Invoking the `msgcomm` Program

```
msgcomm [option] [inputfile]...
```

The `msgcomm` program finds messages which are common to two or more
of the specified PO files.
By using the `--more-than` option, greater commonality may be requested
before messages are printed. Conversely, the `--less-than` option may be
used to specify less commonality before messages are printed (i.e.
`‘--less-than=2’` will only print the unique messages). Translations,
comments and extract comments will be preserved, but only from the first
PO file to define them. File positions from all PO files will be
cumulated.

### 9.6.1 Input file location

`‘inputfile ...’`: Input files. `‘-f file’`: `‘--files-from=file’`: Read the names of the input files from file instead of getting them from the command line. `‘-D directory’`: `‘--directory=directory’`: Add directory to the list of directories. Source files are searched relative to this list of directories. The resulting ‘.po’ file will be written relative to the current directory, though.

If inputfile is `‘-’`, standard input is read.

### 9.6.2 Output file location

`‘-o file’`: `‘--output-file=file’`: Write output to specified file.

The results are written to standard output if no output file is specified
or if it is `‘-’`.

### 9.6.3 Message selection

`‘-< number’`: `‘--less-than=number’`: Print messages with less than number definitions, defaults to infinite if not set. `‘-> number’`: `‘--more-than=number’`: Print messages with more than number definitions, defaults to 1 if not set. `‘-u’`: `‘--unique’`: Shorthand for `‘--less-than=2’`. Requests that only unique messages be printed.

### 9.6.4 Input file syntax

`‘-P’`: `‘--properties-input’`: Assume the input files are Java ResourceBundles in Java `.properties` syntax, not in PO file syntax. `‘--stringtable-input’`: Assume the input files are NeXTstep/GNUstep localized resource files in `.strings` syntax, not in PO file syntax.

### 9.6.5 Output details

`‘--color’`: `‘--color=when’`: Specify whether or when to use colors and other text attributes. See section 9.11.1 The `--color` option for details. `‘--style=style_file’`: Specify the CSS style rule file to use for `--color`. See section 9.11.3 The `--style` option for details. `‘--force-po’`: Always write an output file even if it contains no message. `‘-i’`: `‘--indent’`: Write the .po file using indented style. `‘--no-location’`: Do not write `‘#: filename:line’` lines. `‘-n’`: `‘--add-location’`: Generate `‘#: filename:line’` lines (default). `‘--strict’`: Write out a strict Uniforum conforming PO file. Note that this Uniforum format should be avoided because it doesn't support the GNU extensions. `‘-p’`: `‘--properties-output’`: Write out a Java ResourceBundle in Java `.properties` syntax. Note that this file format doesn't support plural forms and silently drops obsolete messages. `‘--stringtable-output’`: Write out a NeXTstep/GNUstep localized resource file in `.strings` syntax. Note that this file format doesn't support plural forms. `‘-w number’`: `‘--width=number’`: Set the output page width. Long strings in the output files will be split across multiple lines in order to ensure that each line's width (= number of screen columns) is less or equal to the given number. `‘--no-wrap’`: Do not break long message lines. Message lines whose width exceeds the output page width will not be split into several lines. Only file reference lines which are wider than the output page width will be split. `‘-s’`: `‘--sort-output’`: Generate sorted output. Note that using this option makes it much harder for the translator to understand each message's context. `‘-F’`: `‘--sort-by-file’`: Sort output by file location. `‘--omit-header’`: Don't write header with `‘msgid ""’` entry.

### 9.6.6 Informative output

`‘-h’`: `‘--help’`: Display this help and exit. `‘-V’`: `‘--version’`: Output version information and exit.

## 9.7 Invoking the `msgcmp` Program

```
msgcmp [option] def.po ref.pot
```

The `msgcmp` program compares two Uniforum style .po files to check that
both contain the same set of msgid strings. The def.po file is an
existing PO file with the translations. The ref.pot file is the last
created PO file, or a PO Template file (generally created by `xgettext`).
This is useful for checking that you have translated each and every message
in your program. Where an exact match cannot be found, fuzzy matching is
used to produce better diagnostics.

### 9.7.1 Input file location

`‘def.po’`: Translations. `‘ref.pot’`: References to the sources. `‘-D directory’`: `‘--directory=directory’`: Add directory to the list of directories. Source files are searched relative to this list of directories.

### 9.7.2 Operation modifiers

`‘-m’`: `‘--multi-domain’`: Apply ref.pot to each of the domains in def.po. `‘-N’`: `‘--no-fuzzy-matching’`: Do not use fuzzy matching when an exact match is not found. This may speed up the operation considerably. `‘--use-fuzzy’`: Consider fuzzy messages in the def.po file like translated messages. Note that using this option is usually wrong, because fuzzy messages are exactly those which have not been validated by a human translator. `‘--use-untranslated’`: Consider untranslated messages in the def.po file like translated messages. Note that using this option is usually wrong.

### 9.7.3 Input file syntax

`‘-P’`: `‘--properties-input’`: Assume the input files are Java ResourceBundles in Java `.properties` syntax, not in PO file syntax. `‘--stringtable-input’`: Assume the input files are NeXTstep/GNUstep localized resource files in `.strings` syntax, not in PO file syntax.

### 9.7.4 Informative output

`‘-h’`: `‘--help’`: Display this help and exit. `‘-V’`: `‘--version’`: Output version information and exit.

## 9.8 Invoking the `msgattrib` Program

```
msgattrib [option] [inputfile]
```

The `msgattrib` program filters the messages of a translation catalog
according to their attributes, and manipulates the attributes.

### 9.8.1 Input file location

`‘inputfile’`: Input PO file. `‘-D directory’`: `‘--directory=directory’`: Add directory to the list of directories. Source files are searched relative to this list of directories. The resulting ‘.po’ file will be written relative to the current directory, though.

If no inputfile is given or if it is `‘-’`, standard input is read.

### 9.8.2 Output file location

`‘-o file’`: `‘--output-file=file’`: Write output to specified file.

The results are written to standard output if no output file is specified
or if it is `‘-’`.

### 9.8.3 Message selection

`‘--translated’`: Keep translated messages, remove untranslated messages. `‘--untranslated’`: Keep untranslated messages, remove translated messages. `‘--no-fuzzy’`: Remove ‘fuzzy’ marked messages. `‘--only-fuzzy’`: Keep ‘fuzzy’ marked messages, remove all other messages. `‘--no-obsolete’`: Remove obsolete #~ messages. `‘--only-obsolete’`: Keep obsolete #~ messages, remove all other messages.

### 9.8.4 Attribute manipulation

Attributes are modified after the message selection/removal has been
performed. If the `‘--only-file’` or `‘--ignore-file’` option is
specified, the attribute modification is applied only to those messages
that are listed in the only-file and not listed in the
ignore-file.

`‘--set-fuzzy’`: Set all messages ‘fuzzy’. `‘--clear-fuzzy’`: Set all messages non-‘fuzzy’. `‘--set-obsolete’`: Set all messages obsolete. `‘--clear-obsolete’`: Set all messages non-obsolete. `‘--clear-previous’`: Remove the “previous msgid” (`‘#|’`) comments from all messages. `‘--only-file=file’`: Limit the attribute changes to entries that are listed in file. file should be a PO or POT file. `‘--ignore-file=file’`: Limit the attribute changes to entries that are not listed in file. file should be a PO or POT file. `‘--fuzzy’`: Synonym for `‘--only-fuzzy --clear-fuzzy’`: It keeps only the fuzzy messages and removes their ‘fuzzy’ mark. `‘--obsolete’`: Synonym for `‘--only-obsolete --clear-obsolete’`: It keeps only the obsolete messages and makes them non-obsolete.

### 9.8.5 Input file syntax

`‘-P’`: `‘--properties-input’`: Assume the input file is a Java ResourceBundle in Java `.properties` syntax, not in PO file syntax. `‘--stringtable-input’`: Assume the input file is a NeXTstep/GNUstep localized resource file in `.strings` syntax, not in PO file syntax.

### 9.8.6 Output details

`‘--color’`: `‘--color=when’`: Specify whether or when to use colors and other text attributes. See section 9.11.1 The `--color` option for details. `‘--style=style_file’`: Specify the CSS style rule file to use for `--color`. See section 9.11.3 The `--style` option for details. `‘--force-po’`: Always write an output file even if it contains no message. `‘-i’`: `‘--indent’`: Write the .po file using indented style. `‘--no-location’`: Do not write `‘#: filename:line’` lines. `‘-n’`: `‘--add-location’`: Generate `‘#: filename:line’` lines (default). `‘--strict’`: Write out a strict Uniforum conforming PO file. Note that this Uniforum format should be avoided because it doesn't support the GNU extensions. `‘-p’`: `‘--properties-output’`: Write out a Java ResourceBundle in Java `.properties` syntax. Note that this file format doesn't support plural forms and silently drops obsolete messages. `‘--stringtable-output’`: Write out a NeXTstep/GNUstep localized resource file in `.strings` syntax. Note that this file format doesn't support plural forms. `‘-w number’`: `‘--width=number’`: Set the output page width. Long strings in the output files will be split across multiple lines in order to ensure that each line's width (= number of screen columns) is less or equal to the given number. `‘--no-wrap’`: Do not break long message lines. Message lines whose width exceeds the output page width will not be split into several lines. Only file reference lines which are wider than the output page width will be split. `‘-s’`: `‘--sort-output’`: Generate sorted output. Note that using this option makes it much harder for the translator to understand each message's context. `‘-F’`: `‘--sort-by-file’`: Sort output by file location.

### 9.8.7 Informative output

`‘-h’`: `‘--help’`: Display this help and exit. `‘-V’`: `‘--version’`: Output version information and exit.

## 9.9 Invoking the `msgen` Program

```
msgen [option] inputfile
```

The `msgen` program creates an English translation catalog. The
input file is the last created English PO file, or a PO Template file
(generally created by xgettext). Untranslated entries are assigned a
translation that is identical to the msgid.

Note: `‘msginit --no-translator --locale=en’` performs a very similar
task. The main difference is that `msginit` cares specially about
the header entry, whereas `msgen` doesn't.

### 9.9.1 Input file location

`‘inputfile’`: Input PO or POT file. `‘-D directory’`: `‘--directory=directory’`: Add directory to the list of directories. Source files are searched relative to this list of directories. The resulting ‘.po’ file will be written relative to the current directory, though.

If inputfile is `‘-’`, standard input is read.

### 9.9.2 Output file location

`‘-o file’`: `‘--output-file=file’`: Write output to specified file.

The results are written to standard output if no output file is specified
or if it is `‘-’`.

### 9.9.3 Input file syntax

`‘-P’`: `‘--properties-input’`: Assume the input file is a Java ResourceBundle in Java `.properties` syntax, not in PO file syntax. `‘--stringtable-input’`: Assume the input file is a NeXTstep/GNUstep localized resource file in `.strings` syntax, not in PO file syntax.

### 9.9.4 Output details

`‘--lang=catalogname’`: Specify the `‘Language’` field to be used in the header entry. See section 6.2 Filling in the Header Entry for the meaning of this field. Note: The `‘Language-Team’` and `‘Plural-Forms’` fields are not set by this option. `‘--color’`: `‘--color=when’`: Specify whether or when to use colors and other text attributes. See section 9.11.1 The `--color` option for details. `‘--style=style_file’`: Specify the CSS style rule file to use for `--color`. See section 9.11.3 The `--style` option for details. `‘--force-po’`: Always write an output file even if it contains no message. `‘-i’`: `‘--indent’`: Write the .po file using indented style. `‘--no-location’`: Do not write `‘#: filename:line’` lines. `‘--add-location’`: Generate `‘#: filename:line’` lines (default). `‘--strict’`: Write out a strict Uniforum conforming PO file. Note that this Uniforum format should be avoided because it doesn't support the GNU extensions. `‘-p’`: `‘--properties-output’`: Write out a Java ResourceBundle in Java `.properties` syntax. Note that this file format doesn't support plural forms and silently drops obsolete messages. `‘--stringtable-output’`: Write out a NeXTstep/GNUstep localized resource file in `.strings` syntax. Note that this file format doesn't support plural forms. `‘-w number’`: `‘--width=number’`: Set the output page width. Long strings in the output files will be split across multiple lines in order to ensure that each line's width (= number of screen columns) is less or equal to the given number. `‘--no-wrap’`: Do not break long message lines. Message lines whose width exceeds the output page width will not be split into several lines. Only file reference lines which are wider than the output page width will be split. `‘-s’`: `‘--sort-output’`: Generate sorted output. Note that using this option makes it much harder for the translator to understand each message's context. `‘-F’`: `‘--sort-by-file’`: Sort output by file location.

### 9.9.5 Informative output

`‘-h’`: `‘--help’`: Display this help and exit. `‘-V’`: `‘--version’`: Output version information and exit.

## 9.10 Invoking the `msgexec` Program

```
msgexec [option] command [command-option]
```

The `msgexec` program applies a command to all translations of a
translation catalog.
The command can be any program that reads a translation from standard
input. It is invoked once for each translation. Its output becomes
msgexec's output. `msgexec`'s return code is the maximum return code
across all invocations.

A special builtin command called `‘0’` outputs the translation, followed
by a null byte. The output of `‘msgexec 0’` is suitable as input for
`‘xargs -0’`.

During each command invocation, the environment variable
`MSGEXEC_MSGID` is bound to the message's msgid, and the environment
variable `MSGEXEC_LOCATION` is bound to the location in the PO file
of the message. If the message has a context, the environment variable
`MSGEXEC_MSGCTXT` is bound to the message's msgctxt, otherwise it is
unbound.

Note: It is your responsibility to ensure that the command can cope
with input encoded in the translation catalog's encoding. If the
command wants input in a particular encoding, you can in a first step
convert the translation catalog to that encoding using the `‘msgconv’`
program, before invoking `‘msgexec’`. If the command wants input
in the locale's encoding, but you want to avoid the locale's encoding, then
you can first convert the translation catalog to UTF-8 using the
`‘msgconv’` program and then make `‘msgexec’` work in an UTF-8
locale, by using the `LC_ALL` environment variable.

### 9.10.1 Input file location

`‘-i inputfile’`: `‘--input=inputfile’`: Input PO file. `‘-D directory’`: `‘--directory=directory’`: Add directory to the list of directories. Source files are searched relative to this list of directories. The resulting ‘.po’ file will be written relative to the current directory, though.

If no inputfile is given or if it is `‘-’`, standard input is read.

### 9.10.2 Input file syntax

`‘-P’`: `‘--properties-input’`: Assume the input file is a Java ResourceBundle in Java `.properties` syntax, not in PO file syntax. `‘--stringtable-input’`: Assume the input file is a NeXTstep/GNUstep localized resource file in `.strings` syntax, not in PO file syntax.

### 9.10.3 Informative output

`‘-h’`: `‘--help’`: Display this help and exit. `‘-V’`: `‘--version’`: Output version information and exit.

## 9.11 Highlighting parts of PO files

Translators are usually only interested in seeing the untranslated and
fuzzy messages of a PO file. Also, when a message is set fuzzy because
the msgid changed, they want to see the differences between the previous
msgid and the current one (especially if the msgid is long and only few
words in it have changed). Finally, it's always welcome to highlight the
different sections of a message in a PO file (comments, msgid, msgstr, etc.).

Such highlighting is possible through the `msgcat` options
`‘--color’` and `‘--style’`.

### 9.11.1 The `--color` option

The `‘--color=when’` option specifies under which conditions
colorized output should be generated. The when part can be one of
the following:

`always`: `yes`: The output will be colorized. `never`: `no`: The output will not be colorized. `auto`: `tty`: The output will be colorized if the output device is a tty, i.e. when the output goes directly to a text screen or terminal emulator window. `html`: The output will be colorized and be in HTML format.

`‘--color’` is equivalent to `‘--color=yes’`. The default is
`‘--color=auto’`.

Thus, a command like `‘msgcat vi.po’` will produce colorized output
when called by itself in a command window. Whereas in a pipe, such as
`‘msgcat vi.po | less -R’`, it will not produce colorized output. To
get colorized output in this situation nevertheless, use the command
`‘msgcat --color vi.po | less -R’`.

The `‘--color=html’` option will produce output that can be viewed in
a browser. This can be useful, for example, for Indic languages,
because the renderic of Indic scripts in browser is usually better than
in terminal emulators.

Note that the output produced with the `--color` option is *not*
a valid PO file in itself. It contains additional terminal-specific escape
sequences or HTML tags. A PO file reader will give a syntax error when
confronted with such content. Except for the `‘--color=html’` case,
you therefore normally don't need to save output produced with the
`--color` option in a file.

### 9.11.2 The environment variable `TERM`

The environment variable `TERM` contains a identifier for the text
window's capabilities. You can get a detailed list of these cababilities
by using the `‘infocmp’` command, using `‘man 5 terminfo’` as a
reference.

When producing text with embedded color directives, `msgcat` looks
at the `TERM` variable. Text windows today typically support at least
8 colors. Often, however, the text window supports 16 or more colors,
even though the `TERM` variable is set to a identifier denoting only
8 supported colors. It can be worth setting the `TERM` variable to
a different value in these cases:

`xterm`: `xterm` is in most cases built with support for 16 colors. It can also be built with support for 88 or 256 colors (but not both). You can try to set `TERM` to either `xterm-16color`, `xterm-88color`, or `xterm-256color`. `rxvt`: `rxvt` is often built with support for 16 colors. You can try to set `TERM` to `rxvt-16color`. `konsole`: `konsole` too is often built with support for 16 colors. You can try to set `TERM` to `konsole-16color` or `xterm-16color`.

After setting `TERM`, you can verify it by invoking
`‘msgcat --color=test’` and seeing whether the output looks like a
reasonable color map.

### 9.11.3 The `--style` option

The `‘--style=style_file’` option specifies the style file to use
when colorizing. It has an effect only when the `--color` option is
effective.

If the `--style` option is not specified, the environment variable
`PO_STYLE` is considered. It is meant to point to the user's
preferred style for PO files.

The default style file is ‘$prefix/share/gettext/styles/po-default.css’,
where `$prefix` is the installation location.

A few style files are predefined:

‘po-vim.css’: This style imitates the look used by vim 7. ‘po-emacs-x.css’: This style imitates the look used by GNU Emacs 21 and 22 in an X11 window. ‘po-emacs-xterm.css’: ‘po-emacs-xterm16.css’: ‘po-emacs-xterm256.css’: This style imitates the look used by GNU Emacs 22 in a terminal of type `‘xterm’` (8 colors) or `‘xterm-16color’` (16 colors) or `‘xterm-256color’` (256 colors), respectively.

You can use these styles without specifying a directory. They are actually
located in ‘$prefix/share/gettext/styles/’, where `$prefix` is the
installation location.

You can also design your own styles. This is described in the next section.

### 9.11.4 Style rules for PO files

The same style file can be used for styling of a PO file, for terminal
output and for HTML output. It is written in CSS (Cascading Style Sheet)
syntax. See http://www.w3.org/TR/css2/cover.html for a formal
definition of CSS. Many HTML authoring tutorials also contain explanations
of CSS.

In the case of HTML output, the style file is embedded in the HTML output.
In the case of text output, the style file is interpreted by the
`msgcat` program. This means, in particular, that when
`@import` is used with relative file names, the file names are

- relative to the resulting HTML file, in the case of HTML output,- relative to the style sheet containing the `@import`, in the case of
    text output. (Actually, `@import`s are not yet supported in this case,
    due to a limitation in `libcroco`.)

CSS rules are built up from selectors and declarations. The declarations
specify graphical properties; the selectors specify specify when they apply.

In PO files, the following simple selectors (based on "CSS classes", see
the CSS2 spec, section 5.8.3) are supported.

- Selectors that apply to entire messages:

  `.header`: This matches the header entry of a PO file. `.translated`: This matches a translated message. `.untranslated`: This matches an untranslated message (i.e. a message with empty translation). `.fuzzy`: This matches a fuzzy message (i.e. a message which has a translation that needs review by the translator). `.obsolete`: This matches an obsolete message (i.e. a message that was translated but is not needed by the current POT file any more).

  - Selectors that apply to parts of a message in PO syntax. Recall the general
    structure of a message in PO syntax:

    ```
    white-space
    #  translator-comments
    #. extracted-comments
    #: reference...
    #, flag...
    #| msgid previous-untranslated-string
    msgid untranslated-string
    msgstr translated-string
    ```

    `.comment`: This matches all comments (translator comments, extracted comments, source file reference comments, flag comments, previous message comments, as well as the entire obsolete messages). `.translator-comment`: This matches the translator comments. `.extracted-comment`: This matches the extracted comments, i.e. the comments placed by the programmer at the attention of the translator. `.reference-comment`: This matches the source file reference comments (entire lines). `.reference`: This matches the individual source file references inside the source file reference comment lines. `.flag-comment`: This matches the flag comment lines (entire lines). `.flag`: This matches the individual flags inside flag comment lines. `.fuzzy-flag`: This matches the `fuzzy' flag inside flag comment lines. `.previous-comment`: This matches the comments containing the previous untranslated string (entire lines). `.previous`: This matches the previous untranslated string including the string delimiters, the associated keywords (`msgid` etc.) and the spaces between them. `.msgid`: This matches the untranslated string including the string delimiters, the associated keywords (`msgid` etc.) and the spaces between them. `.msgstr`: This matches the translated string including the string delimiters, the associated keywords (`msgstr` etc.) and the spaces between them. `.keyword`: This matches the keywords (`msgid`, `msgstr`, etc.). `.string`: This matches strings, including the string delimiters (double quotes).

    - Selectors that apply to parts of strings:

      `.text`: This matches the entire contents of a string (excluding the string delimiters, i.e. the double quotes). `.escape-sequence`: This matches an escape sequence (starting with a backslash). `.format-directive`: This matches a format string directive (starting with a `‘%’` sign in the case of most programming languages, with a `‘{’` in the case of `java-format` and `csharp-format`, with a `‘~’` in the case of `lisp-format` and `scheme-format`, or with `‘$’` in the case of `sh-format`). `.invalid-format-directive`: This matches an invalid format string directive. `.added`: In an untranslated string, this matches a part of the string that was not present in the previous untranslated string. (Not yet implemented in this release.) `.changed`: In an untranslated string or in a previous untranslated string, this matches a part of the string that is changed or replaced. (Not yet implemented in this release.) `.removed`: In a previous untranslated string, this matches a part of the string that is not present in the current untranslated string. (Not yet implemented in this release.)

These selectors can be combined to hierarchical selectors. For example,

```
.msgstr .invalid-format-directive { color: red; }
```

will highlight the invalid format directives in the translated strings.

In text mode, pseudo-classes (CSS2 spec, section 5.11) and pseudo-elements
(CSS2 spec, section 5.12) are not supported.

The declarations in HTML mode are not limited; any graphical attribute
supported by the browsers can be used.

The declarations in text mode are limited to the following properties. Other
properties will be silently ignored.

`color` (CSS2 spec, section 14.1): `background-color` (CSS2 spec, section 14.2.1): These properties is supported. Colors will be adjusted to match the terminal's capabilities. Note that many terminals support only 8 colors. `font-weight` (CSS2 spec, section 15.2.3): This property is supported, but most terminals can only render two different weights: `normal` and `bold`. Values >= 600 are rendered as `bold`. `font-style` (CSS2 spec, section 15.2.3): This property is supported. The values `italic` and `oblique` are rendered the same way. `text-decoration` (CSS2 spec, section 16.3.1): This property is supported, limited to the values `none` and `underline`.

### 9.11.5 Customizing `less` for viewing PO files

The `‘less’` program is a popular text file browser for use in a text
screen or terminal emulator. It also supports text with embedded escape
sequences for colors and text decorations.

You can use `less` to view a PO file like this (assuming an UTF-8
environment):

```
msgcat --to-code=UTF-8 --color xyz.po | less -R
```

You can simplify this to this simple command:

```
less xyz.po
```

after these three preparations:

1. Add the options `‘-R’` and `‘-f’` to the `LESS` environment
   variable. In sh shells:

   ```
   $ LESS="$LESS -R -f"
   $ export LESS
   ```

   - If your system does not already have the ‘lessopen.sh’ and
     ‘lessclose.sh’ scripts, create them and set the `LESSOPEN` and
     `LESSCLOSE` environment variables, as indicated in the manual page
     (`‘man less’`).- Add to ‘lessopen.sh’ a piece of script that recognizes PO files
       through their file extension and invokes `msgcat` on them, producing
       a temporary file. Like this:

       ```
       case "$1" in
         *.po)
           tmpfile=`mktemp "${TMPDIR-/tmp}/less.XXXXXX"`
           msgcat --to-code=UTF-8 --color "$1" > "$tmpfile"
           echo "$tmpfile"
           exit 0
           ;;
       esac
       ```

## 9.12 Writing your own programs that process PO files

For the tasks for which a combination of `‘msgattrib’`, `‘msgcat’` etc.
is not sufficient, a set of C functions is provided in a library, to make it
possible to process PO files in your own programs. When you use this library,
you don't need to write routines to parse the PO file; instead, you retrieve
a pointer in memory to each of messages contained in the PO file. Functions
for writing PO files are not provided at this time.

The functions are declared in the header file `‘<gettext-po.h>’`, and are
defined in a library called `‘libgettextpo’`.

Data Type: **po\_file\_t**: This is a pointer type that refers to the contents of a PO file, after it has been read into memory.

Data Type: **po\_message\_iterator\_t**: This is a pointer type that refers to an iterator that produces a sequence of messages.

Data Type: **po\_message\_t**: This is a pointer type that refers to a message of a PO file, including its translation.

Function: po\_file\_t **po\_file\_read** *(const char \*filename)*: The `po_file_read` function reads a PO file into memory. The file name is given as argument. The return value is a handle to the PO file's contents, valid until `po_file_free` is called on it. In case of error, the return value is `NULL`, and `errno` is set.

Function: void **po\_file\_free** *(po\_file\_t file)*: The `po_file_free` function frees a PO file's contents from memory, including all messages that are only implicitly accessible through iterators.

Function: const char \* const \* **po\_file\_domains** *(po\_file\_t file)*: The `po_file_domains` function returns the domains for which the given PO file has messages. The return value is a `NULL` terminated array which is valid as long as the file handle is valid. For PO files which contain no `‘domain’` directive, the return value contains only one domain, namely the default domain `"messages"`.

Function: po\_message\_iterator\_t **po\_message\_iterator** *(po\_file\_t file, const char \*domain)*: The `po_message_iterator` returns an iterator that will produce the messages of file that belong to the given domain. If domain is `NULL`, the default domain is used instead. To list the messages, use the function `po_next_message` repeatedly.

Function: void **po\_message\_iterator\_free** *(po\_message\_iterator\_t iterator)*: The `po_message_iterator_free` function frees an iterator previously allocated through the `po_message_iterator` function.

Function: po\_message\_t **po\_next\_message** *(po\_message\_iterator\_t iterator)*: The `po_next_message` function returns the next message from iterator and advances the iterator. It returns `NULL` when the iterator has reached the end of its message list.

The following functions returns details of a `po_message_t`. Recall
that the results are valid as long as the file handle is valid.

Function: const char \* **po\_message\_msgid** *(po\_message\_t message)*: The `po_message_msgid` function returns the `msgid` (untranslated English string) of a message. This is guaranteed to be non-`NULL`.

Function: const char \* **po\_message\_msgid\_plural** *(po\_message\_t message)*: The `po_message_msgid_plural` function returns the `msgid_plural` (untranslated English plural string) of a message with plurals, or `NULL` for a message without plural.

Function: const char \* **po\_message\_msgstr** *(po\_message\_t message)*: The `po_message_msgstr` function returns the `msgstr` (translation) of a message. For an untranslated message, the return value is an empty string.

Function: const char \* **po\_message\_msgstr\_plural** *(po\_message\_t message, int index)*: The `po_message_msgstr_plural` function returns the `msgstr[index]` of a message with plurals, or `NULL` when the index is out of range or for a message without plural.

Here is an example code how these functions can be used.

```
const char *filename = ...;
po_file_t file = po_file_read (filename);

if (file == NULL)
  error (EXIT_FAILURE, errno, "couldn't open the PO file %s", filename);
{
  const char * const *domains = po_file_domains (file);
  const char * const *domainp;

  for (domainp = domains; *domainp; domainp++)
    {
      const char *domain = *domainp;
      po_message_iterator_t iterator = po_message_iterator (file, domain);

      for (;;)
        {
          po_message_t *message = po_next_message (iterator);

          if (message == NULL)
            break;
          {
            const char *msgid = po_message_msgid (message);
            const char *msgstr = po_message_msgstr (message);

            ...
          }
        }
      po_message_iterator_free (iterator);
    }
}
po_file_free (file);
```

---

Go to the first, previous, next, last section, table of contents.
