# Supplementary material for: rDock: A Fast, Versatile and Open Source Program for Docking Ligands to Proteins and Nucleic Acids
Source: PLoS Comput Biol. 2014 Apr 10;10(4):e1003571. doi: 10.1371/journal.pcbi.1003571 (PMC3983074; doi:10.1371/journal.pcbi.1003571)
Supplement: Software S1 — Compressed file with the source code of the rDock software for ligand docking to Proteins and Nucleic Acids. (GZ) [file pcbi.1003571.s010.gz › rDock_2013.1_src/gettext-0.18.tar.gz!/gettext-0.18/gettext-tools/doc/gettext_25.html]

GNU gettext utilities - General Index

Go to the first, previous, next, last section, table of contents.

---

# General Index

Jump to:
&
-
\_
-
a
-
b
-
c
-
d
-
e
-
f
-
g
-
h
-
i
-
j
-
k
-
l
-
m
-
n
-
o
-
p
-
q
-
r
-
s
-
t
-
u
-
v
-
w
-
x
-
y

## &

- ‘ABOUT-NLS’ file- ‘acconfig.h’ file- ‘aclocal.m4’ file- ‘config.h.in’ file- ‘gettext.h’ file- ‘LINGUAS’ file- ‘Makefile.in.in’ extensions- ‘Makevars’ file- ‘mkinstalldirs’ file- ‘POTFILES.in’ file

## \_

- `_`, a macro to mark strings for translation- `_nl_msg_cat_cntr`

## a

- accumulating translations- adding keywords, `xgettext`- ambiguities- apply a filter to translations- apply command to all translations in a catalog- Arabic digits- attribute manipulation- attribute, fuzzy- attributes of a PO file entry- attributes, manipulating- autoconf macros for `gettext`- `autopoint` program, usage- auxiliary PO file- available translations- awk

## b

- backup old file, and `msgmerge` program- bash- bibliography- big picture- bind\_textdomain\_codeset- Boost format strings- bug report address

## c

- C and C-like languages- C trigraphs- C#- C# mode, and `msgfmt` program- C# mode, and `msgunfmt` program- C# resources mode, and `msgfmt` program- C# resources mode, and `msgunfmt` program- C#, string concatenation- catalog encoding and `msgexec` output- `catclose`, a `catgets` function- `catgets`, X/Open specification- `catgets`, a `catgets` function- `catopen`, a `catgets` function- character encoding- charset conversion at runtime- charset of PO files- check format strings- checking of translations- clisp- clisp C sources- codeset- comments in PO files- comments, automatic- comments, extracted- comments, translator- Common Lisp- compare PO files- comparison of interfaces- compatibility with X/Open `msgfmt`- compendium- compendium, creating- concatenate PO files- concatenating PO files into a compendium- concatenation of strings- context- context, argument specification in `xgettext`- context, in MO files- context, in PO files- control characters- convert binary message catalog into PO file- convert translations to a different encoding- converting a package to use `gettext`- country codes- create new PO file- creating a new PO file- creating compendia- currency symbols

## d

- date format- dcngettext- dcpgettext- dcpgettext\_expr- debugging messages marked as format strings- dialect- disabling NLS- distribution tarball- dngettext- dollar substitution- domain ambiguities- dpgettext- dpgettext\_expr- duplicate elimination- duplicate removal

## e

- editing comments in PO files- Editing PO Files- editing translations- Emacs Lisp- Emacs PO Mode- encoding- encoding conversion- encoding conversion at runtime- encoding for your language- encoding list- encoding of PO files- environment variables- `envsubst` program, usage- `eval_gettext` function, usage- `eval_ngettext` function, usage- evolution of packages- extracting parts of a PO file into a compendium

## f

- FDL, GNU Free Documentation License- file format, ‘.mo’- file format, ‘.po’- files, ‘.po’ and ‘.mo’- files, ‘.pot’- filter messages according to attributes- find common messages- force use of fuzzy entries- format strings- Free Pascal- function attribute, \_\_format\_\_- function attribute, \_\_format\_arg\_\_- fuzzy entries

## g

- gawk- GCC-source- generate binary message catalog from PO file- generate translation catalog in English- `gettext` files- `gettext` installation- `gettext` interface- `gettext` program, usage- `gettext`, a programmer's view- `gettext` vs `catgets`- `gettextize` program, usage- GNOME PO file editor- GPL, GNU General Public License- GUI programs- guile

## h

- hash table, inside MO files- he, she, and they- header entry of a PO file- help option- history of GNU `gettext`

## i

- i18n- importing PO files- include file ‘libintl.h’, include file ‘libintl.h’, include file ‘libintl.h’, include file ‘libintl.h’- initialization- initialize new PO file- initialize translations from a compendium- installing `gettext`- interface to `catgets`- internationalization- `inttypes.h`- ISO 3166- ISO 639

## j

- Java- Java mode, and `msgfmt` program- Java mode, and `msgunfmt` program- Java, string concatenation

## k

- KDE format strings- KDE PO file editor- keyboard accelerator checking

## l

- l10n- language codes- language selection- language selection at runtime- large package- LGPL, GNU Lesser General Public License- `libiconv` library- `libintl` for C#- `libintl` for Java- `libintl` library- `librep` Lisp- License, GNU FDL- License, GNU GPL- License, GNU LGPL- Licenses- link with ‘libintl’- Linux, Linux, Linux- Lisp- list of translation teams, where to find- locale categories, locale categories- locale category, LC\_ALL- locale category, LC\_COLLATE- locale category, LC\_CTYPE, locale category, LC\_CTYPE, locale category, LC\_CTYPE- locale category, LC\_MESSAGES, locale category, LC\_MESSAGES- locale category, LC\_MONETARY, locale category, LC\_MONETARY- locale category, LC\_NUMERIC, locale category, LC\_NUMERIC- locale category, LC\_RESPONSES- locale category, LC\_TIME, locale category, LC\_TIME- `locale` program- localization- lookup message translation, lookup message translation- lookup plural message translation, lookup plural message translation

## m

- magic signature of MO files- manipulating PO files- marking Perl sources- marking string initializers- marking strings that require translation- marking strings, preparations- marking translatable strings- markup- menu entries- menu, keyboard accelerator support- merge PO files- merging two PO files- message catalog files location- messages- migration from earlier versions of `gettext`- mnemonics of menu entries- MO file's format- modify message attributes- `msgattrib` program, usage- `msgcat` program, usage- `msgcmp` program, usage- `msgcomm` program, usage- `msgconv` program, usage- `msgen` program, usage- `msgexec` program, usage- `msgfilter` filter and catalog encoding- `msgfilter` program, usage- `msgfmt` program, usage- `msggrep` program, usage- `msginit` program, usage- `msgmerge` program, usage- `msgunfmt` program, usage- `msguniq` program, usage- multi-line strings

## n

- `N_`, a convenience macro- Native Language Support- Natural Language Support- newlines in PO files- ngettext- `ngettext` program, usage- NLS- number format

## o

- Object Pascal- obsolete entries- optimization of `gettext` functions- orthography- outdigits- output to stdout, `xgettext`- overview of `gettext`

## p

- package and version declaration in ‘configure.ac’- package build and installation options- package distributor's view of `gettext`- package installer's view of `gettext`- package maintainer's view of `gettext`- paragraphs- Pascal- Perl- Perl default keywords- Perl invalid string interpolation- Perl long lines- Perl parentheses- Perl pitfalls- Perl quote-like expressions- Perl special keywords for hash-lookups- Perl valid string interpolation- pgettext- pgettext\_expr- PHP- Pike- plural form formulas- plural forms- plural forms, in MO files- plural forms, in PO files- plural forms, translating- PO files' format- PO mode (Emacs) commands- PO template file- po\_file\_domains- po\_file\_free- po\_file\_read- po\_message\_iterator- po\_message\_iterator\_free- po\_message\_msgid- po\_message\_msgid\_plural- po\_message\_msgstr- po\_message\_msgstr\_plural- po\_next\_message- portability problems with `sed`- preparing programs for translation- preparing shell scripts for translation- problems with `catgets` interface- programming languages- Python

## q

- Qt format strings- Qt mode, and `msgfmt` program- quotation marks, quotation marks- quote characters, use in PO files

## r

- `recode-sr-latin` program- related reading- release- RST

## s

- Scheme- scripting languages- search messages in a catalog- selecting message language- sentences- setting up `gettext` at build time- setting up `gettext` at run time- several domains- sex- she, he, and they- shell format string- shell scripts- Smalltalk- sorting `msgcat` output- sorting `msgmerge` output- sorting `msgunfmt` output- sorting output of `xgettext`- specifying plural form in a PO file- standard output, and `msgcat`- standard output, and `msgmerge` program- string concatenation- string normalization in entries- style- supported languages, `xgettext`

## t

- Tcl- Tcl mode, and `msgfmt` program- Tcl mode, and `msgunfmt` program- template PO file- testing ‘.po’ files for equivalence- Tk's scripting language- translated entries- translating menu entries- translation aspects- Translation Matrix- Translation Project- turning off NLS support- tutorial of `gettext` usage

## u

- unify duplicate translations- untranslated entries- update translations from a compendium- upgrading to new versions of `gettext`

## v

- version control for backup files, `msgmerge`

## w

- `wxWidgets` library

## x

- `xargs`, and output from `msgexec`- `xgettext` program, usage- `xmodmap` program, and typing quotation marks

## y

- YaST2 scripting language- YCP

---

Go to the first, previous, next, last section, table of contents.
