# Supplementary material for: rDock: A Fast, Versatile and Open Source Program for Docking Ligands to Proteins and Nucleic Acids
Source: PLoS Comput Biol. 2014 Apr 10;10(4):e1003571. doi: 10.1371/journal.pcbi.1003571 (PMC3983074; doi:10.1371/journal.pcbi.1003571)
Supplement: Software S1 — Compressed file with the source code of the rDock software for ligand docking to Proteins and Nucleic Acids. (GZ) [file pcbi.1003571.s010.gz › rDock_2013.1_src/gettext-0.18.tar.gz!/gettext-0.18/gettext-tools/doc/gettext_3.html]

GNU gettext utilities - 3 The Format of PO Files

Go to the first, previous, next, last section, table of contents.

---

# 3 The Format of PO Files

The GNU `gettext` toolset helps programmers and translators
at producing, updating and using translation files, mainly those
PO files which are textual, editable files. This chapter explains
the format of PO files.

A PO file is made up of many entries, each entry holding the relation
between an original untranslated string and its corresponding
translation. All entries in a given PO file usually pertain
to a single project, and all translations are expressed in a single
target language. One PO file *entry* has the following schematic
structure:

```
white-space
#  translator-comments
#. extracted-comments
#: reference...
#, flag...
#| msgid previous-untranslated-string
msgid untranslated-string
msgstr translated-string
```

The general structure of a PO file should be well understood by
the translator. When using PO mode, very little has to be known
about the format details, as PO mode takes care of them for her.

A simple entry can look like this:

```
#: lib/error.c:116
msgid "Unknown system error"
msgstr "Error desconegut del sistema"
```

Entries begin with some optional white space. Usually, when generated
through GNU `gettext` tools, there is exactly one blank line
between entries. Then comments follow, on lines all starting with the
character `#`. There are two kinds of comments: those which have
some white space immediately following the `#` - the translator
comments -, which comments are created and maintained exclusively by the
translator, and those which have some non-white character just after the
`#` - the automatic comments -, which comments are created and
maintained automatically by GNU `gettext` tools. Comment lines
starting with `#.` contain comments given by the programmer, directed
at the translator; these comments are called extracted comments
because the `xgettext` program extracts them from the program's
source code. Comment lines starting with `#:` contain references to
the program's source code. Comment lines starting with `#,` contain
flags; more about these below. Comment lines starting with `#|`
contain the previous untranslated string for which the translator gave
a translation.

All comments, of either kind, are optional.

After white space and comments, entries show two strings, namely
first the untranslated string as it appears in the original program
sources, and then, the translation of this string. The original
string is introduced by the keyword `msgid`, and the translation,
by `msgstr`. The two strings, untranslated and translated,
are quoted in various ways in the PO file, using `"`
delimiters and `\` escapes, but the translator does not really
have to pay attention to the precise quoting format, as PO mode fully
takes care of quoting for her.

The `msgid` strings, as well as automatic comments, are produced
and managed by other GNU `gettext` tools, and PO mode does not
provide means for the translator to alter these. The most she can
do is merely deleting them, and only by deleting the whole entry.
On the other hand, the `msgstr` string, as well as translator
comments, are really meant for the translator, and PO mode gives her
the full control she needs.

The comment lines beginning with `#,` are special because they are
not completely ignored by the programs as comments generally are. The
comma separated list of flags is used by the `msgfmt`
program to give the user some better diagnostic messages. Currently
there are two forms of flags defined:

`fuzzy`: This flag can be generated by the `msgmerge` program or it can be inserted by the translator herself. It shows that the `msgstr` string might not be a correct translation (anymore). Only the translator can judge if the translation requires further modification, or is acceptable as is. Once satisfied with the translation, she then removes this `fuzzy` attribute. The `msgmerge` program inserts this when it combined the `msgid` and `msgstr` entries after fuzzy search only. See section 8.3.6 Fuzzy Entries. `c-format`: `no-c-format`: These flags should not be added by a human. Instead only the `xgettext` program adds them. In an automated PO file processing system as proposed here, the user's changes would be thrown away again as soon as the `xgettext` program generates a new template file. The `c-format` flag indicates that the untranslated string and the translation are supposed to be C format strings. The `no-c-format` flag indicates that they are not C format strings, even though the untranslated string happens to look like a C format string (with `‘%’` directives). When the `c-format` flag is given for a string the `msgfmt` program does some more tests to check the validity of the translation. See section 10.1 Invoking the `msgfmt` Program, section 4.6 Special Comments preceding Keywords and section 15.3.1 C Format Strings. `objc-format`: `no-objc-format`: Likewise for Objective C, see section 15.3.2 Objective C Format Strings. `sh-format`: `no-sh-format`: Likewise for Shell, see section 15.3.3 Shell Format Strings. `python-format`: `no-python-format`: Likewise for Python, see section 15.3.4 Python Format Strings. `lisp-format`: `no-lisp-format`: Likewise for Lisp, see section 15.3.5 Lisp Format Strings. `elisp-format`: `no-elisp-format`: Likewise for Emacs Lisp, see section 15.3.6 Emacs Lisp Format Strings. `librep-format`: `no-librep-format`: Likewise for librep, see section 15.3.7 librep Format Strings. `scheme-format`: `no-scheme-format`: Likewise for Scheme, see section 15.3.8 Scheme Format Strings. `smalltalk-format`: `no-smalltalk-format`: Likewise for Smalltalk, see section 15.3.9 Smalltalk Format Strings. `java-format`: `no-java-format`: Likewise for Java, see section 15.3.10 Java Format Strings. `csharp-format`: `no-csharp-format`: Likewise for C#, see section 15.3.11 C# Format Strings. `awk-format`: `no-awk-format`: Likewise for awk, see section 15.3.12 awk Format Strings. `object-pascal-format`: `no-object-pascal-format`: Likewise for Object Pascal, see section 15.3.13 Object Pascal Format Strings. `ycp-format`: `no-ycp-format`: Likewise for YCP, see section 15.3.14 YCP Format Strings. `tcl-format`: `no-tcl-format`: Likewise for Tcl, see section 15.3.15 Tcl Format Strings. `perl-format`: `no-perl-format`: Likewise for Perl, see section 15.3.16 Perl Format Strings. `perl-brace-format`: `no-perl-brace-format`: Likewise for Perl brace, see section 15.3.16 Perl Format Strings. `php-format`: `no-php-format`: Likewise for PHP, see section 15.3.17 PHP Format Strings. `gcc-internal-format`: `no-gcc-internal-format`: Likewise for the GCC sources, see section 15.3.18 GCC internal Format Strings. `gfc-internal-format`: `no-gfc-internal-format`: Likewise for the GNU Fortran Compiler sources, see section 15.3.19 GFC internal Format Strings. `qt-format`: `no-qt-format`: Likewise for Qt, see section 15.3.20 Qt Format Strings. `qt-plural-format`: `no-qt-plural-format`: Likewise for Qt plural forms, see section 15.3.21 Qt Format Strings. `kde-format`: `no-kde-format`: Likewise for KDE, see section 15.3.22 KDE Format Strings. `boost-format`: `no-boost-format`: Likewise for Boost, see section 15.3.23 Boost Format Strings.

It is also possible to have entries with a context specifier. They look like
this:

```
white-space
#  translator-comments
#. extracted-comments
#: reference...
#, flag...
#| msgctxt previous-context
#| msgid previous-untranslated-string
msgctxt context
msgid untranslated-string
msgstr translated-string
```

The context serves to disambiguate messages with the same
untranslated-string. It is possible to have several entries with
the same untranslated-string in a PO file, provided that they each
have a different context. Note that an empty context string
and an absent `msgctxt` line do not mean the same thing.

A different kind of entries is used for translations which involve
plural forms.

```
white-space
#  translator-comments
#. extracted-comments
#: reference...
#, flag...
#| msgid previous-untranslated-string-singular
#| msgid_plural previous-untranslated-string-plural
msgid untranslated-string-singular
msgid_plural untranslated-string-plural
msgstr[0] translated-string-case-0
...
msgstr[N] translated-string-case-n
```

Such an entry can look like this:

```
#: src/msgcmp.c:338 src/po-lex.c:699
#, c-format
msgid "found %d fatal error"
msgid_plural "found %d fatal errors"
msgstr[0] "s'ha trobat %d error fatal"
msgstr[1] "s'han trobat %d errors fatals"
```

Here also, a `msgctxt` context can be specified before `msgid`,
like above.

Here, additional kinds of flags can be used:

`range:`: This flag is followed by a range of non-negative numbers, using the syntax `range: minimum-value..maximum-value`. It designates the possible values that the numeric parameter of the message can take. In some languages, translators may produce slightly better translations if they know that the value can only take on values between 0 and 10, for example.

The previous-untranslated-string is optionally inserted by the
`msgmerge` program, at the same time when it marks a message fuzzy.
It helps the translator to see which changes were done by the developers
on the untranslated-string.

It happens that some lines, usually whitespace or comments, follow the
very last entry of a PO file. Such lines are not part of any entry,
and will be dropped when the PO file is processed by the tools, or may
disturb some PO file editors.

The remainder of this section may be safely skipped by those using
a PO file editor, yet it may be interesting for everybody to have a better
idea of the precise format of a PO file. On the other hand, those
wishing to modify PO files by hand should carefully continue reading on.

Each of untranslated-string and translated-string respects
the C syntax for a character string, including the surrounding quotes
and embedded backslashed escape sequences. When the time comes
to write multi-line strings, one should not use escaped newlines.
Instead, a closing quote should follow the last character on the
line to be continued, and an opening quote should resume the string
at the beginning of the following PO file line. For example:

```
msgid ""
"Here is an example of how one might continue a very long string\n"
"for the common case the string represents multi-line output.\n"
```

In this example, the empty string is used on the first line, to
allow better alignment of the `H` from the word `‘Here’`
over the `f` from the word `‘for’`. In this example, the
`msgid` keyword is followed by three strings, which are meant
to be concatenated. Concatenating the empty string does not change
the resulting overall string, but it is a way for us to comply with
the necessity of `msgid` to be followed by a string on the same
line, while keeping the multi-line presentation left-justified, as
we find this to be a cleaner disposition. The empty string could have
been omitted, but only if the string starting with `‘Here’` was
promoted on the first line, right after `msgid`.(2) It was not really necessary
either to switch between the two last quoted strings immediately after
the newline `‘\n’`, the switch could have occurred after *any*
other character, we just did it this way because it is neater.

One should carefully distinguish between end of lines marked as
`‘\n’` *inside* quotes, which are part of the represented
string, and end of lines in the PO file itself, outside string quotes,
which have no incidence on the represented string.

Outside strings, white lines and comments may be used freely.
Comments start at the beginning of a line with `‘#’` and extend
until the end of the PO file line. Comments written by translators
should have the initial `‘#’` immediately followed by some white
space. If the `‘#’` is not immediately followed by white space,
this comment is most likely generated and managed by specialized GNU
tools, and might disappear or be replaced unexpectedly when the PO
file is given to `msgmerge`.

---

Go to the first, previous, next, last section, table of contents.
