# Supplementary material for: rDock: A Fast, Versatile and Open Source Program for Docking Ligands to Proteins and Nucleic Acids
Source: PLoS Comput Biol. 2014 Apr 10;10(4):e1003571. doi: 10.1371/journal.pcbi.1003571 (PMC3983074; doi:10.1371/journal.pcbi.1003571)
Supplement: Software S1 — Compressed file with the source code of the rDock software for ligand docking to Proteins and Nucleic Acids. (GZ) [file pcbi.1003571.s010.gz › rDock_2013.1_src/gettext-0.18.tar.gz!/gettext-0.18/gettext-tools/doc/gettext_15.html]

GNU gettext utilities - 15 Other Programming Languages

Go to the first, previous, next, last section, table of contents.

---

# 15 Other Programming Languages

While the presentation of `gettext` focuses mostly on C and
implicitly applies to C++ as well, its scope is far broader than that:
Many programming languages, scripting languages and other textual data
like GUI resources or package descriptions can make use of the gettext
approach.

## 15.1 The Language Implementor's View

All programming and scripting languages that have the notion of strings
are eligible to supporting `gettext`. Supporting `gettext`
means the following:

1. You should add to the language a syntax for translatable strings. In
   principle, a function call of `gettext` would do, but a shorthand
   syntax helps keeping the legibility of internationalized programs. For
   example, in C we use the syntax `_("string")`, and in GNU awk we use
   the shorthand `_"string"`.- You should arrange that evaluation of such a translatable string at
     runtime calls the `gettext` function, or performs equivalent
     processing.- Similarly, you should make the functions `ngettext`,
       `dcgettext`, `dcngettext` available from within the language.
       These functions are less often used, but are nevertheless necessary for
       particular purposes: `ngettext` for correct plural handling, and
       `dcgettext` and `dcngettext` for obeying other locale-related
       environment variables than `LC_MESSAGES`, such as `LC_TIME` or
       `LC_MONETARY`. For these latter functions, you need to make the
       `LC_*` constants, available in the C header `<locale.h>`,
       referenceable from within the language, usually either as enumeration
       values or as strings.- You should allow the programmer to designate a message domain, either by
         making the `textdomain` function available from within the
         language, or by introducing a magic variable called `TEXTDOMAIN`.
         Similarly, you should allow the programmer to designate where to search
         for message catalogs, by providing access to the `bindtextdomain`
         function.- You should either perform a `setlocale (LC_ALL, "")` call during
           the startup of your language runtime, or allow the programmer to do so.
           Remember that gettext will act as a no-op if the `LC_MESSAGES` and
           `LC_CTYPE` locale categories are not both set.- A programmer should have a way to extract translatable strings from a
             program into a PO file. The GNU `xgettext` program is being
             extended to support very different programming languages. Please
             contact the GNU `gettext` maintainers to help them doing this. If
             the string extractor is best integrated into your language's parser, GNU
             `xgettext` can function as a front end to your string extractor.- The language's library should have a string formatting facility where
               the arguments of a format string are denoted by a positional number or a
               name. This is needed because for some languages and some messages with
               more than one substitutable argument, the translation will need to
               output the substituted arguments in different order. See section 4.6 Special Comments preceding Keywords.- If the language has more than one implementation, and not all of the
                 implementations use `gettext`, but the programs should be portable
                 across implementations, you should provide a no-i18n emulation, that
                 makes the other implementations accept programs written for yours,
                 without actually translating the strings.- To help the programmer in the task of marking translatable strings,
                   which is sometimes performed using the Emacs PO mode (see section 4.5 Marking Translatable Strings),
                   you are welcome to
                   contact the GNU `gettext` maintainers, so they can add support for
                   your language to ‘po-mode.el’.

On the implementation side, three approaches are possible, with
different effects on portability and copyright:

- You may integrate the GNU `gettext`'s ‘intl/’ directory in
  your package, as described in section 13 The Maintainer's View. This allows you to
  have internationalization on all kinds of platforms. Note that when you
  then distribute your package, it legally falls under the GNU General
  Public License, and the GNU project will be glad about your contribution
  to the Free Software pool.- You may link against GNU `gettext` functions if they are found in
    the C library. For example, an autoconf test for `gettext()` and
    `ngettext()` will detect this situation. For the moment, this test
    will succeed on GNU systems and not on other platforms. No severe
    copyright restrictions apply.- You may emulate or reimplement the GNU `gettext` functionality.
      This has the advantage of full portability and no copyright
      restrictions, but also the drawback that you have to reimplement the GNU
      `gettext` features (such as the `LANGUAGE` environment
      variable, the locale aliases database, the automatic charset conversion,
      and plural handling).

## 15.2 The Programmer's View

For the programmer, the general procedure is the same as for the C
language. The Emacs PO mode marking supports other languages, and the GNU
`xgettext` string extractor recognizes other languages based on the
file extension or a command-line option. In some languages,
`setlocale` is not needed because it is already performed by the
underlying language runtime.

## 15.3 The Translator's View

The translator works exactly as in the C language case. The only
difference is that when translating format strings, she has to be aware
of the language's particular syntax for positional arguments in format
strings.

### 15.3.1 C Format Strings

C format strings are described in POSIX (IEEE P1003.1 2001), section
XSH 3 fprintf(),
http://www.opengroup.org/onlinepubs/007904975/functions/fprintf.html.
See also the fprintf() manual page,
http://www.linuxvalley.it/encyclopedia/ldp/manpage/man3/printf.3.php,
http://informatik.fh-wuerzburg.de/student/i510/man/printf.html.

Although format strings with positions that reorder arguments, such as

```
"Only %2$d bytes free on '%1$s'."
```

which is semantically equivalent to

```
"'%s' has only %d bytes free."
```

are a POSIX/XSI feature and not specified by ISO C 99, translators can rely
on this reordering ability: On the few platforms where `printf()`,
`fprintf()` etc. don't support this feature natively, ‘libintl.a’
or ‘libintl.so’ provides replacement functions, and GNU `<libintl.h>`
activates these replacement functions automatically.

As a special feature for Farsi (Persian) and maybe Arabic, translators can
insert an `‘I’` flag into numeric format directives. For example, the
translation of `"%d"` can be `"%Id"`. The effect of this flag,
on systems with GNU `libc`, is that in the output, the ASCII digits are
replaced with the `‘outdigits’` defined in the `LC_CTYPE` locale
category. On other systems, the `gettext` function removes this flag,
so that it has no effect.

Note that the programmer should *not* put this flag into the
untranslated string. (Putting the `‘I’` format directive flag into an
msgid string would lead to undefined behaviour on platforms without
glibc when NLS is disabled.)

### 15.3.2 Objective C Format Strings

Objective C format strings are like C format strings. They support an
additional format directive: "$@", which when executed consumes an argument
of type `Object *`.

### 15.3.3 Shell Format Strings

Shell format strings, as supported by GNU gettext and the `‘envsubst’`
program, are strings with references to shell variables in the form
`$variable` or `${variable}`. References of the form
`${variable-default}`,
`${variable:-default}`,
`${variable=default}`,
`${variable:=default}`,
`${variable+replacement}`,
`${variable:+replacement}`,
`${variable?ignored}`,
`${variable:?ignored}`,
that would be valid inside shell scripts, are not supported. The
variable names must consist solely of alphanumeric or underscore
ASCII characters, not start with a digit and be nonempty; otherwise such
a variable reference is ignored.

### 15.3.4 Python Format Strings

Python format strings are described in
Python Library reference /
2. Built-in Types, Exceptions and Functions /
2.2. Built-in Types /
2.2.6. Sequence Types /
2.2.6.2. String Formatting Operations.
http://www.python.org/doc/2.2.1/lib/typesseq-strings.html.

### 15.3.5 Lisp Format Strings

Lisp format strings are described in the Common Lisp HyperSpec,
chapter 22.3 Formatted Output,
http://www.lisp.org/HyperSpec/Body/sec\_22-3.html.

### 15.3.6 Emacs Lisp Format Strings

Emacs Lisp format strings are documented in the Emacs Lisp reference,
section Formatting Strings,
http://www.gnu.org/manual/elisp-manual-21-2.8/html\_chapter/elisp\_4.html#SEC75.
Note that as of version 21, XEmacs supports numbered argument specifications
in format strings while FSF Emacs doesn't.

### 15.3.7 librep Format Strings

librep format strings are documented in the librep manual, section
Formatted Output,
http://librep.sourceforge.net/librep-manual.html#Formatted%20Output,
http://www.gwinnup.org/research/docs/librep.html#SEC122.

### 15.3.8 Scheme Format Strings

Scheme format strings are documented in the SLIB manual, section
Format Specification.

### 15.3.9 Smalltalk Format Strings

Smalltalk format strings are described in the GNU Smalltalk documentation,
class `CharArray`, methods `‘bindWith:’` and
`‘bindWithArguments:’`.
http://www.gnu.org/software/smalltalk/gst-manual/gst\_68.html#SEC238.
In summary, a directive starts with `‘%’` and is followed by `‘%’`
or a nonzero digit (`‘1’` to `‘9’`).

### 15.3.10 Java Format Strings

Java format strings are described in the JDK documentation for class
`java.text.MessageFormat`,
http://java.sun.com/j2se/1.4/docs/api/java/text/MessageFormat.html.
See also the ICU documentation
http://oss.software.ibm.com/icu/apiref/classMessageFormat.html.

### 15.3.11 C# Format Strings

C# format strings are described in the .NET documentation for class
`System.String` and in
http://msdn.microsoft.com/library/default.asp?url=/library/en-us/cpguide/html/cpConFormattingOverview.asp.

### 15.3.12 awk Format Strings

awk format strings are described in the gawk documentation, section
Printf,
http://www.gnu.org/manual/gawk/html\_node/Printf.html#Printf.

### 15.3.13 Object Pascal Format Strings

Object Pascal format strings are described in the documentation of the
Free Pascal runtime library, section Format,
http://www.freepascal.org/docs-html/rtl/sysutils/format.html.

### 15.3.14 YCP Format Strings

YCP sformat strings are described in the libycp documentation
file:/usr/share/doc/packages/libycp/YCP-builtins.html.
In summary, a directive starts with `‘%’` and is followed by `‘%’`
or a nonzero digit (`‘1’` to `‘9’`).

### 15.3.15 Tcl Format Strings

Tcl format strings are described in the ‘format.n’ manual page,
http://www.scriptics.com/man/tcl8.3/TclCmd/format.htm.

### 15.3.16 Perl Format Strings

There are two kinds format strings in Perl: those acceptable to the
Perl built-in function `printf`, labelled as `‘perl-format’`,
and those acceptable to the `libintl-perl` function `__x`,
labelled as `‘perl-brace-format’`.

Perl `printf` format strings are described in the `sprintf`
section of `‘man perlfunc’`.

Perl brace format strings are described in the
‘Locale::TextDomain(3pm)’ manual page of the CPAN package
libintl-perl. In brief, Perl format uses placeholders put between
braces (`‘{’` and `‘}’`). The placeholder must have the syntax
of simple identifiers.

### 15.3.17 PHP Format Strings

PHP format strings are described in the documentation of the PHP function
`sprintf`, in ‘phpdoc/manual/function.sprintf.html’ or
http://www.php.net/manual/en/function.sprintf.php.

### 15.3.18 GCC internal Format Strings

These format strings are used inside the GCC sources. In such a format
string, a directive starts with `‘%’`, is optionally followed by a
size specifier `‘l’`, an optional flag `‘+’`, another optional flag
`‘#’`, and is finished by a specifier: `‘%’` denotes a literal
percent sign, `‘c’` denotes a character, `‘s’` denotes a string,
`‘i’` and `‘d’` denote an integer, `‘o’`, `‘u’`, `‘x’`
denote an unsigned integer, `‘.*s’` denotes a string preceded by a
width specification, `‘H’` denotes a `‘location_t *’` pointer,
`‘D’` denotes a general declaration, `‘F’` denotes a function
declaration, `‘T’` denotes a type, `‘A’` denotes a function argument,
`‘C’` denotes a tree code, `‘E’` denotes an expression, `‘L’`
denotes a programming language, `‘O’` denotes a binary operator,
`‘P’` denotes a function parameter, `‘Q’` denotes an assignment
operator, `‘V’` denotes a const/volatile qualifier.

### 15.3.19 GFC internal Format Strings

These format strings are used inside the GNU Fortran Compiler sources,
that is, the Fortran frontend in the GCC sources. In such a format
string, a directive starts with `‘%’` and is finished by a
specifier: `‘%’` denotes a literal percent sign, `‘C’` denotes the
current source location, `‘L’` denotes a source location, `‘c’`
denotes a character, `‘s’` denotes a string, `‘i’` and `‘d’`
denote an integer, `‘u’` denotes an unsigned integer. `‘i’`,
`‘d’`, and `‘u’` may be preceded by a size specifier `‘l’`.

### 15.3.20 Qt Format Strings

Qt format strings are described in the documentation of the QString class
file:/usr/lib/qt-4.3.0/doc/html/qstring.html.
In summary, a directive consists of a `‘%’` followed by a digit. The same
directive cannot occur more than once in a format string.

### 15.3.21 Qt Format Strings

Qt format strings are described in the documentation of the QObject::tr method
file:/usr/lib/qt-4.3.0/doc/html/qobject.html.
In summary, the only allowed directive is `‘%n’`.

### 15.3.22 KDE Format Strings

KDE 4 format strings are defined as follows:
A directive consists of a `‘%’` followed by a non-zero decimal number.
If a `‘%n’` occurs in a format strings, all of `‘%1’`, ..., `‘%(n-1)’`
must occur as well, except possibly one of them.

### 15.3.23 Boost Format Strings

Boost format strings are described in the documentation of the
`boost::format` class, at
http://www.boost.org/libs/format/doc/format.html.
In summary, a directive has either the same syntax as in a C format string,
such as `‘%1$+5d’`, or may be surrounded by vertical bars, such as
`‘%|1$+5d|’` or `‘%|1$+5|’`, or consists of just an argument number
between percent signs, such as `‘%1%’`.

## 15.4 The Maintainer's View

For the maintainer, the general procedure differs from the C language
case in two ways.

- For those languages that don't use GNU gettext, the ‘intl/’ directory
  is not needed and can be omitted. This means that the maintainer calls the
  `gettextize` program without the `‘--intl’` option, and that he
  invokes the `AM_GNU_GETTEXT` autoconf macro via
  `‘AM_GNU_GETTEXT([external])’`.- If only a single programming language is used, the `XGETTEXT_OPTIONS`
    variable in ‘po/Makevars’ (see section 13.4.3 ‘Makevars’ in ‘po/’) should be adjusted to
    match the `xgettext` options for that particular programming language.
    If the package uses more than one programming language with `gettext`
    support, it becomes necessary to change the POT file construction rule
    in ‘po/Makefile.in.in’. It is recommended to make one `xgettext`
    invocation per programming language, each with the options appropriate for
    that language, and to combine the resulting files using `msgcat`.

## 15.5 Individual Programming Languages

### 15.5.1 C, C++, Objective C

RPMs: gcc, gpp, gobjc, glibc, gettext File extension: For C: `c`, `h`. For C++: `C`, `c++`, `cc`, `cxx`, `cpp`, `hpp`. For Objective C: `m`. String syntax: `"abc"` gettext shorthand: `_("abc")` gettext/ngettext functions: `gettext`, `dgettext`, `dcgettext`, `ngettext`, `dngettext`, `dcngettext` textdomain: `textdomain` function bindtextdomain: `bindtextdomain` function setlocale: Programmer must call `setlocale (LC_ALL, "")` Prerequisite: `#include <libintl.h>` `#include <locale.h>` `#define _(string) gettext (string)` Use or emulate GNU gettext: Use Extractor: `xgettext -k_` Formatting with positions: `fprintf "%2$d %1$d"` In C++: `autosprintf "%2$d %1$d"` (see section ‘Introduction’ in GNU autosprintf) Portability: autoconf (gettext.m4) and #if ENABLE\_NLS po-mode marking: yes

The following examples are available in the ‘examples’ directory:
`hello-c`, `hello-c-gnome`, `hello-c++`, `hello-c++-qt`,
`hello-c++-kde`, `hello-c++-gnome`, `hello-c++-wxwidgets`,
`hello-objc`, `hello-objc-gnustep`, `hello-objc-gnome`.

### 15.5.2 sh - Shell Script

RPMs: bash, gettext File extension: `sh` String syntax: `"abc"`, `'abc'`, `abc` gettext shorthand: `` "`gettext \"abc\"`" `` gettext/ngettext functions: `gettext`, `ngettext` programs `eval_gettext`, `eval_ngettext` shell functions textdomain: environment variable `TEXTDOMAIN` bindtextdomain: environment variable `TEXTDOMAINDIR` setlocale: automatic Prerequisite: `. gettext.sh` Use or emulate GNU gettext: use Extractor: `xgettext` Formatting with positions: --- Portability: fully portable po-mode marking: ---

An example is available in the ‘examples’ directory: `hello-sh`.

#### 15.5.2.1 Preparing Shell Scripts for Internationalization

Preparing a shell script for internationalization is conceptually similar
to the steps described in section 4 Preparing Program Sources. The concrete steps for shell
scripts are as follows.

1. Insert the line

   ```
   . gettext.sh
   ```

   near the top of the script. `gettext.sh` is a shell function library
   that provides the functions
   `eval_gettext` (see section 15.5.2.6 Invoking the `eval_gettext` function) and
   `eval_ngettext` (see section 15.5.2.7 Invoking the `eval_ngettext` function).
   You have to ensure that `gettext.sh` can be found in the `PATH`.- Set and export the `TEXTDOMAIN` and `TEXTDOMAINDIR` environment
     variables. Usually `TEXTDOMAIN` is the package or program name, and
     `TEXTDOMAINDIR` is the absolute pathname corresponding to
     `$prefix/share/locale`, where `$prefix` is the installation location.

     ```
     TEXTDOMAIN=@PACKAGE@
     export TEXTDOMAIN
     TEXTDOMAINDIR=@LOCALEDIR@
     export TEXTDOMAINDIR
     ```

     - Prepare the strings for translation, as described in section 4.3 Preparing Translatable Strings.- Simplify translatable strings so that they don't contain command substitution
         (`` "`...`" `` or `"$(...)"`), variable access with defaulting (like
         `${variable-default}`), access to positional arguments
         (like `$0`, `$1`, ...) or highly volatile shell variables (like
         `$?`). This can always be done through simple local code restructuring.
         For example,

         ```
         echo "Usage: $0 [OPTION] FILE..."
         ```

         becomes

         ```
         program_name=$0
         echo "Usage: $program_name [OPTION] FILE..."
         ```

         Similarly,

         ```
         echo "Remaining files: `ls | wc -l`"
         ```

         becomes

         ```
         filecount="`ls | wc -l`"
         echo "Remaining files: $filecount"
         ```

         - For each translatable string, change the output command `‘echo’` or
           `‘$echo’` to `‘gettext’` (if the string contains no references to
           shell variables) or to `‘eval_gettext’` (if it refers to shell variables),
           followed by a no-argument `‘echo’` command (to account for the terminating
           newline). Similarly, for cases with plural handling, replace a conditional
           `‘echo’` command with an invocation of `‘ngettext’` or
           `‘eval_ngettext’`, followed by a no-argument `‘echo’` command.
           When doing this, you also need to add an extra backslash before the dollar
           sign in references to shell variables, so that the `‘eval_gettext’`
           function receives the translatable string before the variable values are
           substituted into it. For example,

           ```
           echo "Remaining files: $filecount"
           ```

           becomes

           ```
           eval_gettext "Remaining files: \$filecount"; echo
           ```

           If the output command is not `‘echo’`, you can make it use `‘echo’`
           nevertheless, through the use of backquotes. However, note that inside
           backquotes, backslashes must be doubled to be effective (because the
           backquoting eats one level of backslashes). For example, assuming that
           `‘error’` is a shell function that signals an error,

           ```
           error "file not found: $filename"
           ```

           is first transformed into

           ```
           error "`echo \"file not found: \$filename\"`"
           ```

           which then becomes

           ```
           error "`eval_gettext \"file not found: \\\$filename\"`"
           ```

#### 15.5.2.2 Contents of `gettext.sh`

`gettext.sh`, contained in the run-time package of GNU gettext, provides
the following:

- $echo
  The variable `echo` is set to a command that outputs its first argument
  and a newline, without interpreting backslashes in the argument string.- eval\_gettext
    See section 15.5.2.6 Invoking the `eval_gettext` function.- eval\_ngettext
      See section 15.5.2.7 Invoking the `eval_ngettext` function.

#### 15.5.2.3 Invoking the `gettext` program

```
gettext [option] [[textdomain] msgid]
gettext [option] -s [msgid]...
```

The `gettext` program displays the native language translation of a
textual message.

**Arguments**

`‘-d textdomain’`: `‘--domain=textdomain’`: Retrieve translated messages from textdomain. Usually a textdomain corresponds to a package, a program, or a module of a program. `‘-e’`: Enable expansion of some escape sequences. This option is for compatibility with the `‘echo’` program or shell built-in. The escape sequences `‘\a’`, `‘\b’`, `‘\c’`, `‘\f’`, `‘\n’`, `‘\r’`, `‘\t’`, `‘\v’`, `‘\\’`, and `‘\’` followed by one to three octal digits, are interpreted like the System V `‘echo’` program did. `‘-E’`: This option is only for compatibility with the `‘echo’` program or shell built-in. It has no effect. `‘-h’`: `‘--help’`: Display this help and exit. `‘-n’`: Suppress trailing newline. By default, `gettext` adds a newline to the output. `‘-V’`: `‘--version’`: Output version information and exit. `‘[textdomain] msgid’`: Retrieve translated message corresponding to msgid from textdomain.

If the textdomain parameter is not given, the domain is determined from
the environment variable `TEXTDOMAIN`. If the message catalog is not
found in the regular directory, another location can be specified with the
environment variable `TEXTDOMAINDIR`.

When used with the `-s` option the program behaves like the `‘echo’`
command. But it does not simply copy its arguments to stdout. Instead those
messages found in the selected catalog are translated.

Note: `xgettext` supports only the one-argument form of the
`gettext` invocation, where no options are present and the
textdomain is implicit, from the environment.

#### 15.5.2.4 Invoking the `ngettext` program

```
ngettext [option] [textdomain] msgid msgid-plural count
```

The `ngettext` program displays the native language translation of a
textual message whose grammatical form depends on a number.

**Arguments**

`‘-d textdomain’`: `‘--domain=textdomain’`: Retrieve translated messages from textdomain. Usually a textdomain corresponds to a package, a program, or a module of a program. `‘-e’`: Enable expansion of some escape sequences. This option is for compatibility with the `‘gettext’` program. The escape sequences `‘\a’`, `‘\b’`, `‘\c’`, `‘\f’`, `‘\n’`, `‘\r’`, `‘\t’`, `‘\v’`, `‘\\’`, and `‘\’` followed by one to three octal digits, are interpreted like the System V `‘echo’` program did. `‘-E’`: This option is only for compatibility with the `‘gettext’` program. It has no effect. `‘-h’`: `‘--help’`: Display this help and exit. `‘-V’`: `‘--version’`: Output version information and exit. `‘textdomain’`: Retrieve translated message from textdomain. `‘msgid msgid-plural’`: Translate msgid (English singular) / msgid-plural (English plural). `‘count’`: Choose singular/plural form based on this value.

If the textdomain parameter is not given, the domain is determined from
the environment variable `TEXTDOMAIN`. If the message catalog is not
found in the regular directory, another location can be specified with the
environment variable `TEXTDOMAINDIR`.

Note: `xgettext` supports only the three-arguments form of the
`ngettext` invocation, where no options are present and the
textdomain is implicit, from the environment.

#### 15.5.2.5 Invoking the `envsubst` program

```
envsubst [option] [shell-format]
```

The `envsubst` program substitutes the values of environment variables.

**Operation mode**

`‘-v’`: `‘--variables’`: Output the variables occurring in shell-format.

**Informative output**

`‘-h’`: `‘--help’`: Display this help and exit. `‘-V’`: `‘--version’`: Output version information and exit.

In normal operation mode, standard input is copied to standard output,
with references to environment variables of the form `$VARIABLE` or
`${VARIABLE}` being replaced with the corresponding values. If a
shell-format is given, only those environment variables that are
referenced in shell-format are substituted; otherwise all environment
variables references occurring in standard input are substituted.

These substitutions are a subset of the substitutions that a shell performs
on unquoted and double-quoted strings. Other kinds of substitutions done
by a shell, such as `${variable-default}` or
`$(command-list)` or `` `command-list` ``, are not performed
by the `envsubst` program, due to security reasons.

When `--variables` is used, standard input is ignored, and the output
consists of the environment variables that are referenced in
shell-format, one per line.

#### 15.5.2.6 Invoking the `eval_gettext` function

```
eval_gettext msgid
```

This function outputs the native language translation of a textual message,
performing dollar-substitution on the result. Note that only shell variables
mentioned in msgid will be dollar-substituted in the result.

#### 15.5.2.7 Invoking the `eval_ngettext` function

```
eval_ngettext msgid msgid-plural count
```

This function outputs the native language translation of a textual message
whose grammatical form depends on a number, performing dollar-substitution
on the result. Note that only shell variables mentioned in msgid or
msgid-plural will be dollar-substituted in the result.

### 15.5.3 bash - Bourne-Again Shell Script

GNU `bash` 2.0 or newer has a special shorthand for translating a
string and substituting variable values in it: `$"msgid"`. But
the use of this construct is **discouraged**, due to the security
holes it opens and due to its portability problems.

The security holes of `$"..."` come from the fact that after looking up
the translation of the string, `bash` processes it like it processes
any double-quoted string: dollar and backquote processing, like `‘eval’`
does.

1. In a locale whose encoding is one of BIG5, BIG5-HKSCS, GBK, GB18030, SHIFT\_JIS,
   JOHAB, some double-byte characters have a second byte whose value is
   `0x60`. For example, the byte sequence `\xe0\x60` is a single
   character in these locales. Many versions of `bash` (all versions
   up to bash-2.05, and newer versions on platforms without `mbsrtowcs()`
   function) don't know about character boundaries and see a backquote character
   where there is only a particular Chinese character. Thus it can start
   executing part of the translation as a command list. This situation can occur
   even without the translator being aware of it: if the translator provides
   translations in the UTF-8 encoding, it is the `gettext()` function which
   will, during its conversion from the translator's encoding to the user's
   locale's encoding, produce the dangerous `\x60` bytes.- A translator could - voluntarily or inadvertently - use backquotes
     `` "`...`" `` or dollar-parentheses `"$(...)"` in her translations.
     The enclosed strings would be executed as command lists by the shell.

The portability problem is that `bash` must be built with
internationalization support; this is normally not the case on systems
that don't have the `gettext()` function in libc.

### 15.5.4 Python

RPMs: python File extension: `py` String syntax: `'abc'`, `u'abc'`, `r'abc'`, `ur'abc'`, `"abc"`, `u"abc"`, `r"abc"`, `ur"abc"`, `”'abc”'`, `u”'abc”'`, `r”'abc”'`, `ur”'abc”'`, `"""abc"""`, `u"""abc"""`, `r"""abc"""`, `ur"""abc"""` gettext shorthand: `_('abc')` etc. gettext/ngettext functions: `gettext.gettext`, `gettext.dgettext`, `gettext.ngettext`, `gettext.dngettext`, also `ugettext`, `ungettext` textdomain: `gettext.textdomain` function, or `gettext.install(domain)` function bindtextdomain: `gettext.bindtextdomain` function, or `gettext.install(domain,localedir)` function setlocale: not used by the gettext emulation Prerequisite: `import gettext` Use or emulate GNU gettext: emulate Extractor: `xgettext` Formatting with positions: `'...%(ident)d...' % { 'ident': value }` Portability: fully portable po-mode marking: ---

An example is available in the ‘examples’ directory: `hello-python`.

A note about format strings: Python supports format strings with unnamed
arguments, such as `'...%d...'`, and format strings with named arguments,
such as `'...%(ident)d...'`. The latter are preferable for
internationalized programs, for two reasons:

- When a format string takes more than one argument, the translator can provide
  a translation that uses the arguments in a different order, if the format
  string uses named arguments. For example, the translator can reformulate

  ```
  "'%(volume)s' has only %(freespace)d bytes free."
  ```

  to

  ```
  "Only %(freespace)d bytes free on '%(volume)s'."
  ```

  Additionally, the identifiers also provide some context to the translator.- In the context of plural forms, the format string used for the singular form
    does not use the numeric argument in many languages. Even in English, one
    prefers to write `"one hour"` instead of `"1 hour"`. Omitting
    individual arguments from format strings like this is only possible with
    the named argument syntax. (With unnamed arguments, Python -- unlike C --
    verifies that the format string uses all supplied arguments.)

### 15.5.5 GNU clisp - Common Lisp

RPMs: clisp 2.28 or newer File extension: `lisp` String syntax: `"abc"` gettext shorthand: `(_ "abc")`, `(ENGLISH "abc")` gettext/ngettext functions: `i18n:gettext`, `i18n:ngettext` textdomain: `i18n:textdomain` bindtextdomain: `i18n:textdomaindir` setlocale: automatic Prerequisite: --- Use or emulate GNU gettext: use Extractor: `xgettext -k_ -kENGLISH` Formatting with positions: `format "~1@*~D ~0@*~D"` Portability: On platforms without gettext, no translation. po-mode marking: ---

An example is available in the ‘examples’ directory: `hello-clisp`.

### 15.5.6 GNU clisp C sources

RPMs: clisp File extension: `d` String syntax: `"abc"` gettext shorthand: `ENGLISH ? "abc" : ""` `GETTEXT("abc")` `GETTEXTL("abc")` gettext/ngettext functions: `clgettext`, `clgettextl` textdomain: --- bindtextdomain: --- setlocale: automatic Prerequisite: `#include "lispbibl.c"` Use or emulate GNU gettext: use Extractor: `clisp-xgettext` Formatting with positions: `fprintf "%2$d %1$d"` Portability: On platforms without gettext, no translation. po-mode marking: ---

### 15.5.7 Emacs Lisp

RPMs: emacs, xemacs File extension: `el` String syntax: `"abc"` gettext shorthand: `(_"abc")` gettext/ngettext functions: `gettext`, `dgettext` (xemacs only) textdomain: `domain` special form (xemacs only) bindtextdomain: `bind-text-domain` function (xemacs only) setlocale: automatic Prerequisite: --- Use or emulate GNU gettext: use Extractor: `xgettext` Formatting with positions: `format "%2$d %1$d"` Portability: Only XEmacs. Without `I18N3` defined at build time, no translation. po-mode marking: ---

### 15.5.8 librep

RPMs: librep 0.15.3 or newer File extension: `jl` String syntax: `"abc"` gettext shorthand: `(_"abc")` gettext/ngettext functions: `gettext` textdomain: `textdomain` function bindtextdomain: `bindtextdomain` function setlocale: --- Prerequisite: `(require 'rep.i18n.gettext)` Use or emulate GNU gettext: use Extractor: `xgettext` Formatting with positions: `format "%2$d %1$d"` Portability: On platforms without gettext, no translation. po-mode marking: ---

An example is available in the ‘examples’ directory: `hello-librep`.

### 15.5.9 GNU guile - Scheme

RPMs: guile File extension: `scm` String syntax: `"abc"` gettext shorthand: `(_ "abc")` gettext/ngettext functions: `gettext`, `ngettext` textdomain: `textdomain` bindtextdomain: `bindtextdomain` setlocale: `(catch #t (lambda () (setlocale LC_ALL "")) (lambda args #f))` Prerequisite: `(use-modules (ice-9 format))` Use or emulate GNU gettext: use Extractor: `xgettext -k_` Formatting with positions: --- Portability: On platforms without gettext, no translation. po-mode marking: ---

An example is available in the ‘examples’ directory: `hello-guile`.

### 15.5.10 GNU Smalltalk

RPMs: smalltalk File extension: `st` String syntax: `'abc'` gettext shorthand: `NLS ? 'abc'` gettext/ngettext functions: `LcMessagesDomain>>#at:`, `LcMessagesDomain>>#at:plural:with:` textdomain: `LcMessages>>#domain:localeDirectory:` (returns a `LcMessagesDomain` object). Example: `I18N Locale default messages domain: 'gettext' localeDirectory: /usr/local/share/locale'` bindtextdomain: `LcMessages>>#domain:localeDirectory:`, see above. setlocale: Automatic if you use `I18N Locale default`. Prerequisite: `PackageLoader fileInPackage: 'I18N'!` Use or emulate GNU gettext: emulate Extractor: `xgettext` Formatting with positions: `'%1 %2' bindWith: 'Hello' with: 'world'` Portability: fully portable po-mode marking: ---

An example is available in the ‘examples’ directory:
`hello-smalltalk`.

### 15.5.11 Java

RPMs: java, java2 File extension: `java` String syntax: "abc" gettext shorthand: \_("abc") gettext/ngettext functions: `GettextResource.gettext`, `GettextResource.ngettext`, `GettextResource.pgettext`, `GettextResource.npgettext` textdomain: ---, use `ResourceBundle.getResource` instead bindtextdomain: ---, use CLASSPATH instead setlocale: automatic Prerequisite: --- Use or emulate GNU gettext: ---, uses a Java specific message catalog format Extractor: `xgettext -k_` Formatting with positions: `MessageFormat.format "{1,number} {0,number}"` Portability: fully portable po-mode marking: ---

Before marking strings as internationalizable, uses of the string
concatenation operator need to be converted to `MessageFormat`
applications. For example, `"file "+filename+" not found"` becomes
`MessageFormat.format("file {0} not found", new Object[] { filename })`.
Only after this is done, can the strings be marked and extracted.

GNU gettext uses the native Java internationalization mechanism, namely
`ResourceBundle`s. There are two formats of `ResourceBundle`s:
`.properties` files and `.class` files. The `.properties`
format is a text file which the translators can directly edit, like PO
files, but which doesn't support plural forms. Whereas the `.class`
format is compiled from `.java` source code and can support plural
forms (provided it is accessed through an appropriate API, see below).

To convert a PO file to a `.properties` file, the `msgcat`
program can be used with the option `--properties-output`. To convert
a `.properties` file back to a PO file, the `msgcat` program
can be used with the option `--properties-input`. All the tools
that manipulate PO files can work with `.properties` files as well,
if given the `--properties-input` and/or `--properties-output`
option.

To convert a PO file to a ResourceBundle class, the `msgfmt` program
can be used with the option `--java` or `--java2`. To convert a
ResourceBundle back to a PO file, the `msgunfmt` program can be used
with the option `--java`.

Two different programmatic APIs can be used to access ResourceBundles.
Note that both APIs work with all kinds of ResourceBundles, whether
GNU gettext generated classes, or other `.class` or `.properties`
files.

1. The `java.util.ResourceBundle` API.
   In particular, its `getString` function returns a string translation.
   Note that a missing translation yields a `MissingResourceException`.
   This has the advantage of being the standard API. And it does not require
   any additional libraries, only the `msgcat` generated `.properties`
   files or the `msgfmt` generated `.class` files. But it cannot do
   plural handling, even if the resource was generated by `msgfmt` from
   a PO file with plural handling.- The `gnu.gettext.GettextResource` API.
     Reference documentation in Javadoc 1.1 style format is in the
     javadoc2 directory.
     Its `gettext` function returns a string translation. Note that when
     a translation is missing, the msgid argument is returned unchanged.
     This has the advantage of having the `ngettext` function for plural
     handling and the `pgettext` and `npgettext` for strings constraint
     to a particular context.
     To use this API, one needs the `libintl.jar` file which is part of
     the GNU gettext package and distributed under the LGPL.

Four examples, using the second API, are available in the ‘examples’
directory: `hello-java`, `hello-java-awt`, `hello-java-swing`,
`hello-java-qtjambi`.

Now, to make use of the API and define a shorthand for `‘getString’`,
there are three idioms that you can choose from:

- (This one assumes Java 1.5 or newer.)
  In a unique class of your project, say `‘Util’`, define a static variable
  holding the `ResourceBundle` instance and the shorthand:

  ```
  private static ResourceBundle myResources =
    ResourceBundle.getBundle("domain-name");
  public static String _(String s) {
    return myResources.getString(s);
  }
  ```

  All classes containing internationalized strings then contain

  ```
  import static Util._;
  ```

  and the shorthand is used like this:

  ```
  System.out.println(_("Operation completed."));
  ```

  - In a unique class of your project, say `‘Util’`, define a static variable
    holding the `ResourceBundle` instance:

    ```
    public static ResourceBundle myResources =
      ResourceBundle.getBundle("domain-name");
    ```

    All classes containing internationalized strings then contain

    ```
    private static ResourceBundle res = Util.myResources;
    private static String _(String s) { return res.getString(s); }
    ```

    and the shorthand is used like this:

    ```
    System.out.println(_("Operation completed."));
    ```

    - You add a class with a very short name, say `‘S’`, containing just the
      definition of the resource bundle and of the shorthand:

      ```
      public class S {
        public static ResourceBundle myResources =
          ResourceBundle.getBundle("domain-name");
        public static String _(String s) {
          return myResources.getString(s);
        }
      }
      ```

      and the shorthand is used like this:

      ```
      System.out.println(S._("Operation completed."));
      ```

Which of the three idioms you choose, will depend on whether your project
requires portability to Java versions prior to Java 1.5 and, if so, whether
copying two lines of codes into every class is more acceptable in your project
than a class with a single-letter name.

### 15.5.12 C#

RPMs: pnet, pnetlib 0.6.2 or newer, or mono 0.29 or newer File extension: `cs` String syntax: `"abc"`, `@"abc"` gettext shorthand: \_("abc") gettext/ngettext functions: `GettextResourceManager.GetString`, `GettextResourceManager.GetPluralString` `GettextResourceManager.GetParticularString` `GettextResourceManager.GetParticularPluralString` textdomain: `new GettextResourceManager(domain)` bindtextdomain: ---, compiled message catalogs are located in subdirectories of the directory containing the executable setlocale: automatic Prerequisite: --- Use or emulate GNU gettext: ---, uses a C# specific message catalog format Extractor: `xgettext -k_` Formatting with positions: `String.Format "{1} {0}"` Portability: fully portable po-mode marking: ---

Before marking strings as internationalizable, uses of the string
concatenation operator need to be converted to `String.Format`
invocations. For example, `"file "+filename+" not found"` becomes
`String.Format("file {0} not found", filename)`.
Only after this is done, can the strings be marked and extracted.

GNU gettext uses the native C#/.NET internationalization mechanism, namely
the classes `ResourceManager` and `ResourceSet`. Applications
use the `ResourceManager` methods to retrieve the native language
translation of strings. An instance of `ResourceSet` is the in-memory
representation of a message catalog file. The `ResourceManager` loads
and accesses `ResourceSet` instances as needed to look up the
translations.

There are two formats of `ResourceSet`s that can be directly loaded by
the C# runtime: `.resources` files and `.dll` files.

- The `.resources` format is a binary file usually generated through the
  `resgen` or `monoresgen` utility, but which doesn't support plural
  forms. `.resources` files can also be embedded in .NET `.exe` files.
  This only affects whether a file system access is performed to load the message
  catalog; it doesn't affect the contents of the message catalog.- On the other hand, the `.dll` format is a binary file that is compiled
    from `.cs` source code and can support plural forms (provided it is
    accessed through the GNU gettext API, see below).

Note that these .NET `.dll` and `.exe` files are not tied to a
particular platform; their file format and GNU gettext for C# can be used
on any platform.

To convert a PO file to a `.resources` file, the `msgfmt` program
can be used with the option `‘--csharp-resources’`. To convert a
`.resources` file back to a PO file, the `msgunfmt` program can be
used with the option `‘--csharp-resources’`. You can also, in some cases,
use the `resgen` program (from the `pnet` package) or the
`monoresgen` program (from the `mono`/`mcs` package). These
programs can also convert a `.resources` file back to a PO file. But
beware: as of this writing (January 2004), the `monoresgen` converter is
quite buggy and the `resgen` converter ignores the encoding of the PO
files.

To convert a PO file to a `.dll` file, the `msgfmt` program can be
used with the option `--csharp`. The result will be a `.dll` file
containing a subclass of `GettextResourceSet`, which itself is a subclass
of `ResourceSet`. To convert a `.dll` file containing a
`GettextResourceSet` subclass back to a PO file, the `msgunfmt`
program can be used with the option `--csharp`.

The advantages of the `.dll` format over the `.resources` format
are:

1. Freedom to localize: Users can add their own translations to an application
   after it has been built and distributed. Whereas when the programmer uses
   a `ResourceManager` constructor provided by the system, the set of
   `.resources` files for an application must be specified when the
   application is built and cannot be extended afterwards.- Plural handling: A message catalog in `.dll` format supports the plural
     handling function `GetPluralString`. Whereas `.resources` files can
     only contain data and only support lookups that depend on a single string.- Context handling: A message catalog in `.dll` format supports the
       query-with-context functions `GetParticularString` and
       `GetParticularPluralString`. Whereas `.resources` files can
       only contain data and only support lookups that depend on a single string.- The `GettextResourceManager` that loads the message catalogs in
         `.dll` format also provides for inheritance on a per-message basis.
         For example, in Austrian (`de_AT`) locale, translations from the German
         (`de`) message catalog will be used for messages not found in the
         Austrian message catalog. This has the consequence that the Austrian
         translators need only translate those few messages for which the translation
         into Austrian differs from the German one. Whereas when working with
         `.resources` files, each message catalog must provide the translations
         of all messages by itself.- The `GettextResourceManager` that loads the message catalogs in
           `.dll` format also provides for a fallback: The English msgid is
           returned when no translation can be found. Whereas when working with
           `.resources` files, a language-neutral `.resources` file must
           explicitly be provided as a fallback.

On the side of the programmatic APIs, the programmer can use either the
standard `ResourceManager` API and the GNU `GettextResourceManager`
API. The latter is an extension of the former, because
`GettextResourceManager` is a subclass of `ResourceManager`.

1. The `System.Resources.ResourceManager` API.
   This API works with resources in `.resources` format.
   The creation of the `ResourceManager` is done through

   ```
     new ResourceManager(domainname, Assembly.GetExecutingAssembly())
   ```

   The `GetString` function returns a string's translation. Note that this
   function returns null when a translation is missing (i.e. not even found in
   the fallback resource file).- The `GNU.Gettext.GettextResourceManager` API.
     This API works with resources in `.dll` format.
     Reference documentation is in the
     csharpdoc directory.
     The creation of the `ResourceManager` is done through

     ```
       new GettextResourceManager(domainname)
     ```

     The `GetString` function returns a string's translation. Note that when
     a translation is missing, the msgid argument is returned unchanged.
     The `GetPluralString` function returns a string translation with plural
     handling, like the `ngettext` function in C.
     The `GetParticularString` function returns a string's translation,
     specific to a particular context, like the `pgettext` function in C.
     Note that when a translation is missing, the msgid argument is returned
     unchanged.
     The `GetParticularPluralString` function returns a string translation,
     specific to a particular context, with plural handling, like the
     `npgettext` function in C.
     To use this API, one needs the `GNU.Gettext.dll` file which is part of
     the GNU gettext package and distributed under the LGPL.

You can also mix both approaches: use the
`GNU.Gettext.GettextResourceManager` constructor, but otherwise use
only the `ResourceManager` type and only the `GetString` method.
This is appropriate when you want to profit from the tools for PO files,
but don't want to change an existing source code that uses
`ResourceManager` and don't (yet) need the `GetPluralString` method.

Two examples, using the second API, are available in the ‘examples’
directory: `hello-csharp`, `hello-csharp-forms`.

Now, to make use of the API and define a shorthand for `‘GetString’`,
there are two idioms that you can choose from:

- In a unique class of your project, say `‘Util’`, define a static variable
  holding the `ResourceManager` instance:

  ```
  public static GettextResourceManager MyResourceManager =
    new GettextResourceManager("domain-name");
  ```

  All classes containing internationalized strings then contain

  ```
  private static GettextResourceManager Res = Util.MyResourceManager;
  private static String _(String s) { return Res.GetString(s); }
  ```

  and the shorthand is used like this:

  ```
  Console.WriteLine(_("Operation completed."));
  ```

  - You add a class with a very short name, say `‘S’`, containing just the
    definition of the resource manager and of the shorthand:

    ```
    public class S {
      public static GettextResourceManager MyResourceManager =
        new GettextResourceManager("domain-name");
      public static String _(String s) {
         return MyResourceManager.GetString(s);
      }
    }
    ```

    and the shorthand is used like this:

    ```
    Console.WriteLine(S._("Operation completed."));
    ```

Which of the two idioms you choose, will depend on whether copying two lines
of codes into every class is more acceptable in your project than a class
with a single-letter name.

### 15.5.13 GNU awk

RPMs: gawk 3.1 or newer File extension: `awk` String syntax: `"abc"` gettext shorthand: `_"abc"` gettext/ngettext functions: `dcgettext`, missing `dcngettext` in gawk-3.1.0 textdomain: `TEXTDOMAIN` variable bindtextdomain: `bindtextdomain` function setlocale: automatic, but missing `setlocale (LC_MESSAGES, "")` in gawk-3.1.0 Prerequisite: --- Use or emulate GNU gettext: use Extractor: `xgettext` Formatting with positions: `printf "%2$d %1$d"` (GNU awk only) Portability: On platforms without gettext, no translation. On non-GNU awks, you must define `dcgettext`, `dcngettext` and `bindtextdomain` yourself. po-mode marking: ---

An example is available in the ‘examples’ directory: `hello-gawk`.

### 15.5.14 Pascal - Free Pascal Compiler

RPMs: fpk File extension: `pp`, `pas` String syntax: `'abc'` gettext shorthand: automatic gettext/ngettext functions: ---, use `ResourceString` data type instead textdomain: ---, use `TranslateResourceStrings` function instead bindtextdomain: ---, use `TranslateResourceStrings` function instead setlocale: automatic, but uses only LANG, not LC\_MESSAGES or LC\_ALL Prerequisite: `{$mode delphi}` or `{$mode objfpc}` `uses gettext;` Use or emulate GNU gettext: emulate partially Extractor: `ppc386` followed by `xgettext` or `rstconv` Formatting with positions: `uses sysutils;` `format "%1:d %0:d"` Portability: ? po-mode marking: ---

The Pascal compiler has special support for the `ResourceString` data
type. It generates a `.rst` file. This is then converted to a
`.pot` file by use of `xgettext` or `rstconv`. At runtime,
a `.mo` file corresponding to translations of this `.pot` file
can be loaded using the `TranslateResourceStrings` function in the
`gettext` unit.

An example is available in the ‘examples’ directory: `hello-pascal`.

### 15.5.15 wxWidgets library

RPMs: wxGTK, gettext File extension: `cpp` String syntax: `"abc"` gettext shorthand: `_("abc")` gettext/ngettext functions: `wxLocale::GetString`, `wxGetTranslation` textdomain: `wxLocale::AddCatalog` bindtextdomain: `wxLocale::AddCatalogLookupPathPrefix` setlocale: `wxLocale::Init`, `wxSetLocale` Prerequisite: `#include <wx/intl.h>` Use or emulate GNU gettext: emulate, see `include/wx/intl.h` and `src/common/intl.cpp` Extractor: `xgettext` Formatting with positions: wxString::Format supports positions if and only if the system has `wprintf()`, `vswprintf()` functions and they support positions according to POSIX. Portability: fully portable po-mode marking: yes

### 15.5.16 YCP - YaST2 scripting language

RPMs: libycp, libycp-devel, yast2-core, yast2-core-devel File extension: `ycp` String syntax: `"abc"` gettext shorthand: `_("abc")` gettext/ngettext functions: `_()` with 1 or 3 arguments textdomain: `textdomain` statement bindtextdomain: --- setlocale: --- Prerequisite: --- Use or emulate GNU gettext: use Extractor: `xgettext` Formatting with positions: `sformat "%2 %1"` Portability: fully portable po-mode marking: ---

An example is available in the ‘examples’ directory: `hello-ycp`.

### 15.5.17 Tcl - Tk's scripting language

RPMs: tcl File extension: `tcl` String syntax: `"abc"` gettext shorthand: `[_ "abc"]` gettext/ngettext functions: `::msgcat::mc` textdomain: --- bindtextdomain: ---, use `::msgcat::mcload` instead setlocale: automatic, uses LANG, but ignores LC\_MESSAGES and LC\_ALL Prerequisite: `package require msgcat` `proc _ {s} {return [::msgcat::mc $s]}` Use or emulate GNU gettext: ---, uses a Tcl specific message catalog format Extractor: `xgettext -k_` Formatting with positions: `format "%2\$d %1\$d"` Portability: fully portable po-mode marking: ---

Two examples are available in the ‘examples’ directory:
`hello-tcl`, `hello-tcl-tk`.

Before marking strings as internationalizable, substitutions of variables
into the string need to be converted to `format` applications. For
example, `"file $filename not found"` becomes
`[format "file %s not found" $filename]`.
Only after this is done, can the strings be marked and extracted.
After marking, this example becomes
`[format [_ "file %s not found"] $filename]` or
`[msgcat::mc "file %s not found" $filename]`. Note that the
`msgcat::mc` function implicitly calls `format` when more than one
argument is given.

### 15.5.18 Perl

RPMs: perl File extension: `pl`, `PL`, `pm`, `cgi` String syntax: - `"abc"`- `'abc'`- `qq (abc)`- `q (abc)`- `qr /abc/`- `qx (/bin/date)`- `/pattern match/`- `?pattern match?`- `s/substitution/operators/`- `$tied_hash{"message"}`- `$tied_hash_reference->{"message"}`- etc., issue the command `‘man perlsyn’` for details gettext shorthand: `__` (double underscore) gettext/ngettext functions: `gettext`, `dgettext`, `dcgettext`, `ngettext`, `dngettext`, `dcngettext` textdomain: `textdomain` function bindtextdomain: `bindtextdomain` function bind\_textdomain\_codeset: `bind_textdomain_codeset` function setlocale: Use `setlocale (LC_ALL, "");` Prerequisite: `use POSIX;` `use Locale::TextDomain;` (included in the package libintl-perl which is available on the Comprehensive Perl Archive Network CPAN, http://www.cpan.org/). Use or emulate GNU gettext: platform dependent: gettext\_pp emulates, gettext\_xs uses GNU gettext Extractor: `xgettext -k__ -k\$__ -k%__ -k__x -k__n:1,2 -k__nx:1,2 -k__xn:1,2 -kN__ -k` Formatting with positions: Both kinds of format strings support formatting with positions. `printf "%2\$d %1\$d", ...` (requires Perl 5.8.0 or newer) `__expand("[new] replaces [old]", old => $oldvalue, new => $newvalue)` Portability: The `libintl-perl` package is platform independent but is not part of the Perl core. The programmer is responsible for providing a dummy implementation of the required functions if the package is not installed on the target system. po-mode marking: --- Documentation: Included in `libintl-perl`, available on CPAN (http://www.cpan.org/).

An example is available in the ‘examples’ directory: `hello-perl`.

The `xgettext` parser backend for Perl differs significantly from
the parser backends for other programming languages, just as Perl
itself differs significantly from other programming languages. The
Perl parser backend offers many more string marking facilities than
the other backends but it also has some Perl specific limitations, the
worst probably being its imperfectness.

#### 15.5.18.1 General Problems Parsing Perl Code

It is often heard that only Perl can parse Perl. This is not true.
Perl cannot be *parsed* at all, it can only be *executed*.
Perl has various built-in ambiguities that can only be resolved at runtime.

The following example may illustrate one common problem:

```
print gettext "Hello World!";
```

Although this example looks like a bullet-proof case of a function
invocation, it is not:

```
open gettext, ">testfile" or die;
print gettext "Hello world!"
```

In this context, the string `gettext` looks more like a
file handle. But not necessarily:

```
use Locale::Messages qw (:libintl_h);
open gettext ">testfile" or die;
print gettext "Hello world!";
```

Now, the file is probably syntactically incorrect, provided that the module
`Locale::Messages` found first in the Perl include path exports a
function `gettext`. But what if the module
`Locale::Messages` really looks like this?

```
use vars qw (*gettext);

1;
```

In this case, the string `gettext` will be interpreted as a file
handle again, and the above example will create a file ‘testfile’
and write the string “Hello world!” into it. Even advanced
control flow analysis will not really help:

```
if (0.5 < rand) {
   eval "use Sane";
} else {
   eval "use InSane";
}
print gettext "Hello world!";
```

If the module `Sane` exports a function `gettext` that does
what we expect, and the module `InSane` opens a file for writing
and associates the *handle* `gettext` with this output
stream, we are clueless again about what will happen at runtime. It is
completely unpredictable. The truth is that Perl has so many ways to
fill its symbol table at runtime that it is impossible to interpret a
particular piece of code without executing it.

Of course, `xgettext` will not execute your Perl sources while
scanning for translatable strings, but rather use heuristics in order
to guess what you meant.

Another problem is the ambiguity of the slash and the question mark.
Their interpretation depends on the context:

```
# A pattern match.
print "OK\n" if /foobar/;

# A division.
print 1 / 2;

# Another pattern match.
print "OK\n" if ?foobar?;

# Conditional.
print $x ? "foo" : "bar";
```

The slash may either act as the division operator or introduce a
pattern match, whereas the question mark may act as the ternary
conditional operator or as a pattern match, too. Other programming
languages like `awk` present similar problems, but the consequences of a
misinterpretation are particularly nasty with Perl sources. In `awk`
for instance, a statement can never exceed one line and the parser
can recover from a parsing error at the next newline and interpret
the rest of the input stream correctly. Perl is different, as a
pattern match is terminated by the next appearance of the delimiter
(the slash or the question mark) in the input stream, regardless of
the semantic context. If a slash is really a division sign but
mis-interpreted as a pattern match, the rest of the input file is most
probably parsed incorrectly.

There are certain cases, where the ambiguity cannot be resolved at all:

```
$x = wantarray ? 1 : 0;
```

The Perl built-in function `wantarray` does not accept any arguments.
The Perl parser therefore knows that the question mark does not start
a regular expression but is the ternary conditional operator.

```
sub wantarrays {}
$x = wantarrays ? 1 : 0;
```

Now the situation is different. The function `wantarrays` takes
a variable number of arguments (like any non-prototyped Perl function).
The question mark is now the delimiter of a pattern match, and hence
the piece of code does not compile.

```
sub wantarrays() {}
$x = wantarrays ? 1 : 0;
```

Now the function is prototyped, Perl knows that it does not accept any
arguments, and the question mark is therefore interpreted as the
ternaray operator again. But that unfortunately outsmarts `xgettext`.

The Perl parser in `xgettext` cannot know whether a function has
a prototype and what that prototype would look like. It therefore makes
an educated guess. If a function is known to be a Perl built-in and
this function does not accept any arguments, a following question mark
or slash is treated as an operator, otherwise as the delimiter of a
following regular expression. The Perl built-ins that do not accept
arguments are `wantarray`, `fork`, `time`, `times`,
`getlogin`, `getppid`, `getpwent`, `getgrent`,
`gethostent`, `getnetent`, `getprotoent`, `getservent`,
`setpwent`, `setgrent`, `endpwent`, `endgrent`,
`endhostent`, `endnetent`, `endprotoent`, and
`endservent`.

If you find that `xgettext` fails to extract strings from
portions of your sources, you should therefore look out for slashes
and/or question marks preceding these sections. You may have come
across a bug in `xgettext`'s Perl parser (and of course you
should report that bug). In the meantime you should consider to
reformulate your code in a manner less challenging to `xgettext`.

In particular, if the parser is too dumb to see that a function
does not accept arguments, use parentheses:

```
$x = somefunc() ? 1 : 0;
$y = (somefunc) ? 1 : 0;
```

In fact the Perl parser itself has similar problems and warns you
about such constructs.

#### 15.5.18.2 Which keywords will xgettext look for?

Unless you instruct `xgettext` otherwise by invoking it with one
of the options `--keyword` or `-k`, it will recognize the
following keywords in your Perl sources:

- `gettext`- `dgettext`- `dcgettext`- `ngettext:1,2`
        The first (singular) and the second (plural) argument will be
        extracted.- `dngettext:1,2`
          The first (singular) and the second (plural) argument will be
          extracted.- `dcngettext:1,2`
            The first (singular) and the second (plural) argument will be
            extracted.- `gettext_noop`- `%gettext`
                The keys of lookups into the hash `%gettext` will be extracted.- `$gettext`
                  The keys of lookups into the hash reference `$gettext` will be extracted.

#### 15.5.18.3 How to Extract Hash Keys

Translating messages at runtime is normally performed by looking up the
original string in the translation database and returning the
translated version. The “natural” Perl implementation is a hash
lookup, and, of course, `xgettext` supports such practice.

```
print __"Hello world!";
print $__{"Hello world!"};
print $__->{"Hello world!"};
print $$__{"Hello world!"};
```

The above four lines all do the same thing. The Perl module
`Locale::TextDomain` exports by default a hash `%__` that
is tied to the function `__()`. It also exports a reference
`$__` to `%__`.

If an argument to the `xgettext` option `--keyword`,
resp. `-k` starts with a percent sign, the rest of the keyword is
interpreted as the name of a hash. If it starts with a dollar
sign, the rest of the keyword is interpreted as a reference to a
hash.

Note that you can omit the quotation marks (single or double) around
the hash key (almost) whenever Perl itself allows it:

```
print $gettext{Error};
```

The exact rule is: You can omit the surrounding quotes, when the hash
key is a valid C (!) identifier, i.e. when it starts with an
underscore or an ASCII letter and is followed by an arbitrary number
of underscores, ASCII letters or digits. Other Unicode characters
are *not* allowed, regardless of the `use utf8` pragma.

#### 15.5.18.4 What are Strings And Quote-like Expressions?

Perl offers a plethora of different string constructs. Those that can
be used either as arguments to functions or inside braces for hash
lookups are generally supported by `xgettext`.

- **double-quoted strings**
    

  ```
  print gettext "Hello World!";
  ```

  - **single-quoted strings**
      

    ```
    print gettext 'Hello World!';
    ```

    - **the operator qq**
        

      ```
      print gettext qq |Hello World!|;
      print gettext qq <E-mail: <guido\@imperia.net>>;
      ```

      The operator `qq` is fully supported. You can use arbitrary
      delimiters, including the four bracketing delimiters (round, angle,
      square, curly) that nest.- **the operator q**
          

        ```
        print gettext q |Hello World!|;
        print gettext q <E-mail: <guido@imperia.net>>;
        ```

        The operator `q` is fully supported. You can use arbitrary
        delimiters, including the four bracketing delimiters (round, angle,
        square, curly) that nest.- **the operator qx**
            

          ```
          print gettext qx ;LANGUAGE=C /bin/date;
          print gettext qx [/usr/bin/ls | grep '^[A-Z]*'];
          ```

          The operator `qx` is fully supported. You can use arbitrary
          delimiters, including the four bracketing delimiters (round, angle,
          square, curly) that nest.
          The example is actually a useless use of `gettext`. It will
          invoke the `gettext` function on the output of the command
          specified with the `qx` operator. The feature was included
          in order to make the interface consistent (the parser will extract
          all strings and quote-like expressions).- **here documents**
              

            ```
            print gettext <<'EOF';
            program not found in $PATH
            EOF

            print ngettext <<EOF, <<"EOF";
            one file deleted
            EOF
            several files deleted
            EOF
            ```

            Here-documents are recognized. If the delimiter is enclosed in single
            quotes, the string is not interpolated. If it is enclosed in double
            quotes or has no quotes at all, the string is interpolated.
            Delimiters that start with a digit are not supported!

#### 15.5.18.5 Invalid Uses Of String Interpolation

Perl is capable of interpolating variables into strings. This offers
some nice features in localized programs but can also lead to
problems.

A common error is a construct like the following:

```
print gettext "This is the program $0!\n";
```

Perl will interpolate at runtime the value of the variable `$0`
into the argument of the `gettext()` function. Hence, this
argument is not a string constant but a variable argument (`$0`
is a global variable that holds the name of the Perl script being
executed). The interpolation is performed by Perl before the string
argument is passed to `gettext()` and will therefore depend on
the name of the script which can only be determined at runtime.
Consequently, it is almost impossible that a translation can be looked
up at runtime (except if, by accident, the interpolated string is found
in the message catalog).

The `xgettext` program will therefore terminate parsing with a fatal
error if it encounters a variable inside of an extracted string. In
general, this will happen for all kinds of string interpolations that
cannot be safely performed at compile time. If you absolutely know
what you are doing, you can always circumvent this behavior:

```
my $know_what_i_am_doing = "This is program $0!\n";
print gettext $know_what_i_am_doing;
```

Since the parser only recognizes strings and quote-like expressions,
but not variables or other terms, the above construct will be
accepted. You will have to find another way, however, to let your
original string make it into your message catalog.

If invoked with the option `--extract-all`, resp. `-a`,
variable interpolation will be accepted. Rationale: You will
generally use this option in order to prepare your sources for
internationalization.

Please see the manual page `‘man perlop’` for details of strings and
quote-like expressions that are subject to interpolation and those
that are not. Safe interpolations (that will not lead to a fatal
error) are:

- the escape sequences `\t` (tab, HT, TAB), `\n`
  (newline, NL), `\r` (return, CR), `\f` (form feed, FF),
  `\b` (backspace, BS), `\a` (alarm, bell, BEL), and `\e`
  (escape, ESC).- octal chars, like `\033`
      
    Note that octal escapes in the range of 400-777 are translated into a
    UTF-8 representation, regardless of the presence of the `use utf8` pragma.- hex chars, like `\x1b`- wide hex chars, like `\x{263a}`
          
        Note that this escape is translated into a UTF-8 representation,
        regardless of the presence of the `use utf8` pragma.- control chars, like `\c[` (CTRL-[)- named Unicode chars, like `\N{LATIN CAPITAL LETTER C WITH CEDILLA}`
              
            Note that this escape is translated into a UTF-8 representation,
            regardless of the presence of the `use utf8` pragma.

The following escapes are considered partially safe:

- `\l` lowercase next char- `\u` uppercase next char- `\L` lowercase till \E- `\U` uppercase till \E- `\E` end case modification- `\Q` quote non-word characters till \E

These escapes are only considered safe if the string consists of
ASCII characters only. Translation of characters outside the range
defined by ASCII is locale-dependent and can actually only be performed
at runtime; `xgettext` doesn't do these locale-dependent translations
at extraction time.

Except for the modifier `\Q`, these translations, albeit valid,
are generally useless and only obfuscate your sources. If a
translation can be safely performed at compile time you can just as
well write what you mean.

#### 15.5.18.6 Valid Uses Of String Interpolation

Perl is often used to generate sources for other programming languages
or arbitrary file formats. Web applications that output HTML code
make a prominent example for such usage.

You will often come across situations where you want to intersperse
code written in the target (programming) language with translatable
messages, like in the following HTML example:

```
print gettext <<EOF;
<h1>My Homepage</h1>
<script language="JavaScript"><!--
for (i = 0; i < 100; ++i) {
    alert ("Thank you so much for visiting my homepage!");
}
//--></script>
EOF
```

The parser will extract the entire here document, and it will appear
entirely in the resulting PO file, including the JavaScript snippet
embedded in the HTML code. If you exaggerate with constructs like
the above, you will run the risk that the translators of your package
will look out for a less challenging project. You should consider an
alternative expression here:

```
print <<EOF;
<h1>$gettext{"My Homepage"}</h1>
<script language="JavaScript"><!--
for (i = 0; i < 100; ++i) {
    alert ("$gettext{'Thank you so much for visiting my homepage!'}");
}
//--></script>
EOF
```

Only the translatable portions of the code will be extracted here, and
the resulting PO file will begrudgingly improve in terms of readability.

You can interpolate hash lookups in all strings or quote-like
expressions that are subject to interpolation (see the manual page
`‘man perlop’` for details). Double interpolation is invalid, however:

```
# TRANSLATORS: Replace "the earth" with the name of your planet.
print gettext qq{Welcome to $gettext->{"the earth"}};
```

The `qq`-quoted string is recognized as an argument to `xgettext` in
the first place, and checked for invalid variable interpolation. The
dollar sign of hash-dereferencing will therefore terminate the parser
with an “invalid interpolation” error.

It is valid to interpolate hash lookups in regular expressions:

```
if ($var =~ /$gettext{"the earth"}/) {
   print gettext "Match!\n";
}
s/$gettext{"U. S. A."}/$gettext{"U. S. A."} $gettext{"(dial +0)"}/g;
```

#### 15.5.18.7 When To Use Parentheses

In Perl, parentheses around function arguments are mostly optional.
`xgettext` will always assume that all
recognized keywords (except for hashes and hash references) are names
of properly prototyped functions, and will (hopefully) only require
parentheses where Perl itself requires them. All constructs in the
following example are therefore ok to use:

```
print gettext ("Hello World!\n");
print gettext "Hello World!\n";
print dgettext ($package => "Hello World!\n");
print dgettext $package, "Hello World!\n";

# The "fat comma" => turns the left-hand side argument into a
# single-quoted string!
print dgettext smellovision => "Hello World!\n";

# The following assignment only works with prototyped functions.
# Otherwise, the functions will act as "greedy" list operators and
# eat up all following arguments.
my $anonymous_hash = {
   planet => gettext "earth",
   cakes => ngettext "one cake", "several cakes", $n,
   still => $works,
};
# The same without fat comma:
my $other_hash = {
   'planet', gettext "earth",
   'cakes', ngettext "one cake", "several cakes", $n,
   'still', $works,
};

# Parentheses are only significant for the first argument.
print dngettext 'package', ("one cake", "several cakes", $n), $discarded;
```

#### 15.5.18.8 How To Grok with Long Lines

The necessity of long messages can often lead to a cumbersome or
unreadable coding style. Perl has several options that may prevent
you from writing unreadable code, and
`xgettext` does its best to do likewise. This is where the dot
operator (the string concatenation operator) may come in handy:

```
print gettext ("This is a very long"
               . " message that is still"
               . " readable, because"
               . " it is split into"
               . " multiple lines.\n");
```

Perl is smart enough to concatenate these constant string fragments
into one long string at compile time, and so is
`xgettext`. You will only find one long message in the resulting
POT file.

Note that the future Perl 6 will probably use the underscore
(`‘_’`) as the string concatenation operator, and the dot
(`‘.’`) for dereferencing. This new syntax is not yet supported by
`xgettext`.

If embedded newline characters are not an issue, or even desired, you
may also insert newline characters inside quoted strings wherever you
feel like it:

```
print gettext ("<em>In HTML output
embedded newlines are generally no
problem, since adjacent whitespace
is always rendered into a single
space character.</em>");
```

You may also consider to use here documents:

```
print gettext <<EOF;
<em>In HTML output
embedded newlines are generally no
problem, since adjacent whitespace
is always rendered into a single
space character.</em>
EOF
```

Please do not forget that the line breaks are real, i.e. they
translate into newline characters that will consequently show up in
the resulting POT file.

#### 15.5.18.9 Bugs, Pitfalls, And Things That Do Not Work

The foregoing sections should have proven that
`xgettext` is quite smart in extracting translatable strings from
Perl sources. Yet, some more or less exotic constructs that could be
expected to work, actually do not work.

One of the more relevant limitations can be found in the
implementation of variable interpolation inside quoted strings. Only
simple hash lookups can be used there:

```
print <<EOF;
$gettext{"The dot operator"
          . " does not work"
          . "here!"}
Likewise, you cannot @{[ gettext ("interpolate function calls") ]}
inside quoted strings or quote-like expressions.
EOF
```

This is valid Perl code and will actually trigger invocations of the
`gettext` function at runtime. Yet, the Perl parser in
`xgettext` will fail to recognize the strings. A less obvious
example can be found in the interpolation of regular expressions:

```
s/<!--START_OF_WEEK-->/gettext ("Sunday")/e;
```

The modifier `e` will cause the substitution to be interpreted as
an evaluable statement. Consequently, at runtime the function
`gettext()` is called, but again, the parser fails to extract the
string “Sunday”. Use a temporary variable as a simple workaround if
you really happen to need this feature:

```
my $sunday = gettext "Sunday";
s/<!--START_OF_WEEK-->/$sunday/;
```

Hash slices would also be handy but are not recognized:

```
my @weekdays = @gettext{'Sunday', 'Monday', 'Tuesday', 'Wednesday',
                        'Thursday', 'Friday', 'Saturday'};
# Or even:
@weekdays = @gettext{qw (Sunday Monday Tuesday Wednesday Thursday
                         Friday Saturday) };
```

This is perfectly valid usage of the tied hash `%gettext` but the
strings are not recognized and therefore will not be extracted.

Another caveat of the current version is its rudimentary support for
non-ASCII characters in identifiers. You may encounter serious
problems if you use identifiers with characters outside the range of
'A'-'Z', 'a'-'z', '0'-'9' and the underscore '\_'.

Maybe some of these missing features will be implemented in future
versions, but since you can always make do without them at minimal effort,
these todos have very low priority.

A nasty problem are brace format strings that already contain braces
as part of the normal text, for example the usage strings typically
encountered in programs:

```
die "usage: $0 {OPTIONS} FILENAME...\n";
```

If you want to internationalize this code with Perl brace format strings,
you will run into a problem:

```
die __x ("usage: {program} {OPTIONS} FILENAME...\n", program => $0);
```

Whereas `‘{program}’` is a placeholder, `‘{OPTIONS}’`
is not and should probably be translated. Yet, there is no way to teach
the Perl parser in `xgettext` to recognize the first one, and leave
the other one alone.

There are two possible work-arounds for this problem. If you are
sure that your program will run under Perl 5.8.0 or newer (these
Perl versions handle positional parameters in `printf()`) or
if you are sure that the translator will not have to reorder the arguments
in her translation -- for example if you have only one brace placeholder
in your string, or if it describes a syntax, like in this one --, you can
mark the string as `no-perl-brace-format` and use `printf()`:

```
# xgettext: no-perl-brace-format
die sprintf ("usage: %s {OPTIONS} FILENAME...\n", $0);
```

If you want to use the more portable Perl brace format, you will have to do
put placeholders in place of the literal braces:

```
die __x ("usage: {program} {[}OPTIONS{]} FILENAME...\n",
         program => $0, '[' => '{', ']' => '}');
```

Perl brace format strings know no escaping mechanism. No matter how this
escaping mechanism looked like, it would either give the programmer a
hard time, make translating Perl brace format strings heavy-going, or
result in a performance penalty at runtime, when the format directives
get executed. Most of the time you will happily get along with
`printf()` for this special case.

### 15.5.19 PHP Hypertext Preprocessor

RPMs: mod\_php4, mod\_php4-core, phpdoc File extension: `php`, `php3`, `php4` String syntax: `"abc"`, `'abc'` gettext shorthand: `_("abc")` gettext/ngettext functions: `gettext`, `dgettext`, `dcgettext`; starting with PHP 4.2.0 also `ngettext`, `dngettext`, `dcngettext` textdomain: `textdomain` function bindtextdomain: `bindtextdomain` function setlocale: Programmer must call `setlocale (LC_ALL, "")` Prerequisite: --- Use or emulate GNU gettext: use Extractor: `xgettext` Formatting with positions: `printf "%2\$d %1\$d"` Portability: On platforms without gettext, the functions are not available. po-mode marking: ---

An example is available in the ‘examples’ directory: `hello-php`.

### 15.5.20 Pike

RPMs: roxen File extension: `pike` String syntax: `"abc"` gettext shorthand: --- gettext/ngettext functions: `gettext`, `dgettext`, `dcgettext` textdomain: `textdomain` function bindtextdomain: `bindtextdomain` function setlocale: `setlocale` function Prerequisite: `import Locale.Gettext;` Use or emulate GNU gettext: use Extractor: --- Formatting with positions: --- Portability: On platforms without gettext, the functions are not available. po-mode marking: ---

### 15.5.21 GNU Compiler Collection sources

RPMs: gcc File extension: `c`, `h`. String syntax: `"abc"` gettext shorthand: `_("abc")` gettext/ngettext functions: `gettext`, `dgettext`, `dcgettext`, `ngettext`, `dngettext`, `dcngettext` textdomain: `textdomain` function bindtextdomain: `bindtextdomain` function setlocale: Programmer must call `setlocale (LC_ALL, "")` Prerequisite: `#include "intl.h"` Use or emulate GNU gettext: Use Extractor: `xgettext -k_` Formatting with positions: --- Portability: Uses autoconf macros po-mode marking: yes

## 15.6 Internationalizable Data

Here is a list of other data formats which can be internationalized
using GNU gettext.

### 15.6.1 POT - Portable Object Template

RPMs: gettext File extension: `pot`, `po` Extractor: `xgettext`

### 15.6.2 Resource String Table

RPMs: fpk File extension: `rst` Extractor: `xgettext`, `rstconv`

### 15.6.3 Glade - GNOME user interface description

RPMs: glade, libglade, glade2, libglade2, intltool File extension: `glade`, `glade2` Extractor: `xgettext`, `libglade-xgettext`, `xml-i18n-extract`, `intltool-extract`

---

Go to the first, previous, next, last section, table of contents.
