# Supplementary material for: rDock: A Fast, Versatile and Open Source Program for Docking Ligands to Proteins and Nucleic Acids
Source: PLoS Comput Biol. 2014 Apr 10;10(4):e1003571. doi: 10.1371/journal.pcbi.1003571 (PMC3983074; doi:10.1371/journal.pcbi.1003571)
Supplement: Software S1 — Compressed file with the source code of the rDock software for ligand docking to Proteins and Nucleic Acids. (GZ) [file pcbi.1003571.s010.gz › rDock_2013.1_src/gettext-0.18.tar.gz!/gettext-0.18/gettext-tools/doc/gettext_21.html]

 
 
 

 
 GNU gettext utilities - Option Index 
 
 
Go to the  first ,  previous ,  next ,  last  section,  table of contents .
   


  Option Index  

 
Jump to:
 - 
 
  -  
 
  --add-comments,  xgettext  option 
  --add-location,  msgattrib  option 
  --add-location,  msgcat  option 
  --add-location,  msgcomm  option 
  --add-location,  msgconv  option 
  --add-location,  msgen  option 
  --add-location,  msgfilter  option 
  --add-location,  msggrep  option 
  --add-location,  msgmerge  option 
  --add-location,  msguniq  option 
  --add-location,  xgettext  option 
  --alignment,  msgfmt  option 
  --backup,  msgmerge  option 
  --boost,  xgettext  option 
  --c++,  xgettext  option 
  --check,  msgfmt  option 
  --check-accelerators,  msgfmt  option 
  --check-compatibility,  msgfmt  option 
  --check-domain,  msgfmt  option 
  --check-format,  msgfmt  option 
  --check-header,  msgfmt  option 
  --clear-fuzzy,  msgattrib  option 
  --clear-obsolete,  msgattrib  option 
  --clear-previous,  msgattrib  option 
  --color,  msgattrib  option 
  --color,  msgcat  option ,  --color,  msgcat  option 
  --color,  msgcomm  option 
  --color,  msgconv  option 
  --color,  msgen  option 
  --color,  msgfilter  option 
  --color,  msggrep  option 
  --color,  msginit  option 
  --color,  msgmerge  option 
  --color,  msgunfmt  option 
  --color,  msguniq  option 
  --color,  xgettext  option 
  --comment,  msggrep  option 
  --compendium,  msgmerge  option 
  --copyright-holder,  xgettext  option 
  --csharp,  msgfmt  option 
  --csharp,  msgunfmt  option 
  --csharp-resources,  msgfmt  option 
  --csharp-resources,  msgunfmt  option 
  --debug,  xgettext  option 
  --default-domain,  xgettext  option 
  --directory,  msgattrib  option 
  --directory,  msgcat  option 
  --directory,  msgcmp  option 
  --directory,  msgcomm  option 
  --directory,  msgconv  option 
  --directory,  msgen  option 
  --directory,  msgexec  option 
  --directory,  msgfilter  option 
  --directory,  msgfmt  option 
  --directory,  msggrep  option 
  --directory,  msgmerge  option 
  --directory,  msguniq  option 
  --directory,  xgettext  option 
  --domain,  gettext  option 
  --domain,  msggrep  option 
  --domain,  ngettext  option 
  --dry-run,  autopoint  option 
  --dry-run,  gettextize  option 
  --exclude-file,  xgettext  option 
  --expression,  msgfilter  option 
  --extended-regexp,  msggrep  option 
  --extract-all,  xgettext  option 
  --extracted-comment,  msggrep  option 
  --file,  msgfilter  option 
  --file,  msggrep  option 
  --files-from,  msgcat  option 
  --files-from,  msgcomm  option 
  --files-from,  xgettext  option 
  --fixed-strings,  msggrep  option 
  --flag,  xgettext  option 
  --force,  autopoint  option 
  --force,  gettextize  option 
  --force-po,  msgattrib  option 
  --force-po,  msgcat  option 
  --force-po,  msgcomm  option 
  --force-po,  msgconv  option 
  --force-po,  msgen  option 
  --force-po,  msgfilter  option 
  --force-po,  msggrep  option 
  --force-po,  msgmerge  option 
  --force-po,  msgunfmt  option 
  --force-po,  msguniq  option 
  --force-po,  xgettext  option 
  --foreign-user,  xgettext  option 
  --from-code,  xgettext  option 
  --fuzzy,  msgattrib  option 
  --help,  autopoint  option 
  --help,  envsubst  option 
  --help,  gettext  option 
  --help,  gettextize  option 
  --help,  msgattrib  option 
  --help,  msgcat  option 
  --help,  msgcmp  option 
  --help,  msgcomm  option 
  --help,  msgconv  option 
  --help,  msgen  option 
  --help,  msgexec  option 
  --help,  msgfilter  option 
  --help,  msgfmt  option 
  --help,  msggrep  option 
  --help,  msginit  option 
  --help,  msgmerge  option 
  --help,  msgunfmt  option 
  --help,  msguniq  option 
  --help,  ngettext  option 
  --help,  xgettext  option 
  --ignore-case,  msggrep  option 
  --ignore-file,  msgattrib  option 
  --indent,  msgattrib  option 
  --indent,  msgcat  option 
  --indent,  msgcomm  option 
  --indent,  msgconv  option 
  --indent,  msgen  option 
  --indent,  msgfilter  option 
  --indent,  msggrep  option 
  --indent,  msgmerge  option 
  --indent,  msgunfmt  option 
  --indent,  msguniq  option 
  --indent,  xgettext  option 
  --input,  msgexec  option 
  --input,  msgfilter  option 
  --input,  msginit  option 
  --intl,  gettextize  option 
  --invert-match,  msggrep  option 
  --java,  msgfmt  option 
  --java,  msgunfmt  option 
  --java2,  msgfmt  option 
  --join-existing,  xgettext  option 
  --kde,  xgettext  option 
  --keep-header,  msgfilter  option 
  --keyword,  xgettext  option 
  --lang,  msgcat  option ,  --lang,  msgcat  option ,  --lang,  msgcat  option 
  --language,  xgettext  option 
  --less-than,  msgcat  option 
  --less-than,  msgcomm  option 
  --locale,  msgfmt  option ,  --locale,  msgfmt  option ,  --locale,  msgfmt  option 
  --locale,  msginit  option 
  --locale,  msgunfmt  option ,  --locale,  msgunfmt  option ,  --locale,  msgunfmt  option 
  --location,  msggrep  option 
  --more-than,  msgcat  option 
  --more-than,  msgcomm  option 
  --msgctxt,  msggrep  option 
  --msgid,  msggrep  option 
  --msgid-bugs-address,  xgettext  option 
  --msgstr,  msggrep  option 
  --msgstr-prefix,  xgettext  option 
  --msgstr-suffix,  xgettext  option 
  --multi-domain,  msgcmp  option 
  --multi-domain,  msgmerge  option 
  --no-changelog,  gettextize  option 
  --no-fuzzy,  msgattrib  option 
  --no-fuzzy-matching,  msgcmp  option 
  --no-fuzzy-matching,  msgmerge  option 
  --no-hash,  msgfmt  option 
  --no-location,  msgattrib  option 
  --no-location,  msgcat  option 
  --no-location,  msgcomm  option 
  --no-location,  msgconv  option 
  --no-location,  msgen  option 
  --no-location,  msgfilter  option 
  --no-location,  msggrep  option 
  --no-location,  msgmerge  option 
  --no-location,  msguniq  option 
  --no-location,  xgettext  option 
  --no-obsolete,  msgattrib  option 
  --no-translator,  msginit  option 
  --no-wrap,  msgattrib  option 
  --no-wrap,  msgcat  option 
  --no-wrap,  msgcomm  option 
  --no-wrap,  msgconv  option 
  --no-wrap,  msgen  option 
  --no-wrap,  msgfilter  option 
  --no-wrap,  msggrep  option 
  --no-wrap,  msginit  option 
  --no-wrap,  msgmerge  option 
  --no-wrap,  msgunfmt  option 
  --no-wrap,  msguniq  option 
  --no-wrap,  xgettext  option 
  --obsolete,  msgattrib  option 
  --omit-header,  msgcomm  option 
  --omit-header,  xgettext  option 
  --only-file,  msgattrib  option 
  --only-fuzzy,  msgattrib  option 
  --only-obsolete,  msgattrib  option 
  --output,  xgettext  option 
  --output-dir,  xgettext  option 
  --output-file,  msgattrib  option 
  --output-file,  msgcat  option 
  --output-file,  msgcomm  option 
  --output-file,  msgconv  option 
  --output-file,  msgen  option 
  --output-file,  msgfilter  option 
  --output-file,  msgfmt  option 
  --output-file,  msggrep  option 
  --output-file,  msginit  option 
  --output-file,  msgmerge  option 
  --output-file,  msgunfmt  option 
  --output-file,  msguniq  option 
  --package-name,  xgettext  option 
  --package-version,  xgettext  option 
  --po-dir,  gettextize  option 
  --previous,  msgmerge  option 
  --properties-input,  msgattrib  option 
  --properties-input,  msgcat  option 
  --properties-input,  msgcmp  option 
  --properties-input,  msgcomm  option 
  --properties-input,  msgconv  option 
  --properties-input,  msgen  option 
  --properties-input,  msgexec  option 
  --properties-input,  msgfilter  option 
  --properties-input,  msgfmt  option 
  --properties-input,  msggrep  option 
  --properties-input,  msginit  option 
  --properties-input,  msgmerge  option 
  --properties-input,  msguniq  option 
  --properties-output,  msgattrib  option 
  --properties-output,  msgcat  option 
  --properties-output,  msgcomm  option 
  --properties-output,  msgconv  option 
  --properties-output,  msgen  option 
  --properties-output,  msgfilter  option 
  --properties-output,  msggrep  option 
  --properties-output,  msginit  option 
  --properties-output,  msgmerge  option 
  --properties-output,  msgunfmt  option 
  --properties-output,  msguniq  option 
  --properties-output,  xgettext  option 
  --qt,  msgfmt  option 
  --qt,  xgettext  option 
  --quiet,  msgfilter  option 
  --quiet,  msgmerge  option 
  --regexp=,  msggrep  option 
  --repeated,  msguniq  option 
  --resource,  msgfmt  option ,  --resource,  msgfmt  option 
  --resource,  msgunfmt  option ,  --resource,  msgunfmt  option 
  --set-fuzzy,  msgattrib  option 
  --set-obsolete,  msgattrib  option 
  --silent,  msgfilter  option 
  --silent,  msgmerge  option 
  --sort-by-file,  msgattrib  option 
  --sort-by-file,  msgcat  option 
  --sort-by-file,  msgcomm  option 
  --sort-by-file,  msgconv  option 
  --sort-by-file,  msgen  option 
  --sort-by-file,  msgfilter  option 
  --sort-by-file,  msggrep  option 
  --sort-by-file,  msgmerge  option 
  --sort-by-file,  msguniq  option 
  --sort-by-file,  xgettext  option 
  --sort-output,  msgattrib  option 
  --sort-output,  msgcat  option 
  --sort-output,  msgcomm  option 
  --sort-output,  msgconv  option 
  --sort-output,  msgen  option 
  --sort-output,  msgfilter  option 
  --sort-output,  msggrep  option 
  --sort-output,  msgmerge  option 
  --sort-output,  msgunfmt  option 
  --sort-output,  msguniq  option 
  --sort-output,  xgettext  option 
  --statistics,  msgfmt  option 
  --strict,  msgattrib  option 
  --strict,  msgcat  option 
  --strict,  msgcomm  option 
  --strict,  msgconv  option 
  --strict,  msgen  option 
  --strict,  msgfilter  option 
  --strict,  msgfmt  option 
  --strict,  msggrep  option 
  --strict,  msgmerge  option 
  --strict,  msgunfmt  option 
  --strict,  msguniq  option 
  --strict,  xgettext  option 
  --stringtable-input,  msgattrib  option 
  --stringtable-input,  msgcat  option 
  --stringtable-input,  msgcmp  option 
  --stringtable-input,  msgcomm  option 
  --stringtable-input,  msgen  option 
  --stringtable-input,  msgexec  option 
  --stringtable-input,  msgfilter  option 
  --stringtable-input,  msgfmt  option 
  --stringtable-input,  msggrep  option 
  --stringtable-input,  msginit  option 
  --stringtable-input,  msgmerge  option 
  --stringtable-input,  msgonv  option 
  --stringtable-input,  msguniq  option 
  --stringtable-output,  msgattrib  option 
  --stringtable-output,  msgcat  option 
  --stringtable-output,  msgcomm  option 
  --stringtable-output,  msgconv  option 
  --stringtable-output,  msgen  option 
  --stringtable-output,  msgfilter  option 
  --stringtable-output,  msggrep  option 
  --stringtable-output,  msginit  option 
  --stringtable-output,  msgmerge  option 
  --stringtable-output,  msgunfmt  option 
  --stringtable-output,  msguniq  option 
  --stringtable-output,  xgettext  option 
  --style,  msgattrib  option 
  --style,  msgcat  option ,  --style,  msgcat  option 
  --style,  msgcomm  option 
  --style,  msgconv  option 
  --style,  msgen  option 
  --style,  msgfilter  option 
  --style,  msggrep  option 
  --style,  msginit  option 
  --style,  msgmerge  option 
  --style,  msgunfmt  option 
  --style,  msguniq  option 
  --style,  xgettext  option 
  --suffix,  msgmerge  option 
  --symlink,  gettextize  option 
  --tcl,  msgfmt  option 
  --tcl,  msgunfmt  option 
  --to-code,  msgcat  option 
  --to-code,  msgconv  option 
  --to-code,  msguniq  option 
  --translated,  msgattrib  option 
  --trigraphs,  xgettext  option 
  --unique,  msgcat  option 
  --unique,  msgcomm  option 
  --unique,  msguniq  option 
  --untranslated,  msgattrib  option 
  --update,  msgmerge  option 
  --use-first,  msgcat  option 
  --use-first,  msguniq  option 
  --use-fuzzy,  msgcmp  option 
  --use-fuzzy,  msgfmt  option 
  --use-untranslated,  msgcmp  option 
  --variables,  envsubst  option 
  --verbose,  msgfmt  option 
  --verbose,  msgmerge  option 
  --verbose,  msgunfmt  option 
  --version,  autopoint  option 
  --version,  envsubst  option 
  --version,  gettext  option 
  --version,  gettextize  option 
  --version,  msgattrib  option 
  --version,  msgcat  option 
  --version,  msgcmp  option 
  --version,  msgcomm  option 
  --version,  msgconv  option 
  --version,  msgen  option 
  --version,  msgexec  option 
  --version,  msgfilter  option 
  --version,  msgfmt  option 
  --version,  msggrep  option 
  --version,  msginit  option 
  --version,  msgmerge  option 
  --version,  msgunfmt  option 
  --version,  msguniq  option 
  --version,  ngettext  option 
  --version,  xgettext  option 
  --width,  msgattrib  option 
  --width,  msgcat  option 
  --width,  msgcomm  option 
  --width,  msgconv  option 
  --width,  msgen  option 
  --width,  msgfilter  option 
  --width,  msggrep  option 
  --width,  msginit  option 
  --width,  msgmerge  option 
  --width,  msgunfmt  option 
  --width,  msguniq  option 
  --width,  xgettext  option 
  -<,  msgcat  option 
  -<,  msgcomm  option 
  ->,  msgcat  option 
  ->,  msgcomm  option 
  -a,  msgfmt  option 
  -a,  xgettext  option 
  -C,  msgfmt  option 
  -c,  msgfmt  option 
  -C,  msggrep  option 
  -C,  msgmerge  option 
  -C,  xgettext  option 
  -c,  xgettext  option 
  -d,  autopoint  option 
  -d,  gettext  option 
  -d,  gettextize  option 
  -D,  msgattrib  option 
  -D,  msgcat  option 
  -D,  msgcmp  option 
  -D,  msgcomm  option 
  -D,  msgconv  option 
  -D,  msgen  option 
  -D,  msgexec  option 
  -D,  msgfilter  option 
  -d,  msgfmt  option ,  -d,  msgfmt  option ,  -d,  msgfmt  option 
  -D,  msgfmt  option 
  -D,  msggrep  option 
  -D,  msgmerge  option 
  -d,  msgunfmt  option ,  -d,  msgunfmt  option 
  -D,  msguniq  option 
  -d,  msguniq  option 
  -d,  ngettext  option 
  -D,  xgettext  option 
  -d,  xgettext  option 
  -e,  gettext  option 
  -E,  gettext  option 
  -e,  msgfilter  option 
  -E,  msggrep  option 
  -e,  msggrep  option 
  -e,  ngettext  option 
  -E,  ngettext  option 
  -f,  autopoint  option 
  -f,  gettextize  option 
  -F,  msgattrib  option 
  -f,  msgcat  option 
  -F,  msgcat  option 
  -F,  msgcomm  option 
  -f,  msgcomm  option 
  -F,  msgconv  option 
  -F,  msgen  option 
  -F,  msgfilter  option 
  -f,  msgfilter  option 
  -f,  msgfmt  option 
  -f,  msggrep  option 
  -F,  msggrep  option 
  -F,  msgmerge  option 
  -F,  msguniq  option 
  -F,  xgettext  option 
  -f,  xgettext  option 
  -h,  envsubst  option 
  -h,  gettext  option 
  -h,  msgattrib  option 
  -h,  msgcat  option 
  -h,  msgcmp  option 
  -h,  msgcomm  option 
  -h,  msgconv  option 
  -h,  msgen  option 
  -h,  msgexec  option 
  -h,  msgfilter  option 
  -h,  msgfmt  option 
  -h,  msggrep  option 
  -h,  msginit  option 
  -h,  msgmerge  option 
  -h,  msgunfmt  option 
  -h,  msguniq  option 
  -h,  ngettext  option 
  -h,  xgettext  option 
  -i,  msgattrib  option 
  -i,  msgcat  option 
  -i,  msgcomm  option 
  -i,  msgconv  option 
  -i,  msgen  option 
  -i,  msgexec  option 
  -i,  msgfilter  option 
  -i,  msggrep  option 
  -i,  msginit  option 
  -i,  msgmerge  option 
  -i,  msgunfmt  option 
  -i,  msguniq  option 
  -i,  xgettext  option 
  -j,  msgfmt  option 
  -J,  msggrep  option 
  -j,  msgunfmt  option 
  -j,  xgettext  option 
  -K,  msggrep  option 
  -k,  xgettext  option 
  -l,  msgfmt  option ,  -l,  msgfmt  option ,  -l,  msgfmt  option 
  -l,  msginit  option 
  -l,  msgunfmt  option ,  -l,  msgunfmt  option ,  -l,  msgunfmt  option 
  -L,  xgettext  option 
  -m,  msgcmp  option 
  -M,  msggrep  option 
  -m,  msgmerge  option 
  -M,  xgettext  option 
  -m,  xgettext  option 
  -n,  gettext  option 
  -n,  msgattrib  option 
  -n,  msgcat  option 
  -N,  msgcmp  option 
  -n,  msgcomm  option 
  -n,  msgfilter  option 
  -N,  msggrep  option 
  -N,  msgmerge  option 
  -n,  msguniq  option 
  -n,  xgettext  option 
  -o,  msgattrib  option 
  -o,  msgcat  option 
  -o,  msgcomm  option 
  -o,  msgconv  option 
  -o,  msgen  option 
  -o,  msgfilter  option 
  -o,  msgfmt  option 
  -o,  msggrep  option 
  -o,  msginit  option 
  -o,  msgmerge  option 
  -o,  msgunfmt  option 
  -o,  msguniq  option 
  -o,  xgettext  option 
  -P,  msgattrib  option 
  -p,  msgattrib  option 
  -P,  msgcat  option 
  -p,  msgcat  option 
  -P,  msgcmp  option 
  -P,  msgcomm  option 
  -p,  msgcomm  option 
  -P,  msgconv  option 
  -p,  msgconv  option 
  -P,  msgen  option 
  -p,  msgen  option 
  -P,  msgexec  option 
  -P,  msgfilter  option 
  -p,  msgfilter  option 
  -P,  msgfmt  option 
  -p,  msggrep  option 
  -P,  msggrep  option 
  -P,  msginit  option 
  -p,  msginit  option 
  -p,  msgmerge  option 
  -P,  msgmerge  option 
  -p,  msgunfmt  option 
  -p,  msguniq  option 
  -P,  msguniq  option 
  -p,  xgettext  option 
  -q,  msgmerge  option 
  -r,  msgfmt  option ,  -r,  msgfmt  option 
  -r,  msgunfmt  option ,  -r,  msgunfmt  option 
  -s,  msgattrib  option 
  -s,  msgcat  option 
  -s,  msgcomm  option 
  -s,  msgconv  option 
  -s,  msgen  option 
  -s,  msgfilter  option 
  -s,  msgmerge  option 
  -s,  msgunfmt  option 
  -s,  msguniq  option 
  -s,  xgettext  option 
  -t,  msgcat  option 
  -t,  msgconv  option 
  -T,  msggrep  option 
  -t,  msguniq  option 
  -T,  xgettext  option 
  -u,  msgcat  option 
  -u,  msgcomm  option 
  -U,  msgmerge  option 
  -u,  msguniq  option 
  -v,  envsubst  option 
  -V,  envsubst  option 
  -V,  gettext  option 
  -V,  msgattrib  option 
  -V,  msgcat  option 
  -V,  msgcmp  option 
  -V,  msgcomm  option 
  -V,  msgconv  option 
  -V,  msgen  option 
  -V,  msgexec  option 
  -V,  msgfilter  option 
  -V,  msgfmt  option 
  -v,  msgfmt  option 
  -V,  msggrep  option 
  -v,  msggrep  option 
  -V,  msginit  option 
  -v,  msgmerge  option 
  -V,  msgmerge  option 
  -v,  msgunfmt  option 
  -V,  msgunfmt  option 
  -V,  msguniq  option 
  -V,  ngettext  option 
  -V,  xgettext  option 
  -w,  msgattrib  option 
  -w,  msgcat  option 
  -w,  msgcomm  option 
  -w,  msgconv  option 
  -w,  msgen  option 
  -w,  msgfilter  option 
  -w,  msggrep  option 
  -w,  msginit  option 
  -w,  msgmerge  option 
  -w,  msgunfmt  option 
  -w,  msguniq  option 
  -w,  xgettext  option 
  -X,  msggrep  option 
  -x,  xgettext  option 
 

 
   
Go to the  first ,  previous ,  next ,  last  section,  table of contents .
