# Supplementary material for: rDock: A Fast, Versatile and Open Source Program for Docking Ligands to Proteins and Nucleic Acids
Source: PLoS Comput Biol. 2014 Apr 10;10(4):e1003571. doi: 10.1371/journal.pcbi.1003571 (PMC3983074; doi:10.1371/journal.pcbi.1003571)
Supplement: Software S1 — Compressed file with the source code of the rDock software for ligand docking to Proteins and Nucleic Acids. (GZ) [file pcbi.1003571.s010.gz › rDock_2013.1_src/gettext-0.18.tar.gz!/gettext-0.18/gettext-tools/doc/gettext_16.html]

GNU gettext utilities - 16 Concluding Remarks

Go to the first, previous, next, last section, table of contents.

---

# 16 Concluding Remarks

We would like to conclude this GNU `gettext` manual by presenting
an history of the Translation Project so far. We finally give
a few pointers for those who want to do further research or readings
about Native Language Support matters.

## 16.1 History of GNU `gettext`

Internationalization concerns and algorithms have been informally
and casually discussed for years in GNU, sometimes around GNU
`libc`, maybe around the incoming `Hurd`, or otherwise
(nobody clearly remembers). And even then, when the work started for
real, this was somewhat independently of these previous discussions.

This all began in July 1994, when Patrick D'Cruze had the idea and
initiative of internationalizing version 3.9.2 of GNU `fileutils`.
He then asked Jim Meyering, the maintainer, how to get those changes
folded into an official release. That first draft was full of
`#ifdef`s and somewhat disconcerting, and Jim wanted to find
nicer ways. Patrick and Jim shared some tries and experimentations
in this area. Then, feeling that this might eventually have a deeper
impact on GNU, Jim wanted to know what standards were, and contacted
Richard Stallman, who very quickly and verbally described an overall
design for what was meant to become `glocale`, at that time.

Jim implemented `glocale` and got a lot of exhausting feedback
from Patrick and Richard, of course, but also from Mitchum DSouza
(who wrote a `catgets`-like package), Roland McGrath, maybe David
MacKenzie, François Pinard, and Paul Eggert, all pushing and
pulling in various directions, not always compatible, to the extent
that after a couple of test releases, `glocale` was torn apart.
In particular, Paul Eggert -- always keeping an eye on developments
in Solaris -- advocated the use of the `gettext` API over
`glocale`'s `catgets`-based API.

While Jim took some distance and time and became dad for a second
time, Roland wanted to get GNU `libc` internationalized, and
got Ulrich Drepper involved in that project. Instead of starting
from `glocale`, Ulrich rewrote something from scratch, but
more conforming to the set of guidelines who emerged out of the
`glocale` effort. Then, Ulrich got people from the previous
forum to involve themselves into this new project, and the switch
from `glocale` to what was first named `msgutils`, renamed
`nlsutils`, and later `gettext`, became officially accepted
by Richard in May 1995 or so.

Let's summarize by saying that Ulrich Drepper wrote GNU `gettext`
in April 1995. The first official release of the package, including
PO mode, occurred in July 1995, and was numbered 0.7. Other people
contributed to the effort by providing a discussion forum around
Ulrich, writing little pieces of code, or testing. These are quoted
in the `THANKS` file which comes with the GNU `gettext`
distribution.

While this was being done, François adapted half a dozen of
GNU packages to `glocale` first, then later to `gettext`,
putting them in pretest, so providing along the way an effective
user environment for fine tuning the evolving tools. He also took
the responsibility of organizing and coordinating the Translation
Project. After nearly a year of informal exchanges between people from
many countries, translator teams started to exist in May 1995, through
the creation and support by Patrick D'Cruze of twenty unmoderated
mailing lists for that many native languages, and two moderated
lists: one for reaching all teams at once, the other for reaching
all willing maintainers of internationalized free software packages.

François also wrote PO mode in June 1995 with the collaboration
of Greg McGary, as a kind of contribution to Ulrich's package.
He also gave a hand with the GNU `gettext` Texinfo manual.

In 1997, Ulrich Drepper released the GNU libc 2.0, which included the
`gettext`, `textdomain` and `bindtextdomain` functions.

In 2000, Ulrich Drepper added plural form handling (the `ngettext`
function) to GNU libc. Later, in 2001, he released GNU libc 2.2.x,
which is the first free C library with full internationalization support.

Ulrich being quite busy in his role of General Maintainer of GNU libc,
he handed over the GNU `gettext` maintenance to Bruno Haible in
2000. Bruno added the plural form handling to the tools as well, added
support for UTF-8 and CJK locales, and wrote a few new tools for
manipulating PO files.

## 16.2 Related Readings

**NOTE:**  This documentation section is outdated and needs to be
revised.

Eugene H. Dorr (‘dorre@well.com’) maintains an interesting
bibliography on internationalization matters, called
Internationalization Reference List, which is available as:

```
ftp://ftp.ora.com/pub/examples/nutshell/ujip/doc/i18n-books.txt
```

Michael Gschwind (‘mike@vlsivie.tuwien.ac.at’) maintains a
Frequently Asked Questions (FAQ) list, entitled Programming for
Internationalisation. This FAQ discusses writing programs which
can handle different language conventions, character sets, etc.;
and is applicable to all character set encodings, with particular
emphasis on ISO 8859-1. It is regularly published in Usenet
groups ‘comp.unix.questions’, ‘comp.std.internat’,
‘comp.software.international’, ‘comp.lang.c’,
‘comp.windows.x’, ‘comp.std.c’, ‘comp.answers’
and ‘news.answers’. The home location of this document is:

```
ftp://ftp.vlsivie.tuwien.ac.at/pub/8bit/ISO-programming
```

Patrick D'Cruze (‘pdcruze@li.org’) wrote a tutorial about NLS
matters, and Jochen Hein (‘Hein@student.tu-clausthal.de’) took
over the responsibility of maintaining it. It may be found as:

```
ftp://sunsite.unc.edu/pub/Linux/utils/nls/catalogs/Incoming/...
     ...locale-tutorial-0.8.txt.gz
```

This site is mirrored in:

```
ftp://ftp.ibp.fr/pub/linux/sunsite/
```

A French version of the same tutorial should be findable at:

```
ftp://ftp.ibp.fr/pub/linux/french/docs/
```

together with French translations of many Linux-related documents.

---

Go to the first, previous, next, last section, table of contents.
