# Supplementary material for: rDock: A Fast, Versatile and Open Source Program for Docking Ligands to Proteins and Nucleic Acids
Source: PLoS Comput Biol. 2014 Apr 10;10(4):e1003571. doi: 10.1371/journal.pcbi.1003571 (PMC3983074; doi:10.1371/journal.pcbi.1003571)
Supplement: Software S1 — Compressed file with the source code of the rDock software for ligand docking to Proteins and Nucleic Acids. (GZ) [file pcbi.1003571.s010.gz › rDock_2013.1_src/gettext-0.18.tar.gz!/gettext-0.18/gettext-tools/doc/gettext_toc.html]

GNU gettext utilities - Table of Contents


# GNU gettext tools, version 0.18

## Native Language Support Library and Tools

## Edition 0.18, 25 April 2010

Ulrich Drepper
Jim Meyering
François Pinard
Bruno Haible

---

- 1 Introduction
  - 1.1 The Purpose of GNU `gettext`- 1.2 I18n, L10n, and Such- 1.3 Aspects in Native Language Support- 1.4 Files Conveying Translations- 1.5 Overview of GNU `gettext`
  - 2 The User's View
    - 2.1 Operating System Installation- 2.2 Setting the Locale Used by GUI Programs- 2.3 Setting the Locale through Environment Variables
          - 2.3.1 Locale Names- 2.3.2 Locale Environment Variables- 2.3.3 Specifying a Priority List of Languages
          - 2.4 Installing Translations for Particular Programs
    - 3 The Format of PO Files- 4 Preparing Program Sources
        - 4.1 Importing the `gettext` declaration- 4.2 Triggering `gettext` Operations- 4.3 Preparing Translatable Strings- 4.4 How Marks Appear in Sources- 4.5 Marking Translatable Strings- 4.6 Special Comments preceding Keywords- 4.7 Special Cases of Translatable Strings- 4.8 Letting Users Report Translation Bugs- 4.9 Marking Proper Names for Translation- 4.10 Preparing Library Sources
        - 5 Making the PO Template File
          - 5.1 Invoking the `xgettext` Program
            - 5.1.1 Input file location- 5.1.2 Output file location- 5.1.3 Choice of input file language- 5.1.4 Input file interpretation- 5.1.5 Operation mode- 5.1.6 Language specific options- 5.1.7 Output details- 5.1.8 Informative output
          - 6 Creating a New PO File
            - 6.1 Invoking the `msginit` Program
              - 6.1.1 Input file location- 6.1.2 Output file location- 6.1.3 Input file syntax- 6.1.4 Output details- 6.1.5 Informative output
              - 6.2 Filling in the Header Entry
            - 7 Updating Existing PO Files
              - 7.1 Invoking the `msgmerge` Program
                - 7.1.1 Input file location- 7.1.2 Operation mode- 7.1.3 Output file location- 7.1.4 Output file location in update mode- 7.1.5 Operation modifiers- 7.1.6 Input file syntax- 7.1.7 Output details- 7.1.8 Informative output
              - 8 Editing PO Files
                - 8.1 KDE's PO File Editor- 8.2 GNOME's PO File Editor- 8.3 Emacs's PO File Editor
                      - 8.3.1 Completing GNU `gettext` Installation- 8.3.2 Main PO mode Commands- 8.3.3 Entry Positioning- 8.3.4 Normalizing Strings in Entries- 8.3.5 Translated Entries- 8.3.6 Fuzzy Entries- 8.3.7 Untranslated Entries- 8.3.8 Obsolete Entries- 8.3.9 Modifying Translations- 8.3.10 Modifying Comments- 8.3.11 Details of Sub Edition- 8.3.12 C Sources Context- 8.3.13 Consulting Auxiliary PO Files
                      - 8.4 Using Translation Compendia
                        - 8.4.1 Creating Compendia
                          - 8.4.1.1 Concatenate PO Files- 8.4.1.2 Extract a Message Subset from a PO File
                          - 8.4.2 Using Compendia
                            - 8.4.2.1 Initialize a New Translation File- 8.4.2.2 Update an Existing Translation File
                - 9 Manipulating PO Files
                  - 9.1 Invoking the `msgcat` Program
                    - 9.1.1 Input file location- 9.1.2 Output file location- 9.1.3 Message selection- 9.1.4 Input file syntax- 9.1.5 Output details- 9.1.6 Informative output
                    - 9.2 Invoking the `msgconv` Program
                      - 9.2.1 Input file location- 9.2.2 Output file location- 9.2.3 Conversion target- 9.2.4 Input file syntax- 9.2.5 Output details- 9.2.6 Informative output
                      - 9.3 Invoking the `msggrep` Program
                        - 9.3.1 Input file location- 9.3.2 Output file location- 9.3.3 Message selection- 9.3.4 Input file syntax- 9.3.5 Output details- 9.3.6 Informative output- 9.3.7 Examples
                        - 9.4 Invoking the `msgfilter` Program
                          - 9.4.1 Input file location- 9.4.2 Output file location- 9.4.3 The filter- 9.4.4 Useful filter-options when the filter is `‘sed’`- 9.4.5 Built-in filters- 9.4.6 Input file syntax- 9.4.7 Output details- 9.4.8 Informative output- 9.4.9 Examples
                          - 9.5 Invoking the `msguniq` Program
                            - 9.5.1 Input file location- 9.5.2 Output file location- 9.5.3 Message selection- 9.5.4 Input file syntax- 9.5.5 Output details- 9.5.6 Informative output
                            - 9.6 Invoking the `msgcomm` Program
                              - 9.6.1 Input file location- 9.6.2 Output file location- 9.6.3 Message selection- 9.6.4 Input file syntax- 9.6.5 Output details- 9.6.6 Informative output
                              - 9.7 Invoking the `msgcmp` Program
                                - 9.7.1 Input file location- 9.7.2 Operation modifiers- 9.7.3 Input file syntax- 9.7.4 Informative output
                                - 9.8 Invoking the `msgattrib` Program
                                  - 9.8.1 Input file location- 9.8.2 Output file location- 9.8.3 Message selection- 9.8.4 Attribute manipulation- 9.8.5 Input file syntax- 9.8.6 Output details- 9.8.7 Informative output
                                  - 9.9 Invoking the `msgen` Program
                                    - 9.9.1 Input file location- 9.9.2 Output file location- 9.9.3 Input file syntax- 9.9.4 Output details- 9.9.5 Informative output
                                    - 9.10 Invoking the `msgexec` Program
                                      - 9.10.1 Input file location- 9.10.2 Input file syntax- 9.10.3 Informative output
                                      - 9.11 Highlighting parts of PO files
                                        - 9.11.1 The `--color` option- 9.11.2 The environment variable `TERM`- 9.11.3 The `--style` option- 9.11.4 Style rules for PO files- 9.11.5 Customizing `less` for viewing PO files
                                        - 9.12 Writing your own programs that process PO files
                  - 10 Producing Binary MO Files
                    - 10.1 Invoking the `msgfmt` Program
                      - 10.1.1 Input file location- 10.1.2 Operation mode- 10.1.3 Output file location- 10.1.4 Output file location in Java mode- 10.1.5 Output file location in C# mode- 10.1.6 Output file location in Tcl mode- 10.1.7 Input file syntax- 10.1.8 Input file interpretation- 10.1.9 Output details- 10.1.10 Informative output
                      - 10.2 Invoking the `msgunfmt` Program
                        - 10.2.1 Operation mode- 10.2.2 Input file location- 10.2.3 Input file location in Java mode- 10.2.4 Input file location in C# mode- 10.2.5 Input file location in Tcl mode- 10.2.6 Output file location- 10.2.7 Output details- 10.2.8 Informative output
                        - 10.3 The Format of GNU MO Files
                    - 11 The Programmer's View
                      - 11.1 About `catgets`
                        - 11.1.1 The Interface- 11.1.2 Problems with the `catgets` Interface?!
                        - 11.2 About `gettext`
                          - 11.2.1 The Interface- 11.2.2 Solving Ambiguities- 11.2.3 Locating Message Catalog Files- 11.2.4 How to specify the output character set `gettext` uses- 11.2.5 Using contexts for solving ambiguities- 11.2.6 Additional functions for plural forms- 11.2.7 Optimization of the \*gettext functions
                          - 11.3 Comparing the Two Interfaces- 11.4 Using libintl.a in own programs- 11.5 Being a `gettext` grok- 11.6 Temporary Notes for the Programmers Chapter
                                  - 11.6.1 Temporary - Two Possible Implementations- 11.6.2 Temporary - About `catgets`- 11.6.3 Temporary - Why a single implementation- 11.6.4 Temporary - Notes
                      - 12 The Translator's View
                        - 12.1 Introduction 0- 12.2 Introduction 1- 12.3 Discussions- 12.4 Organization
                                - 12.4.1 Central Coordination- 12.4.2 National Teams
                                    - 12.4.2.1 Sub-Cultures- 12.4.2.2 Organizational Ideas
                                    - 12.4.3 Mailing Lists
                                - 12.5 Information Flow- 12.6 Translating plural forms- 12.7 Prioritizing messages: How to determine which messages to translate first
                        - 13 The Maintainer's View
                          - 13.1 Flat or Non-Flat Directory Structures- 13.2 Prerequisite Works- 13.3 Invoking the `gettextize` Program- 13.4 Files You Must Create or Alter
                                  - 13.4.1 ‘POTFILES.in’ in ‘po/’- 13.4.2 ‘LINGUAS’ in ‘po/’- 13.4.3 ‘Makevars’ in ‘po/’- 13.4.4 Extending ‘Makefile’ in ‘po/’- 13.4.5 ‘configure.ac’ at top level- 13.4.6 ‘config.guess’, ‘config.sub’ at top level- 13.4.7 ‘mkinstalldirs’ at top level- 13.4.8 ‘aclocal.m4’ at top level- 13.4.9 ‘acconfig.h’ at top level- 13.4.10 ‘config.h.in’ at top level- 13.4.11 ‘Makefile.in’ at top level- 13.4.12 ‘Makefile.in’ in ‘src/’- 13.4.13 ‘gettext.h’ in ‘lib/’
                                  - 13.5 Autoconf macros for use in ‘configure.ac’
                                    - 13.5.1 AM\_GNU\_GETTEXT in ‘gettext.m4’- 13.5.2 AM\_GNU\_GETTEXT\_VERSION in ‘gettext.m4’- 13.5.3 AM\_GNU\_GETTEXT\_NEED in ‘gettext.m4’- 13.5.4 AM\_GNU\_GETTEXT\_INTL\_SUBDIR in ‘intldir.m4’- 13.5.5 AM\_PO\_SUBDIRS in ‘po.m4’- 13.5.6 AM\_XGETTEXT\_OPTION in ‘po.m4’- 13.5.7 AM\_ICONV in ‘iconv.m4’
                                    - 13.6 Integrating with CVS
                                      - 13.6.1 Avoiding version mismatch in distributed development- 13.6.2 Files to put under CVS version control- 13.6.3 Invoking the `autopoint` Program
                                            - 13.6.3.1 Options- 13.6.3.2 Informative output
                                      - 13.7 Creating a Distribution Tarball
                          - 14 The Installer's and Distributor's View- 15 Other Programming Languages
                              - 15.1 The Language Implementor's View- 15.2 The Programmer's View- 15.3 The Translator's View
                                    - 15.3.1 C Format Strings- 15.3.2 Objective C Format Strings- 15.3.3 Shell Format Strings- 15.3.4 Python Format Strings- 15.3.5 Lisp Format Strings- 15.3.6 Emacs Lisp Format Strings- 15.3.7 librep Format Strings- 15.3.8 Scheme Format Strings- 15.3.9 Smalltalk Format Strings- 15.3.10 Java Format Strings- 15.3.11 C# Format Strings- 15.3.12 awk Format Strings- 15.3.13 Object Pascal Format Strings- 15.3.14 YCP Format Strings- 15.3.15 Tcl Format Strings- 15.3.16 Perl Format Strings- 15.3.17 PHP Format Strings- 15.3.18 GCC internal Format Strings- 15.3.19 GFC internal Format Strings- 15.3.20 Qt Format Strings- 15.3.21 Qt Format Strings- 15.3.22 KDE Format Strings- 15.3.23 Boost Format Strings
                                    - 15.4 The Maintainer's View- 15.5 Individual Programming Languages
                                        - 15.5.1 C, C++, Objective C- 15.5.2 sh - Shell Script
                                            - 15.5.2.1 Preparing Shell Scripts for Internationalization- 15.5.2.2 Contents of `gettext.sh`- 15.5.2.3 Invoking the `gettext` program- 15.5.2.4 Invoking the `ngettext` program- 15.5.2.5 Invoking the `envsubst` program- 15.5.2.6 Invoking the `eval_gettext` function- 15.5.2.7 Invoking the `eval_ngettext` function
                                            - 15.5.3 bash - Bourne-Again Shell Script- 15.5.4 Python- 15.5.5 GNU clisp - Common Lisp- 15.5.6 GNU clisp C sources- 15.5.7 Emacs Lisp- 15.5.8 librep- 15.5.9 GNU guile - Scheme- 15.5.10 GNU Smalltalk- 15.5.11 Java- 15.5.12 C#- 15.5.13 GNU awk- 15.5.14 Pascal - Free Pascal Compiler- 15.5.15 wxWidgets library- 15.5.16 YCP - YaST2 scripting language- 15.5.17 Tcl - Tk's scripting language- 15.5.18 Perl
                                                                            - 15.5.18.1 General Problems Parsing Perl Code- 15.5.18.2 Which keywords will xgettext look for?- 15.5.18.3 How to Extract Hash Keys- 15.5.18.4 What are Strings And Quote-like Expressions?- 15.5.18.5 Invalid Uses Of String Interpolation- 15.5.18.6 Valid Uses Of String Interpolation- 15.5.18.7 When To Use Parentheses- 15.5.18.8 How To Grok with Long Lines- 15.5.18.9 Bugs, Pitfalls, And Things That Do Not Work
                                                                            - 15.5.19 PHP Hypertext Preprocessor- 15.5.20 Pike- 15.5.21 GNU Compiler Collection sources
                                        - 15.6 Internationalizable Data
                                          - 15.6.1 POT - Portable Object Template- 15.6.2 Resource String Table- 15.6.3 Glade - GNOME user interface description
                              - 16 Concluding Remarks
                                - 16.1 History of GNU `gettext`- 16.2 Related Readings
                                - A Language Codes
                                  - A.1 Usual Language Codes- A.2 Rare Language Codes
                                  - B Country Codes- C Licenses
                                      - C.1 GNU GENERAL PUBLIC LICENSE
                                        - Preamble- Appendix: How to Apply These Terms to Your New Programs
                                        - C.2 GNU LESSER GENERAL PUBLIC LICENSE
                                          - Preamble- How to Apply These Terms to Your New Libraries
                                          - C.3 GNU Free Documentation License
                                            - ADDENDUM: How to use this License for your documents
                                      - Program Index- Option Index- Variable Index- PO Mode Index- Autoconf Macro Index- General Index

---

This document was generated on 9 May 2010 using the
texi2html
translator version 1.52b.
