# Supplementary material for: rDock: A Fast, Versatile and Open Source Program for Docking Ligands to Proteins and Nucleic Acids
Source: PLoS Comput Biol. 2014 Apr 10;10(4):e1003571. doi: 10.1371/journal.pcbi.1003571 (PMC3983074; doi:10.1371/journal.pcbi.1003571)
Supplement: Software S1 — Compressed file with the source code of the rDock software for ligand docking to Proteins and Nucleic Acids. (GZ) [file pcbi.1003571.s010.gz › rDock_2013.1_src/gettext-0.18.tar.gz!/gettext-0.18/gettext-tools/doc/gettext_10.html]

GNU gettext utilities - 10 Producing Binary MO Files

Go to the first, previous, next, last section, table of contents.

---

# 10 Producing Binary MO Files

## 10.1 Invoking the `msgfmt` Program

```
msgfmt [option] filename.po ...
```

The `msgfmt` programs generates a binary message catalog from a textual
translation description.

### 10.1.1 Input file location

`‘filename.po ...’`: `‘-D directory’`: `‘--directory=directory’`: Add directory to the list of directories. Source files are searched relative to this list of directories. The resulting ‘.po’ file will be written relative to the current directory, though.

If an input file is `‘-’`, standard input is read.

### 10.1.2 Operation mode

`‘-j’`: `‘--java’`: Java mode: generate a Java `ResourceBundle` class. `‘--java2’`: Like --java, and assume Java2 (JDK 1.2 or higher). `‘--csharp’`: C# mode: generate a .NET .dll file containing a subclass of `GettextResourceSet`. `‘--csharp-resources’`: C# resources mode: generate a .NET ‘.resources’ file. `‘--tcl’`: Tcl mode: generate a tcl/msgcat ‘.msg’ file. `‘--qt’`: Qt mode: generate a Qt ‘.qm’ file.

### 10.1.3 Output file location

`‘-o file’`: `‘--output-file=file’`: Write output to specified file. `‘--strict’`: Direct the program to work strictly following the Uniforum/Sun implementation. Currently this only affects the naming of the output file. If this option is not given the name of the output file is the same as the domain name. If the strict Uniforum mode is enabled the suffix ‘.mo’ is added to the file name if it is not already present. We find this behaviour of Sun's implementation rather silly and so by default this mode is *not* selected.

If the output file is `‘-’`, output is written to standard output.

### 10.1.4 Output file location in Java mode

`‘-r resource’`: `‘--resource=resource’`: Specify the resource name. `‘-l locale’`: `‘--locale=locale’`: Specify the locale name, either a language specification of the form ll or a combined language and country specification of the form ll\_CC. `‘-d directory’`: Specify the base directory of classes directory hierarchy.

The class name is determined by appending the locale name to the resource name,
separated with an underscore. The `‘-d’` option is mandatory. The class
is written under the specified directory.

### 10.1.5 Output file location in C# mode

`‘-r resource’`: `‘--resource=resource’`: Specify the resource name. `‘-l locale’`: `‘--locale=locale’`: Specify the locale name, either a language specification of the form ll or a combined language and country specification of the form ll\_CC. `‘-d directory’`: Specify the base directory for locale dependent ‘.dll’ files.

The `‘-l’` and `‘-d’` options are mandatory. The ‘.dll’ file is
written in a subdirectory of the specified directory whose name depends on the
locale.

### 10.1.6 Output file location in Tcl mode

`‘-l locale’`: `‘--locale=locale’`: Specify the locale name, either a language specification of the form ll or a combined language and country specification of the form ll\_CC. `‘-d directory’`: Specify the base directory of ‘.msg’ message catalogs.

The `‘-l’` and `‘-d’` options are mandatory. The ‘.msg’ file is
written in the specified directory.

### 10.1.7 Input file syntax

`‘-P’`: `‘--properties-input’`: Assume the input files are Java ResourceBundles in Java `.properties` syntax, not in PO file syntax. `‘--stringtable-input’`: Assume the input files are NeXTstep/GNUstep localized resource files in `.strings` syntax, not in PO file syntax.

### 10.1.8 Input file interpretation

`‘-c’`: `‘--check’`: Perform all the checks implied by `--check-format`, `--check-header`, `--check-domain`. `‘--check-format’`: Check language dependent format strings. If the string represents a format string used in a `printf`-like function both strings should have the same number of `‘%’` format specifiers, with matching types. If the flag `c-format` or `possible-c-format` appears in the special comment `#,` for this entry a check is performed. For example, the check will diagnose using `‘%.*s’` against `‘%s’`, or `‘%d’` against `‘%s’`, or `‘%d’` against `‘%x’`. It can even handle positional parameters. Normally the `xgettext` program automatically decides whether a string is a format string or not. This algorithm is not perfect, though. It might regard a string as a format string though it is not used in a `printf`-like function and so `msgfmt` might report errors where there are none. To solve this problem the programmer can dictate the decision to the `xgettext` program (see section 15.3.1 C Format Strings). The translator should not consider removing the flag from the `#,` line. This "fix" would be reversed again as soon as `msgmerge` is called the next time. `‘--check-header’`: Verify presence and contents of the header entry. See section 6.2 Filling in the Header Entry, for a description of the various fields in the header entry. `‘--check-domain’`: Check for conflicts between domain directives and the `--output-file` option `‘-C’`: `‘--check-compatibility’`: Check that GNU msgfmt behaves like X/Open msgfmt. This will give an error when attempting to use the GNU extensions. `‘--check-accelerators[=char]’`: Check presence of keyboard accelerators for menu items. This is based on the convention used in some GUIs that a keyboard accelerator in a menu item string is designated by an immediately preceding `‘&’` character. Sometimes a keyboard accelerator is also called "keyboard mnemonic". This check verifies that if the untranslated string has exactly one `‘&’` character, the translated string has exactly one `‘&’` as well. If this option is given with a char argument, this char should be a non-alphanumeric character and is used as keyboard accelerator mark instead of `‘&’`. `‘-f’`: `‘--use-fuzzy’`: Use fuzzy entries in output. Note that using this option is usually wrong, because fuzzy messages are exactly those which have not been validated by a human translator.

### 10.1.9 Output details

`‘-a number’`: `‘--alignment=number’`: Align strings to number bytes (default: 1). `‘--no-hash’`: Don't include a hash table in the binary file. Lookup will be more expensive at run time (binary search instead of hash table lookup).

### 10.1.10 Informative output

`‘-h’`: `‘--help’`: Display this help and exit. `‘-V’`: `‘--version’`: Output version information and exit. `‘--statistics’`: Print statistics about translations. When the option `--verbose` is used in combination with `--statistics`, the input file name is printed in front of the statistics line. `‘-v’`: `‘--verbose’`: Increase verbosity level.

## 10.2 Invoking the `msgunfmt` Program

```
msgunfmt [option] [file]...
```

The `msgunfmt` program converts a binary message catalog to a
Uniforum style .po file.

### 10.2.1 Operation mode

`‘-j’`: `‘--java’`: Java mode: input is a Java `ResourceBundle` class. `‘--csharp’`: C# mode: input is a .NET .dll file containing a subclass of `GettextResourceSet`. `‘--csharp-resources’`: C# resources mode: input is a .NET ‘.resources’ file. `‘--tcl’`: Tcl mode: input is a tcl/msgcat ‘.msg’ file.

### 10.2.2 Input file location

`‘file ...’`: Input .mo files.

If no input file is given or if it is `‘-’`, standard input is read.

### 10.2.3 Input file location in Java mode

`‘-r resource’`: `‘--resource=resource’`: Specify the resource name. `‘-l locale’`: `‘--locale=locale’`: Specify the locale name, either a language specification of the form ll or a combined language and country specification of the form ll\_CC.

The class name is determined by appending the locale name to the resource name,
separated with an underscore. The class is located using the `CLASSPATH`.

### 10.2.4 Input file location in C# mode

`‘-r resource’`: `‘--resource=resource’`: Specify the resource name. `‘-l locale’`: `‘--locale=locale’`: Specify the locale name, either a language specification of the form ll or a combined language and country specification of the form ll\_CC. `‘-d directory’`: Specify the base directory for locale dependent ‘.dll’ files.

The `‘-l’` and `‘-d’` options are mandatory. The ‘.msg’ file is
located in a subdirectory of the specified directory whose name depends on the
locale.

### 10.2.5 Input file location in Tcl mode

`‘-l locale’`: `‘--locale=locale’`: Specify the locale name, either a language specification of the form ll or a combined language and country specification of the form ll\_CC. `‘-d directory’`: Specify the base directory of ‘.msg’ message catalogs.

The `‘-l’` and `‘-d’` options are mandatory. The ‘.msg’ file is
located in the specified directory.

### 10.2.6 Output file location

`‘-o file’`: `‘--output-file=file’`: Write output to specified file.

The results are written to standard output if no output file is specified
or if it is `‘-’`.

### 10.2.7 Output details

`‘--color’`: `‘--color=when’`: Specify whether or when to use colors and other text attributes. See section 9.11.1 The `--color` option for details. `‘--style=style_file’`: Specify the CSS style rule file to use for `--color`. See section 9.11.3 The `--style` option for details. `‘--force-po’`: Always write an output file even if it contains no message. `‘-i’`: `‘--indent’`: Write the .po file using indented style. `‘--strict’`: Write out a strict Uniforum conforming PO file. Note that this Uniforum format should be avoided because it doesn't support the GNU extensions. `‘-p’`: `‘--properties-output’`: Write out a Java ResourceBundle in Java `.properties` syntax. Note that this file format doesn't support plural forms and silently drops obsolete messages. `‘--stringtable-output’`: Write out a NeXTstep/GNUstep localized resource file in `.strings` syntax. Note that this file format doesn't support plural forms. `‘-w number’`: `‘--width=number’`: Set the output page width. Long strings in the output files will be split across multiple lines in order to ensure that each line's width (= number of screen columns) is less or equal to the given number. `‘--no-wrap’`: Do not break long message lines. Message lines whose width exceeds the output page width will not be split into several lines. Only file reference lines which are wider than the output page width will be split. `‘-s’`: `‘--sort-output’`: Generate sorted output. Note that using this option makes it much harder for the translator to understand each message's context.

### 10.2.8 Informative output

`‘-h’`: `‘--help’`: Display this help and exit. `‘-V’`: `‘--version’`: Output version information and exit. `‘-v’`: `‘--verbose’`: Increase verbosity level.

## 10.3 The Format of GNU MO Files

The format of the generated MO files is best described by a picture,
which appears below.

The first two words serve the identification of the file. The magic
number will always signal GNU MO files. The number is stored in the
byte order of the generating machine, so the magic number really is
two numbers: `0x950412de` and `0xde120495`.

The second word describes the current revision of the file format,
composed of a major and a minor revision number. The revision numbers
ensure that the readers of MO files can distinguish new formats from
old ones and handle their contents, as far as possible. For now the
major revision is 0 or 1, and the minor revision is also 0 or 1. More
revisions might be added in the future. A program seeing an unexpected
major revision number should stop reading the MO file entirely; whereas
an unexpected minor revision number means that the file can be read but
will not reveal its full contents, when parsed by a program that
supports only smaller minor revision numbers.

The version is kept
separate from the magic number, instead of using different magic
numbers for different formats, mainly because ‘/etc/magic’ is
not updated often.

Follow a number of pointers to later tables in the file, allowing
for the extension of the prefix part of MO files without having to
recompile programs reading them. This might become useful for later
inserting a few flag bits, indication about the charset used, new
tables, or other things.

Then, at offset O and offset T in the picture, two tables
of string descriptors can be found. In both tables, each string
descriptor uses two 32 bits integers, one for the string length,
another for the offset of the string in the MO file, counting in bytes
from the start of the file. The first table contains descriptors
for the original strings, and is sorted so the original strings
are in increasing lexicographical order. The second table contains
descriptors for the translated strings, and is parallel to the first
table: to find the corresponding translation one has to access the
array slot in the second array with the same index.

Having the original strings sorted enables the use of simple binary
search, for when the MO file does not contain an hashing table, or
for when it is not practical to use the hashing table provided in
the MO file. This also has another advantage, as the empty string
in a PO file GNU `gettext` is usually *translated* into
some system information attached to that particular MO file, and the
empty string necessarily becomes the first in both the original and
translated tables, making the system information very easy to find.

The size S of the hash table can be zero. In this case, the
hash table itself is not contained in the MO file. Some people might
prefer this because a precomputed hashing table takes disk space, and
does not win *that* much speed. The hash table contains indices
to the sorted array of strings in the MO file. Conflict resolution is
done by double hashing. The precise hashing algorithm used is fairly
dependent on GNU `gettext` code, and is not documented here.

As for the strings themselves, they follow the hash file, and each
is terminated with a `NUL`, and this `NUL` is not counted in
the length which appears in the string descriptor. The `msgfmt`
program has an option selecting the alignment for MO file strings.
With this option, each string is separately aligned so it starts at
an offset which is a multiple of the alignment value. On some RISC
machines, a correct alignment will speed things up.

Contexts are stored by storing the concatenation of the context, a
`EOT` byte, and the original string, instead of the original string.

Plural forms are stored by letting the plural of the original string
follow the singular of the original string, separated through a
`NUL` byte. The length which appears in the string descriptor
includes both. However, only the singular of the original string
takes part in the hash table lookup. The plural variants of the
translation are all stored consecutively, separated through a
`NUL` byte. Here also, the length in the string descriptor
includes all of them.

Nothing prevents a MO file from having embedded `NUL`s in strings.
However, the program interface currently used already presumes
that strings are `NUL` terminated, so embedded `NUL`s are
somewhat useless. But the MO file format is general enough so other
interfaces would be later possible, if for example, we ever want to
implement wide characters right in MO files, where `NUL` bytes may
accidentally appear. (No, we don't want to have wide characters in MO
files. They would make the file unnecessarily large, and the
`‘wchar_t’` type being platform dependent, MO files would be
platform dependent as well.)

This particular issue has been strongly debated in the GNU
`gettext` development forum, and it is expectable that MO file
format will evolve or change over time. It is even possible that many
formats may later be supported concurrently. But surely, we have to
start somewhere, and the MO file format described here is a good start.
Nothing is cast in concrete, and the format may later evolve fairly
easily, so we should feel comfortable with the current approach.

```
        byte
             +------------------------------------------+
          0  | magic number = 0x950412de                |
             |                                          |
          4  | file format revision = 0                 |
             |                                          |
          8  | number of strings                        |  == N
             |                                          |
         12  | offset of table with original strings    |  == O
             |                                          |
         16  | offset of table with translation strings |  == T
             |                                          |
         20  | size of hashing table                    |  == S
             |                                          |
         24  | offset of hashing table                  |  == H
             |                                          |
             .                                          .
             .    (possibly more entries later)         .
             .                                          .
             |                                          |
          O  | length & offset 0th string  ----------------.
      O + 8  | length & offset 1st string  ------------------.
              ...                                    ...   | |
O + ((N-1)*8)| length & offset (N-1)th string           |  | |
             |                                          |  | |
          T  | length & offset 0th translation  ---------------.
      T + 8  | length & offset 1st translation  -----------------.
              ...                                    ...   | | | |
T + ((N-1)*8)| length & offset (N-1)th translation      |  | | | |
             |                                          |  | | | |
          H  | start hash table                         |  | | | |
              ...                                    ...   | | | |
  H + S * 4  | end hash table                           |  | | | |
             |                                          |  | | | |
             | NUL terminated 0th string  <----------------' | | |
             |                                          |    | | |
             | NUL terminated 1st string  <------------------' | |
             |                                          |      | |
              ...                                    ...       | |
             |                                          |      | |
             | NUL terminated 0th translation  <---------------' |
             |                                          |        |
             | NUL terminated 1st translation  <-----------------'
             |                                          |
              ...                                    ...
             |                                          |
             +------------------------------------------+
```

---

Go to the first, previous, next, last section, table of contents.
