# Supplementary material for: rDock: A Fast, Versatile and Open Source Program for Docking Ligands to Proteins and Nucleic Acids
Source: PLoS Comput Biol. 2014 Apr 10;10(4):e1003571. doi: 10.1371/journal.pcbi.1003571 (PMC3983074; doi:10.1371/journal.pcbi.1003571)
Supplement: Software S1 — Compressed file with the source code of the rDock software for ligand docking to Proteins and Nucleic Acids. (GZ) [file pcbi.1003571.s010.gz › rDock_2013.1_src/gettext-0.18.tar.gz!/gettext-0.18/gettext-tools/doc/gettext_22.html]

GNU gettext utilities - Variable Index

Go to the first, previous, next, last section, table of contents.

---

# Variable Index

Jump to:
g
-
l
-
m
-
p
-
t

## g

- GETTEXT\_LOG\_UNTRANSLATED, environment variable

## l

- LANG, environment variable, LANG, environment variable- LANGUAGE, environment variable, LANGUAGE, environment variable, LANGUAGE, environment variable- LC\_ALL, environment variable, LC\_ALL, environment variable- LC\_COLLATE, environment variable, LC\_COLLATE, environment variable- LC\_CTYPE, environment variable, LC\_CTYPE, environment variable- LC\_MESSAGES, environment variable, LC\_MESSAGES, environment variable- LC\_MONETARY, environment variable, LC\_MONETARY, environment variable- LC\_NUMERIC, environment variable, LC\_NUMERIC, environment variable- LC\_TIME, environment variable, LC\_TIME, environment variable- LINGUAS, environment variable

## m

- MSGEXEC\_LOCATION, environment variable- MSGEXEC\_MSGCTXT, environment variable- MSGEXEC\_MSGID, environment variable- MSGFILTER\_LOCATION, environment variable- MSGFILTER\_MSGCTXT, environment variable- MSGFILTER\_MSGID, environment variable

## p

- PO\_STYLE, environment variable

## t

- TERM, environment variable- TEXTDOMAIN, environment variable- TEXTDOMAINDIR, environment variable

---

Go to the first, previous, next, last section, table of contents.
