# Supplementary material for: rDock: A Fast, Versatile and Open Source Program for Docking Ligands to Proteins and Nucleic Acids
Source: PLoS Comput Biol. 2014 Apr 10;10(4):e1003571. doi: 10.1371/journal.pcbi.1003571 (PMC3983074; doi:10.1371/journal.pcbi.1003571)
Supplement: Software S1 — Compressed file with the source code of the rDock software for ligand docking to Proteins and Nucleic Acids. (GZ) [file pcbi.1003571.s010.gz › rDock_2013.1_src/gettext-0.18.tar.gz!/gettext-0.18/gettext-tools/doc/gettext_7.html]

GNU gettext utilities - 7 Updating Existing PO Files

Go to the first, previous, next, last section, table of contents.

---

# 7 Updating Existing PO Files

## 7.1 Invoking the `msgmerge` Program

```
msgmerge [option] def.po ref.pot
```

The `msgmerge` program merges two Uniforum style .po files together.
The def.po file is an existing PO file with translations which will
be taken over to the newly created file as long as they still match;
comments will be preserved, but extracted comments and file positions will
be discarded. The ref.pot file is the last created PO file with
up-to-date source references but old translations, or a PO Template file
(generally created by `xgettext`); any translations or comments
in the file will be discarded, however dot comments and file positions
will be preserved. Where an exact match cannot be found, fuzzy matching
is used to produce better results.

### 7.1.1 Input file location

`‘def.po’`: Translations referring to old sources. `‘ref.pot’`: References to the new sources. `‘-D directory’`: `‘--directory=directory’`: Add directory to the list of directories. Source files are searched relative to this list of directories. The resulting ‘.po’ file will be written relative to the current directory, though. `‘-C file’`: `‘--compendium=file’`: Specify an additional library of message translations. See section 8.4 Using Translation Compendia. This option may be specified more than once.

### 7.1.2 Operation mode

`‘-U’`: `‘--update’`: Update def.po. Do nothing if def.po is already up to date.

### 7.1.3 Output file location

`‘-o file’`: `‘--output-file=file’`: Write output to specified file.

The results are written to standard output if no output file is specified
or if it is `‘-’`.

### 7.1.4 Output file location in update mode

The result is written back to def.po.

`‘--backup=control’`: Make a backup of def.po `‘--suffix=suffix’`: Override the usual backup suffix.

The version control method may be selected via the `--backup` option
or through the `VERSION_CONTROL` environment variable. Here are the
values:

`‘none’`: `‘off’`: Never make backups (even if `--backup` is given). `‘numbered’`: `‘t’`: Make numbered backups. `‘existing’`: `‘nil’`: Make numbered backups if numbered backups for this file already exist, otherwise make simple backups. `‘simple’`: `‘never’`: Always make simple backups.

The backup suffix is `‘~’`, unless set with `--suffix` or the
`SIMPLE_BACKUP_SUFFIX` environment variable.

### 7.1.5 Operation modifiers

`‘-m’`: `‘--multi-domain’`: Apply ref.pot to each of the domains in def.po. `‘-N’`: `‘--no-fuzzy-matching’`: Do not use fuzzy matching when an exact match is not found. This may speed up the operation considerably. `‘--previous’`: Keep the previous msgids of translated messages, marked with `‘#|’`, when adding the fuzzy marker to such messages.

### 7.1.6 Input file syntax

`‘-P’`: `‘--properties-input’`: Assume the input files are Java ResourceBundles in Java `.properties` syntax, not in PO file syntax. `‘--stringtable-input’`: Assume the input files are NeXTstep/GNUstep localized resource files in `.strings` syntax, not in PO file syntax.

### 7.1.7 Output details

`‘--lang=catalogname’`: Specify the `‘Language’` field to be used in the header entry. See section 6.2 Filling in the Header Entry for the meaning of this field. Note: The `‘Language-Team’` and `‘Plural-Forms’` fields are left unchanged. If this option is not specified, the `‘Language’` field is inferred, as best as possible, from the `‘Language-Team’` field. `‘--color’`: `‘--color=when’`: Specify whether or when to use colors and other text attributes. See section 9.11.1 The `--color` option for details. `‘--style=style_file’`: Specify the CSS style rule file to use for `--color`. See section 9.11.3 The `--style` option for details. `‘--force-po’`: Always write an output file even if it contains no message. `‘-i’`: `‘--indent’`: Write the .po file using indented style. `‘--no-location’`: Do not write `‘#: filename:line’` lines. `‘--add-location’`: Generate `‘#: filename:line’` lines (default). `‘--strict’`: Write out a strict Uniforum conforming PO file. Note that this Uniforum format should be avoided because it doesn't support the GNU extensions. `‘-p’`: `‘--properties-output’`: Write out a Java ResourceBundle in Java `.properties` syntax. Note that this file format doesn't support plural forms and silently drops obsolete messages. `‘--stringtable-output’`: Write out a NeXTstep/GNUstep localized resource file in `.strings` syntax. Note that this file format doesn't support plural forms. `‘-w number’`: `‘--width=number’`: Set the output page width. Long strings in the output files will be split across multiple lines in order to ensure that each line's width (= number of screen columns) is less or equal to the given number. `‘--no-wrap’`: Do not break long message lines. Message lines whose width exceeds the output page width will not be split into several lines. Only file reference lines which are wider than the output page width will be split. `‘-s’`: `‘--sort-output’`: Generate sorted output. Note that using this option makes it much harder for the translator to understand each message's context. `‘-F’`: `‘--sort-by-file’`: Sort output by file location.

### 7.1.8 Informative output

`‘-h’`: `‘--help’`: Display this help and exit. `‘-V’`: `‘--version’`: Output version information and exit. `‘-v’`: `‘--verbose’`: Increase verbosity level. `‘-q’`: `‘--quiet’`: `‘--silent’`: Suppress progress indicators.

---

Go to the first, previous, next, last section, table of contents.
