# Supplementary material for: rDock: A Fast, Versatile and Open Source Program for Docking Ligands to Proteins and Nucleic Acids
Source: PLoS Comput Biol. 2014 Apr 10;10(4):e1003571. doi: 10.1371/journal.pcbi.1003571 (PMC3983074; doi:10.1371/journal.pcbi.1003571)
Supplement: Software S1 — Compressed file with the source code of the rDock software for ligand docking to Proteins and Nucleic Acids. (GZ) [file pcbi.1003571.s010.gz › rDock_2013.1_src/data/masses.rtf]

* ...

* ...

* Copyright (c) 1986, 1987, 1988, 1989, 1990, 1991, 1992

* Molecular Simulations Incorporated

* Confidential and Proprietary: All Rights Reserved

* ...

* ....

* TEMPLATE FOR CREATING CHARMm RTF file

* ....

*	(%_m) replaced by first 4 characters of molecule name

*	in QUANTA (if used)

* ..

* Build RTF for %m

* ...

* sorted alphabetically; metals follow organics

* Atom type numbers 201-229 are reserved for special QUANTA display atom types.

* Last current active number is 234.

*

22	0

MASS	7 B	10.81	B ! Boron

MASS	14 C	12.01100 C ! Carbonyl or Guanidinium carbon (RBT::SP2)

MASS	190 C3	12.01100 C ! Carbonyl carbon in 3-membered aliphatic ring (RBT::SP2)

MASS	192 C4	12.01100 C ! Carbonyl carbons in 4-membered aliphatic ring (RBT::SP2)

MASS	21 C5R	12.01100 C ! Aromatic carbon in a five membered ring (RBT::SP2)

MASS	23 C5RE 13.01900 C ! Extended aromatic carbon in five membered ring (RBT::SP2)

MASS	28 C5RP 12.01100 C ! for Aryl-Aryl bond between C5R rings (RBT::SP2)

MASS	189 C5RQ 12.01100 C ! for second Ar-Ar bond between C5RP rings (ortho) (RBT::SP2)

MASS	22 C6R	12.01100 C ! Aromatic carbon in a six membered ring (RBT::SP2)

MASS	24 C6RE 13.01900 C ! Extended aromatic carbon in six membered ring (RBT::SP2)

MASS	29 C6RP 12.01100 C ! for Aryl-Aryl bond between C6R rings (RBT::SP2)

MASS	194 C6RQ 12.01100 C ! Carbon of C6RP type ortho to C6RP pair (RBT::SP2)

MASS	230 CF1	12.01100 C ! Carbon with one Fluorine (RBT::SP3)

MASS	231 CF2	12.01100 C ! Carbon with two Fluorines (RBT::SP3)

MASS	232 CF3	12.01100 C ! Carbons with three Fluorines (RBT::SP3)

MASS	11 CH1E 13.01900 C ! Extended atom carbon with one hydrogen (RBT::SP3)

MASS	12 CH2E 14.02700 C ! Extended atom carbon with two hydrogens (RBT::SP3)

MASS	13 CH3E 15.03500 C ! Extended atom carbon with three hydrogens (RBT::SP3)

MASS	15 CM	12.01100 C ! Carbonmonoxide, or other triply bonded, carbon (RBT::SP)

MASS	199 CP3	12.01100 C ! Carbon on nitrogen in proline ring (RBT::SP3)

MASS	197 CPH1 12.01100 C ! CG and CD2 carbons in histidine ring (RBT::SP2)

MASS	198 CPH2 12.01100 C ! CE1 carbon in histidine ring (RBT::SP2)

MASS	195 CQ66 12.01100 C ! Third adjacent pair of CR66 types in fused rings (RBT::SP2)

MASS	25 CR55 12.01100 C ! Aromatic carbon-merged five membered rings (RBT::SP2)

MASS	26 CR56 12.01100 C ! Aromatic carbon-merged five/six membered rings (RBT::SP2)

MASS	27 CR66 12.01100 C ! Aromatic carbon-merged six membered rings (RBT::SP2)

MASS	196 CS66 12.01100 C ! Second adjacent pair of CR66 types in fused rings (RBT::SP2)

MASS	10 CT	12.01100 C ! Aliphatic carbon (tetrahedral) (RBT::SP3)

MASS	191 CT3	12.01100 C ! in 3-membered aliphatic ring, usually tetrahedral (RBT::SP3)

MASS	193 CT4	12.01100 C ! in 4-membered aliphatic ring, usually tetrahedral (RBT::SP3)

MASS	16 CUA1 12.01100 C ! Carbon in double bond, first pair (RBT::SP2)

MASS	17 CUA2 12.01100 C ! Carbon in double bond, second conjugated pair (RBT::SP2)

MASS	20 CUA3 12.01100 C ! Carbon in double bond, third conjugated pair (RBT::SP2)

MASS	18 CUY1 12.01100 C ! Carbon in triple bond, first pair (RBT::SP)

MASS	19 CUY2 12.01100 C ! Carbon in triple bond, second conjugated pair (RBT::SP)

MASS	1 H	1.00800 H ! Hydrogen bonding hydrogen (neutral group) (RBT::SP3)

MASS	3 HA	1.00800 H ! Aliphatic or aromatic hydrogen (RBT::SP3)

MASS	2 HC	1.00800 H ! Hydrogen bonding hydrogen (charged group) (RBT::SP3)

MASS	9 HMU	1.00800 H ! Mu-bonded hydrogen for metals and boron-hydride (RBT::SP3)

MASS	8 HO	1.00800 H ! Hydrogen on an alcohol oxygen (RBT::SP3)

MASS	4 HT	1.00800 H ! TIPS3P water model hydrogen (RBT::SP3)

MASS	31 N	14.00670 N ! Nitrogen; planar-valence of 3, i.e. nitrile, etc. (RBT::TRI)

MASS	234 N3	14.00670 N ! Nitrogen in a three membered ring (RBT::SP3)

MASS	34 N5R	14.00670 N ! Nitrogen in a five membered aromatic ring (RBT::TRI)

MASS	30 N5RP 14.00670 N ! for Ar-Ar bond between five membered rings (RBT::TRI)

MASS	35 N6R	14.00670 N ! Nitrogen in a six membered aromatic ring (RBT::SP2)

MASS	39 N6RP 14.00670 N ! for Aryl-Aryl bond between six membered rings (RBT::SP2)

MASS	37 NC	14.00670 N ! Charged guanidinium-type nitrogen (RBT::TRI)

MASS	186 NC2	14.00670 N ! for neutral guanidinium group - Arg sidechain (RBT::SP2)

MASS	38 NO2	14.00670 N ! Nitrogen in nitro, or related, group (RBT::TRI)

MASS	32 NP	14.00670 N ! Nitrogen in peptide, amide, or related, group (RBT::TRI)

MASS	183 NR1	14.00670 N ! Protonated nitrogen in neutral histidine ring (RBT::TRI)

MASS	184 NR2	14.00670 N ! Unprotonated nitrogen in neutral histidine ring (RBT::SP2)

MASS	185 NR3	14.00670 N ! Nitrogens in charged histidine ring (RBT::SP2)

MASS	182 NR55 14.00670 N ! N at fused bond between two 5-membered aromatics (RBT::SP2)

MASS	180 NR56 14.00670 N ! N at fused bond between 5 and 6-membered aryls (RBT::SP2)

MASS	181 NR66 14.00670 N ! N at fused bond between two 6-membered aromatics (RBT::SP2)

MASS	36 NT	14.00670 N ! Nitrogen (tetrahedral), i.e. Amine, etc. (RBT::SP3)

MASS	33 NX	14.00670 N ! Proline nitrogen, or similar (RBT::TRI)

MASS	40 O	15.99940 O ! Carbonyl oxygen for amides, or related structures (RBT::SP2)

MASS	56 O2M	15.99940 O ! Oxygen in Si-O-Al or Al-O-Al bond (RBT::SP3)

MASS	52 O5R	15.99940 O ! Oxygen in 5 membered aromatic ring-radicals, etc. (RBT::SP3)

MASS	53 O6R	15.99940 O ! Oxygen in 6 membered aromatic ring-radicals, etc. (RBT::SP3)

MASS	41 OA	15.99940 O ! Carbonyl oxygen for aldehydes, or related (RBT::SP2)

MASS	51 OAC	15.99940 O ! Carbonyl oxygen for acids, or related (RBT::SP2)

MASS	43 OC	15.99940 O ! Charged oxygen (RBT::SP3)

MASS	50 OE	15.99940 O ! Ether oxygen / Acetal oxygen (RBT::SP3)

MASS	47 OH2	15.99940 O ! ST2 water model oxygen (RBT::SP3)

MASS	42 OK	15.99940 O ! Carbonyl oxygen for ketones, or related (RBT::SP2)

MASS	48 OM	15.99940 O ! Carbonmonoxide, or other triply bonded, oxygen

MASS	49 OS	15.99940 O ! Ester oxygen (RBT::TRI)

MASS	57 OSH	0.00000 O ! Massless O for zeolites, or related cage cmpds.

MASS	55 OSI	15.99940 O ! Oxygen in Si-O-Si bond (RBT::SP3)

MASS	45 OT	15.99940 O ! Hydroxyl oxygen (tetrahedral) or Ionizable acid (RBT::SP3)

MASS	46 OW	15.99940 O ! TIP3P water model oxygen (RBT::SP3)

MASS	64 P6R	30.9738 P ! Phosphorous in aromatic 6-membered ring (RBT::SP3)

MASS	61 PO3	30.9738 P ! Phosphorous bonded to three oxygens (RBT::SP2)

MASS	62 PO4	30.9738 P ! Phosphorous bonded to four oxygens (RBT::SP2)

MASS	60 PT	30.9738 P ! Phosphorous, general; usually tetrahedral (RBT::SP3)

MASS	63 PUA1 30.9738 P ! Phosphorous double bond

MASS	233 PUY1 30.9738 P ! Triple bonded phosphorus

MASS	72 S5R	32.0600 S ! Sulphur in a five membered aromatic ring (RBT::SP3)

MASS	73 S6R	32.0600 S ! Sulphur in a six membered aromatic ring (RBT::SP3)

MASS	74 SE	32.060	S ! Thioether sulphur (RBT::SP3)

MASS	71 SH1E 33.06800 S ! Extended atom sulphur with one hydrogen (RBT::SP3)

MASS	75 SK	32.060	S ! Thioketone sulphur (RBT::SP2)

MASS	76 SO1	32.0600 S ! Sulphur bonded to one oxygen (RBT::SP3)

MASS	77 SO2	32.0600 S ! Sulphur bonded to two oxygens (RBT::SP3)

MASS	78 SO3	32.0600 S ! Sulphur bonded to three oxygens (RBT::SP2)

MASS	79 SO4	32.0600 S ! Sulphur bonded to four oxygens (RBT::SP2)

MASS	70 ST	32.06000 S ! Sulphur, general; usually tetrahedral (RBT::SP3)

MASS	94 XBR	79.904	Br ! Bromine

MASS	93 XCL	35.45300 Cl ! Chlorine

MASS	92 XF	18.99840 F ! Fluorine

MASS	95 XI	126.9045 I ! Iodine

MASS	91 MAL	26.9815 Al ! ALuminum

MASS	6 MBE	9.01218 Be ! Beryllium

MASS	84 MCA	40.080	Ca ! Calcium

MASS	83 MK	39.098	K ! Potassium

MASS	80 MLI	6.941	Li ! Lithium

MASS	82 MMG	24.305	Mg ! Magnesium

MASS	81 MNA	22.9898 Na ! Sodium

MASS	90 MSI	28.0855 Si ! Silicon

MASS	177 MSIU 28.08550 Si ! Silicon when as double bond

MASS	86 MFE	55.85	Fe ! Iron

MASS	87 MZN 65.3800	Zn ! Zinc (Added DM 30/7/2001)

* 4 Jan 1999 (DM) Added old types from

* /darius/dsk1/progs/insightII/insight97/data/xplor/toppar/topallhdg.pro

* just to keep rbscore happy. The numeric force field types are ficticious

* and will not be recognised by Charmm

MASS	301 CX	12.011	C ! (RBT::SP2)

MASS	302 CW	12.011	C ! (RBT::SP2)

MASS	303 CN	12.011	C ! (RBT::SP2)

MASS	304 CA	12.011	C ! (RBT::SP2)

MASS	305 CB	12.011	C ! (RBT::SP2)

MASS	306 CC	12.011	C ! (RBT::SP2)

MASS	307 CV	12.011	C ! (RBT::SP2)

MASS	308 CR	12.011	C ! (RBT::SP2)

MASS	309 CP	12.011	C ! Proline carbon CG,CD (RBT::SP3)

MASS	310 NA	14.007	N ! (RBT::TRI)

MASS	311 NB	14.007	N ! (RBT::TRI)

MASS	312 NH1	14.007	N ! (RBT::TRI)

MASS	313 NH2	14.007	N ! (RBT::TRI)

MASS	314 NH3	14.007	N ! (RBT::SP3)

MASS	315 OH	15.999	O ! (RBT::SP3)

MASS	316 S	32.060	S ! (RBT::SP3)

* 4 Jan 1999 (DM) Added old types from

* /darius/dsk1/progs/insightII/insight97/data/xplor/toppar/topallhdg.dna

* just to keep rbscore happy. The numeric force field types are ficticious

* and will not be recognised by Charmm

MASS	400 H2	1.00800 H ! (RBT::SP3)

MASS	401 CH	12.01100 C ! (RBT::SP3)

MASS	402 C2	12.01100 C ! (RBT::SP3)

MASS	403 CE	12.01100 C ! (RBT::SP2)

MASS	404 CF	12.01100 C ! (RBT::SP2)

MASS	405 CS	12.01100 C ! (RBT::SP2)

MASS	406 N2	14.00670 N ! (RBT::TRI) Corrected DM 23/8/1999

MASS	407 NS	14.00670 N ! (RBT::TRI)

MASS	408 NH2E 16.02270 N ! (RBT::SP3)

MASS	409 O2	15.99940 O ! (RBT::SP3)

MASS	411 SD	22.98980 Na !

MASS	412 P	30.97400 P ! (RBT::SP3)

MASS	413 BR	79.90400 Br !

MASS	414 MG	24.30500 Mg !

* 8 Jun 1999 (DM) Added types to keep wtTARrbt158.psf happy

MASS	450 HE	1.00800 H ! (RBT::SP3)

* 18 Jan 2001 (DM) Dummy atom type

MASS	666 DUM	0.00000 DU ! Dummy atom

* Charmm 27 additions

MASS	5 H	1.00800 H ! Nonpolar H prom charmm27 all-atom FF (RBT::SP3)

MASS	54 HG	1.00800 H ! Nonpolar H prom charmm27 all-atom FF (RBT::SP3)

MASS	58 NE	14.00670 N ! charmm27 N

RESI XXXX 0.0

END
